# Genome-Wide Population-Based Association Study of Extremely Overweight Young Adults – The GOYA Study

**DOI:** 10.1371/journal.pone.0024303

**Published:** 2011-09-15

**Authors:** Lavinia Paternoster, David M. Evans, Ellen Aagaard Nohr, Claus Holst, Valerie Gaborieau, Paul Brennan, Anette Prior Gjesing, Niels Grarup, Daniel R. Witte, Torben Jørgensen, Allan Linneberg, Torsten Lauritzen, Anelli Sandbaek, Torben Hansen, Oluf Pedersen, Katherine S. Elliott, John P. Kemp, Beate St. Pourcain, George McMahon, Diana Zelenika, Jörg Hager, Mark Lathrop, Nicholas J. Timpson, George Davey Smith, Thorkild I. A. Sørensen

**Affiliations:** 1 MRC CAiTE centre, University of Bristol, Bristol, United Kingdom; 2 School of Social and Community Medicine, University of Bristol, Bristol, United Kingdom; 3 Institute of Public Health, Aarhus University, Aarhus, Denmark; 4 Institute of Preventive Medicine, Copenhagen University Hospitals, Copenhagen, Denmark; 5 International Agency for Research on Cancer (IARC), Lyon, France; 6 The Novo Nordisk Center for Metabolic Research, Faculty of Health Sciences, University of Copenhagen, Copenhagen, Denmark; 7 Faculty of Health Sciences, University of Southern Denmark, Odense, Denmark; 8 Steno Diabetes Center, Copenhagen, Denmark; 9 Research Centre for Prevention and Health, Glostrup University Hospital, Glostrup, Denmark; 10 Faculty of Health Science, University of Copenhagen, Copenhagen, Denmark; 11 Department of General Practice, University of Aarhus, Aarhus, Denmark; 12 Institute of Biomedical Science, Faculty of Health Science, University of Copenhagen, Copenhagen, Demark; 13 Faculty of Health Science, University of Aarhus, Aarhus, Denmark; 14 Wellcome Trust Centre for Human Genetics, Oxford, United Kingdom; 15 Centre National de Génotypage, Evry, France; 16 Foundation Jean Dausset, CEPH, Paris, France; University of Montreal, Canada

## Abstract

**Background:**

Thirty-two common variants associated with body mass index (BMI) have been identified in genome-wide association studies, explaining ∼1.45% of BMI variation in general population cohorts. We performed a genome-wide association study in a sample of young adults enriched for extremely overweight individuals. We aimed to identify new loci associated with BMI and to ascertain whether using an extreme sampling design would identify the variants known to be associated with BMI in general populations.

**Methodology/Principal Findings:**

From two large Danish cohorts we selected all extremely overweight young men and women (n = 2,633), and equal numbers of population-based controls (n = 2,740, drawn randomly from the same populations as the extremes, representing ∼212,000 individuals). We followed up novel (at the time of the study) association signals (p<0.001) from the discovery cohort in a genome-wide study of 5,846 Europeans, before attempting to replicate the most strongly associated 28 SNPs in an independent sample of Danish individuals (n = 20,917) and a population-based cohort of 15-year-old British adolescents (n = 2,418). Our discovery analysis identified SNPs at three loci known to be associated with BMI with genome-wide confidence (P<5×10^−8^; *FTO*, *MC4R* and *FAIM2*). We also found strong evidence of association at the known *TMEM18*, *GNPDA2*, *SEC16B*, *TFAP2B*, *SH2B1* and *KCTD15* loci (p<0.001), and nominal association (p<0.05) at a further 8 loci known to be associated with BMI. However, meta-analyses of our discovery and replication cohorts identified no novel associations.

**Significance:**

Our results indicate that the detectable genetic variation associated with extreme overweight is very similar to that previously found for general BMI. This suggests that population-based study designs with enriched sampling of individuals with the extreme phenotype may be an efficient method for identifying common variants that influence quantitative traits and a valid alternative to genotyping all individuals in large population-based studies, which may require tens of thousands of subjects to achieve similar power.

## Introduction

Genome-wide association (GWA) studies have successfully identified genetic loci associated with body mass index (BMI) [1]–[3]. Despite the very large sample-sizes employed in these studies (>120,000 in the most recent), most of the heritability has yet to be explained, with the 32 confirmed loci accounting for only ∼1.45% of the variance in BMI (2–4% of the genetic variance) [3].

Given the difficulty in identifying loci responsible for small proportions of the phenotypic variance in BMI, a number of strategies have been proposed to increase the power to detect association. One suggestion has been to selectively genotype individuals at either one or both ends of the distribution of BMI scores (i.e. obese and/or extremely lean individuals) [4]–[7]. The rationale is that individuals taken from the extreme ends of the sample are more likely to be enriched for alleles of interest than individuals sampled from the middle of the distribution. Theoretically, under simple models of many variants of small effect, this selection strategy should markedly increase power to detect association relative to a similar size sample of unselected individuals. Case individuals can be considered, under certain assumptions, to reflect extreme scores on an underlying continuous distribution of disease liability, which is the primary reason why case-control studies are expected to have greater power to detect loci than the same number of individuals selected randomly from a population cohort.

Less well appreciated is that under certain scenarios, selecting extreme individuals will not always increase power to detect common genetic variants [8]. One reason is that some individuals exhibiting extreme trait values may carry rare alleles of large effect, rather than reflecting the normal variation from common alleles at quantitative trait loci. Consequently, an extreme sample may not be enriched for common alleles of interest. Whilst rare alleles will also be of interest, it may be difficult to identify them via genome-wide association, since commercial SNP chips have limited ability to tag rare variants [9] and the power to detect rare variants via genetic association is low in general. In addition, individuals may exhibit extreme BMI because of non-genetic factors or rare combinations of gene-gene or gene-environment interactions, all of which may also decrease power to detect common variants of small effect. Thus if the extremes have risk factors that are unique from the general distribution of BMI, such a study design may not be useful for identifying general population BMI risk alleles.

Three previous GWA studies have employed an extreme sampling strategy to detect obesity loci. One study, which compared gastric bypass surgery patients with population controls, found no novel loci, but reported that 6 of the 12 BMI variants known at the time were associated with risk of severe obesity, suggesting that generally obesity represents the extreme of a phenotypic spectrum, rather than a distinct condition [10]. Another study, sampled early-onset obese children and morbidly obese (BMI>40 kg/m^2^) adults, and compared them to normal weight controls [11]. As well as identifying variants in *FTO* and *MC4R*, they detected a further three loci associated with obesity (*NPC1*, *MAF* and *PTER*). Another, aimed to identify variants associated with early-onset extreme obesity [12]. They detected the known *FTO*, *MC4R* and *TMEM18* loci as well as two novel loci (*SDCCAG8* and *TNKS/MSRA*). However, the authors of these three studies analysed the data using a dichotomous coding (i.e. obese vs non-obese), effectively discarding information from the underlying continuous distribution of BMI values. Since case-control methods ignore the underlying BMI trait values, they do not make complete use of the data, and are therefore inefficient and likely to be less powerful than approaches that incorporate quantitative information [13].

In this study we aimed to investigate the genetic profile at the upper extreme of the BMI distribution using a population sample enriched for these individuals. We employed a genome-wide association approach including a large sample of 2,633 extremely overweight young Danish adults from the top 1% (males) and 4% (females) of the distribution of BMI scores, and an approximately equal number of control individuals (randomly selected from the same populations that the extremely overweight individuals were identified in), whilst retaining the quantitative BMI information of these individuals in the analysis. Our aims were twofold: (i) to detect new variants associated with obesity and more generally, BMI; and (ii) to ascertain whether an extreme sampling design could be used to powerfully detect BMI variants previously identified using population based samples.

## Methods

**Figure 1:**
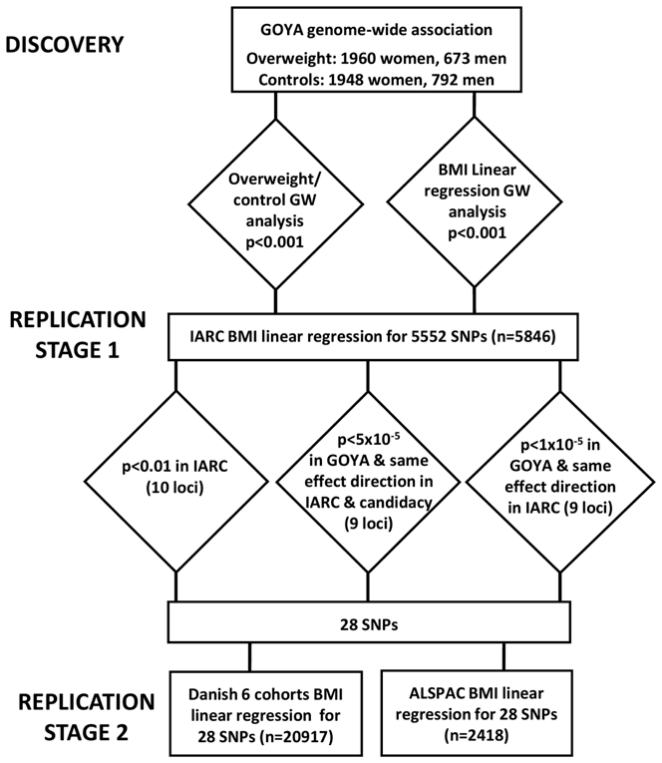
Discovery and replication process. GOYA = Genetics of Overweight Young Adults. IARC = International Agency for Research on Cancer. ALSPAC = Avon Longitudinal Study of Parents and Children.

**Figure 1** depicts the flow of analysis and SNP selection through discovery and replication stages carried out in this study.

### GOYA discovery analysis

#### GOYA participants

We employed a case-cohort sampling design in which cases were defined as the individuals in a cohort with the upper-most extreme BMI scores and the controls selected by random sampling from the same cohort. Two different cohorts were used, the Danish National Birth Cohort for women and a draft board examination cohort for men, both of which were constituted by young Danish adults with negligible admixture of other ethnicities.

In total, 91,387 pregnant women were recruited to the Danish National Birth Cohort during 1996–2002. The study is described in detail elsewhere [14], but pertinent to this study, pre-pregnancy BMI was calculated from self-reported height and weight collected during a telephone interview at ∼16 weeks of gestation. We included in the current study the 67,853 women who had given birth to a live born infant, had provided a blood sample during pregnancy and had BMI information available [14]. We selected the 3.6% of these women with the largest residuals from the regression of BMI on age and parity (all entered as continuous variables). The BMI for these 2451 women ranged from 32.6 to 64.4. From the remaining cohort we selected a random sample of similar size (2450). Of these, 1,960 extremely overweight and 1,948 control women were genotyped (and passed quality control (QC), 102 failed QC). With a sampling fraction of 3.6%, these controls represent about 54,000 women.

A randomly selected control group of one in every hundred men (n = 3,601) and all extremely overweight men (n = 1,930) were identified from the records of 362,200 Caucasian men examined at the mean age of 20 years at the draft boards in Copenhagen and its surroundings during 1943–77. Standing height (without shoes) and weight (in underwear only) were measured at the draft board examinations. Obesity was defined as 35% overweight relative to a local standard in use at the time (mid 1970's), corresponding to a BMI ≥31.0 kg/m^2^, which proved to be above the 99th percentile. All extremely overweight men and a random sample of half the men, who were still living in the region, were invited to a follow-up survey in 1992–94 at the mean age of 46 years, at which time the blood samples were taken (753 extremely overweight and 879 control men attended). The criteria for invitation to the follow-up surveys and participation have been described previously [15], [16]. Of these, 673 extremely overweight and 792 control men were genotyped (and passed quality control (QC), 61 failed QC). With a sampling fraction of 0.5% (50% of 1%), these controls represent about 158,000 men among whom the case group was the most obese.

The study was approved by the regional scientific ethics committee and by the Danish Data Protection Board.

#### Genotyping

Genome-wide genotyping on the Illumina 610 k quad chip was carried out at the Centre National de Génotypage (CNG), Evry, France. We excluded SNPs with minor allele frequency <1%, >5% missing genotypes or which failed an exact test of Hardy-Weinberg equilibrium (HWE) in the controls (p<10^−7^). We also excluded any individual who did not cluster with the CEU individuals (Utah residents with ancestry from northern and western Europe) in a multidimensional scaling analysis seeded with individuals from the International HapMap release 22 (22 individuals) (Figure S1 - there was little evidence for additional population stratification as shown by the GWAS lambdas: 1.05 and 1.06), who had >5% missing data (5 individuals), outlying heterozygosity of >35% or <30.2% (35 individuals), both samples in the case of genetic duplicates (4 individuals), one of each pair of genetically related individuals (50 individuals), 4 individuals with sex discrepancies and one individual whose genotyping was discordant with a previous project. After data cleaning, 5,373 individuals (2,633 extremely overweight and 2,740 random controls) and 545,349 SNPs remained. We carried out imputation to HapMap release 22 (CEU individuals) using Mach 1.0, Markov Chain Haplotyping [17].

#### Genome-wide association analysis

We analysed only those imputed SNPs, which had a minor allele frequency >0.01 and an R^2^ imputation quality score >0.3. We carried out genome-wide association analysis using two analysis methods.

We first regressed overweight/control status on expected genotype dosage (adjusting for age and sex) using the software package MACH2DAT, which accounts for uncertainty in prediction of the imputed data by weighting genotypes by their posterior probabilities [17]. However, it is possible that this method does not make optimal use of the data in that it ignores quantitative information within the extreme and central parts of the BMI distribution.

We therefore also analysed BMI as a quantitative trait using linear regression of sex-specific BMI z-scores on genotype, including age as a covariate (in STATA). Z-scores were constructed using the mean and standard deviation of the randomly selected normal weight control group. Though the p-value from such an analysis is not biased, the estimated regression coefficient for BMI on genotype is biased because of the non-normality of BMI with the case-cohort sampling design and we therefore only report the p-value and direction of effect (sign of beta coefficient) for this analysis. Genomic inflation was measured by λ = median(chi^2^)/0.456.

### Stage 1 replication

**Figure 2:**
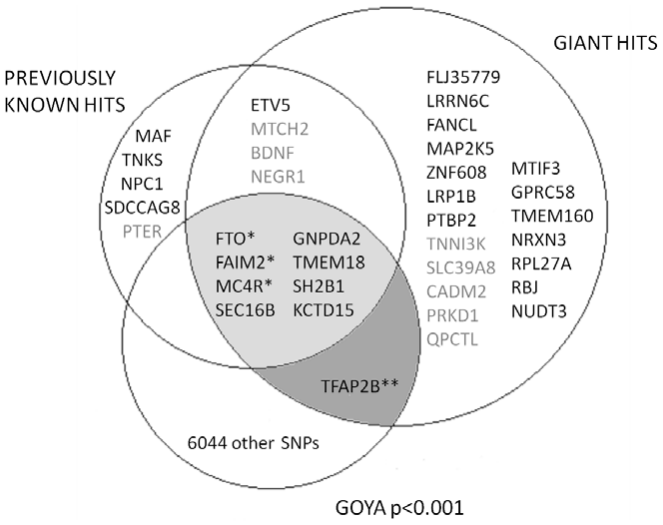
The overlap of previously known BMI hits, GIANT BMI hits and GOYA results. Shown are the loci with p<0.0001 in the current GOYA study (that were taken through to replication), the previously known BMI hits [1], [2], [11] and the recent GIANT BMI hits [3]. The light grey shaded area shows the loci which had p<0.001 in GOYA, but were excluded from replication because there was already strong evidence for an association with BMI and the dark grey shaded area shows the TFAP2B loci which we included in our replication stage (as it had not been identified in previous studies) and found genome-wide significant evidence for its association with BMI, and since our replication stage it has been identified as associated with BMI in the GIANT study. The grey previously associated loci had p<0.05 in GOYA. *p<5×10−8 in GOYA discovery analysis, ** p<0.001 in GOYA discovery & replication meta-analysis.

We selected SNPs for in silico replication if they had a p<0.001 in either the overweight/control or BMI z-score linear regression GWAS, but excluded SNPs from any region where there was a previously confirmed association with BMI or obesity at the time of the study (i.e. *FTO*, *FAIM2*, *MC4R*, *SEC16B*, *GNPDA2*, *KCTD15*, *TMEM18*, *SH2B1*, *NEGR1*, *ETV5*, *BDNF*, *MTCH2* regions, **Figure 2**) [1], [2], [11].

Stage 1 replication was conducted using data available from the International Agency for Research on Cancer (IARC). Full details of the participants, genotyping and analysis are in **Text S1**. The data were analysed using linear regression of the inverse rank normal BMI, adjusted for age, sex and country.

#### GeneSniffer

We used GeneSniffer (www.genesniffer.org) to prioritize genes within the LD vicinity (+/−0.1 cM) of each of the discovery associated SNPs (p<5×10^−5^) for trait candidacy by assigning a relevance score (full details available in **Text S1**).

### Stage 2 replication

Following the in silico replication we took the best SNPs forward to replication genotyping in several Danish cohorts and in silico replication in the ALSPAC cohort.

SNPs for the final stage of replication were selected according to three strategies. First, the top SNP from each region which showed moderate evidence of association (i.e. p<0.001) from the z-score linear regression analysis of BMI in the discovery cohort and also displayed nominal evidence of replication in the IARC cohort (i.e. p<0.01 in the same direction) were selected for further replication. Secondly, the top SNP was taken from any region with p<1×10^−5^ in either of the two discovery cohort analyses and with the same direction of effect in the IARC replication sample. Lastly, the top SNP from any region with p<5×10^−5^ in either of the two discovery cohort analyses, with the same direction of effect in the IARC replication sample and with evidence of candidacy (as assessed by GeneSniffer or from other published evidence).

Stage 2 replication was carried out in a set of Danish cohorts and the Avon Longitudinal Study of Parents and Children (ALSPAC). Full details on the participants, genotyping and analysis are in **Text S1** and **Table S1**. The data were analysed by linear regression of sex-specific BMI z-score on genotype, adjusting for age and EIGENSTRAT values, as appropriate.

### Meta-analysis

To estimate an overall p-value for each of the SNPs, we performed an unweighted Stouffer's Z-transform meta-analysis (inputting the p-value and direction of effect from each study to construct a directional z-score and applying equal weights to each study to generate an overall Z-score and p-value) on all participating cohorts in METAL (http://www.sph.umich.edu/csg/abecasis/Metal/) using the analysis from each cohort which analysed z-score BMI as a continuous trait (we re-analysed the IARC data using the same methods as the other cohorts - linear regression of BMI z-score on genotype, adjusting for age).

## Results

### Discovery cohorts

Whilst the GOYA men were selected from a much larger cohort and the overweight group was more extreme, the GOYA women had higher average BMI (1.2 kg/m^2^ higher in the controls and 3.8 kg/m^2^ higher in the overweight group) (**Table 1**), which can be attributed to the fact that they were sampled later in the development of the obesity epidemic and that they were older than the men. However, the pattern of association looked similar across the two genders, so the two groups were analysed together assuming that the genotype-phenotype associations across the entire phenotype distribution of the two populations were the same (data not shown).

**Table 1:** Summary characteristics of the GOYA study participants.

	Men		Women	
	OverweightN = 673	NormalN = 792	OverweightN = 1960	NormalN = 1948
Age, years	19 (18–24)	19 (18–24)	29 (22–36)	29 (22–36)
Height, cm	177 (167–188)	177 (166–188)	168 (158–178)	169 (160–180)
Weight, kg	104 (91–124)	67 (55–83)	103 (89–128)	64 (51–86)
BMI, kg/m^2^	32.5 (31.6–34.1)	21.3 (18.4–24.7)	36.3 (33.8–43.9)	22.5 (18.6–30.1)

Values are median (5–95 percentile range).

**Figure 3:**
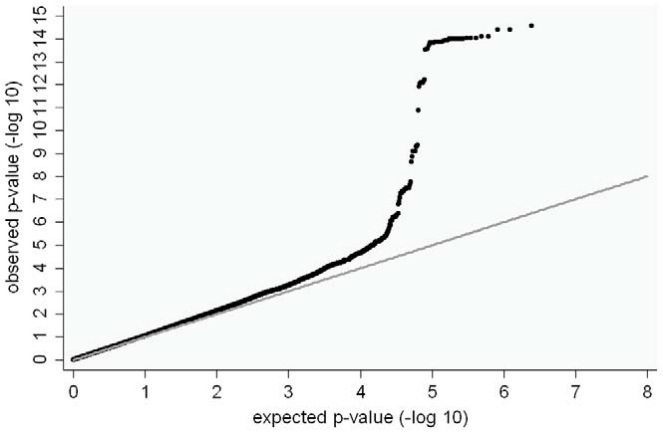
QQ plot for the GOYA BMI z-score linear regression genome-wide analysis. Lambda = 1.057.

**Figure 4:**
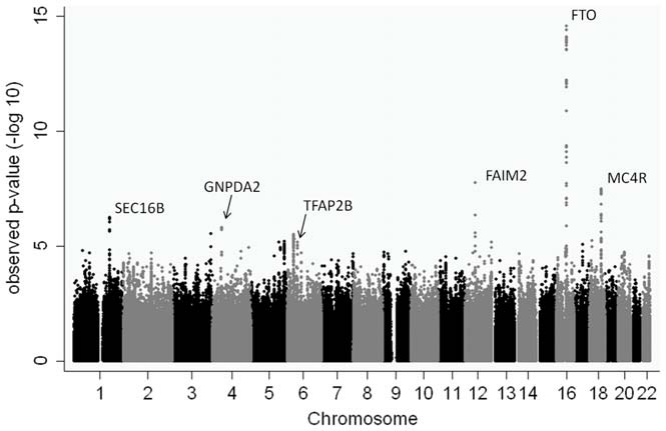
Manhattan Plot for the GOYA BMI z-score linear regression genome-wide analysis.

The QQ plots for the genome-wide analyses in the discovery cohort for the two analysis methods were broadly similar and demonstrated little evidence of systematic inflation from expectation. Lambda values for BMI z-score linear regression and overweight/control analyses were consistent with this observation: λ = 1.06 and λ = 1.05 respectively). Despite this, there was evidence for favourable departure of test metrics from the null expectation towards the extremes (**Figure 3** & **Figure S2**). Specifically, three regions showed p-values with considerable genome-wide evidence against the null hypothesis (defined as p<5×10^−8^) (**Table 2**). These three regions (*FTO*, *FAIM2* and *MC4R*) have previously been associated with BMI and other related traits [1], [2] (**Figure 2**). The next two most strongly associated regions (p<1×10^−5^) in our analyses (*SEC16B* and *GNPDA2*) have also been associated with BMI previously [1], [2]. In total 22 regions had p<1×10^−5^ in the overweight/control or the BMI z-score linear regression analysis (**Table 2**, **Figure 4**, **Figure S3**).

**Table 2:** Top SNP per region for all GOYA hits p<1×10^−5^ in either the overweight/control dichotomous analysis or the quantitative BMI linear regression ananlysis.

							Overweight/control analysis			BMI linear regression analysis		
chr	position	MARKER	EFFECT ALLELE	OTHER ALLELE	EFFECT ALLELE FREQ	R^2^	OR	95% CI	p	beta§	p	known genes
16	52376670	rs9936385	C	T	0.42	0.94	1.35	1.25,1.46	1.4E-13*	+	2.8E-1* ^/^ **	*FTO*
12	48549415	rs7132908	A	G	0.44	0.98	1.26	1.17,1.37	6.1E-09*	+	1.8E-08* ^/^ **	*FAIM2*
18	56009809	rs8089364	C	T	0.30	0.99	1.23	1.14,1.34	7.20E-07	+	3.2E-08* ^/^ **	*MC4R*
1	176144602	rs604388	C	T	0.36	1	1.21	1.12,1.31	2.20E-06	+	5.80E-07*	*SEC16B*
4	44870448	rs13130484	C	T	0.58	1	0.85	0.78,0.91	1.90E-05	−	1.60E-06*	*GNPDA2*
3	191585563	rs17504169	A	G	0.15	0.99	1.23	1.11,1.37	1.40E-04	+	2.80E-06	
6	33183421	rs910320	C	T	0.77	0.98	0.84	0.77,0.92	2.30E-04	−	3.20E-06	
18	10426288	rs2115706	A	G	0.63	0.81	1.20	1.1,1.31	5.50E-05	+	6.00E-06	
5	164598808	rs7720663	A	G	0.60	1	1.16	1.07,1.25	2.30E-04	+	6.30E-06	
12	130882177	rs4964926	C	G	0.91	0.85	0.73	0.63,0.85	4.00E-05	−	6.60E-06	
5	138008375	rs3849047	C	T	0.67	0.88	1.18	1.08,1.29	1.30E-04	+	6.90E-06	
6	50944238	rs734597	A	G	0.17	0.98	1.25	1.13,1.39	2.00E-05	+	7.00E-06	
17	41439785	rs8067056	C	T	0.36	0.95	1.19	1.09,1.29	4.30E-05	+	8.30E-06	
16	51179613	rs11640537	A	G	0.19	1	1.20	1.09,1.33	1.70E-04	+	9.90E-06	
5	145822662	rs17104665	A	G	0.93	1	1.41	1.22,1.64	4.50E-06	+	1.20E-05	
9	114439415	rs2798311	C	T	0.95	0.64	1.71	1.36,2.14	3.80E-06	+	1.80E-05	
11	38745191	rs11034952	C	T	0.57	1	1.19	1.11,1.29	5.60E-06	+	3.10E-05	
14	21857530	rs10047878	A	T	0.54	0.99	0.83	0.77,0.90	2.60E-06	−	4.80E-05	
9	128502322	rs10987417	G	T	0.61	0.92	0.83	0.76,0.90	5.10E-06	−	5.10E-05	
14	31842078	rs6571507	A	G	0.37	0.99	1.20	1.11,1.29	7.70E-06	+	7.70E-05	
2	17902293	rs6758546	A	G	0.10	1	0.75	0.66,0.85	9.20E-06	−	8.80E-05	
2	23392348	rs1002158	G	T	0.73	1	0.82	0.75,0.89	6.80E-06	−	1.20E-04	

*regions with previous evidence for association with obesity.

**genome-wide significant p<5×10^−8^.

§ only the sign (+/−) of the beta coefficients are shown for the BMI linear regression analysis, as the extreme sampling strategy will produce biased beta coefficients.

R^2^ is the MACH derived imputation quality score. OR denotes odds ratio, CI denotes confidence interval, ‘beta’ for the BMI linear regression analysis denotes the sign of the coefficient.

Nine of the 32 variants associated with BMI in the recent GIANT GWA study [3] had p<0.001 (and an effect in the same direction) in our in BMI z-score linear regression analysis (**Table 3**, **Figure 2**) and a total of seventeen out of 32 had p<0.05 (and an effect in the same direction) (**Table 3**). In contrast, 15 of the GIANT BMI-associated genes failed to show evidence of association (i.e. p>0.05) in our discovery cohort, although all but one showed an effect in the same direction. We found p<0.05 (and an effect in the same direction) for one of the three loci (*PTER*) identified in the Meyre et al. extremes scan [11]. There was no evidence in our study for association with the other two loci (*MAF* and *NPC1*) from the Meyre et al. paper or with the two loci (*SDCCAG8* and *TNKS/MSRA*) from the Scherag et al paper [12] (p>0.05 and effects in the opposite direction to the original studies). The majority of known SNPs had lower p-values in the BMI z-score linear regression analysis than the analysis of overweight versus normal individuals, although in most cases the difference was relatively small (i.e. less than an order of magnitude) (**Table 3**).

**Table 3:** GOYA result for previously identified BMI loci.

GIANT										GOYA				
Gene	Chr	SNP	position	effect	other	freq effect	p	per allele change in BMI	SE	BMI p	beta	obesity p	OR	95% CI
*FTO*	16	rs1558902	52,361,075	a	t	0.42	4.80E-120	0.39	0.02	3.90E-15*	+	4.90E-14*	1.34	1.24,1.45
*TMEM18*	2	rs2867125	612,827	c	t	0.83	2.80E-49	0.31	0.03	2.20E-04*	+	4.20E-04*	1.21	1.09,1.34
*MC4R*	18	rs571312	55,990,749	a	c	0.24	6.40E-42	0.23	0.03	4.90E-07*	+	4.60E-06*	1.22	1.12,1.33
*GNPDA2*	4	rs10938397	44,877,284	g	a	0.43	3.80E-31	0.18	0.02	2.00E-06*	+	2.20E-05*	1.18	1.10,1.28
*BDNF*	11	rs10767664	27,682,562	a	t	0.78	4.70E-26	0.19	0.03	0.018*	+	0.139	1.07	0.98,1.18
*SEC16B*	1	rs543874	176,156,103	g	a	0.19	3.60E-23	0.22	0.03	2.20E-05*	+	6.50E-05*	1.20	1.10,1.32
*NEGR1*	1	rs2815752	72,585,028	a	g	0.61	1.60E-22	0.13	0.02	0.01*	+	0.04*	1.08	1.00,1.17
*RBJ*	2	rs713586	25,011,512	c	t	0.47	6.20E-22	0.14	0.02	0.058	+	0.033*	1.09	1.01,1.17
*GPRC5B*	16	rs12444979	19,841,101	c	t	0.87	2.90E-21	0.17	0.03	0.421	+	0.941	1.00	0.89,1.13
*SH2B1*	16	rs7359397	28,793,160	t	c	0.4	1.90E-20	0.15	0.02	4.50E-04*	+	0.001*	1.14	1.05,1.23
*TFAP2B*	6	rs987237	50,911,009	g	a	0.18	2.90E-20	0.13	0.03	1.00E-05*	+	2.70E-05*	1.24	1.12,1.37
*MAP2K5*	15	rs2241423	65,873,892	g	a	0.78	1.20E-18	0.13	0.02	0.226	+	0.288	1.05	0.96,1.15
*ETV5*	3	rs9816226	187,317,193	t	a	0.82	1.70E-18	0.14	0.03	0.159	+	0.249	1.06	0.96,1.18
*FAIM2*	12	rs7138803	48,533,735	a	g	0.38	1.80E-17	0.12	0.02	4.60E-07*	+	6.40E-08*	1.24	1.15,1.34
*QPCTL*	19	rs2287019	50,894,012	c	t	0.8	1.90E-16	0.15	0.03	0.045*	+	0.133	1.07	0.98,1.18
*TNNI3K*	1	rs1514175	74,764,232	a	g	0.43	8.20E-14	0.07	0.02	0.001*	+	0.001*	1.14	1.05,1.23
*SLC39A8*	4	rs13107325	103,407,732	t	c	0.07	1.50E-13	0.19	0.04	0.035*	+	0.058	1.19	0.99,1.43
*FLJ35779*	5	rs2112347	75,050,998	t	g	0.63	2.20E-13	0.1	0.02	0.365	+	0.526	1.03	0.95,1.11
*LRRN6C*	9	rs10968576	28,404,339	g	a	0.31	2.70E-13	0.11	0.02	0.106	+	0.366	1.04	0.96,1.12
*MTCH2*	11	rs3817334	47,607,569	t	c	0.41	1.60E-12	0.06	0.02	0.037*	+	0.039*	1.08	1.00,1.17
*TMEM160*	19	rs3810291	52,260,843	a	g	0.67	1.60E-12	0.09	0.02	0.345	+	0.232	1.06	0.97,1.16
*FANCL*	2	rs887912	59,156,381	t	c	0.29	1.80E-12	0.1	0.02	0.563	+	0.642	0.98	0.90,1.07
*NRXN3*	14	rs10150332	79,006,717	c	t	0.21	2.80E-11	0.13	0.03	0.556	+	0.517	1.03	0.94,1.13
*CADM2*	3	rs13078807	85,966,840	g	a	0.2	3.90E-11	0.1	0.02	0.016*	+	0.017*	1.12	1.02,1.23
*PRKD1*	14	rs11847697	29,584,863	t	c	0.04	5.80E-11	0.17	0.05	0.011*	+	0.03*	1.24	1.02,1.51
*LRP1B*	2	rs2890652	142,676,401	c	t	0.18	1.40E-10	0.09	0.03	0.655	+	0.938	1.00	0.91,1.09
*PTBP2*	1	rs1555543	96,717,385	c	a	0.59	3.70E-10	0.06	0.02	0.756	+	0.773	1.01	0.94,1.09
*MTIF3*	13	rs4771122	26,918,180	g	a	0.24	9.50E-10	0.09	0.03	0.072	+	0.111	1.08	0.98,1.18
*ZNF608*	5	rs4836133	124,360,002	a	c	0.48	2.00E-09	0.07	0.02	0.845	−	0.394	0.97	0.89,1.05
*RPL27A*	11	rs4929949	8,561,169	c	t	0.52	2.80E-09	0.06	0.02	0.111	+	0.389	1.03	0.96,1.12
*KCTD15*	19	rs29941	39,001,372	g	a	0.67	3.00E-09	0.06	0.02	3.00E-04*	+	6.30E-05*	1.18	1.09,1.28
*NUDT3*	6	rs206936	34,410,847	g	a	0.21	3.00E-08	0.06	0.02	0.981	+	0.821	1.01	0.92,1.11
Meyre et al. 2009**														
*PTER*	10	rs10508503	16,339,957	c	t	NA	2.10E-07	NA	NA	0.029*	+	0.098	1.12	0.98,1.29
*MAF*	16	rs1424233	78,240,252	a	g	NA	3.80E-13	NA	NA	0.655	−	0.399	0.97	0.90,1.04
*NPC1*	18	rs1805081	19,394,430	g	a	NA	2.90E-07	NA	NA	0.634	−	0.640	0.98	0.91,1.06
Scherag et al. 2010**														
*SDCCAG8*	1	rs12145833	241,550,377	t	g	NA	4.80E-07	NA	NA	0.962	−	0.957	1.00	0.90,1.10
*TNKS/MSRA*	8	rs17150703	9,783,208	a	g	NA	1.90E-09	NA	NA	0.813	−	0.366	0.94	0.83,1.07

*p<0.05 in GOYA. BMI refers to the ‘BMI p’ linear regression analysis and ‘obesity p’ refers to overweight/control analysis. ‘beta’ for the BMI linear regression denotes the sign of the coefficient, with ‘+’ indicating the same direction of effect as in GIANT [3].

**overall effect estimates not available for Meyre et al. 2009 [11] or Scherag et al. 2010 [12]. Effect allele is the BMI increasing allele (according to the stage 2 adult results in Meyre et al. 2009). NA = not available.

In total, 3,741 SNPs had p<0.001 in the overweight/control analysis and 4,297 SNPs had p<0.001 in the BMI z-score linear regression analysis, resulting in 6,045 SNPs with a p<0.001 in at least one of these analyses (**Table S2** contains a full list of these SNPs), which is more than one would expect by chance (2,544 each for independent traits and SNPs). 487 SNPs were in regions that have been previously associated with BMI or obesity (**Figure 2**) [2], [1], [11]. We therefore selected the remaining 5,558 SNPs for in silico replication in the IARC sample.

### Stage 1 replication

We obtained results data for the association of 5,552 SNPs and inverse rank normal BMI from the IARC study (n = 5,846). Forty-four of these 5,552 SNPs had p<0.01 and an effect in the same direction as that observed in the GOYA sample (full results available in **Table S2**). These 44 SNPs are in 10 loci. 18 additional SNPs met the other criteria for stage two replication (if they didn't meet p<0.01 in IARC we still included SNPs with the same direction of effect in IARC and stronger levels of evidence in GOYA (p<1×10^−5^), in addition to including those with moderate evidence of association in GOYA (p<5×10^−5^) and evidence of candidacy), providing a total of 28 SNPs for replication (rationale for inclusion of each SNP in the replication are shown in Supplementary **Table S3**).

### Stage 2 replication

Of the 28 SNPs only one (rs734597) had p<0.05 and an effect in the same direction in the ALSPAC cohort and three (rs734597, rs6758546 and rs12130212) had p<0.01 and an effect in the same direction in the Danish replication cohort (**Table 4**). We carried out a meta-analysis of all cohorts and rs734597 was the only SNP to reach genome-wide significance (p = 3.1×10^−8^), with the “A” allele associated with increased BMI. This SNP is in the TFAP2B gene, which has also recently been identified by GIANT as being associated with BMI (**Figure 2**, **Table 3**) [3]. Of the other 27, although none reached genome-wide significance, 13 showed consistent directions of effect across all cohorts (where only 6 or 7 would be expected by chance) and 6 SNPs (in/near *CAMK1G*, *RGS6*, *FLJ39743*, *TLE3*, *CENTD3* and *AKAP6*) had smaller p-values in the meta-analysis than in the discovery.

**Table 4:** The 28 SNPs taken forward to replication in Danish cohort and ALSPAC.

	effect allele	other allele				GOYA IR (5373)		IARC rep stage 1 (5846)			Danish rep stage 2 (20917)		ALSPAC rep stage 2 (2418)			Meta-analysis (34554)	
SNP			Chr	Position	Gene	beta*	p	beta	se	p	beta*	p	beta	se	p	p	dir
rs734597	A	G	6	50944238	*TFAP2B*	+	6.97E-06	0.037	0.022	0.224	+	0.0008	0.074	0.037	0.0435	3.1E-08	++++
rs12130212	T	A	1	207793880	*CAMK1G*	−	4.86E-04	−0.066	0.023	0.001	−	0.0094	−0.003	0.033	0.923	2.4E-06	----
rs699363	G	A	14	71762246	*RGS6*	+	7.36E-04	0.210	0.061	2E-05	+	0.4289	0.065	0.09	0.4727	4.7E-06	++++
rs970843	C	G	15	96693552	*FLJ39743*	−	9.07E-04	−0.070	0.028	0.008	−	0.1423	−0.064	0.038	0.0883	5.0E-06	----
rs1912967	T	A	15	68370390	*TLE3*	+	6.82E-04	0.067	0.022	4E-04	+	0.5138	0.029	0.031	0.342	1.9E-05	++++
rs32930	A	G	5	141093111	*CENTD3*	−	1.81E-04	−0.051	0.020	0.009	−	0.6574	−0.043	0.029	0.1394	3.4E-05	----
rs6758546	A	G	2	17902293	*AKAP6*	−	8.75E-05	−0.020	0.030	0.875	−	0.0018	−0.043	0.044	0.3299	4.3E-05	----
rs791903	G	C	6	33810623	*IHPK3*	−	5.01E-06	−0.023	0.019	0545	−	0.2615	−0.045	0.028	0.1025	7.4E-05	----
rs9566498	C	T	13	39671652	*FOX01A*	−	4.45E-05	−0.075	0.030	0.012	−	0.4019	0.008	0.042	0.8522	3.0E-04	---+
rs12938476	C	T	17	41168668	*CRHR1*	+	4.31E-05	0.030	0.019	0.282	+	0.0888	0.004	0.029	0.894	4.6E-04	++++
rs7720663	G	A	5	164598808	−	−	6.34E-06	−0.030	0.019	0.274	−	0.4282	0	0.028	0.9987	0.001	---0
rs582683	A	T	18	7707565	*PTPRM*	+	4.44E-05	0.015	0.021	0.850	+	0.2168	0.037	0.032	0.2492	0.001	++++
rs6571507	A	G	14	31842078	−	+	7.71E-05	0.013	0.019	0.854	+	0.1475	0.032	0.029	0.2594	0.001	++++
rs7542125	C	T	1	201137861	*KLHL12*	+	2.72E-04	0.060	0.022	0.003	+	0.6138	−0.01	0.032	0.7565	0.001	+++-
rs10987417	T	G	9	128502322	*LMX1B*	+	5.07E-05	0.018	0.020	0.753	+	0.4153	0.03	0.031	0.3312	0.002	++++
rs1420533	A	G	16	51121127	*TNRC9*	+	3.40E-04	0.049	0.018	0.004	−	0.6192	0.004	0.027	0.8868	0.002	++-+
rs17504169	A	G	3	191585563	*CLDN16*	+	2.80E-06	0.032	0.029	0.603	+	0.8705	0.008	0.039	0.8415	0.005	++++
rs4765158	C	A	12	123658249	*NCOR2*	−	9.62E-04	−0.077	0.027	0.002	−	0.7028	0.045	0.04	0.2537	0.005	---+
rs9532670	T	C	13	40399827	*ELF1*	+	2.50E-03	0.051	0.020	0.007	+	0.7617	−0.002	0.03	0.4576	0.008	+++-
rs8067056	C	T	17	41439785	*MAPT*	+	8.31E-06	0.017	0.020	0.769	+	0.2889	−0.021	0.029	0.4789	0.011	+++-
rs4964926	G	C	12	130882177	*MMP17*	+	6.63E-06	0.005	0.034	0.993	+	0.8134	0.007	0.052	0.8885	0.014	++++
rs12155554	G	A	8	118391632	*SLC30A8*	−	1.04E-04	−0.030	0.044	0.860	+	0.6973	−0.084	0.069	0.2187	0.014	--+-
rs17241549	G	C	6	100971001	*SIM1*	−	1.31E-04	−0.004	0.084	0.999	−	0.0919	0.081	0.107	0.4472	0.018	---+
rs876338	C	T	2	137005617	*CXCR4*	−	2.05E-05	−0.021	0.019	0.613	+	0.4564	−0.014	0.031	0.6514	0.025	--+-
rs10047878	T	A	14	21857530	*DAD1*	+	4.75E-05	0.012	0.019	0.883	−	0.7729	−0.007	0.028	0.792	0.067	++--
rs365760	T	A	16	53949133	*MMP2*	+	7.39E-04	0.007	0.020	0.971	−	0.7951	0.014	0.032	0.6722	0.074	++-+
rs10102742	T	C	8	132373690	*ADCY8*	+	2.41E-03	0.054	0.019	0.001	−	0.0021	0.007	0.028	0.8115	0.077	++-+
rs17104665	G	A	5	145822662	*TCERG1*	−	1.15E-05	−0.015	0.037	0.952	+	0.629	0.041	0.055	0.4559	0.108	--++

Betas (and SEs) are in z-scores.

*betas for GOYA and the Danish replication are biased by the sample strategy, so only the sign of the coefficients are shown.

## Discussion

In this study we found strong evidence of association between BMI and many known BMI loci, including *FTO*, *MC4R*, *TMEM18*, *GNPDA2*, *FAIM2*, *SEC16B*, *TFAP2B*, SH2B1 and *KCTD15* using an efficient and powerful selective genotyping approach. Although we did not identify any new BMI loci that reached genome-wide confidence levels in the combined meta-analysis, a number of variants showed consistent directions of effect in the replication analyses (more than expected by chance) and it would be worth following up these loci in other cohorts to investigate a possible small effect on BMI.

There has been some concern that study designs which involve genotyping individuals with extreme phenotypic scores from one or both ends of the distribution may not translate into increased power to detect common variants in genome-wide association analysis if the individuals at the extremes are not enriched for common alleles, but instead harbor rare alleles of large effect and/or are influenced by extreme environmental factors. We have shown that it was possible to replicate many of the known common variants underlying variance in BMI using a sample selected for extreme obesity compared to a sample of randomly selected controls from the same population, demonstrating that the extremes may be enriched for the common variants. Additionally, many of the variants identified showed strong evidence of association in our sample using much smaller numbers than previous genome-wide association studies, most of which involved tens of thousands of individuals combined via meta-analysis. For example, our discovery strategy successfully identified the *FAIM2* region at genome-wide significant levels using far fewer numbers (n = 5,373) than the initial study, which identified this region as associated with BMI (n = 34,416) [1]. Many of the other known BMI-associated regions were also near the top of our p-value distribution, and had this been an initial discovery study, we would have identified several of the known hits using the present efficient study design. Consistent with Cotsapas et al. [10] (who compared severely obese individuals with controls) we found little/no evidence for association between BMI and *ETV5*, *NPC1* or *MAF* variants using our extreme genotyping design. However, for the 10 other known BMI SNPs studied both in the Cotsapas et al paper and here, we found stronger evidence for all, including four (*MC4R*, *MTCH2*, *SEC16B* and *BDNF*), for which we found evidence for association, whilst Cotsapas et al. report little or none.

Seventeen of the known BMI loci showed little evidence for association using our extreme study design. However, most of these were only recently found to be associated in the exceptionally large GIANT study [3] and have very small effect sizes, which even with our efficient design, we had little power to detect. Of interest are the variants in/near five genes (*PTER*, *NPC1*, *MAF*, *SDCCAG8* and *TNKS/MSRA*) that had previously been associated with obesity also using samples of individuals who were extremely overweight [11], [12]. GIANT found limited or no evidence of association at these variants with BMI [3]. We found evidence for an association with the *PTER* variant in our extremes linear regression analysis, but no evidence of association with the other four variants. It could be that the *PTER* variant specifically conveys risk of extreme obesity, and is not associated with the general distribution of BMI e.g. tagging rare SNPs of large effect. The reason for lack of association with the other two genes in our study could be that they are associated only with extreme obesity in a particular population (i.e. children and adolescents for *TNKS/MSRA*), perhaps dependent on particular gene-environment interactions or that we did not have the power to detect them in our discovery sample.

One SNP which we took forward to replication reached genome-wide significance in the combined meta-analysis (rs734597 in *TFAP2B*). Variants within this gene have been previously associated with waist circumference and waist hip ratio [18] so it was perhaps not surprising that this locus was associated with BMI/obesity too, and since our discovery analysis a variant in this gene (rs987237) in LD with rs734597 (r^2^ = 1) was shown to be associated with BMI [3]. We did not identify any novel variants associated with BMI at genome-wide confidence levels. This might be because although we used a powerful study design in our discovery set, drawn from a population of ∼212,000 individuals, we attempted replication in population samples, which have much less power. As previous meta-analyses combining population sample GWA studies have been much larger than our replication sample, it would perhaps be unlikely to obtain genome-wide significance in a population sample replication set of this size.

Finally we noticed that on the majority of occasions, analysing the data using BMI z-score linear regression yielded stronger evidence against the null for known obesity loci than treating BMI as a dichotomous variable and analyzing the data via logistic regression. Such a result is consistent with the literature, which suggests that the inclusion of quantitative information usually yields a more powerful test of association [13], [15], [16].

In summary, we have confirmed that employing a selective genotyping design whereby extremely overweight individuals are compared to individuals from a random population sample is a powerful way to identify common variants of relatively small effect that influence BMI. Many of the variants that were previously found to influence BMI in large population based cohorts also showed strong or nominal levels of association in our study. Our results suggest that population-based study designs with enriched sampling of individuals with the extreme phenotype, combined with using the phenotype as a quantitative trait may be an efficient and powerful method for identifying common variants that influence BMI and possibly other quantitative traits and a valid alternative to genotyping all individuals in large population-based studies, which may require tens of thousands of subjects to achieve similar levels of power.

## Supporting Information

Figure S1 **MDS Plot for the GOYA participants.** Seeded with the HapMap CEU (Utah residents with northern and western European ancestry from the CEPH collection), YRI (Yoruba from Ibadan, Nigeria) and JPT and CHB (Japanese from Tokyo, Japan and Chinese from Beijing, China) panels (release 22). Blue curves indicate the thresholds outside of which GOYA individuals were excluded from the GWAS. There was little evidence for additional population structure as indicated by the GWAS lambdas λ = 1.05 and 1.06.
(TIF)

Figure S2 **QQ plot for the GOYA overweight/control genome-wide analysis.** Lambda = 1.051.
(TIF)

Figure S3 **Manhattan plot for the GOYA overweight/control genome-wide analysis.**
(TIF)

Table S1 Characteristics of the six Danish stage 2 replication cohorts.
(PDF)

Table S2 GOYA and IARC results for the 6,045 SNPs with p<0.001 in GOYA. Known SNPs were excluded from the IARC analysis.
(PDF)

Table S3 Reasons for including each SNP in the stage 2 replication.
(PDF)

Text S1 Stage 1 Replication—IARC participants, genotyping and analysis; GeneSniffer methods; Stage 2 Replication—Danish Cohorts participants, genotyping and analysis; and Stage 2 Replication—ALSPAC participants, genotyping and analysis.
(DOC)
