# Supplementary figures and images for: Genome-Wide Population-Based Association Study of Extremely Overweight Young Adults – The GOYA Study
Source: PLoS One. 2011 Sep 15;6(9):e24303. doi: 10.1371/journal.pone.0024303 (PMC3174168; doi:10.1371/journal.pone.0024303)

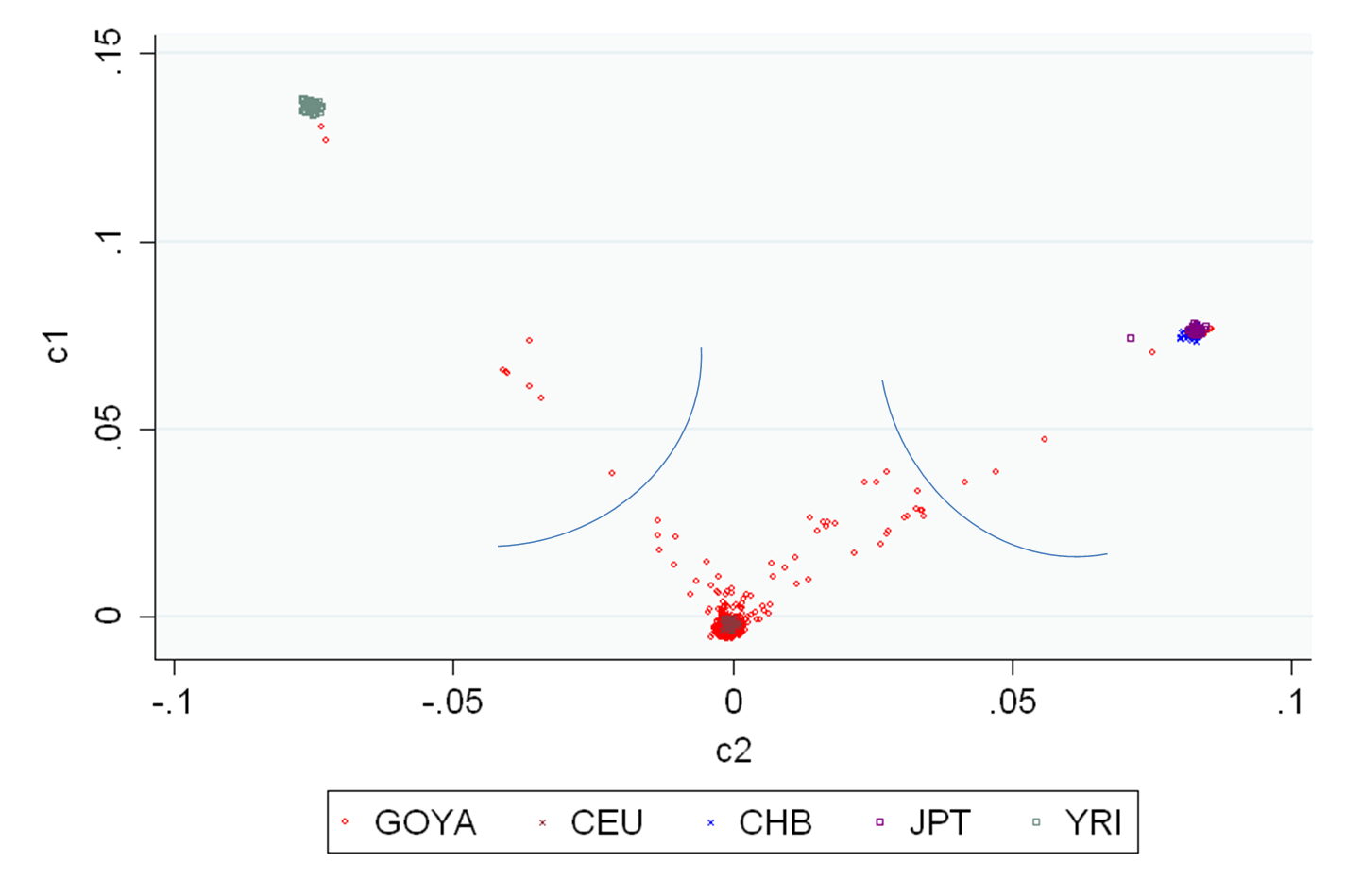

Supplement: Figure S1 — MDS Plot for the GOYA participants. Seeded with the HapMap CEU (Utah residents with northern and western European ancestry from the CEPH collection), YRI (Yoruba from Ibadan, Nigeria) and JPT and CHB (Japanese from Tokyo, Japan and Chinese from Beijing, China) panels (release 22). Blue curves indicate the thresholds outside of which GOYA individuals were excluded from the GWAS. There was little evidence for additional population structure as indicated by the GWAS lambdas λ = 1.05 and 1.06. (TIF) [file pone.0024303.s001.tif]

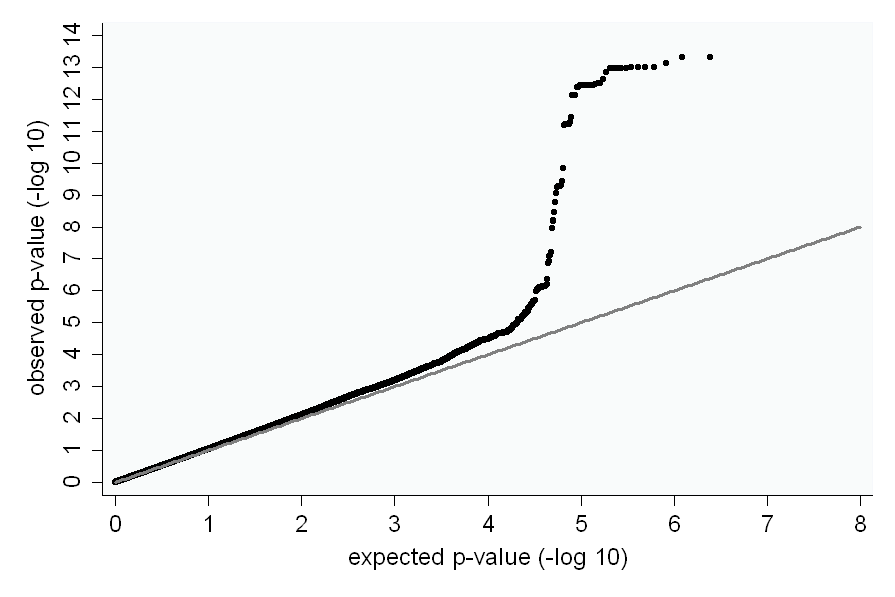

Supplement: Figure S2 — QQ plot for the GOYA overweight/control genome-wide analysis. Lambda = 1.051. (TIF) [file pone.0024303.s002.tif]

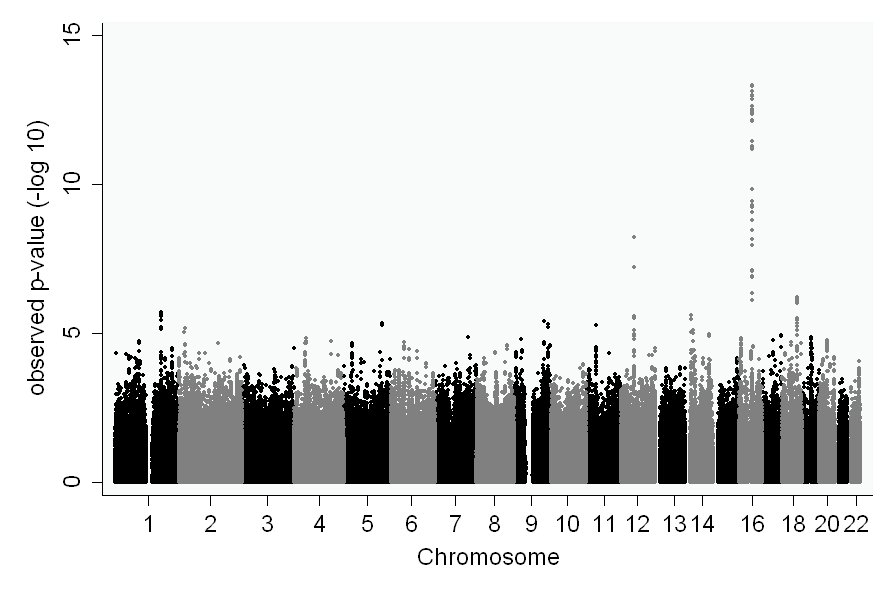

Supplement: Figure S3 — Manhattan plot for the GOYA overweight/control genome-wide analysis. (TIF) [file pone.0024303.s003.tif]
