# Supplementary material for: Genome-Wide Population-Based Association Study of Extremely Overweight Young Adults – The GOYA Study
Source: PLoS One. 2011 Sep 15;6(9):e24303. doi: 10.1371/journal.pone.0024303 (PMC3174168; doi:10.1371/journal.pone.0024303)
Supplement: Table S1 — Characteristics of the six Danish stage 2 replication cohorts. (PDF) [file pone.0024303.s004.pdf]

	Inter99	Helbred 06	SDC1	SDC2	Ung92	ADDITION
**N (male/female)**	6360 (3117/3243)	3229 (1456/1773)	681 (340/341)	2038 (1239/799)	377 (186/191)	8602 (4695/3907)
**Age (SD)**	46.2 (7.9)	49.2 (13.2)	57.6 (9.4)	61.9 (11.4)	25.2 (3.5)	60.0 (6.8)
**BMI (SD)**	26.3 (4.6)	25.9 (4.7)	26.3 (4.2)	30.0 (5.6)	23.6 (3.7)	28.6 (4.9)

Values are mean (standard deviation), apart from N which is the total number of subject in each study, and numbers split by gender
