# Supplementary material for: Genome-Wide Population-Based Association Study of Extremely Overweight Young Adults – The GOYA Study
Source: PLoS One. 2011 Sep 15;6(9):e24303. doi: 10.1371/journal.pone.0024303 (PMC3174168; doi:10.1371/journal.pone.0024303)
Supplement: Table S2 — GOYA and IARC results for the 6,045 SNPs with p<0.001 in GOYA. Known SNPs were excluded from the IARC analysis. (PDF) [file pone.0024303.s005.pdf]

					GOYA QC		GOYA Overweight/control			GOYA BMI continuous	known	IARC QC		IARC results		
MARKER	chr	position	A1	A2	FREQ1	Rsqr	Beta	SE	p	p	gene name	freq1	Rsqr	in_beta	in_SE	in_p
rs1157688	1	4536627	C	T	0.786	1.00	0.194	0.048	0.0000479	0.0004078		0.771	0.99	-0.04712	0.02206	0.032606
rs6540992	1	11721815	A	T	0.656	0.88	0.104	0.044	0.01682	0.0008958		0.641	0.92	0.027364	0.02027	0.1767074
rs4073574	1	11725021	G	T	0.336	1.00	-0.097	0.041	0.01859	0.0008585		0.350	1.00	-0.02609	0.01958	0.1824246
rs12752700	1	11730013	C	T	0.336	1.00	-0.097	0.041	0.01926	0.0008883		0.350	0.99	-0.02598	0.01966	0.1859836
rs2489258	1	12623402	C	T	0.030	0.39	0.627	0.186	0.000745	0.0094447		0.025	0.25	0.104025	0.13585	0.4434387
rs10927872	1	13757790	C	T	0.428	1.00	-0.137	0.039	0.0005066	0.00033		0.508	1.00	0.008183	0.01812	0.6512945
rs16850285	1	14659997	C	T	0.953	0.93	-0.324	0.094	0.0005865	0.0010365		0.936	0.38	-0.03864	0.06088	0.5252646
rs2977272	1	17409539	C	T	0.674	0.99	-0.138	0.041	0.0007657	0.0362394		0.667	0.99	-0.01412	0.01943	0.4671409
rs17351871	1	18710909	C	G	0.036	0.93	0.363	0.109	0.000875	0.0036091		0.057	0.48	-0.02591	0.05634	0.6453883
rs818968	1	20151992	C	T	0.413	1.00	0.114	0.039	0.003846	0.0007436		0.392	1.00	0.002639	0.01843	0.8860075
rs1796926	1	20152318	A	G	0.587	1.00	-0.114	0.039	0.003862	0.0007475		0.608	0.99	-0.00263	0.01843	0.8863637
rs7551768	1	20152446	C	G	0.587	1.00	-0.114	0.039	0.0039	0.0007566		0.608	0.99	-0.00256	0.01846	0.8897983
rs10753476	1	20153198	A	G	0.413	1.00	0.114	0.04	0.003913	0.0007597		0.392	0.99	0.002554	0.01847	0.8899079
rs1796925	1	20154102	A	T	0.397	0.92	0.12	0.041	0.00386	0.0005034		0.376	0.92	0.00056	0.01942	0.9769978
rs1796924	1	20154556	A	G	0.413	1.00	0.114	0.039	0.00401	0.0007837		0.392	0.99	0.002463	0.01851	0.8940535
rs4654973	1	21800407	C	G	0.463	0.93	-0.135	0.04	0.0007216	0.0084218		0.439	0.85	0.000162	0.01984	0.9934655
rs2139539	1	31890366	C	T	0.420	1.00	-0.119	0.04	0.002667	0.0006763		0.454	1.00	-0.01817	0.01836	0.3221119
rs3817398	1	31892018	C	T	0.579	1.00	0.118	0.04	0.003013	0.0007544		0.546	1.00	0.018223	0.01837	0.3207634
rs16835229	1	33218424	C	T	0.884	0.94	-0.206	0.063	0.001078	0.0007536		0.916	0.87	-0.03629	0.03423	0.2885616
rs7547654	1	41603948	C	G	0.472	0.70	-0.189	0.047	0.0000515	0.0000157		0.479	0.67	-0.04543	0.02263	0.0445945
rs11210886	1	43844133	A	G	0.076	0.99	-0.252	0.074	0.000648	0.0088526		0.050	0.99	0.101795	0.04072	0.0123738
rs11210888	1	43849217	C	T	0.924	0.99	0.255	0.074	0.0005547	0.0078588		0.952	0.98	-0.09261	0.04138	0.0251262
rs11804031	1	43854499	A	G	0.924	1.00	0.255	0.074	0.00054	0.0076921		0.952	0.98	-0.09224	0.04139	0.0257354
rs16831024	1	43857207	G	T	0.924	1.00	0.256	0.074	0.0005364	0.0076482		0.952	0.98	-0.09198	0.04139	0.0261729
rs11210893	1	43874354	G	T	0.076	1.00	-0.259	0.074	0.0004606	0.0069315		0.047	1.00	0.081558	0.04153	0.0494088
rs11210894	1	43875174	C	T	0.924	1.00	0.259	0.074	0.00046	0.0069229		0.953	1.00	-0.08147	0.04153	0.049645
rs11210896	1	43875862	A	G	0.076	1.00	-0.259	0.074	0.0004592	0.0069115		0.047	1.00	0.081438	0.04153	0.0497368
rs4336891	1	43899027	A	G	0.075	0.95	-0.264	0.076	0.000547	0.0088374		0.048	0.93	0.080988	0.04287	0.058671
rs10789441	1	43903151	A	G	0.925	0.92	0.266	0.077	0.0005977	0.009808		0.952	0.93	-0.08098	0.04289	0.0588178
rs7543627	1	47439370	A	C	0.041	0.94	-0.29	0.103	0.004665	0.0009106		0.031	0.98	-0.05622	0.05247	0.2835659
rs12123358	1	47496411	C	G	0.702	1.00	0.132	0.042	0.001699	0.000941		0.718	1.00	0.012428	0.02033	0.5405843
rs13376679	1	47498674	C	T	0.298	1.00	-0.132	0.042	0.001696	0.0009374		0.283	1.00	-0.01217	0.02033	0.5489002
rs13374382	1	47500534	C	G	0.702	1.00	0.132	0.042	0.001696	0.0009374		0.718	1.00	0.012173	0.02033	0.5489136
rs11211500	1	47504965	G	T	0.702	1.00	0.132	0.042	0.001696	0.0009375		0.718	1.00	0.012173	0.02033	0.5489136
rs2405787	1	47511544	C	T	0.298	1.00	-0.132	0.042	0.001735	0.0009714		0.283	1.00	-0.01219	0.02033	0.5483097
rs12132030	1	48079764	A	G	0.015	0.33	-0.888	0.295	0.002648	0.0000765		0.015	0.23	0.063299	0.15155	0.6759261
rs1740109	1	48444700	C	G	0.492	0.99	-0.13	0.039	0.0008927	0.0011019		0.491	0.99	0.011701	0.01825	0.5211499
rs537303	1	48446523	C	T	0.162	1.00	0.179	0.053	0.0006454	0.0009801		0.211	0.98	-0.05049	0.0225	0.0247435
rs490631	1	48455992	A	T	0.838	1.00	-0.18	0.053	0.0005955	0.0009054		0.789	0.98	0.050368	0.02253	0.0252748
rs212989	1	48467668	A	G	0.161	1.00	0.173	0.053	0.0009844	0.0011889		0.206	1.00	-0.04474	0.02258	0.0474001
rs540944	1	48478466	C	T	0.166	0.98	0.18	0.052	0.0005778	0.000683		0.235	0.94	-0.04893	0.02222	0.0275337
rs850763	1	48480815	G	T	0.839	1.00	-0.175	0.053	0.0008963	0.001129		0.794	0.99	0.044944	0.0226	0.0465868
rs3766467	1	54145773	G	T	0.970	0.90	0.484	0.123	0.0000776	0.0001466		0.968	0.87	0.114671	0.05957	0.0540488
rs12073655	1	54166080	A	C	0.033	1.00	-0.447	0.112	0.0000611	0.0001425		0.033	0.93	-0.10754	0.05647	0.0567189
rs11206252	1	54169828	A	G	0.033	1.00	-0.447	0.112	0.0000614	0.0001421		0.033	0.93	-0.10743	0.05646	0.0569278
rs12092723	1	54195489	C	G	0.967	1.00	0.445	0.112	0.00007	0.0001383		0.967	0.93	0.104697	0.05615	0.0620573
rs10888828	1	54214344	A	G	0.963	1.00	0.392	0.106	0.00022	0.00018		0.959	0.95	0.064123	0.05014	0.2005987
rs11206266	1	54216455	C	T	0.963	1.00	0.391	0.106	0.0002213	0.0001809		0.959	0.95	0.064121	0.05014	0.2005863
rs7515322	1	54223489	A	G	0.963	0.99	0.397	0.106	0.0001912	0.0001511		0.959	0.95	0.064379	0.05012	0.1986231
rs7525809	1	54223668	A	G	0.037	0.99	-0.397	0.106	0.0001909	0.0001499		0.041	0.95	-0.06462	0.0502	0.1976195
rs17109656	1	54248735	A	G	0.036	0.97	-0.411	0.109	0.0001681	0.0001298		0.035	0.86	-0.07376	0.05657	0.1919509
rs11805505	1	54252998	A	T	0.034	0.98	-0.378	0.111	0.0006488	0.0003391		0.033	0.82	-0.08081	0.05992	0.1770778
rs6688562	1	57039195	C	G	0.743	0.94	0.139	0.045	0.002272	0.0004312		0.725	0.90	-0.01069	0.02124	0.6142704
rs1361727	1	57039264	C	G	0.257	0.95	-0.138	0.045	0.002272	0.000432		0.275	0.92	0.0101	0.02105	0.6310726
rs7513447	1	57039676	G	T	0.260	0.94	-0.14	0.045	0.002067	0.0004089		0.279	0.92	0.011467	0.02085	0.5820844
rs1361730	1	57040534	C	T	0.749	1.00	0.136	0.044	0.002274	0.000457		0.731	1.00	-0.00892	0.02032	0.6603464
rs7554784	1	57043547	C	T	0.743	0.99	0.14	0.044	0.001601	0.000232		0.708	0.95	-0.00476	0.02038	0.8151197
rs7531769	1	57043625	A	G	0.230	0.80	-0.153	0.051	0.002749	0.0003493		0.258	0.74	0.005921	0.02404	0.8052672
rs1581762	1	58023648	A	G	0.422	1.00	-0.123	0.039	0.001633	0.0002299		0.414	0.94	0.006123	0.01917	0.7492753
rs10889072	1	58024576	C	G	0.579	1.00	0.124	0.039	0.001577	0.000221		0.586	0.94	-0.00614	0.01917	0.7483119
rs2057225	1	58076581	A	G	0.881	1.00	0.194	0.06	0.001166	0.0000442		0.888	0.99	-0.01487	0.02845	0.6007422
rs1359178	1	58078020	C	G	0.835	0.99	0.137	0.052	0.008269	0.0009077		0.843	0.98	0.008531	0.02512	0.7339064
rs764742	1	58098782	A	G	0.109	0.95	0.256	0.064	0.0000695	0.0006355		0.117	0.35	-0.03593	0.04756	0.4495929
rs1933213	1	59956261	G	T	0.181	0.99	0.176	0.05	0.0004999	0.0041137		0.139	0.51	0.02774	0.0379	0.4638186
rs9436182	1	59958268	A	C	0.819	0.96	-0.177	0.051	0.0005372	0.0043837		0.860	0.49	-0.02753	0.03853	0.4746263
rs7551363	1	60661587	G	T	0.094	0.99	-0.228	0.067	0.0006936	0.0006064		0.099	0.76	0.020571	0.03389	0.5435728
rs12028543	1	61141307	A	G	0.067	0.97	0.265	0.08	0.0008801	0.0018792		0.089	0.97	0.027838	0.03314	0.4004505
rs7554824	1	63414577	A	C	0.485	1.00	0.156	0.039	0.0000644	0.0001096		0.487	0.98	0.008774	0.01849	0.6348267
rs7515372	1	63415839	A	C	0.485	1.00	0.156	0.039	0.0000639	0.0001084		0.487	0.98	0.008804	0.01849	0.6336414
rs4253945	1	63428502	C	T	0.502	1.00	-0.133	0.039	0.0006105	0.0015962		0.494	1.00	-0.01346	0.01832	0.4620579
rs12724092	1	63430237	C	G	0.502	1.00	-0.133	0.039	0.0006053	0.0016052		0.494	1.00	-0.01348	0.01832	0.4615104
rs12070428	1	63474307	C	G	0.384	1.00	-0.116	0.04	0.003803	0.0003561		0.381	0.99	-0.00196	0.01897	0.9178094
rs1854335	1	63475918	A	C	0.616	1.00	0.116	0.04	0.003633	0.0003244		0.618	0.99	0.001754	0.01897	0.9262528
rs621058	1	63489346	C	T	0.615	1.00	0.118	0.04	0.003224	0.0002486		0.616	1.00	0.000775	0.01888	0.9672321
rs538071	1	63492812	C	T	0.615	1.00	0.118	0.04	0.003218	0.000248		0.616	1.00	0.000846	0.01889	0.9642247
rs6588052	1	63873066	A	T	0.695	0.92	0.147	0.044	0.0008742	0.0247785		0.603	0.79	-0.01216	0.02083	0.558914
rs2269248	1	63874702	A	G	0.711	1.00	0.148	0.044	0.0006913	0.0154068		0.619	0.87	-0.01058	0.02032	0.6020968
rs344938	1	67899025	A	G	0.858	1.00	0.187	0.056	0.0007713	0.0024329		0.878	0.97	-0.02653	0.02792	0.3416561
rs344931	1	67908426	C	T	0.858	1.00	0.186	0.056	0.0008674	0.0024758		0.879	0.98	-0.0267	0.02782	0.3368396
rs598602	1	67934425	A	G	0.145	0.98	-0.184	0.056	0.0009393	0.0019627		0.128	0.96	0.026708	0.02755	0.331915
rs2820500	1	68431549	C	T	0.328	1.00	-0.144	0.041	0.0004658	0.0036794		0.277	0.99	0.049459	0.02037	0.0151421
rs1036066	1	68432793	A	C	0.672	1.00	0.144	0.041	0.0004533	0.0036332		0.724	0.98	-0.0496	0.02046	0.0153053

					GOYA QC		GOYA Overweight/control			GOYA BMI continuous	known	IARC QC		IARC results		
MARKER	chr	position	A1	A2	FREQ1	Rsqr	Beta	SE	p	p	gene name	freq1	Rsqr	in_beta	in_SE	in_p
rs2116047	1	68434339	A	G	0.672	1.00	0.145	0.041	0.0004209	0.0035088		0.723	0.91	-0.051	0.02126	0.0164019
rs2039154	1	68435401	A	G	0.328	1.00	-0.145	0.041	0.0004357	0.0035588		0.277	0.91	0.051082	0.02132	0.0165246
rs919540	1	68435731	C	T	0.672	1.00	0.145	0.041	0.000448	0.0035973		0.723	0.90	-0.05112	0.02135	0.0165692
rs12125823	1	71705132	A	G	0.512	0.99	0.108	0.039	0.005447	0.0009119	NEGR1					
rs12082706	1	71926379	C	T	0.034	0.69	0.381	0.129	0.003188	0.0007695	NEGR1					
rs6690871	1	74749865	A	G	0.561	0.95	-0.132	0.04	0.0009693	0.0006374		0.603	0.95	-0.01707	0.01915	0.3724137
rs1040070	1	74750458	C	G	0.561	0.96	-0.131	0.04	0.0009937	0.0006548		0.602	0.96	-0.01675	0.01905	0.3790034
rs10493544	1	74756423	C	T	0.562	1.00	-0.127	0.039	0.001123	0.0007369		0.601	0.99	-0.01573	0.01874	0.4008123
rs1514177	1	74763990	C	G	0.434	1.00	0.127	0.039	0.001083	0.0006927		0.393	0.97	0.01926	0.01901	0.310683
rs1514176	1	74764184	A	G	0.566	1.00	-0.127	0.039	0.001088	0.0006981		0.607	0.97	-0.01927	0.01902	0.3104713
rs1514175	1	74764232	A	G	0.434	1.00	0.127	0.039	0.001092	0.0007028		0.393	0.97	0.019276	0.01905	0.3110929
rs6604867	1	74764866	C	T	0.558	1.00	-0.126	0.039	0.001223	0.0009532		0.601	0.96	-0.01583	0.01901	0.4046647
rs6604866	1	74765134	C	G	0.558	1.00	-0.126	0.039	0.001224	0.0009537		0.601	0.96	-0.01589	0.01908	0.4045298
rs1514174	1	74765651	C	T	0.443	1.00	0.126	0.039	0.001226	0.0009545		0.400	0.95	0.015908	0.01909	0.4042641
rs7526762	1	74765906	A	G	0.443	1.00	0.126	0.039	0.001227	0.0009548		0.400	0.95	0.0159	0.0191	0.4047689
rs7551507	1	74767813	C	T	0.445	1.00	0.127	0.039	0.001122	0.0009482		0.405	0.91	0.014877	0.01945	0.4438764
rs12042908	1	74770350	A	G	0.445	1.00	0.127	0.039	0.001125	0.0009508		0.405	0.91	0.014894	0.01947	0.4439343
rs2344508	1	74772301	A	G	0.555	1.00	-0.127	0.039	0.001138	0.0009597		0.595	0.91	-0.01491	0.01953	0.4446248
rs12566985	1	74774781	A	G	0.555	1.00	-0.126	0.039	0.001162	0.0009882		0.595	0.90	-0.01496	0.01963	0.4456291
rs7520945	1	74777531	C	T	0.555	1.00	-0.126	0.039	0.001152	0.00098		0.595	0.87	-0.01509	0.01995	0.4490871
rs7553348	1	74777655	A	G	0.555	1.00	-0.127	0.039	0.001133	0.0009569		0.594	0.86	-0.01512	0.02002	0.4497
rs7553158	1	74777826	A	G	0.555	1.00	-0.127	0.039	0.001107	0.0009237		0.594	0.85	-0.01515	0.02011	0.4508293
rs3895907	1	74778615	A	G	0.445	1.00	0.127	0.039	0.00109	0.0009036		0.406	0.84	0.01521	0.02026	0.4524928
rs7514705	1	74779308	C	T	0.555	1.00	-0.127	0.039	0.001064	0.0008753		0.594	0.83	-0.01526	0.02039	0.4538556
rs10789396	1	74786950	C	T	0.561	0.84	-0.142	0.042	0.000782	0.0003961		0.590	0.65	-0.0165	0.02306	0.4740866
rs1048575	1	77330645	C	G	0.243	0.96	-0.153	0.046	0.0009888	0.0000556		0.204	0.92	-0.01851	0.02331	0.4267624
rs2069224	1	77332804	C	T	0.747	0.99	0.156	0.045	0.000462	0.0000807		0.774	0.99	0.00393	0.02151	0.8548984
rs11162271	1	77336576	A	G	0.254	1.00	-0.155	0.045	0.0005103	0.000089		0.228	1.00	-0.0047	0.02137	0.8259641
rs11806823	1	77338003	A	G	0.746	1.00	0.155	0.045	0.0005181	0.0000894		0.773	1.00	0.004752	0.02137	0.8238809
rs4949766	1	77342077	C	T	0.746	1.00	0.154	0.044	0.0005315	0.0000897		0.773	1.00	0.004904	0.02137	0.8183188
rs7538430	1	77363549	A	G	0.746	1.00	0.154	0.045	0.0005289	0.0000888		0.773	1.00	0.004831	0.02139	0.8212152
rs1146675	1	77402641	A	G	0.746	1.00	0.155	0.045	0.0005224	0.0000866		0.774	0.99	0.005095	0.02147	0.8122912
rs1167217	1	77558593	A	G	0.241	1.00	0.137	0.045	0.002459	0.0005907		0.183	1.00	0.01994	0.02368	0.3993207
rs2602951	1	77563613	G	T	0.223	0.92	0.147	0.048	0.002374	0.0007776		0.166	0.91	0.027641	0.02581	0.2837942
rs2815324	1	77564277	C	T	0.243	1.00	0.14	0.045	0.001998	0.0005277		0.182	1.00	0.020081	0.02367	0.3959111
rs2815325	1	77567878	G	T	0.757	1.00	-0.14	0.045	0.001985	0.0005262		0.818	1.00	-0.02013	0.02367	0.3947149
rs2815326	1	77571533	C	T	0.757	1.00	-0.14	0.045	0.00197	0.0005239		0.818	1.00	-0.0202	0.02367	0.3930803
rs1463324	1	77579542	A	G	0.757	1.00	-0.141	0.045	0.001882	0.0005121		0.818	1.00	-0.02007	0.02368	0.3962673
rs6662853	1	77581888	C	T	0.757	1.00	-0.141	0.045	0.001814	0.0005036		0.818	1.00	-0.01997	0.02368	0.3987538
rs1874819	1	77685557	C	T	0.254	1.00	0.143	0.045	0.001379	0.0008593		0.241	0.97	0.013162	0.02162	0.5423781
rs1498405	1	77700536	A	G	0.740	0.99	-0.149	0.045	0.0008725	0.0005244		0.744	0.98	-0.00163	0.0211	0.9384273
rs6704141	1	77704683	C	T	0.730	1.00	-0.143	0.044	0.001138	0.0002707		0.735	0.99	0.000309	0.02075	0.9881142
rs12047928	1	77705338	A	G	0.730	0.97	-0.137	0.044	0.002064	0.0008037		0.742	0.92	-0.00744	0.02176	0.7322035
rs6695572	1	77718223	A	G	0.183	0.98	0.185	0.051	0.0002755	0.0000899		0.165	0.96	0.012148	0.02515	0.6287131
rs6698295	1	77718553	A	G	0.183	0.98	0.185	0.051	0.0002758	0.0000904		0.165	0.96	0.012077	0.02514	0.6306059
rs2088518	1	77723918	A	T	0.817	0.98	-0.185	0.051	0.00028	0.0000949		0.835	0.96	-0.01201	0.02513	0.6323645
rs9324162	1	77727392	A	G	0.815	1.00	-0.191	0.05	0.0001459	0.0000848		0.836	1.00	-0.00947	0.02476	0.7019154
rs17380544	1	77731300	A	G	0.297	1.00	0.15	0.043	0.0004786	0.000256		0.283	0.98	-0.00933	0.02064	0.6508931
rs2803155	1	77731425	A	C	0.332	1.00	0.142	0.041	0.0006272	0.000245		0.327	0.99	0.012561	0.01975	0.52442
rs11162355	1	77733662	G	T	0.332	1.00	0.141	0.041	0.0006695	0.0002595		0.329	1.00	0.011542	0.01958	0.5551322
rs7514041	1	77736857	A	G	0.668	1.00	-0.141	0.041	0.000644	0.0002487		0.671	1.00	-0.01138	0.01959	0.560822
rs11806197	1	77737443	A	G	0.812	1.00	-0.179	0.05	0.0003326	0.0001503		0.833	0.99	-0.00896	0.02455	0.7148375
rs2803152	1	77739344	C	T	0.332	1.00	0.141	0.041	0.0006442	0.0002512		0.329	1.00	0.011454	0.01959	0.5584696
rs12049202	1	77740111	C	T	0.805	1.00	-0.158	0.049	0.001289	0.0007992		0.827	0.98	-0.01256	0.02435	0.605778
rs12040471	1	77740989	A	T	0.188	1.00	0.179	0.05	0.0003421	0.0001558		0.167	0.99	0.00895	0.02456	0.7152628
rs12042881	1	77741031	A	G	0.188	1.00	0.179	0.05	0.0003447	0.0001573		0.167	0.99	0.008954	0.02456	0.7151707
rs17380796	1	77748705	A	G	0.787	1.00	-0.19	0.048	0.0000704	0.0000202		0.795	1.00	-0.03196	0.02263	0.1575048
rs17384946	1	77750793	A	G	0.209	0.89	0.165	0.051	0.001178	0.000371		0.184	0.92	0.012684	0.02478	0.6084536
rs12729914	1	77752823	C	T	0.188	1.00	0.177	0.05	0.0004003	0.0001792		0.161	0.95	0.003691	0.02543	0.8844689
rs11811611	1	77754605	C	T	0.812	1.00	-0.177	0.05	0.0003986	0.0001753		0.839	0.95	-0.00368	0.02543	0.8848547
rs12042177	1	77759226	A	G	0.812	0.97	-0.18	0.051	0.0003629	0.0001607		0.839	0.95	-0.00372	0.02553	0.8842102
rs634531	1	84639443	C	T	0.613	0.99	-0.146	0.04	0.0002327	0.0116615		0.554	0.93	0.01699	0.01911	0.3736599
rs3116406	1	84646830	C	G	0.660	0.99	-0.162	0.041	0.0000783	0.0013846		0.602	0.96	0.020661	0.01918	0.2810884
rs3768250	1	84652968	C	T	0.339	1.00	0.16	0.041	0.0000921	0.0015932		0.400	0.98	-0.0207	0.01896	0.2745021
rs1506702	1	84666643	C	T	0.339	1.00	0.159	0.041	0.0000951	0.0016336		0.400	0.98	-0.02084	0.01896	0.2714411
rs7514800	1	84669029	A	G	0.339	1.00	0.159	0.041	0.0001044	0.001584		0.400	0.98	-0.02113	0.01896	0.2649033
rs959363	1	84670909	A	C	0.648	0.98	-0.157	0.041	0.0001272	0.0029657		0.572	0.96	0.020855	0.01897	0.2711836
rs10874441	1	84671892	A	G	0.624	0.98	-0.153	0.04	0.0001318	0.0061985		0.551	0.98	0.017193	0.01862	0.3554314
rs1340598	1	84676687	C	T	0.326	1.00	0.15	0.041	0.000272	0.0024004		0.390	0.99	-0.02514	0.01895	0.1842781
rs10493746	1	84676867	A	G	0.323	1.00	0.145	0.041	0.0004143	0.0029572		0.386	0.99	-0.02234	0.019	0.2394702
rs4345821	1	84680558	C	T	0.326	1.00	0.15	0.041	0.000264	0.0023604		0.390	0.99	-0.02517	0.01894	0.1836149
rs12755232	1	84690644	A	C	0.674	0.99	-0.152	0.041	0.000238	0.0021509		0.610	0.99	0.025235	0.01895	0.1825268
rs12729133	1	84695141	C	T	0.682	0.96	-0.162	0.042	0.0001282	0.0010339		0.620	0.86	0.02668	0.02041	0.1908323
rs7540794	1	84696767	A	T	0.682	0.96	-0.162	0.042	0.000129	0.0010412		0.620	0.86	0.026711	0.02042	0.1904517
rs12142266	1	87070437	C	T	0.556	1.00	0.129	0.039	0.0009892	0.0031574		0.509	1.00	-0.02161	0.01837	0.2390936
rs4656013	1	88376745	C	T	0.421	0.50	-0.189	0.056	0.0006566	0.0002354		0.432	0.42	0.019907	0.0278	0.4736354
rs4658231	1	91814090	A	G	0.740	0.99	0.156	0.044	0.0004375	0.0036526		0.731	0.98	0.016604	0.02081	0.4246175
rs4658232	1	91814627	A	G	0.260	0.99	-0.157	0.044	0.0004271	0.0036769		0.268	0.98	-0.0169	0.02088	0.4180544
rs17131499	1	91833748	C	T	0.958	0.98	0.326	0.098	0.0008961	0.0414467		0.947	0.83	-0.02758	0.04933	0.5758122
rs12031250	1	91834033	C	T	0.042	1.00	-0.326	0.098	0.000845	0.0404283		0.053	0.83	0.027581	0.04933	0.5757524
rs12029135	1	91834662	C	T	0.958	1.00	0.326	0.098	0.0008415	0.040359		0.947	0.83	-0.02759	0.04932	0.5756153
rs12028899	1	91836938	C	T	0.042	1.00	-0.326	0.097	0.0008405	0.040341		0.053	0.84	0.027738	0.0493	0.5733321

					GOYA QC		GOYA Overweight/control			GOYA BMI continuous	known	IARC QC		IARC results		
MARKER	chr	position	A1	A2	FREQ1	Rsqr	Beta	SE	p	p	gene name	freq1	Rsqr	in_beta	in_SE	in_p
rs12024768	1	91838052	G	T	0.958	1.00	0.328	0.097	0.0007622	0.0376143		0.947	0.84	-0.02772	0.04929	0.5734966
rs4147836	1	94289062	C	T	0.744	1.00	-0.162	0.044	0.0002396	0.0032681		0.753	0.98	-0.00658	0.02122	0.7564448
rs4147833	1	94300951	C	T	0.755	0.99	-0.171	0.045	0.0001499	0.0055023		0.792	0.90	-0.01033	0.02346	0.6592984
rs4847273	1	94302331	A	G	0.697	1.00	-0.18	0.042	0.0000182	0.0010458		0.721	0.98	0.002635	0.02018	0.8960159
rs6661519	1	94302751	A	C	0.303	1.00	0.18	0.042	0.0000187	0.0010322		0.277	0.99	-0.00428	0.02018	0.8318412
rs1007347	1	94303106	C	T	0.303	1.00	0.179	0.042	0.0000206	0.0010804		0.279	0.98	-0.00537	0.02022	0.7902844
rs3789405	1	94303912	C	T	0.302	1.00	0.179	0.042	0.000021	0.0011		0.277	1.00	-0.00431	0.02013	0.8303025
rs3789407	1	94304194	C	G	0.251	1.00	0.175	0.045	0.0000877	0.0040132		0.231	0.98	0.009716	0.02145	0.6502905
rs4140392	1	94304601	C	T	0.749	1.00	-0.175	0.045	0.0000945	0.0042497		0.769	0.97	-0.0098	0.02152	0.6486732
rs1931575	1	94305602	C	T	0.261	0.99	0.183	0.045	0.0000393	0.0014561		0.271	0.85	0.006912	0.02191	0.7521825
rs2151849	1	94307762	A	G	0.780	1.00	-0.168	0.047	0.0003621	0.0056885		0.761	0.74	-0.01464	0.02454	0.5503492
rs3789412	1	94308655	C	T	0.779	1.00	-0.166	0.047	0.0003952	0.0064345		0.761	0.74	-0.01471	0.02459	0.5492375
rs2275035	1	94317748	C	T	0.733	1.00	-0.178	0.043	0.0000406	0.0026464		0.733	0.91	2.22E-05	0.0217	0.9991812
rs4847196	1	94327041	A	G	0.241	1.00	0.152	0.045	0.0007545	0.0134979		0.246	0.95	0.004032	0.0219	0.8538147
rs2068334	1	94332303	A	G	0.171	1.00	0.176	0.051	0.0006134	0.006824		0.198	0.85	-0.00515	0.0253	0.8385747
rs4147823	1	94333860	A	C	0.794	1.00	-0.158	0.047	0.0007506	0.0219814		0.781	0.88	-0.0097	0.02367	0.6815788
rs4147822	1	94334077	A	G	0.477	1.00	-0.141	0.039	0.0002677	0.0088589		0.471	0.65	-0.00449	0.02277	0.8435662
rs3789449	1	94355801	G	T	0.966	0.91	0.316	0.113	0.005025	0.000838		0.963	0.57	-0.00096	0.0645	0.9881445
rs11165433	1	95749386	A	G	0.051	0.92	-0.37	0.094	0.0000879	0.0001763		0.046	0.73	0.008416	0.05218	0.8717626
rs1582123	1	95750033	A	G	0.060	1.00	-0.322	0.083	0.0001001	0.0005944		0.070	1.00	-0.00162	0.03632	0.964453
rs945827	1	95754164	A	G	0.940	1.00	0.323	0.083	0.0000956	0.0005455		0.931	1.00	0.001605	0.03632	0.9647349
rs945826	1	95756744	A	G	0.940	0.99	0.323	0.083	0.0000945	0.000529		0.930	0.99	0.001278	0.0363	0.9718863
rs491043	1	95760562	A	G	0.939	0.99	0.32	0.083	0.0001042	0.0005705		0.930	0.99	0.001296	0.0363	0.9714965
rs7522301	1	95765869	C	T	0.061	0.99	-0.32	0.083	0.0001055	0.0005758		0.071	0.99	-0.00142	0.03628	0.9688589
rs611087	1	95768015	A	G	0.939	0.99	0.32	0.082	0.000107	0.0005825		0.929	0.98	0.001543	0.03627	0.966038
rs563232	1	95781289	C	T	0.939	0.98	0.321	0.082	0.0000995	0.0005549		0.929	0.98	0.001705	0.03625	0.9624614
rs12126657	1	104312973	C	G	0.038	0.84	-0.375	0.113	0.0009275	0.0014449		0.027	0.47	0.051297	0.07711	0.5055349
rs12144936	1	104484270	C	G	0.965	0.83	0.385	0.117	0.0009588	0.0013755		0.975	0.52	-0.01507	0.0756	0.8419014
rs1516155	1	105836120	A	G	0.425	0.97	0.135	0.04	0.0007113	0.0009367		0.430	0.96	0.011457	0.01908	0.5478244
rs611060	1	109583713	C	T	0.574	1.00	0.145	0.04	0.0002549	0.0006955		0.570	0.96	0.015354	0.01861	0.4089652
rs12043391	1	111655358	A	C	0.166	1.00	0.167	0.052	0.001418	0.0004759		0.175	0.99	0.003346	0.02444	0.8909942
rs197437	1	112076176	A	G	0.887	1.00	-0.21	0.061	0.000628	0.0043529		0.872	0.98	0.006791	0.02736	0.8038133
rs11102329	1	112078131	C	T	0.127	1.00	0.2	0.058	0.0005612	0.0026652		0.138	0.97	-0.00973	0.02658	0.7141083
rs197394	1	112087832	C	T	0.128	1.00	0.199	0.058	0.0005945	0.0030329		0.137	1.00	-0.01017	0.02628	0.6985277
rs197380	1	112094229	A	T	0.886	1.00	-0.202	0.061	0.000962	0.0060765		0.873	1.00	0.008019	0.02705	0.766699
rs197381	1	112094301	G	T	0.114	1.00	0.202	0.061	0.0009653	0.0060898		0.128	1.00	-0.00806	0.02705	0.7654966
rs191803	1	112101643	C	G	0.114	1.00	0.202	0.061	0.0009776	0.0061372		0.128	1.00	-0.00816	0.02705	0.7627303
rs197404	1	112104409	A	G	0.114	1.00	0.202	0.061	0.0009936	0.0061979		0.128	1.00	-0.0083	0.02704	0.7587296
rs197409	1	112109138	A	G	0.872	1.00	-0.193	0.058	0.000895	0.0039215		0.864	1.00	0.010975	0.02625	0.6756604
rs197410	1	112109398	C	T	0.128	1.00	0.192	0.058	0.0009194	0.0039894		0.137	1.00	-0.01106	0.02625	0.6732959
rs197411	1	112109701	A	G	0.128	1.00	0.192	0.058	0.0009257	0.0040065		0.137	1.00	-0.01115	0.02625	0.6707904
rs197414	1	112110646	A	C	0.128	1.00	0.192	0.058	0.0009447	0.0040586		0.137	1.00	-0.01136	0.02624	0.6647062
rs85276	1	112110854	C	T	0.128	1.00	0.192	0.058	0.0009447	0.0040586		0.137	1.00	-0.01136	0.02624	0.6647062
rs7522845	1	112113828	A	G	0.872	1.00	-0.192	0.058	0.0009419	0.004055		0.863	1.00	0.011183	0.02624	0.6697079
rs11803207	1	112116088	C	T	0.128	1.00	0.192	0.058	0.0009398	0.004052		0.137	1.00	-0.0111	0.02624	0.6720263
rs499817	1	112116345	A	G	0.872	1.00	-0.192	0.058	0.0009391	0.0040507		0.863	1.00	0.011079	0.02624	0.6726275
rs12138774	1	112119215	C	G	0.872	1.00	-0.192	0.058	0.0009425	0.0040865		0.863	1.00	0.010766	0.02626	0.6815252
rs11807526	1	149382621	A	C	0.823	1.00	-0.165	0.05	0.0009813	0.0018019		0.861	1.00	0.003336	0.02674	0.9006335
rs11204782	1	149384659	C	G	0.747	1.00	-0.138	0.044	0.001627	0.0003924		0.779	0.99	0.021322	0.02218	0.3360386
rs12087892	1	149385124	C	T	0.253	1.00	0.138	0.044	0.001627	0.0003929		0.221	1.00	-0.02131	0.02215	0.3356937
rs2067606	1	149395878	A	G	0.747	1.00	-0.138	0.044	0.00162	0.0003966		0.779	1.00	0.02118	0.02213	0.3382047
rs10494301	1	152967842	C	T	0.662	1.00	0.141	0.041	0.0005676	0.0199101		0.693	0.96	-0.02502	0.01997	0.2099458
rs11264489	1	154747455	A	G	0.624	0.99	-0.136	0.04	0.0006001	0.0024541		0.639	0.99	-0.0122	0.01901	0.5209081
rs6674079	1	154752685	A	G	0.629	1.00	-0.139	0.04	0.0005069	0.0023257		0.640	1.00	-0.01088	0.01893	0.5650697
rs1750307	1	154755044	A	T	0.376	1.00	0.136	0.04	0.0006218	0.0024901		0.360	1.00	0.012057	0.01893	0.523922
rs4971115	1	156096326	A	G	0.052	0.73	0.34	0.102	0.0008852	0.0103004		0.056	0.62	-0.01672	0.05077	0.7416829
rs17690088	1	156101482	C	T	0.948	0.74	-0.338	0.102	0.0008754	0.0100599		0.943	0.64	0.018718	0.04972	0.7063106
rs17656210	1	157161483	G	T	0.065	0.78	0.211	0.089	0.01792	0.0008863		0.062	0.72	0.025707	0.04375	0.5564439
rs16841398	1	157212332	A	G	0.931	0.85	0.222	0.084	0.008167	0.0006924		0.897	0.77	0.001522	0.03436	0.9646398
rs6695377	1	157840074	C	T	0.768	1.00	0.136	0.046	0.003208	0.000187		0.728	1.00	-0.03744	0.02088	0.0727454
rs12048222	1	160560718	A	G	0.752	1.00	0.108	0.045	0.0163	0.0009741		0.765	0.89	-0.00679	0.02306	0.768203
rs10917872	1	162122580	A	G	0.483	1.00	-0.129	0.039	0.001102	0.000551		0.484	0.99	-0.0272	0.01822	0.1351735
rs6669526	1	162123655	C	T	0.543	0.97	0.132	0.04	0.000952	0.0009316		0.544	0.96	0.024697	0.01844	0.1802223
rs945794	1	162416026	C	T	0.015	0.66	0.617	0.2	0.001992	0.0004125		0.012	0.58	0.069779	0.10554	0.5081557
rs6675659	1	165436133	A	T	0.473	0.92	-0.139	0.041	0.0005953	0.0032136		0.587	0.82	-0.04122	0.01978	0.0370614
rs10800292	1	165439062	A	G	0.521	0.88	0.143	0.041	0.0005571	0.0025608		0.407	0.79	0.04185	0.02027	0.0388691
rs16848281	1	173589465	C	T	0.089	1.00	-0.224	0.068	0.0009948	0.0002919		0.103	1.00	-0.02617	0.03143	0.4045599
rs10912966	1	173590009	A	G	0.085	0.96	-0.237	0.071	0.0007896	0.0002129		0.098	0.95	-0.02842	0.03288	0.3869687
rs16848304	1	173602808	C	G	0.089	1.00	-0.225	0.068	0.0009394	0.0002802		0.103	1.00	-0.02672	0.03146	0.3953169
rs17377915	1	173978650	A	G	0.031	0.43	-0.593	0.175	0.0007298	0.0009339		0.032	0.15	-0.09398	0.13623	0.4899132
rs4478775	1	176036720	A	G	0.242	1.00	0.137	0.046	0.002753	0.0007502	LZTR2					
rs11585009	1	176037491	A	C	0.242	1.00	0.137	0.046	0.002667	0.0007296	LZTR2					
rs4618929	1	176039338	C	T	0.691	1.00	0.147	0.042	0.0005163	0.0003076	LZTR2					
rs4442331	1	176039438	A	G	0.669	1.00	0.14	0.042	0.0007284	0.000256	LZTR2					
rs4532784	1	176039722	C	G	0.669	1.00	0.141	0.042	0.0007105	0.0002488	LZTR2					
rs4650976	1	176044073	C	G	0.362	1.00	-0.158	0.041	0.0001031	0.0000373	LZTR2					
rs4650977	1	176044231	C	G	0.638	1.00	0.158	0.041	0.0001018	0.0000367	LZTR2					
rs12135579	1	176044714	C	T	0.758	1.00	-0.14	0.046	0.002207	0.000609	LZTR2					
rs6668375	1	176047907	C	T	0.950	0.84	0.328	0.098	0.0008108	0.0009957	LZTR2					
rs10798570	1	176054631	G	T	0.082	1.00	-0.242	0.072	0.0007258	0.0006115	LZTR2					
rs10798571	1	176054803	C	T	0.082	1.00	-0.242	0.072	0.0007215	0.0006088	LZTR2					
rs4081367	1	176056685	C	T	0.082	1.00	-0.243	0.072	0.0006871	0.0005871	LZTR2					

					GOYA QC		GOYA Overweight/control			GOYA BMI continuous	known	IARC QC		IARC results		
MARKER	chr	position	A1	A2	FREQ1	Rsqr	Beta	SE	p	p	gene name	freq1	Rsqr	in_beta	in_SE	in_p
rs10913435	1	176057334	A	T	0.082	1.00	-0.243	0.072	0.0006752	0.0005793	LZTR2					
rs11586243	1	176065460	C	T	0.227	1.00	0.134	0.046	0.003562	0.0009347	LZTR2					
rs4509539	1	176066511	C	T	0.227	1.00	0.134	0.046	0.003467	0.0009158	LZTR2					
rs4132288	1	176069333	A	G	0.227	1.00	0.134	0.046	0.003421	0.0009065	LZTR2					
rs12760319	1	176070814	A	G	0.264	0.90	0.126	0.046	0.006268	0.0004578	LZTR2					
rs2902200	1	176073461	A	G	0.773	1.00	-0.135	0.046	0.003256	0.0008737	LZTR2					
rs10913445	1	176076183	C	G	0.181	0.81	0.177	0.056	0.001446	0.0003792	LZTR2					
rs12737338	1	176078562	C	T	0.775	1.00	-0.147	0.046	0.001544	0.0004767	LZTR2					
rs12757638	1	176082392	A	G	0.229	0.99	0.157	0.046	0.0006824	0.0002647	LZTR2					
rs3131312	1	176087103	C	T	0.082	1.00	-0.249	0.071	0.0004858	0.0006244	LZTR2					
rs3131315	1	176089802	A	T	0.918	1.00	0.249	0.071	0.0004904	0.0006065	LZTR2					
rs3131318	1	176092026	A	C	0.083	1.00	-0.249	0.071	0.0004774	0.0005576	LZTR2					
rs3131319	1	176092455	G	T	0.788	1.00	0.158	0.048	0.0009885	0.0028158	LZTR2					
rs1571347	1	176093961	C	T	0.212	1.00	-0.158	0.048	0.0009749	0.0027742	LZTR2					
rs1410410	1	176095359	A	G	0.083	1.00	-0.249	0.071	0.0004744	0.000545	LZTR2					
rs3131321	1	176096081	A	G	0.083	1.00	-0.249	0.071	0.0004699	0.0005218	LZTR2					
rs3131322	1	176097153	C	T	0.788	1.00	0.159	0.048	0.0008951	0.0025305	LZTR2					
rs4142027	1	176100199	C	T	0.792	0.81	-0.173	0.052	0.0009044	0.0002863	LZTR2					
rs1536669	1	176100700	C	T	0.911	0.94	0.248	0.071	0.0004909	0.000704	LZTR2					
rs943763	1	176102887	A	C	0.083	1.00	-0.25	0.071	0.0004568	0.0004724	LZTR2					
rs2902202	1	176108518	A	G	0.762	1.00	0.14	0.046	0.002182	0.0001516	LZTR2					
rs633715	1	176119203	C	T	0.229	0.99	0.185	0.046	0.000057	0.0000224	LZTR2					
rs10913454	1	176119412	A	C	0.594	0.99	-0.148	0.039	0.0001804	0.0000772	LZTR2					
rs10798577	1	176119843	C	G	0.406	0.99	0.148	0.039	0.0001809	0.0000774	LZTR2					
rs10798578	1	176123548	C	T	0.380	0.99	0.141	0.04	0.0003933	0.0001697	LZTR2					
rs1336785	1	176126265	C	G	0.620	0.99	-0.141	0.04	0.0003955	0.0001705	LZTR2					
rs527065	1	176135505	G	T	0.645	1.00	-0.193	0.041	0.00000204	0.000000593	LZTR2					
rs685233	1	176136564	A	G	0.645	1.00	-0.193	0.041	0.00000207	0.000000591	LZTR2					
rs623130	1	176138509	C	T	0.355	1.00	0.192	0.041	0.00000212	0.000000589	LZTR2					
rs574367	1	176139833	G	T	0.771	1.00	-0.183	0.046	0.0000676	0.0000232	LZTR2					
rs10157480	1	176139956	A	G	0.405	1.00	0.147	0.039	0.0001916	0.0000709	LZTR2					
rs576101	1	176140006	A	T	0.355	1.00	0.192	0.041	0.0000022	0.000000586	LZTR2					
rs527248	1	176142137	A	G	0.771	1.00	-0.183	0.046	0.0000697	0.0000237	LZTR2					
rs589500	1	176143569	C	T	0.771	1.00	-0.182	0.046	0.0000704	0.0000239	LZTR2					
rs604388	1	176144602	C	T	0.355	1.00	0.192	0.041	0.00000221	0.000000584	LZTR2					
rs693232	1	176148274	C	G	0.771	1.00	-0.182	0.046	0.0000722	0.0000245	LZTR2					
rs617763	1	176151861	C	T	0.645	1.00	-0.191	0.041	0.00000235	0.000000621	LZTR2					
rs618759	1	176152111	A	G	0.645	1.00	-0.19	0.041	0.00000261	0.000000686	LZTR2					
rs630372	1	176152385	A	G	0.254	1.00	0.181	0.044	0.0000452	0.0000152	LZTR2					
rs10913461	1	176153740	C	G	0.406	1.00	0.144	0.039	0.0002511	0.0000936	LZTR2					
rs512898	1	176155052	C	T	0.357	1.00	0.187	0.04	0.00000364	0.000000948	LZTR2					
rs543874	1	176156103	A	G	0.776	1.00	-0.185	0.046	0.0000651	0.0000223	LZTR2					
rs506589	1	176160910	C	T	0.225	1.00	0.184	0.046	0.0000657	0.0000226	LZTR2					
rs8030	1	176164598	C	T	0.673	1.00	-0.186	0.041	0.00000634	0.00000195	LZTR2					
rs545608	1	176165744	C	G	0.225	1.00	0.184	0.046	0.0000661	0.0000228	LZTR2					
rs575908	1	176166721	C	T	0.327	1.00	0.185	0.041	0.00000731	0.00000223	LZTR2					
rs10489882	1	176171118	C	T	0.400	1.00	-0.133	0.04	0.0008852	0.0017524	LZTR2					
rs2902210	1	176171797	C	T	0.578	1.00	-0.137	0.039	0.0004204	0.0001236	LZTR2					
rs10913469	1	176180142	C	T	0.226	1.00	0.163	0.046	0.0003918	0.0000394	LZTR2					
rs3935288	1	176801440	C	T	0.894	0.87	-0.208	0.067	0.002052	0.000726	LZTR2					
rs16853281	1	176938373	A	G	0.145	1.00	0.169	0.055	0.002103	0.0007939	LZTR2					
rs6682262	1	179791184	A	T	0.599	0.80	0.146	0.044	0.0009865	0.0030411		0.628	0.55	0.032308	0.02546	0.2040888
rs10911556	1	182549999	A	G	0.196	1.00	0.157	0.049	0.001385	0.0004244		0.177	0.97	0.032225	0.02396	0.1783248
rs10911557	1	182550129	C	T	0.805	1.00	-0.158	0.049	0.001311	0.0003958		0.823	0.97	-0.03209	0.02395	0.1800477
rs9645302	1	182554400	A	G	0.195	1.00	0.159	0.049	0.001214	0.0003591		0.177	0.97	0.032007	0.02395	0.1810395
rs12096575	1	182555443	A	G	0.195	1.00	0.159	0.049	0.00119	0.0003496		0.177	0.97	0.031956	0.02394	0.1816614
rs4618934	1	182557248	G	T	0.195	1.00	0.16	0.049	0.00113	0.0003372		0.176	0.98	0.031248	0.02389	0.1905552
rs12727053	1	182557707	A	T	0.805	1.00	-0.16	0.049	0.001118	0.0003348		0.824	0.98	-0.03122	0.02389	0.1909555
rs12727591	1	182557762	A	G	0.196	1.00	0.16	0.049	0.001089	0.0003295		0.176	0.98	0.031169	0.02388	0.1915455
rs10911560	1	182558865	A	G	0.804	1.00	-0.167	0.049	0.0006728	0.0002334		0.821	1.00	-0.0286	0.02371	0.2274591
rs1930293	1	182560790	C	T	0.197	1.00	0.166	0.049	0.0006676	0.0002337		0.179	1.00	0.028464	0.0237	0.2292836
rs12074351	1	182567036	A	G	0.803	1.00	-0.167	0.049	0.0006729	0.0002413		0.819	0.95	-0.02797	0.02409	0.2452956
rs12059311	1	182569117	A	G	0.197	0.98	0.169	0.049	0.000636	0.0002172		0.184	0.91	0.026846	0.0244	0.2708455
rs4568789	1	182581034	A	G	0.289	0.99	0.133	0.044	0.002265	0.000691		0.286	0.98	0.01919	0.02046	0.347783
rs13374592	1	182638826	A	G	0.946	1.00	-0.234	0.086	0.006434	0.0006052		0.965	0.93	0.005298	0.05054	0.9164446
rs12136470	1	182642675	A	C	0.054	1.00	0.233	0.086	0.006822	0.0006639		0.034	0.96	-0.00089	0.05031	0.9858724
rs12130008	1	182643212	A	T	0.946	1.00	-0.233	0.086	0.006865	0.0006706		0.966	0.96	0.000863	0.0503	0.986295
rs12128343	1	182650421	A	T	0.054	1.00	0.228	0.086	0.008009	0.000856		0.034	0.97	-0.0007	0.0503	0.9889644
rs12129504	1	182651474	C	T	0.054	1.00	0.227	0.086	0.008223	0.0008925		0.034	0.97	-0.00065	0.0503	0.9896657
rs12142568	1	182653794	C	G	0.946	1.00	-0.226	0.086	0.008466	0.0009161		0.967	0.98	-0.00043	0.05023	0.9932103
rs11799574	1	182657902	A	C	0.054	1.00	0.226	0.086	0.008581	0.0009264		0.033	0.98	0.00046	0.05023	0.9926886
rs12128424	1	182659714	A	G	0.054	1.00	0.226	0.086	0.008697	0.000937		0.033	0.98	0.000496	0.05023	0.9921166
rs12145326	1	182661579	C	T	0.946	1.00	-0.225	0.086	0.00882	0.0009486		0.967	0.98	-0.00052	0.05023	0.9917388
rs12745536	1	182872304	A	G	0.032	0.55	0.43	0.15	0.004137	0.000633		0.038	0.57	-0.16751	0.06585	0.0109215
rs861578	1	182995655	C	G	0.437	0.98	-0.111	0.04	0.004844	0.000867		0.493	0.96	-0.00211	0.01847	0.9090284
rs861581	1	182998892	G	T	0.437	0.98	-0.111	0.04	0.004868	0.0008753		0.493	0.96	-0.00214	0.01847	0.9076887
rs338563	1	183043026	C	T	0.782	0.99	-0.16	0.047	0.0007041	0.001425		0.835	0.98	-0.02626	0.02455	0.2842683
rs338565	1	183043724	A	G	0.218	0.99	0.16	0.047	0.0007047	0.001427		0.165	0.98	0.025938	0.02451	0.2895484
rs543912	1	183043800	A	C	0.218	0.99	0.16	0.047	0.0007043	0.00143		0.165	0.98	0.025873	0.0245	0.2906107
rs338566	1	183044090	A	T	0.717	0.99	-0.149	0.044	0.0006299	0.0005503		0.784	0.99	-0.02746	0.022	0.2116813
rs338567	1	183044436	C	T	0.218	0.99	0.16	0.047	0.0007028	0.0014316		0.166	1.00	0.024375	0.02433	0.3160363
rs338568	1	183044503	A	C	0.218	0.99	0.16	0.047	0.0007027	0.0014367		0.166	1.00	0.024325	0.02433	0.3169382

					GOYA QC		GOYA Overweight/control			GOYA BMI continuous	known	IARC QC		IARC results		
MARKER	chr	position	A1	A2	FREQ1	Rsqr	Beta	SE	p	p	gene name	freq1	Rsqr	in_beta	in_SE	in_p
rs10797988	1	183050322	C	T	0.283	0.99	0.149	0.044	0.0006383	0.000564		0.216	0.99	0.026904	0.02194	0.2198408
rs10797989	1	183061009	C	G	0.282	0.99	0.148	0.044	0.0006733	0.0006044		0.216	0.99	0.027014	0.02193	0.2176709
rs4651234	1	183065966	C	T	0.282	0.99	0.147	0.044	0.0007587	0.0006895		0.215	0.99	0.026895	0.02198	0.2207576
rs481215	1	183067018	G	T	0.783	0.99	-0.157	0.047	0.0009188	0.0020186		0.834	1.00	-0.02485	0.02436	0.3074636
rs906285	1	183067253	A	G	0.718	0.99	-0.147	0.044	0.0007473	0.0006668		0.785	0.99	-0.02685	0.02199	0.2217052
rs484847	1	183067380	C	T	0.783	0.99	-0.157	0.047	0.0009255	0.0020434		0.835	0.99	-0.02493	0.02448	0.307961
rs1321999	1	183791541	A	G	0.949	0.96	-0.299	0.089	0.0008018	0.0018979		0.964	0.88	-0.0012	0.04972	0.980786
rs10911755	1	183792666	A	C	0.949	0.96	-0.299	0.089	0.0007898	0.0018588		0.964	0.88	-0.00099	0.04963	0.9840945
rs11586588	1	183799928	A	G	0.052	0.98	0.291	0.088	0.0009185	0.002048		0.037	0.94	-0.00566	0.04834	0.9067531
rs11583193	1	183807241	A	C	0.051	1.00	0.299	0.088	0.0006485	0.0014608		0.035	1.00	-0.00088	0.04785	0.9853917
rs12402607	1	183810498	A	G	0.949	1.00	-0.299	0.088	0.0006655	0.0014961		0.965	1.00	0.0009	0.04786	0.9849921
rs12048982	1	183819769	G	T	0.051	1.00	0.298	0.088	0.0006904	0.0015444		0.035	1.00	-0.00091	0.04789	0.9848163
rs12046138	1	183820147	A	G	0.051	1.00	0.298	0.088	0.0006923	0.0015459		0.035	1.00	-0.00093	0.0479	0.9844552
rs10921053	1	190234692	A	G	0.662	1.00	0.147	0.041	0.0003205	0.0003069		0.639	0.99	-0.00759	0.01918	0.6921842
rs6428079	1	190250481	A	C	0.662	1.00	0.147	0.041	0.0003278	0.0003247		0.639	1.00	-0.00728	0.01912	0.7030105
rs3012198	1	190317297	A	C	0.228	0.95	-0.158	0.046	0.0006562	0.0015822		0.276	0.76	0.008051	0.02355	0.7321771
rs12736318	1	190357914	A	G	0.774	0.96	0.155	0.046	0.0007899	0.0017493		0.725	0.74	-0.00626	0.02389	0.7931893
rs6658573	1	192954888	A	G	0.052	0.98	-0.285	0.089	0.001406	0.0003123		0.100	0.98	0.040516	0.03058	0.1848579
rs10921677	1	193081013	A	C	0.027	0.79	-0.375	0.136	0.005865	0.0008724		0.083	0.82	0.055744	0.03587	0.1198837
rs1171215	1	193082284	A	G	0.973	0.79	0.375	0.136	0.005875	0.0008744		0.917	0.82	-0.05575	0.03587	0.1198668
rs494902	1	193115277	C	T	0.960	0.98	0.257	0.101	0.01113	0.0009444		0.898	0.96	-0.05947	0.03095	0.0545228
rs555096	1	193168212	C	T	0.960	0.98	0.269	0.101	0.007769	0.0006472		0.893	0.95	-0.06006	0.03062	0.0496615
rs530237	1	193169917	A	C	0.041	0.98	-0.269	0.101	0.007764	0.0006462		0.107	0.95	0.06005	0.03062	0.0497092
rs1032828	1	193183324	A	C	0.145	0.97	-0.185	0.056	0.0009015	0.0163572		0.126	0.94	-0.03083	0.02919	0.2904963
rs866059	1	193212635	C	T	0.961	0.95	0.279	0.104	0.007422	0.0006189		0.893	0.95	-0.05299	0.03103	0.087444
rs12035121	1	193227287	C	T	0.039	0.95	-0.279	0.104	0.007412	0.0006199		0.107	0.94	0.052978	0.03105	0.0876914
rs12044689	1	193304925	A	G	0.039	0.94	-0.288	0.105	0.006047	0.0004726		0.107	0.94	0.055496	0.03118	0.0749253
rs17686400	1	194217909	C	T	0.943	1.00	0.32	0.084	0.0001414	0.00207		0.951	0.99	-0.03381	0.04312	0.4325697
rs17686963	1	194241413	A	G	0.057	0.99	-0.335	0.085	0.0000831	0.0015669		0.046	0.93	0.025356	0.04565	0.5782625
rs17687163	1	194252008	A	C	0.944	0.98	0.339	0.085	0.0000738	0.001458		0.955	0.93	-0.02514	0.0458	0.5826744
rs12737611	1	194254212	C	T	0.944	0.98	0.339	0.085	0.0000726	0.0014429		0.955	0.92	-0.02505	0.04583	0.584316
rs12735954	1	194257569	A	G	0.944	0.98	0.342	0.086	0.0000689	0.0013931		0.955	0.88	-0.02489	0.04689	0.5952772
rs2819361	1	200246742	C	G	0.341	0.91	-0.154	0.043	0.0002904	0.0006804		0.320	0.79	-0.02461	0.0216	0.2540124
rs12074979	1	200866087	C	G	0.024	0.70	-0.458	0.154	0.002862	0.0006478		0.028	0.22	0.029274	0.12077	0.8083141
rs6699298	1	201120216	C	T	0.756	1.00	-0.149	0.045	0.0009193	0.0002886		0.781	0.99	-0.05866	0.02173	0.006911
rs3806346	1	201126223	A	G	0.756	1.00	-0.149	0.045	0.0008994	0.0002806		0.781	0.99	-0.05878	0.02171	0.0067385
rs3766572	1	201126690	C	T	0.244	1.00	0.149	0.045	0.0008867	0.0002752		0.219	0.99	0.058806	0.0217	0.0067028
rs10920519	1	201132954	A	C	0.756	1.00	-0.149	0.045	0.0008838	0.0002742		0.782	1.00	-0.05891	0.02168	0.0065613
rs7542125	1	201137861	C	T	0.244	1.00	0.149	0.045	0.0008789	0.0002722		0.218	1.00	0.058923	0.02168	0.0065352
rs7536140	1	201145431	C	T	0.758	1.00	-0.146	0.045	0.00117	0.0003457		0.782	1.00	-0.05913	0.02167	0.006339
rs10800877	1	201150686	C	T	0.758	1.00	-0.148	0.045	0.001036	0.0003186		0.782	1.00	-0.05935	0.02168	0.0061595
rs10800880	1	201159540	C	T	0.242	1.00	0.148	0.045	0.0009611	0.0003045		0.218	1.00	0.059276	0.02171	0.0062885
rs2363772	1	201165984	A	C	0.216	0.99	0.148	0.047	0.001572	0.0005109		0.193	0.99	0.049195	0.02248	0.0285042
rs2185781	1	201173129	C	T	0.785	1.00	-0.146	0.047	0.001817	0.0006449		0.808	1.00	-0.04821	0.02244	0.0315212
rs4336908	1	201175159	A	G	0.217	0.98	0.145	0.047	0.002006	0.0006833		0.194	0.94	0.047543	0.02301	0.0386633
rs2802782	1	201838389	G	T	0.492	0.81	0.158	0.043	0.0002165	0.0046437		0.468	0.73	0.03067	0.02151	0.1536859
rs9645427	1	201854029	C	G	0.601	0.98	-0.131	0.04	0.0009123	0.0248328		0.644	0.88	-0.02475	0.02059	0.2289681
rs12120868	1	201854161	G	T	0.445	0.98	0.137	0.039	0.0004708	0.0059524		0.399	0.92	0.03926	0.01943	0.0431839
rs4301679	1	202271641	C	T	0.706	0.71	-0.167	0.05	0.0009047	0.0015703		0.702	0.61	-0.03534	0.02608	0.174999
rs12133634	1	202281230	C	T	0.181	0.98	0.183	0.051	0.000344	0.0001056		0.145	0.95	0.004567	0.02635	0.8622903
rs12133665	1	202281316	C	T	0.181	0.99	0.182	0.051	0.0003402	0.0001034		0.145	0.95	0.004581	0.02634	0.8618162
rs11802889	1	202876675	C	G	0.129	0.97	0.179	0.059	0.002264	0.0009285		0.162	0.77	0.038769	0.02891	0.1795071
rs17413714	1	202899963	A	C	0.767	0.99	-0.168	0.046	0.0002227	0.0014322		0.750	0.59	0.020485	0.02696	0.4469103
rs7516408	1	203192569	C	T	0.756	1.00	0.113	0.046	0.01319	0.0005829		0.779	0.99	-0.00821	0.02151	0.7024643
rs17415030	1	203192996	A	G	0.763	1.00	0.106	0.046	0.02071	0.0009121		0.786	1.00	-0.0089	0.02166	0.6808122
rs7519658	1	203193603	C	T	0.763	1.00	0.106	0.046	0.02078	0.0009168		0.786	1.00	-0.00926	0.02164	0.6684241
rs2275696	1	203193759	A	G	0.756	1.00	0.112	0.045	0.01362	0.0005809		0.777	1.00	-0.00674	0.02133	0.7516365
rs11810663	1	203194041	A	G	0.763	1.00	0.105	0.046	0.02159	0.0009616		0.786	1.00	-0.00937	0.02165	0.6649569
rs12740109	1	203197700	A	G	0.220	0.98	-0.125	0.048	0.009365	0.0006651		0.203	0.93	0.0162	0.02282	0.4773349
rs17045468	1	205723998	C	T	0.990	0.88	-0.571	0.209	0.006189	0.0003321		0.993	0.61	-0.03087	0.13359	0.8171151
rs879047	1	206451877	A	G	0.167	0.85	0.159	0.057	0.005477	0.000922		0.149	0.76	-0.02154	0.02878	0.4537003
rs1262046	1	206467115	C	T	0.188	0.99	0.142	0.051	0.005284	0.0008865		0.194	0.99	-0.00631	0.02307	0.784148
rs2590681	1	206474126	C	T	0.834	0.88	-0.164	0.056	0.003745	0.0003957		0.838	0.84	0.007057	0.0265	0.7898474
rs2590680	1	206476063	A	G	0.816	0.90	-0.153	0.054	0.004325	0.0006604		0.808	0.87	0.007225	0.02461	0.7688861
rs12750718	1	206633240	A	G	0.824	0.72	-0.175	0.06	0.003703	0.0004307		0.854	0.65	-0.00569	0.03201	0.8588243
rs7534677	1	207306919	C	G	0.702	1.00	-0.147	0.042	0.0004904	0.0002879		0.623	0.99	0.012866	0.0189	0.4955748
rs12130212	1	207793880	A	T	0.564	0.73	0.134	0.046	0.003429	0.0004863		0.581	0.65	0.064429	0.02274	0.004592
rs6683256	1	207798165	C	T	0.657	0.98	0.129	0.041	0.001893	0.0008191		0.660	0.90	0.050383	0.02051	0.0139774
rs2356934	1	207830834	C	T	0.922	0.70	0.254	0.088	0.003662	0.0006558		0.947	0.47	0.00269	0.05767	0.962767
rs2028706	1	209898003	A	C	0.783	1.00	0.157	0.047	0.0008322	0.0017777		0.737	1.00	0.017976	0.0209	0.3893778
rs12031285	1	209903412	C	T	0.217	1.00	-0.158	0.047	0.0007979	0.0016417		0.263	1.00	-0.01786	0.02091	0.3927312
rs11119789	1	209904019	C	T	0.814	0.96	0.179	0.051	0.0004387	0.0013767		0.789	0.95	0.001214	0.02321	0.9582444
rs10159325	1	209904754	C	G	0.141	0.94	-0.197	0.058	0.0006641	0.0004222		0.185	0.95	-0.0153	0.02445	0.531074
rs6698450	1	209910643	C	G	0.784	1.00	0.16	0.047	0.0006947	0.0014101		0.737	1.00	0.017638	0.02093	0.3989317
rs11119792	1	209916592	C	T	0.784	1.00	0.163	0.047	0.0005643	0.0011566		0.737	1.00	0.017364	0.02094	0.4064795
rs7551769	1	209923033	A	G	0.216	1.00	-0.165	0.047	0.0004803	0.0009782		0.262	1.00	-0.01755	0.02096	0.4019007
rs10494939	1	209923118	C	T	0.216	1.00	-0.166	0.047	0.0004548	0.0009125		0.262	1.00	-0.01757	0.02096	0.4013814
rs1656023	1	211592523	C	T	0.768	0.99	-0.169	0.046	0.0002527	0.0002733		0.766	0.99	0.014584	0.02159	0.4990693
rs1656022	1	211593865	A	G	0.681	1.00	-0.154	0.042	0.0002593	0.0004085		0.675	1.00	0.012944	0.01931	0.5022713
rs1656021	1	211594211	C	T	0.319	0.99	0.155	0.042	0.0002596	0.0004111		0.325	0.97	-0.01312	0.0195	0.5008726
rs7528988	1	213315040	C	T	0.780	1.00	0.154	0.047	0.0009779	0.0029982		0.748	1.00	0.007428	0.02136	0.7278273
rs17667371	1	215872635	A	G	0.963	0.85	0.312	0.113	0.005621	0.0004923		0.970	0.77	-0.05215	0.05843	0.3717394

					GOYA QC		GOYA Overweight/control			GOYA BMI continuous	known	IARC QC		IARC results		
MARKER	chr	position	A1	A2	FREQ1	Rsqr	Beta	SE	p	p	gene name	freq1	Rsqr	in_beta	in_SE	in_p
rs17046740	1	215902143	A	C	0.959	0.88	0.311	0.105	0.002996	0.0003475		0.969	0.80	-0.05144	0.05588	0.3569416
rs10495068	1	215908803	C	T	0.043	0.90	-0.304	0.102	0.002941	0.0003645		0.032	0.80	0.05008	0.05573	0.3684877
rs17675740	1	216037584	A	G	0.072	0.98	-0.245	0.077	0.001396	0.0005274		0.053	0.94	0.031528	0.03817	0.4084404
rs17046879	1	216055212	C	G	0.931	0.99	0.244	0.078	0.001742	0.0008841		0.956	0.88	-0.02433	0.04183	0.56051
rs1567448	1	216085915	A	G	0.069	0.99	-0.243	0.078	0.00183	0.0009278		0.044	0.88	0.023779	0.042	0.5709527
rs1567447	1	216085933	C	G	0.069	0.99	-0.243	0.078	0.001834	0.0009323		0.044	0.88	0.023726	0.04203	0.5720918
rs12130346	1	216446178	C	T	0.953	0.66	0.373	0.114	0.001074	0.0005205		0.957	0.73	0.057601	0.05522	0.2965155
rs10863429	1	217366417	C	T	0.138	0.98	0.198	0.057	0.0004742	0.0012687		0.119	0.95	-0.01809	0.02798	0.517561
rs12067803	1	217373546	A	G	0.834	0.87	-0.19	0.056	0.0007082	0.0018855		0.845	0.84	0.004642	0.02678	0.8622794
rs7543820	1	217374522	A	G	0.129	0.98	0.196	0.058	0.0007731	0.0026785		0.123	0.96	-0.0161	0.02767	0.5603868
rs7523306	1	217387469	A	G	0.129	0.99	0.195	0.058	0.0008015	0.0028242		0.123	0.97	-0.01641	0.02762	0.552084
rs7539768	1	217387802	G	T	0.129	0.99	0.195	0.058	0.0008055	0.0028445		0.123	0.97	-0.01642	0.02762	0.551766
rs12079503	1	217389901	C	T	0.129	0.99	0.195	0.058	0.0008097	0.0028652		0.122	0.97	-0.01645	0.02762	0.5512273
rs6657059	1	217398681	G	T	0.128	0.99	0.194	0.058	0.0008547	0.0030887		0.122	0.98	-0.01667	0.02758	0.5452065
rs11118229	1	217399920	C	T	0.872	0.99	-0.194	0.058	0.0008619	0.0031227		0.878	0.98	0.016677	0.02756	0.5448398
rs3748635	1	217403711	A	T	0.872	0.99	-0.194	0.058	0.0008828	0.0032203		0.878	0.98	0.016834	0.02752	0.5404048
rs6699816	1	217422412	A	G	0.873	1.00	-0.193	0.058	0.0009306	0.0034519		0.879	1.00	0.017111	0.02743	0.5324734
rs12093892	1	217434971	G	T	0.873	1.00	-0.192	0.058	0.0009843	0.0034121		0.878	1.00	0.016351	0.02741	0.550419
rs12080335	1	217526610	C	T	0.130	1.00	0.199	0.058	0.000601	0.0015968		0.120	0.99	-0.01245	0.02759	0.6516031
rs17005229	1	217530107	A	G	0.130	1.00	0.199	0.058	0.0006157	0.0016418		0.120	0.98	-0.01187	0.02765	0.6674741
rs17578266	1	219468302	C	G	0.975	0.65	0.627	0.161	0.0000952	0.0001485		0.966	0.50	0.017114	0.08115	0.8328335
rs1442440	1	220306157	C	T	0.377	1.00	0.147	0.04	0.0002279	0.0007273		0.390	1.00	-0.01819	0.01877	0.3319592
rs7513781	1	220306555	C	T	0.414	1.00	0.135	0.039	0.0005849	0.0012584		0.418	0.96	-0.02037	0.01893	0.2815672
rs7556050	1	220306615	A	G	0.586	1.00	-0.135	0.039	0.0005774	0.0012464		0.582	0.96	0.020412	0.01894	0.2806416
rs1339875	1	220306945	G	T	0.416	1.00	0.132	0.039	0.0007593	0.0017102		0.423	0.97	-0.02084	0.01881	0.2676678
rs1856620	1	220307002	C	T	0.416	1.00	0.132	0.039	0.0007492	0.0016929		0.423	0.97	-0.02085	0.01882	0.2674051
rs1339876	1	220307041	A	T	0.584	1.00	-0.132	0.039	0.0007382	0.0016756		0.577	0.97	0.020876	0.01882	0.2668632
rs7548749	1	220308311	A	G	0.404	1.00	0.135	0.039	0.0005613	0.0011997		0.411	0.97	-0.0198	0.0189	0.2942705
rs12038701	1	220309400	A	G	0.416	1.00	0.132	0.039	0.0007318	0.0016637		0.423	0.97	-0.02092	0.01882	0.2658839
rs11118934	1	220310078	C	T	0.379	1.00	0.145	0.04	0.0002772	0.0009501		0.395	0.98	-0.0194	0.01893	0.3052125
rs12134885	1	220311003	C	T	0.379	1.00	0.145	0.04	0.0002728	0.000941		0.395	0.97	-0.01953	0.01895	0.3022365
rs10863680	1	220311503	C	T	0.379	1.00	0.145	0.04	0.0002718	0.0009384		0.395	0.97	-0.01957	0.01895	0.3012845
rs10863682	1	220314598	C	T	0.406	1.00	0.163	0.039	0.0000361	0.0001476		0.419	0.96	-0.00126	0.01879	0.9465511
rs1890757	1	220315305	A	C	0.557	1.00	-0.15	0.039	0.0001107	0.0002705		0.552	0.99	0.002967	0.01837	0.8715628
rs1890758	1	220315446	A	G	0.557	1.00	-0.15	0.039	0.0001142	0.0002679		0.552	0.99	0.002636	0.01836	0.8857536
rs12096184	1	220317058	A	G	0.557	1.00	-0.15	0.039	0.0001111	0.0002697		0.552	0.99	0.002975	0.01836	0.8712019
rs11118936	1	220317666	A	G	0.557	1.00	-0.15	0.039	0.0001109	0.0002694		0.552	0.99	0.002973	0.01836	0.8712733
rs10863684	1	220317955	A	G	0.406	1.00	0.163	0.039	0.0000367	0.000148		0.419	0.96	-0.00133	0.01878	0.9434801
rs11118937	1	220318050	C	T	0.406	1.00	0.163	0.039	0.0000367	0.0001481		0.419	0.96	-0.00132	0.01878	0.9439753
rs7525314	1	220318131	C	T	0.557	1.00	-0.15	0.039	0.0001099	0.0002679		0.552	0.99	0.002959	0.01836	0.8718939
rs12138480	1	220318644	A	G	0.406	1.00	0.163	0.039	0.000036	0.0001426		0.429	0.94	-0.00435	0.01893	0.8180382
rs1890759	1	220321247	A	T	0.455	1.00	0.147	0.039	0.0001449	0.0003792		0.461	1.00	-0.00411	0.01835	0.8227604
rs10863685	1	220322546	A	G	0.545	1.00	-0.147	0.039	0.000145	0.0003794		0.539	1.00	0.004103	0.01835	0.8228899
rs1591912	1	220322763	A	G	0.545	1.00	-0.147	0.039	0.0001451	0.0003797		0.539	1.00	0.004099	0.01834	0.8230473
rs10495184	1	220326300	A	C	0.427	0.96	0.165	0.04	0.0000328	0.0001245		0.442	0.93	-0.00376	0.01899	0.8428447
rs12561821	1	220327852	C	T	0.417	0.99	0.158	0.039	0.0000616	0.0002439		0.433	0.96	-0.00255	0.01875	0.8918771
rs11118942	1	220332176	A	G	0.444	0.99	0.141	0.039	0.0003007	0.0004294		0.448	0.98	-0.00294	0.01856	0.8741726
rs11118943	1	220333229	A	G	0.444	1.00	0.14	0.039	0.0003234	0.0004531		0.448	0.98	-0.00302	0.01858	0.8708154
rs12125086	1	220335479	C	T	0.557	1.00	-0.14	0.039	0.0003367	0.0004701		0.553	0.97	0.003202	0.01861	0.8632989
rs6702063	1	220337347	C	T	0.557	1.00	-0.139	0.039	0.0003419	0.0004781		0.553	0.97	0.003267	0.01862	0.8605958
rs6678715	1	220343906	C	G	0.407	0.97	0.153	0.04	0.0001296	0.0002504		0.419	0.92	-0.00258	0.01918	0.8929865
rs10863689	1	220349108	C	T	0.624	0.99	-0.147	0.04	0.0002591	0.0003792		0.597	0.97	0.014162	0.01887	0.4526178
rs10779468	1	220349505	C	T	0.624	0.99	-0.147	0.04	0.0002603	0.0003804		0.597	0.97	0.014139	0.01887	0.4533451
rs10916034	1	225018938	C	T	0.361	1.00	0.14	0.041	0.0005963	0.0099177		0.328	1.00	0.000651	0.01965	0.9735532
rs853444	1	228749436	A	G	0.105	1.00	0.223	0.064	0.0004593	0.0014283		0.143	0.84	0.03455	0.02948	0.2409102
rs9431646	1	229340173	A	G	0.274	1.00	0.147	0.043	0.0007347	0.0008878		0.331	1.00	-0.01035	0.01935	0.592243
rs9431933	1	229340279	C	T	0.729	0.98	-0.146	0.044	0.0009082	0.001299		0.671	0.96	0.009758	0.01977	0.621244
rs9431934	1	229341083	C	T	0.763	0.99	-0.151	0.046	0.000906	0.0027337		0.714	0.98	0.010559	0.02041	0.604527
rs926007	1	231549629	A	G	0.614	0.99	0.134	0.04	0.0007315	0.0021575		0.593	0.99	-0.02305	0.0188	0.2199082
rs6678607	1	231550354	C	G	0.386	0.99	-0.134	0.04	0.0007669	0.00219		0.404	0.99	0.023941	0.01874	0.2011231
rs6703635	1	231550377	C	T	0.614	0.99	0.133	0.04	0.0007765	0.0021985		0.596	0.99	-0.02397	0.01874	0.2005506
rs10797448	1	231551310	C	T	0.615	1.00	0.131	0.039	0.0008876	0.0023204		0.596	1.00	-0.02399	0.01874	0.2001828
rs6424180	1	231555931	A	T	0.615	1.00	0.131	0.039	0.0009452	0.0024248		0.596	1.00	-0.02413	0.01872	0.1971853
rs7539016	1	231556122	A	G	0.615	1.00	0.131	0.039	0.0009487	0.002431		0.596	1.00	-0.02414	0.01872	0.1968368
rs7549137	1	231556152	A	G	0.385	1.00	-0.131	0.039	0.0009495	0.00244		0.404	1.00	0.024143	0.01872	0.1969014
rs10910133	1	231556616	A	G	0.385	1.00	-0.131	0.039	0.0009502	0.0024451		0.404	1.00	0.024153	0.01873	0.1968073
rs10797449	1	231556761	A	G	0.385	1.00	-0.131	0.039	0.0009505	0.0024497		0.404	1.00	0.024156	0.01873	0.1967911
rs2049183	1	231557031	A	T	0.385	1.00	-0.131	0.039	0.0009531	0.0024665		0.404	0.99	0.024176	0.01874	0.1967454
rs2689178	1	236996976	C	T	0.645	1.00	-0.105	0.04	0.009403	0.000348		0.667	0.86	-0.01644	0.02111	0.4356657
rs16838569	1	237842712	A	G	0.050	0.99	-0.31	0.09	0.0005996	0.0010829		0.043	0.98	-0.02656	0.04304	0.5368368
rs10925928	1	237843762	G	T	0.051	1.00	-0.325	0.089	0.0002765	0.0004758		0.044	0.97	-0.01869	0.04256	0.6602624
rs726169	1	237860900	C	T	0.322	1.00	-0.123	0.041	0.003025	0.0005352		0.357	1.00	0.049843	0.01883	0.0080848
rs12090480	1	237880576	A	G	0.308	1.00	-0.125	0.042	0.002761	0.0004043		0.339	1.00	0.050725	0.01912	0.0079414
rs685550	1	237991031	A	G	0.777	0.79	0.176	0.053	0.0008267	0.0016869		0.799	0.70	0.004295	0.02608	0.869102
rs12410855	1	239778757	C	T	0.656	0.90	-0.11	0.043	0.01041	0.0001401		0.637	0.41	0.03263	0.02941	0.2667701
rs10927277	1	242833949	C	G	0.449	0.63	-0.181	0.049	0.0002134	0.0005326		0.485	0.42	0.02368	0.02819	0.4004542
rs12131373	1	244164637	C	T	0.952	0.98	0.321	0.092	0.0004735	0.0035455		0.933	0.25	0.115254	0.0741	0.1195528
rs2683992	2	590575	G	T	0.160	1.00	-0.176	0.053	0.000875	0.0004561	TMEM18					
rs2867105	2	591905	G	T	0.840	1.00	0.176	0.053	0.000875	0.0004551	TMEM18					
rs2867131	2	600603	C	T	0.847	0.99	0.21	0.054	0.0001077	0.0000961	TMEM18					
rs2947411	2	604168	A	G	0.156	1.00	-0.201	0.054	0.0001905	0.0001243	TMEM18					
rs2860323	2	604210	A	G	0.156	1.00	-0.201	0.054	0.0001907	0.0001244	TMEM18					

					GOYA QC		GOYA Overweight/control			GOYA BMI continuous	known	IARC QC		IARC results		
MARKER	chr	position	A1	A2	FREQ1	Rsqr	Beta	SE	p	p	gene name	freq1	Rsqr	in_beta	in_SE	in_p
rs7567570	2	605140	C	T	0.845	1.00	0.201	0.054	0.0001911	0.0001246	TMEM18					
rs6548237	2	611461	A	C	0.157	0.99	-0.195	0.054	0.0002965	0.0001928	TMEM18					
rs939584	2	611558	C	T	0.206	0.95	-0.198	0.05	0.0000718	0.0001756	TMEM18					
rs1320331	2	612161	C	G	0.845	1.00	0.191	0.054	0.000382	0.0002048	TMEM18					
rs1320330	2	612225	G	T	0.843	0.99	0.193	0.054	0.0003286	0.0002075	TMEM18					
rs939583	2	612531	C	T	0.155	1.00	-0.19	0.054	0.0004062	0.0002144	TMEM18					
rs939582	2	612723	A	G	0.155	1.00	-0.19	0.054	0.0004127	0.000217	TMEM18					
rs2867125	2	612827	C	T	0.845	1.00	0.19	0.054	0.0004189	0.0002194	TMEM18					
rs11127483	2	613691	A	G	0.837	0.96	0.198	0.054	0.0002258	0.0001223	TMEM18					
rs11127484	2	613798	C	T	0.845	1.00	0.192	0.054	0.0003696	0.0001957	TMEM18					
rs6719518	2	613935	C	T	0.155	1.00	-0.193	0.054	0.0003502	0.0001866	TMEM18					
rs6728726	2	613976	C	T	0.859	0.99	0.185	0.056	0.001035	0.0006563	TMEM18					
rs6711012	2	614034	C	G	0.845	1.00	0.193	0.054	0.0003425	0.000183	TMEM18					
rs2867123	2	614524	C	G	0.845	0.99	0.194	0.054	0.0003348	0.0001794	TMEM18					
rs2867122	2	614581	A	C	0.155	0.99	-0.194	0.054	0.0003298	0.0001771	TMEM18					
rs2903492	2	614678	A	G	0.845	0.99	0.194	0.054	0.0003273	0.0001759	TMEM18					
rs7576624	2	615029	C	T	0.154	0.99	-0.209	0.054	0.0001208	0.0000759	TMEM18					
rs7576635	2	615057	C	T	0.154	0.99	-0.209	0.054	0.0001185	0.0000748	TMEM18					
rs6744646	2	618504	A	G	0.154	0.99	-0.209	0.054	0.0001178	0.0000744	TMEM18					
rs6744653	2	618524	A	G	0.154	0.99	-0.209	0.054	0.000117	0.000074	TMEM18					
rs12463617	2	619244	A	C	0.125	0.94	-0.219	0.061	0.0002954	0.0001825	TMEM18					
rs6743060	2	619510	A	C	0.847	0.99	0.209	0.054	0.0001148	0.0000729	TMEM18					
rs6752470	2	619694	A	T	0.847	0.99	0.209	0.054	0.0001135	0.0000722	TMEM18					
rs12995480	2	619881	C	T	0.847	0.99	0.209	0.054	0.000112	0.0000715	TMEM18					
rs6732471	2	619914	A	G	0.847	0.99	0.209	0.054	0.0001114	0.0000712	TMEM18					
rs13007080	2	620024	A	C	0.154	0.99	-0.21	0.054	0.00011	0.0000705	TMEM18					
rs13007086	2	620034	A	T	0.154	0.99	-0.21	0.054	0.0001087	0.0000698	TMEM18					
rs6725549	2	620323	A	C	0.847	1.00	0.211	0.054	0.0000959	0.0000632	TMEM18					
rs6731348	2	620339	A	C	0.153	1.00	-0.211	0.054	0.0000947	0.0000626	TMEM18					
rs6731688	2	620662	A	C	0.153	1.00	-0.211	0.054	0.0000942	0.0000623	TMEM18					
rs5017303	2	620995	G	T	0.847	1.00	0.212	0.054	0.0000931	0.0000618	TMEM18					
rs5017300	2	621099	C	G	0.153	1.00	-0.212	0.054	0.0000926	0.0000615	TMEM18					
rs7585056	2	621528	A	G	0.153	1.00	-0.212	0.054	0.0000921	0.0000612	TMEM18					
rs11127485	2	622028	C	T	0.153	1.00	-0.212	0.054	0.000091	0.0000607	TMEM18					
rs12623218	2	622146	A	T	0.847	1.00	0.212	0.054	0.0000895	0.0000599	TMEM18					
rs12992154	2	622300	G	T	0.153	1.00	-0.212	0.054	0.000089	0.0000597	TMEM18					
rs13021737	2	622348	A	G	0.153	1.00	-0.212	0.054	0.0000879	0.0000591	TMEM18					
rs13012571	2	622550	C	T	0.153	1.00	-0.212	0.054	0.000087	0.0000586	TMEM18					
rs6548238	2	624905	C	T	0.858	0.96	0.199	0.057	0.0004475	0.0003424	TMEM18					
rs6734363	2	625200	A	G	0.847	1.00	0.212	0.054	0.000085	0.0000576	TMEM18					
rs6755502	2	625721	C	T	0.847	1.00	0.213	0.054	0.0000845	0.0000573	TMEM18					
rs13388043	2	627597	C	T	0.153	1.00	-0.213	0.054	0.0000836	0.0000569	TMEM18					
rs13393304	2	627830	A	G	0.153	1.00	-0.213	0.054	0.0000827	0.0000563	TMEM18					
rs4854344	2	628144	G	T	0.153	1.00	-0.213	0.054	0.0000822	0.0000561	TMEM18					
rs7601028	2	632499	C	G	0.139	0.99	-0.207	0.056	0.0002494	0.000211	TMEM18					
rs7604609	2	633303	C	G	0.153	1.00	-0.213	0.054	0.0000815	0.0000558	TMEM18					
rs7561317	2	634953	A	G	0.153	1.00	-0.213	0.054	0.0000804	0.0000552	TMEM18					
rs11127491	2	636145	C	T	0.847	1.00	0.213	0.054	0.0000801	0.0000552	TMEM18					
rs10189761	2	636364	A	T	0.845	1.00	0.206	0.054	0.0001269	0.0001081	TMEM18					
rs10190052	2	636674	C	T	0.847	1.00	0.214	0.054	0.000078	0.0000552	TMEM18					
rs10173167	2	636767	A	G	0.847	1.00	0.214	0.054	0.0000774	0.0000552	TMEM18					
rs7571957	2	636803	C	T	0.847	1.00	0.214	0.054	0.0000768	0.0000553	TMEM18					
rs10193244	2	637580	C	T	0.847	1.00	0.214	0.054	0.0000765	0.0000554	TMEM18					
rs4854348	2	637760	A	G	0.153	1.00	-0.214	0.054	0.0000762	0.0000553	TMEM18					
rs4854349	2	637861	C	T	0.847	1.00	0.214	0.054	0.0000759	0.0000554	TMEM18					
rs7570198	2	638198	C	T	0.153	1.00	-0.214	0.054	0.0000752	0.0000554	TMEM18					
rs4423631	2	638758	C	T	0.847	1.00	0.214	0.054	0.0000743	0.0000554	TMEM18					
rs4452188	2	638810	A	G	0.847	1.00	0.214	0.054	0.0000737	0.0000554	TMEM18					
rs1320338	2	639347	G	T	0.153	1.00	-0.214	0.054	0.000077	0.0000584	TMEM18					
rs1320337	2	639867	A	T	0.847	1.00	0.214	0.054	0.0000777	0.000059	TMEM18					
rs1320336	2	640012	A	G	0.153	1.00	-0.214	0.054	0.0000784	0.0000596	TMEM18					
rs2867108	2	640143	C	T	0.153	1.00	-0.213	0.054	0.00008	0.000061	TMEM18					
rs13386517	2	640479	C	G	0.153	1.00	-0.213	0.054	0.0000808	0.0000616	TMEM18					
rs13401686	2	640519	A	G	0.847	1.00	0.213	0.054	0.0000816	0.0000623	TMEM18					
rs13386627	2	640560	C	G	0.153	1.00	-0.213	0.054	0.0000823	0.0000629	TMEM18					
rs4613321	2	640647	A	G	0.847	1.00	0.213	0.054	0.0000839	0.0000642	TMEM18					
rs13386964	2	640828	A	G	0.153	1.00	-0.213	0.054	0.0000856	0.0000656	TMEM18					
rs2867109	2	641030	G	T	0.153	1.00	-0.213	0.054	0.0000874	0.000067	TMEM18					
rs2867110	2	641105	C	G	0.153	1.00	-0.213	0.054	0.0000882	0.0000677	TMEM18					
rs2867112	2	641349	G	T	0.153	0.99	-0.212	0.054	0.0000966	0.0000747	TMEM18					
rs2867113	2	641365	A	G	0.153	0.99	-0.211	0.054	0.0000986	0.0000764	TMEM18					
rs12714414	2	641407	C	T	0.153	0.99	-0.211	0.054	0.0001006	0.000078	TMEM18					
rs12714415	2	641430	C	T	0.153	0.99	-0.211	0.054	0.0001026	0.0000797	TMEM18					
rs6719980	2	641507	C	T	0.153	0.99	-0.211	0.054	0.0001048	0.0000816	TMEM18					
rs7608050	2	642247	A	G	0.153	0.99	-0.21	0.054	0.0001082	0.0000844	TMEM18					
rs7574359	2	642542	C	T	0.153	0.99	-0.21	0.054	0.0001117	0.0000873	TMEM18					
rs13415094	2	643093	C	T	0.153	0.99	-0.209	0.054	0.0001164	0.0000912	TMEM18					
rs13396935	2	643195	A	G	0.153	0.99	-0.209	0.054	0.000119	0.0000933	TMEM18					
rs13397165	2	643354	A	G	0.153	0.99	-0.209	0.054	0.0001215	0.0000953	TMEM18					
rs10188334	2	643874	C	T	0.847	0.99	0.208	0.054	0.0001272	0.0001001	TMEM18					
rs4645032	2	1799067	C	T	0.550	0.99	-0.141	0.039	0.0002972	0.0036401		0.591	1.00	0.026967	0.0185	0.1447351
rs11692670	2	2669986	A	G	0.027	0.40	0.524	0.193	0.006622	0.0006774		0.025	0.17	-0.08338	0.13048	0.5224795

					GOYA QC		GOYA Overweight/control			GOYA BMI continuous	known	IARC QC		IARC results		
MARKER	chr	position	A1	A2	FREQ1	Rsqr	Beta	SE	p	p	gene name	freq1	Rsqr	in_beta	in_SE	in_p
rs13017659	2	6172764	A	C	0.078	0.58	0.202	0.095	0.0325	0.0008514		0.066	0.61	-0.05876	0.04829	0.223312
rs4669318	2	8688015	A	G	0.921	0.98	0.252	0.073	0.0005704	0.0019913		0.936	0.79	0.044298	0.04222	0.2936875
rs4668743	2	11957145	C	T	0.917	0.96	-0.241	0.072	0.0007816	0.0019633		0.935	0.89	-0.01881	0.03916	0.6306843
rs11680328	2	17901288	A	T	0.885	1.00	0.245	0.061	0.00005	0.0003337		0.880	0.89	0.002986	0.0296	0.9195823
rs6758546	2	17902293	A	G	0.101	1.00	-0.283	0.064	0.00000924	0.0000875		0.104	0.97	-0.01667	0.03029	0.5817924
rs1002159	2	23392137	A	C	0.785	1.00	-0.184	0.047	0.0000943	0.0002594		0.783	0.73	-0.00887	0.02524	0.7250684
rs1002158	2	23392348	G	T	0.737	1.00	-0.198	0.044	0.00000683	0.0001194		0.708	0.71	0.006257	0.02355	0.7903284
rs2033768	2	23396083	C	T	0.769	1.00	-0.157	0.046	0.0006195	0.0014585		0.761	1.00	-0.00723	0.02102	0.7307851
rs2723120	2	23402161	C	T	0.770	1.00	-0.157	0.046	0.000627	0.0016814		0.761	0.99	-0.00581	0.02106	0.782339
rs6720873	2	23405275	A	G	0.228	0.98	0.162	0.047	0.0005155	0.0014946		0.234	0.96	0.006529	0.02147	0.7608361
rs1122899	2	23408655	C	T	0.772	0.96	-0.162	0.047	0.0006072	0.0014656		0.763	0.94	-0.01388	0.02147	0.5175024
rs2723124	2	23411519	A	C	0.773	0.96	-0.162	0.047	0.0006275	0.0015386		0.762	0.94	-0.01313	0.02146	0.5404589
rs2577711	2	23425354	C	T	0.227	1.00	0.16	0.047	0.000634	0.0011552		0.226	0.98	0.003281	0.02141	0.878092
rs2723140	2	23426262	A	G	0.227	1.00	0.16	0.047	0.0006357	0.0011466		0.226	0.98	0.003239	0.0214	0.879589
rs6749822	2	23435063	C	T	0.774	1.00	-0.155	0.047	0.0009492	0.0015841		0.767	0.97	-0.00236	0.02138	0.9121204
rs6720565	2	23435124	A	G	0.226	1.00	0.155	0.047	0.0009462	0.0015652		0.234	0.97	0.002219	0.02132	0.9170428
rs2577705	2	23442944	A	G	0.205	0.98	0.199	0.049	0.000043	0.000129		0.198	0.93	0.0019	0.02294	0.9339113
rs2577703	2	23445724	C	T	0.196	1.00	0.197	0.049	0.000058	0.0001316		0.193	0.96	0.003031	0.0228	0.8941658
rs2577756	2	23455061	A	G	0.203	1.00	0.205	0.048	0.0000234	0.0000567		0.200	1.00	0.002977	0.02211	0.8927921
rs1030907	2	23468532	C	T	0.741	1.00	-0.187	0.044	0.0000259	0.000058		0.740	1.00	0.014748	0.02031	0.467443
rs920271	2	23473751	C	T	0.767	0.98	-0.166	0.046	0.0003205	0.0003473		0.772	0.98	0.003263	0.0214	0.8786945
rs2577748	2	23477767	C	T	0.764	0.99	-0.162	0.046	0.0004145	0.0004036		0.769	0.98	0.00405	0.02125	0.8486949
rs2577744	2	23481816	A	T	0.763	0.98	-0.161	0.046	0.0004585	0.0004373		0.769	0.98	0.004252	0.02127	0.8413893
rs12621454	2	23487074	A	G	0.297	0.96	0.151	0.043	0.0004833	0.0007155		0.288	0.94	-0.02641	0.02041	0.1952996
rs2723111	2	23487805	A	G	0.239	0.97	0.155	0.046	0.0007697	0.000697		0.230	0.95	-0.00592	0.02167	0.7844746
rs6544822	2	23489454	C	T	0.277	0.97	0.156	0.044	0.0003975	0.0008946		0.263	0.85	-0.02536	0.02222	0.2534074
rs17734019	2	24543529	A	C	0.951	0.61	-0.337	0.115	0.003547	0.0008852		0.958	0.60	-0.02503	0.0601	0.6768407
rs2584920	2	24561900	A	T	0.243	1.00	-0.132	0.046	0.003697	0.0009782		0.276	0.99	-0.01489	0.02091	0.4760363
rs17734264	2	24573214	C	T	0.757	1.00	0.132	0.046	0.003819	0.0009967		0.724	1.00	0.014856	0.0209	0.476815
rs17734306	2	24575942	A	G	0.757	1.00	0.132	0.046	0.003833	0.000999		0.724	1.00	0.014844	0.0209	0.4771012
rs17799872	2	24898461	A	G	0.067	1.00	0.261	0.078	0.000821	0.0010001		0.062	1.00	0.038429	0.03901	0.3242309
rs13390269	2	24922297	A	G	0.072	0.95	0.263	0.077	0.0006808	0.0011716		0.066	0.94	0.035599	0.0388	0.3585202
rs17046666	2	24923855	C	G	0.072	0.95	0.263	0.077	0.0006923	0.0011879		0.066	0.94	0.035561	0.03884	0.3595435
rs6749646	2	25047502	A	T	0.787	0.98	-0.162	0.048	0.0006932	0.0000224		0.853	0.97	-0.01249	0.02576	0.627525
rs13388020	2	25049770	C	G	0.213	0.98	0.163	0.048	0.0006834	0.0000216		0.147	0.97	0.012527	0.02575	0.6263835
rs17744531	2	25156350	A	G	0.272	0.96	0.141	0.044	0.001442	0.0002199		0.215	0.96	0.000174	0.02196	0.9936779
rs17801121	2	25157791	C	T	0.728	0.97	-0.14	0.044	0.001492	0.0002299		0.785	0.96	-0.00011	0.02195	0.9960618
rs491147	2	25166505	C	G	0.272	0.97	0.139	0.044	0.001582	0.0002484		0.215	0.97	-0.00018	0.02192	0.9934666
rs7575363	2	25196418	A	G	0.717	1.00	-0.147	0.043	0.0005659	0.0000523		0.779	1.00	0.000145	0.02136	0.9945861
rs3731631	2	25212926	A	G	0.284	1.00	0.126	0.043	0.003136	0.0006711		0.218	1.00	0.005008	0.02161	0.8165539
rs1866146	2	25234077	A	G	0.666	1.00	0.137	0.041	0.0009334	0.0010196		0.579	1.00	0.012035	0.01848	0.5144576
rs934778	2	25242728	A	G	0.693	1.00	-0.139	0.042	0.0009054	0.0003629		0.766	0.99	0.011704	0.02127	0.5818982
rs6547102	2	26627371	C	T	0.030	0.65	-0.479	0.144	0.0008788	0.001271		0.033	0.22	0.019036	0.10577	0.8570575
rs6714085	2	29037771	A	G	0.133	0.98	0.211	0.058	0.0002389	0.0038757		0.151	0.99	0.037329	0.02582	0.1479869
rs4666156	2	29050217	C	G	0.135	1.00	0.22	0.057	0.0001044	0.0027333		0.151	1.00	0.036478	0.02561	0.1540046
rs4666157	2	29050289	C	T	0.135	1.00	0.22	0.057	0.0001031	0.002719		0.151	1.00	0.03612	0.0256	0.1578753
rs4456659	2	29524338	A	G	0.475	1.00	0.137	0.038	0.0003553	0.000137		0.434	0.99	-0.01063	0.01829	0.5609784
rs4497832	2	29524381	C	T	0.526	1.00	-0.135	0.038	0.0004429	0.0001808		0.566	1.00	0.010552	0.01827	0.5631456
rs4341891	2	29524608	G	T	0.527	1.00	-0.133	0.038	0.000564	0.0002442		0.566	1.00	0.010525	0.01825	0.5638294
rs4589706	2	29524631	C	T	0.527	1.00	-0.133	0.038	0.0005481	0.0002377		0.566	1.00	0.010509	0.01825	0.5644327
rs10779969	2	29525305	A	C	0.473	1.00	0.134	0.038	0.0005143	0.0002224		0.434	1.00	-0.01048	0.01825	0.5653342
rs4366858	2	29526051	C	T	0.473	1.00	0.136	0.038	0.0004177	0.0001767		0.434	1.00	-0.01043	0.01825	0.5672764
rs4299298	2	29526260	C	T	0.527	1.00	-0.136	0.038	0.0004124	0.0001767		0.566	1.00	0.010489	0.01825	0.5651749
rs6547930	2	29526444	C	T	0.473	1.00	0.136	0.038	0.0004097	0.0001767		0.434	1.00	-0.01054	0.01825	0.5631812
rs11127225	2	29527111	C	T	0.527	1.00	-0.136	0.038	0.000407	0.0001767		0.566	1.00	0.010604	0.01826	0.56103
rs4233736	2	29530469	C	T	0.527	1.00	-0.137	0.039	0.0003761	0.0001779		0.566	1.00	0.011032	0.01827	0.5457209
rs4233737	2	29530540	C	G	0.500	0.99	0.127	0.039	0.0009697	0.0004252		0.477	0.98	-0.00881	0.0183	0.6299209
rs4602182	2	29531026	C	T	0.404	0.99	-0.135	0.04	0.000684	0.000271		0.412	0.98	-0.00925	0.01838	0.6143736
rs4665461	2	29561490	C	T	0.501	0.98	0.135	0.039	0.0005132	0.0002385		0.498	0.99	-0.02969	0.01821	0.102703
rs6708397	2	29586648	G	T	0.448	1.00	0.114	0.039	0.003386	0.0002929		0.450	1.00	0.004607	0.01799	0.7977201
rs6705644	2	29586878	A	G	0.553	1.00	-0.114	0.039	0.003349	0.0002863		0.550	1.00	-0.00457	0.01799	0.7992021
rs12990552	2	29586909	C	T	0.704	1.00	0.127	0.043	0.002921	0.000644		0.714	0.99	0.00401	0.01992	0.8402732
rs4665463	2	29587105	C	T	0.448	1.00	0.115	0.039	0.003243	0.0002676		0.450	1.00	0.004538	0.01799	0.8006496
rs4665464	2	29587202	C	T	0.448	1.00	0.115	0.039	0.00321	0.0002617		0.450	1.00	0.004522	0.01799	0.8013514
rs6719507	2	29587305	A	G	0.437	1.00	-0.137	0.039	0.0004952	0.0000343		0.432	1.00	0.000437	0.01806	0.9806908
rs12618086	2	29592506	G	T	0.724	1.00	0.118	0.044	0.00671	0.00095		0.736	0.99	0.001099	0.02058	0.957391
rs4567889	2	29592681	C	T	0.449	1.00	0.116	0.039	0.002872	0.0001903		0.451	1.00	0.004094	0.018	0.8199012
rs6750887	2	29592714	C	T	0.449	1.00	0.116	0.039	0.002869	0.0001905		0.451	1.00	0.004075	0.018	0.8207685
rs4468777	2	29597085	G	T	0.567	0.98	-0.141	0.039	0.000363	0.0004221		0.573	0.95	0.005401	0.01862	0.7715765
rs7578465	2	29597237	C	T	0.546	0.98	-0.137	0.039	0.0004867	0.0010733		0.523	0.94	0.000274	0.01868	0.9882811
rs7562040	2	29597326	C	G	0.451	0.98	0.141	0.039	0.0003347	0.0006731		0.475	0.94	-0.0007	0.01865	0.9699544
rs10168427	2	29598487	C	T	0.435	0.98	0.141	0.04	0.000375	0.0004668		0.427	0.95	-0.00538	0.01863	0.7725107
rs6547949	2	29645345	A	G	0.545	1.00	-0.129	0.039	0.0009614	0.0045747		0.551	1.00	0.004323	0.01822	0.8122795
rs955214	2	30299430	A	G	0.058	0.78	0.334	0.095	0.0004172	0.0049573		0.066	0.78	-0.00574	0.04327	0.8944095
rs932559	2	31406406	A	C	0.466	1.00	-0.107	0.039	0.006074	0.0008193		0.457	1.00	0.004145	0.01837	0.8213792
rs10490361	2	31407053	C	G	0.464	0.98	-0.111	0.039	0.004674	0.0005153		0.456	0.98	8.73E-05	0.01855	0.9962419
rs169596	2	31419628	A	G	0.490	0.78	-0.144	0.044	0.001086	0.0001871		0.483	0.71	-0.00598	0.02195	0.7852319
rs11692723	2	33320559	C	G	0.233	0.86	0.145	0.05	0.003502	0.0002299		0.189	0.71	0.015716	0.02746	0.5667794
rs2716727	2	39807250	C	T	0.426	1.00	-0.138	0.039	0.0004504	0.0041764		0.430	0.99	-0.00491	0.0184	0.7894456
rs4953676	2	42975230	A	C	0.085	1.00	0.248	0.069	0.0002995	0.0010471		0.099	0.97	-0.00619	0.02973	0.8350326
rs4953682	2	42982252	C	G	0.913	0.99	-0.237	0.068	0.0005068	0.0012906		0.888	0.92	0.010246	0.0291	0.7245487
rs4952947	2	42992437	A	G	0.098	1.00	0.25	0.064	0.0001031	0.0003701		0.120	1.00	-0.00689	0.02769	0.8034604

					GOYA QC		GOYA Overweight/control			GOYA BMI continuous	known	IARC QC		IARC results		
MARKER	chr	position	A1	A2	FREQ1	Rsqr	Beta	SE	p	p	gene name	freq1	Rsqr	in_beta	in_SE	in_p
rs4953246	2	45833614	C	T	0.315	0.96	0.145	0.043	0.000651	0.0003328		0.355	0.91	0.025881	0.01994	0.1940551
rs867286	2	45835534	A	G	0.322	0.99	0.147	0.042	0.0004385	0.0003183		0.356	0.94	0.026421	0.01971	0.1797379
rs884401	2	45836112	C	T	0.353	0.98	0.136	0.041	0.0009921	0.0011371		0.390	0.93	0.022174	0.01937	0.2520509
rs884399	2	45836429	G	T	0.678	1.00	-0.147	0.042	0.0004217	0.0003359		0.648	0.99	-0.0271	0.01923	0.1583095
rs4953476	2	47286969	A	T	0.017	0.93	-0.615	0.161	0.0001398	0.0022228		0.023	0.71	0.018241	0.07245	0.8010421
rs3136326	2	47877906	C	T	0.136	0.96	0.167	0.058	0.003876	0.0008858		0.156	0.97	0.023189	0.0257	0.3664912
rs3136329	2	47878380	C	T	0.432	0.98	0.143	0.04	0.0003331	0.0008553		0.473	0.98	0.014748	0.01868	0.4294409
rs1800937	2	47879268	C	T	0.871	0.97	-0.187	0.059	0.001503	0.0002663		0.851	0.98	-0.01644	0.02605	0.527632
rs3136337	2	47881969	C	T	0.877	1.00	-0.209	0.059	0.0004357	0.0001007		0.863	1.00	-0.00594	0.02688	0.8250687
rs17395820	2	47930683	C	G	0.124	0.97	0.192	0.06	0.001339	0.0004859		0.143	0.99	0.016425	0.02672	0.5384662
rs11693533	2	48063490	A	G	0.140	1.00	0.203	0.056	0.0002803	0.000078		0.134	1.00	0.005794	0.02697	0.8297786
rs11695693	2	48076813	A	G	0.862	1.00	-0.205	0.056	0.0002586	0.0001086		0.868	0.99	-0.00638	0.02715	0.8139657
rs11692660	2	48098088	A	C	0.134	1.00	0.192	0.057	0.0006932	0.0001657		0.132	1.00	0.004149	0.02713	0.8783426
rs6745620	2	48099246	C	T	0.866	1.00	-0.192	0.057	0.0007069	0.0001669		0.868	1.00	-0.00415	0.02713	0.8784439
rs17037087	2	48100059	G	T	0.866	1.00	-0.192	0.057	0.0007175	0.0001681		0.868	1.00	-0.00414	0.02713	0.8785342
rs11682328	2	48101605	C	T	0.134	1.00	0.192	0.057	0.0007364	0.0001706		0.132	1.00	0.004136	0.02713	0.878756
rs11681243	2	48104660	G	T	0.866	1.00	-0.192	0.057	0.0007298	0.0001689		0.868	1.00	-0.00414	0.02714	0.8786834
rs11687232	2	48104997	C	T	0.134	1.00	0.192	0.057	0.0007259	0.0001679		0.132	1.00	0.00414	0.02714	0.8786638
rs11677590	2	48128572	G	T	0.134	1.00	0.194	0.057	0.0006697	0.0001532		0.132	0.99	0.004058	0.02718	0.8812112
rs11693696	2	48138651	C	T	0.866	0.99	-0.199	0.057	0.0004974	0.0001106		0.868	0.99	-0.00406	0.02718	0.8812152
rs11680404	2	48149609	A	G	0.134	0.98	0.203	0.057	0.0004092	0.00009		0.133	0.99	0.003954	0.02723	0.8844582
rs11689818	2	48152495	G	T	0.133	0.98	0.204	0.057	0.0003757	0.0000839		0.133	0.98	0.003997	0.02727	0.8833833
rs7561696	2	48157355	A	G	0.867	0.98	-0.204	0.057	0.0003727	0.0000833		0.867	0.98	-0.00399	0.02727	0.8835554
rs11687989	2	48160151	A	G	0.133	0.98	0.205	0.057	0.0003708	0.0000829		0.133	0.98	0.004005	0.02728	0.8831691
rs7583719	2	48191474	A	G	0.138	0.93	0.211	0.058	0.0003035	0.0001361		0.146	0.89	-0.00856	0.0276	0.7563657
rs17396793	2	48218522	A	G	0.132	0.98	0.209	0.058	0.0003113	0.0001021		0.134	0.96	0.004645	0.02769	0.8666756
rs7581510	2	48233082	A	G	0.869	0.99	-0.208	0.058	0.0003247	0.0001418		0.866	0.96	-0.00718	0.02768	0.7950502
rs6733558	2	48248990	A	G	0.869	0.99	-0.21	0.058	0.0002768	0.0001253		0.866	0.96	-0.00852	0.02766	0.7578633
rs11676381	2	48267428	A	G	0.131	0.99	0.21	0.058	0.0002761	0.0001246		0.135	0.95	0.00853	0.02776	0.7584103
rs11691493	2	48267657	C	T	0.869	0.99	-0.21	0.058	0.000276	0.0001246		0.865	0.95	-0.00854	0.02776	0.7582039
rs11683689	2	48277385	A	C	0.131	0.99	0.211	0.058	0.0002639	0.0001196		0.135	0.95	0.00851	0.02775	0.7589347
rs13431982	2	48286425	C	T	0.132	0.99	0.211	0.058	0.0002503	0.0001158		0.135	0.95	0.008486	0.02774	0.7595155
rs10495950	2	48288268	G	T	0.132	0.99	0.211	0.058	0.0002513	0.0001161		0.135	0.95	0.008473	0.02774	0.759816
rs17325048	2	48290070	A	C	0.868	0.99	-0.21	0.058	0.0002519	0.0001163		0.865	0.95	-0.00847	0.02774	0.7598286
rs11681876	2	48298832	A	G	0.868	0.99	-0.208	0.058	0.0002985	0.0001352		0.865	0.96	-0.00846	0.02771	0.7600181
rs11680162	2	48310478	C	T	0.868	0.99	-0.206	0.058	0.0003343	0.0001535		0.863	0.95	-0.00714	0.02767	0.7963372
rs17397094	2	48321212	C	G	0.872	0.97	-0.196	0.059	0.00086	0.0003038		0.865	0.96	-0.00546	0.02767	0.843408
rs952468	2	48323636	G	T	0.128	0.97	0.196	0.059	0.0008783	0.0003096		0.135	0.96	0.005391	0.02767	0.8454102
rs6747738	2	48324873	C	T	0.873	0.97	-0.196	0.059	0.0008838	0.0003109		0.865	0.96	-0.00539	0.02767	0.8455209
rs17325209	2	48332714	A	G	0.128	0.97	0.197	0.059	0.0008678	0.0003016		0.135	0.96	0.005234	0.02771	0.8500369
rs11695923	2	48340711	C	T	0.873	0.97	-0.198	0.059	0.0008282	0.0002792		0.865	0.96	-0.00511	0.02773	0.8535716
rs11682838	2	48494659	A	G	0.867	0.99	-0.195	0.057	0.00068	0.0002054		0.859	0.99	-0.02035	0.02709	0.4521138
rs2301267	2	48837895	C	T	0.498	1.00	-0.128	0.039	0.0009328	0.0002504		0.537	1.00	-0.00606	0.01823	0.7392953
rs12478799	2	48842028	A	G	0.507	0.98	0.127	0.039	0.001147	0.0002817		0.468	0.95	0.007631	0.01864	0.6819849
rs4334540	2	48849821	C	G	0.481	0.96	-0.127	0.039	0.001291	0.0005357		0.516	0.87	-0.01516	0.01937	0.4334959
rs4245821	2	48850113	A	G	0.481	0.96	-0.127	0.039	0.001291	0.0005327		0.515	0.86	-0.01535	0.01951	0.4311131
rs4245822	2	48850254	C	T	0.494	0.95	-0.127	0.04	0.001315	0.0003191		0.538	0.83	-0.01037	0.01986	0.6012778
rs13426172	2	48853286	A	G	0.890	0.73	0.273	0.072	0.0001531	0.0016935		0.876	0.52	0.064817	0.03672	0.0773596
rs1452773	2	50131453	G	T	0.141	0.95	-0.169	0.057	0.003142	0.0009181		0.125	0.94	0.066823	0.0284	0.0185469
rs17268047	2	53600941	A	T	0.461	1.00	0.127	0.039	0.0009991	0.0018426		0.438	0.99	-0.02633	0.01836	0.1512379
rs2357359	2	53610060	A	G	0.450	1.00	0.137	0.039	0.0003964	0.0010042		0.434	1.00	-0.0297	0.01828	0.1038853
rs759719	2	53682548	C	G	0.158	0.77	-0.203	0.061	0.0007885	0.0024142		0.151	0.63	-0.00821	0.03152	0.7943763
rs12623902	2	55039733	C	T	0.727	0.99	0.149	0.044	0.0007044	0.006751		0.645	0.97	-0.00561	0.0197	0.7755678
rs17346629	2	55039898	A	C	0.273	0.99	-0.149	0.044	0.0007039	0.0067549		0.355	0.97	0.005613	0.0197	0.7755657
rs6741304	2	55041699	A	G	0.723	1.00	0.151	0.043	0.0004955	0.0054627		0.634	1.00	-0.00567	0.01936	0.7696568
rs2920848	2	55199271	A	T	0.957	1.00	0.288	0.096	0.002618	0.0006256		0.938	1.00	0.071607	0.04004	0.0734782
rs2920849	2	55199433	A	G	0.957	1.00	0.288	0.096	0.002619	0.0006255		0.938	1.00	0.071529	0.04003	0.0737428
rs2920852	2	55200156	A	G	0.044	1.00	-0.288	0.096	0.002614	0.0006207		0.062	1.00	-0.07138	0.04001	0.0742179
rs2920853	2	55200851	G	T	0.957	1.00	0.288	0.096	0.002627	0.000621		0.938	1.00	0.071268	0.04001	0.0746762
rs1444261	2	55207970	C	T	0.045	1.00	-0.28	0.094	0.002994	0.0008087		0.062	1.00	-0.06735	0.03994	0.0914917
rs2968803	2	55215174	C	T	0.955	1.00	0.281	0.094	0.002885	0.000758		0.938	1.00	0.066201	0.03987	0.0965734
rs4530400	2	55218009	A	G	0.955	1.00	0.282	0.094	0.002873	0.0007533		0.938	1.00	0.065367	0.03991	0.1011701
rs4268958	2	55218826	C	T	0.045	1.00	-0.282	0.094	0.002862	0.0007486		0.062	1.00	-0.06533	0.03991	0.1013596
rs17047369	2	56051905	A	G	0.867	1.00	-0.205	0.057	0.0003539	0.0005267		0.909	0.67	-0.00593	0.03843	0.877219
rs2868992	2	56086059	G	T	0.229	1.00	0.144	0.046	0.001757	0.0005373		0.202	0.93	0.051324	0.02342	0.0282927
rs7587002	2	56092504	C	T	0.771	1.00	-0.144	0.046	0.001789	0.0005443		0.798	0.94	-0.05132	0.02341	0.0282468
rs13399945	2	56093401	A	G	0.792	1.00	-0.14	0.048	0.003553	0.0005852		0.815	1.00	-0.05383	0.02353	0.0221002
rs6741589	2	56093499	C	T	0.792	1.00	-0.14	0.048	0.003557	0.0005852		0.815	1.00	-0.05383	0.02353	0.0220869
rs7597360	2	56094801	C	T	0.771	1.00	-0.144	0.046	0.00184	0.0005622		0.798	0.94	-0.05134	0.0234	0.0281194
rs10210936	2	56095128	A	C	0.229	1.00	0.143	0.046	0.001996	0.0006243		0.202	0.94	0.051345	0.0234	0.0281002
rs4553860	2	56097600	A	G	0.772	1.00	-0.14	0.046	0.002378	0.0007815		0.798	0.94	-0.05135	0.0234	0.0280798
rs12713313	2	56097861	A	T	0.228	1.00	0.14	0.046	0.002355	0.0007623		0.202	0.94	0.051345	0.02339	0.0280741
rs12713314	2	56098247	C	T	0.772	1.00	-0.14	0.046	0.002345	0.0007548		0.798	0.94	-0.05135	0.02339	0.0280245
rs11693755	2	56099058	A	G	0.207	1.00	0.137	0.048	0.004365	0.0006978		0.184	1.00	0.053889	0.02352	0.0218601
rs1347093	2	56099844	G	T	0.793	1.00	-0.137	0.048	0.00436	0.0006939		0.816	1.00	-0.05389	0.02352	0.02184
rs2903976	2	56100252	A	G	0.227	1.00	0.142	0.046	0.002094	0.0005843		0.202	0.94	0.051343	0.02339	0.0280417
rs11125750	2	58431475	C	T	0.543	1.00	0.137	0.039	0.0003996	0.0016577		0.547	1.00	-0.0347	0.01817	0.0560662
rs6761469	2	58434522	A	G	0.457	1.00	-0.137	0.039	0.0003999	0.0016545		0.453	1.00	0.034678	0.01817	0.0561511
rs4399710	2	58471266	A	G	0.507	1.00	0.137	0.038	0.000357	0.0058151		0.531	0.97	-0.03677	0.01841	0.0456604
rs2192663	2	58487057	C	T	0.494	1.00	-0.135	0.038	0.0004238	0.0062379		0.469	0.96	0.036724	0.01844	0.0462276
rs7340335	2	58492582	A	G	0.494	1.00	-0.135	0.038	0.0004341	0.0063234		0.469	0.96	0.036717	0.01844	0.0463051
rs10179027	2	58505942	A	T	0.494	1.00	-0.129	0.039	0.0007764	0.0087988		0.469	0.96	0.036696	0.01845	0.0465387

					GOYA QC		GOYA Overweight/control			GOYA BMI continuous	known	IARC QC		IARC results		
MARKER	chr	position	A1	A2	FREQ1	Rsqr	Beta	SE	p	p	gene name	freq1	Rsqr	in_beta	in_SE	in_p
rs17049763	2	58918383	C	G	0.903	0.99	-0.227	0.067	0.0006604	0.0003039		0.882	0.98	0.007663	0.02954	0.7951672
rs7593324	2	58919312	A	G	0.096	1.00	0.227	0.067	0.0006601	0.0003202		0.117	1.00	-0.0077	0.02948	0.7936369
rs17049767	2	58924706	C	T	0.906	1.00	-0.219	0.067	0.00105	0.0005207		0.889	0.99	0.009343	0.03012	0.756214
rs17049773	2	58927053	C	G	0.906	1.00	-0.219	0.067	0.001066	0.0005258		0.889	0.99	0.009449	0.03012	0.7535006
rs17049775	2	58927838	A	G	0.906	1.00	-0.219	0.067	0.001068	0.0005268		0.889	0.99	0.009586	0.03012	0.7500786
rs4140881	2	58945569	C	G	0.904	0.99	-0.219	0.067	0.001028	0.0005806		0.881	0.97	0.011938	0.02927	0.6831415
rs12618463	2	58949304	A	G	0.083	0.88	0.239	0.075	0.001559	0.0009816		0.109	0.84	-0.01323	0.03243	0.6830111
rs1019264	2	59866306	A	G	0.384	1.00	-0.143	0.04	0.0003229	0.011785		0.434	0.87	0.00678	0.01985	0.732451
rs11687648	2	66229568	C	T	0.287	0.99	-0.15	0.044	0.0006203	0.0003446		0.258	0.97	-0.02162	0.02074	0.2968282
rs1906900	2	66233293	A	G	0.287	1.00	-0.15	0.044	0.0005963	0.0003294		0.258	0.98	-0.02168	0.02059	0.2917843
rs10198265	2	66234330	C	T	0.713	1.00	0.15	0.044	0.0005929	0.0003272		0.742	0.99	0.021706	0.02057	0.2909501
rs6712489	2	66235892	A	G	0.713	1.00	0.15	0.044	0.0005921	0.0003267		0.742	0.99	0.021723	0.02055	0.2900154
rs7573350	2	66237753	C	T	0.287	1.00	-0.15	0.044	0.0005923	0.0003265		0.258	0.99	-0.02171	0.02054	0.290011
rs13424573	2	66238937	A	T	0.713	1.00	0.15	0.044	0.0005924	0.0003263		0.742	0.99	0.021708	0.02053	0.2900348
rs6748113	2	66240611	A	C	0.713	1.00	0.15	0.044	0.0005924	0.0003262		0.742	0.99	0.021714	0.02052	0.2896581
rs7600569	2	66241643	A	G	0.715	1.00	0.15	0.044	0.0005856	0.0003002		0.742	0.99	0.021716	0.0205	0.2891339
rs2861009	2	66241794	C	T	0.285	1.00	-0.151	0.044	0.0005838	0.0002994		0.258	0.99	-0.02169	0.02051	0.2898948
rs6761361	2	66244394	A	C	0.715	1.00	0.151	0.044	0.0005803	0.0002977		0.742	0.99	0.021508	0.02053	0.2944945
rs12613969	2	66244550	A	G	0.715	1.00	0.15	0.044	0.0006017	0.0003016		0.734	0.95	0.020832	0.02077	0.3155866
rs1320859	2	66244946	A	C	0.285	1.00	-0.15	0.044	0.0006003	0.000301		0.266	0.95	-0.02083	0.02078	0.3156154
rs10200852	2	66245336	A	G	0.285	1.00	-0.15	0.044	0.0005984	0.0003002		0.266	0.95	-0.02068	0.02079	0.3194996
rs10177124	2	66245345	A	G	0.715	1.00	0.15	0.044	0.0005961	0.0002992		0.734	0.95	0.020674	0.02079	0.3196859
rs2954974	2	66245399	C	G	0.536	0.97	-0.151	0.039	0.0001249	0.0004348		0.480	0.80	0.002524	0.01995	0.8992281
rs1320861	2	66245766	C	T	0.285	1.00	-0.15	0.044	0.0005972	0.0002988		0.266	0.95	-0.02059	0.0208	0.321758
rs1320862	2	66245829	A	G	0.285	1.00	-0.15	0.044	0.0005977	0.0002986		0.266	0.95	-0.02056	0.0208	0.322564
rs1997383	2	66247154	C	T	0.285	1.00	-0.15	0.044	0.0005855	0.0002956		0.266	0.95	-0.02053	0.02081	0.3235364
rs1385931	2	67526971	C	T	0.506	0.99	0.109	0.039	0.004934	0.0000617		0.505	1.00	-0.00292	0.01814	0.8719834
rs600524	2	67527284	A	G	0.389	0.99	-0.144	0.04	0.0003091	0.0000541		0.394	1.00	0.007074	0.01863	0.7038468
rs600579	2	67527327	A	T	0.611	0.99	0.143	0.04	0.000312	0.0000541		0.606	1.00	-0.00701	0.01863	0.706584
rs650129	2	67527434	C	G	0.540	0.97	0.134	0.039	0.0006297	0.0001693		0.511	0.90	-0.01468	0.01903	0.4399948
rs650638	2	67527546	A	G	0.359	0.99	-0.117	0.04	0.00368	0.0004542		0.366	1.00	0.003873	0.01896	0.8379904
rs658602	2	67540293	A	G	0.492	1.00	-0.109	0.039	0.004547	0.000066		0.494	1.00	0.002552	0.01817	0.8881841
rs581955	2	67543727	A	G	0.352	0.94	-0.122	0.042	0.00352	0.0004612		0.362	0.97	0.002814	0.01928	0.8838578
rs624414	2	67546309	A	C	0.347	0.89	-0.123	0.043	0.004325	0.0005595		0.361	0.97	0.002611	0.01936	0.8926177
rs6710260	2	69183622	A	G	0.855	0.99	-0.189	0.055	0.0006642	0.0031341		0.835	0.71	-0.01656	0.02883	0.5653489
rs4241349	2	69183788	A	G	0.759	1.00	-0.15	0.045	0.0009913	0.0084224		0.760	0.82	-0.02146	0.02337	0.3579821
rs7564112	2	69196653	C	T	0.888	0.50	-0.332	0.088	0.0001525	0.0003252		0.884	0.48	-0.00303	0.04059	0.9403896
rs12232994	2	76809501	C	T	0.250	0.96	0.179	0.045	0.0000744	0.0005621		0.230	0.79	-0.00912	0.02404	0.7043413
rs10169851	2	76810002	C	G	0.216	0.98	0.156	0.047	0.000927	0.0027858		0.207	0.83	-0.01474	0.02442	0.5456599
rs7569282	2	76820160	A	G	0.247	1.00	0.181	0.045	0.0000481	0.0003965		0.217	0.84	-0.00602	0.02382	0.8004584
rs4853267	2	76821152	C	G	0.254	1.00	0.18	0.044	0.0000467	0.0003843		0.229	0.82	-0.00576	0.02359	0.8068476
rs2056207	2	76822781	A	G	0.746	1.00	-0.18	0.044	0.0000474	0.0004366		0.769	0.81	-0.0012	0.02367	0.9596775
rs2056208	2	76822886	C	T	0.746	1.00	-0.179	0.044	0.0000482	0.0004395		0.769	0.81	-0.00124	0.02367	0.9581731
rs13396159	2	76830085	A	G	0.759	1.00	-0.157	0.045	0.0004941	0.0013077		0.745	0.98	0.000248	0.02076	0.9904447
rs13403042	2	76831776	A	T	0.759	1.00	-0.151	0.045	0.0007811	0.0019093		0.745	0.98	0.000152	0.02073	0.994156
rs4853272	2	76867232	A	C	0.310	1.00	0.151	0.042	0.0003119	0.0003303		0.340	0.94	0.002784	0.01982	0.8881825
rs6743728	2	76868522	C	T	0.373	0.98	0.142	0.039	0.0002964	0.0003823		0.396	0.90	-0.01696	0.01966	0.3879954
rs13001302	2	77142992	G	T	0.054	0.83	0.31	0.095	0.001136	0.0004717		0.094	0.79	-0.01156	0.03501	0.740995
rs2861857	2	79759614	A	G	0.457	1.00	-0.111	0.039	0.004772	0.0004956		0.430	1.00	0.01821	0.01819	0.3163277
rs17762507	2	85247495	C	G	0.051	0.99	0.229	0.088	0.009479	0.0008489		0.051	0.92	0.012636	0.04311	0.7692581
rs2366958	2	86023500	C	T	0.533	1.00	0.134	0.039	0.000616	0.0002029		0.554	0.99	-0.01362	0.01841	0.4590947
rs9309628	2	86025839	C	T	0.533	1.00	0.135	0.039	0.0005573	0.000175		0.554	1.00	-0.01355	0.01835	0.4599588
rs735738	2	86030833	C	T	0.533	1.00	0.135	0.039	0.0005592	0.000175		0.554	1.00	-0.01359	0.01835	0.458408
rs12185570	2	94821556	C	T	0.087	0.94	-0.236	0.072	0.0009535	0.0002287		0.091	0.67	0.018981	0.0404	0.6381863
rs17842087	2	94843502	C	T	0.087	0.94	-0.236	0.071	0.0009561	0.0002309		0.091	0.67	0.019053	0.04031	0.6361408
rs10445976	2	95040165	C	T	0.896	1.00	0.223	0.063	0.0004215	0.0001273		0.887	1.00	0.007453	0.0291	0.7976948
rs1316873	2	95062373	C	G	0.896	0.99	0.228	0.064	0.0003435	0.0000888		0.887	0.99	0.006664	0.02925	0.8196167
rs17119064	2	95085186	C	T	0.896	0.99	0.229	0.064	0.0003394	0.0000869		0.887	0.98	0.005494	0.02944	0.851828
rs10184772	2	95142092	A	G	0.130	1.00	-0.185	0.058	0.001331	0.0001624		0.133	1.00	-0.00127	0.02748	0.9631465
rs13385838	2	95231974	A	G	0.103	1.00	-0.223	0.064	0.0004805	0.0001384		0.116	1.00	0.001741	0.02918	0.9523886
rs13387294	2	95252161	G	T	0.103	1.00	-0.218	0.064	0.0006077	0.000179		0.116	1.00	0.001507	0.02917	0.9587556
rs2174353	2	98360067	A	G	0.432	0.97	-0.163	0.04	0.0000467	0.0001324		0.384	0.54	0.00236	0.02545	0.9260727
rs12469806	2	100558382	A	G	0.355	0.90	0.155	0.042	0.0002559	0.0003653		0.344	0.62	0.00157	0.02456	0.9489743
rs855044	2	108182705	C	T	0.304	0.95	0.154	0.043	0.0003392	0.0006224		0.304	0.95	-0.02999	0.02007	0.1348119
rs6741327	2	115020103	A	T	0.496	0.31	0.23	0.069	0.000829	0.0075602		0.492	0.22	-0.04919	0.03816	0.1970601
rs17043351	2	115159564	C	T	0.050	1.00	-0.299	0.091	0.0009759	0.0005317		0.036	0.98	0.001078	0.04534	0.9810226
rs1435879	2	115209357	A	G	0.875	1.00	0.23	0.059	0.0001016	0.0000819		0.881	1.00	-0.00991	0.02856	0.7282668
rs11122867	2	122231684	G	T	0.557	0.89	-0.155	0.041	0.0001628	0.0090781		0.594	0.70	0.024357	0.02215	0.271057
rs6748146	2	124019119	A	G	0.012	0.59	-0.952	0.255	0.0001829	0.0006026		0.009	0.45	0.121235	0.16792	0.4699349
rs11899372	2	127490350	G	T	0.930	1.00	-0.219	0.075	0.003361	0.000744		0.926	0.52	0.028269	0.04616	0.5399556
rs11903472	2	127490368	A	C	0.071	0.98	0.216	0.075	0.003997	0.0009126		0.072	0.53	-0.02809	0.04632	0.5438886
rs2466388	2	130276517	A	T	0.511	0.94	0.096	0.04	0.01605	0.0004979		0.587	0.83	0.007252	0.02028	0.7204363
rs2466384	2	130286676	A	G	0.528	1.00	0.092	0.039	0.01709	0.0007569		0.592	0.85	0.007663	0.02005	0.7021334
rs2464776	2	130288300	A	G	0.528	1.00	0.092	0.039	0.01727	0.0007642		0.592	0.85	0.00768	0.02006	0.7015369
rs2707643	2	130289777	A	G	0.528	1.00	0.091	0.038	0.01806	0.0007972		0.592	0.85	0.007703	0.02006	0.7007489
rs2466383	2	130291594	A	G	0.528	1.00	0.091	0.038	0.0182	0.0008011		0.592	0.85	0.007775	0.02007	0.6982326
rs2707644	2	130292516	A	G	0.528	1.00	0.091	0.038	0.0182	0.0007997		0.592	0.84	0.007786	0.02007	0.6978425
rs10179686	2	134931040	C	T	0.983	0.86	-0.547	0.166	0.0009426	0.00103		0.963	0.76	-0.0046	0.05172	0.9290457
rs7561441	2	136913621	C	G	0.263	0.99	-0.118	0.044	0.00765	0.0003105		0.425	0.97	-0.01812	0.01874	0.3331897
rs4954419	2	136918471	C	G	0.737	0.99	0.119	0.044	0.007139	0.0002844		0.574	0.97	0.017852	0.01871	0.339642
rs4954616	2	136920488	A	G	0.736	1.00	0.124	0.044	0.004783	0.000165		0.574	0.98	0.017807	0.01871	0.3407058
rs12691889	2	136921965	A	C	0.271	0.97	-0.124	0.044	0.004938	0.0002708		0.459	0.94	-0.00988	0.0189	0.6007016

					GOYA QC		GOYA Overweight/control			GOYA BMI continuous	known	IARC QC		IARC results		
MARKER	chr	position	A1	A2	FREQ1	Rsqr	Beta	SE	p	p	gene name	freq1	Rsqr	in_beta	in_SE	in_p
rs6760376	2	136936764	C	T	0.774	1.00	0.119	0.046	0.01033	0.0007635		0.635	0.99	0.001044	0.01888	0.9558775
rs2163270	2	136936855	C	G	0.734	1.00	0.131	0.044	0.002954	0.0000864		0.573	1.00	0.017901	0.01851	0.332984
rs47150	2	136937213	A	G	0.226	1.00	-0.119	0.046	0.01006	0.0007452		0.365	0.99	-0.00108	0.01888	0.9542069
rs2033172	2	136956774	A	G	0.266	1.00	-0.129	0.044	0.003357	0.0001037		0.427	1.00	-0.01802	0.01848	0.3292542
rs643821	2	136957924	A	G	0.774	1.00	0.117	0.046	0.0114	0.0008853		0.635	1.00	0.001148	0.01885	0.9514062
rs483230	2	136968905	C	G	0.226	1.00	-0.118	0.046	0.0113	0.0008713		0.365	1.00	-0.00106	0.01885	0.9550415
rs581054	2	136970675	C	T	0.278	0.96	-0.126	0.044	0.004273	0.0002222		0.438	0.96	-0.01783	0.01874	0.3411529
rs10200731	2	136975594	A	G	0.267	1.00	-0.134	0.044	0.002415	0.0000589		0.423	0.99	-0.0175	0.01862	0.3469253
rs649801	2	136976367	A	C	0.773	1.00	0.121	0.046	0.009107	0.0005622		0.638	0.99	0.000192	0.01894	0.9919238
rs12465885	2	136985782	C	T	0.029	0.90	-0.408	0.122	0.0008529	0.0001246		0.043	0.81	0.033118	0.04922	0.5006813
rs470705	2	136996851	C	T	0.733	1.00	0.134	0.044	0.002305	0.0000569		0.577	0.99	0.017261	0.0186	0.3528782
rs694510	2	136997331	G	T	0.773	1.00	0.121	0.046	0.008997	0.0005583		0.638	0.99	-0.00016	0.01894	0.9933128
rs562370	2	136999489	A	C	0.143	1.00	-0.147	0.055	0.007771	0.0007109		0.207	1.00	0.001452	0.02244	0.9483815
rs13035996	2	137002246	A	G	0.267	1.00	-0.134	0.044	0.002277	0.0000577		0.423	1.00	-0.01706	0.01856	0.3576469
rs876338	2	137005617	C	T	0.253	1.00	-0.148	0.045	0.0009459	0.0000205		0.413	0.99	-0.01977	0.01869	0.2898237
rs7355665	2	137010147	C	T	0.267	1.00	-0.134	0.044	0.002294	0.0000589		0.422	1.00	-0.01703	0.01857	0.3585387
rs10205768	2	137016561	G	T	0.718	0.99	0.135	0.044	0.001933	0.000077		0.525	0.98	0.013867	0.01856	0.4545424
rs6715684	2	137020581	C	G	0.733	1.00	0.134	0.044	0.002247	0.0000592		0.578	0.99	0.017005	0.01858	0.3596096
rs705388	2	137023640	A	G	0.911	0.99	0.187	0.069	0.006289	0.000961		0.863	1.00	-0.03157	0.02623	0.2285199
rs4954625	2	137026495	G	T	0.264	0.99	-0.143	0.044	0.001229	0.0000385		0.409	0.96	-0.01414	0.01895	0.4553821
rs12612204	2	137030710	A	G	0.272	1.00	-0.116	0.044	0.007808	0.0004571		0.403	0.86	-0.01032	0.01993	0.6043
rs12691893	2	137032209	G	T	0.766	0.99	0.127	0.046	0.005797	0.0008068		0.643	0.94	0.015857	0.01961	0.4184718
rs13005339	2	137037900	A	G	0.277	1.00	-0.115	0.044	0.008299	0.0003774		0.411	0.88	-0.01104	0.01965	0.5739948
rs12994851	2	137040456	C	T	0.235	1.00	-0.128	0.046	0.005258	0.0007152		0.356	0.94	-0.01551	0.01962	0.4289879
rs11686763	2	137042465	G	T	0.750	0.97	0.137	0.046	0.002618	0.0006344		0.599	0.87	0.008523	0.02005	0.6705042
rs10193021	2	137058182	C	T	0.282	1.00	-0.108	0.043	0.01241	0.0005007		0.406	0.81	-0.00416	0.02046	0.8386849
rs12691894	2	137058414	A	G	0.718	1.00	0.108	0.043	0.01233	0.0004964		0.594	0.81	0.004141	0.02047	0.8395146
rs533344	2	137065457	A	T	0.767	0.91	0.106	0.048	0.02741	0.0005614		0.690	0.77	-0.01241	0.02222	0.5760921
rs13392503	2	137147604	G	T	0.771	0.99	0.125	0.046	0.006715	0.0003483		0.654	0.98	0.004458	0.01913	0.81562
rs10199153	2	137213276	C	G	0.179	1.00	-0.148	0.051	0.003548	0.0001889		0.277	0.98	0.003701	0.02047	0.8564066
rs7564005	2	137229168	G	T	0.835	1.00	0.14	0.052	0.007509	0.0004417		0.752	0.98	0.00788	0.02089	0.7057177
rs1427591	2	137230888	G	T	0.182	1.00	-0.131	0.05	0.009413	0.0005795		0.284	0.99	-0.01386	0.02004	0.4887051
rs6750400	2	137484018	C	T	0.323	0.99	0.154	0.042	0.0002279	0.000358		0.373	0.56	0.047223	0.02503	0.0590428
rs1991735	2	137620484	A	G	0.643	1.00	0.14	0.04	0.0005605	0.0002932		0.687	1.00	0.022539	0.0197	0.2520798
rs17346940	2	137728279	A	G	0.830	0.98	-0.174	0.052	0.0008451	0.0001935		0.855	0.95	0.037797	0.02643	0.1523082
rs10490734	2	137728653	A	G	0.793	1.00	-0.149	0.049	0.002144	0.0003193		0.803	1.00	0.012753	0.02294	0.5779325
rs11680942	2	137738826	C	T	0.833	1.00	-0.156	0.052	0.002655	0.0002377		0.855	0.97	0.034233	0.02594	0.1865141
rs12614095	2	137762081	C	T	0.839	1.00	-0.15	0.053	0.004588	0.0005567		0.860	0.95	0.041538	0.02675	0.120191
rs11688662	2	137764810	A	G	0.829	1.00	-0.178	0.052	0.0005836	0.0001475		0.854	0.94	0.044934	0.02657	0.090609
rs17738873	2	137790265	A	G	0.804	0.73	-0.202	0.058	0.000474	0.0003571		0.810	0.60	0.032867	0.02947	0.2642948
rs354694	2	143641987	A	G	0.664	0.96	-0.145	0.042	0.0005868	0.0019041		0.606	0.99	-0.00597	0.01878	0.7502847
rs723529	2	147993393	A	G	0.917	0.99	-0.298	0.07	0.0000224	0.000748		0.917	0.98	0.04748	0.03414	0.1640044
rs10930910	2	152638864	C	T	0.511	1.00	0.139	0.039	0.0003833	0.0015689		0.478	0.99	-0.04827	0.01856	0.0092454
rs12472524	2	152680942	A	G	0.773	0.98	-0.157	0.047	0.0007897	0.0444249		0.810	0.97	0.043649	0.02328	0.0606131
rs10200655	2	152683206	A	T	0.314	0.98	0.142	0.042	0.0007281	0.0464513		0.289	0.94	-0.02699	0.02052	0.1880754
rs10497095	2	152686234	A	G	0.227	0.98	0.157	0.047	0.0007634	0.0436445		0.191	0.97	-0.04353	0.02326	0.0611303
rs3820702	2	152689099	A	T	0.314	0.99	0.142	0.042	0.0006972	0.0453142		0.289	0.94	-0.02704	0.02051	0.1870375
rs4664531	2	152694287	C	T	0.285	1.00	0.154	0.043	0.0003314	0.0221233		0.278	0.95	-0.028	0.02061	0.1740092
rs12623355	2	152694839	C	T	0.774	1.00	-0.16	0.046	0.000556	0.0366648		0.809	0.99	0.04238	0.02311	0.0665115
rs12613984	2	152695792	A	C	0.774	1.00	-0.159	0.046	0.0005913	0.0378581		0.809	0.99	0.042365	0.02311	0.0665859
rs3768651	2	152698967	G	T	0.227	1.00	0.159	0.046	0.0006302	0.039122		0.191	1.00	-0.04207	0.02307	0.0680033
rs3768650	2	152699178	A	G	0.686	1.00	-0.143	0.042	0.0006312	0.0428475		0.711	0.95	0.025617	0.02037	0.2080936
rs12612031	2	152700483	A	G	0.309	1.00	0.141	0.042	0.0007291	0.0462842		0.283	0.95	-0.02933	0.02051	0.1524123
rs12621378	2	152703423	C	T	0.774	1.00	-0.159	0.046	0.0006427	0.0371175		0.809	1.00	0.041917	0.02304	0.0687193
rs12612557	2	152703507	G	T	0.226	1.00	0.159	0.046	0.0006421	0.0370927		0.191	1.00	-0.04188	0.02304	0.0689341
rs11893841	2	152706798	A	G	0.226	1.00	0.16	0.046	0.000591	0.0356179		0.191	1.00	-0.04175	0.02304	0.0698131
rs12617139	2	152708583	A	T	0.501	1.00	0.129	0.039	0.0008923	0.0054141		0.449	0.96	-0.01434	0.01859	0.4400607
rs3768649	2	152711350	C	G	0.241	0.99	0.152	0.046	0.0009048	0.0704293		0.206	0.94	-0.0352	0.02305	0.1264455
rs3768648	2	152711658	C	T	0.228	0.99	0.162	0.047	0.0005107	0.0332456		0.202	0.95	-0.03435	0.02312	0.137084
rs4664535	2	152712499	A	G	0.241	0.99	0.152	0.046	0.0008993	0.0702504		0.206	0.94	-0.03489	0.02304	0.1296809
rs4664536	2	152712631	A	C	0.501	1.00	0.129	0.039	0.0008657	0.0053445		0.449	0.96	-0.01386	0.01859	0.4554918
rs10184862	2	152714090	A	G	0.691	1.00	-0.143	0.042	0.0006549	0.0444478		0.710	0.95	0.022434	0.0204	0.2709779
rs13003423	2	152714698	C	G	0.242	0.99	0.152	0.046	0.000887	0.0698676		0.207	0.94	-0.03446	0.02303	0.1343132
rs6650779	2	152715280	A	G	0.758	0.99	-0.152	0.046	0.0008852	0.0698023		0.793	0.94	0.03438	0.02303	0.1351683
rs6650771	2	152717377	A	G	0.499	1.00	-0.13	0.039	0.0008421	0.0052846		0.550	0.96	0.013572	0.01858	0.4647925
rs10930958	2	152717655	C	T	0.499	1.00	-0.13	0.039	0.0008391	0.0052768		0.550	0.96	0.013439	0.01858	0.4691022
rs10930959	2	152717715	A	G	0.758	0.99	-0.152	0.046	0.0008809	0.0696678		0.793	0.94	0.034066	0.02302	0.1386076
rs6731625	2	152728858	A	G	0.514	0.98	-0.13	0.039	0.0008816	0.0041821		0.555	0.95	0.013942	0.01871	0.4559149
rs4664084	2	152730317	C	T	0.758	0.99	-0.152	0.046	0.0008773	0.0695205		0.793	0.94	0.033823	0.02301	0.1413179
rs6738680	2	152732359	A	G	0.501	1.00	0.129	0.039	0.0008622	0.0053818		0.450	0.96	-0.01311	0.01857	0.4798884
rs4611639	2	152733276	C	G	0.238	0.96	0.169	0.047	0.0002828	0.0126568		0.219	0.92	-0.02063	0.0228	0.3651796
rs12328733	2	152737313	A	G	0.229	0.99	0.162	0.047	0.000492	0.0324922		0.210	0.95	-0.02868	0.02287	0.2095142
rs10497097	2	152739115	A	G	0.229	0.99	0.162	0.047	0.0004932	0.0325222		0.210	0.95	-0.02851	0.02286	0.2119906
rs2345639	2	152739359	C	T	0.500	1.00	-0.129	0.039	0.0009148	0.0056174		0.548	0.97	0.010602	0.01851	0.5664362
rs4368329	2	152740839	A	G	0.242	0.99	0.152	0.046	0.0008823	0.0693557		0.214	0.94	-0.02864	0.02277	0.2081697
rs6740224	2	152741036	A	C	0.688	1.00	-0.143	0.042	0.0006187	0.0434675		0.699	0.96	0.013847	0.02007	0.489843
rs10803946	2	152741763	A	C	0.231	0.97	0.163	0.047	0.0004934	0.030918		0.219	0.99	-0.01858	0.02218	0.4019687
rs4664089	2	152753282	A	G	0.763	0.99	-0.162	0.046	0.0004376	0.0286932		0.797	0.94	0.012452	0.02335	0.5935068
rs11679305	2	152811160	C	T	0.234	1.00	0.157	0.046	0.0006574	0.0315402		0.221	1.00	-0.01691	0.02198	0.4412067
rs12997179	2	152856035	A	G	0.234	0.97	0.165	0.047	0.0004388	0.0127794		0.217	0.98	-0.01495	0.02215	0.499457
rs2345814	2	152858443	A	T	0.707	0.98	-0.152	0.043	0.0004276	0.0108218		0.710	0.98	0.006449	0.01992	0.7458913
rs2881225	2	152858973	C	T	0.708	1.00	-0.148	0.043	0.0005676	0.0129024		0.710	0.98	0.006399	0.01992	0.7477906

					GOYA QC		GOYA Overweight/control			GOYA BMI continuous	known	IARC QC		IARC results		
MARKER	chr	position	A1	A2	FREQ1	Rsqr	Beta	SE	p	p	gene name	freq1	Rsqr	in_beta	in_SE	in_p
rs4308089	2	152861405	A	G	0.709	1.00	-0.147	0.043	0.0006001	0.0133545		0.710	0.98	0.006274	0.01991	0.7524502
rs4664560	2	152861979	C	T	0.231	0.99	0.156	0.047	0.0007909	0.0179885		0.214	0.99	-0.01283	0.02206	0.5605111
rs7568759	2	152862858	A	G	0.308	1.00	0.14	0.042	0.0008714	0.0282792		0.297	1.00	-0.0009	0.01964	0.9632258
rs11674860	2	152862926	G	T	0.231	0.99	0.156	0.047	0.0007929	0.0180011		0.213	0.99	-0.01332	0.02207	0.5457446
rs12693313	2	152865289	A	G	0.291	1.00	0.147	0.043	0.0006145	0.0135555		0.287	0.99	-0.00556	0.01983	0.7791628
rs5005667	2	152871081	C	T	0.231	0.99	0.157	0.047	0.0007322	0.0169303		0.213	0.99	-0.01338	0.02207	0.5438826
rs2345876	2	152872760	A	G	0.291	1.00	0.147	0.043	0.0005638	0.0127195		0.287	0.99	-0.00568	0.01983	0.7744483
rs2345877	2	152873036	C	T	0.770	0.99	-0.156	0.047	0.0008004	0.0179596		0.787	0.99	0.013453	0.02207	0.5418086
rs12693320	2	152874418	A	T	0.291	1.00	0.145	0.043	0.0007082	0.0149742		0.287	0.99	-0.00575	0.01983	0.7716942
rs4140692	2	155006928	A	G	0.062	0.98	0.251	0.081	0.002	0.0002067		0.035	0.86	-0.00758	0.04698	0.8716973
rs9967675	2	159346833	C	T	0.346	0.99	0.128	0.041	0.00188	0.0006492		0.328	1.00	-0.0214	0.01938	0.2690371
rs2356507	2	159347591	C	T	0.652	1.00	-0.127	0.041	0.002004	0.0006615		0.672	1.00	0.021394	0.01937	0.2691233
rs12464272	2	163013969	A	T	0.064	0.99	0.267	0.08	0.0008317	0.0165754		0.063	0.92	-0.03535	0.03845	0.3574564
rs6734436	2	163019448	C	T	0.064	1.00	0.268	0.08	0.0008124	0.0160007		0.063	0.92	-0.0353	0.03845	0.3581155
rs16846727	2	163025089	A	G	0.064	1.00	0.268	0.08	0.0008101	0.0159488		0.063	0.92	-0.03527	0.03845	0.3585376
rs16846732	2	163027377	A	T	0.064	0.98	0.273	0.081	0.0007075	0.0135176		0.063	0.92	-0.03526	0.03845	0.3586608
rs16846743	2	163029260	C	T	0.936	0.98	-0.274	0.081	0.0006882	0.0130517		0.937	0.92	0.035219	0.03845	0.3592567
rs6758303	2	163034049	C	G	0.063	0.97	0.28	0.082	0.0005971	0.0105431		0.063	0.92	-0.03322	0.03832	0.3855243
rs7594587	2	163038340	A	T	0.937	0.96	-0.282	0.082	0.0005561	0.0090549		0.937	0.92	0.033163	0.03831	0.3863158
rs6715546	2	163039641	G	T	0.063	0.96	0.282	0.082	0.000555	0.0090031		0.063	0.92	-0.03314	0.03831	0.3865358
rs985621	2	163047879	A	G	0.063	0.96	0.283	0.082	0.0005482	0.0088927		0.064	0.91	-0.03153	0.03845	0.4119048
rs1485978	2	163053530	A	G	0.063	0.96	0.288	0.082	0.0004801	0.0080083		0.064	0.91	-0.03102	0.0385	0.4199275
rs12052788	2	163054809	C	T	0.062	0.94	0.298	0.083	0.0003652	0.006436		0.064	0.87	-0.01724	0.0393	0.6606335
rs1352070	2	163066129	A	G	0.905	0.76	-0.267	0.076	0.0004709	0.0076935		0.893	0.69	0.005452	0.03503	0.8762368
rs901303	2	163124693	A	G	0.042	0.87	0.373	0.106	0.0004282	0.008581		0.056	0.86	0.014075	0.04359	0.7465804
rs10497348	2	169426547	A	G	0.031	0.98	0.373	0.114	0.001046	0.0002725		0.044	0.97	0.052355	0.04516	0.245919
rs10497345	2	169443047	C	G	0.032	1.00	0.358	0.113	0.00146	0.0004344		0.044	0.97	0.052813	0.045	0.2402153
rs7576155	2	170690437	C	G	0.538	0.83	0.157	0.043	0.0002248	0.000071		0.553	0.79	-0.02401	0.02032	0.2368525
rs6711421	2	170697022	C	T	0.538	0.83	0.157	0.042	0.000224	0.0000712		0.553	0.80	-0.02376	0.02015	0.2379328
rs1861288	2	171208133	C	T	0.215	0.84	-0.185	0.052	0.0003931	0.0040705		0.194	0.80	0.000354	0.02492	0.9886629
rs10803851	2	171208413	C	T	0.845	0.91	0.188	0.057	0.0009112	0.0058407		0.866	0.90	0.013308	0.02711	0.623204
rs4513261	2	174386179	G	T	0.651	1.00	-0.095	0.041	0.02019	0.0007617		0.645	0.92	-0.01019	0.01956	0.6018711
rs2121362	2	174574103	C	T	0.364	1.00	-0.146	0.04	0.0002906	0.0004691		0.381	1.00	0.000864	0.01871	0.9631315
rs2056109	2	180246687	A	G	0.961	1.00	0.337	0.101	0.0008503	0.0048872		0.947	0.99	0.027207	0.04035	0.4997817
rs7424112	2	180254478	G	T	0.047	0.86	-0.34	0.1	0.0006498	0.003574		0.062	0.87	-0.02487	0.04006	0.5343746
rs932116	2	180263264	C	G	0.961	1.00	0.341	0.101	0.0007382	0.004454		0.947	0.99	0.026588	0.0404	0.5101054
rs2222033	2	180264474	A	C	0.039	1.00	-0.341	0.101	0.0007329	0.0044275		0.053	0.99	-0.02654	0.04041	0.5109681
rs1196067	2	182737857	A	G	0.294	1.00	0.14	0.042	0.0009348	0.0211397		0.271	0.99	-0.00832	0.02072	0.6879991
rs4850486	2	193067489	G	T	0.859	0.99	-0.173	0.056	0.002131	0.0004454		0.794	0.98	-0.01092	0.02272	0.6305823
rs4850494	2	193068097	A	G	0.859	1.00	-0.173	0.056	0.00209	0.000435		0.794	0.99	-0.01096	0.02268	0.6287571
rs717622	2	193148890	C	T	0.032	0.90	0.425	0.118	0.000303	0.0000883		0.026	0.91	0.011251	0.06794	0.8683468
rs17442387	2	193191216	A	T	0.907	0.61	-0.301	0.085	0.0004378	0.0001323		0.932	0.49	0.076697	0.05542	0.166042
rs2197471	2	193505588	A	G	0.507	0.99	0.129	0.039	0.0008833	0.0014063		0.492	0.98	-0.02016	0.01841	0.2731019
rs2203204	2	193505648	A	G	0.493	0.99	-0.129	0.039	0.000882	0.001401		0.509	0.98	0.020139	0.01841	0.2735777
rs10203662	2	193507924	G	T	0.507	1.00	0.129	0.039	0.0008767	0.0013798		0.491	0.98	-0.02013	0.01841	0.2737058
rs7585105	2	193509734	C	T	0.507	1.00	0.129	0.039	0.0008759	0.0013767		0.491	0.98	-0.02013	0.01841	0.2739421
rs1596748	2	193511236	A	G	0.494	1.00	-0.129	0.039	0.0008737	0.0013674		0.509	0.98	0.020111	0.01841	0.2743253
rs1596749	2	193511446	A	G	0.494	1.00	-0.129	0.039	0.0008728	0.0013636		0.517	1.00	0.018872	0.01825	0.3005978
rs10931581	2	193511948	A	G	0.506	1.00	0.129	0.039	0.0008677	0.0013411		0.483	1.00	-0.01885	0.01824	0.3010177
rs765278	2	193514069	C	T	0.506	1.00	0.129	0.039	0.000868	0.0013401		0.483	1.00	-0.01884	0.01824	0.3012732
rs12613154	2	193514820	A	G	0.494	1.00	-0.129	0.039	0.0008743	0.0013488		0.517	1.00	0.018865	0.01824	0.300537
rs13006519	2	193515521	C	G	0.506	1.00	0.129	0.039	0.000898	0.0013817		0.483	1.00	-0.01889	0.01824	0.3000212
rs10931584	2	193516615	C	T	0.494	1.00	-0.129	0.039	0.0009081	0.0013959		0.517	1.00	0.018941	0.01824	0.2986595
rs10204100	2	193517438	C	G	0.494	1.00	-0.129	0.039	0.0009146	0.0014051		0.517	1.00	0.018957	0.01824	0.2982483
rs4850844	2	193518471	A	G	0.506	1.00	0.128	0.039	0.000922	0.0014147		0.483	1.00	-0.01899	0.01824	0.297437
rs13010260	2	193520901	A	G	0.506	1.00	0.128	0.039	0.0009431	0.0014441		0.483	1.00	-0.01908	0.01824	0.2951941
rs13011989	2	193521670	A	G	0.509	0.99	0.129	0.039	0.0009514	0.0014473		0.487	0.98	-0.01913	0.01835	0.2966352
rs986652	2	193523649	A	T	0.494	1.00	-0.128	0.039	0.0009581	0.0014649		0.517	1.00	0.019153	0.01824	0.2934031
rs12995239	2	193525931	A	G	0.494	1.00	-0.128	0.039	0.0009658	0.0014755		0.517	1.00	0.019193	0.01825	0.2924235
rs10174037	2	193529377	A	T	0.506	1.00	0.128	0.039	0.0009729	0.0014852		0.483	1.00	-0.01923	0.01825	0.2915044
rs1512755	2	193529500	C	T	0.506	1.00	0.128	0.039	0.0009915	0.0015105		0.483	1.00	-0.01931	0.01825	0.289671
rs10497737	2	193532430	A	G	0.506	1.00	0.128	0.039	0.0009995	0.0015215		0.483	1.00	-0.01937	0.01825	0.2879866
rs1430692	2	195572268	A	C	0.834	1.00	-0.204	0.052	0.0000907	0.0002419		0.863	1.00	-0.0284	0.02645	0.2825174
rs10174127	2	195575277	C	T	0.836	0.96	-0.2	0.053	0.0001856	0.0007286		0.863	0.93	-0.03225	0.02755	0.2412937
rs2068896	2	195577280	G	T	0.196	0.98	0.173	0.049	0.000462	0.0007962		0.177	0.95	0.018387	0.02438	0.4503094
rs1430678	2	195592266	C	T	0.833	0.98	-0.208	0.053	0.0000733	0.0002969		0.861	0.96	-0.03076	0.02661	0.2472951
rs1400979	2	199272588	A	G	0.250	0.99	-0.155	0.044	0.0004745	0.0014137		0.260	0.95	0.025074	0.02124	0.2373222
rs1400976	2	199275159	C	T	0.751	1.00	0.154	0.044	0.0005045	0.001465		0.740	0.95	-0.0252	0.02121	0.2344928
rs1400975	2	199275208	G	T	0.798	0.91	0.171	0.05	0.0005524	0.0004814		0.807	0.84	-0.02547	0.02492	0.3063654
rs1356494	2	199281141	A	G	0.249	1.00	-0.153	0.044	0.0005209	0.0014475		0.253	1.00	0.025947	0.02088	0.2135907
rs13020013	2	199283968	C	T	0.751	1.00	0.155	0.044	0.0004606	0.0012954		0.746	0.99	-0.02616	0.02092	0.2107743
rs6434983	2	199284390	C	T	0.751	1.00	0.155	0.044	0.0004526	0.001276		0.746	0.99	-0.02617	0.02092	0.2105515
rs1533540	2	199284782	C	T	0.747	0.99	0.146	0.044	0.0009027	0.0022303		0.746	0.99	-0.02618	0.02092	0.2104817
rs1533541	2	199285097	A	G	0.253	0.99	-0.146	0.044	0.0009065	0.0022382		0.255	0.99	0.026187	0.02092	0.2103799
rs1518099	2	199285370	A	G	0.747	0.99	0.146	0.044	0.0009178	0.0022601		0.745	0.99	-0.0262	0.02093	0.2102491
rs6740484	2	199286862	C	T	0.746	0.99	0.145	0.044	0.0009812	0.002386		0.745	0.99	-0.02624	0.02093	0.2096879
rs10439328	2	199289967	C	T	0.779	0.97	0.152	0.047	0.001109	0.0007843		0.790	0.93	-0.02492	0.023	0.2783783
rs1400984	2	199318114	A	G	0.297	0.89	0.148	0.045	0.0009557	0.0005632		0.272	0.84	-0.0038	0.02207	0.8630605
rs6705243	2	199329186	A	G	0.702	0.94	-0.146	0.044	0.0008187	0.0005054		0.729	0.89	0.002904	0.02138	0.8918541
rs1533534	2	199331843	C	T	0.702	0.94	-0.145	0.043	0.0008312	0.0005234		0.729	0.90	0.002797	0.02127	0.8953042
rs1518081	2	199342013	C	T	0.314	1.00	0.141	0.041	0.0006574	0.0004224		0.277	0.96	-0.00831	0.02043	0.6840937
rs1518079	2	199344305	C	T	0.298	0.99	0.138	0.042	0.001086	0.0005835		0.267	0.95	-0.00484	0.02084	0.8161705

					GOYA QC		GOYA Overweight/control			GOYA BMI continuous	known	IARC QC		IARC results		
MARKER	chr	position	A1	A2	FREQ1	Rsqr	Beta	SE	p	p	gene name	freq1	Rsqr	in_beta	in_SE	in_p
rs1356488	2	199346623	C	T	0.706	0.99	-0.141	0.043	0.0009378	0.0006107		0.733	0.90	0.004458	0.02146	0.8352787
rs12986948	2	199348177	A	G	0.706	0.99	-0.14	0.042	0.0009438	0.0006401		0.733	0.84	0.004015	0.02222	0.8565023
rs11680034	2	199348665	C	T	0.294	1.00	0.14	0.042	0.0009501	0.0006558		0.267	0.83	-0.00399	0.02226	0.8577356
rs7582813	2	199348740	C	T	0.707	0.97	-0.142	0.043	0.0009419	0.0006436		0.733	0.83	0.003966	0.02231	0.8588183
rs17829392	2	199353729	C	T	0.300	0.91	0.141	0.044	0.001456	0.0009637		0.276	0.78	-0.00194	0.02276	0.9321519
rs12991894	2	199353985	G	T	0.776	0.79	-0.176	0.052	0.00074	0.0015111		0.789	0.69	0.017723	0.02675	0.5072629
rs17233334	2	199363301	A	T	0.292	0.88	0.15	0.045	0.0009054	0.0005929		0.267	0.77	-0.00345	0.02312	0.8812743
rs1013162	2	199363458	A	G	0.292	0.85	0.153	0.046	0.0009032	0.0005818		0.267	0.76	-0.00338	0.02328	0.8843944
rs1878663	2	199363765	G	T	0.708	0.84	-0.153	0.046	0.0008995	0.0005787		0.733	0.75	0.003283	0.0234	0.8883368
rs849243	2	206003364	A	C	0.051	0.86	0.256	0.094	0.00667	0.000672		0.055	0.71	-0.03753	0.04864	0.4400124
rs12613990	2	221162593	A	C	0.429	1.00	-0.135	0.039	0.0006074	0.0008025		0.485	1.00	-0.0295	0.01843	0.1092975
rs10932873	2	221165868	C	T	0.572	1.00	0.136	0.04	0.000555	0.0007327		0.515	1.00	0.029078	0.01846	0.115005
rs1850490	2	221167017	A	T	0.428	1.00	-0.137	0.04	0.0005423	0.0007206		0.485	1.00	-0.02895	0.01847	0.1166465
rs6716476	2	221169084	C	T	0.590	1.00	0.131	0.04	0.0009555	0.0013757		0.535	0.99	0.023514	0.01855	0.2046226
rs6730649	2	221169674	A	G	0.428	1.00	-0.137	0.04	0.0005183	0.0006983		0.485	1.00	-0.02882	0.01847	0.1183313
rs1517441	2	221170201	A	T	0.590	1.00	0.132	0.04	0.0009098	0.0013294		0.535	0.99	0.023377	0.01855	0.207219
rs1589097	2	221172714	C	G	0.410	1.00	-0.132	0.04	0.0008799	0.0012995		0.465	1.00	-0.02314	0.01854	0.211614
rs1589098	2	221172748	C	T	0.410	1.00	-0.132	0.04	0.0008722	0.001292		0.466	1.00	-0.02307	0.01854	0.2130292
rs1607212	2	221172954	A	C	0.409	1.00	-0.132	0.04	0.0008483	0.0012525		0.466	1.00	-0.02302	0.01854	0.213927
rs1607213	2	221173301	A	G	0.409	1.00	-0.133	0.04	0.0008257	0.0012153		0.466	1.00	-0.02297	0.01853	0.2147563
rs10169304	2	221173426	C	T	0.421	0.96	-0.137	0.04	0.0006796	0.0010464		0.473	0.97	-0.02367	0.01876	0.2068239
rs12619185	2	221173799	A	C	0.409	1.00	-0.134	0.04	0.0007384	0.0010704		0.466	1.00	-0.02288	0.01853	0.2165798
rs1850492	2	221179425	C	T	0.427	1.00	-0.14	0.039	0.0003937	0.0005281		0.486	1.00	-0.02808	0.01845	0.1276798
rs1356397	2	221182102	C	T	0.591	1.00	0.134	0.04	0.0007006	0.0010078		0.534	1.00	0.022608	0.01852	0.2218801
rs2392718	2	221183329	A	G	0.573	1.00	0.14	0.039	0.0003786	0.0005043		0.515	1.00	0.02794	0.01845	0.1295941
rs1949415	2	221186337	A	C	0.409	1.00	-0.135	0.04	0.0006743	0.0009642		0.466	1.00	-0.02234	0.01852	0.2273427
rs10207622	2	221186479	G	T	0.591	1.00	0.135	0.04	0.0006681	0.0009537		0.534	1.00	0.022183	0.01852	0.2305962
rs7558221	2	221195011	A	G	0.567	1.00	0.135	0.039	0.0005998	0.0007445		0.498	0.97	0.026278	0.01874	0.1604903
rs6719602	2	221198679	A	G	0.567	1.00	0.134	0.039	0.000654	0.0007815		0.498	0.97	0.026075	0.01874	0.1638685
rs1568179	2	221201003	A	G	0.455	1.00	-0.14	0.039	0.0003735	0.0004722		0.524	0.99	-0.02487	0.01859	0.1806851
rs12471834	2	221206973	A	C	0.437	1.00	-0.134	0.039	0.0006498	0.0008959		0.505	1.00	-0.019	0.0186	0.3068235
rs11684111	2	221229405	C	G	0.508	0.90	0.145	0.041	0.0004511	0.0005152		0.458	0.91	0.027168	0.0195	0.1632425
rs11896343	2	221238122	A	G	0.453	1.00	-0.144	0.039	0.0002532	0.0004799		0.510	0.99	-0.02805	0.01871	0.1334815
rs7582123	2	221242177	C	G	0.453	1.00	-0.144	0.039	0.0002511	0.0004819		0.510	0.99	-0.0281	0.0187	0.1326547
rs10932878	2	221243274	C	T	0.453	1.00	-0.144	0.039	0.0002499	0.0004832		0.510	0.99	-0.02816	0.0187	0.1318369
rs10932879	2	221243491	C	T	0.547	1.00	0.144	0.039	0.0002475	0.0004859		0.490	0.99	0.028167	0.0187	0.1316703
rs7558655	2	221246686	C	T	0.548	1.00	0.143	0.039	0.0002775	0.0005182		0.491	0.99	0.028251	0.01869	0.1303661
rs1517449	2	221248065	A	G	0.452	1.00	-0.143	0.039	0.0002896	0.0005304		0.509	0.99	-0.02829	0.01869	0.129772
rs16863657	2	222872762	A	G	0.884	0.68	0.216	0.074	0.003708	0.0003937		0.874	0.62	-0.04114	0.03668	0.2616719
rs11884475	2	223714735	C	T	0.929	0.99	0.269	0.076	0.0004054	0.0007372		0.933	0.91	-0.00756	0.03683	0.8372563
rs11894487	2	223719165	A	T	0.928	1.00	0.272	0.076	0.0003205	0.0006252		0.933	0.97	-0.00661	0.03553	0.8523516
rs16864483	2	223720380	A	G	0.928	1.00	0.272	0.076	0.0003202	0.0006267		0.933	0.97	-0.00657	0.03553	0.8531333
rs16864484	2	223720562	A	C	0.928	1.00	0.272	0.076	0.0003198	0.0006284		0.933	0.97	-0.00656	0.03553	0.853326
rs11899732	2	223731461	C	T	0.072	1.00	-0.272	0.076	0.000317	0.0007246		0.067	0.97	0.006091	0.0356	0.8640588
rs11902770	2	223736877	C	T	0.928	1.00	0.272	0.076	0.0003165	0.0007241		0.937	0.97	0.008044	0.03647	0.8252736
rs11674099	2	225011266	A	G	0.035	1.00	0.284	0.105	0.006832	0.0009747		0.048	0.95	-0.03656	0.04242	0.3884884
rs2442322	2	232033024	A	G	0.305	0.84	-0.146	0.046	0.001452	0.0001347		0.283	0.83	0.035558	0.02222	0.1093161
rs7583124	2	233543898	C	T	0.412	1.00	0.139	0.04	0.000495	0.0038248		0.434	0.99	-0.00909	0.01831	0.6192288
rs11678495	2	233546691	A	G	0.412	1.00	0.138	0.04	0.0005126	0.0039312		0.434	1.00	-0.00919	0.01826	0.6145013
rs6437079	2	233547418	A	G	0.582	1.00	-0.137	0.04	0.0005678	0.0028146		0.566	1.00	0.009269	0.01826	0.6113381
rs954526	2	233548307	A	G	0.417	0.99	0.14	0.04	0.0004553	0.0023547		0.434	0.99	-0.00949	0.01831	0.6040087
rs938572	2	233549544	A	G	0.568	0.98	-0.148	0.04	0.0002003	0.000779		0.554	0.96	0.012542	0.01854	0.4982762
rs938573	2	233549610	A	G	0.431	0.98	0.15	0.04	0.0001818	0.0007161		0.444	0.95	-0.01202	0.01863	0.5183977
rs1104953	2	233578610	C	G	0.357	0.99	0.142	0.04	0.0004585	0.0028835		0.343	0.78	-0.02146	0.02126	0.3125095
rs10169320	2	233579863	A	G	0.357	1.00	0.142	0.04	0.0004537	0.0028726		0.344	0.79	-0.02137	0.02126	0.3143405
rs10169752	2	233580293	A	G	0.357	1.00	0.141	0.04	0.0004825	0.0030836		0.344	0.79	-0.02138	0.02125	0.3141422
rs13396115	2	233590956	C	T	0.649	0.99	-0.138	0.041	0.0006985	0.0031859		0.688	0.94	0.01988	0.02004	0.3208467
rs11673739	2	233736810	A	G	0.758	1.00	-0.149	0.045	0.0009547	0.0007024		0.777	0.99	0.020755	0.02219	0.3491885
rs10929180	2	233737595	C	G	0.242	1.00	0.149	0.045	0.0009668	0.0007014		0.223	0.99	-0.0208	0.02221	0.3485945
rs10175194	2	233738858	C	T	0.758	1.00	-0.148	0.045	0.001046	0.0006224		0.773	0.92	0.022374	0.02282	0.3265596
rs11889446	2	233740362	A	G	0.758	1.00	-0.148	0.045	0.001068	0.000618		0.770	0.91	0.022998	0.02299	0.3167643
rs10803668	2	233742397	G	T	0.758	1.00	-0.148	0.045	0.001078	0.0006162		0.770	0.91	0.02307	0.023	0.3155145
rs13408647	2	233745250	A	G	0.206	1.00	0.139	0.047	0.003339	0.0006596		0.193	0.91	-0.04557	0.02446	0.062267
rs10193017	2	233746895	A	G	0.207	0.99	0.14	0.048	0.003112	0.000604		0.195	0.94	-0.04456	0.02402	0.0633276
rs10179304	2	233746972	A	T	0.208	0.99	0.141	0.048	0.002954	0.0005672		0.195	0.94	-0.04445	0.02396	0.0633889
rs4436949	2	233757131	A	G	0.776	1.00	-0.186	0.047	0.0000658	0.0000653		0.758	1.00	0.00633	0.02162	0.769459
rs4663445	2	235168591	C	T	0.573	0.62	-0.166	0.049	0.0007733	0.0152134		0.555	0.20	0.022043	0.03956	0.5770599
rs1316476	2	238172838	C	T	0.298	1.00	-0.142	0.043	0.0008754	0.003664		0.284	1.00	-0.00044	0.0199	0.9821873
rs13414926	2	238189006	C	T	0.363	1.00	0.134	0.04	0.0007733	0.0016674		0.360	1.00	0.009904	0.01857	0.5935223
rs1151801	2	238382993	A	T	0.853	0.92	0.177	0.057	0.001815	0.0008592		0.867	0.86	-0.00565	0.02916	0.8460817
rs1198814	2	238406798	C	T	0.174	1.00	-0.162	0.051	0.001389	0.000854		0.181	1.00	0.014988	0.02429	0.5368512
rs11692472	2	238468125	C	G	0.093	0.99	-0.234	0.067	0.0005211	0.0081275		0.079	0.35	0.022359	0.05736	0.6964579
rs11675972	2	238468217	A	G	0.093	1.00	-0.234	0.067	0.00052	0.0081152		0.079	0.35	0.022225	0.05723	0.6974892
rs11679505	2	240164313	C	T	0.974	0.98	0.438	0.125	0.0004606	0.0007351		0.976	0.68	-0.00886	0.07577	0.9068336
rs11684324	2	240175845	A	G	0.026	0.91	-0.436	0.13	0.0008286	0.0017743		0.023	0.79	0.002705	0.07221	0.9700876
rs12470565	2	240305049	C	T	0.443	0.83	0.132	0.043	0.00192	0.0007563		0.493	0.81	0.034997	0.02051	0.087686
rs3806680	3	3127384	A	G	0.734	0.84	-0.163	0.048	0.0006236	0.0045783		0.776	0.82	0.000713	0.02394	0.976219
rs9814648	3	3130229	A	G	0.272	1.00	0.145	0.043	0.0008445	0.0030962		0.229	0.99	0.006422	0.02166	0.7667178
rs9836040	3	4916141	C	T	0.181	0.92	0.205	0.053	0.0001191	0.000087		0.233	0.88	-0.00486	0.02324	0.8343655
rs1531939	3	7592025	C	G	0.735	0.97	0.132	0.044	0.0029	0.0006898		0.730	0.95	0.011383	0.02098	0.587185
rs3804910	3	7592847	C	T	0.740	1.00	0.133	0.044	0.002534	0.0005684		0.737	0.99	0.011227	0.02076	0.5882669
rs3804908	3	7594230	G	T	0.740	1.00	0.133	0.044	0.002529	0.0005683		0.737	0.99	0.011182	0.02074	0.5894711

					GOYA QC		GOYA Overweight/control			GOYA BMI continuous	known	IARC QC		IARC results		
MARKER	chr	position	A1	A2	FREQ1	Rsqr	Beta	SE	p	p	gene name	freq1	Rsqr	in_beta	in_SE	in_p
rs9826424	3	7596512	C	T	0.740	1.00	0.133	0.044	0.002388	0.0005409		0.737	1.00	0.011398	0.02071	0.581732
rs3804906	3	7596654	A	C	0.260	1.00	-0.133	0.044	0.002371	0.0005369		0.263	1.00	-0.01146	0.02071	0.5796289
rs3804904	3	7596977	G	T	0.740	1.00	0.134	0.044	0.002334	0.0005291		0.737	1.00	0.011499	0.02071	0.5783363
rs3792460	3	7597270	C	T	0.260	1.00	-0.134	0.044	0.002298	0.0005215		0.263	1.00	-0.01157	0.0207	0.5760793
rs10510373	3	7886027	C	G	0.105	1.00	-0.185	0.064	0.003802	0.0002814		0.083	0.98	0.062756	0.03369	0.0622956
rs9818393	3	11484668	C	T	0.602	0.98	0.152	0.04	0.0001523	0.0001698		0.625	0.98	-0.01191	0.01894	0.5293277
rs7617187	3	11580944	C	G	0.447	0.70	0.176	0.046	0.0001464	0.0001769		0.446	0.59	-0.02643	0.02397	0.2696828
rs6781822	3	11650632	C	T	0.624	1.00	-0.146	0.04	0.0002924	0.0001222		0.633	0.90	0.009166	0.02015	0.6488769
rs2616543	3	11656487	A	C	0.675	0.89	-0.142	0.044	0.001187	0.00049		0.673	0.78	0.011923	0.02214	0.5899854
rs4603942	3	13679114	C	T	0.528	0.97	-0.127	0.039	0.0009531	0.0042714		0.513	0.94	-0.0264	0.01869	0.1574996
rs6792244	3	13692200	A	G	0.425	1.00	0.131	0.039	0.0006858	0.0045905		0.434	1.00	0.020505	0.01837	0.2640362
rs11128653	3	13692664	A	G	0.425	1.00	0.131	0.039	0.0006607	0.0043317		0.434	1.00	0.020405	0.01837	0.2663362
rs6775581	3	13695098	C	T	0.425	1.00	0.132	0.039	0.0006411	0.0041238		0.434	1.00	0.020332	0.01837	0.2679893
rs4557142	3	13697408	A	C	0.423	0.98	0.128	0.039	0.0009972	0.0064437		0.438	0.96	0.020553	0.01874	0.2724022
rs4383497	3	13697636	A	G	0.423	0.98	0.128	0.039	0.0009997	0.0064597		0.438	0.96	0.020563	0.01874	0.2722411
rs9849455	3	13701271	A	G	0.466	0.98	0.137	0.039	0.0004053	0.0018363		0.492	0.95	0.026876	0.01856	0.1472943
rs9849613	3	13701335	A	G	0.461	1.00	0.128	0.038	0.000828	0.0031982		0.492	0.95	0.026776	0.01857	0.1490985
rs6781368	3	13701841	A	G	0.569	1.00	-0.128	0.039	0.0009163	0.0075968		0.555	0.94	-0.0215	0.01894	0.2558895
rs6794344	3	13701889	C	T	0.540	1.00	-0.128	0.038	0.0008542	0.0033037		0.508	0.95	-0.02591	0.01865	0.1643128
rs6781488	3	13702087	C	G	0.460	1.00	0.128	0.038	0.0008668	0.0033299		0.493	0.94	0.025749	0.01866	0.1672145
rs6795216	3	13705683	G	T	0.460	1.00	0.127	0.038	0.000923	0.0034528		0.493	0.94	0.025686	0.01866	0.1684347
rs9826161	3	13705961	G	T	0.540	1.00	-0.128	0.038	0.0008556	0.0032992		0.507	0.94	-0.02569	0.01867	0.1685627
rs9830904	3	13706646	C	T	0.568	0.99	-0.129	0.039	0.0008947	0.0072971		0.555	0.93	-0.02131	0.01897	0.261016
rs9848869	3	13706857	A	G	0.625	0.95	0.14	0.041	0.000565	0.0013269		0.681	0.92	0.016331	0.02039	0.4228929
rs7638294	3	18650422	C	T	0.651	1.00	-0.152	0.041	0.0002454	0.0095114		0.614	0.99	-0.03411	0.01895	0.0716535
rs11128892	3	18659750	A	G	0.668	1.00	-0.138	0.042	0.0009302	0.0189447		0.641	1.00	-0.03182	0.01914	0.0961362
rs10780005	3	18668183	C	T	0.669	1.00	-0.139	0.042	0.0009278	0.0186719		0.642	0.99	-0.03206	0.01914	0.0937093
rs13073817	3	18681862	A	G	0.332	1.00	0.14	0.042	0.0008396	0.0169974		0.359	0.98	0.03168	0.01926	0.0997485
rs2201910	3	20970853	C	T	0.598	0.98	0.136	0.04	0.0007084	0.0004413		0.529	0.72	0.017664	0.02161	0.4134263
rs4858283	3	20971219	A	G	0.408	0.99	-0.145	0.04	0.000274	0.0001674		0.477	0.71	-0.01846	0.02172	0.3951757
rs6791186	3	20971403	C	T	0.560	0.87	0.15	0.042	0.0003345	0.0007009		0.495	0.60	0.024032	0.02397	0.3156611
rs12489570	3	21978369	C	T	0.354	0.96	0.145	0.042	0.0004973	0.0092845		0.288	0.95	0.014225	0.02055	0.4884735
rs7619247	3	21979462	C	G	0.341	1.00	0.136	0.041	0.0009364	0.0160066		0.272	1.00	0.024897	0.02035	0.2207791
rs7642008	3	21979551	A	C	0.341	1.00	0.136	0.041	0.0009346	0.0159892		0.272	1.00	0.024879	0.02034	0.221004
rs12487974	3	21980309	C	T	0.659	1.00	-0.136	0.041	0.0009057	0.0156423		0.728	1.00	-0.02476	0.02033	0.2228459
rs6765798	3	21984026	C	T	0.618	0.90	-0.151	0.042	0.0003527	0.0084433		0.687	0.88	-0.02689	0.02077	0.1952144
rs1585863	3	21987422	A	G	0.659	1.00	-0.138	0.041	0.0008105	0.0138675		0.728	1.00	-0.02415	0.02031	0.2339754
rs2291818	3	21994798	A	G	0.659	1.00	-0.138	0.041	0.0007946	0.0135287		0.728	1.00	-0.02412	0.02031	0.2346054
rs12638192	3	21996634	A	T	0.341	1.00	0.138	0.041	0.0007982	0.014064		0.272	1.00	0.024167	0.02033	0.2340959
rs1162135	3	22746581	A	T	0.953	0.66	-0.36	0.112	0.001335	0.0003115		0.955	0.56	0.013247	0.05569	0.8118277
rs7643926	3	24682097	C	T	0.581	0.97	-0.136	0.04	0.0006986	0.0030079		0.557	0.97	-0.01598	0.01841	0.3848868
rs1551762	3	30681394	A	T	0.833	0.84	0.142	0.057	0.01206	0.0007977		0.824	0.45	-0.02295	0.0351	0.5127874
rs11716829	3	34255102	A	G	0.016	0.34	-0.712	0.27	0.008367	0.0004814		0.018	0.24	-0.11049	0.1463	0.4497567
rs13061600	3	35620822	C	T	0.947	0.88	0.31	0.093	0.0008535	0.0014592		0.940	0.44	0.093741	0.05867	0.1098102
rs2137581	3	39221146	G	T	0.712	0.98	-0.148	0.043	0.0006088	0.0036728		0.686	0.94	-0.0108	0.02034	0.5950223
rs11720021	3	39229759	A	G	0.287	1.00	0.144	0.043	0.0007823	0.0048985		0.300	1.00	0.007414	0.02007	0.7116384
rs11707681	3	39232896	C	T	0.692	0.97	-0.141	0.042	0.0008384	0.0037993		0.687	0.98	-0.01071	0.01991	0.5902398
rs9825080	3	44226274	C	T	0.743	0.89	-0.141	0.047	0.00259	0.0007121		0.722	0.87	-0.01646	0.022	0.4541845
rs9284879	3	44259588	A	G	0.489	1.00	0.131	0.039	0.0008411	0.0005343		0.476	1.00	0.001282	0.01815	0.9436508
rs1488623	3	44266854	C	T	0.511	1.00	-0.131	0.039	0.0008298	0.0005293		0.524	1.00	-0.00144	0.01815	0.936538
rs2171569	3	44267403	A	G	0.511	1.00	-0.131	0.039	0.0008246	0.0005268		0.524	1.00	-0.00145	0.01815	0.9364011
rs13067367	3	44268279	C	T	0.483	0.99	0.131	0.04	0.0009298	0.0005848		0.468	0.98	0.001685	0.01832	0.9266499
rs9311352	3	44268843	C	G	0.511	1.00	-0.131	0.039	0.0008221	0.0005256		0.524	1.00	-0.00145	0.01815	0.936341
rs4682735	3	44270188	C	T	0.489	1.00	0.131	0.039	0.0008183	0.0005237		0.476	1.00	0.001451	0.01816	0.9362478
rs4682960	3	44271407	C	T	0.489	1.00	0.132	0.039	0.0008146	0.0005219		0.476	1.00	0.001455	0.01816	0.9360608
rs7631790	3	44274209	C	T	0.511	1.00	-0.132	0.039	0.0008096	0.0005195		0.524	1.00	-0.00146	0.01816	0.9359542
rs1124124	3	44277549	A	T	0.511	1.00	-0.132	0.039	0.000807	0.0005182		0.524	1.00	-0.00146	0.01816	0.9358326
rs9821268	3	44278128	A	G	0.511	1.00	-0.132	0.039	0.0008058	0.0005176		0.524	1.00	-0.00146	0.01816	0.9357347
rs11130023	3	44282892	A	G	0.511	1.00	-0.132	0.039	0.0008021	0.0005158		0.524	1.00	-0.00147	0.01816	0.935522
rs12631341	3	44284272	A	G	0.511	1.00	-0.132	0.039	0.0008009	0.0005152		0.524	1.00	-0.00147	0.01816	0.935446
rs6768749	3	44284624	G	T	0.511	1.00	-0.132	0.039	0.0007984	0.000514		0.524	1.00	-0.00147	0.01816	0.9354373
rs7349467	3	44289782	C	T	0.511	1.00	-0.132	0.039	0.0007948	0.0005122		0.524	1.00	-0.00147	0.01816	0.9352711
rs1387046	3	44299118	C	G	0.511	1.00	-0.132	0.039	0.0007935	0.0005116		0.524	1.00	-0.00148	0.01816	0.9351355
rs12486452	3	44299569	A	G	0.489	1.00	0.132	0.039	0.0007911	0.0005104		0.476	1.00	0.001476	0.01816	0.9351524
rs7649004	3	44300475	C	G	0.489	1.00	0.132	0.039	0.0007886	0.0005092		0.476	1.00	0.001478	0.01816	0.9350904
rs6803491	3	44308157	A	T	0.489	1.00	0.132	0.039	0.0007793	0.0005046		0.476	1.00	0.001528	0.01816	0.9328881
rs1565215	3	44309103	C	T	0.489	1.00	0.132	0.039	0.0007698	0.0004993		0.476	1.00	0.001602	0.01817	0.929684
rs1565214	3	44309303	A	C	0.489	1.00	0.132	0.039	0.0007686	0.0004987		0.476	1.00	0.001624	0.01817	0.9287206
rs6780605	3	44313246	A	G	0.489	1.00	0.132	0.039	0.0007646	0.0004965		0.476	1.00	0.001675	0.01817	0.926497
rs1488638	3	44317658	A	C	0.512	1.00	-0.132	0.039	0.0007619	0.000495		0.524	1.00	-0.0017	0.01817	0.9253359
rs2372598	3	44318366	C	T	0.489	1.00	0.132	0.039	0.0007598	0.0004937		0.476	1.00	0.00173	0.01817	0.9240997
rs7630980	3	44323127	A	G	0.512	1.00	-0.133	0.039	0.0007446	0.0004854		0.524	1.00	-0.00191	0.01818	0.9163225
rs9853705	3	44328149	G	T	0.486	0.99	0.134	0.039	0.0007028	0.0004283		0.469	0.99	0.004393	0.01827	0.8098235
rs12386385	3	44432544	A	G	0.620	1.00	-0.134	0.04	0.0007981	0.0016736		0.581	0.98	-0.01165	0.01861	0.5310013
rs1402751	3	44569034	A	C	0.352	0.98	0.136	0.041	0.0008529	0.0012088		0.400	0.97	0.016027	0.01886	0.3951048
rs2228561	3	48603018	A	G	0.134	1.00	-0.134	0.057	0.01823	0.0007171		0.114	0.99	-0.00325	0.02769	0.9064627
rs2215466	3	52464114	A	G	0.049	0.97	0.211	0.09	0.01961	0.0008239		0.043	0.97	0.047597	0.04673	0.3080193
rs9855470	3	52468315	A	G	0.050	0.98	0.216	0.089	0.01545	0.0005227		0.043	0.98	0.046476	0.0466	0.3181953
rs3774409	3	52471269	C	T	0.050	0.98	0.216	0.089	0.01537	0.0005173		0.043	0.98	0.046363	0.04658	0.3191775
rs1011064	3	52479543	C	T	0.050	0.98	0.217	0.089	0.01518	0.0005054		0.043	0.98	0.046248	0.04657	0.3202187
rs929528	3	52480485	C	T	0.050	0.98	0.217	0.089	0.01482	0.0004832		0.043	0.98	0.046132	0.04654	0.3212251
rs4312651	3	52481322	A	G	0.050	0.98	0.217	0.089	0.01473	0.0004776		0.043	0.98	0.045601	0.04646	0.3259644

					GOYA QC		GOYA Overweight/control			GOYA BMI continuous	known	IARC QC		IARC results		
MARKER	chr	position	A1	A2	FREQ1	Rsqr	Beta	SE	p	p	gene name	freq1	Rsqr	in_beta	in_SE	in_p
rs6445486	3	52481531	A	G	0.050	0.99	0.217	0.089	0.01438	0.0004556		0.043	0.99	0.04546	0.04644	0.3272521
rs1011062	3	52481943	A	G	0.050	0.99	0.217	0.089	0.01433	0.000451		0.043	0.99	0.045354	0.04643	0.3282274
rs9867823	3	52488067	C	G	0.950	0.97	-0.218	0.089	0.01454	0.0004329		0.957	0.97	-0.04466	0.04705	0.3421048
rs4687618	3	52488980	C	T	0.950	0.97	-0.218	0.089	0.01457	0.0004338		0.957	0.97	-0.04464	0.04707	0.3425134
rs758803	3	52490573	C	T	0.950	0.97	-0.218	0.089	0.0146	0.0004337		0.957	0.97	-0.04465	0.04708	0.3424958
rs728408	3	52492246	A	G	0.950	0.97	-0.218	0.089	0.01465	0.0004331		0.958	0.96	-0.04466	0.04745	0.3461956
rs4687619	3	52493826	C	T	0.950	0.97	-0.218	0.089	0.01468	0.000432		0.958	0.96	-0.04464	0.04752	0.3471963
rs6810027	3	52499614	A	C	0.949	0.95	-0.218	0.09	0.01553	0.0004256		0.958	0.95	-0.04451	0.04801	0.3534833
rs758800	3	52504306	C	T	0.949	0.94	-0.217	0.09	0.0157	0.0004258		0.958	0.95	-0.0445	0.04803	0.3537591
rs9846089	3	52504813	G	T	0.949	0.94	-0.217	0.09	0.01574	0.0004273		0.958	0.95	-0.04428	0.04829	0.3587141
rs7620619	3	53816844	G	T	0.015	0.41	0.658	0.254	0.009525	0.0007755		0.010	0.14	0.158025	0.18882	0.402263
rs4077974	3	54201040	A	G	0.081	1.00	0.235	0.071	0.0009539	0.0006014		0.078	0.85	-0.00076	0.03834	0.9842359
rs11925114	3	56989122	A	G	0.040	0.69	-0.418	0.122	0.000619	0.0033196		0.032	0.53	0.123715	0.0696	0.0752518
rs9879276	3	60928629	A	G	0.288	1.00	-0.125	0.043	0.00345	0.0007503		0.286	1.00	-0.00019	0.02013	0.9924676
rs895713	3	63011522	A	G	0.378	0.99	-0.119	0.04	0.00267	0.000132		0.370	0.99	-0.01302	0.01886	0.4897052
rs10155055	3	63013464	C	T	0.423	1.00	-0.115	0.039	0.003054	0.0001288		0.413	1.00	-0.01314	0.01845	0.4759786
rs2367598	3	63026382	A	G	0.420	1.00	-0.11	0.039	0.004438	0.0001871		0.408	1.00	-0.01468	0.01844	0.4255713
rs12629597	3	63030390	A	G	0.626	1.00	0.115	0.04	0.003614	0.0001803		0.635	1.00	0.014083	0.01882	0.453893
rs7622156	3	63037218	C	T	0.401	1.00	-0.105	0.039	0.006919	0.000405		0.394	1.00	-0.01673	0.01861	0.3682643
rs7629251	3	63040134	C	T	0.357	0.99	-0.116	0.04	0.00392	0.0002619		0.351	0.99	-0.01627	0.01899	0.3911932
rs9844044	3	63041670	A	G	0.623	0.99	0.123	0.04	0.001938	0.0000988		0.635	0.99	0.014201	0.01885	0.4509814
rs1816941	3	63044146	A	G	0.403	0.99	-0.108	0.039	0.006176	0.0009609		0.391	0.99	-0.00898	0.01851	0.6272087
rs1900580	3	63045591	G	T	0.424	0.99	-0.114	0.039	0.003496	0.0004245		0.406	0.99	-0.00755	0.01844	0.6818686
rs7631847	3	63049756	C	T	0.641	1.00	0.124	0.04	0.001854	0.0001256		0.649	1.00	0.015582	0.01894	0.4101931
rs9843021	3	63053078	A	T	0.359	1.00	-0.124	0.04	0.001829	0.0001246		0.352	1.00	-0.01555	0.01894	0.4112414
rs4688374	3	63054541	A	G	0.406	1.00	-0.113	0.039	0.003887	0.0006242		0.392	1.00	-0.00864	0.01846	0.639383
rs7610130	3	63057156	A	G	0.595	0.99	0.112	0.039	0.004319	0.0007646		0.608	0.99	0.008762	0.01852	0.6359366
rs9866402	3	63061523	C	G	0.406	1.00	-0.113	0.039	0.003946	0.0006791		0.393	0.99	-0.00848	0.01848	0.6460246
rs9833079	3	63062034	A	G	0.406	1.00	-0.113	0.039	0.003871	0.000693		0.395	1.00	-0.00816	0.01847	0.6583517
rs7611505	3	63062213	A	G	0.360	1.00	-0.123	0.04	0.001979	0.0001607		0.355	1.00	-0.01495	0.01895	0.4298114
rs982678	3	63063355	A	C	0.452	1.00	-0.11	0.039	0.004295	0.0006273		0.439	1.00	-0.00967	0.01827	0.5962853
rs921160	3	63063832	A	G	0.405	1.00	-0.11	0.039	0.00475	0.0009172		0.396	1.00	-0.00816	0.01846	0.6582331
rs12715583	3	63065054	A	C	0.405	1.00	-0.111	0.039	0.00454	0.0008889		0.396	1.00	-0.00819	0.01847	0.6570067
rs6792395	3	63246525	A	G	0.466	1.00	-0.145	0.039	0.0002356	0.0000355		0.410	0.98	0.010769	0.01872	0.5646791
rs6792508	3	63246786	C	T	0.530	1.00	0.137	0.039	0.0004824	0.0000772		0.587	0.99	-0.0093	0.01856	0.6158168
rs6792896	3	63247008	A	G	0.470	1.00	-0.137	0.039	0.0004772	0.0000763		0.414	0.99	0.009308	0.01856	0.6156507
rs6792744	3	63247074	C	T	0.530	1.00	0.137	0.039	0.0004752	0.0000759		0.587	0.99	-0.00931	0.01856	0.6155372
rs6768281	3	63247098	A	C	0.530	1.00	0.137	0.039	0.0004732	0.0000756		0.587	0.99	-0.00932	0.01856	0.6152908
rs6801595	3	63247508	A	G	0.530	1.00	0.137	0.039	0.0004704	0.0000751		0.587	0.99	-0.00932	0.01856	0.6153125
rs6790463	3	63247597	C	T	0.470	1.00	-0.137	0.039	0.0004684	0.0000748		0.413	0.99	0.00932	0.01855	0.6151614
rs9867170	3	63247866	A	T	0.470	1.00	-0.137	0.039	0.0004674	0.0000746		0.413	0.99	0.009321	0.01855	0.6150964
rs6777885	3	63248108	C	T	0.530	1.00	0.137	0.039	0.0004657	0.0000743		0.587	0.99	-0.00932	0.01855	0.6150523
rs6793545	3	63248314	A	T	0.470	1.00	-0.137	0.039	0.0004647	0.0000741		0.413	0.99	0.009322	0.01855	0.6150754
rs9873263	3	63249342	C	T	0.470	1.00	-0.137	0.039	0.0004598	0.0000733		0.413	0.99	0.00936	0.01855	0.6135324
rs9815672	3	63249476	A	T	0.530	1.00	0.137	0.039	0.0004588	0.0000731		0.587	0.99	-0.00936	0.01855	0.6134476
rs9853765	3	63249619	C	G	0.530	1.00	0.138	0.039	0.0004554	0.0000725		0.587	0.99	-0.00936	0.01855	0.6133322
rs2367783	3	63250311	C	T	0.530	1.00	0.138	0.039	0.0004543	0.0000723		0.587	0.99	-0.00937	0.01855	0.6132739
rs4688390	3	63250891	C	T	0.530	1.00	0.138	0.039	0.0004535	0.0000722		0.587	0.99	-0.00937	0.01855	0.6131335
rs4688391	3	63250969	C	T	0.530	1.00	0.138	0.039	0.0004526	0.000072		0.587	1.00	-0.00947	0.01853	0.6090901
rs9857192	3	63253008	A	G	0.530	1.00	0.138	0.039	0.0004423	0.0000703		0.587	1.00	-0.0095	0.01853	0.6079411
rs9311870	3	63253351	C	T	0.530	1.00	0.138	0.039	0.0004382	0.0000695		0.587	1.00	-0.00951	0.01853	0.6075391
rs2887081	3	63253849	C	T	0.470	1.00	-0.138	0.039	0.0004372	0.0000694		0.413	1.00	0.009511	0.01853	0.607369
rs2367788	3	63256467	C	T	0.530	1.00	0.138	0.039	0.0004159	0.0000657		0.588	1.00	-0.00952	0.01852	0.6068979
rs1403703	3	63257690	C	T	0.530	1.00	0.138	0.039	0.0004241	0.0000664		0.588	1.00	-0.00953	0.01852	0.6065618
rs2056546	3	63258705	C	T	0.530	1.00	0.138	0.039	0.0004269	0.0000667		0.588	1.00	-0.00961	0.0185	0.6029791
rs2056545	3	63258741	C	T	0.530	1.00	0.138	0.039	0.0004283	0.0000669		0.588	1.00	-0.00962	0.0185	0.6028894
rs10510893	3	63260668	C	T	0.530	1.00	0.138	0.039	0.000447	0.0000693		0.588	1.00	-0.00964	0.0185	0.6021202
rs11130920	3	63260778	A	T	0.526	0.99	0.14	0.04	0.0003983	0.0000784		0.585	0.98	-0.00993	0.01864	0.5938576
rs870364	3	63261294	C	G	0.530	1.00	0.138	0.039	0.0004438	0.0000692		0.589	1.00	-0.00948	0.01851	0.6083354
rs1608452	3	63269730	A	T	0.470	1.00	-0.138	0.039	0.0004317	0.0000708		0.410	1.00	0.009092	0.0185	0.6227819
rs7626955	3	63269842	A	T	0.470	1.00	-0.138	0.039	0.0004315	0.0000709		0.410	1.00	0.009035	0.01849	0.6248156
rs1523436	3	63272944	C	T	0.469	1.00	-0.139	0.039	0.0004025	0.0000688		0.410	1.00	0.008976	0.01848	0.6269066
rs4688393	3	63274092	A	C	0.469	1.00	-0.139	0.039	0.0004019	0.0000677		0.409	1.00	0.008986	0.01848	0.6265539
rs1851666	3	63274989	A	G	0.469	1.00	-0.139	0.039	0.0004021	0.0000675		0.409	1.00	0.008987	0.01848	0.6264956
rs7636388	3	63281859	A	G	0.469	1.00	-0.139	0.039	0.000407	0.000065		0.409	1.00	0.008999	0.01849	0.626107
rs1451312	3	63282908	A	T	0.531	1.00	0.139	0.039	0.0004085	0.0000647		0.591	1.00	-0.00901	0.01849	0.6256987
rs11130924	3	63283200	C	T	0.469	1.00	-0.139	0.039	0.0004086	0.0000646		0.409	1.00	0.009012	0.01849	0.6256442
rs1349478	3	63287007	A	C	0.531	1.00	0.139	0.039	0.0004147	0.0000642		0.592	1.00	-0.00904	0.01848	0.6245487
rs2037571	3	63287738	C	T	0.474	0.99	-0.134	0.039	0.0007135	0.0001412		0.412	0.99	0.00941	0.01854	0.6113864
rs2037570	3	63288184	A	G	0.531	1.00	0.139	0.039	0.0004207	0.0000654		0.592	1.00	-0.00905	0.01848	0.6239094
rs11714718	3	63289608	G	T	0.531	1.00	0.139	0.039	0.0004267	0.0000666		0.592	1.00	-0.00908	0.01848	0.6229213
rs13093232	3	63994682	A	G	0.068	0.99	-0.264	0.077	0.0006333	0.0014298		0.075	0.67	0.025953	0.04487	0.5626404
rs6548660	3	67736343	C	T	0.461	1.00	0.12	0.039	0.002205	0.0009919		0.475	0.99	0.030821	0.01842	0.0939515
rs2885693	3	67738233	A	T	0.539	1.00	-0.12	0.039	0.002086	0.0009561		0.525	1.00	-0.03131	0.01834	0.0875551
rs2363086	3	67738366	C	T	0.539	1.00	-0.12	0.039	0.00208	0.0009544		0.525	1.00	-0.03134	0.01834	0.0871432
rs6778484	3	67738776	A	G	0.539	1.00	-0.121	0.039	0.002	0.000925		0.525	1.00	-0.03138	0.01833	0.0866432
rs2363085	3	67738821	A	T	0.462	1.00	0.121	0.039	0.001961	0.0009109		0.476	1.00	0.031442	0.01832	0.0858919
rs2363084	3	67738892	C	T	0.462	1.00	0.121	0.039	0.001924	0.0008974		0.476	1.00	0.031459	0.01832	0.0856706
rs9844454	3	67739359	C	T	0.539	1.00	-0.121	0.039	0.001906	0.0008907		0.524	1.00	-0.03147	0.01832	0.0855557
rs7629617	3	67739382	C	T	0.462	1.00	0.122	0.039	0.00187	0.0008776		0.476	1.00	0.031501	0.01831	0.0851397
rs7627391	3	67739439	A	G	0.539	1.00	-0.122	0.039	0.001835	0.0008647		0.524	1.00	-0.03152	0.01831	0.0848853
rs6796037	3	67740187	G	T	0.538	1.00	-0.122	0.039	0.001823	0.0008766		0.524	1.00	-0.03158	0.01831	0.0843524

					GOYA QC		GOYA Overweight/control			GOYA BMI continuous	known	IARC QC		IARC results		
MARKER	chr	position	A1	A2	FREQ1	Rsqr	Beta	SE	p	p	gene name	freq1	Rsqr	in_beta	in_SE	in_p
rs6796133	3	67740282	A	G	0.462	1.00	0.122	0.039	0.001815	0.0008862		0.476	1.00	0.031608	0.01831	0.0840824
rs6772356	3	67740339	C	G	0.538	1.00	-0.122	0.039	0.001813	0.0008895		0.524	1.00	-0.03164	0.01831	0.0838066
rs9873616	3	67740664	C	T	0.462	1.00	0.122	0.039	0.00181	0.0008929		0.476	1.00	0.031678	0.01831	0.0834178
rs9811518	3	67740724	G	T	0.538	1.00	-0.122	0.039	0.001809	0.0008964		0.525	1.00	-0.03171	0.01831	0.0831623
rs2290175	3	67742739	A	G	0.538	1.00	-0.122	0.039	0.001807	0.0009		0.525	1.00	-0.03173	0.01831	0.0828964
rs9309843	3	67742938	C	G	0.462	1.00	0.122	0.039	0.001803	0.0009074		0.476	1.00	0.03176	0.01831	0.0826385
rs9813992	3	67743505	C	T	0.462	1.00	0.122	0.039	0.001799	0.000915		0.476	1.00	0.031786	0.01831	0.0823902
rs9865853	3	67743518	C	T	0.538	1.00	-0.122	0.039	0.001796	0.0009228		0.525	1.00	-0.0318	0.01831	0.0822673
rs9814167	3	67743598	C	T	0.462	1.00	0.122	0.039	0.001795	0.0009268		0.476	1.00	0.031812	0.01831	0.0821457
rs11706848	3	67743702	C	T	0.462	1.00	0.122	0.039	0.001794	0.0009311		0.476	1.00	0.031825	0.01831	0.0820201
rs9875474	3	67744860	C	G	0.538	1.00	-0.122	0.039	0.001793	0.0009355		0.525	1.00	-0.03191	0.01831	0.0811739
rs9836781	3	67744995	A	G	0.462	1.00	0.122	0.039	0.001793	0.0009356		0.475	1.00	0.031939	0.01831	0.0809158
rs9877255	3	67746615	C	T	0.468	0.96	0.128	0.04	0.001312	0.0008865		0.477	0.93	0.032199	0.01896	0.0892705
rs6799003	3	67746728	A	G	0.462	1.00	0.122	0.039	0.001794	0.000936		0.475	1.00	0.032011	0.01831	0.0802037
rs6790570	3	67746800	C	T	0.462	1.00	0.122	0.039	0.001794	0.0009361		0.475	1.00	0.032047	0.01831	0.0798491
rs2242022	3	67746919	A	G	0.538	1.00	-0.122	0.039	0.001794	0.0009361		0.525	1.00	-0.03206	0.01831	0.0797495
rs2242021	3	67747120	C	T	0.462	1.00	0.122	0.039	0.001781	0.0009313		0.475	1.00	0.032064	0.01831	0.0797284
rs2242020	3	67747185	A	G	0.538	1.00	-0.122	0.039	0.001753	0.0009216		0.525	1.00	-0.03208	0.01831	0.0796553
rs2242019	3	67747509	C	T	0.462	1.00	0.123	0.039	0.00174	0.000917		0.475	1.00	0.032077	0.01832	0.0796612
rs1463233	3	67747620	A	T	0.462	1.00	0.123	0.039	0.001713	0.0009076		0.475	1.00	0.032083	0.01832	0.0796534
rs1463231	3	67747830	C	G	0.462	1.00	0.123	0.039	0.001661	0.0008894		0.475	1.00	0.032112	0.01833	0.0795589
rs6797199	3	67748571	C	T	0.462	1.00	0.124	0.039	0.001587	0.0008634		0.475	1.00	0.032147	0.01834	0.0793434
rs2363082	3	67749094	A	G	0.538	1.00	-0.124	0.039	0.001552	0.000851		0.525	0.99	-0.0324	0.01842	0.0782553
rs2122477	3	67749537	C	T	0.462	1.00	0.124	0.039	0.001529	0.0008429		0.475	0.99	0.032423	0.01842	0.0781294
rs1868674	3	67750490	C	T	0.462	1.00	0.124	0.039	0.001496	0.000831		0.475	0.99	0.032435	0.01843	0.0781693
rs6789513	3	67751964	A	G	0.538	1.00	-0.124	0.039	0.001474	0.0008232		0.525	0.98	-0.03245	0.01843	0.0780633
rs6792270	3	67752071	C	T	0.462	1.00	0.125	0.039	0.001452	0.0008156		0.475	0.98	0.032458	0.01843	0.078058
rs9828970	3	67752082	A	G	0.538	1.00	-0.125	0.039	0.001431	0.0008081		0.525	0.98	-0.03247	0.01844	0.0780239
rs9833802	3	67752561	C	T	0.462	1.00	0.125	0.039	0.00141	0.0008008		0.475	0.98	0.032476	0.01844	0.0779933
rs9810550	3	67752573	C	T	0.538	1.00	-0.125	0.039	0.0014	0.0007972		0.525	0.98	-0.03248	0.01844	0.0779416
rs4355299	3	67753002	A	G	0.538	1.00	-0.125	0.039	0.00138	0.0007901		0.525	0.98	-0.03249	0.01844	0.0778894
rs9835458	3	67753793	A	G	0.538	1.00	-0.125	0.039	0.00137	0.0007865		0.525	0.98	-0.0325	0.01845	0.0778913
rs7642995	3	67753904	C	T	0.462	1.00	0.125	0.039	0.001361	0.000783		0.475	0.98	0.032507	0.01845	0.077878
rs7629728	3	67754410	C	T	0.538	1.00	-0.126	0.039	0.001322	0.0007695		0.525	0.98	-0.03261	0.01849	0.0775083
rs7651495	3	67754617	C	G	0.462	1.00	0.125	0.039	0.001329	0.0007731		0.475	0.98	0.032625	0.01849	0.0774322
rs7646094	3	67754861	C	T	0.573	1.00	0.143	0.039	0.0002483	0.0000619		0.574	0.80	0.039097	0.02049	0.0561464
rs7623157	3	71307711	A	G	0.767	1.00	-0.154	0.046	0.0008639	0.0016696		0.752	0.82	-0.00456	0.02294	0.842245
rs3913574	3	76658543	A	G	0.450	1.00	0.131	0.039	0.0007246	0.0396577		0.457	1.00	-0.00579	0.01805	0.7483615
rs11127548	3	76681380	C	T	0.550	1.00	-0.134	0.039	0.0005349	0.0348487		0.545	0.99	0.005659	0.01813	0.7547803
rs1495893	3	86499470	C	T	0.984	1.00	0.424	0.159	0.007575	0.0009931		0.982	0.98	0.097659	0.0744	0.1889379
rs1495890	3	86518206	A	T	0.984	1.00	0.424	0.159	0.007583	0.0009949		0.982	0.98	0.097722	0.07443	0.1888303
rs12633748	3	86523535	C	G	0.016	1.00	-0.424	0.159	0.007586	0.0009954		0.018	0.98	-0.09784	0.07461	0.189405
rs11706497	3	89621056	C	T	0.903	1.00	0.229	0.067	0.0005752	0.0020802		0.912	0.80	0.07595	0.03503	0.0300304
rs12629811	3	107156666	C	T	0.737	1.00	-0.132	0.044	0.002833	0.0004148		0.731	0.91	0.002016	0.02149	0.9251783
rs9876108	3	108315962	C	T	0.884	0.77	-0.217	0.069	0.001677	0.0006004		0.895	0.77	-0.00652	0.0331	0.8438166
rs9863794	3	108318930	C	T	0.815	1.00	-0.17	0.05	0.0006552	0.0008519		0.777	0.94	0.035569	0.02252	0.1139461
rs4682438	3	114047353	C	G	0.119	0.99	0.203	0.061	0.0008456	0.0026262		0.151	0.99	-0.06652	0.02529	0.0084997
rs4682123	3	114080396	A	G	0.886	1.00	-0.205	0.061	0.0008111	0.0035745		0.852	1.00	0.055911	0.02569	0.0294013
rs4682124	3	114080534	C	G	0.886	1.00	-0.205	0.061	0.0008164	0.0035951		0.852	1.00	0.055948	0.02569	0.0293187
rs7652799	3	118669685	C	T	0.987	0.98	-0.587	0.176	0.000862	0.0009566		0.969	0.91	-0.03819	0.0508	0.4517653
rs1350967	3	118670020	A	G	0.013	0.98	0.588	0.176	0.0008675	0.000942		0.031	0.91	0.038339	0.05085	0.4505066
rs4688049	3	119018303	C	T	0.845	0.99	-0.156	0.053	0.003417	0.0007195		0.785	0.98	-0.0035	0.02355	0.8817927
rs9985380	3	119018895	A	G	0.155	0.99	0.157	0.053	0.003286	0.0006819		0.215	0.98	0.003344	0.02356	0.8870211
rs9839006	3	119038035	C	T	0.154	1.00	0.16	0.053	0.002754	0.0004941		0.216	0.99	-0.00385	0.02351	0.869657
rs9833554	3	119053347	A	C	0.822	0.99	-0.152	0.051	0.00288	0.000768		0.750	0.98	0.007342	0.02215	0.7400906
rs9874340	3	119066172	A	G	0.823	1.00	-0.155	0.051	0.002394	0.0005849		0.754	0.98	0.011935	0.02222	0.5908255
rs7622841	3	119074046	A	G	0.844	1.00	-0.164	0.053	0.002006	0.0003084		0.783	0.99	0.011481	0.0233	0.6218585
rs9812067	3	119077331	C	T	0.162	1.00	0.177	0.052	0.0007328	0.0001447		0.217	1.00	-0.00821	0.02322	0.7234246
rs9844592	3	119078338	C	G	0.824	1.00	-0.155	0.051	0.002314	0.0004776		0.758	0.99	0.01605	0.02222	0.469818
rs9860111	3	119080849	G	T	0.847	0.99	-0.163	0.054	0.002454	0.0003071		0.790	0.99	0.00731	0.02362	0.7567308
rs9860811	3	119081328	C	G	0.175	0.99	0.154	0.051	0.002595	0.0004956		0.241	0.99	-0.01579	0.02232	0.4790698
rs936202	3	119093308	A	T	0.829	0.99	-0.149	0.052	0.003982	0.0006372		0.763	0.99	0.01441	0.02247	0.5209414
rs936201	3	119093627	C	T	0.173	0.98	0.155	0.052	0.002771	0.0004832		0.240	0.98	-0.01669	0.02249	0.4577299
rs16827703	3	119101853	C	G	0.170	0.99	0.148	0.052	0.004218	0.0006645		0.236	0.99	-0.01412	0.02246	0.5290448
rs1350159	3	119143699	A	T	0.158	0.99	0.167	0.053	0.00164	0.0002447		0.220	0.98	-0.00164	0.02332	0.9439896
rs6784897	3	119146567	G	T	0.189	0.97	0.153	0.05	0.002377	0.0003079		0.248	0.99	-0.00294	0.02202	0.8936167
rs1902416	3	119148022	C	G	0.168	0.99	0.162	0.052	0.001683	0.0002134		0.226	0.99	0.002695	0.02292	0.9063023
rs10934425	3	119150930	C	T	0.848	0.99	-0.145	0.054	0.006757	0.0007983		0.783	0.99	-0.00172	0.02336	0.9411092
rs2870561	3	119151441	A	G	0.830	0.99	-0.162	0.051	0.001654	0.0002072		0.770	1.00	0.000693	0.02268	0.9756075
rs865309	3	119157489	A	G	0.811	0.97	-0.153	0.05	0.002395	0.000313		0.752	0.99	0.002762	0.02199	0.8999832
rs843853	3	119160491	A	C	0.830	1.00	-0.16	0.051	0.00181	0.0002331		0.769	1.00	0.000618	0.02267	0.9782462
rs2605530	3	119169248	A	C	0.829	1.00	-0.159	0.051	0.001891	0.0002469		0.769	1.00	0.000443	0.02264	0.9843836
rs2605532	3	119181490	C	T	0.162	1.00	0.146	0.052	0.00525	0.0005005		0.225	1.00	0.000796	0.02287	0.9722183
rs17723204	3	119187030	C	T	0.162	1.00	0.149	0.052	0.004214	0.0004561		0.225	1.00	0.003128	0.02289	0.8911982
rs861000	3	119191781	A	G	0.169	1.00	0.161	0.051	0.001677	0.0002608		0.228	1.00	0.005578	0.02285	0.8069637
rs817503	3	119202724	C	T	0.846	0.99	-0.146	0.053	0.00641	0.0009545		0.781	1.00	-0.00454	0.0233	0.8453873
rs817502	3	119204349	A	G	0.830	1.00	-0.155	0.051	0.002483	0.0004774		0.772	1.00	-0.00519	0.02289	0.8205504
rs861672	3	119205236	A	C	0.837	0.99	-0.143	0.052	0.006324	0.0008784		0.775	0.99	-0.00271	0.02299	0.9059485
rs1456198	3	119206879	A	C	0.159	0.98	0.145	0.053	0.006371	0.0009303		0.223	0.99	0.000991	0.02314	0.9658079
rs1378831	3	119224714	A	G	0.161	0.99	0.145	0.053	0.005991	0.0008419		0.219	0.97	0.005876	0.02363	0.803414
rs843855	3	119239225	A	G	0.159	0.97	0.149	0.053	0.005171	0.0007403		0.217	0.96	0.004425	0.02376	0.8521051
rs2062513	3	119239304	C	T	0.159	0.97	0.149	0.053	0.005202	0.0007426		0.217	0.96	0.004365	0.02376	0.8541267

					GOYA QC		GOYA Overweight/control			GOYA BMI continuous	known	IARC QC			IARC results	
MARKER	chr	position	A1	A2	FREQ1	Rsqr	Beta	SE	p	p	gene name	freq1	Rsqr	in_beta	in_SE	in_p
rs13078855	3	120589967	C	T	0.823	1.00	-0.172	0.051	0.0007876	0.0041544		0.839	1.00	0.011583	0.0246	0.6375073
rs3732419	3	120679024	G	T	0.494	1.00	-0.136	0.039	0.0004591	0.0013603		0.450	1.00	0.023312	0.01824	0.2009564
rs6799691	3	120679471	A	G	0.506	1.00	0.136	0.039	0.0004608	0.0013659		0.551	1.00	-0.02328	0.01824	0.2016242
rs12053990	3	120916198	A	G	0.974	0.89	-0.493	0.131	0.0001679	0.0004208		0.981	0.78	0.012863	0.06699	0.8476095
rs2700388	3	125125326	C	T	0.149	0.94	0.189	0.056	0.0007934	0.0008872		0.166	0.95	-0.01132	0.02464	0.6458004
rs6779131	3	125186517	A	T	0.128	0.90	0.194	0.061	0.001628	0.0004144		0.158	0.95	-0.01342	0.0252	0.593994
rs2332717	3	125190738	C	T	0.914	0.92	-0.236	0.073	0.001155	0.0002611		0.882	0.93	0.015909	0.02941	0.5883
rs4678074	3	125196222	C	G	0.913	0.97	-0.239	0.07	0.0006797	0.000172		0.881	0.96	0.013753	0.02888	0.6335719
rs7429448	3	125198542	A	G	0.100	0.99	0.23	0.065	0.000404	0.0005922		0.124	0.96	-0.01689	0.02845	0.5525566
rs2877780	3	125206138	C	T	0.900	1.00	-0.229	0.065	0.0004133	0.0005932		0.876	0.96	0.017045	0.02852	0.5496882
rs12629234	3	125287381	A	G	0.908	1.00	-0.214	0.067	0.001528	0.0008169		0.880	1.00	0.016747	0.02834	0.5542581
rs11721323	3	125289702	C	T	0.908	1.00	-0.215	0.067	0.001437	0.0007553		0.880	1.00	0.01702	0.02836	0.5480351
rs16835100	3	125307023	C	T	0.908	1.00	-0.215	0.067	0.001446	0.0007607		0.881	1.00	0.018263	0.0284	0.5199034
rs11714061	3	125308212	A	G	0.092	1.00	0.215	0.067	0.001447	0.0007612		0.119	1.00	-0.01831	0.02841	0.5189131
rs11710702	3	125312510	A	G	0.908	1.00	-0.217	0.068	0.0013	0.0006812		0.881	1.00	0.018536	0.02843	0.5140812
rs1316375	3	125316304	A	G	0.082	0.94	0.268	0.073	0.0002598	0.0002306		0.099	0.95	-0.02143	0.03147	0.4955381
rs1444767	3	125407493	G	T	0.921	1.00	-0.26	0.072	0.0003024	0.0000689		0.896	1.00	0.018891	0.02998	0.5282453
rs1822791	3	125407985	A	G	0.921	1.00	-0.26	0.072	0.0003029	0.000069		0.896	1.00	0.01892	0.03	0.527968
rs2084017	3	125410638	G	T	0.914	0.92	-0.256	0.072	0.0003945	0.00011		0.889	0.93	0.021646	0.03018	0.4728239
rs1444755	3	125422297	A	G	0.919	0.82	-0.231	0.078	0.002915	0.0006667		0.901	0.70	0.035379	0.03521	0.3145772
rs13078438	3	125465662	A	G	0.187	1.00	0.183	0.05	0.0002507	0.0016952		0.170	0.97	0.005999	0.02503	0.8104018
rs2276739	3	125466294	C	T	0.814	1.00	-0.182	0.05	0.0002556	0.0017386		0.836	0.99	-0.00156	0.02515	0.9505032
rs13064819	3	125468805	C	G	0.186	1.00	0.181	0.05	0.0002796	0.0019438		0.163	1.00	0.00127	0.02514	0.959695
rs9868324	3	125472134	A	G	0.186	1.00	0.181	0.05	0.0002845	0.0019751		0.163	1.00	0.001156	0.02514	0.9632884
rs9812928	3	125472155	G	T	0.186	1.00	0.181	0.05	0.0002853	0.0019801		0.163	1.00	0.001126	0.02514	0.9642279
rs12695435	3	125473453	C	T	0.814	1.00	-0.181	0.05	0.0002879	0.0019964		0.837	1.00	-0.0011	0.02514	0.9650682
rs13100913	3	125474937	A	C	0.186	1.00	0.181	0.05	0.0002898	0.002008		0.163	1.00	0.000775	0.02512	0.975374
rs9819643	3	125477254	C	G	0.814	1.00	-0.181	0.05	0.0002923	0.0020244		0.837	1.00	-0.00057	0.0251	0.9817553
rs7610173	3	125479313	A	G	0.189	0.99	0.185	0.05	0.0002034	0.0010579		0.175	0.96	0.000635	0.02477	0.9795318
rs9813330	3	125481425	G	T	0.814	1.00	-0.18	0.05	0.0003044	0.0020854		0.837	1.00	-0.00051	0.0251	0.9837443
rs4634050	3	125482120	A	C	0.186	1.00	0.18	0.05	0.0003048	0.0020887		0.163	1.00	0.000431	0.02509	0.9862737
rs7628367	3	125488511	A	G	0.813	1.00	-0.179	0.05	0.0003263	0.0027476		0.837	1.00	-0.00056	0.02511	0.9821678
rs13076923	3	125498689	A	G	0.813	1.00	-0.177	0.05	0.0003623	0.0030071		0.836	0.99	-0.002	0.02523	0.9367361
rs7623994	3	125499273	A	C	0.187	1.00	0.177	0.05	0.0003645	0.0030306		0.164	0.99	0.002001	0.02523	0.9367174
rs12695436	3	125500686	C	T	0.813	1.00	-0.177	0.05	0.0003666	0.0030537		0.836	0.98	-0.00202	0.02523	0.9362822
rs976634	3	125501112	C	T	0.187	1.00	0.177	0.05	0.0003709	0.0031002		0.164	0.98	0.002016	0.02524	0.9362701
rs4678107	3	125502077	C	T	0.187	1.00	0.177	0.05	0.0003731	0.0031237		0.164	0.98	0.002026	0.02524	0.9359743
rs9818967	3	125504552	A	C	0.813	1.00	-0.177	0.05	0.0003801	0.0031979		0.836	0.98	-0.00207	0.02525	0.9346685
rs4280578	3	125505432	A	G	0.187	1.00	0.176	0.05	0.0003836	0.0032343		0.164	0.98	0.002098	0.02526	0.9337631
rs1444752	3	125509248	A	T	0.187	1.00	0.176	0.05	0.0003844	0.0032433		0.164	0.98	0.002118	0.02527	0.9331486
rs6797486	3	125510189	C	T	0.813	1.00	-0.176	0.05	0.000385	0.0032497		0.836	0.98	-0.00213	0.02527	0.9328949
rs6797788	3	125510463	A	C	0.187	1.00	0.176	0.05	0.0003854	0.0032546		0.164	0.98	0.002133	0.02528	0.9326997
rs9861401	3	125510915	A	G	0.187	1.00	0.176	0.05	0.0003865	0.0032653		0.164	0.98	0.002142	0.02528	0.9324331
rs13082414	3	125512352	A	G	0.187	1.00	0.176	0.05	0.0003852	0.0032537		0.164	0.98	0.002175	0.02529	0.9313939
rs6793443	3	125514417	A	G	0.187	1.00	0.176	0.05	0.0003842	0.0032461		0.164	0.98	0.002193	0.02529	0.9308349
rs9832290	3	131241212	C	G	0.298	0.48	0.173	0.061	0.004307	0.0006311		0.271	0.29	0.006293	0.03837	0.8696187
rs1542829	3	131620161	A	G	0.062	0.97	0.262	0.083	0.001529	0.0008506		0.058	0.99	0.026701	0.04132	0.5178231
rs11927775	3	131630604	C	G	0.935	0.92	-0.3	0.083	0.0003075	0.0004791		0.937	0.91	-0.02185	0.04163	0.5993734
rs16828332	3	131664961	A	G	0.044	0.99	0.3	0.096	0.001877	0.0004176		0.040	0.98	0.03186	0.0503	0.5261112
rs16828578	3	131685052	A	C	0.957	1.00	-0.295	0.097	0.002262	0.0004811		0.961	1.00	-0.04293	0.05041	0.3940064
rs11719898	3	131686417	C	T	0.957	1.00	-0.295	0.097	0.002227	0.0004726		0.961	1.00	-0.04264	0.05041	0.3972736
rs949934	3	131705639	A	G	0.947	1.00	-0.286	0.088	0.001094	0.000646		0.946	0.99	-0.00871	0.04322	0.8400789
rs6792960	3	131711899	C	T	0.053	1.00	0.285	0.088	0.001174	0.0008465		0.054	0.99	0.003843	0.04314	0.9289457
rs16828872	3	131714199	C	T	0.065	0.98	0.282	0.08	0.0004442	0.0002838		0.067	0.78	0.007204	0.04384	0.8693516
rs11923817	3	131716607	C	G	0.065	0.96	0.285	0.081	0.0004515	0.0002837		0.067	0.76	0.007086	0.04433	0.8729005
rs6794690	3	131717240	C	T	0.065	0.96	0.286	0.081	0.0004547	0.0002841		0.067	0.74	0.00685	0.04478	0.8783382
rs187586	3	136106877	C	T	0.675	0.99	0.139	0.042	0.0008091	0.0014528		0.653	0.73	-0.01862	0.02217	0.4004599
rs2345270	3	138808080	A	G	0.090	0.81	0.265	0.075	0.0004188	0.000327		0.111	0.55	0.017027	0.03978	0.6683425
rs16847489	3	139007893	A	G	0.891	0.98	-0.199	0.064	0.001778	0.0003607		0.883	0.94	-0.05461	0.02977	0.0663853
rs12633893	3	148860190	A	G	0.940	0.93	0.308	0.085	0.0002969	0.0037265		0.945	0.95	-0.0371	0.04026	0.3562942
rs1879815	3	169285809	A	G	0.493	0.99	-0.098	0.039	0.01158	0.0009561		0.470	0.47	0.015743	0.0273	0.5637838
rs1357282	3	169304577	A	G	0.784	0.59	0.221	0.062	0.000362	0.0000573		0.813	0.48	-0.00472	0.03472	0.8918003
rs7637041	3	169311606	A	C	0.542	0.89	0.105	0.041	0.01089	0.0008466		0.560	0.86	-0.00474	0.01992	0.8116993
rs1608124	3	169352126	A	C	0.138	0.43	-0.284	0.086	0.0009876	0.0001749		0.124	0.40	-0.00405	0.04621	0.930123
rs2253178	3	169373412	A	C	0.481	1.00	-0.105	0.039	0.006879	0.0003035		0.473	1.00	0.004123	0.01835	0.8220829
rs16825520	3	176549958	C	T	0.867	0.99	-0.197	0.058	0.0006307	0.0044612		0.886	0.91	-0.04078	0.0294	0.1650761
rs12186003	3	179868784	A	G	0.683	1.00	-0.105	0.041	0.01081	0.0002039		0.696	0.99	0.02201	0.01989	0.2681864
rs6443558	3	179871353	C	T	0.317	1.00	0.105	0.041	0.01118	0.0002132		0.304	1.00	-0.02223	0.01978	0.2606465
rs4478059	3	179872105	C	T	0.315	0.99	0.104	0.041	0.01232	0.0002213		0.304	1.00	-0.02244	0.0198	0.2567439
rs9868067	3	179873655	A	G	0.686	0.99	-0.103	0.041	0.01328	0.0002324		0.696	1.00	0.022581	0.01981	0.2540215
rs7374977	3	179874115	A	G	0.313	0.99	0.102	0.041	0.01372	0.0002382		0.304	1.00	-0.02263	0.01982	0.2530911
rs6775196	3	179874893	C	T	0.313	1.00	0.102	0.041	0.01419	0.000245		0.304	1.00	-0.02272	0.01982	0.2513238
rs6765989	3	179875881	C	G	0.312	1.00	0.101	0.041	0.01478	0.000254		0.304	1.00	-0.0228	0.01982	0.2496341
rs6778882	3	179876050	C	T	0.312	1.00	0.101	0.041	0.01496	0.0002569		0.304	1.00	-0.02289	0.01983	0.2479708
rs12696457	3	179877570	A	G	0.688	1.00	-0.1	0.041	0.01533	0.0002628		0.696	1.00	0.022962	0.01983	0.2464405
rs13081714	3	179878625	G	T	0.688	1.00	-0.1	0.041	0.01552	0.0002659		0.696	1.00	0.02304	0.01983	0.2448398
rs6792781	3	179880116	C	T	0.312	1.00	0.1	0.041	0.01551	0.0002657		0.305	0.99	-0.02311	0.01985	0.2440981
rs6764174	3	179880305	C	T	0.688	1.00	-0.1	0.041	0.0155	0.0002655		0.695	0.99	0.023107	0.01985	0.2441033
rs6788934	3	179880728	A	G	0.688	1.00	-0.1	0.041	0.01549	0.0002652		0.695	0.99	0.023114	0.01986	0.2440233
rs13100616	3	179882245	A	G	0.688	1.00	-0.1	0.041	0.01548	0.000265		0.695	0.99	0.023116	0.01986	0.2440546
rs13064782	3	179883522	A	G	0.312	1.00	0.1	0.041	0.01548	0.000265		0.305	0.99	-0.02312	0.01986	0.2440147
rs13081178	3	179885835	A	G	0.688	1.00	-0.1	0.041	0.01547	0.0002647		0.695	0.99	0.023124	0.01986	0.2439643

					GOYA QC		GOYA Overweight/control			GOYA BMI continuous	known	IARC QC		IARC results		
MARKER	chr	position	A1	A2	FREQ1	Rsqr	Beta	SE	p	p	gene name	freq1	Rsqr	in_beta	in_SE	in_p
rs13088032	3	179887235	C	T	0.688	1.00	-0.1	0.041	0.01543	0.000264		0.695	0.99	0.02313	0.01986	0.2438948
rs13073955	3	179887709	A	G	0.688	1.00	-0.1	0.041	0.01541	0.0002636		0.695	0.99	0.023131	0.01987	0.2438913
rs4464427	3	179889219	A	G	0.688	1.00	-0.1	0.041	0.01537	0.0002627		0.695	0.99	0.023139	0.01987	0.243808
rs13092314	3	179890808	A	T	0.688	1.00	-0.1	0.041	0.01534	0.0002621		0.695	0.99	0.023141	0.01987	0.2437907
rs13092965	3	179890847	C	G	0.312	1.00	0.1	0.041	0.01532	0.0002619		0.305	0.99	-0.02316	0.01987	0.2435011
rs13062569	3	179893178	C	T	0.688	1.00	-0.1	0.041	0.0153	0.0002612		0.695	0.99	0.023158	0.01987	0.2435618
rs7433215	3	179893884	C	T	0.312	1.00	0.1	0.041	0.01528	0.0002611		0.305	0.99	-0.02318	0.01988	0.2432292
rs4560280	3	179899681	A	G	0.312	1.00	0.101	0.041	0.01521	0.0002598		0.305	0.99	-0.02318	0.01989	0.243411
rs12106872	3	179903170	C	T	0.312	1.00	0.101	0.041	0.01519	0.0002595		0.305	0.99	-0.02318	0.01989	0.2434214
rs7372467	3	179903904	C	T	0.312	1.00	0.101	0.041	0.01516	0.0002589		0.305	0.99	-0.0232	0.01989	0.2431341
rs7374960	3	179904016	A	G	0.312	1.00	0.101	0.041	0.01516	0.0002589		0.305	0.99	-0.02319	0.01989	0.2433106
rs13060090	3	179904983	A	C	0.271	0.91	0.113	0.045	0.01251	0.0005464		0.271	0.89	-0.02472	0.02163	0.2528587
rs13071153	3	179906545	A	C	0.287	0.94	0.11	0.044	0.01224	0.0004401		0.283	0.92	-0.02884	0.02103	0.1699403
rs6443565	3	179907526	C	T	0.312	1.00	0.101	0.041	0.01521	0.0002611		0.306	0.99	-0.0232	0.01989	0.2430511
rs6443566	3	179907676	A	G	0.312	1.00	0.101	0.041	0.01525	0.0002631		0.306	0.99	-0.0232	0.01989	0.2431237
rs13087051	3	179920260	C	T	0.867	0.96	-0.168	0.058	0.003776	0.0006559		0.862	0.93	0.013758	0.02744	0.6157677
rs13080577	3	179926080	G	T	0.867	0.97	-0.166	0.058	0.003996	0.0006991		0.861	0.94	0.013294	0.02727	0.6256086
rs13060883	3	179926410	C	T	0.135	0.99	0.161	0.057	0.004528	0.000802		0.142	0.98	-0.01153	0.02654	0.6636742
rs13082447	3	179927775	C	T	0.135	1.00	0.16	0.057	0.004735	0.0008431		0.143	0.99	-0.01118	0.02638	0.6714936
rs6783917	3	179928796	A	T	0.865	1.00	-0.16	0.057	0.004668	0.0008314		0.857	0.99	0.011247	0.02634	0.6691654
rs6772891	3	179929161	A	T	0.135	1.00	0.16	0.057	0.004648	0.0008293		0.143	0.99	-0.01131	0.02634	0.6674368
rs7612729	3	179935635	C	T	0.865	1.00	-0.162	0.057	0.00416	0.0007779		0.857	0.99	0.011855	0.02632	0.652151
rs7611588	3	179940440	C	T	0.865	1.00	-0.164	0.057	0.003819	0.0007367		0.857	0.99	0.011966	0.02631	0.6489235
rs12495431	3	180080215	C	T	0.919	0.94	0.262	0.073	0.0003067	0.0028004		0.905	0.91	0.024203	0.03277	0.4597595
rs10433478	3	180092784	C	T	0.644	1.00	-0.121	0.041	0.002986	0.0008218		0.633	1.00	-0.02842	0.01919	0.1382123
rs6804849	3	180103403	A	G	0.075	0.99	-0.257	0.073	0.0004596	0.0043899		0.083	0.97	-0.01223	0.03337	0.7136658
rs9872496	3	180121446	C	T	0.925	1.00	0.256	0.073	0.000473	0.0044795		0.917	0.98	0.012424	0.03329	0.7087487
rs9840960	3	182018077	C	T	0.089	0.97	0.208	0.069	0.002477	0.0009431		0.123	0.97	0.0388	0.02874	0.1765984
rs11925231	3	182021989	G	T	0.089	0.98	0.208	0.069	0.002471	0.0009427		0.123	0.98	0.038787	0.02869	0.1761075
rs9819622	3	182024635	A	G	0.911	0.98	-0.208	0.069	0.002472	0.0009427		0.877	0.98	-0.03878	0.02866	0.1756747
rs9845584	3	182027546	A	G	0.089	0.99	0.206	0.068	0.002539	0.0009686		0.123	0.98	0.038793	0.02866	0.1755052
rs9827910	3	182065190	A	T	0.097	1.00	0.216	0.066	0.0009756	0.0001937		0.128	0.97	0.041129	0.02819	0.1442261
rs1805612	3	182155887	A	G	0.956	0.77	-0.32	0.108	0.002935	0.0005568		0.953	0.71	-0.04427	0.05029	0.3783012
rs1805614	3	182156115	C	T	0.068	0.99	0.27	0.077	0.0004918	0.0000484		0.081	0.91	0.016988	0.03499	0.6269905
rs13081180	3	182581411	C	T	0.977	0.99	-0.431	0.131	0.000978	0.0083661		0.980	0.98	-0.12336	0.07051	0.0799829
rs11718683	3	182582367	A	T	0.977	0.99	-0.434	0.131	0.00093	0.0081947		0.980	0.98	-0.12324	0.07047	0.0800838
rs12636303	3	182586977	A	G	0.023	0.99	0.438	0.131	0.0008601	0.0079376		0.020	0.98	0.123089	0.07039	0.0801454
rs1518932	3	182601966	C	G	0.023	0.99	0.45	0.132	0.0006736	0.0073183		0.020	0.99	0.122433	0.0701	0.0804865
rs9821187	3	182619762	G	T	0.978	1.00	-0.455	0.133	0.0006018	0.006793		0.980	0.99	-0.12233	0.0701	0.0807784
rs9820920	3	182619785	C	T	0.978	1.00	-0.455	0.133	0.0005967	0.0067455		0.980	0.99	-0.1223	0.07011	0.0808496
rs10937057	3	182632756	A	G	0.022	1.00	0.446	0.133	0.0007904	0.0077611		0.020	0.99	0.120365	0.07036	0.0869195
rs16832504	3	182651393	A	G	0.022	1.00	0.449	0.133	0.0007482	0.0076059		0.020	0.98	0.116907	0.07088	0.0988203
rs11717567	3	182669053	C	T	0.978	0.99	-0.454	0.134	0.0007023	0.0072478		0.980	0.96	-0.11762	0.07139	0.0991931
rs9968130	3	187436307	G	T	0.961	0.99	0.263	0.1	0.008577	0.0005822	ETV5					
rs974385	3	187438757	C	T	0.961	1.00	0.277	0.1	0.005703	0.0003264	ETV5					
rs888383	3	187440328	C	T	0.961	1.00	0.278	0.1	0.005312	0.0003028	ETV5					
rs6793168	3	187447958	A	G	0.969	0.99	0.279	0.113	0.01361	0.0009563	ETV5					
rs12629364	3	187910876	A	C	0.023	0.54	0.586	0.181	0.001205	0.0001145	ETV5					
rs4012249	3	188262558	A	G	0.422	1.00	-0.13	0.039	0.0008728	0.0103737	ETV5					
rs4687002	3	190104562	C	T	0.741	1.00	0.097	0.044	0.02724	0.0008196		0.763	0.99	0.022592	0.02121	0.2864355
rs1355559	3	190104735	C	T	0.190	0.99	-0.111	0.049	0.02338	0.0007015		0.158	0.98	-0.04253	0.025	0.0886752
rs11706039	3	190673308	A	C	0.410	1.00	0.141	0.039	0.000347	0.0000113		0.406	0.87	-0.01931	0.01989	0.3312384
rs17504169	3	191585563	A	G	0.151	0.99	0.206	0.054	0.0001441	0.0000028		0.122	0.97	0.024768	0.02847	0.3838378
rs1425115	3	191587549	C	T	0.619	1.00	-0.104	0.04	0.009677	0.0008069		0.692	0.98	-0.00925	0.0199	0.6418729
rs16865430	3	191590503	G	T	0.381	1.00	0.104	0.04	0.009581	0.0007951		0.308	0.98	0.009513	0.0199	0.6322722
rs13098887	3	191591150	A	G	0.623	1.00	-0.107	0.04	0.00778	0.0005484		0.697	0.97	-0.006	0.02	0.7642025
rs1425116	3	191593119	A	C	0.847	0.99	-0.207	0.054	0.0001279	0.00000298		0.879	0.97	-0.02565	0.02849	0.3674889
rs7642120	3	191624899	A	C	0.230	1.00	0.137	0.046	0.002764	0.0008659		0.188	1.00	0.021099	0.02327	0.3641862
rs7651888	3	191628089	C	T	0.792	0.99	-0.156	0.048	0.001103	0.0001244		0.842	0.99	-0.01695	0.02491	0.4958562
rs6791513	3	191644059	C	T	0.159	1.00	0.169	0.053	0.001445	0.0000599		0.128	0.99	0.015911	0.0272	0.5582485
rs17446205	3	191645795	A	G	0.155	1.00	0.173	0.053	0.00115	0.0000411		0.125	0.99	0.008891	0.02787	0.7495068
rs10513852	3	191650227	C	T	0.845	1.00	-0.173	0.053	0.001159	0.0000414		0.875	0.99	-0.00884	0.02787	0.750818
rs17505919	3	191653697	A	G	0.841	1.00	-0.168	0.053	0.00146	0.0000603		0.872	0.99	-0.01591	0.02719	0.5580476
rs6793890	3	192364598	C	T	0.110	1.00	0.219	0.063	0.0004974	0.0065601		0.130	0.94	0.015068	0.02783	0.5879641
rs9818146	3	197126305	C	T	0.850	1.00	0.199	0.055	0.0002863	0.0017281		0.815	0.99	0.044894	0.02317	0.0524656
rs9866616	3	197204282	A	G	0.922	0.71	0.362	0.087	0.0000329	0.0001837		0.912	0.65	0.042546	0.03767	0.2583495
rs6797622	3	197219312	C	T	0.915	1.00	0.237	0.07	0.0007099	0.0017567		0.890	1.00	0.029047	0.02919	0.3193136
rs9837977	3	197230509	C	T	0.907	0.95	0.234	0.069	0.0006412	0.0024396		0.872	0.95	0.025107	0.02815	0.3719897
rs7683216	4	7473514	C	T	0.587	0.93	0.154	0.041	0.0001537	0.0019628		0.609	0.82	0.016688	0.02065	0.4185824
rs2867467	4	12934839	C	G	0.032	0.77	-0.482	0.129	0.0001842	0.0038108		0.023	0.74	-0.05893	0.0702	0.4007739
rs6842438	4	12937471	C	T	0.032	0.77	-0.482	0.129	0.000184	0.0038112		0.023	0.74	-0.05891	0.0702	0.4009431
rs10019897	4	12939193	C	T	0.969	0.77	0.485	0.129	0.000177	0.0036879		0.977	0.74	0.05834	0.07021	0.4056115
rs9996364	4	12942511	A	G	0.969	0.77	0.486	0.129	0.0001748	0.0036471		0.977	0.74	0.058199	0.07021	0.4067592
rs11946968	4	12943492	C	T	0.969	0.77	0.486	0.129	0.0001749	0.0036495		0.977	0.74	0.058227	0.07021	0.4065463
rs11945340	4	12947563	C	T	0.969	0.77	0.486	0.13	0.0001736	0.0036298		0.977	0.74	0.058247	0.07021	0.4063886
rs6839621	4	12949347	C	T	0.031	0.77	-0.487	0.13	0.0001727	0.0036168		0.023	0.74	-0.0582	0.07021	0.4067286
rs6837169	4	12953488	C	G	0.969	0.77	0.487	0.13	0.0001724	0.00361		0.977	0.74	0.058097	0.07021	0.407597
rs11930614	4	12955700	C	T	0.969	0.77	0.49	0.13	0.0001671	0.0035137		0.977	0.73	0.056544	0.07018	0.4200601
rs10021881	4	12960071	C	T	0.031	0.77	-0.49	0.13	0.000167	0.0035066		0.023	0.73	-0.05628	0.07018	0.4222116
rs16888596	4	12965491	A	G	0.031	0.77	-0.491	0.13	0.0001651	0.0034688		0.023	0.73	-0.0563	0.07018	0.4219899
rs11732938	4	12966372	A	G	0.969	0.77	0.492	0.13	0.0001639	0.0034237		0.977	0.73	0.056248	0.07017	0.4224178
rs10016671	4	12968145	C	T	0.970	0.78	0.492	0.131	0.0001633	0.0033823		0.977	0.73	0.056201	0.07017	0.4228066

					GOYA QC		GOYA Overweight/control			GOYA BMI continuous	known	IARC QC		IARC results		
MARKER	chr	position	A1	A2	FREQ1	Rsqr	Beta	SE	p	p	gene name	freq1	Rsqr	in_beta	in_SE	in_p
rs11947529	4	12969720	A	T	0.030	0.78	-0.492	0.131	0.0001633	0.0033832		0.023	0.73	-0.05618	0.07017	0.4229817
rs12331157	4	12973056	A	G	0.970	0.78	0.492	0.131	0.0001638	0.003387		0.977	0.73	0.055065	0.0701	0.4317978
rs12332023	4	12973072	C	T	0.970	0.78	0.492	0.131	0.0001641	0.0033944		0.977	0.73	0.054939	0.0701	0.4328037
rs7673680	4	12975579	C	T	0.970	0.78	0.492	0.131	0.0001641	0.0033924		0.977	0.73	0.054704	0.07007	0.4346263
rs10031623	4	12978422	C	T	0.970	0.78	0.492	0.131	0.000165	0.0034021		0.977	0.73	0.054671	0.07007	0.4348801
rs10003958	4	12979406	C	T	0.030	0.79	-0.492	0.13	0.0001649	0.0033896		0.023	0.73	-0.05467	0.07006	0.4348191
rs9999851	4	12982681	C	T	0.970	0.79	0.491	0.13	0.0001656	0.0033981		0.977	0.74	0.051595	0.06973	0.4589636
rs9291610	4	12983560	A	G	0.030	0.79	-0.491	0.13	0.0001653	0.0033982		0.023	0.74	-0.05156	0.06973	0.4592484
rs13435249	4	12988547	C	T	0.030	0.79	-0.492	0.131	0.0001681	0.0034323		0.023	0.74	-0.04989	0.06976	0.4741621
rs9993832	4	12998688	G	T	0.970	0.79	0.492	0.131	0.0001707	0.0034728		0.977	0.73	0.047896	0.07067	0.4975899
rs10022847	4	13000186	A	C	0.030	0.78	-0.493	0.131	0.0001727	0.0035019		0.023	0.73	-0.0478	0.07069	0.49854
rs10001262	4	13002981	C	T	0.030	0.78	-0.494	0.132	0.0001811	0.0036459		0.023	0.73	-0.04771	0.07072	0.4995323
rs9998914	4	13002995	A	T	0.970	0.78	0.495	0.132	0.0001814	0.0036556		0.977	0.73	0.047733	0.07075	0.4995186
rs9999092	4	13003140	A	G	0.970	0.78	0.495	0.132	0.0001819	0.0036638		0.977	0.73	0.047736	0.07077	0.4996358
rs6820841	4	13003890	A	G	0.030	0.78	-0.495	0.132	0.0001846	0.0037137		0.023	0.73	-0.04765	0.07082	0.5006738
rs16888633	4	13004522	C	T	0.970	0.78	0.496	0.133	0.0001853	0.0037277		0.977	0.73	0.047501	0.07088	0.5023639
rs9998670	4	13004995	A	G	0.030	0.78	-0.496	0.133	0.0001856	0.0037357		0.023	0.73	-0.04754	0.0709	0.5022077
rs11943295	4	13012953	A	G	0.029	0.77	-0.497	0.134	0.0001967	0.0039218		0.022	0.71	-0.04266	0.07297	0.5584156
rs11943330	4	13013096	A	G	0.029	0.77	-0.498	0.134	0.0001976	0.0039418		0.022	0.71	-0.04243	0.07304	0.5609616
rs7677336	4	13013228	G	T	0.971	0.77	0.499	0.134	0.000201	0.0039965		0.978	0.71	0.042357	0.07306	0.5617461
rs7673732	4	13013815	A	C	0.972	0.75	0.505	0.138	0.0002614	0.0049707		0.978	0.71	0.042305	0.07309	0.5623934
rs11737264	4	13049129	C	G	0.972	0.75	0.505	0.139	0.0002686	0.0050776		0.978	0.71	0.041381	0.07337	0.5724429
rs11737360	4	13049369	C	T	0.972	0.75	0.505	0.139	0.0002693	0.0050917		0.978	0.71	0.041373	0.0734	0.5726444
rs16888654	4	13058630	A	C	0.972	0.75	0.505	0.139	0.0002711	0.0051238		0.978	0.71	0.041276	0.07341	0.5735897
rs16888661	4	13061120	A	C	0.028	0.75	-0.506	0.139	0.000273	0.0051452		0.022	0.71	-0.04117	0.07345	0.574772
rs11933841	4	13073089	C	T	0.028	0.74	-0.507	0.14	0.0002855	0.0053473		0.022	0.70	-0.04065	0.07375	0.5811812
rs11947665	4	13074808	A	T	0.028	0.74	-0.507	0.14	0.0002883	0.0053836		0.022	0.70	-0.04059	0.0738	0.581976
rs10034452	4	13139922	G	T	0.157	0.99	-0.199	0.054	0.0002181	0.0003256		0.131	0.99	-0.04392	0.02663	0.0987962
rs2031007	4	16995648	A	G	0.726	0.73	-0.168	0.051	0.0009492	0.0014571		0.747	0.66	-0.01271	0.02502	0.6111843
rs6821103	4	16998305	C	T	0.726	0.73	-0.168	0.051	0.0009273	0.0014308		0.747	0.66	-0.01278	0.02501	0.6090176
rs1491385	4	23354888	A	G	0.169	1.00	0.122	0.051	0.01788	0.0009973		0.198	1.00	0.020443	0.0227	0.3674066
rs16879040	4	26850658	A	G	0.010	0.98	-0.579	0.202	0.004095	0.0007743		0.013	0.90	0.071473	0.08509	0.4005578
rs7678873	4	26852884	A	T	0.990	0.98	0.579	0.202	0.004077	0.0007637		0.987	0.90	-0.07157	0.08509	0.3999226
rs7678355	4	26858638	A	C	0.990	0.98	0.583	0.202	0.003945	0.0006814		0.987	0.89	-0.072	0.08513	0.3973266
rs6848537	4	28112147	A	G	0.820	0.94	-0.168	0.052	0.001163	0.0004906		0.824	0.90	-0.00822	0.02508	0.7428598
rs9306948	4	37080714	A	G	0.236	1.00	-0.133	0.045	0.003307	0.0006325		0.231	0.88	-0.01424	0.02309	0.5369222
rs1479772	4	37102099	C	G	0.764	0.98	0.126	0.046	0.006079	0.0009336		0.777	0.94	0.008205	0.0225	0.7151175
rs13122883	4	37378454	G	T	0.963	0.68	-0.442	0.126	0.0004343	0.0006922		0.966	0.51	0.024306	0.0712	0.732603
rs9991419	4	40177953	C	T	0.373	0.94	0.144	0.041	0.0004731	0.000095		0.375	0.90	-0.00353	0.02001	0.8597242
rs4861170	4	40185222	C	T	0.454	0.98	0.119	0.039	0.002372	0.0008985		0.457	0.97	-0.00715	0.01872	0.702373
rs1106390	4	41574726	A	T	0.805	0.91	-0.176	0.051	0.0005604	0.0056292		0.760	0.87	0.025133	0.02295	0.2731498
rs4861148	4	41577358	C	T	0.229	0.96	0.163	0.047	0.0004936	0.0060472		0.278	0.92	-0.01875	0.02143	0.3812237
rs9993759	4	41578240	C	T	0.227	1.00	0.163	0.046	0.000436	0.0056232		0.279	1.00	-0.01921	0.02061	0.3508291
rs7675823	4	41593665	C	G	0.879	1.00	-0.199	0.06	0.0009125	0.0089066		0.847	1.00	0.006021	0.02523	0.8112375
rs13122139	4	41626538	A	G	0.898	1.00	-0.237	0.065	0.000241	0.0044845		0.889	1.00	-0.023	0.02908	0.4286104
rs17445354	4	41659094	A	G	0.890	0.94	-0.216	0.064	0.000789	0.0133142		0.877	0.94	-0.01221	0.02892	0.6727519
rs2660343	4	41672523	A	G	0.785	0.96	-0.161	0.048	0.000889	0.0082881		0.730	0.95	0.014565	0.0213	0.4936933
rs10938217	4	42471899	G	T	0.130	0.93	-0.241	0.06	0.0000526	0.000264		0.115	0.94	0.028789	0.02888	0.3184801
rs10938218	4	42473969	C	T	0.870	0.95	0.238	0.059	0.0000541	0.0002648		0.885	0.94	-0.02879	0.02887	0.3182988
rs12642750	4	42481358	C	T	0.871	0.96	0.238	0.059	0.0000547	0.0002659		0.885	0.94	-0.02871	0.02881	0.3187394
rs2575555	4	42481914	A	T	0.767	0.99	-0.176	0.045	0.0001059	0.0002146		0.818	0.97	-0.00249	0.02392	0.9170744
rs12331684	4	42484887	A	G	0.135	1.00	-0.222	0.057	0.0000904	0.0002693		0.134	0.98	0.021623	0.02667	0.4172064
rs6856377	4	42485271	C	T	0.135	1.00	-0.222	0.057	0.000089	0.0002653		0.134	0.98	0.021623	0.02667	0.4170963
rs12506552	4	42486583	C	T	0.135	1.00	-0.222	0.057	0.000088	0.0002623		0.134	0.98	0.021627	0.02666	0.4169005
rs12640374	4	42490867	G	T	0.865	1.00	0.222	0.057	0.0000876	0.000261		0.866	0.99	-0.02161	0.02665	0.417153
rs12650389	4	42490884	C	T	0.135	1.00	-0.222	0.057	0.0000867	0.0002584		0.134	0.99	0.021617	0.02664	0.4166631
rs12640399	4	42490920	C	G	0.138	0.98	-0.222	0.057	0.0000939	0.0002791		0.136	0.97	0.019084	0.02678	0.4756701
rs12640422	4	42490992	A	G	0.135	1.00	-0.222	0.057	0.0000866	0.0002582		0.134	0.99	0.021633	0.02661	0.4159083
rs12640425	4	42491040	C	G	0.135	1.00	-0.222	0.057	0.0000865	0.0002581		0.134	0.99	0.021657	0.02661	0.4152701
rs2575513	4	42491051	C	T	0.232	1.00	0.174	0.045	0.000123	0.0002544		0.180	1.00	0.003118	0.02378	0.8955768
rs12650499	4	42491265	A	T	0.135	1.00	-0.222	0.057	0.0000865	0.000258		0.134	1.00	0.021616	0.02645	0.4134912
rs12650501	4	42491278	C	T	0.135	1.00	-0.222	0.057	0.0000865	0.0002579		0.134	1.00	0.021627	0.02645	0.413189
rs17637428	4	42491847	C	T	0.135	1.00	-0.222	0.057	0.0000864	0.0002578		0.134	1.00	0.021622	0.02644	0.4131151
rs7697036	4	42492031	C	T	0.207	0.79	-0.184	0.054	0.0006097	0.0027552		0.194	0.80	0.021707	0.02562	0.3964695
rs2598306	4	42494347	A	G	0.232	1.00	0.174	0.045	0.0001241	0.0002584		0.180	1.00	0.003086	0.02376	0.8965725
rs2575515	4	42495213	G	T	0.232	1.00	0.174	0.045	0.0001237	0.0002575		0.180	1.00	0.003148	0.02376	0.8945254
rs1901224	4	42496766	G	T	0.232	1.00	0.174	0.045	0.0001226	0.000255		0.180	1.00	0.003245	0.02377	0.8913316
rs12505784	4	42500358	C	T	0.870	1.00	0.233	0.058	0.0000554	0.0002512		0.885	0.99	-0.02675	0.02808	0.3403079
rs12644472	4	42503847	C	T	0.865	1.00	0.222	0.057	0.0000862	0.0002576		0.866	1.00	-0.02163	0.02643	0.4126966
rs6831242	4	42504025	C	T	0.135	1.00	-0.222	0.057	0.0000862	0.0002576		0.134	1.00	0.021634	0.02643	0.4126725
rs17637488	4	42504877	A	G	0.866	1.00	0.224	0.057	0.0000791	0.00024		0.866	0.99	-0.022	0.02661	0.4080079
rs1562838	4	42508479	A	G	0.773	1.00	-0.187	0.046	0.0000446	0.0001253		0.822	1.00	-0.01749	0.02405	0.466842
rs1562839	4	42508557	A	G	0.773	1.00	-0.187	0.046	0.0000443	0.0001246		0.822	1.00	-0.01753	0.02405	0.4657563
rs1450913	4	42508647	A	G	0.253	1.00	-0.163	0.045	0.0002503	0.0076633		0.253	1.00	0.003292	0.02102	0.8754428
rs4861224	4	42510046	A	T	0.227	1.00	0.187	0.046	0.0000455	0.0001291		0.178	1.00	0.017607	0.02406	0.463861
rs12642002	4	42516252	G	T	0.265	0.96	-0.19	0.045	0.0000216	0.0016312		0.260	0.96	0.00288	0.02127	0.8921869
rs13101582	4	42517936	A	G	0.265	0.96	-0.19	0.045	0.0000219	0.0016547		0.260	0.95	0.002868	0.02129	0.8927634
rs13142647	4	44713340	A	G	0.825	1.00	0.136	0.051	0.007576	0.0009474	GNPDA2					
rs13144044	4	44776993	C	G	0.548	1.00	-0.164	0.039	0.0000216	0.0000237	GNPDA2					
rs959904	4	44797386	C	T	0.472	1.00	-0.13	0.039	0.0007753	0.0012225	GNPDA2					
rs1473819	4	44798779	A	G	0.415	1.00	-0.116	0.039	0.003111	0.0008252	GNPDA2					
rs4541546	4	44800748	A	G	0.415	1.00	-0.116	0.039	0.003091	0.0008252	GNPDA2					

					GOYA QC		GOYA Overweight/control			GOYA BMI continuous	known	IARC QC		IARC results		
MARKER	chr	position	A1	A2	FREQ1	Rsqr	Beta	SE	p	p	gene name	freq1	Rsqr	in_beta	in_SE	in_p
rs10008064	4	44808022	A	G	0.585	1.00	0.116	0.039	0.003083	0.0008281	GNPDA2					
rs10011163	4	44808697	C	T	0.402	0.98	-0.118	0.04	0.003002	0.0006166	GNPDA2					
rs2203165	4	44810892	A	G	0.585	1.00	0.116	0.039	0.003075	0.0008309	GNPDA2					
rs2203166	4	44810954	C	T	0.415	1.00	-0.116	0.039	0.003072	0.0008318	GNPDA2					
rs969992	4	44812591	C	T	0.415	1.00	-0.116	0.039	0.003077	0.000833	GNPDA2					
rs9999372	4	44814649	C	T	0.585	1.00	0.116	0.039	0.003057	0.0008293	GNPDA2					
rs12504429	4	44817768	C	T	0.474	1.00	-0.134	0.039	0.0005539	0.0010658	GNPDA2					
rs1913018	4	44820085	A	G	0.474	1.00	-0.134	0.039	0.0005553	0.0010668	GNPDA2					
rs12650122	4	44820260	A	G	0.474	1.00	-0.134	0.039	0.0005556	0.0010672	GNPDA2					
rs10032137	4	44823084	C	G	0.415	1.00	-0.116	0.039	0.00307	0.0008314	GNPDA2					
rs1512312	4	44825231	A	G	0.474	1.00	-0.134	0.039	0.0005559	0.0010675	GNPDA2					
rs12512251	4	44826318	A	G	0.474	1.00	-0.134	0.039	0.0005559	0.0010675	GNPDA2					
rs6845132	4	44834430	C	T	0.474	1.00	-0.134	0.039	0.0005538	0.0010279	GNPDA2					
rs1849338	4	44837562	A	G	0.548	1.00	-0.167	0.039	0.0000146	0.000019	GNPDA2					
rs7654230	4	44851770	C	T	0.535	1.00	0.135	0.039	0.000486	0.0009384	GNPDA2					
rs1996023	4	44859394	G	T	0.416	0.98	-0.119	0.04	0.002917	0.0005598	GNPDA2					
rs13130484	4	44870448	C	T	0.577	1.00	-0.168	0.039	0.0000187	0.00000157	GNPDA2					
rs16858082	4	44870561	C	T	0.416	0.98	-0.118	0.04	0.003062	0.0005815	GNPDA2					
rs12641981	4	44874640	C	T	0.576	1.00	-0.167	0.039	0.0000206	0.0000018	GNPDA2					
rs1581095	4	44874954	A	C	0.585	0.98	0.117	0.04	0.003453	0.0006943	GNPDA2					
rs10938397	4	44877284	A	G	0.575	0.99	-0.168	0.039	0.0000216	0.00000201	GNPDA2					
rs348495	4	44879199	A	G	0.412	0.99	-0.114	0.04	0.0042	0.0009435	GNPDA2					
rs13104545	4	44879664	A	G	0.204	0.73	0.204	0.056	0.0002814	0.0000264	GNPDA2					
rs2253189	4	52631342	A	C	0.620	0.95	-0.148	0.041	0.0003061	0.0007182		0.599	0.95	0.013083	0.0192	0.4953428
rs2680907	4	52631681	A	T	0.330	0.99	0.145	0.041	0.0004517	0.0019608		0.361	0.99	-0.00604	0.01921	0.7531325
rs3860707	4	52633000	C	T	0.665	0.97	-0.144	0.041	0.0005252	0.0021536		0.638	1.00	0.005533	0.01912	0.7720699
rs11727580	4	52642875	A	G	0.670	1.00	-0.145	0.041	0.0003986	0.0017942		0.639	1.00	0.005832	0.01917	0.7607341
rs7696028	4	52645417	A	C	0.330	1.00	0.145	0.041	0.0003979	0.0017921		0.362	1.00	-0.00583	0.01917	0.7609239
rs10866420	4	52651772	A	G	0.670	1.00	-0.145	0.041	0.0003965	0.0017881		0.639	1.00	0.005819	0.01917	0.7612883
rs3214049	4	52655665	A	T	0.670	1.00	-0.146	0.041	0.0003925	0.0017772		0.639	1.00	0.005805	0.01918	0.7619223
rs6817395	4	52661730	A	G	0.671	1.00	-0.147	0.041	0.0003379	0.0015358		0.644	0.99	0.006633	0.01935	0.7316127
rs11722603	4	52664489	A	G	0.372	1.00	0.132	0.04	0.0008814	0.002003		0.394	0.98	-0.00681	0.01912	0.7214433
rs4146562	4	52666776	C	T	0.673	0.99	-0.15	0.041	0.0002707	0.0011338		0.644	0.99	0.006567	0.01936	0.7342156
rs7682221	4	52667032	C	T	0.327	0.99	0.15	0.041	0.0002652	0.0010993		0.356	0.99	-0.00655	0.01936	0.7348654
rs11133357	4	52678143	A	G	0.669	0.98	-0.155	0.041	0.0001879	0.0007295		0.639	0.97	0.008582	0.01943	0.6584147
rs4359971	4	52688952	C	T	0.632	0.99	-0.135	0.04	0.0007484	0.0015075		0.608	0.98	0.006015	0.01914	0.7531729
rs7699031	4	52690159	C	G	0.632	0.99	-0.134	0.04	0.0007679	0.0015379		0.608	0.98	0.005968	0.01914	0.7550545
rs13128128	4	52694912	A	C	0.365	0.99	0.132	0.04	0.0009506	0.0018182		0.391	0.98	-0.00536	0.01916	0.7794905
rs1460556	4	52696062	A	T	0.635	0.99	-0.132	0.04	0.0009533	0.0018206		0.609	0.98	0.005277	0.01916	0.7827778
rs7699248	4	52763795	A	T	0.593	0.93	-0.134	0.041	0.000971	0.0021341		0.592	0.89	0.007843	0.0198	0.6917951
rs2412862	4	58555675	A	C	0.804	1.00	-0.167	0.05	0.0007848	0.0024391		0.832	0.99	0.013413	0.02394	0.5749112
rs2054261	4	58563471	A	C	0.803	1.00	-0.168	0.05	0.0007044	0.0024519		0.832	0.99	0.013049	0.02393	0.5852423
rs17290134	4	58601386	A	G	0.803	1.00	-0.165	0.05	0.0008542	0.0024088		0.833	1.00	0.009093	0.02391	0.7035326
rs12508002	4	59057875	C	T	0.983	0.65	0.72	0.197	0.0002574	0.0046467		0.978	0.60	0.009158	0.09742	0.9250391
rs13134853	4	59291516	C	T	0.985	0.96	0.574	0.169	0.0006616	0.005391		0.983	0.91	-0.03729	0.09253	0.6866764
rs12503260	4	59294173	G	T	0.015	0.96	-0.574	0.169	0.0006621	0.0053958		0.017	0.91	0.037376	0.09252	0.6859735
rs11732112	4	61086564	C	T	0.816	0.96	0.183	0.051	0.0003589	0.0188687		0.839	0.94	-0.01365	0.02549	0.5920321
rs12509655	4	62578623	A	T	0.459	0.97	0.122	0.039	0.001895	0.0006799		0.492	0.94	0.000945	0.01881	0.959887
rs2171386	4	68112443	C	T	0.736	0.98	-0.146	0.044	0.001043	0.0007864		0.797	0.97	-0.01863	0.0227	0.4112885
rs13132585	4	68114811	A	G	0.264	0.98	0.146	0.044	0.001029	0.0007798		0.203	0.97	0.018479	0.02268	0.4149112
rs11731488	4	68120448	A	G	0.738	1.00	-0.151	0.044	0.0006714	0.0005815		0.796	0.99	-0.01609	0.02241	0.4724548
rs11725922	4	68126787	A	G	0.263	1.00	0.152	0.044	0.0005935	0.000542		0.204	1.00	0.015022	0.02228	0.4997059
rs17631747	4	68128907	C	T	0.737	1.00	-0.152	0.044	0.0005883	0.0005305		0.795	1.00	-0.01511	0.02228	0.4973843
rs12507679	4	68129635	C	T	0.737	1.00	-0.152	0.044	0.0005814	0.0005174		0.794	0.99	-0.01543	0.02224	0.4874821
rs931360	4	68135468	C	G	0.263	1.00	0.153	0.044	0.0005649	0.000477		0.207	0.99	0.015586	0.02224	0.4830289
rs3821982	4	77314814	C	T	0.138	0.89	-0.179	0.059	0.002434	0.0009613		0.130	0.88	-0.01772	0.02792	0.5254166
rs894251	4	77348864	A	G	0.137	0.97	-0.19	0.057	0.0008299	0.0001754		0.120	0.96	-0.02736	0.0273	0.3159111
rs6817593	4	77680035	C	T	0.430	1.00	0.131	0.039	0.0008267	0.001354		0.497	0.99	0.032193	0.01826	0.0776693
rs17002080	4	77683548	A	G	0.432	0.99	0.129	0.039	0.0009716	0.001411		0.497	0.98	0.033461	0.01839	0.0686336
rs6532475	4	77692853	A	G	0.579	0.92	-0.135	0.04	0.0008055	0.0010788		0.584	0.88	-0.01893	0.01969	0.3358246
rs1584984	4	80279231	A	G	0.295	1.00	-0.144	0.043	0.0007852	0.0016616		0.297	0.99	0.02508	0.02013	0.2124459
rs11733517	4	80281253	A	G	0.705	1.00	0.144	0.043	0.0007786	0.0016516		0.703	0.99	-0.02511	0.02012	0.2116506
rs1037589	4	80286091	G	T	0.705	1.00	0.144	0.043	0.0007762	0.001648		0.703	0.99	-0.02517	0.02011	0.2103305
rs4975160	4	80290650	C	T	0.295	1.00	-0.145	0.043	0.0007692	0.0016375		0.298	0.99	0.025215	0.02009	0.2091601
rs10518214	4	80304966	C	T	0.174	1.00	-0.175	0.051	0.0006011	0.0006158		0.207	0.99	-0.0007	0.02268	0.9753466
rs9790513	4	80305472	A	C	0.170	1.00	-0.183	0.052	0.0003917	0.0005556		0.197	0.98	-0.00917	0.02311	0.6911864
rs2010696	4	80327883	C	G	0.154	0.95	-0.184	0.056	0.0009277	0.0006592		0.174	0.92	-0.02046	0.02504	0.4135733
rs1431559	4	90454511	A	G	0.165	0.72	0.204	0.062	0.0009758	0.0007732		0.175	0.67	0.008385	0.03014	0.7806342
rs7664724	4	90460506	A	G	0.816	0.98	-0.172	0.051	0.0006744	0.0003398		0.802	0.98	0.025026	0.02361	0.2888122
rs4106153	4	90463499	A	C	0.816	1.00	-0.17	0.05	0.0007168	0.0003716		0.801	1.00	0.024922	0.0234	0.2864885
rs10002808	4	90477425	C	G	0.815	0.99	-0.167	0.05	0.0008736	0.0003978		0.805	0.99	0.025572	0.02368	0.279757
rs6831965	4	93618705	A	G	0.051	1.00	0.255	0.089	0.003932	0.000893		0.049	0.76	-0.11472	0.04942	0.0201839
rs6846665	4	96116392	A	G	0.963	0.62	-0.333	0.13	0.01049	0.0007966		0.964	0.77	0.05141	0.05101	0.3131579
rs1023804	4	96117322	A	G	0.972	0.90	-0.306	0.125	0.01429	0.0006209		0.964	0.80	0.050728	0.05043	0.3140992
rs1023803	4	96117426	C	T	0.026	1.00	0.291	0.121	0.01665	0.0007401		0.036	0.81	-0.05055	0.05027	0.3142091
rs1023802	4	96117548	C	G	0.967	0.82	-0.318	0.12	0.007919	0.0002229		0.959	0.72	0.029137	0.04973	0.5575873
rs1318972	4	96117678	C	T	0.026	1.00	0.29	0.121	0.01675	0.0007433		0.036	0.81	-0.05054	0.05024	0.3140634
rs1023801	4	96118146	G	T	0.026	1.00	0.289	0.121	0.01712	0.0007553		0.036	0.81	-0.05042	0.0501	0.3137962
rs1023800	4	96118181	A	T	0.974	1.00	-0.288	0.121	0.01747	0.0007685		0.965	0.83	0.050132	0.0498	0.3137485
rs2099996	4	96119448	A	G	0.974	1.00	-0.288	0.121	0.01755	0.0007709		0.965	0.88	0.048974	0.04893	0.3164964
rs920912	4	96119704	A	G	0.027	1.00	0.287	0.121	0.0179	0.0007832		0.035	0.88	-0.04896	0.04893	0.3165543
rs12507710	4	96120361	A	T	0.974	1.00	-0.287	0.121	0.01802	0.0007876		0.965	0.89	0.048786	0.04878	0.3168681

					GOYA QC		GOYA Overweight/control			GOYA BMI continuous	known	IARC QC		IARC results		
MARKER	chr	position	A1	A2	FREQ1	Rsqr	Beta	SE	p	p	gene name	freq1	Rsqr	in_beta	in_SE	in_p
rs2028761	4	96121200	A	G	0.027	1.00	0.286	0.121	0.01827	0.000796		0.035	0.89	-0.04875	0.04873	0.3167691
rs13145631	4	96121407	A	T	0.027	1.00	0.286	0.121	0.01828	0.0007964		0.035	0.89	-0.04876	0.04872	0.3165205
rs6532520	4	96121889	A	T	0.974	1.00	-0.286	0.121	0.01841	0.0008007		0.966	0.90	0.048532	0.04854	0.3169841
rs3755874	4	96122001	C	T	0.027	1.00	0.286	0.121	0.01847	0.0008028		0.035	0.90	-0.04855	0.04852	0.3165568
rs1530459	4	96122686	A	G	0.027	1.00	0.286	0.121	0.01851	0.0008041		0.035	0.90	-0.04856	0.04851	0.3164144
rs2044328	4	96123847	A	G	0.027	1.00	0.285	0.121	0.01853	0.0008049		0.034	0.93	-0.04814	0.04816	0.3171073
rs3775033	4	96124806	C	T	0.974	1.00	-0.285	0.121	0.01864	0.0008092		0.966	0.93	0.04804	0.04809	0.3174257
rs3755875	4	96126469	C	T	0.027	1.00	0.285	0.121	0.01869	0.0008111		0.034	0.95	-0.04754	0.04776	0.3191519
rs965447	4	96128305	G	T	0.974	1.00	-0.285	0.121	0.0187	0.0008115		0.966	0.96	0.047561	0.04772	0.3185003
rs1838501	4	96128638	A	T	0.974	1.00	-0.285	0.121	0.01872	0.000812		0.966	0.96	0.04754	0.04771	0.3186472
rs1867542	4	96129981	C	T	0.974	1.00	-0.285	0.121	0.01874	0.0008125		0.966	0.96	0.04758	0.0477	0.3181196
rs1545329	4	96131913	C	T	0.027	1.00	0.283	0.121	0.01951	0.00084		0.034	0.96	-0.04753	0.04768	0.3184668
rs1545327	4	96133421	A	G	0.974	1.00	-0.283	0.121	0.01955	0.0008417		0.966	0.96	0.047508	0.04766	0.3184431
rs716190	4	96135811	A	G	0.027	1.00	0.283	0.121	0.01964	0.0008439		0.034	0.98	-0.04719	0.04735	0.3185874
rs6836504	4	96137788	C	G	0.974	1.00	-0.283	0.121	0.01965	0.0008441		0.966	0.98	0.04715	0.04734	0.3188575
rs6843668	4	96139108	C	T	0.027	1.00	0.283	0.121	0.01965	0.0008441		0.034	0.98	-0.04713	0.04733	0.3190395
rs11097454	4	96140065	G	T	0.027	1.00	0.282	0.121	0.01966	0.0008446		0.034	0.98	-0.04709	0.04732	0.3192667
rs7698787	4	96140839	A	G	0.974	1.00	-0.282	0.121	0.01967	0.000845		0.966	0.98	0.047077	0.04731	0.3193482
rs2120832	4	96143930	C	G	0.027	1.00	0.282	0.121	0.01969	0.0008462		0.034	0.98	-0.04701	0.04727	0.319504
rs1444920	4	96145017	A	G	0.027	1.00	0.282	0.121	0.01971	0.0008469		0.034	0.99	-0.04702	0.04725	0.3193555
rs4699828	4	96150126	C	T	0.027	1.00	0.282	0.121	0.01971	0.000847		0.034	0.99	-0.04701	0.04724	0.319318
rs973887	4	96151546	A	G	0.027	1.00	0.282	0.121	0.01971	0.000847		0.034	0.99	-0.04687	0.04714	0.3197079
rs1347346	4	96162091	C	T	0.026	1.00	0.282	0.121	0.02017	0.0008717		0.034	0.99	-0.04696	0.04715	0.3189074
rs6815448	4	96164739	C	T	0.026	1.00	0.282	0.121	0.02032	0.0008803		0.033	0.99	-0.04696	0.04716	0.3190024
rs6844029	4	96169194	A	G	0.974	1.00	-0.279	0.122	0.0219	0.0009786		0.967	1.00	0.04675	0.04719	0.3214009
rs1160695	4	96171888	A	G	0.026	1.00	0.279	0.122	0.02198	0.0009836		0.033	1.00	-0.04675	0.04719	0.3214211
rs2525795	4	98127927	A	C	0.413	1.00	-0.131	0.04	0.0009855	0.0017757		0.478	0.91	0.035115	0.01904	0.0649964
rs1567287	4	98728153	C	T	0.416	1.00	-0.131	0.039	0.0008347	0.0001966		0.402	1.00	0.001111	0.01849	0.9520645
rs1508466	4	98731082	A	G	0.416	1.00	-0.131	0.039	0.000826	0.0001956		0.402	1.00	0.001093	0.01849	0.9528358
rs12649979	4	98732951	A	G	0.416	1.00	-0.131	0.039	0.0008134	0.0001942		0.402	1.00	0.00107	0.01849	0.9538268
rs13149481	4	98739355	A	G	0.416	1.00	-0.131	0.039	0.000809	0.0001939		0.402	1.00	0.001065	0.01848	0.9540052
rs1355044	4	98746509	A	C	0.584	1.00	0.131	0.039	0.000807	0.0001949		0.598	1.00	-0.00117	0.01848	0.9496465
rs2865799	4	98750698	C	G	0.584	1.00	0.131	0.039	0.0008065	0.0001955		0.598	1.00	-0.00121	0.01848	0.9478152
rs4568246	4	98759866	C	T	0.416	1.00	-0.131	0.039	0.0008063	0.0001958		0.402	1.00	0.001232	0.01848	0.9467968
rs1605637	4	98762235	A	T	0.416	1.00	-0.131	0.039	0.0008061	0.0001961		0.402	1.00	0.00125	0.01848	0.9460455
rs2136081	4	98766250	C	T	0.416	1.00	-0.131	0.039	0.0008061	0.0001962		0.402	1.00	0.001267	0.01848	0.9452799
rs4153	4	98767243	C	T	0.584	1.00	0.131	0.039	0.0008063	0.0001966		0.598	1.00	-0.00133	0.01848	0.9424627
rs1508475	4	98768385	A	G	0.582	1.00	0.129	0.039	0.0009745	0.0002421		0.598	1.00	-0.00137	0.01849	0.9410715
rs2659528	4	102376308	A	C	0.195	0.89	-0.162	0.052	0.001928	0.000585		0.235	0.91	-0.01364	0.02244	0.5430294
rs236768	4	103295006	A	G	0.824	1.00	0.115	0.05	0.0221	0.0007935		0.819	1.00	0.022425	0.02435	0.3567363
rs236770	4	103298868	A	G	0.824	1.00	0.115	0.05	0.02224	0.0007967		0.819	0.99	0.022408	0.0244	0.3579667
rs236781	4	103303597	A	G	0.833	0.97	0.111	0.052	0.03141	0.0009193		0.823	0.93	0.024165	0.02533	0.339664
rs10034263	4	107720790	A	G	0.145	0.87	0.195	0.059	0.0008688	0.0004722		0.136	0.92	0.016018	0.02798	0.5666872
rs17036903	4	107799561	C	T	0.783	0.86	-0.178	0.051	0.0004468	0.000833		0.831	0.78	0.011505	0.02751	0.6755696
rs6831071	4	113671486	C	G	0.854	1.00	0.185	0.055	0.0007567	0.0005075		0.826	1.00	0.02437	0.0239	0.3075472
rs11098163	4	113676967	A	G	0.854	1.00	0.185	0.055	0.0007846	0.0005379		0.826	1.00	0.024124	0.02388	0.3120735
rs2306775	4	113680376	A	C	0.123	1.00	-0.2	0.059	0.0007195	0.0002863		0.146	0.99	-0.01675	0.02577	0.5154305
rs12054589	4	113685420	A	G	0.847	0.98	0.181	0.054	0.0008692	0.0004941		0.822	0.99	0.027717	0.0239	0.2457314
rs1486863	4	113710475	C	T	0.119	0.99	-0.215	0.06	0.0003814	0.0001167		0.146	0.99	-0.01896	0.02585	0.4628085
rs12503100	4	113712427	G	T	0.881	0.99	0.215	0.06	0.0003765	0.0001142		0.855	0.99	0.019144	0.02585	0.4585347
rs10031880	4	113730034	C	G	0.119	0.99	-0.215	0.06	0.0003741	0.0001105		0.146	0.99	-0.01925	0.02585	0.4560677
rs11724428	4	113739056	A	G	0.115	0.93	-0.165	0.063	0.009257	0.0009175		0.145	0.94	-0.02899	0.02658	0.2750413
rs9993534	4	113753245	C	T	0.120	0.98	-0.214	0.06	0.0004036	0.0001067		0.148	0.98	-0.01962	0.02558	0.4427182
rs6533631	4	113797715	A	G	0.094	0.97	-0.234	0.068	0.0006056	0.0005805		0.098	0.96	-0.03591	0.02963	0.2250855
rs1129065	4	113798058	C	T	0.873	1.00	0.202	0.058	0.000525	0.0001238		0.856	0.98	0.023703	0.02543	0.3509822
rs6810996	4	113804390	A	G	0.893	0.88	0.243	0.067	0.0003006	0.0001247		0.876	0.93	0.024404	0.02757	0.3756628
rs7692994	4	120646937	A	C	0.345	0.99	0.136	0.041	0.0009678	0.0096387		0.311	0.98	-0.04193	0.0198	0.0340977
rs2389882	4	120786181	A	C	0.708	0.98	-0.145	0.043	0.0007433	0.0045791		0.723	0.98	0.06082	0.02063	0.003187
rs2389886	4	120789870	A	G	0.292	0.98	0.145	0.043	0.0007209	0.0045224		0.277	0.98	-0.06087	0.02063	0.0031611
rs2389887	4	120790092	C	T	0.292	0.98	0.145	0.043	0.0007194	0.0045178		0.277	0.98	-0.06089	0.02063	0.0031508
rs10022185	4	120791213	C	G	0.708	0.98	-0.145	0.043	0.0007162	0.0045091		0.723	0.98	0.060991	0.02063	0.0030971
rs7664440	4	120797988	A	G	0.708	0.98	-0.147	0.043	0.0006334	0.00432		0.722	0.98	0.061638	0.02057	0.0027173
rs12642411	4	120799973	C	T	0.291	0.99	0.146	0.043	0.0006489	0.0043604		0.276	0.99	-0.06078	0.02051	0.0030285
rs17051356	4	120804756	C	T	0.710	1.00	-0.146	0.043	0.000611	0.0043254		0.724	0.99	0.060762	0.02051	0.0030304
rs17051206	4	121806206	C	G	0.978	0.99	-0.443	0.132	0.0008115	0.0003991		0.980	0.92	-0.07516	0.06305	0.2329201
rs1390559	4	121829939	A	G	0.978	0.99	-0.444	0.132	0.0007845	0.0004058		0.980	0.91	-0.07422	0.06356	0.2425952
rs2390228	4	122881704	A	G	0.036	0.43	0.569	0.161	0.00041	0.0000759		0.059	0.45	0.008433	0.06141	0.8906728
rs3097920	4	124942803	C	T	0.587	1.00	0.11	0.039	0.004819	0.0004365		0.614	0.91	0.018159	0.01958	0.3533327
rs3097917	4	124947002	A	G	0.408	0.99	-0.115	0.039	0.003355	0.0002808		0.393	0.94	-0.01371	0.01928	0.4765191
rs2390587	4	124950752	C	T	0.467	1.00	0.106	0.039	0.00617	0.0008919		0.474	1.00	0.015369	0.01822	0.3986631
rs3113385	4	124955485	C	T	0.423	1.00	-0.109	0.039	0.005233	0.0002808		0.421	0.99	-0.01184	0.01868	0.5260206
rs3097913	4	124955556	A	G	0.577	1.00	0.109	0.039	0.005167	0.0002778		0.579	0.99	0.011843	0.01869	0.5258688
rs721456	4	124955942	C	T	0.423	1.00	-0.109	0.039	0.005041	0.0002721		0.421	0.98	-0.01188	0.01871	0.524884
rs1991839	4	124964497	A	C	0.580	1.00	0.108	0.039	0.005494	0.0003195		0.579	0.94	0.013232	0.01919	0.4901436
rs978534	4	124968827	G	T	0.580	1.00	0.109	0.039	0.005163	0.0002946		0.580	0.94	0.013356	0.0192	0.4863897
rs1016592	4	124971364	A	G	0.580	0.99	0.109	0.039	0.00511	0.0002904		0.580	0.94	0.013351	0.0192	0.4865508
rs1398927	4	139487508	A	G	0.269	0.99	0.145	0.044	0.0009618	0.0041274		0.272	0.86	-0.02818	0.02219	0.2038674
rs11930778	4	142065139	C	T	0.067	1.00	0.276	0.078	0.0004148	0.0002737		0.058	0.96	0.028533	0.03737	0.4448029
rs17339131	4	142067650	C	T	0.067	1.00	0.276	0.078	0.0004034	0.0002797		0.059	0.98	0.028483	0.03694	0.4402894
rs17423804	4	142071229	C	T	0.933	1.00	-0.271	0.078	0.0004956	0.0003866		0.941	0.99	-0.02876	0.03686	0.434891
rs6537022	4	142072132	A	G	0.058	0.97	0.344	0.085	0.0000521	0.0000177		0.056	0.95	0.021069	0.03899	0.5886076
rs7658326	4	142072890	A	G	0.910	0.98	-0.297	0.069	0.000018	0.0000341		0.896	0.80	-0.01752	0.03254	0.5898516

					GOYA QC		GOYA Overweight/control			GOYA BMI continuous	known	IARC QC		IARC results		
MARKER	chr	position	A1	A2	FREQ1	Rsqr	Beta	SE	p	p	gene name	freq1	Rsqr	in_beta	in_SE	in_p
rs17006670	4	142073832	C	T	0.058	0.97	0.344	0.085	0.0000513	0.0000175		0.056	0.95	0.020918	0.03898	0.5911782
rs4956410	4	142907245	A	G	0.066	0.89	-0.273	0.083	0.001073	0.0004556		0.097	0.91	0.051237	0.03282	0.1181556
rs7683103	4	148954077	A	G	0.899	0.98	0.223	0.066	0.0007155	0.0020972		0.905	0.88	0.009102	0.03228	0.7777755
rs360904	4	153077724	A	G	0.485	1.00	0.114	0.039	0.003429	0.0009189		0.458	1.00	0.001161	0.01803	0.9486048
rs12504286	4	154875651	C	T	0.522	1.00	0.107	0.039	0.005627	0.0008953		0.505	0.97	-0.02242	0.01861	0.2279656
rs6848765	4	154875710	G	T	0.522	1.00	0.107	0.039	0.005621	0.0008927		0.505	0.97	-0.02241	0.01861	0.2282442
rs2445617	4	154878478	C	T	0.478	1.00	-0.107	0.039	0.005582	0.0008707		0.497	0.96	0.021536	0.01868	0.2486612
rs12648400	4	154881881	C	T	0.522	1.00	0.107	0.039	0.005572	0.0008654		0.503	0.96	-0.02149	0.01868	0.2497324
rs11099898	4	154884471	C	T	0.522	1.00	0.107	0.039	0.005567	0.0008565		0.503	0.95	-0.02137	0.01869	0.2525178
rs12647687	4	154884663	C	G	0.478	1.00	-0.107	0.039	0.005644	0.0008637		0.497	0.95	0.02136	0.01869	0.2526773
rs1598632	4	154886832	A	G	0.522	1.00	0.107	0.039	0.00589	0.0008872		0.503	0.95	-0.0213	0.01869	0.2539996
rs11099899	4	154887037	A	G	0.522	1.00	0.107	0.039	0.005885	0.0008863		0.503	0.95	-0.02128	0.01869	0.2544563
rs6851366	4	154887879	C	T	0.478	1.00	-0.107	0.039	0.005879	0.0008855		0.497	0.95	0.021152	0.01869	0.2573809
rs4637381	4	154889706	C	G	0.522	1.00	0.107	0.039	0.005865	0.0008831		0.503	0.95	-0.02114	0.01869	0.2577132
rs11099902	4	154893728	C	T	0.478	1.00	-0.107	0.039	0.00575	0.0008713		0.498	0.95	0.020976	0.01869	0.261356
rs12512426	4	154894051	C	T	0.522	1.00	0.107	0.039	0.005727	0.0008693		0.502	0.95	-0.02096	0.01869	0.2616852
rs12498803	4	157780407	A	C	0.849	0.99	0.182	0.055	0.0009252	0.0014635		0.810	0.96	-0.012	0.02402	0.6170018
rs6536515	4	161525062	C	T	0.451	0.98	-0.121	0.039	0.002199	0.0002935		0.414	0.97	0.002083	0.0186	0.9107459
rs7677508	4	161525089	A	G	0.451	0.98	-0.121	0.039	0.002198	0.000294		0.414	0.98	0.002087	0.01859	0.9105142
rs1946867	4	161526913	C	T	0.547	0.99	0.112	0.039	0.004353	0.0005413		0.585	0.99	-0.00242	0.01844	0.8956178
rs13112097	4	161551619	C	G	0.735	0.78	0.174	0.05	0.0004471	0.0009321		0.757	0.61	0.024864	0.02677	0.3526061
rs7377326	4	161555964	A	C	0.542	0.99	0.125	0.039	0.001367	0.0001693		0.568	0.99	0.000167	0.01829	0.9927262
rs1492465	4	163248423	A	C	0.859	1.00	0.185	0.056	0.0009952	0.0031776		0.795	1.00	0.036978	0.02302	0.1079329
rs1994771	4	163253595	C	T	0.859	1.00	0.186	0.056	0.0009247	0.0029816		0.795	1.00	0.037243	0.02303	0.1055537
rs6817504	4	165133323	C	G	0.100	0.89	-0.174	0.068	0.01084	0.0009738		0.072	0.42	0.10923	0.05192	0.035265
rs1430976	4	165328425	C	T	0.815	0.99	0.143	0.05	0.00463	0.0007066		0.834	1.00	0.009195	0.02517	0.7145945
rs1430975	4	165328514	A	G	0.815	0.99	0.143	0.05	0.004612	0.0007039		0.834	1.00	0.009264	0.02516	0.712527
rs13115598	4	165336979	C	T	0.816	0.98	0.146	0.051	0.004161	0.0006351		0.838	0.97	0.012239	0.02581	0.6350858
rs7688298	4	165343709	G	T	0.923	0.99	0.227	0.074	0.00206	0.0002669		0.953	0.94	-0.0028	0.04203	0.9468166
rs10001526	4	165352417	G	T	0.923	0.99	0.23	0.074	0.001852	0.0002227		0.953	0.96	0.000558	0.04195	0.989386
rs12500150	4	165380764	C	T	0.160	1.00	-0.163	0.053	0.002073	0.0001663		0.123	1.00	-0.05763	0.02827	0.0413506
rs4437208	4	165381656	A	T	0.840	1.00	0.163	0.053	0.002078	0.0001676		0.877	1.00	0.057535	0.02827	0.0416591
rs4437209	4	165381722	G	T	0.840	1.00	0.163	0.053	0.002094	0.0001712		0.877	1.00	0.057488	0.02826	0.0418184
rs4337693	4	165382054	A	C	0.840	1.00	0.163	0.053	0.002104	0.0001732		0.877	1.00	0.0572	0.02826	0.0428037
rs4599363	4	165382163	A	T	0.160	1.00	-0.163	0.053	0.002114	0.0001751		0.123	1.00	-0.05706	0.02825	0.043297
rs4561891	4	165388371	A	C	0.840	1.00	0.163	0.053	0.002129	0.0001779		0.877	1.00	0.056957	0.02825	0.0436477
rs13141215	4	165389441	A	T	0.922	1.00	0.205	0.073	0.004727	0.0007759		0.953	1.00	0.004133	0.0414	0.9204088
rs17044785	4	165390408	A	T	0.922	1.00	0.207	0.073	0.004365	0.0007013		0.953	1.00	0.004173	0.04139	0.9196306
rs7682877	4	165390554	A	T	0.922	1.00	0.207	0.073	0.00436	0.0007004		0.953	1.00	0.004179	0.04139	0.9195073
rs17044794	4	165391095	C	T	0.078	1.00	-0.207	0.073	0.004347	0.0006977		0.047	1.00	-0.00419	0.04139	0.9193467
rs17044801	4	165392213	A	G	0.078	1.00	-0.207	0.073	0.004339	0.0006961		0.047	1.00	-0.0042	0.04139	0.9192038
rs12711364	4	165392765	C	T	0.922	1.00	0.207	0.073	0.004336	0.0006954		0.953	1.00	0.004255	0.04139	0.9180478
rs17044809	4	165393567	A	G	0.078	1.00	-0.208	0.073	0.004263	0.0006808		0.047	1.00	-0.00445	0.04137	0.9141994
rs2321381	4	165395793	A	G	0.078	1.00	-0.208	0.073	0.004259	0.0006798		0.047	1.00	-0.00447	0.04137	0.9139164
rs4362777	4	165396088	A	G	0.078	1.00	-0.207	0.073	0.004397	0.0006951		0.047	1.00	-0.00414	0.04139	0.920158
rs13109568	4	165397507	A	G	0.922	1.00	0.207	0.073	0.004397	0.0006951		0.953	0.98	0.008454	0.04148	0.8383646
rs7688153	4	165398055	C	T	0.922	1.00	0.207	0.073	0.004397	0.0006951		0.953	0.98	0.00875	0.04148	0.8327978
rs17044823	4	165399131	A	T	0.078	1.00	-0.207	0.073	0.004397	0.0006951		0.047	0.98	-0.00882	0.04148	0.8315063
rs6853526	4	165399149	A	G	0.078	1.00	-0.207	0.073	0.004397	0.0006951		0.047	0.98	-0.00885	0.04148	0.8308513
rs9308071	4	165401355	G	T	0.922	1.00	0.207	0.073	0.004397	0.0006951		0.953	0.98	0.008954	0.04148	0.8289567
rs17044842	4	165402196	A	C	0.922	1.00	0.207	0.073	0.004397	0.0006951		0.953	0.98	0.008987	0.04148	0.8283272
rs9284674	4	165403008	A	T	0.078	1.00	-0.207	0.073	0.004398	0.0006953		0.047	0.99	-0.01668	0.0413	0.6860189
rs9308076	4	165403267	A	C	0.922	1.00	0.207	0.073	0.004398	0.0006953		0.954	0.99	0.016743	0.04129	0.6848738
rs9308077	4	165403384	C	T	0.078	1.00	-0.207	0.073	0.004516	0.0007124		0.047	0.99	-0.01681	0.04129	0.6835753
rs17044855	4	165420652	C	T	0.078	1.00	-0.202	0.073	0.005664	0.0008815		0.046	0.99	-0.01764	0.04124	0.6686333
rs6829588	4	165441421	G	T	0.890	0.68	0.25	0.075	0.0008308	0.0001609		0.917	0.62	0.051536	0.04051	0.2029928
rs6845804	4	167709536	C	G	0.209	0.61	0.165	0.061	0.006873	0.0008339		0.202	0.52	0.019869	0.03027	0.5112646
rs11935476	4	169880243	C	T	0.572	0.97	0.144	0.04	0.0002787	0.0005154		0.591	0.98	-0.01254	0.01895	0.5077994
rs2062591	4	169892055	A	G	0.536	0.99	-0.145	0.039	0.0002316	0.0004853		0.505	0.94	0.013086	0.01882	0.4865107
rs13122019	4	169894890	A	G	0.462	0.99	0.146	0.039	0.0001972	0.0003561		0.487	0.95	-0.01517	0.01873	0.4177012
rs2320079	4	169899705	C	T	0.539	1.00	-0.146	0.039	0.0001963	0.0003475		0.519	0.96	0.015924	0.01863	0.392324
rs2320080	4	169899739	G	T	0.566	0.93	-0.164	0.041	0.0000554	0.000174		0.542	0.90	0.014467	0.01929	0.452839
rs6811238	4	169900190	G	T	0.539	1.00	-0.146	0.039	0.0001959	0.0003394		0.520	0.97	0.01612	0.0186	0.3856275
rs7657509	4	169901819	A	C	0.461	1.00	0.146	0.039	0.0001958	0.0003367		0.480	0.97	-0.01619	0.01858	0.3831452
rs955911	4	169903754	G	T	0.539	1.00	-0.146	0.039	0.0001956	0.0003352		0.526	0.99	0.016822	0.01841	0.3604436
rs6857155	4	169904341	C	T	0.461	1.00	0.146	0.039	0.0001957	0.0003339		0.474	0.99	-0.01685	0.0184	0.3594394
rs1392756	4	169905396	A	G	0.461	1.00	0.146	0.039	0.000189	0.0003178		0.474	0.99	-0.01685	0.01842	0.3597431
rs11132458	4	169905936	A	C	0.539	1.00	-0.146	0.039	0.0001842	0.0003064		0.526	0.99	0.01686	0.01844	0.3601564
rs9654305	4	169906466	C	T	0.461	1.00	0.147	0.039	0.0001766	0.0002883		0.474	0.98	-0.01689	0.01852	0.3612307
rs6854697	4	169909064	A	C	0.539	1.00	-0.147	0.039	0.0001788	0.0002926		0.526	0.97	0.016926	0.01861	0.3625897
rs1039385	4	169910252	A	G	0.540	0.99	-0.147	0.039	0.0001867	0.0003067		0.527	0.96	0.016967	0.0187	0.3638171
rs11722206	4	172922670	G	T	0.953	0.97	0.318	0.094	0.0007187	0.0043398		0.955	0.93	-0.03415	0.04549	0.4524584
rs17069668	4	181702281	A	G	0.070	0.97	-0.256	0.077	0.0009244	0.0008607		0.081	0.95	-0.00017	0.03404	0.9961191
rs17069773	4	181717703	C	T	0.072	0.75	-0.266	0.087	0.002189	0.0004692		0.091	0.52	-0.022	0.04344	0.612269
rs7664603	4	182229350	G	T	0.949	0.94	-0.334	0.091	0.0002523	0.0007522		0.959	0.94	-0.06048	0.04754	0.2029036
rs10213366	4	182234749	C	T	0.949	0.94	-0.334	0.091	0.0002527	0.000754		0.959	0.94	-0.06057	0.04752	0.202131
rs7440625	4	182285686	A	G	0.050	0.96	0.33	0.092	0.0003254	0.001093		0.039	0.90	0.070954	0.04912	0.1482855
rs12651346	4	182299772	C	T	0.048	0.82	0.372	0.101	0.0002303	0.0007927		0.039	0.77	0.076537	0.05225	0.1426395
rs12649204	4	182301235	G	T	0.952	0.82	-0.373	0.101	0.0002266	0.0007812		0.961	0.77	-0.07662	0.05232	0.1428143
rs13121504	4	182400130	A	G	0.936	1.00	-0.246	0.08	0.002005	0.0000119		0.914	1.00	-0.05433	0.03475	0.1176612
rs10024557	4	182400901	A	G	0.921	0.93	-0.203	0.075	0.006442	0.0001181		0.905	0.93	-0.05902	0.03407	0.0830003
rs6552883	4	186649909	A	G	0.986	0.87	0.615	0.188	0.001038	0.0001263		0.977	0.52	-0.04507	0.08583	0.5991777

					GOYA QC		GOYA Overweight/control			GOYA BMI continuous	known	IARC QC		IARC results		
MARKER	chr	position	A1	A2	FREQ1	Rsqr	Beta	SE	p	p	gene name	freq1	Rsqr	in_beta	in_SE	in_p
rs4862539	4	186650898	A	G	0.014	0.89	-0.604	0.185	0.00111	0.0001336		0.024	0.53	0.044542	0.08456	0.5980691
rs1385581	4	186651620	A	G	0.986	0.99	0.556	0.174	0.001443	0.0001686		0.977	0.53	-0.0445	0.08445	0.5979282
rs1385580	4	186651846	C	T	0.014	0.99	-0.556	0.175	0.001456	0.000168		0.024	0.54	0.044235	0.08371	0.5968972
rs7685674	4	186656448	A	G	0.989	0.98	0.545	0.196	0.005336	0.0006177		0.988	0.81	-0.01401	0.09299	0.8801169
rs7690403	4	186656479	A	G	0.989	0.98	0.543	0.196	0.005477	0.0006269		0.988	0.81	-0.01394	0.09278	0.8804985
rs7673854	4	186670182	C	T	0.989	0.98	0.544	0.196	0.005528	0.0005867		0.988	0.82	-0.0178	0.09288	0.8479308
rs6552890	4	186681104	C	T	0.010	0.99	-0.519	0.199	0.009154	0.0007236		0.012	0.81	0.019741	0.09375	0.8330845
rs341267	5	171406	C	T	0.316	1.00	0.149	0.041	0.0002888	0.0010417		0.326	1.00	0.013193	0.01946	0.4975053
rs17538179	5	1998495	C	T	0.032	0.73	-0.505	0.132	0.0001276	0.0014081		0.042	0.70	0.046611	0.05528	0.3986982
rs11741203	5	2271025	A	G	0.330	1.00	-0.138	0.041	0.0007569	0.0025339		0.402	1.00	-0.00156	0.01884	0.9338356
rs1379917	5	2284570	C	T	0.668	0.97	0.14	0.042	0.0007335	0.0027164		0.596	0.93	0.002759	0.01941	0.8869127
rs10475138	5	2292725	C	T	0.666	0.94	0.143	0.042	0.0007422	0.0027377		0.596	0.92	0.002896	0.01955	0.8821331
rs4866599	5	3727775	C	G	0.319	0.77	0.163	0.047	0.0005007	0.0008414		0.356	0.72	-0.00469	0.0221	0.8320011
rs462168	5	4318442	A	G	0.819	1.00	-0.156	0.05	0.0019	0.0005568		0.817	0.95	0.001504	0.02321	0.9482756
rs463734	5	4318681	C	T	0.815	0.94	-0.155	0.051	0.002364	0.0006295		0.808	0.88	-0.00044	0.02373	0.9850489
rs458537	5	4319325	A	G	0.824	1.00	-0.165	0.051	0.001119	0.0003803		0.833	0.95	0.000819	0.02423	0.9730101
rs463694	5	4319700	G	T	0.813	0.97	-0.155	0.05	0.002052	0.0005891		0.811	0.92	0.002496	0.02332	0.9146904
rs463803	5	4320126	C	G	0.819	1.00	-0.156	0.05	0.001911	0.0005623		0.817	0.96	0.001844	0.02318	0.9365534
rs437337	5	4320704	C	T	0.181	1.00	0.156	0.05	0.001914	0.0005631		0.183	0.96	-0.00186	0.02318	0.9359589
rs464085	5	4321221	A	G	0.181	1.00	0.156	0.05	0.001884	0.0005547		0.171	0.92	8.59E-05	0.02444	0.9971923
rs465560	5	4321276	A	G	0.181	1.00	0.156	0.05	0.001886	0.0005555		0.171	0.92	-3.9E-05	0.02443	0.998743
rs443315	5	4321960	A	C	0.819	1.00	-0.153	0.05	0.00228	0.0006708		0.817	0.96	0.002045	0.02316	0.9295672
rs458649	5	4322490	A	G	0.181	1.00	0.152	0.05	0.002377	0.0006987		0.183	0.96	-0.00208	0.02316	0.9282617
rs463691	5	4322779	A	G	0.176	1.00	0.16	0.051	0.001552	0.0005301		0.167	0.95	-0.00123	0.0242	0.9595543
rs458633	5	4323945	C	T	0.819	1.00	-0.152	0.05	0.002467	0.000718		0.830	0.92	0.000241	0.02442	0.9921336
rs460779	5	4324117	A	G	0.176	1.00	0.16	0.051	0.001536	0.0005191		0.167	0.95	-0.00144	0.02418	0.9525597
rs457677	5	4324693	C	T	0.824	1.00	-0.161	0.051	0.001531	0.0005159		0.834	0.95	0.001578	0.02417	0.947914
rs465955	5	4325349	C	G	0.177	1.00	0.162	0.051	0.001398	0.0004087		0.167	0.95	-0.00161	0.02417	0.9469287
rs436137	5	4325866	A	T	0.183	1.00	0.152	0.05	0.002391	0.0006009		0.183	0.98	-0.00369	0.02299	0.8723691
rs465033	5	4326276	A	T	0.817	1.00	-0.152	0.05	0.002392	0.0006001		0.817	0.98	0.003723	0.02299	0.8712437
rs466308	5	4326601	C	T	0.188	0.97	0.151	0.05	0.002612	0.000638		0.189	0.95	-0.0045	0.02312	0.8453558
rs456342	5	4327075	C	T	0.183	1.00	0.152	0.05	0.002393	0.0005974		0.183	0.98	-0.00376	0.02298	0.8700053
rs382860	5	4327723	C	T	0.817	1.00	-0.152	0.05	0.002394	0.0005956		0.817	0.98	0.003815	0.02297	0.8679892
rs375554	5	4327734	A	G	0.183	1.00	0.152	0.05	0.002379	0.00059		0.171	0.94	-0.00201	0.02427	0.9337819
rs456468	5	4328075	A	T	0.183	1.00	0.152	0.05	0.002403	0.0005964		0.183	0.98	-0.00387	0.02297	0.8659368
rs458177	5	4328344	A	G	0.817	1.00	-0.152	0.05	0.002419	0.0006007		0.817	0.98	0.003923	0.02296	0.8642224
rs462021	5	4328538	A	G	0.817	1.00	-0.151	0.05	0.002448	0.0006084		0.817	0.98	0.003948	0.02296	0.863353
rs457623	5	4328558	G	T	0.181	0.99	0.149	0.05	0.003143	0.0009376		0.167	0.93	-0.00019	0.02442	0.9937315
rs456741	5	4330513	A	G	0.817	1.00	-0.151	0.05	0.002483	0.0006175		0.817	0.99	0.004058	0.02294	0.8595031
rs463750	5	4330711	C	T	0.817	1.00	-0.151	0.05	0.002504	0.0006233		0.817	0.99	0.004081	0.02294	0.8586846
rs457961	5	4330869	A	G	0.183	1.00	0.151	0.05	0.002513	0.0006258		0.183	0.99	-0.0041	0.02294	0.8580329
rs462922	5	4330958	C	T	0.823	1.00	-0.161	0.051	0.001467	0.0004227		0.834	0.97	0.003006	0.02401	0.9002904
rs458695	5	4331281	C	T	0.183	1.00	0.151	0.05	0.00254	0.000633		0.183	0.99	-0.00418	0.02293	0.8552883
rs456859	5	4331385	A	G	0.183	1.00	0.151	0.05	0.002554	0.0006367		0.183	0.99	-0.00427	0.02291	0.8522002
rs466371	5	4332514	C	G	0.184	0.98	0.151	0.05	0.002773	0.0007101		0.184	0.99	-0.00434	0.02295	0.849785
rs455960	5	4332643	C	G	0.187	0.94	0.149	0.051	0.003506	0.0009664		0.173	0.91	-0.00329	0.02458	0.8936064
rs1389220	5	24920136	A	G	0.917	0.98	-0.242	0.071	0.0006381	0.005788		0.895	0.90	-0.02076	0.03135	0.5075369
rs1389219	5	24920151	A	T	0.083	0.98	0.241	0.071	0.0006394	0.0057958		0.105	0.90	0.020793	0.03133	0.5065925
rs6452233	5	24941475	A	G	0.083	1.00	0.24	0.07	0.0006131	0.0055692		0.105	0.95	0.019773	0.0304	0.5150732
rs890916	5	28363993	C	T	0.298	0.96	-0.158	0.044	0.0002816	0.0041244		0.285	0.96	0.000445	0.0205	0.9826615
rs1582922	5	28365330	C	T	0.690	1.00	0.153	0.042	0.0002937	0.0045026		0.710	0.97	-0.00201	0.02016	0.9204093
rs1582924	5	28365424	A	G	0.652	1.00	0.137	0.041	0.0007817	0.0051256		0.686	0.98	0.002418	0.01965	0.9020077
rs1582925	5	28365696	C	T	0.652	1.00	0.137	0.041	0.0007793	0.005142		0.686	0.98	0.002388	0.01965	0.9032151
rs2468463	5	28367977	C	G	0.652	1.00	0.137	0.041	0.0007749	0.0051786		0.686	0.98	0.002369	0.01965	0.9039513
rs1593900	5	28368428	A	T	0.690	1.00	0.153	0.042	0.0002877	0.004533		0.710	0.98	-0.00211	0.02016	0.916635
rs1593901	5	28368549	G	T	0.310	1.00	-0.153	0.042	0.0002849	0.0045486		0.290	0.98	0.002147	0.02016	0.9151516
rs1593902	5	28368592	C	T	0.310	1.00	-0.153	0.042	0.000282	0.0045628		0.290	0.98	0.002286	0.02016	0.9096661
rs1593903	5	28368733	G	T	0.310	1.00	-0.153	0.042	0.0002795	0.0045823		0.290	0.98	0.00231	0.02016	0.9087005
rs1074316	5	28368945	C	T	0.652	1.00	0.138	0.041	0.0007548	0.0053355		0.686	0.98	0.002138	0.01964	0.9132462
rs2115179	5	28369635	C	G	0.310	1.00	-0.154	0.042	0.0002734	0.0046298		0.290	0.98	0.002362	0.02016	0.9066503
rs2115180	5	28369729	A	G	0.690	1.00	0.154	0.042	0.0002712	0.0046512		0.710	0.98	-0.00238	0.02016	0.9059241
rs4867532	5	28381250	A	G	0.690	1.00	0.154	0.042	0.0002563	0.0049795		0.710	0.98	-0.00317	0.02014	0.8746979
rs6450614	5	28381360	C	T	0.690	1.00	0.154	0.042	0.0002583	0.0050369		0.710	0.98	-0.00318	0.02014	0.874473
rs10472127	5	28410779	A	T	0.261	0.95	-0.15	0.045	0.0009326	0.0042464		0.255	0.97	-0.01507	0.02131	0.4791422
rs3100963	5	29751421	A	G	0.246	0.72	0.187	0.053	0.0004248	0.0024597		0.223	0.69	0.015359	0.02731	0.5735425
rs1276216	5	30962955	A	T	0.125	0.85	0.195	0.063	0.002149	0.0006108		0.120	0.85	0.042578	0.03013	0.1572463
rs1276224	5	30970064	C	T	0.932	0.99	-0.202	0.077	0.008215	0.000873		0.928	0.98	-0.03841	0.03559	0.2801648
rs1276225	5	30971092	G	T	0.068	0.99	0.202	0.077	0.008232	0.000871		0.072	0.98	0.038443	0.03565	0.2804517
rs12188067	5	31406516	G	T	0.614	0.97	-0.161	0.04	0.0000694	0.003327		0.652	0.92	-0.02262	0.01991	0.2554627
rs6872363	5	31409057	A	T	0.626	1.00	-0.168	0.04	0.0000273	0.0019459		0.661	0.98	-0.01646	0.01949	0.3979137
rs13175906	5	31409677	A	G	0.374	1.00	0.168	0.04	0.0000271	0.0019352		0.339	0.98	0.016424	0.01949	0.3988906
rs10940946	5	31410410	A	T	0.374	1.00	0.168	0.04	0.0000246	0.0018335		0.339	0.98	0.016287	0.01948	0.4026792
rs13186629	5	31411810	C	T	0.626	1.00	-0.17	0.04	0.0000221	0.0016971		0.637	0.92	-0.01898	0.0199	0.3397032
rs10940948	5	31412244	G	T	0.627	1.00	-0.168	0.04	0.0000261	0.0019129		0.662	0.98	-0.01618	0.01948	0.4058146
rs7717134	5	31412880	C	T	0.633	0.97	-0.163	0.041	0.0000569	0.0029546		0.662	0.98	-0.01634	0.0195	0.4017176
rs17482570	5	31413783	A	T	0.633	0.97	-0.163	0.041	0.0000603	0.0030518		0.663	0.98	-0.01647	0.01952	0.3982893
rs6883386	5	31414285	A	G	0.637	0.96	-0.159	0.041	0.0001042	0.0041468		0.664	0.98	-0.01686	0.01956	0.3882547
rs5015013	5	31414606	A	T	0.663	0.92	-0.173	0.042	0.0000472	0.0018414		0.694	0.93	-0.01837	0.02063	0.3727146
rs5015009	5	31414799	C	G	0.638	0.96	-0.158	0.041	0.000119	0.0044674		0.667	0.97	-0.01833	0.0197	0.3516511
rs5015008	5	31414815	A	C	0.362	0.96	0.157	0.041	0.0001207	0.0045041		0.333	0.97	0.018416	0.0197	0.3495566
rs12652221	5	31415528	C	T	0.353	0.96	0.14	0.041	0.0006509	0.0115164		0.329	0.97	0.020072	0.01979	0.310053
rs12652698	5	31415847	C	T	0.352	0.96	0.14	0.041	0.0006794	0.0117996		0.322	0.99	0.022355	0.01969	0.2558766

					GOYA QC		GOYA Overweight/control			GOYA BMI continuous	known	IARC QC		IARC results		
MARKER	chr	position	A1	A2	FREQ1	Rsqr	Beta	SE	p	p	gene name	freq1	Rsqr	in_beta	in_SE	in_p
rs6876179	5	31416431	C	T	0.352	0.96	0.139	0.041	0.0007037	0.0120414		0.322	1.00	0.022444	0.01968	0.2537808
rs6895674	5	31416496	A	G	0.352	0.96	0.139	0.041	0.000721	0.0122067		0.322	1.00	0.022509	0.01968	0.25223
rs550517	5	31421693	A	C	0.479	0.84	-0.14	0.042	0.0009555	0.0135065		0.496	0.64	-0.01495	0.02304	0.5162488
rs328620	5	32086408	C	G	0.245	0.42	-0.262	0.07	0.0002015	0.0005895		0.297	0.35	0.035734	0.03349	0.285553
rs11742689	5	35841905	C	T	0.957	0.82	0.365	0.107	0.0006818	0.0025262		0.962	0.69	0.001654	0.0632	0.9791085
rs11567697	5	35894081	A	G	0.874	1.00	0.194	0.058	0.0008962	0.0005284		0.899	0.99	0.027464	0.03065	0.3698638
rs11567698	5	35894100	G	T	0.126	1.00	-0.194	0.058	0.0008953	0.0005277		0.101	0.99	-0.02748	0.03065	0.3696012
rs11567714	5	35898742	C	T	0.874	1.00	0.194	0.058	0.0008919	0.0005262		0.899	0.99	0.027523	0.03064	0.3685911
rs10941315	5	36768545	G	T	0.503	0.92	0.151	0.04	0.000171	0.0005006		0.502	0.85	0.041697	0.01966	0.0338384
rs16903808	5	37982670	C	G	0.914	0.99	0.236	0.071	0.0008523	0.00048		0.930	0.96	-0.0449	0.0349	0.1979209
rs1585698	5	51896668	G	T	0.243	1.00	-0.167	0.046	0.00028	0.0072881		0.219	1.00	-0.03538	0.02249	0.1154908
rs10512971	5	51897194	A	G	0.758	1.00	0.167	0.046	0.0002718	0.0074802		0.786	0.97	0.035161	0.023	0.1261017
rs149489	5	55564572	C	T	0.279	1.00	0.143	0.043	0.000845	0.0274307		0.315	0.95	0.007239	0.02031	0.7213289
rs152347	5	55572245	A	G	0.279	1.00	0.143	0.043	0.0008224	0.0269834		0.315	0.94	0.006997	0.02033	0.7304935
rs36734	5	55580681	A	G	0.280	1.00	0.143	0.043	0.0008037	0.0266055		0.315	0.94	0.006819	0.02034	0.7372201
rs32498	5	55584554	C	T	0.280	1.00	0.143	0.043	0.0007952	0.026435		0.315	0.94	0.006768	0.02035	0.7391887
rs152345	5	55595454	C	T	0.279	0.99	0.146	0.043	0.0006761	0.0243826		0.315	0.94	0.006629	0.02035	0.7444267
rs152344	5	55595641	A	G	0.721	0.99	-0.146	0.043	0.0006737	0.0241642		0.685	0.94	-0.00659	0.02035	0.7459801
rs152343	5	55596334	C	T	0.279	0.99	0.146	0.043	0.000692	0.0245742		0.315	0.94	0.006411	0.02039	0.7529863
rs10074627	5	64970018	A	C	0.106	0.99	0.227	0.063	0.0003532	0.0009473		0.078	0.58	-0.05449	0.04116	0.185148
rs7380465	5	67134036	A	G	0.856	0.56	-0.286	0.074	0.0001045	0.0002943		0.848	0.52	-0.0499	0.03585	0.1636295
rs7723009	5	67140957	C	T	0.161	0.90	0.221	0.056	0.0000728	0.0004209		0.167	0.69	0.035429	0.0297	0.2325314
rs1007047	5	67146473	C	T	0.851	1.00	-0.201	0.054	0.0002038	0.0009585		0.824	0.88	-0.0235	0.02579	0.3618143
rs17317938	5	67146778	A	G	0.851	1.00	-0.201	0.054	0.0002028	0.0009539		0.824	0.89	-0.02336	0.02571	0.3631217
rs2242991	5	76150615	C	G	0.894	0.77	0.197	0.072	0.005875	0.0007131		0.913	0.52	-0.05238	0.0429	0.2217191
rs6860843	5	76754453	C	G	0.229	0.96	0.183	0.047	0.0000936	0.0006227		0.219	0.88	-0.01076	0.02306	0.6403819
rs526264	5	78414166	A	T	0.380	0.99	0.126	0.041	0.002075	0.0006109		0.405	0.98	0.043744	0.01909	0.0218184
rs309585	5	82903929	A	G	0.298	0.89	0.148	0.045	0.0009201	0.0271422		0.311	0.88	-0.03072	0.0209	0.1412646
rs309583	5	82905841	C	G	0.298	0.89	0.148	0.045	0.0009217	0.027115		0.311	0.88	-0.03069	0.02088	0.1412995
rs10514345	5	90460035	C	T	0.906	0.86	-0.233	0.072	0.001146	0.0006315		0.904	0.87	-0.04433	0.03284	0.1767465
rs13158036	5	90461707	A	C	0.905	0.87	-0.231	0.071	0.00115	0.0006271		0.903	0.89	-0.04379	0.03227	0.1745343
rs2950858	5	90462389	A	G	0.097	0.89	0.226	0.07	0.001186	0.0006317		0.098	0.90	0.043541	0.03204	0.1738451
rs13163651	5	90462804	C	T	0.903	0.89	-0.225	0.069	0.001192	0.0006335		0.902	0.90	-0.04349	0.032	0.1736927
rs13190101	5	90466233	A	C	0.102	0.96	0.21	0.065	0.001374	0.0006968		0.098	0.91	0.043424	0.03192	0.1734047
rs13158026	5	90467535	C	T	0.896	1.00	-0.203	0.064	0.001481	0.0007403		0.902	0.91	-0.04338	0.0319	0.1735661
rs13161782	5	90467628	A	C	0.896	1.00	-0.203	0.064	0.001473	0.0007347		0.898	0.96	-0.04169	0.03039	0.1697375
rs6873773	5	90474229	C	T	0.894	1.00	-0.217	0.063	0.000612	0.0004352		0.892	0.97	-0.04073	0.02974	0.1705992
rs10059760	5	90475858	A	T	0.959	0.37	0.544	0.165	0.000963	0.0003354		0.962	0.32	0.054162	0.08064	0.5014345
rs13158963	5	90484910	A	G	0.106	1.00	0.221	0.064	0.0005044	0.0003342		0.108	0.97	0.041168	0.02973	0.1658335
rs13160072	5	90485563	A	G	0.106	1.00	0.221	0.064	0.0005043	0.0003338		0.108	0.97	0.041278	0.02974	0.1648121
rs13184171	5	90485869	C	T	0.106	1.00	0.221	0.064	0.0005031	0.0003327		0.108	0.97	0.041308	0.02974	0.1645267
rs13182804	5	90507349	C	T	0.892	1.00	-0.198	0.063	0.001537	0.0009685		0.884	0.93	-0.04702	0.02981	0.1145324
rs4392660	5	90582427	C	T	0.961	0.95	0.283	0.103	0.006285	0.000819		0.963	0.74	-0.02113	0.0531	0.6904219
rs7708343	5	107233081	A	G	0.137	1.00	0.186	0.056	0.0009745	0.0180107		0.117	1.00	0.024996	0.02936	0.3942134
rs1559080	5	114545914	A	T	0.061	0.83	0.286	0.09	0.001525	0.0009354		0.047	0.78	0.008952	0.04757	0.8506244
rs11241317	5	114554176	A	T	0.061	0.86	0.282	0.089	0.001472	0.0009157		0.047	0.87	0.007352	0.04497	0.8700181
rs2059061	5	114578607	C	T	0.102	0.99	0.216	0.065	0.0009072	0.0031047		0.105	0.92	0.003352	0.03097	0.9137251
rs4705731	5	114592299	A	G	0.934	0.99	-0.262	0.08	0.0009787	0.0008545		0.950	0.94	-0.03119	0.04266	0.4644005
rs1005056	5	114608641	C	T	0.066	0.98	0.261	0.08	0.001063	0.0009198		0.050	0.94	0.029011	0.04266	0.496148
rs2909841	5	114610587	A	G	0.066	0.98	0.261	0.08	0.001063	0.0009213		0.050	0.94	0.028966	0.04266	0.4968142
rs267305	5	114618790	G	T	0.934	0.98	-0.26	0.08	0.001079	0.0009449		0.950	0.94	-0.02864	0.04266	0.50166
rs169127	5	114654654	C	T	0.065	0.97	0.267	0.081	0.0009667	0.0010629		0.052	0.94	0.020375	0.04212	0.6282803
rs7732689	5	114712005	C	G	0.715	0.76	0.145	0.049	0.002794	0.0002831		0.736	0.65	0.028747	0.02511	0.2519389
rs2963765	5	114720973	A	G	0.547	1.00	0.107	0.039	0.005821	0.0004637		0.540	0.96	0.010925	0.0187	0.5587116
rs2964560	5	114721020	C	T	0.547	1.00	0.107	0.039	0.005781	0.0004621		0.538	0.95	0.01024	0.01871	0.5839112
rs2925172	5	114729688	A	G	0.483	1.00	-0.119	0.039	0.002085	0.0002526		0.477	0.96	-0.0057	0.01859	0.7590622
rs17383755	5	114730035	A	T	0.367	0.97	-0.129	0.041	0.001581	0.0005258		0.358	0.92	-0.01108	0.01956	0.5706255
rs2963749	5	114734391	A	G	0.483	1.00	-0.12	0.039	0.00188	0.000233		0.478	0.96	-0.00554	0.01857	0.7653742
rs17383865	5	114735264	A	G	0.517	1.00	0.12	0.039	0.001846	0.0002298		0.522	0.96	0.005515	0.01857	0.7662787
rs2963747	5	114735588	A	G	0.517	1.00	0.12	0.039	0.001796	0.000225		0.522	0.96	0.005486	0.01856	0.7674277
rs2925170	5	114736503	C	T	0.483	1.00	-0.121	0.039	0.001732	0.000219		0.478	0.96	-0.00543	0.01856	0.7696867
rs2198712	5	114741070	C	G	0.482	0.99	-0.121	0.039	0.001715	0.0002329		0.478	0.96	-0.0048	0.01853	0.7954542
rs11950882	5	114771607	C	G	0.324	0.88	-0.164	0.044	0.0001895	0.0000279		0.256	0.83	-0.02987	0.02258	0.1855766
rs2963750	5	114772203	A	G	0.547	0.99	0.126	0.039	0.001209	0.0002152		0.635	0.89	0.021985	0.0196	0.2616946
rs269526	5	114782323	A	G	0.500	0.99	0.103	0.039	0.008025	0.0007367		0.465	0.97	0.031998	0.01839	0.0817219
rs17428689	5	119984971	C	T	0.933	0.62	0.283	0.099	0.004224	0.0003167		0.933	0.61	0.045826	0.04492	0.3072338
rs7705981	5	123603509	A	G	0.064	0.82	0.304	0.088	0.0005104	0.0018422		0.084	0.69	-0.01627	0.04131	0.6933647
rs1476096	5	133864920	A	C	0.407	0.99	0.135	0.039	0.0006088	0.0066492		0.419	1.00	-0.02205	0.01859	0.235078
rs1981627	5	133866079	A	G	0.408	1.00	0.131	0.039	0.0008638	0.008814		0.421	0.99	-0.02278	0.01863	0.2211706
rs489175	5	134475577	A	G	0.018	0.95	-0.457	0.153	0.0029	0.0005679		0.018	0.76	0.002877	0.06977	0.9670755
rs3849047	5	138008375	C	T	0.669	0.88	0.167	0.044	0.000126	0.00000685		0.603	0.53	-0.0025	0.02499	0.9203213
rs6596446	5	138008754	C	T	0.342	1.00	-0.138	0.041	0.0006642	0.0001519		0.415	0.57	-0.0225	0.02408	0.3496183
rs734597	5	141093111	A	G	0.469	1.00	-0.159	0.039	0.0000446	0.0001813		0.431	0.84	-0.05165	0.01993	0.0095132
rs887269	5	141094801	A	G	0.171	0.99	-0.187	0.052	0.0003108	0.0003745		0.148	1.00	-0.08342	0.02543	0.0010312
rs32923	5	141096246	A	G	0.744	0.99	0.134	0.044	0.00257	0.0005744		0.778	0.88	0.068313	0.023	0.0029578
rs32926	5	141099224	C	T	0.655	0.99	0.139	0.041	0.0007356	0.0001713		0.704	0.87	0.047367	0.02112	0.0248315
rs39990	5	141101261	C	G	0.752	0.98	0.134	0.045	0.002979	0.0007524		0.779	0.87	0.068664	0.02315	0.0029982
rs3797057	5	141109313	C	G	0.171	0.99	-0.193	0.052	0.000208	0.0003434		0.150	0.96	-0.08659	0.02575	0.0007685
rs32942	5	141111641	A	G	0.230	0.96	-0.147	0.047	0.001629	0.0007791		0.215	0.89	-0.06253	0.0232	0.0070085
rs42757	5	141117481	C	G	0.830	0.97	0.191	0.052	0.0002713	0.0004143		0.849	0.93	0.082936	0.02609	0.0014727
rs6873289	5	143060513	A	G	0.017	0.98	-0.456	0.158	0.003775	0.0009361		0.017	0.97	-0.01954	0.06875	0.7760715
rs7709610	5	143067477	C	T	0.017	0.98	-0.457	0.157	0.003719	0.0009181		0.017	0.97	-0.01851	0.06861	0.7871647

					GOYA QC		GOYA Overweight/control			GOYA BMI continuous	known	IARC QC		IARC results		
MARKER	chr	position	A1	A2	FREQ1	Rsqr	Beta	SE	p	p	gene name	freq1	Rsqr	in_beta	in_SE	in_p
rs3850575	5	143882583	A	G	0.227	1.00	-0.129	0.046	0.005195	0.0008787		0.192	0.99	-0.00718	0.02314	0.7561911
rs13166009	5	143885977	A	G	0.774	1.00	0.129	0.046	0.005318	0.0009676		0.808	0.99	0.007258	0.02313	0.7535285
rs17702773	5	143890165	C	T	0.775	1.00	0.132	0.046	0.004498	0.0008983		0.810	0.98	0.008082	0.02328	0.7281944
rs7728919	5	143891780	C	T	0.251	1.00	-0.132	0.045	0.003077	0.00087		0.222	0.99	-0.01056	0.02201	0.6312822
rs13184741	5	143894324	A	C	0.749	1.00	0.132	0.045	0.003076	0.0008708		0.778	0.99	0.010652	0.022	0.6280371
rs6870605	5	143900921	C	T	0.251	1.00	-0.132	0.045	0.003075	0.0008717		0.222	0.99	-0.01087	0.02198	0.6207335
rs6873273	5	143954162	G	T	0.226	0.96	-0.138	0.047	0.003367	0.0004679		0.208	0.95	-0.00535	0.023	0.8159686
rs17451641	5	144010781	A	G	0.774	1.00	0.131	0.046	0.004674	0.0008851		0.807	1.00	0.011126	0.02309	0.6296188
rs17451662	5	144010947	A	C	0.226	1.00	-0.131	0.046	0.004661	0.0008835		0.193	1.00	-0.01121	0.02309	0.6271043
rs247567	5	144140032	C	T	0.316	1.00	-0.138	0.042	0.00097	0.0011651		0.311	1.00	0.004704	0.01957	0.8098485
rs7730610	5	144221548	A	G	0.311	1.00	-0.144	0.042	0.0005952	0.0004711		0.307	0.96	-0.00043	0.0199	0.982697
rs13181482	5	144223143	A	C	0.315	1.00	-0.144	0.042	0.0005643	0.0004585		0.317	0.94	-0.00422	0.02004	0.833056
rs1368338	5	144223480	A	G	0.311	1.00	-0.144	0.042	0.0005942	0.0004701		0.307	0.96	-0.00048	0.0199	0.9805399
rs6887305	5	144224305	C	T	0.312	1.00	-0.143	0.042	0.0006348	0.0004998		0.311	0.98	-0.00091	0.01963	0.9628119
rs6887503	5	144224429	C	T	0.312	1.00	-0.143	0.042	0.0006346	0.0004998		0.311	0.98	-0.00095	0.01963	0.9615065
rs13185780	5	144238228	C	G	0.312	1.00	-0.144	0.042	0.0006315	0.0004938		0.310	0.98	-0.00092	0.01964	0.9626998
rs7733075	5	144243435	G	T	0.340	1.00	-0.125	0.041	0.002273	0.0004901		0.334	1.00	0.007825	0.01902	0.6804827
rs6859325	5	144249706	C	T	0.340	1.00	-0.125	0.041	0.002159	0.0004647		0.334	1.00	0.007928	0.01899	0.6760897
rs13158888	5	144257996	A	G	0.660	1.00	0.126	0.041	0.002011	0.0004323		0.666	1.00	-0.00809	0.01899	0.6697446
rs13183837	5	144258139	C	T	0.660	1.00	0.126	0.041	0.001989	0.0004276		0.666	1.00	-0.00838	0.01898	0.6583623
rs12717928	5	144260312	C	T	0.660	1.00	0.126	0.041	0.00195	0.0004191		0.666	1.00	-0.00849	0.01898	0.6544417
rs12717929	5	144260390	A	G	0.340	1.00	-0.126	0.041	0.001932	0.000415		0.334	1.00	0.00852	0.01897	0.6531386
rs11741175	5	144262430	A	T	0.660	1.00	0.127	0.041	0.001911	0.0004106		0.667	1.00	-0.00855	0.01897	0.6518585
rs728301	5	144263698	A	C	0.660	1.00	0.127	0.041	0.001838	0.0003946		0.667	1.00	-0.00865	0.01897	0.6481795
rs7723406	5	144271503	A	C	0.659	1.00	0.128	0.041	0.00176	0.0003699		0.667	1.00	-0.00836	0.01897	0.659039
rs4912684	5	144272984	A	C	0.675	0.99	0.129	0.041	0.00176	0.0004782		0.701	0.99	-0.01364	0.0195	0.4838836
rs3902103	5	144275148	C	T	0.341	1.00	-0.128	0.041	0.001733	0.0003611		0.334	1.00	0.00813	0.01896	0.6678478
rs11167873	5	144276327	A	G	0.622	0.99	0.129	0.04	0.001312	0.0004846		0.607	0.99	-0.01154	0.01865	0.535652
rs995166	5	144278036	A	C	0.341	1.00	-0.127	0.041	0.001767	0.0003695		0.334	1.00	0.008193	0.01897	0.6654875
rs10070261	5	144280014	A	G	0.341	1.00	-0.127	0.041	0.001795	0.0003762		0.334	1.00	0.008224	0.01897	0.6642918
rs10071840	5	144280144	C	T	0.659	1.00	0.127	0.041	0.001812	0.0003801		0.667	1.00	-0.00824	0.01897	0.6637151
rs11749797	5	144282314	A	T	0.659	1.00	0.127	0.041	0.001828	0.0003841		0.667	1.00	-0.00826	0.01897	0.6630498
rs7736281	5	144282862	A	G	0.341	1.00	-0.127	0.041	0.001845	0.0003881		0.334	1.00	0.008273	0.01897	0.6624734
rs7726039	5	144284739	A	G	0.659	1.00	0.127	0.041	0.001862	0.0003921		0.667	1.00	-0.00829	0.01897	0.6617813
rs4913011	5	144286140	C	T	0.659	1.00	0.127	0.041	0.001896	0.0004003		0.667	1.00	-0.0083	0.01897	0.6612729
rs12522679	5	144288027	C	T	0.659	1.00	0.127	0.041	0.001913	0.0004046		0.667	1.00	-0.00832	0.01897	0.6606013
rs3944085	5	144288885	A	G	0.341	1.00	-0.126	0.041	0.001931	0.0004088		0.334	1.00	0.008353	0.01897	0.659461
rs6863541	5	144300869	G	T	0.660	1.00	0.125	0.041	0.002224	0.0004966		0.666	0.99	-0.00904	0.019	0.6340235
rs2108662	5	144303818	G	T	0.624	1.00	0.122	0.04	0.002151	0.0006923		0.632	0.99	-0.00905	0.01874	0.6287813
rs10477259	5	144304352	C	T	0.624	1.00	0.122	0.04	0.002235	0.0007312		0.632	0.99	-0.00907	0.01874	0.6279668
rs5007554	5	144304688	G	T	0.624	1.00	0.121	0.04	0.002323	0.0007724		0.632	0.99	-0.00914	0.01874	0.6254947
rs767665	5	144307607	A	G	0.624	1.00	0.119	0.04	0.002725	0.0009673		0.632	0.99	-0.00935	0.01875	0.6176149
rs4913013	5	144317485	A	C	0.624	1.00	0.119	0.04	0.002784	0.0009824		0.632	0.99	-0.00974	0.01876	0.6031708
rs9325009	5	145784610	C	T	0.824	0.99	-0.143	0.052	0.005716	0.0002323		0.842	0.42	0.046292	0.03876	0.2319832
rs17104665	5	145822662	A	G	0.927	1.00	0.347	0.076	0.00000454	0.0000115		0.949	1.00	0.013674	0.03662	0.7086014
rs998051	5	145855452	G	T	0.073	1.00	-0.346	0.076	0.00000464	0.0000119		0.051	1.00	-0.01376	0.03664	0.7070692
rs2033471	5	145857761	C	G	0.927	1.00	0.346	0.076	0.00000468	0.000012		0.949	0.99	0.013775	0.03673	0.7073592
rs1802027	5	145870421	A	G	0.073	1.00	-0.346	0.076	0.00000482	0.0000124		0.050	0.95	-0.01411	0.03777	0.7084003
rs17104742	5	145875751	A	G	0.073	0.99	-0.346	0.076	0.00000542	0.0000144		0.050	0.93	-0.01422	0.03818	0.709434
rs17651412	5	146918333	C	T	0.213	1.00	0.158	0.047	0.000759	0.0037265		0.263	0.61	-0.07056	0.02685	0.0085498
rs17625543	5	147260570	C	T	0.930	1.00	-0.303	0.077	0.0000781	0.0020069		0.929	0.99	-0.04317	0.03659	0.2377156
rs17703848	5	147261222	A	G	0.070	1.00	0.302	0.077	0.0000796	0.0020419		0.071	0.99	0.043167	0.03659	0.2377358
rs7715716	5	147269992	A	G	0.920	0.84	-0.273	0.078	0.0004973	0.0108871		0.931	0.71	-0.05659	0.04441	0.2021753
rs17537018	5	155391381	A	G	0.795	0.83	0.177	0.053	0.0008152	0.0143798		0.792	0.80	-0.0077	0.02521	0.7599317
rs4601027	5	155400015	C	T	0.211	1.00	-0.158	0.048	0.0009237	0.0073397		0.212	0.95	0.014636	0.02278	0.5202433
rs17537158	5	155400711	G	T	0.211	1.00	-0.158	0.048	0.0009228	0.007313		0.212	0.99	0.014213	0.02229	0.5233145
rs7736045	5	155401074	C	G	0.790	1.00	0.158	0.048	0.0009289	0.0073552		0.788	0.99	-0.0142	0.02227	0.5231785
rs4704943	5	155403024	C	T	0.211	1.00	-0.158	0.048	0.0009376	0.0074152		0.212	1.00	0.014145	0.0222	0.5236135
rs7733049	5	158422054	C	G	0.381	0.66	-0.148	0.049	0.002348	0.0008834		0.351	0.63	0.049113	0.02376	0.0385602
rs4326180	5	161658090	C	T	0.068	0.68	-0.32	0.093	0.0005869	0.0008272		0.057	0.66	-0.06794	0.04785	0.1553128
rs1833534	5	162753563	C	G	0.858	0.98	-0.172	0.057	0.002516	0.0005452		0.867	0.94	-0.04446	0.02738	0.1041013
rs1025014	5	162762526	A	G	0.862	1.00	-0.176	0.057	0.002008	0.0003536		0.866	1.00	-0.04347	0.02657	0.1015092
rs4869081	5	162765865	A	G	0.110	0.97	0.195	0.063	0.002101	0.0003231		0.112	0.96	0.027305	0.02897	0.3456077
rs299304	5	162779300	A	G	0.857	1.00	-0.178	0.056	0.00159	0.0002213		0.865	1.00	-0.04035	0.02621	0.1233627
rs2112770	5	162780410	A	G	0.147	0.90	0.18	0.058	0.002056	0.0002134		0.137	0.89	0.01574	0.0272	0.5625245
rs299284	5	162829228	C	T	0.876	1.00	-0.184	0.059	0.001913	0.0002041		0.881	1.00	-0.01595	0.02719	0.5570819
rs299282	5	162831595	A	G	0.877	1.00	-0.184	0.06	0.001969	0.0002089		0.881	1.00	-0.01561	0.0272	0.5657127
rs213504	5	162831905	C	T	0.877	1.00	-0.184	0.06	0.001975	0.0002095		0.881	1.00	-0.0155	0.0272	0.5684249
rs7737940	5	164406324	A	G	0.605	0.96	0.128	0.041	0.001755	0.0000296		0.610	0.99	0.009271	0.01892	0.6238012
rs4407647	5	164409001	G	T	0.605	0.97	0.127	0.041	0.001856	0.0000318		0.611	1.00	0.00924	0.01888	0.6242812
rs7720621	5	164419339	G	T	0.596	0.99	0.126	0.04	0.001595	0.0000373		0.596	0.99	0.014039	0.01885	0.4559421
rs9986163	5	164421844	A	G	0.596	0.99	0.126	0.04	0.00161	0.0000381		0.596	0.99	0.01409	0.01885	0.4543155
rs7708696	5	164427314	A	G	0.404	0.99	-0.127	0.04	0.001483	0.0000339		0.404	0.99	-0.01403	0.01886	0.4566111
rs10866751	5	164428700	A	G	0.421	0.95	-0.122	0.041	0.002761	0.0001468		0.425	0.96	-0.00795	0.01915	0.6779118
rs1421689	5	164434997	A	G	0.421	0.95	-0.122	0.041	0.002721	0.0001433		0.425	0.96	-0.00791	0.01915	0.6791945
rs7724765	5	164435608	C	T	0.516	0.99	0.122	0.039	0.001918	0.0003817		0.529	0.97	-0.00679	0.01891	0.7191101
rs7727218	5	164439221	A	G	0.601	1.00	0.127	0.04	0.001525	0.0000304		0.599	1.00	0.012729	0.01888	0.499873
rs13160655	5	164439509	C	T	0.601	1.00	0.126	0.04	0.001528	0.0000304		0.599	1.00	0.012683	0.01888	0.5013711
rs7707544	5	164439818	A	G	0.400	1.00	-0.126	0.04	0.001529	0.0000303		0.401	1.00	-0.01265	0.01888	0.5023459
rs1946187	5	164439990	A	G	0.601	1.00	0.126	0.04	0.001528	0.0000303		0.599	1.00	0.012634	0.01888	0.5029582
rs1421681	5	164440850	C	T	0.400	1.00	-0.126	0.04	0.001528	0.0000302		0.401	1.00	-0.01262	0.01888	0.5034688
rs1019444	5	164442866	C	T	0.400	1.00	-0.126	0.04	0.001528	0.0000299		0.401	1.00	-0.01256	0.01887	0.5052249

					GOYA QC		GOYA Overweight/control			GOYA BMI continuous	known	IARC QC		IARC results		
MARKER	chr	position	A1	A2	FREQ1	Rsqr	Beta	SE	p	p	gene name	freq1	Rsqr	in_beta	in_SE	in_p
rs17072571	5	164444082	A	G	0.601	1.00	0.126	0.04	0.001635	0.0000309		0.599	1.00	0.012815	0.01889	0.4970688
rs13160815	5	164447459	C	T	0.399	1.00	-0.125	0.04	0.001668	0.0000313		0.401	1.00	-0.01291	0.01889	0.4939152
rs6895727	5	164452150	C	T	0.484	1.00	-0.121	0.039	0.002063	0.0003856		0.471	0.97	0.006482	0.01891	0.7315568
rs1895161	5	164452680	A	G	0.601	1.00	0.126	0.04	0.001623	0.0000306		0.599	1.00	0.013163	0.0189	0.4858441
rs6887177	5	164453716	C	T	0.517	0.99	0.121	0.039	0.001967	0.0003732		0.529	0.97	-0.00634	0.01892	0.7373854
rs7735043	5	164462127	A	G	0.485	1.00	-0.12	0.039	0.002056	0.0004619		0.492	0.97	0.008022	0.01873	0.6681167
rs10073086	5	164464892	C	T	0.601	1.00	0.132	0.04	0.0008958	0.0000223		0.596	0.99	0.017687	0.0189	0.3489862
rs1592758	5	164471755	C	T	0.399	1.00	-0.133	0.04	0.0008781	0.0000221		0.404	0.99	-0.01782	0.01889	0.3452522
rs4577712	5	164478789	C	T	0.601	1.00	0.134	0.04	0.0008059	0.0000214		0.596	0.99	0.018014	0.01888	0.3396357
rs4323250	5	164481325	C	T	0.399	1.00	-0.134	0.04	0.0007551	0.0000205		0.405	0.99	-0.01847	0.01884	0.326508
rs1363113	5	164483147	A	G	0.601	1.00	0.135	0.04	0.0007279	0.0000201		0.595	0.99	0.018457	0.01883	0.3266174
rs10476496	5	164484231	C	T	0.399	1.00	-0.135	0.04	0.0007103	0.0000198		0.405	0.99	-0.01845	0.01883	0.3266667
rs13162946	5	164487857	C	G	0.601	1.00	0.135	0.04	0.0006822	0.0000193		0.595	1.00	0.018429	0.01882	0.3269794
rs6876275	5	164491161	G	T	0.399	1.00	-0.136	0.04	0.0006502	0.0000188		0.405	1.00	-0.01842	0.01881	0.3271385
rs10515891	5	164494733	C	T	0.399	1.00	-0.136	0.04	0.0006416	0.0000187		0.405	1.00	-0.01843	0.0188	0.3265935
rs6556809	5	164496526	A	T	0.399	1.00	-0.136	0.04	0.0006117	0.0000177		0.405	1.00	-0.01853	0.0188	0.3239988
rs7447491	5	164499284	A	G	0.602	1.00	0.138	0.04	0.0005143	0.0000149		0.595	1.00	0.018569	0.01879	0.3226829
rs1421713	5	164516134	A	G	0.398	1.00	-0.139	0.04	0.0004557	0.0000137		0.405	1.00	-0.01843	0.01877	0.3259735
rs6556811	5	164518542	C	T	0.602	1.00	0.14	0.04	0.0004439	0.0000133		0.595	1.00	0.018298	0.01882	0.330585
rs9314055	5	164520661	G	T	0.604	0.99	0.138	0.04	0.0005531	0.0000172		0.600	0.97	0.020642	0.01907	0.2787304
rs1363117	5	164548975	C	T	0.604	0.99	0.146	0.04	0.0002658	0.0000126		0.596	0.99	0.019519	0.01896	0.3029758
rs13156646	5	164551933	C	G	0.396	0.99	-0.146	0.04	0.0002573	0.0000119		0.404	0.99	-0.01982	0.01896	0.2955204
rs13186159	5	164558781	A	G	0.604	0.99	0.148	0.04	0.0002191	0.00001		0.596	0.99	0.020196	0.01896	0.286374
rs11135356	5	164564196	C	T	0.396	0.99	-0.148	0.04	0.0002071	0.0000095		0.404	0.99	-0.02044	0.01896	0.2807595
rs11135357	5	164564286	A	G	0.605	0.99	0.149	0.04	0.0002044	0.00000939		0.596	0.99	0.020444	0.01897	0.2806437
rs6870178	5	164575914	C	T	0.609	0.97	0.141	0.04	0.000522	0.0000388		0.607	0.96	0.027195	0.01923	0.1569261
rs885477	5	164578292	A	G	0.598	1.00	0.146	0.04	0.0002334	0.00000999		0.597	1.00	0.027737	0.01889	0.1416843
rs2013312	5	164578503	A	T	0.598	1.00	0.146	0.04	0.0002342	0.00001		0.597	1.00	0.027762	0.01889	0.1413142
rs13165528	5	164588206	C	T	0.598	1.00	0.147	0.04	0.000231	0.00000951		0.597	0.99	0.027873	0.01891	0.1402404
rs11135361	5	164590574	A	C	0.548	1.00	0.1	0.039	0.01033	0.0006335		0.544	0.99	0.027098	0.01865	0.1459402
rs7720663	5	164598808	A	G	0.599	1.00	0.147	0.04	0.0002253	0.00000634		0.596	0.99	0.028111	0.01896	0.1378497
rs1433004	5	164605980	G	T	0.402	1.00	-0.146	0.04	0.0002366	0.00000779		0.404	0.98	-0.02929	0.01907	0.1243801
rs6556813	5	164611614	A	G	0.592	0.94	0.142	0.041	0.0004832	0.0000153		0.593	0.95	0.029781	0.01932	0.1229188
rs1024997	5	164612866	C	T	0.469	0.92	-0.135	0.041	0.0008896	0.0000594		0.502	0.92	-0.01686	0.01906	0.3760384
rs7732563	5	164625394	A	G	0.501	1.00	0.109	0.039	0.005141	0.0003661		0.482	0.96	0.022623	0.0187	0.2260998
rs11135362	5	164628249	A	G	0.502	1.00	0.109	0.039	0.00514	0.000366		0.482	0.96	0.022584	0.0187	0.2268395
rs1432999	5	164640667	C	T	0.498	1.00	-0.107	0.039	0.005639	0.00041		0.517	0.98	-0.01889	0.01851	0.3070692
rs1368422	5	164646953	A	T	0.502	1.00	0.108	0.039	0.005364	0.0003801		0.483	0.98	0.018956	0.01851	0.3054081
rs1433007	5	164673606	C	T	0.501	1.00	0.11	0.039	0.00456	0.00031		0.483	0.98	0.018696	0.01852	0.3123507
rs1347151	5	164680701	A	C	0.498	1.00	-0.111	0.039	0.004355	0.0002865		0.517	0.98	-0.01855	0.01853	0.3164048
rs1368416	5	164697390	A	C	0.518	1.00	0.112	0.039	0.003789	0.0003549		0.481	1.00	0.010402	0.01849	0.5734209
rs4323253	5	164716400	C	T	0.517	0.98	0.117	0.039	0.00282	0.0001953		0.481	0.93	0.011091	0.01903	0.5597386
rs2216637	5	164912173	A	G	0.540	1.00	-0.133	0.039	0.0005761	0.0122158		0.529	0.94	-0.01246	0.0189	0.509586
rs6864400	5	169417805	C	T	0.284	1.00	-0.13	0.043	0.00228	0.0009363		0.310	1.00	0.049818	0.01982	0.01189
rs10044671	5	171942404	C	G	0.264	0.99	-0.105	0.044	0.01749	0.0008575		0.303	0.99	-0.02939	0.01986	0.1385122
rs10036547	5	171942585	C	T	0.265	1.00	-0.106	0.044	0.01639	0.0009122		0.306	0.99	-0.03022	0.0198	0.1266665
rs12652104	5	171943029	A	G	0.735	1.00	0.106	0.044	0.0162	0.0008992		0.694	0.99	0.030192	0.0198	0.1270642
rs6869285	5	171944056	A	G	0.265	1.00	-0.106	0.044	0.01574	0.0008672		0.306	0.99	-0.0301	0.01983	0.1287493
rs10069595	5	171944547	A	G	0.265	1.00	-0.107	0.044	0.01548	0.0008489		0.306	0.98	-0.02998	0.01987	0.1310291
rs7735018	5	171945071	C	T	0.265	0.99	-0.109	0.044	0.0138	0.0007354		0.306	0.98	-0.02996	0.01987	0.1313632
rs10037250	5	171945112	A	T	0.736	0.99	0.109	0.044	0.01334	0.0007048		0.694	0.98	0.029932	0.01988	0.1318599
rs7714953	5	171945200	A	G	0.736	0.99	0.109	0.044	0.01319	0.0006952		0.694	0.98	0.029905	0.01989	0.1324476
rs714715	5	171945891	A	G	0.262	1.00	-0.117	0.044	0.008331	0.0003992		0.306	0.98	-0.02986	0.0199	0.1331903
rs7726917	5	171947950	A	C	0.738	0.99	0.118	0.044	0.007954	0.0003523		0.694	0.98	0.029768	0.01993	0.1349402
rs11950830	5	171954196	C	T	0.711	0.94	0.134	0.044	0.002204	0.0001357		0.660	0.91	0.033339	0.02018	0.0982643
rs6873790	5	171957742	C	G	0.715	0.89	0.117	0.045	0.009395	0.0006382		0.683	0.73	0.033277	0.02297	0.1471432
rs359419	5	173279257	C	T	0.962	0.93	0.401	0.108	0.0002003	0.0002046		0.975	0.97	-0.11622	0.05501	0.0345206
rs1106693	5	173285369	A	G	0.962	0.93	0.399	0.108	0.0002064	0.0002143		0.975	0.97	-0.11621	0.05502	0.0345481
rs6556096	5	173295063	C	T	0.951	0.79	0.354	0.102	0.0005398	0.0003675		0.962	0.76	-0.11094	0.05169	0.0317344
rs11739436	5	173296531	C	G	0.961	0.92	0.392	0.107	0.0002452	0.0002818		0.975	0.96	-0.1156	0.05505	0.0356065
rs2170951	5	173322339	A	C	0.039	0.92	-0.394	0.106	0.0002185	0.000249		0.025	0.96	0.109602	0.05515	0.0467431
rs6874931	5	177325761	A	G	0.021	0.56	0.73	0.185	0.0000769	0.0007386		0.024	0.50	0.081978	0.08518	0.3354376
rs6896573	5	177327743	A	G	0.171	1.00	0.162	0.051	0.001612	0.0001684		0.152	0.76	0.032703	0.0287	0.2540818
rs6885798	5	177328806	A	G	0.829	1.00	-0.162	0.051	0.001612	0.0001684		0.848	0.75	-0.03276	0.02881	0.2550515
rs12652161	5	178774482	C	T	0.629	1.00	-0.129	0.04	0.00143	0.0006876		0.649	0.94	-0.03125	0.01962	0.1108424
rs11749727	5	179540966	C	T	0.363	1.00	-0.148	0.041	0.0002743	0.0050347		0.402	0.36	-0.01907	0.03169	0.5470205
rs13168609	5	179545868	G	T	0.578	0.76	0.151	0.045	0.0008418	0.0093727		0.533	0.39	0.007472	0.02985	0.8021424
rs2166085	5	179583945	C	T	0.014	0.83	0.528	0.189	0.005134	0.0004882		0.015	0.71	-0.00026	0.09779	0.9978582
rs2493013	6	461350	A	C	0.227	1.00	-0.158	0.046	0.0006574	0.0060525		0.253	0.99	0.053607	0.02124	0.01154
rs465582	6	1159290	A	G	0.275	1.00	-0.143	0.043	0.0008954	0.0112071		0.259	0.97	-0.03037	0.02063	0.1407326
rs9503125	6	2158254	A	G	0.162	1.00	-0.175	0.053	0.0009292	0.0020422		0.145	1.00	0.004788	0.02509	0.8485199
rs3778566	6	2158784	C	T	0.162	1.00	-0.175	0.053	0.0009292	0.0020431		0.145	1.00	0.004829	0.02508	0.8472092
rs9501818	6	2159631	A	G	0.162	1.00	-0.175	0.053	0.0009213	0.0020238		0.145	1.00	0.004775	0.02508	0.8488879
rs6903476	6	2167461	A	G	0.162	1.00	-0.175	0.053	0.0008951	0.0019553		0.145	1.00	0.004364	0.02509	0.8617767
rs9503127	6	2167732	C	G	0.162	1.00	-0.175	0.053	0.0008992	0.0019611		0.145	1.00	0.004326	0.02509	0.8629671
rs9503146	6	2291268	A	C	0.180	1.00	-0.168	0.051	0.0008938	0.0020349		0.160	1.00	-0.00227	0.02452	0.9262173
rs6917662	6	2302933	G	T	0.181	0.97	-0.169	0.051	0.0009689	0.0022261		0.162	0.97	-0.00194	0.02478	0.9374671
rs12661523	6	5117008	G	T	0.287	1.00	0.117	0.043	0.006229	0.0008943		0.336	0.90	-0.01373	0.02053	0.5033249
rs12525299	6	5199623	G	T	0.749	0.88	-0.143	0.048	0.002561	0.0005041		0.719	0.80	0.004098	0.02299	0.858381
rs17141291	6	5728257	C	G	0.883	0.98	0.194	0.062	0.001605	0.0002283		0.882	0.73	-0.01155	0.03334	0.7288643
rs4960132	6	5728966	C	T	0.883	0.99	0.196	0.061	0.001475	0.000206		0.882	0.70	-0.01292	0.03393	0.7031595
rs6597163	6	5730425	C	G	0.117	0.99	-0.196	0.061	0.001449	0.0002017		0.118	0.70	0.013125	0.034	0.6992666

					GOYA QC		GOYA Overweight/control			GOYA BMI continuous	known	IARC QC		IARC results		
MARKER	chr	position	A1	A2	FREQ1	Rsqr	Beta	SE	p	p	gene name	freq1	Rsqr	in_beta	in_SE	in_p
rs1108303	6	5730673	A	T	0.117	0.99	-0.196	0.061	0.001438	0.0001997		0.118	0.69	0.013288	0.03408	0.6963584
rs1108304	6	5730788	C	T	0.883	0.99	0.196	0.061	0.001417	0.0001961		0.882	0.69	-0.01332	0.0341	0.6958426
rs12528811	6	5732399	A	G	0.884	1.00	0.198	0.061	0.001232	0.0001653		0.881	0.68	-0.0141	0.0344	0.6816745
rs4960135	6	5732976	A	G	0.116	1.00	-0.196	0.061	0.001401	0.0002019		0.119	0.68	0.01421	0.03442	0.6794922
rs4960136	6	5733000	C	T	0.884	1.00	0.195	0.061	0.001448	0.0002124		0.881	0.68	-0.01424	0.03444	0.6788661
rs4960137	6	5733013	C	T	0.116	0.97	-0.198	0.062	0.001418	0.0002045		0.119	0.67	0.014551	0.03458	0.6736285
rs4960138	6	5733021	G	T	0.961	0.55	0.486	0.138	0.000433	0.0001336		0.965	0.33	-0.0553	0.08525	0.5162
rs1742929	6	6173024	A	G	0.140	0.93	-0.195	0.058	0.0008125	0.0001071		0.112	0.91	0.019371	0.02922	0.5070448
rs1742930	6	6173405	A	G	0.141	1.00	-0.184	0.056	0.001015	0.0001377		0.112	0.99	0.018636	0.02803	0.5058015
rs1622769	6	6182206	A	G	0.858	0.95	0.188	0.057	0.001022	0.000169		0.887	0.84	-0.01895	0.03012	0.5289793
rs1742923	6	6186007	A	G	0.137	0.91	-0.195	0.059	0.0009648	0.0001754		0.105	0.77	0.01627	0.03284	0.6199549
rs1781795	6	6186280	C	T	0.863	0.91	0.196	0.059	0.0009519	0.000174		0.895	0.77	-0.01626	0.03284	0.6202332
rs11970705	6	11337939	A	G	0.107	0.73	0.261	0.075	0.0004636	0.0107628		0.133	0.81	0.008403	0.02936	0.7745561
rs9380152	6	11342021	C	T	0.904	0.63	-0.307	0.083	0.0002394	0.0041946		0.875	0.70	-0.00541	0.03205	0.8659512
rs10428851	6	11759903	C	T	0.657	0.98	0.115	0.042	0.005782	0.0009662		0.710	0.96	-0.01563	0.02016	0.4378898
rs1150579	6	11761465	A	G	0.654	1.00	0.117	0.041	0.004532	0.0006081		0.714	0.99	-0.01341	0.01992	0.5005387
rs863699	6	11762081	A	G	0.655	1.00	0.117	0.041	0.00447	0.0006002		0.715	0.99	-0.01339	0.0199	0.5007526
rs1150584	6	11766154	A	G	0.659	1.00	0.121	0.041	0.003259	0.000391		0.716	1.00	-0.01334	0.01985	0.5011454
rs1206003	6	11778115	A	T	0.662	0.98	0.123	0.042	0.003242	0.0003597		0.719	0.97	-0.01243	0.02021	0.5382311
rs1413343	6	11808227	C	T	0.655	0.95	0.106	0.042	0.01144	0.000888		0.719	0.96	-0.01246	0.02036	0.5402379
rs11751539	6	13706535	A	T	0.914	0.94	0.231	0.072	0.001392	0.0008167		0.881	0.78	-0.02572	0.03228	0.425218
rs421172	6	16509776	A	G	0.026	0.77	-0.497	0.142	0.0004809	0.0057275		0.038	0.74	-0.03344	0.06122	0.5846684
rs395065	6	16512559	A	T	0.025	0.76	-0.528	0.149	0.0003743	0.005716		0.038	0.73	-0.03528	0.06183	0.5679445
rs6915772	6	16838593	C	T	0.976	0.57	-0.371	0.169	0.02797	0.0008485		0.986	0.28	0.053914	0.15492	0.7275997
rs16878970	6	16851888	A	G	0.930	1.00	-0.216	0.075	0.004034	0.0003679		0.932	0.99	0.030534	0.03766	0.417055
rs10456788	6	16858835	C	G	0.070	1.00	0.214	0.075	0.004428	0.0004231		0.068	1.00	-0.03202	0.03765	0.3946646
rs10456789	6	16859046	C	T	0.070	1.00	0.214	0.075	0.004388	0.0004179		0.068	1.00	-0.03245	0.03765	0.3884281
rs10456790	6	16860772	C	T	0.070	1.00	0.214	0.075	0.00436	0.0004145		0.068	1.00	-0.03258	0.03765	0.3864784
rs12526840	6	17914727	A	G	0.442	0.99	-0.132	0.04	0.00097	0.0021366		0.514	0.98	0.014852	0.01854	0.4226085
rs6918261	6	17923410	A	G	0.391	1.00	-0.137	0.041	0.0007437	0.0007437		0.463	1.00	0.010297	0.01835	0.5744362
rs676754	6	17958016	A	G	0.426	0.98	-0.136	0.04	0.0007307	0.0013132		0.504	0.96	0.011845	0.01868	0.5256277
rs2064187	6	17987197	A	T	0.620	1.00	0.151	0.04	0.0001753	0.0002413		0.527	0.89	-0.02015	0.01934	0.2970746
rs9367972	6	17989196	A	G	0.620	1.00	0.151	0.04	0.0001848	0.0002437		0.528	0.89	-0.02018	0.01934	0.2964513
rs724915	6	17993330	A	G	0.619	1.00	0.15	0.04	0.0001919	0.0002313		0.528	0.89	-0.02019	0.01934	0.2961833
rs1014536	6	18006894	A	T	0.815	1.00	0.158	0.049	0.001349	0.0007975		0.725	0.99	-0.00762	0.02092	0.7154245
rs1014535	6	18007625	A	G	0.775	0.72	0.198	0.055	0.000313	0.0001571		0.695	0.70	-0.0057	0.02424	0.813946
rs1014534	6	18007675	A	G	0.815	1.00	0.158	0.049	0.001348	0.0007953		0.725	0.99	-0.0077	0.02092	0.7123905
rs9477555	6	18011399	A	G	0.185	1.00	-0.158	0.049	0.001346	0.0007924		0.275	0.99	0.007723	0.02092	0.711717
rs9297052	6	18027542	C	T	0.813	1.00	0.154	0.049	0.001759	0.0009652		0.735	1.00	-0.00887	0.02099	0.6721418
rs9396816	6	18027569	A	G	0.187	1.00	-0.154	0.049	0.001738	0.0009509		0.265	1.00	0.008833	0.02099	0.6735929
rs1983573	6	18029601	C	G	0.884	0.78	0.221	0.069	0.001278	0.0004336		0.858	0.72	-0.01123	0.03067	0.7141348
rs6924302	6	18032821	A	G	0.187	1.00	-0.154	0.049	0.00166	0.0008973		0.265	1.00	0.008571	0.02099	0.6828089
rs742282	6	18035992	C	T	0.813	1.00	0.155	0.049	0.001613	0.0008713		0.735	1.00	-0.00844	0.02099	0.6873931
rs7757011	6	18036468	A	G	0.813	1.00	0.155	0.049	0.001603	0.0008677		0.735	1.00	-0.0084	0.02099	0.68892
rs6459580	6	18042358	A	T	0.187	1.00	-0.155	0.049	0.001579	0.0008591		0.265	1.00	0.008163	0.02099	0.697165
rs7760740	6	18042494	C	G	0.813	1.00	0.155	0.049	0.001574	0.0008574		0.735	1.00	-0.0081	0.02099	0.6993327
rs4712321	6	18043397	C	T	0.187	1.00	-0.155	0.049	0.001555	0.0008507		0.265	1.00	0.007976	0.02099	0.7037814
rs6937334	6	18043983	C	T	0.187	1.00	-0.155	0.049	0.00155	0.000849		0.265	1.00	0.007909	0.02099	0.7061312
rs6459581	6	18046560	C	T	0.187	1.00	-0.156	0.049	0.001532	0.0008424		0.265	1.00	0.007816	0.02099	0.7094509
rs2876466	6	18059935	C	T	0.187	1.00	-0.156	0.049	0.001456	0.0008064		0.265	1.00	0.007411	0.02099	0.7238846
rs2744057	6	18081947	C	T	0.813	1.00	0.158	0.049	0.001316	0.000725		0.735	1.00	-0.00712	0.02099	0.7344465
rs2328176	6	18082613	A	G	0.813	1.00	0.158	0.049	0.00131	0.000721		0.735	1.00	-0.00707	0.021	0.7362836
rs2050029	6	18100507	G	T	0.796	0.96	0.15	0.048	0.001941	0.0006761		0.717	0.96	-0.00564	0.02098	0.7877647
rs11965998	6	18117930	G	T	0.207	0.94	-0.152	0.049	0.001771	0.0006391		0.286	0.94	0.00524	0.02113	0.8039697
rs4716212	6	18143820	C	T	0.844	0.88	0.181	0.057	0.001333	0.0003302		0.783	0.90	0.019276	0.02388	0.4192655
rs9465092	6	18149635	A	G	0.149	1.00	-0.16	0.054	0.003086	0.000915		0.204	1.00	-0.01735	0.02303	0.4509301
rs20510	6	18152751	A	G	0.149	1.00	-0.159	0.054	0.003154	0.0009321		0.204	1.00	-0.01738	0.02301	0.4497888
rs5008499	6	18153157	C	T	0.852	0.99	0.175	0.055	0.001336	0.0006415		0.796	1.00	0.017343	0.02302	0.4508341
rs4716213	6	18160428	C	T	0.148	1.00	-0.177	0.054	0.001138	0.0006243		0.204	0.99	-0.01709	0.02307	0.4584117
rs7754358	6	18197411	A	C	0.981	0.97	-0.41	0.145	0.004645	0.0009458		0.989	0.76	-0.06984	0.09585	0.4658578
rs2056954	6	20000565	C	T	0.847	0.75	-0.211	0.062	0.0006479	0.0017443		0.839	0.70	0.04368	0.03041	0.1505509
rs2523851	6	31132691	C	T	0.882	0.96	0.231	0.062	0.000188	0.0002872		0.762	1.00	-0.01721	0.02209	0.4355256
rs2523850	6	31132727	C	T	0.873	0.96	0.214	0.06	0.0003544	0.0003768		0.756	1.00	-0.02549	0.02185	0.2430321
rs2523849	6	31133030	C	T	0.133	1.00	-0.21	0.058	0.0002761	0.0001409		0.245	1.00	0.025537	0.02183	0.2417002
rs2523848	6	31133083	A	G	0.133	1.00	-0.21	0.058	0.0002761	0.0001409		0.245	1.00	0.025538	0.02183	0.2417226
rs2523845	6	31135372	A	C	0.870	1.00	0.216	0.058	0.0001949	0.0001037		0.759	0.99	-0.0235	0.02202	0.2855805
rs2428514	6	31135495	A	G	0.108	1.00	-0.22	0.063	0.0004724	0.0018955		0.201	1.00	0.02083	0.02353	0.3756556
rs2517512	6	31137664	C	T	0.892	1.00	0.22	0.063	0.0004724	0.0018955		0.800	1.00	-0.02084	0.02353	0.3755312
rs2523841	6	31138262	A	G	0.130	1.00	-0.216	0.058	0.0001949	0.0001037		0.242	0.99	0.023509	0.02203	0.2854474
rs2523840	6	31138404	A	G	0.130	1.00	-0.216	0.058	0.0001949	0.0001037		0.242	0.99	0.023511	0.02203	0.2854073
rs1634746	6	31390756	C	T	0.369	0.79	-0.13	0.045	0.003845	0.0006921		0.357	0.79	0.029801	0.02169	0.1690824
rs3130048	6	31721718	C	T	0.279	1.00	0.118	0.043	0.005979	0.0004393		0.320	0.83	0.024005	0.02089	0.2500597
rs3129944	6	32448850	C	G	0.755	0.98	-0.122	0.046	0.007566	0.0001215		0.821	0.89	-0.0068	0.02457	0.781899
rs3117099	6	32466248	A	G	0.246	1.00	0.122	0.045	0.006799	0.0001047		0.176	0.97	0.004635	0.02383	0.8456328
rs3117097	6	32466667	A	G	0.808	1.00	-0.125	0.05	0.01218	0.0006696		0.876	1.00	-0.02606	0.02673	0.3290721
rs3129953	6	32469799	C	T	0.808	1.00	-0.125	0.05	0.01208	0.0006664		0.876	1.00	-0.02605	0.02673	0.3294751
rs1980493	6	32471193	C	T	0.192	1.00	0.125	0.05	0.01208	0.0006664		0.124	1.00	0.026045	0.02673	0.3294972
rs3129959	6	32483774	A	T	0.808	1.00	-0.125	0.05	0.01262	0.0007261		0.876	1.00	-0.0263	0.02674	0.3248798
rs2071541	6	32920836	A	G	0.851	1.00	-0.167	0.055	0.002558	0.0003621		0.894	1.00	-0.06199	0.02821	0.0278964
rs1057373	6	32921257	A	C	0.112	1.00	0.163	0.062	0.008344	0.0006459		0.069	1.00	0.055994	0.03337	0.0931556
rs4711312	6	32922637	A	G	0.842	1.00	-0.134	0.054	0.01262	0.0007494		0.874	0.99	-0.05324	0.02646	0.0440287
rs1431403	6	33155009	C	T	0.271	1.00	0.138	0.044	0.001642	0.0002015		0.256	0.90	-0.00871	0.02182	0.6896486

					GOYA QC		GOYA Overweight/control			GOYA BMI continuous	known	IARC QC		IARC results		
MARKER	chr	position	A1	A2	FREQ1	Rsqr	Beta	SE	p	p	gene name	freq1	Rsqr	in_beta	in_SE	in_p
rs928976	6	33157189	C	T	0.727	0.99	-0.138	0.044	0.001621	0.0001731		0.745	0.90	0.008796	0.0219	0.687749
rs9277359	6	33158002	A	C	0.276	0.99	0.141	0.044	0.001329	0.0001199		0.268	0.86	-0.01341	0.02215	0.5446315
rs9277366	6	33158085	A	G	0.262	0.97	0.14	0.045	0.00179	0.0001041		0.260	0.84	-0.01761	0.02255	0.4345443
rs9277377	6	33158181	C	G	0.751	0.98	-0.143	0.045	0.001706	0.0000705		0.755	0.84	0.010818	0.02319	0.6405497
rs9277378	6	33158257	A	G	0.724	0.99	-0.141	0.044	0.001311	0.000117		0.747	0.90	0.008388	0.02196	0.7022333
rs9277380	6	33158347	C	G	0.747	0.98	-0.146	0.045	0.001114	0.0000641		0.749	0.85	0.018482	0.02264	0.4139354
rs9277394	6	33158948	A	T	0.714	1.00	-0.148	0.043	0.0005637	0.0000434		0.736	0.91	0.010266	0.02163	0.6347337
rs9277395	6	33159029	A	G	0.714	1.00	-0.148	0.043	0.0005622	0.0000433		0.736	0.91	0.010252	0.02163	0.6351767
rs9277396	6	33159117	A	G	0.286	1.00	0.148	0.043	0.0005611	0.0000431		0.264	0.91	-0.01026	0.02163	0.6348643
rs9277409	6	33159602	C	G	0.714	1.00	-0.148	0.043	0.0005612	0.0000431		0.736	0.91	0.010274	0.02162	0.6343896
rs9277410	6	33159618	A	G	0.286	1.00	0.148	0.043	0.0005612	0.0000431		0.264	0.92	-0.01027	0.02162	0.6343267
rs9277412	6	33159667	C	T	0.717	0.99	-0.149	0.043	0.0005995	0.0000488		0.739	0.90	0.009957	0.0219	0.6490847
rs9277421	6	33159798	A	G	0.286	1.00	0.148	0.043	0.0005613	0.0000431		0.264	0.92	-0.01027	0.02161	0.6344751
rs9277424	6	33159843	A	C	0.714	1.00	-0.148	0.043	0.0005611	0.0000431		0.736	0.92	0.010274	0.02161	0.6341373
rs9277426	6	33159888	A	C	0.286	1.00	0.148	0.043	0.0005632	0.0000432		0.248	0.97	-0.00535	0.02137	0.802004
rs9277431	6	33160006	C	T	0.714	1.00	-0.148	0.043	0.000561	0.0000431		0.737	0.92	0.010228	0.02155	0.6347232
rs9277432	6	33160050	A	T	0.714	1.00	-0.148	0.043	0.0005631	0.0000432		0.754	0.98	0.00529	0.02127	0.8034734
rs9277434	6	33160164	A	G	0.714	1.00	-0.148	0.043	0.000563	0.0000432		0.754	0.98	0.005288	0.02127	0.8035156
rs9277437	6	33160228	A	G	0.714	1.00	-0.148	0.043	0.0005629	0.0000432		0.754	0.98	0.005274	0.02126	0.8039547
rs9277450	6	33160673	G	T	0.286	1.00	0.148	0.043	0.000563	0.0000432		0.246	0.98	-0.00526	0.02126	0.8044537
rs9277458	6	33161145	A	C	0.714	1.00	-0.148	0.043	0.0005613	0.0000431		0.739	0.94	0.010093	0.02143	0.6374415
rs9277460	6	33161230	A	G	0.286	1.00	0.148	0.043	0.0005628	0.0000432		0.246	0.98	-0.00528	0.02124	0.8035764
rs9277463	6	33161285	C	T	0.714	1.00	-0.148	0.043	0.0005628	0.0000432		0.754	0.98	0.005282	0.02124	0.8034273
rs9277468	6	33161433	C	T	0.714	1.00	-0.148	0.043	0.0005628	0.0000432		0.754	0.98	0.005262	0.02123	0.8041101
rs9277469	6	33161446	G	T	0.714	1.00	-0.148	0.043	0.0005628	0.0000432		0.754	0.98	0.005255	0.02123	0.8043346
rs9277470	6	33161455	C	G	0.286	1.00	0.148	0.043	0.0005628	0.0000432		0.246	0.98	-0.00524	0.02123	0.8049883
rs9277477	6	33161750	A	G	0.286	1.00	0.148	0.043	0.0005627	0.0000432		0.245	0.99	-0.00526	0.02122	0.8041418
rs9277479	6	33161767	A	G	0.714	1.00	-0.148	0.043	0.0005627	0.0000432		0.755	0.99	0.005245	0.02121	0.8045779
rs9277492	6	33161960	C	G	0.286	1.00	0.148	0.043	0.0005626	0.0000432		0.245	0.99	-0.00524	0.0212	0.8045176
rs9277497	6	33162069	C	T	0.714	1.00	-0.148	0.043	0.0005626	0.0000432		0.755	0.99	0.005228	0.02119	0.8049874
rs9277515	6	33162246	A	C	0.286	1.00	0.148	0.043	0.0005626	0.0000432		0.245	0.99	-0.00522	0.02118	0.8051292
rs9277518	6	33162280	C	T	0.286	1.00	0.148	0.043	0.000561	0.0000431		0.261	0.94	-0.01003	0.02136	0.6382061
rs1042448	6	33162320	A	T	0.286	1.00	0.148	0.043	0.0005626	0.0000432		0.245	0.99	-0.00522	0.02117	0.8048947
rs1042544	6	33162435	A	G	0.714	1.00	-0.148	0.043	0.0005626	0.0000432		0.755	0.99	0.005216	0.02116	0.8050706
rs929	6	33162597	A	G	0.286	1.00	0.148	0.043	0.0005625	0.0000432		0.245	0.99	-0.00522	0.02115	0.8049871
rs9277533	6	33162699	C	T	0.714	1.00	-0.148	0.043	0.0005625	0.0000432		0.756	0.99	0.005202	0.02114	0.8054363
rs9277534	6	33162785	A	G	0.714	1.00	-0.148	0.043	0.0005624	0.0000432		0.756	1.00	0.005197	0.02112	0.8054622
rs9277538	6	33163025	A	G	0.714	1.00	-0.148	0.043	0.0005625	0.0000432		0.756	1.00	0.005186	0.02111	0.8057708
rs9277540	6	33163101	A	G	0.714	1.00	-0.148	0.043	0.0005627	0.0000432		0.756	1.00	0.005197	0.02111	0.8053542
rs9277541	6	33163136	A	G	0.714	1.00	-0.148	0.043	0.0005627	0.0000432		0.756	1.00	0.005201	0.02111	0.8052123
rs9277542	6	33163225	C	T	0.286	1.00	0.148	0.043	0.0005627	0.0000432		0.244	1.00	-0.0052	0.02111	0.8051425
rs9277545	6	33163301	C	T	0.740	1.00	-0.149	0.044	0.0007629	0.0000267		0.779	0.99	0.004765	0.02202	0.8285488
rs9277546	6	33163324	G	T	0.286	1.00	0.148	0.043	0.0005628	0.0000432		0.244	1.00	-0.00521	0.02111	0.8048827
rs9277547	6	33163345	A	C	0.286	1.00	0.148	0.043	0.0005629	0.0000432		0.244	1.00	-0.00521	0.02111	0.8048777
rs9277548	6	33163368	C	T	0.714	1.00	-0.148	0.043	0.000563	0.0000432		0.756	1.00	0.005211	0.02111	0.8048139
rs9277553	6	33163494	C	T	0.286	1.00	0.148	0.043	0.000563	0.0000432		0.244	1.00	-0.00521	0.02111	0.8047019
rs9277554	6	33163516	C	T	0.714	1.00	-0.148	0.043	0.0005631	0.0000432		0.756	1.00	0.005218	0.02111	0.8045548
rs9277555	6	33163583	A	G	0.286	1.00	0.146	0.043	0.0006438	0.0000506		0.244	1.00	-0.00528	0.02111	0.8021889
rs3128963	6	33163758	C	T	0.714	1.00	-0.146	0.043	0.0006444	0.0000506		0.756	1.00	0.005347	0.02112	0.7999873
rs3117228	6	33164413	G	T	0.714	1.00	-0.146	0.043	0.0006467	0.0000508		0.757	1.00	0.00565	0.02115	0.7892045
rs9277566	6	33164894	G	T	0.741	1.00	-0.147	0.044	0.0008869	0.0000323		0.781	1.00	0.005948	0.022	0.7867013
rs3130188	6	33165154	C	T	0.286	1.00	0.148	0.043	0.0005883	0.0000459		0.242	0.99	-0.00644	0.02121	0.7611954
rs3091282	6	33165176	C	G	0.286	1.00	0.148	0.043	0.0005829	0.0000454		0.242	0.99	-0.00646	0.02121	0.7604558
rs3091283	6	33165191	C	T	0.741	1.00	-0.149	0.044	0.0007948	0.0000285		0.781	1.00	0.006241	0.02201	0.7765962
rs3091284	6	33165222	G	T	0.253	1.00	0.147	0.045	0.001043	0.0000333		0.213	1.00	-0.00521	0.02219	0.8142877
rs3117226	6	33165637	A	G	0.253	1.00	0.147	0.045	0.001007	0.000032		0.213	1.00	-0.00532	0.02219	0.8103508
rs3117225	6	33165689	A	G	0.286	1.00	0.149	0.043	0.0005378	0.0000416		0.242	0.99	-0.00668	0.02122	0.7528066
rs3097652	6	33165813	C	T	0.714	1.00	-0.149	0.043	0.0005284	0.0000409		0.758	0.99	0.006701	0.02122	0.7519312
rs1367730	6	33166092	C	T	0.740	1.00	-0.151	0.044	0.0006593	0.0000231		0.780	1.00	0.00663	0.02202	0.7631691
rs3128972	6	33166752	C	T	0.260	1.00	0.151	0.044	0.0006421	0.0000225		0.220	1.00	-0.0067	0.02202	0.7606748
rs2179920	6	33166852	C	T	0.777	1.00	-0.149	0.046	0.001282	0.000023		0.814	1.00	0.000644	0.02317	0.9778099
rs2179919	6	33167240	C	T	0.260	1.00	0.152	0.044	0.000616	0.0000214		0.220	1.00	-0.00681	0.02202	0.7570016
rs2395313	6	33167314	A	C	0.740	1.00	-0.152	0.044	0.0006118	0.0000213		0.781	1.00	0.00682	0.02202	0.7565867
rs3117224	6	33167603	C	G	0.182	0.95	0.15	0.051	0.003375	0.0001548		0.133	0.96	0.008672	0.02634	0.7417918
rs3128917	6	33167974	G	T	0.260	1.00	0.155	0.044	0.0004682	0.0000156		0.220	1.00	-0.00687	0.02202	0.7550176
rs3117223	6	33168042	A	G	0.260	1.00	0.155	0.044	0.000468	0.0000156		0.220	1.00	-0.00687	0.02202	0.7550176
rs2295118	6	33168870	C	T	0.713	1.00	-0.154	0.043	0.0003406	0.0000252		0.758	0.99	0.006889	0.02122	0.7452812
rs3117222	6	33168927	C	T	0.740	1.00	-0.155	0.044	0.0004668	0.0000155		0.781	1.00	0.006866	0.02202	0.7550176
rs3128919	6	33169604	A	T	0.261	1.00	0.157	0.044	0.0003851	0.0000125		0.220	1.00	-0.00684	0.02202	0.7558654
rs3130190	6	33169668	C	T	0.301	0.86	0.168	0.046	0.0002283	0.00000504		0.281	0.80	-0.01094	0.02277	0.6307516
rs3130191	6	33169849	C	T	0.262	1.00	0.162	0.044	0.0002437	0.00000768		0.221	1.00	-0.00618	0.022	0.7786008
rs2395314	6	33170651	G	T	0.738	0.98	-0.164	0.045	0.0002421	0.00000692		0.778	0.98	0.005404	0.02217	0.8072883
rs3117217	6	33171249	A	G	0.262	1.00	0.163	0.044	0.0002234	0.00000701		0.221	1.00	-0.00608	0.02199	0.7818687
rs6914651	6	33171677	A	G	0.738	1.00	-0.164	0.044	0.0002139	0.00000671		0.779	1.00	0.005933	0.02198	0.7870143
rs3117213	6	33172583	G	T	0.738	1.00	-0.164	0.044	0.0002103	0.00000659		0.779	1.00	0.005909	0.02198	0.7878326
rs2144014	6	33173791	A	G	0.262	1.00	0.164	0.044	0.0002085	0.00000654		0.222	1.00	-0.00582	0.02197	0.7907597
rs3117242	6	33177871	A	G	0.738	1.00	-0.164	0.044	0.0002067	0.00000648		0.779	1.00	0.005804	0.02197	0.79143
rs3128921	6	33178727	A	C	0.256	1.00	0.161	0.044	0.0002911	0.00000906		0.210	1.00	0.002342	0.02221	0.9159545
rs3128923	6	33179300	C	G	0.738	1.00	-0.164	0.044	0.0002014	0.00000632		0.779	1.00	0.005719	0.02196	0.7943737
rs2064478	6	33180244	C	T	0.775	1.00	-0.162	0.046	0.0004533	0.000007		0.813	1.00	-0.0012	0.0231	0.9584509
rs3130210	6	33180707	G	T	0.776	0.99	-0.165	0.047	0.0003818	0.00000536		0.814	0.99	-7E-05	0.02324	0.9976125
rs3117236	6	33181046	A	G	0.769	1.00	-0.165	0.046	0.0003082	0.00000472		0.801	1.00	0.008209	0.02278	0.7183377

					GOYA QC		GOYA Overweight/control			GOYA BMI continuous	known	IARC QC		IARC results		
MARKER	chr	position	A1	A2	FREQ1	Rsqr	Beta	SE	p	p	gene name	freq1	Rsqr	in_beta	in_SE	in_p
rs3130211	6	33181054	A	G	0.232	1.00	0.166	0.046	0.000306	0.00000467		0.199	1.00	-0.00822	0.02278	0.7181377
rs2064476	6	33181300	A	G	0.713	1.00	-0.161	0.043	0.0001737	0.0000125		0.758	0.99	0.005843	0.0213	0.7836363
rs2064475	6	33181418	A	G	0.225	1.00	0.163	0.046	0.0004141	0.00000613		0.187	1.00	0.000432	0.02308	0.9850442
rs2064474	6	33181441	A	G	0.231	1.00	0.166	0.046	0.000292	0.00000436		0.199	1.00	-0.00825	0.02278	0.7169303
rs3117234	6	33181962	A	G	0.769	1.00	-0.166	0.046	0.0002863	0.00000424		0.801	1.00	0.008291	0.02278	0.7156663
rs3128927	6	33182266	C	T	0.743	1.00	-0.161	0.044	0.0002811	0.0000106		0.781	0.98	0.008134	0.02195	0.7107862
rs3117233	6	33182326	C	T	0.225	1.00	0.164	0.046	0.0003927	0.00000567		0.187	1.00	0.000357	0.02308	0.9876629
rs3130212	6	33182367	C	G	0.231	1.00	0.167	0.046	0.0002791	0.00000408		0.199	1.00	-0.00831	0.02278	0.7148794
rs3117232	6	33182547	A	G	0.769	1.00	-0.167	0.046	0.0002756	0.00000401		0.801	1.00	0.008344	0.02278	0.7139007
rs3128928	6	33182590	A	C	0.190	0.95	0.17	0.05	0.000742	0.0000289		0.146	0.96	-0.00084	0.02565	0.9737605
rs3130213	6	33182685	A	G	0.104	0.93	0.186	0.066	0.005141	0.0002962		0.094	0.94	0.008585	0.03309	0.7951194
rs3128929	6	33182831	C	T	0.769	1.00	-0.167	0.046	0.0002621	0.00000371		0.801	1.00	0.008394	0.02278	0.712249
rs3117231	6	33182886	A	G	0.745	1.00	-0.166	0.045	0.0002029	0.00000509		0.784	1.00	0.004883	0.02213	0.8252301
rs910320	6	33183421	C	T	0.772	0.98	-0.171	0.046	0.0002315	0.00000321		0.802	0.98	0.005481	0.02306	0.8119862
rs3117230	6	33183613	A	G	0.775	1.00	-0.165	0.046	0.0003521	0.00000486		0.813	1.00	-0.00021	0.02307	0.9926851
rs3128930	6	33183644	C	T	0.744	1.00	-0.163	0.044	0.0002389	0.00000842		0.782	0.98	0.008271	0.02198	0.7065188
rs872956	6	33184068	A	T	0.231	1.00	0.168	0.046	0.0002581	0.00000353		0.199	0.98	-0.00806	0.02301	0.7260001
rs3116961	6	33232009	C	T	0.874	1.00	-0.143	0.058	0.01389	0.0006922		0.915	0.96	-0.0161	0.03189	0.613351
rs3130159	6	33232671	C	G	0.126	1.00	0.143	0.058	0.01375	0.0006827		0.085	0.96	0.01615	0.0319	0.6124047
rs3130161	6	33233836	A	C	0.874	1.00	-0.144	0.058	0.01349	0.0006636		0.914	0.94	-0.01648	0.03203	0.6066182
rs3129203	6	33235735	C	T	0.874	1.00	-0.144	0.058	0.01343	0.0006593		0.914	0.94	-0.01648	0.03203	0.6066284
rs2855438	6	33246817	A	T	0.863	0.99	-0.153	0.057	0.006879	0.0005809		0.894	0.98	-0.01389	0.03021	0.6454328
rs2855430	6	33249258	A	G	0.136	1.00	0.158	0.057	0.005289	0.0004671		0.106	1.00	0.01166	0.02995	0.6968458
rs2076312	6	33250140	A	C	0.156	1.00	0.152	0.054	0.004592	0.0005056		0.140	1.00	0.02956	0.02648	0.2639884
rs2855426	6	33251114	A	G	0.156	1.00	0.152	0.054	0.004555	0.0005021		0.140	1.00	0.029296	0.02649	0.2683015
rs2744507	6	33256856	C	T	0.864	1.00	-0.154	0.057	0.00642	0.0005926		0.894	1.00	-0.01089	0.02996	0.7159986
rs2744505	6	33261100	C	G	0.863	1.00	-0.151	0.057	0.007452	0.0006695		0.893	0.99	-0.01075	0.02996	0.7196149
rs3129200	6	33261811	A	G	0.864	1.00	-0.159	0.057	0.004922	0.000438		0.894	1.00	-0.0101	0.02996	0.7358553
rs2855459	6	33262634	A	G	0.136	1.00	0.16	0.057	0.004863	0.0004334		0.106	1.00	0.010071	0.02996	0.7365479
rs749338	6	33761426	C	T	0.546	0.96	0.102	0.04	0.009699	0.0002605		0.532	0.93	0.019151	0.01883	0.3088026
rs11759396	6	33764632	C	G	0.521	0.98	0.092	0.039	0.0178	0.0005659		0.494	0.95	0.011516	0.01864	0.5363989
rs3818521	6	33765224	C	T	0.540	0.98	0.106	0.039	0.006535	0.0002631		0.526	0.95	0.019369	0.01865	0.2986953
rs4711336	6	33767024	A	G	0.479	0.98	-0.092	0.039	0.01761	0.0005637		0.506	0.95	-0.01149	0.01863	0.5370712
rs2229642	6	33767450	C	G	0.520	0.99	0.091	0.039	0.01919	0.0006341		0.494	0.97	0.011009	0.01851	0.5516879
rs2296742	6	33767771	A	G	0.480	1.00	-0.094	0.039	0.01504	0.0005669		0.512	0.98	-0.0118	0.01838	0.520469
rs4711338	6	33768166	C	T	0.480	1.00	-0.093	0.039	0.01616	0.0006192		0.506	0.97	-0.01081	0.01844	0.5574063
rs3818523	6	33768349	A	G	0.480	1.00	-0.094	0.039	0.01505	0.0005677		0.513	0.99	-0.01154	0.01831	0.5281017
rs3818527	6	33769013	A	G	0.461	1.00	-0.111	0.039	0.004237	0.0002219		0.486	0.99	-0.02003	0.01828	0.2729283
rs3818530	6	33769200	C	G	0.540	1.00	0.111	0.039	0.004245	0.0002038		0.519	1.00	0.019923	0.01829	0.275703
rs3227	6	33770273	C	G	0.468	0.95	-0.114	0.04	0.004148	0.0002393		0.498	0.89	-0.01168	0.01935	0.545639
rs4713658	6	33775095	C	G	0.520	1.00	0.092	0.039	0.0175	0.0006501		0.487	1.00	0.011455	0.01828	0.5306472
rs4711339	6	33775177	A	G	0.539	1.00	0.109	0.039	0.004945	0.0002509		0.514	1.00	0.019924	0.01826	0.2749487
rs9469566	6	33775583	A	C	0.460	1.00	-0.109	0.039	0.005027	0.0002348		0.481	1.00	-0.01991	0.01829	0.2761359
rs4713659	6	33775817	A	G	0.540	1.00	0.109	0.039	0.005046	0.0002356		0.519	1.00	0.019906	0.01829	0.2761278
rs942643	6	33776348	A	G	0.540	1.00	0.108	0.039	0.005247	0.0002638		0.513	1.00	0.019772	0.01825	0.2783558
rs7739505	6	33778348	A	G	0.461	1.00	-0.108	0.039	0.005267	0.0002647		0.487	1.00	-0.01978	0.01825	0.2782352
rs7759668	6	33778384	C	T	0.461	1.00	-0.108	0.039	0.00527	0.0002648		0.487	1.00	-0.01977	0.01825	0.2783211
rs6457739	6	33781809	A	G	0.540	1.00	0.107	0.039	0.005715	0.0002663		0.518	1.00	0.019723	0.01828	0.2801875
rs7770465	6	33782134	C	G	0.460	1.00	-0.107	0.039	0.005704	0.0002885		0.486	1.00	-0.01961	0.01825	0.2823487
rs4713661	6	33782152	A	C	0.460	1.00	-0.107	0.039	0.005777	0.0002926		0.486	1.00	-0.01956	0.01825	0.2833525
rs4713662	6	33782166	A	G	0.540	1.00	0.107	0.039	0.005853	0.0002968		0.514	1.00	0.019542	0.01825	0.2838885
rs2281829	6	33783620	A	G	0.540	1.00	0.106	0.039	0.006177	0.0002903		0.519	1.00	0.019593	0.01828	0.2833148
rs6904816	6	33784134	A	G	0.460	1.00	-0.107	0.039	0.006049	0.0003078		0.486	1.00	-0.01958	0.01825	0.2830202
rs6928010	6	33784568	C	T	0.460	1.00	-0.106	0.039	0.006106	0.0002863		0.481	1.00	-0.01996	0.01828	0.274488
rs6916949	6	33786268	C	G	0.540	1.00	0.107	0.039	0.005742	0.0003		0.514	1.00	0.020446	0.01826	0.2623716
rs6917140	6	33786372	C	G	0.540	1.00	0.108	0.039	0.005593	0.0002985		0.514	1.00	0.020541	0.01826	0.2601553
rs4713667	6	33787994	C	G	0.460	1.00	-0.108	0.039	0.005231	0.0002991		0.486	1.00	-0.02082	0.01826	0.2537534
rs9394163	6	33790392	C	T	0.460	1.00	-0.108	0.039	0.005253	0.0003038		0.486	1.00	-0.02079	0.01826	0.2545404
rs2966	6	33797498	C	T	0.540	1.00	0.108	0.039	0.005349	0.000314		0.515	1.00	0.020792	0.01826	0.2544197
rs10947434	6	33799479	G	T	0.541	1.00	0.108	0.039	0.005542	0.0002955		0.519	1.00	0.021019	0.01829	0.2500919
rs4713670	6	33807860	C	T	0.460	1.00	-0.113	0.039	0.003775	0.0002404		0.488	0.98	-0.02025	0.01837	0.269905
rs4713671	6	33807877	C	T	0.471	0.99	-0.11	0.039	0.004679	0.0002808		0.510	0.96	-0.01251	0.01853	0.4993174
rs791902	6	33810595	A	G	0.518	0.97	0.102	0.039	0.00903	0.0006155		0.478	0.96	0.025831	0.01857	0.1639479
rs791903	6	33810623	C	G	0.516	0.95	0.138	0.04	0.0004812	0.00000501		0.481	0.92	0.02231	0.01898	0.2395042
rs4713672	6	33813044	A	G	0.472	0.98	-0.107	0.039	0.006503	0.0003526		0.514	0.98	-0.02222	0.01845	0.2280424
rs560111	6	33814255	C	T	0.521	0.98	0.11	0.039	0.004983	0.00022		0.475	0.96	0.024303	0.01854	0.1896441
rs4713674	6	33816147	A	G	0.528	0.98	0.108	0.039	0.00611	0.0003273		0.486	0.98	0.022168	0.01845	0.2290968
rs4711343	6	33822526	A	G	0.472	0.98	-0.108	0.039	0.005931	0.0003152		0.514	0.98	-0.02205	0.01844	0.2314856
rs9469583	6	33825748	C	T	0.544	1.00	-0.1	0.039	0.01035	0.0001545		0.596	1.00	0.006142	0.01865	0.7417575
rs10947435	6	33826053	A	G	0.467	1.00	-0.111	0.039	0.004447	0.0001999		0.514	0.99	-0.02239	0.0184	0.2231388
rs6919321	6	33831982	A	G	0.608	1.00	-0.106	0.04	0.007973	0.0007749		0.620	1.00	-0.00703	0.01871	0.7069743
rs12189570	6	37691326	A	T	0.040	0.35	0.517	0.17	0.002415	0.0005317		0.039	0.29	-0.06415	0.08099	0.4279542
rs804857	6	37720604	C	T	0.981	0.80	0.534	0.163	0.001083	0.000836		0.983	0.58	0.022883	0.09031	0.7998
rs804858	6	37721570	A	G	0.019	0.80	-0.534	0.163	0.001084	0.0008369		0.017	0.58	-0.02281	0.09028	0.8003539
rs7738064	6	45901581	A	T	0.983	0.51	0.813	0.223	0.000267	0.0000868		0.982	0.48	0.055786	0.10559	0.596974
rs9463130	6	45917828	C	T	0.984	0.53	0.832	0.225	0.0002099	0.0000785		0.982	0.50	0.029578	0.1042	0.7763357
rs9463132	6	45918784	A	G	0.984	0.53	0.833	0.225	0.0002113	0.0000793		0.982	0.50	0.029305	0.10426	0.7784616
rs4714942	6	46597167	A	G	0.734	1.00	0.144	0.044	0.0009086	0.0002091		0.769	0.90	-0.0141	0.0229	0.5377815
rs9472780	6	46615405	G	T	0.268	1.00	-0.144	0.043	0.0009606	0.0002833		0.229	0.99	0.007469	0.02174	0.7309261
rs806511	6	46623524	A	C	0.732	1.00	0.144	0.043	0.0009527	0.0002806		0.771	0.99	-0.00744	0.02173	0.7316654
rs806506	6	46628215	C	T	0.268	1.00	-0.144	0.043	0.0009344	0.0002745		0.228	1.00	0.007333	0.02168	0.7349333
rs699941	6	46634975	A	T	0.732	1.00	0.145	0.043	0.0008691	0.0002605		0.772	1.00	-0.00725	0.02168	0.7379687

					GOYA QC		GOYA Overweight/control			GOYA BMI continuous	known	IARC QC		IARC results		
MARKER	chr	position	A1	A2	FREQ1	Rsqr	Beta	SE	p	p	gene name	freq1	Rsqr	in_beta	in_SE	in_p
rs806489	6	46644374	G	T	0.732	1.00	0.145	0.043	0.000869	0.0002605		0.772	1.00	-0.00715	0.02169	0.7415019
rs806484	6	46655461	A	C	0.267	0.99	-0.144	0.044	0.0009917	0.0002822		0.228	0.99	0.007239	0.02174	0.738919
rs1727595	6	46657929	C	T	0.733	0.99	0.144	0.044	0.0009958	0.0002829		0.772	0.99	-0.00731	0.02178	0.7370471
rs737011	6	46663360	A	G	0.266	0.98	-0.144	0.044	0.0009986	0.0002776		0.228	0.98	0.007213	0.02184	0.7410054
rs7748263	6	46894142	G	T	0.623	1.00	-0.131	0.04	0.0009721	0.0027101		0.571	0.99	0.003351	0.01841	0.8554786
rs13203899	6	46998968	C	T	0.043	0.95	0.34	0.098	0.0004894	0.0048065		0.042	0.86	-4.5E-06	0.04884	0.9999259
rs12210004	6	47402984	G	T	0.942	0.84	-0.312	0.091	0.0006189	0.0040825		0.936	0.63	0.052585	0.04851	0.2779757
rs12198405	6	47404186	A	G	0.942	0.85	-0.305	0.091	0.0007598	0.0042199		0.936	0.63	0.05372	0.04851	0.2677399
rs11753524	6	47818391	C	T	0.273	0.91	0.119	0.046	0.009339	0.0008956		0.312	0.89	0.00869	0.02104	0.6792671
rs12528623	6	50741578	A	G	0.966	0.78	-0.404	0.122	0.0009429	0.0005562		0.964	0.68	-0.10869	0.05638	0.0537116
rs12528998	6	50772139	A	C	0.903	1.00	-0.222	0.065	0.0006821	0.0010311		0.872	0.90	-0.04739	0.02805	0.0909126
rs12527048	6	50774927	C	T	0.966	0.81	-0.414	0.121	0.0006319	0.000331		0.964	0.68	-0.10625	0.0557	0.0562729
rs2206277	6	50906485	C	T	0.820	0.99	-0.213	0.051	0.0000337	0.0000126		0.772	0.99	-0.04051	0.02188	0.0638874
rs987237	6	50911009	A	G	0.820	1.00	-0.215	0.051	0.0000266	0.0000103		0.771	1.00	-0.03742	0.02165	0.0836886
rs2817419	6	50920865	A	G	0.741	0.95	0.127	0.045	0.004994	0.0009669		0.747	0.93	-0.00304	0.02179	0.8891076
rs2635727	6	50928899	C	T	0.762	1.00	0.132	0.046	0.003809	0.0005541		0.769	1.00	-0.004	0.0216	0.8527719
rs4715208	6	50937430	A	G	0.239	1.00	-0.135	0.046	0.003185	0.0004812		0.231	1.00	0.004111	0.02159	0.8488564
rs734597	6	50944238	A	G	0.171	0.98	0.225	0.053	0.0000195	0.00000697		0.223	0.98	0.037106	0.02202	0.0917881
rs2207139	6	50953449	A	G	0.831	1.00	-0.219	0.052	0.0000286	0.0000103		0.776	0.99	-0.03782	0.02193	0.0843681
rs943005	6	50973779	C	T	0.831	1.00	-0.219	0.052	0.0000283	0.00001		0.777	1.00	-0.03754	0.02185	0.085498
rs12529728	6	51004589	A	G	0.831	1.00	-0.219	0.052	0.0000283	0.00001		0.777	1.00	-0.0373	0.02185	0.0876219
rs4715210	6	51005210	C	T	0.831	1.00	-0.219	0.052	0.0000283	0.00001		0.777	1.00	-0.03722	0.02185	0.0882385
rs3857596	6	51013026	C	T	0.831	1.00	-0.219	0.052	0.000028	0.00000906		0.777	1.00	-0.03694	0.02185	0.0907161
rs4715213	6	51019050	C	T	0.831	1.00	-0.219	0.052	0.0000277	0.000009		0.777	1.00	-0.0368	0.02186	0.092045
rs6904450	6	51029561	A	T	0.823	0.99	-0.206	0.052	0.0000696	0.0000236		0.768	0.99	-0.03776	0.02163	0.0807267
rs3857599	6	51046206	A	C	0.167	0.96	0.224	0.054	0.0000297	0.00000923		0.222	0.95	0.035829	0.02249	0.1108283
rs4438957	6	51253131	C	G	0.248	0.90	0.146	0.048	0.002297	0.000603		0.304	0.89	-0.02625	0.02122	0.2158395
rs4615388	6	51254979	A	T	0.248	0.90	0.145	0.048	0.002319	0.0006099		0.305	0.91	-0.02541	0.02108	0.2276139
rs13211684	6	51283445	C	T	0.396	0.96	0.121	0.04	0.002613	0.0000914		0.369	0.93	-0.03564	0.01956	0.0682618
rs13202780	6	51288351	C	T	0.305	0.96	0.115	0.043	0.007061	0.0009482		0.256	0.93	-0.04538	0.0219	0.0381473
rs2709669	6	51308885	C	T	0.560	0.97	-0.098	0.039	0.01268	0.0008362		0.584	0.95	0.034996	0.01895	0.0646244
rs2709670	6	51309216	G	T	0.554	1.00	-0.097	0.039	0.01226	0.0006011		0.575	0.98	0.035194	0.01857	0.0578331
rs2709671	6	51312363	C	T	0.446	1.00	0.097	0.039	0.01227	0.000602		0.425	0.98	-0.03521	0.01856	0.0576869
rs2244114	6	51313241	C	T	0.446	1.00	0.097	0.039	0.01228	0.0006051		0.425	0.98	-0.03524	0.01856	0.0574666
rs1409892	6	51373567	A	G	0.444	1.00	0.097	0.039	0.01296	0.0007188		0.416	1.00	-0.03714	0.01849	0.0444062
rs1409884	6	51389246	A	C	0.083	0.98	-0.188	0.071	0.008313	0.0002941		0.065	0.99	-0.03592	0.03554	0.3117375
rs2767730	6	51390004	A	T	0.917	0.98	0.189	0.071	0.008256	0.0002917		0.936	0.99	0.036018	0.03554	0.310436
rs6919930	6	51417432	G	T	0.081	1.00	-0.201	0.072	0.00503	0.0002584		0.065	0.99	-0.03649	0.03545	0.3028756
rs9474014	6	51422204	A	C	0.918	0.99	0.2	0.072	0.005268	0.0002699		0.935	0.99	0.036943	0.03536	0.2957633
rs9463692	6	51447005	A	G	0.085	1.00	-0.183	0.07	0.009331	0.0005029		0.066	1.00	-0.03778	0.03515	0.2820491
rs6902943	6	51478360	A	C	0.914	1.00	0.178	0.07	0.01065	0.0006376		0.950	0.85	0.044559	0.04311	0.3009703
rs10498788	6	51490356	C	T	0.090	1.00	-0.178	0.068	0.009131	0.0006535		0.067	0.99	-0.0391	0.03514	0.265367
rs11752336	6	51516312	C	T	0.031	0.71	-0.425	0.134	0.00149	0.0009617		0.029	0.62	0.074331	0.06634	0.2621666
rs661603	6	53478066	C	T	0.423	0.97	0.141	0.04	0.00044	0.0505054		0.405	0.93	0.01409	0.01925	0.4637611
rs542914	6	53484607	A	C	0.388	0.79	0.155	0.044	0.0004708	0.0052622		0.369	0.77	0.025972	0.02174	0.2319424
rs546726	6	53485081	C	T	0.535	1.00	-0.142	0.039	0.0002728	0.0123493		0.563	0.99	-0.01738	0.0185	0.3469645
rs634657	6	53485509	A	G	0.535	1.00	-0.142	0.039	0.000276	0.0123844		0.563	0.99	-0.01745	0.01848	0.3447289
rs572496	6	53485578	C	T	0.535	1.00	-0.142	0.039	0.000278	0.0124072		0.564	0.99	-0.01745	0.01847	0.3445085
rs574389	6	53485793	C	T	0.535	1.00	-0.141	0.039	0.0002826	0.012448		0.565	1.00	-0.01766	0.01842	0.3371806
rs648595	6	53486328	G	T	0.465	1.00	0.141	0.039	0.0002881	0.0126029		0.436	0.99	0.017687	0.01843	0.3367657
rs3736729	6	53487364	A	C	0.536	1.00	-0.141	0.039	0.0003085	0.0131554		0.565	0.99	-0.01771	0.01843	0.3363435
rs84933	6	53488349	A	G	0.464	1.00	0.139	0.039	0.0003462	0.0141153		0.436	0.99	0.017707	0.01844	0.3365098
rs512827	6	53490219	A	T	0.536	1.00	-0.139	0.039	0.0003473	0.014166		0.565	0.99	-0.01772	0.01845	0.3364761
rs600033	6	53490329	C	G	0.464	1.00	0.139	0.039	0.0003462	0.0141547		0.435	0.99	0.017736	0.01845	0.3360394
rs570818	6	53491989	A	C	0.551	0.99	-0.135	0.039	0.0006016	0.0119257		0.574	0.96	-0.01644	0.0189	0.3841277
rs13212365	6	53493043	C	T	0.552	0.99	-0.132	0.039	0.0007942	0.0128404		0.579	0.98	-0.0177	0.01875	0.344734
rs6937228	6	55293002	A	G	0.101	1.00	0.164	0.065	0.01139	0.0008388		0.072	1.00	-0.03144	0.03716	0.3971044
rs9453680	6	67059698	A	G	0.798	0.87	-0.122	0.052	0.01877	0.0009435		0.784	0.91	-0.02652	0.02259	0.2400296
rs1634222	6	67094742	C	T	0.699	0.44	0.211	0.063	0.0008935	0.0004862		0.700	0.45	0.070301	0.02984	0.0183942
rs568360	6	69597311	A	T	0.196	0.96	-0.17	0.05	0.0006413	0.0005755		0.204	0.97	0.009471	0.02317	0.6824607
rs570379	6	69597557	A	G	0.196	0.96	-0.17	0.05	0.0006438	0.0005776		0.204	0.98	0.009467	0.02316	0.6824948
rs571299	6	69597671	A	G	0.196	0.97	-0.169	0.049	0.0006583	0.0005974		0.204	0.98	0.009415	0.02315	0.6839728
rs577328	6	69598052	A	G	0.192	0.98	-0.175	0.05	0.0004385	0.0004411		0.204	0.98	0.009319	0.02312	0.6866873
rs497240	6	69599012	C	T	0.808	0.98	0.175	0.05	0.0004364	0.0004393		0.796	0.98	-0.00929	0.02312	0.6875522
rs551876	6	69602150	A	G	0.808	0.98	0.175	0.05	0.000435	0.0004385		0.796	0.98	-0.00922	0.0231	0.6896717
rs482005	6	69602514	G	T	0.190	0.99	-0.177	0.05	0.0003764	0.0003997		0.204	0.99	0.009065	0.02306	0.6939946
rs555588	6	69602576	C	T	0.810	0.99	0.177	0.05	0.0003761	0.0003997		0.796	0.99	-0.00905	0.02306	0.6945047
rs483875	6	69602717	G	T	0.190	0.99	-0.177	0.05	0.0003739	0.0003985		0.204	0.99	0.009029	0.02305	0.6950422
rs580073	6	69602942	C	T	0.810	0.99	0.177	0.05	0.0003732	0.0003984		0.796	0.99	-0.00904	0.02305	0.6947775
rs510779	6	69603360	C	T	0.190	0.99	-0.177	0.05	0.0003733	0.0003988		0.204	0.99	0.009025	0.02305	0.6951478
rs540974	6	69604363	G	T	0.190	1.00	-0.177	0.05	0.0003716	0.0003983		0.204	0.99	0.008991	0.02304	0.6961434
rs483942	6	69605924	C	T	0.190	1.00	-0.176	0.05	0.0003756	0.0004042		0.203	0.99	0.008973	0.02302	0.6965005
rs2224011	6	69611902	A	C	0.810	1.00	0.176	0.05	0.0003778	0.0004069		0.797	0.99	-0.00904	0.02302	0.6944567
rs547423	6	69621551	A	G	0.190	1.00	-0.172	0.05	0.0005283	0.0005747		0.203	1.00	0.009668	0.02299	0.6738939
rs558452	6	69646308	A	G	0.190	1.00	-0.172	0.05	0.0005245	0.0005733		0.203	1.00	0.009728	0.02297	0.671614
rs478789	6	69649257	A	G	0.190	1.00	-0.172	0.05	0.0005236	0.0005722		0.203	1.00	0.009738	0.02296	0.6711903
rs4706061	6	69653313	C	T	0.810	1.00	0.172	0.05	0.0005236	0.0005722		0.797	1.00	-0.00951	0.02297	0.6785444
rs4706712	6	69654618	A	T	0.810	1.00	0.172	0.05	0.0005236	0.0005722		0.797	1.00	-0.00943	0.02298	0.6811466
rs6906092	6	69657825	A	C	0.810	1.00	0.172	0.05	0.0005236	0.0005722		0.797	1.00	-0.00909	0.02299	0.6923986
rs1523936	6	69662403	C	T	0.190	1.00	-0.172	0.05	0.0005236	0.0005722		0.203	1.00	0.00878	0.02301	0.7025167
rs9454639	6	69662538	A	G	0.197	0.98	-0.162	0.049	0.001058	0.0007428		0.210	0.97	0.006281	0.02299	0.7845567
rs1523938	6	69665213	A	C	0.188	1.00	-0.183	0.05	0.0002354	0.0002379		0.196	0.99	0.004828	0.02342	0.8365554

					GOYA QC		GOYA Overweight/control			GOYA BMI continuous	known	IARC QC		IARC results		
MARKER	chr	position	A1	A2	FREQ1	Rsqr	Beta	SE	p	p	gene name	freq1	Rsqr	in_beta	in_SE	in_p
rs9689807	6	69668386	A	G	0.812	1.00	0.184	0.05	0.0002253	0.000229		0.804	0.99	-0.00476	0.02342	0.8388906
rs17481523	6	69858921	A	G	0.877	0.98	0.196	0.06	0.001149	0.0002174		0.869	0.98	-0.02469	0.02725	0.3644228
rs2046251	6	69870434	A	G	0.218	0.98	-0.187	0.048	0.0000828	0.0001263		0.226	0.96	0.012416	0.02223	0.5761898
rs314194	6	69879307	A	G	0.842	0.97	0.225	0.054	0.0000347	0.0000198		0.852	0.98	-0.02244	0.02605	0.3885426
rs1482327	6	69895115	A	C	0.840	1.00	0.211	0.053	0.0000726	0.0000535		0.848	1.00	-0.02563	0.02553	0.3149771
rs67807	6	69904688	A	G	0.160	1.00	-0.211	0.053	0.0000711	0.0000544		0.151	1.00	0.025247	0.02567	0.3248865
rs1912988	6	69908537	C	T	0.840	1.00	0.212	0.053	0.000067	0.0000505		0.849	1.00	-0.02676	0.02559	0.2952051
rs1912987	6	69908591	G	T	0.840	1.00	0.212	0.053	0.000067	0.0000505		0.849	1.00	-0.02681	0.02559	0.2942902
rs7739401	6	69915651	A	T	0.160	1.00	-0.212	0.053	0.0000676	0.0000501		0.151	0.99	0.027135	0.02563	0.2892755
rs9454723	6	70039823	A	G	0.275	0.99	-0.156	0.044	0.0003628	0.0023692		0.290	0.99	0.041467	0.02041	0.0420878
rs1885331	6	70064271	G	T	0.250	1.00	0.163	0.045	0.0002834	0.0002416		0.250	1.00	0.001359	0.02069	0.9475745
rs529599	6	70243907	C	G	0.060	0.93	-0.277	0.086	0.00121	0.0002333		0.085	0.82	0.010448	0.03816	0.7840747
rs12190522	6	74109476	G	T	0.048	0.64	-0.379	0.111	0.0006145	0.0083288		0.046	0.58	0.017446	0.04951	0.7243264
rs12198600	6	74110463	A	C	0.952	0.66	0.375	0.109	0.0006059	0.0082718		0.955	0.59	-0.01566	0.04912	0.7496564
rs1341278	6	81095640	G	T	0.058	1.00	0.302	0.084	0.0003212	0.0045483		0.051	1.00	-0.05767	0.04015	0.1506359
rs9352828	6	81116717	A	T	0.080	0.98	0.24	0.073	0.0009971	0.0094707		0.078	0.84	-0.03271	0.03719	0.3787285
rs9344001	6	81193020	C	G	0.800	0.83	-0.179	0.054	0.0008202	0.0048893		0.822	0.78	0.046505	0.02723	0.0874288
rs7749042	6	86041794	A	G	0.359	1.00	0.133	0.04	0.0009551	0.0031636		0.351	0.98	-0.01295	0.01976	0.5118261
rs9403208	6	100461656	C	T	0.569	1.00	0.123	0.039	0.001603	0.0008127		0.556	1.00	-0.01175	0.01844	0.5236886
rs9376547	6	100472445	C	T	0.431	1.00	-0.123	0.039	0.001583	0.0008		0.444	0.99	0.011749	0.01844	0.5237508
rs12717189	6	100513167	C	T	0.566	0.93	0.144	0.04	0.0003855	0.0002812		0.553	0.95	-0.01231	0.01882	0.5126708
rs9389906	6	100513917	A	T	0.438	0.90	-0.146	0.041	0.0003794	0.0002794		0.448	0.93	0.013536	0.01905	0.4769936
rs11155381	6	100862940	A	T	0.325	1.00	-0.137	0.042	0.001028	0.0008875		0.296	0.63	-0.02127	0.02515	0.3973685
rs3957418	6	100867130	C	T	0.668	0.76	0.148	0.047	0.001803	0.0008241		0.682	0.63	0.018561	0.02492	0.4560122
rs874476	6	100926638	A	G	0.192	0.45	-0.213	0.073	0.003337	0.0005348		0.188	0.39	-0.05491	0.03789	0.1469576
rs17241549	6	100971001	C	G	0.931	0.33	0.559	0.136	0.0000402	0.0001314		0.944	0.22	0.001111	0.08367	0.9893965
rs1414733	6	101308702	C	G	0.970	0.93	0.482	0.121	0.0000674	0.0002297		0.965	0.89	0.01897	0.04988	0.7034546
rs9390697	6	101401152	A	G	0.034	1.00	-0.367	0.11	0.0008642	0.0033822		0.042	1.00	-0.01184	0.04224	0.7790915
rs9399696	6	101402594	A	G	0.966	1.00	0.367	0.11	0.0008642	0.0033822		0.958	1.00	0.011733	0.04224	0.7810125
rs9498409	6	101406687	A	G	0.034	1.00	-0.367	0.11	0.0008642	0.0033822		0.042	1.00	-0.0116	0.04224	0.7833841
rs9485417	6	101406759	C	T	0.034	1.00	-0.373	0.11	0.0007011	0.00258		0.043	0.99	-0.00803	0.04223	0.8489917
rs3734351	6	101413852	C	T	0.966	1.00	0.367	0.11	0.0008642	0.0033822		0.958	1.00	0.011494	0.04224	0.7853273
rs10485299	6	101420222	G	T	0.028	1.00	-0.449	0.122	0.0002206	0.0007044		0.032	0.99	-0.02783	0.04965	0.574772
rs9498414	6	101429697	C	T	0.966	1.00	0.366	0.11	0.0008897	0.0034393		0.958	1.00	0.01135	0.04226	0.7880965
rs9404058	6	101429799	C	T	0.034	1.00	-0.366	0.11	0.0008919	0.0034447		0.042	1.00	-0.01134	0.04226	0.788234
rs11969893	6	101502128	A	G	0.028	1.00	-0.411	0.121	0.0006663	0.001435		0.032	0.98	-0.02628	0.04994	0.5984302
rs6936675	6	103577331	A	G	0.774	0.92	0.15	0.048	0.00179	0.0004227		0.792	0.90	-0.00728	0.02342	0.7555832
rs10223608	6	103581869	A	G	0.787	0.98	0.156	0.048	0.001047	0.0002668		0.810	0.98	-0.00967	0.02334	0.6783863
rs6900581	6	103582316	A	G	0.213	0.99	-0.157	0.048	0.0009592	0.0002498		0.190	0.98	0.009722	0.02333	0.6766363
rs9485765	6	103589312	G	T	0.212	1.00	-0.158	0.047	0.0008553	0.0002301		0.188	1.00	0.010674	0.02322	0.6455381
rs4131383	6	104422184	C	G	0.159	0.99	-0.164	0.053	0.002112	0.0004089		0.127	0.87	-0.00094	0.02825	0.97338
rs3922556	6	104422394	A	G	0.159	0.99	-0.163	0.053	0.002136	0.0004111		0.130	0.87	-0.00113	0.02807	0.9678445
rs13219521	6	104425902	C	T	0.160	1.00	-0.162	0.053	0.002164	0.0004153		0.137	0.88	-0.00143	0.0274	0.9582406
rs13204470	6	104447705	A	G	0.162	0.99	-0.164	0.053	0.001955	0.0004228		0.140	0.89	-0.00129	0.02717	0.9622423
rs13195292	6	104452420	A	G	0.162	0.99	-0.163	0.053	0.001971	0.0004377		0.140	0.89	-0.00108	0.02714	0.9681405
rs11965133	6	104470668	C	T	0.162	0.99	-0.165	0.053	0.001824	0.0004354		0.146	0.91	0.003401	0.02663	0.8983123
rs9320373	6	112071965	A	G	0.059	0.54	-0.371	0.112	0.0008716	0.0095875		0.033	0.43	0.06331	0.07009	0.3659681
rs773682	6	114127633	A	G	0.225	0.88	0.173	0.049	0.0004383	0.0022356		0.206	0.84	0.002208	0.02497	0.9294649
rs9488450	6	115112846	G	T	0.553	0.99	0.109	0.039	0.005749	0.0004264		0.536	0.98	-0.0019	0.01853	0.9183139
rs7745314	6	115133003	A	C	0.460	0.99	-0.108	0.039	0.006012	0.0006161		0.482	0.99	-0.00081	0.01836	0.9646168
rs549262	6	116860688	A	G	0.375	1.00	-0.127	0.04	0.001397	0.0004329		0.350	1.00	-0.00239	0.01866	0.897918
rs550373	6	116860843	G	T	0.605	0.99	0.129	0.04	0.001249	0.0003067		0.629	0.97	0.003995	0.0188	0.8315591
rs479454	6	116863360	A	G	0.375	1.00	-0.127	0.04	0.001466	0.0004649		0.350	1.00	-0.00246	0.01866	0.8949573
rs205924	6	118632392	C	T	0.305	0.93	0.144	0.043	0.0008832	0.0072514		0.300	0.94	0.017039	0.0205	0.405546
rs9375478	6	127274638	A	G	0.545	0.99	0.112	0.039	0.003997	0.0005982		0.546	0.95	0.002839	0.01882	0.8799885
rs1930955	6	127302190	C	G	0.550	0.97	0.12	0.039	0.00211	0.0003589		0.555	0.93	0.005383	0.01896	0.7763463
rs9385412	6	127303760	C	T	0.444	1.00	-0.107	0.039	0.005793	0.0009625		0.444	0.93	0.00193	0.019	0.9190198
rs6908100	6	127304631	A	G	0.444	1.00	-0.107	0.039	0.005763	0.0009588		0.444	0.93	0.00191	0.019	0.9198399
rs6907995	6	127304781	A	G	0.557	1.00	0.107	0.039	0.005741	0.0009568		0.556	0.93	-0.0019	0.019	0.9201632
rs1343658	6	127304968	A	G	0.557	1.00	0.107	0.039	0.005706	0.0009529		0.556	0.93	-0.00188	0.019	0.9212167
rs1930956	6	127315435	A	T	0.504	0.93	0.112	0.04	0.005166	0.0003758		0.511	0.88	-0.02367	0.01937	0.2214531
rs6906261	6	127316974	C	T	0.557	1.00	0.108	0.039	0.005598	0.0009488		0.555	0.93	-0.00168	0.01898	0.9293509
rs10872310	6	127318574	C	T	0.557	1.00	0.108	0.039	0.005594	0.0009494		0.555	0.93	-0.0016	0.01897	0.9325461
rs1930958	6	127326261	A	G	0.441	1.00	-0.111	0.039	0.004062	0.0009603		0.445	0.97	0.004928	0.01866	0.7915831
rs9401925	6	127329189	A	G	0.441	1.00	-0.112	0.039	0.003896	0.0009208		0.445	0.97	0.004878	0.01866	0.7936236
rs988693	6	127339126	C	T	0.442	0.95	-0.119	0.04	0.00282	0.0007904		0.443	0.93	0.004474	0.01914	0.8149727
rs6929547	6	127354066	C	T	0.564	0.96	0.114	0.04	0.003851	0.0009756		0.566	0.94	-0.00313	0.01902	0.8690411
rs9401933	6	127355381	A	T	0.436	0.96	-0.114	0.04	0.003842	0.0009723		0.434	0.94	0.003126	0.01903	0.8693953
rs7756698	6	127357269	A	G	0.560	0.96	0.114	0.04	0.003823	0.0008584		0.556	0.92	-0.00503	0.01921	0.7933606
rs6920351	6	127357460	C	G	0.560	0.96	0.115	0.04	0.00384	0.0008598		0.556	0.92	-0.00501	0.01923	0.7944841
rs1930941	6	127358536	A	C	0.436	0.96	-0.115	0.04	0.003865	0.0009731		0.434	0.93	0.003108	0.0191	0.8706033
rs997112	6	127363178	A	G	0.566	0.93	0.118	0.04	0.003492	0.0008712		0.567	0.88	-0.00307	0.01961	0.8754091
rs17572870	6	127366607	A	T	0.434	0.93	-0.118	0.04	0.003494	0.0008714		0.433	0.88	0.003057	0.01966	0.8763244
rs6913010	6	127369036	A	G	0.562	0.92	0.119	0.04	0.003158	0.0007582		0.561	0.84	-0.00486	0.02003	0.8080004
rs4897200	6	127369187	A	C	0.439	0.92	-0.118	0.04	0.003472	0.0007669		0.445	0.83	0.004779	0.02015	0.8123406
rs9401934	6	127371660	C	T	0.562	0.89	0.121	0.041	0.003248	0.0007607		0.560	0.83	-0.00482	0.02012	0.810298
rs12176348	6	127373235	C	T	0.443	0.89	-0.121	0.041	0.003207	0.000669		0.451	0.81	0.006699	0.02031	0.7412935
rs9375482	6	127373644	A	T	0.557	0.88	0.121	0.041	0.003224	0.0006708		0.549	0.81	-0.00669	0.02031	0.7417758
rs17651529	6	127381761	C	T	0.289	0.81	0.156	0.047	0.0008989	0.0000598		0.309	0.72	-0.04179	0.02329	0.072579
rs802741	6	128316864	G	T	0.089	1.00	-0.189	0.069	0.006264	0.00097		0.074	0.97	0.056397	0.03521	0.1089814
rs6900409	6	131889250	G	T	0.102	0.97	-0.218	0.066	0.0009225	0.0112657		0.090	0.97	-0.01743	0.03106	0.574341
rs17060362	6	131908954	A	G	0.108	0.99	-0.221	0.064	0.0005115	0.0090381		0.098	0.92	-0.02058	0.03103	0.5067236

					GOYA QC		GOYA Overweight/control			GOYA BMI continuous	known	IARC QC		IARC results		
MARKER	chr	position	A1	A2	FREQ1	Rsqr	Beta	SE	p	p	gene name	freq1	Rsqr	in_beta	in_SE	in_p
rs212764	6	133832186	A	G	0.757	1.00	-0.174	0.045	0.0001124	0.0004915		0.776	0.95	-0.04023	0.0227	0.0760849
rs212778	6	133835902	G	T	0.783	0.89	-0.188	0.049	0.0001422	0.0007285		0.802	0.84	-0.04342	0.02502	0.0824212
rs509453	6	133838137	C	T	0.762	0.99	-0.176	0.045	0.0001081	0.0005617		0.779	0.95	-0.04003	0.02273	0.0779521
rs6911807	6	133869288	A	G	0.777	0.96	-0.182	0.047	0.0001052	0.0006155		0.780	0.90	-0.03649	0.02332	0.1173207
rs9375969	6	133909495	A	G	0.233	0.98	0.167	0.046	0.0002891	0.0051783		0.236	0.82	0.029677	0.02381	0.2121711
rs4289679	6	138368048	A	G	0.155	0.34	0.288	0.092	0.001771	0.0007255		0.143	0.25	-0.0501	0.05012	0.3170542
rs9495200	6	139037155	C	T	0.025	0.66	0.51	0.154	0.0009595	0.0004579		0.034	0.64	0.082136	0.07181	0.2523313
rs3777667	6	139542435	A	G	0.034	0.99	0.316	0.108	0.003477	0.000864		0.036	0.97	-0.06315	0.0533	0.2357282
rs1952319	6	139557959	A	G	0.966	0.98	-0.324	0.108	0.002808	0.0007362		0.965	0.96	0.059227	0.0539	0.2714669
rs1952318	6	139558226	C	T	0.966	0.98	-0.324	0.108	0.002789	0.000732		0.965	0.96	0.059228	0.05391	0.27156
rs1952317	6	139558394	A	T	0.034	0.98	0.324	0.108	0.002776	0.0007295		0.035	0.96	-0.05922	0.05391	0.2716373
rs7765885	6	139560239	A	G	0.035	0.94	0.334	0.109	0.002216	0.0006164		0.035	0.95	-0.05924	0.05406	0.2727887
rs12660752	6	139560403	A	G	0.035	0.94	0.334	0.109	0.002213	0.0006159		0.035	0.95	-0.05929	0.05407	0.2724321
rs1927339	6	139561776	A	G	0.035	0.94	0.335	0.109	0.002199	0.000613		0.035	0.95	-0.05925	0.05408	0.2728685
rs12664938	6	139563976	A	G	0.965	0.94	-0.336	0.109	0.002142	0.0006006		0.965	0.95	0.059268	0.05411	0.2729503
rs6929631	6	144776012	C	G	0.264	0.86	0.148	0.047	0.001663	0.0008991		0.237	0.78	-0.05245	0.02397	0.0285126
rs9497136	6	145302882	C	T	0.021	0.92	-0.44	0.145	0.002385	0.0003829		0.025	0.78	-0.03333	0.06674	0.6172349
rs9497137	6	145303027	A	G	0.970	0.64	0.45	0.144	0.001831	0.0001902		0.967	0.59	0.033291	0.06722	0.6201378
rs7748391	6	145304315	A	G	0.980	0.92	0.439	0.145	0.002427	0.0003918		0.975	0.78	0.03293	0.06659	0.6206586
rs9497139	6	145306033	A	G	0.021	0.92	-0.438	0.145	0.002507	0.0004073		0.025	0.78	-0.03278	0.06655	0.6220276
rs9484926	6	145306251	A	G	0.980	0.92	0.437	0.145	0.002519	0.0004096		0.975	0.78	0.032723	0.06653	0.622534
rs9484927	6	145308590	C	T	0.980	0.93	0.431	0.145	0.00298	0.0005012		0.975	0.78	0.032119	0.06624	0.627441
rs9497144	6	145316664	A	T	0.020	0.93	-0.424	0.146	0.003625	0.0007303		0.027	0.80	-0.0267	0.06386	0.6756753
rs9497145	6	145316779	A	G	0.020	0.93	-0.424	0.146	0.003624	0.0007315		0.027	0.80	-0.02667	0.06384	0.675809
rs9484931	6	145318563	C	T	0.020	0.93	-0.424	0.146	0.003633	0.0007458		0.027	0.80	-0.02642	0.06371	0.6781154
rs11962423	6	145319315	A	G	0.980	0.93	0.424	0.146	0.003611	0.0007444		0.973	0.80	0.026275	0.06363	0.6794098
rs11969952	6	145319333	C	T	0.020	0.93	-0.424	0.146	0.003603	0.0007433		0.029	0.83	-0.01999	0.0603	0.7400872
rs7773726	6	145320221	A	G	0.980	0.93	0.424	0.146	0.003584	0.0007414		0.971	0.84	0.019936	0.06026	0.740568
rs7759704	6	145320267	C	T	0.020	0.93	-0.424	0.146	0.003576	0.0007407		0.029	0.84	-0.01987	0.06023	0.7412832
rs9322010	6	145322686	A	G	0.020	0.93	-0.424	0.145	0.003531	0.0007361		0.030	0.84	-0.0194	0.05995	0.7460607
rs9322011	6	145322787	A	C	0.980	0.93	0.425	0.145	0.003487	0.0007319		0.971	0.84	0.019342	0.05992	0.7466624
rs9322013	6	145322973	C	T	0.980	0.93	0.425	0.145	0.003335	0.0007206		0.970	0.84	0.019242	0.05984	0.7475904
rs9322014	6	145323022	C	T	0.020	0.93	-0.425	0.145	0.00331	0.0007188		0.030	0.84	-0.01922	0.05982	0.7478235
rs9484932	6	145323340	C	G	0.980	0.93	0.425	0.145	0.003298	0.0007189		0.970	0.84	0.01916	0.05979	0.7484336
rs7756445	6	145323725	C	T	0.980	0.93	0.426	0.145	0.003288	0.0007178		0.970	0.84	0.019027	0.05971	0.7497669
rs9497153	6	145323857	A	G	0.980	0.93	0.426	0.145	0.003281	0.0007175		0.970	0.84	0.018965	0.05967	0.750411
rs6570665	6	145324189	A	G	0.020	0.93	-0.426	0.145	0.003275	0.0007172		0.030	0.84	-0.01888	0.05964	0.7513873
rs6925292	6	145324313	A	G	0.020	0.93	-0.426	0.145	0.00326	0.0007166		0.030	0.84	-0.01884	0.05961	0.7517505
rs6925230	6	145324563	A	G	0.980	0.93	0.425	0.144	0.003152	0.0007147		0.970	0.84	0.018826	0.0596	0.7518932
rs9497155	6	145325080	C	G	0.980	0.94	0.425	0.144	0.00311	0.0007234		0.970	0.85	0.018361	0.05933	0.7567686
rs1953794	6	145328902	A	C	0.020	0.95	-0.421	0.144	0.0034	0.0008715		0.030	0.85	-0.01799	0.05908	0.7606037
rs3861426	6	148225792	A	G	0.693	0.84	-0.154	0.046	0.0007449	0.0110265		0.745	0.79	-0.02315	0.0232	0.317988
rs17077549	6	148262110	A	T	0.282	0.98	0.144	0.043	0.0008701	0.0075847		0.212	0.97	0.031241	0.02193	0.1540391
rs7758196	6	150487529	C	G	0.908	1.00	-0.226	0.067	0.0007834	0.0004305		0.914	0.99	0.016048	0.03206	0.6164027
rs630548	6	152655817	A	C	0.840	0.96	0.123	0.053	0.02029	0.0007473		0.818	0.90	-0.0098	0.02523	0.6974578
rs640698	6	152665581	A	G	0.191	0.95	-0.128	0.05	0.009903	0.0005229		0.239	0.99	0.016732	0.02183	0.4430005
rs674724	6	152665613	C	T	0.191	0.95	-0.129	0.05	0.009882	0.0005166		0.240	0.99	0.016746	0.02182	0.4424636
rs1951925	6	152666392	A	G	0.809	0.95	0.129	0.05	0.009888	0.0005126		0.761	0.97	-0.01711	0.02207	0.4379267
rs1145108	6	157284757	G	T	0.035	0.96	-0.355	0.11	0.001195	0.0001634		0.040	0.91	-0.01249	0.053	0.8135603
rs284427	6	157290918	C	T	0.947	0.99	0.3	0.087	0.0005689	0.0002575		0.942	0.89	-0.00468	0.04444	0.91605
rs9458254	6	161783500	C	T	0.873	0.99	-0.19	0.058	0.0009652	0.002068		0.864	0.88	0.015896	0.02899	0.5831005
rs7755681	6	161916360	C	T	0.438	0.97	0.143	0.039	0.0002898	0.0016235		0.445	0.96	0.006175	0.01866	0.7404289
rs2143742	6	161918396	A	T	0.601	0.98	-0.14	0.04	0.0004316	0.0019871		0.604	0.95	-0.03157	0.01903	0.0968866
rs1883875	6	161919388	C	G	0.436	0.98	0.143	0.039	0.0002704	0.001264		0.442	0.97	0.006542	0.01853	0.723786
rs992037	6	161921426	C	T	0.603	0.99	-0.14	0.04	0.0003909	0.0014834		0.604	0.97	-0.03154	0.01891	0.0949934
rs994465	6	161924721	A	G	0.602	0.99	-0.14	0.04	0.0004239	0.0019254		0.606	0.97	-0.03063	0.01884	0.1036444
rs910177	6	161926519	A	T	0.560	1.00	-0.144	0.039	0.0002286	0.0011026		0.559	0.99	-0.00515	0.01835	0.7787048
rs9295157	6	161927812	A	C	0.440	1.00	0.144	0.039	0.0002353	0.0011116		0.441	0.99	0.005118	0.01834	0.7799937
rs6927285	6	161930319	A	G	0.560	1.00	-0.143	0.039	0.0002475	0.0011403		0.559	0.99	-0.00509	0.01833	0.7810553
rs6940957	6	161932297	C	T	0.397	0.99	0.138	0.04	0.000462	0.0016211		0.391	0.98	0.031969	0.01887	0.0899778
rs9355914	6	161933501	C	T	0.582	0.99	-0.135	0.039	0.0005703	0.0015553		0.584	0.98	0.003903	0.01862	0.8338081
rs6455736	6	161934424	C	G	0.583	0.99	-0.135	0.039	0.0005762	0.0015722		0.584	0.98	0.003883	0.01862	0.83465
rs742769	6	161937164	C	T	0.603	0.99	-0.138	0.039	0.0004807	0.0016971		0.609	0.97	-0.03204	0.01888	0.0894286
rs2003713	6	161938126	C	T	0.583	0.99	-0.135	0.039	0.0006068	0.0016586		0.584	0.97	0.003831	0.01863	0.8369294
rs12055483	6	161941630	C	T	0.568	0.99	-0.144	0.039	0.0002363	0.0009273		0.572	0.97	-0.01286	0.0187	0.4914354
rs9458314	6	161942482	C	T	0.397	1.00	0.135	0.039	0.0006275	0.002688		0.389	0.97	0.032968	0.01893	0.0813942
rs6924602	6	161952077	C	T	0.436	1.00	0.144	0.039	0.00023	0.0007175		0.423	0.94	0.006603	0.01895	0.72724
rs10945759	6	161952117	G	T	0.438	0.99	0.143	0.039	0.0002558	0.0007262		0.436	0.93	0.000416	0.01897	0.9825073
rs6455737	6	161959051	C	T	0.390	1.00	0.146	0.04	0.0002767	0.0005393		0.400	0.99	-0.00646	0.01867	0.72899
rs9295158	6	161959888	C	T	0.370	1.00	0.141	0.04	0.0004755	0.0006322		0.376	1.00	-0.01632	0.01878	0.3846546
rs6455738	6	161960395	A	G	0.370	1.00	0.141	0.04	0.0004884	0.0006688		0.376	1.00	-0.01624	0.01877	0.3866473
rs6455739	6	161960450	A	G	0.368	0.99	0.141	0.04	0.0005089	0.0005779		0.376	1.00	-0.01653	0.01878	0.3784162
rs9355356	6	161961748	A	G	0.387	0.97	0.148	0.041	0.0002636	0.0004458		0.400	0.98	-0.00648	0.0188	0.7298809
rs9356338	6	165377036	C	T	0.942	0.97	0.248	0.085	0.003694	0.0005585		0.942	0.72	-0.03057	0.04661	0.5115491
rs9365863	6	165389951	A	G	0.058	0.97	-0.249	0.085	0.003604	0.0005451		0.058	0.71	0.029037	0.04674	0.5341227
rs12174875	6	165391472	A	G	0.943	1.00	0.252	0.085	0.00286	0.0004444		0.936	0.65	-0.03441	0.04602	0.4541736
rs9347986	6	165394868	C	T	0.057	1.00	-0.254	0.085	0.002726	0.0004569		0.064	0.63	0.037113	0.04717	0.4310053
rs2072900	6	165398733	A	G	0.943	1.00	0.254	0.085	0.002725	0.0004675		0.937	0.61	-0.03883	0.04782	0.4164823
rs6932001	6	166828810	G	T	0.100	0.90	-0.196	0.068	0.003931	0.0006471		0.150	0.84	0.001538	0.02814	0.9563841
rs2917756	7	2573865	A	G	0.343	1.00	0.141	0.041	0.0005509	0.002126		0.309	0.99	0.020058	0.01983	0.3114587
rs7809283	7	3106990	C	T	0.855	0.95	-0.186	0.056	0.0009725	0.000773		0.861	0.92	-0.0315	0.02762	0.2536933
rs6968867	7	3110903	C	G	0.137	0.95	0.186	0.058	0.001267	0.0008178		0.131	0.93	0.040134	0.02824	0.1548818

					GOYA QC		GOYA Overweight/control			GOYA BMI continuous	known	IARC QC		IARC results		
MARKER	chr	position	A1	A2	FREQ1	Rsqr	Beta	SE	p	p	gene name	freq1	Rsqr	in_beta	in_SE	in_p
rs4634530	7	3164393	C	T	0.568	0.54	0.187	0.053	0.0004232	0.000076		0.540	0.44	0.027601	0.02741	0.3135436
rs12702772	7	8761280	A	G	0.710	0.84	0.136	0.047	0.00361	0.0003909		0.693	0.92	0.011703	0.02072	0.571868
rs3757444	7	12691991	A	C	0.571	0.97	0.126	0.04	0.00158	0.0006267		0.524	0.90	0.001427	0.01913	0.9404723
rs9647960	7	13223023	A	G	0.861	0.89	-0.217	0.06	0.000274	0.0000729		0.867	0.89	0.02037	0.02838	0.4725903
rs7789107	7	13228834	A	T	0.868	0.99	-0.177	0.057	0.002025	0.0007553		0.870	0.99	0.012999	0.02722	0.6326934
rs13221823	7	13230378	A	T	0.864	1.00	-0.196	0.057	0.0005494	0.0001782		0.868	1.00	0.012987	0.02693	0.62932
rs7780368	7	13235440	C	T	0.137	1.00	0.195	0.057	0.0005512	0.0001756		0.132	1.00	-0.01273	0.0269	0.6358364
rs4298398	7	13355193	A	G	0.150	1.00	0.184	0.055	0.0008009	0.0047729		0.147	0.96	-0.00216	0.02591	0.9334496
rs6954033	7	13358543	C	G	0.150	1.00	0.184	0.055	0.0007853	0.0044378		0.148	0.96	-0.00226	0.02578	0.9300794
rs253429	7	21417787	C	T	0.391	0.88	0.106	0.042	0.01143	0.0009166		0.400	0.84	0.010971	0.02037	0.5898926
rs4722037	7	21553120	C	T	0.569	0.98	0.13	0.04	0.0009733	0.0061218		0.569	0.97	0.006977	0.01858	0.7070187
rs10950865	7	21647091	C	T	0.645	0.91	0.139	0.042	0.0009911	0.0093099		0.629	0.84	0.012661	0.02079	0.5422558
rs12700288	7	21649628	A	G	0.303	0.99	-0.141	0.042	0.0008473	0.0100575		0.348	0.94	-0.00474	0.01983	0.8107204
rs13234537	7	27337233	G	T	0.713	0.95	0.151	0.044	0.0005933	0.0022026		0.675	0.93	0.015887	0.02016	0.4302728
rs2189037	7	27379337	C	T	0.650	1.00	-0.142	0.041	0.0005054	0.0006623		0.632	1.00	0.007227	0.01917	0.7060002
rs6462011	7	27380180	A	G	0.351	1.00	0.141	0.041	0.0005143	0.0006718		0.368	1.00	-0.00716	0.0192	0.7090288
rs5743335	7	30465354	A	T	0.019	0.92	0.469	0.15	0.001739	0.0008323		0.017	0.78	-0.04864	0.07367	0.5087758
rs5743334	7	30465636	C	G	0.981	0.92	-0.469	0.15	0.001737	0.0008323		0.983	0.78	0.048815	0.07363	0.5069898
rs2970510	7	30521300	G	T	0.011	0.90	0.567	0.206	0.005813	0.0007339		0.008	0.83	-0.12601	0.11412	0.269137
rs2970509	7	30526871	A	C	0.011	0.91	0.564	0.206	0.006059	0.000784		0.008	0.83	-0.12449	0.11406	0.274679
rs17159526	7	30865222	C	G	0.098	0.99	-0.181	0.066	0.00618	0.0006161		0.086	0.87	0.042417	0.03236	0.1895708
rs7795447	7	30866227	A	G	0.098	0.99	-0.181	0.066	0.006395	0.0006323		0.086	0.87	0.042443	0.03239	0.1896802
rs11771371	7	31418453	A	G	0.946	0.99	-0.315	0.086	0.000264	0.0008633		0.931	0.86	-0.04538	0.03748	0.2255983
rs12674467	7	31426184	A	G	0.929	1.00	-0.268	0.075	0.0003752	0.0034672		0.893	0.87	-0.03652	0.03065	0.2331205
rs17160143	7	31428570	A	G	0.929	0.99	-0.259	0.076	0.0006129	0.0052352		0.892	0.87	-0.03648	0.03064	0.2334709
rs17160618	7	31861913	C	T	0.377	0.99	0.136	0.041	0.0008262	0.0059073		0.400	0.93	0.001979	0.01933	0.9183805
rs2191866	7	31872140	A	G	0.282	1.00	0.148	0.043	0.0006084	0.0035085		0.283	0.98	0.013565	0.02038	0.5052451
rs17170396	7	33747746	C	G	0.880	0.96	0.202	0.061	0.0009376	0.0087892		0.842	0.97	0.0213	0.02626	0.4168393
rs11974259	7	33748760	C	G	0.120	0.96	-0.201	0.061	0.0009551	0.0088593		0.159	0.98	-0.02133	0.02622	0.4155757
rs17171141	7	37722758	A	G	0.981	0.38	0.907	0.237	0.0001331	0.0008786		0.976	0.26	-0.09386	0.11471	0.412831
rs909158	7	38346755	C	T	0.804	0.85	-0.171	0.053	0.001243	0.0006246		0.848	0.78	-0.00488	0.02905	0.8665737
rs7789243	7	39407418	A	G	0.911	1.00	-0.227	0.069	0.0009338	0.0001636		0.927	1.00	0.049261	0.03421	0.1495211
rs6960454	7	42413078	A	G	0.106	0.96	-0.184	0.064	0.004155	0.0006224		0.086	0.82	0.041963	0.03568	0.2391411
rs7799125	7	44921399	C	G	0.133	0.97	0.169	0.057	0.003269	0.0009494		0.111	0.96	-0.00054	0.02955	0.9852753
rs4724332	7	44921641	A	G	0.867	1.00	-0.168	0.057	0.003132	0.0007918		0.889	0.96	0.000473	0.02946	0.9871672
rs6463252	7	44924043	A	G	0.871	0.97	-0.171	0.058	0.003393	0.000723		0.893	0.95	-0.00149	0.03037	0.9609009
rs2331175	7	44926448	A	G	0.133	1.00	0.167	0.057	0.003122	0.0007812		0.110	0.99	-0.00028	0.0292	0.9922125
rs4236376	7	44934721	A	G	0.133	0.96	0.17	0.058	0.003372	0.0009512		0.111	0.91	-0.00126	0.03036	0.9669438
rs2935265	7	45836747	C	T	0.455	0.68	-0.128	0.047	0.006568	0.0008361		0.510	0.66	-0.00124	0.02238	0.9556748
rs6944270	7	45841356	C	T	0.406	0.68	-0.147	0.047	0.001924	0.0001249		0.432	0.63	0.000869	0.02305	0.9698969
rs4999732	7	45848478	A	G	0.405	0.68	-0.147	0.047	0.001944	0.0001282		0.432	0.63	0.000522	0.02305	0.9819242
rs10951835	7	45853323	A	G	0.325	0.70	-0.156	0.049	0.001528	0.0001809		0.349	0.67	-0.00361	0.02288	0.874624
rs12673689	7	45885468	A	G	0.145	0.99	-0.182	0.055	0.0009737	0.0083765		0.146	1.00	0.036432	0.02587	0.1586863
rs10231774	7	45889002	C	G	0.146	0.98	-0.183	0.055	0.0008928	0.0074521		0.146	0.99	0.035743	0.02599	0.1687852
rs1874479	7	45898751	A	G	0.852	0.99	0.189	0.055	0.00054	0.0029911		0.853	0.99	-0.03045	0.02598	0.2408657
rs9658239	7	45900684	A	G	0.852	0.99	0.189	0.055	0.0005284	0.0028247		0.853	0.99	-0.03008	0.02595	0.2460628
rs12671457	7	45913451	A	C	0.834	1.00	0.184	0.052	0.0003707	0.0025906		0.844	1.00	-0.04063	0.02518	0.1063707
rs12671484	7	45913680	A	G	0.835	1.00	0.184	0.052	0.0003789	0.0026241		0.844	1.00	-0.04088	0.02519	0.1043834
rs13238599	7	47006266	C	T	0.217	1.00	0.153	0.046	0.000986	0.0328705		0.219	1.00	-0.01994	0.02272	0.3795913
rs1544440	7	47032306	C	G	0.217	0.99	0.154	0.047	0.0009357	0.0320902		0.219	0.99	-0.01869	0.02284	0.4129387
rs1025520	7	48462371	C	T	0.657	1.00	0.126	0.041	0.002017	0.0003647		0.667	1.00	-0.01622	0.01931	0.4006182
rs1975194	7	48468620	A	G	0.347	1.00	-0.121	0.041	0.00298	0.0006147		0.335	1.00	0.012384	0.01931	0.5208853
rs4083233	7	48469856	G	T	0.347	1.00	-0.121	0.041	0.002992	0.0006168		0.335	1.00	0.012341	0.0193	0.5222742
rs10233704	7	48473969	C	T	0.653	1.00	0.12	0.041	0.003116	0.0006529		0.666	1.00	-0.01212	0.01932	0.5303019
rs4917150	7	48476211	C	T	0.346	1.00	-0.119	0.041	0.003297	0.0007025		0.334	1.00	0.012023	0.01932	0.5334655
rs1030707	7	48479666	A	G	0.654	1.00	0.12	0.041	0.003202	0.0007034		0.667	1.00	-0.01266	0.01932	0.5119517
rs7810026	7	48481843	C	T	0.346	1.00	-0.118	0.041	0.003678	0.0008395		0.333	1.00	0.012956	0.01932	0.5020364
rs6978160	7	48727841	A	G	0.558	0.98	0.105	0.04	0.007758	0.0002297		0.524	0.99	0.034267	0.01829	0.0607936
rs11977916	7	48728667	A	T	0.558	0.98	0.106	0.039	0.007489	0.0002192		0.524	0.99	0.03431	0.01828	0.0603653
rs4557645	7	48733847	C	T	0.442	0.99	-0.106	0.039	0.007275	0.0002115		0.476	0.99	-0.03435	0.01828	0.0600172
rs10435487	7	48763382	A	T	0.445	1.00	-0.104	0.039	0.00798	0.0001987		0.476	1.00	-0.03462	0.01819	0.0568912
rs7801597	7	48765456	C	T	0.555	1.00	0.104	0.039	0.008243	0.0002048		0.524	1.00	0.034837	0.01819	0.055281
rs2363125	7	48765682	C	T	0.555	1.00	0.104	0.039	0.008243	0.0002048		0.524	1.00	0.034837	0.01819	0.055281
rs4917174	7	48769990	C	T	0.555	1.00	0.104	0.039	0.00782	0.0001861		0.524	1.00	0.034367	0.01819	0.0587244
rs7810989	7	48770140	C	T	0.445	1.00	-0.104	0.039	0.007686	0.0001803		0.476	1.00	-0.03417	0.01819	0.0601665
rs10487572	7	48775385	A	G	0.556	1.00	0.105	0.039	0.007301	0.0001642		0.524	1.00	0.033775	0.01819	0.0631864
rs2117723	7	48777751	C	T	0.555	1.00	0.105	0.039	0.007434	0.0001697		0.524	0.99	0.033509	0.01823	0.0658333
rs10487568	7	48780604	A	T	0.554	1.00	0.104	0.039	0.008063	0.000191		0.524	0.99	0.032819	0.0183	0.0727272
rs1529867	7	48785573	A	G	0.446	1.00	-0.103	0.039	0.008269	0.0001976		0.476	0.98	-0.03275	0.01831	0.073466
rs11767191	7	48788511	C	T	0.515	0.99	-0.13	0.039	0.0008206	0.0000393		0.538	0.97	-0.03417	0.0185	0.0645896
rs12670376	7	48793700	A	G	0.446	1.00	-0.103	0.039	0.008389	0.0002016		0.476	0.98	-0.03202	0.01835	0.080771
rs2164322	7	48803247	C	T	0.375	1.00	-0.101	0.04	0.01099	0.0006125		0.403	1.00	-0.01125	0.01851	0.5430506
rs6960037	7	48809123	C	G	0.626	1.00	0.102	0.04	0.01076	0.0005972		0.597	1.00	0.011162	0.01851	0.5461085
rs6979016	7	48810941	C	T	0.374	1.00	-0.102	0.04	0.01059	0.0005855		0.403	1.00	-0.011	0.0185	0.5517154
rs963161	7	50662811	A	G	0.138	0.79	0.229	0.064	0.0003206	0.0386787		0.169	0.75	-0.03169	0.02841	0.264222
rs4557653	7	51582999	A	G	0.286	1.00	-0.149	0.043	0.0004844	0.0052889		0.243	1.00	-0.0294	0.02116	0.1644066
rs12533589	7	51585846	C	T	0.714	1.00	0.149	0.043	0.0004819	0.0052226		0.757	0.99	0.029521	0.02118	0.1631407
rs4947372	7	51595499	C	T	0.715	1.00	0.152	0.043	0.0004014	0.0047401		0.760	0.98	0.028012	0.02135	0.1890597
rs12671195	7	51601068	C	G	0.718	0.98	0.151	0.043	0.0004771	0.0056778		0.764	0.97	0.030237	0.02162	0.1615454
rs1159835	7	51605077	A	G	0.283	1.00	-0.155	0.043	0.0003224	0.0042546		0.233	0.98	-0.02688	0.02164	0.2139255
rs12718513	7	51618541	C	T	0.717	1.00	0.155	0.043	0.000315	0.0041878		0.767	0.98	0.026925	0.02168	0.2138751
rs12698903	7	69475784	A	G	0.074	1.00	-0.26	0.075	0.0005335	0.0009528		0.061	0.84	0.055686	0.03975	0.1609093

					GOYA QC		GOYA Overweight/control			GOYA BMI continuous	known	IARC QC		IARC results		
MARKER	chr	position	A1	A2	FREQ1	Rsqr	Beta	SE	p	p	gene name	freq1	Rsqr	in_beta	in_SE	in_p
rs6963105	7	74935424	A	G	0.418	0.98	-0.146	0.04	0.0002473	0.0000879		0.462	0.97	-0.04015	0.01852	0.0300988
rs17207196	7	74939001	C	T	0.601	1.00	0.145	0.04	0.0002718	0.0000657		0.553	1.00	0.041821	0.0183	0.0221782
rs1167827	7	75001105	A	G	0.412	1.00	-0.139	0.04	0.0004571	0.0000925		0.455	1.00	-0.03941	0.01834	0.0315547
rs2536365	7	76322020	A	G	0.367	0.50	0.106	0.057	0.06358	0.0007338		0.352	0.24	-0.00917	0.03885	0.8132168
rs7804646	7	76405926	A	C	0.817	0.75	-0.166	0.058	0.004311	0.0006687		0.766	0.27	0.0044	0.04136	0.9152018
rs2245368	7	76446079	C	T	0.167	0.69	0.185	0.063	0.003323	0.0003431		0.207	0.25	0.010232	0.04402	0.8160546
rs2430307	7	76447880	C	T	0.833	0.68	-0.185	0.063	0.00334	0.000347		0.793	0.25	-0.01019	0.04401	0.8167455
rs17149254	7	76472399	C	T	0.832	0.67	-0.185	0.063	0.003472	0.00039		0.793	0.26	-0.0102	0.04364	0.8150175
rs4729098	7	76475006	A	G	0.832	0.67	-0.185	0.063	0.003466	0.0003902		0.793	0.26	-0.01018	0.04359	0.8151196
rs6955651	7	76477807	C	T	0.823	0.58	-0.214	0.067	0.00146	0.0001827		0.794	0.24	0.029456	0.0451	0.5133316
rs699332	7	77713346	G	T	0.128	1.00	0.189	0.057	0.0009695	0.0224485		0.077	0.92	0.024978	0.03291	0.4474371
rs2191724	7	78439555	G	T	0.735	0.98	0.154	0.045	0.0005392	0.0005965		0.730	0.80	-0.00732	0.0225	0.7448308
rs2215546	7	78447866	C	T	0.735	0.98	0.154	0.044	0.0005476	0.0006028		0.730	0.80	-0.00741	0.02238	0.7403343
rs2215547	7	78448304	A	G	0.265	0.99	-0.153	0.044	0.0005577	0.0006101		0.270	0.81	0.007416	0.02238	0.7401122
rs10259881	7	78450011	G	T	0.735	0.99	0.153	0.044	0.0005575	0.0006099		0.730	0.81	-0.00742	0.02236	0.7399666
rs12534480	7	78451446	C	T	0.265	0.99	-0.153	0.044	0.0005609	0.0006128		0.271	0.82	0.007494	0.0222	0.7354078
rs7793390	7	78452998	A	C	0.735	0.99	0.153	0.044	0.0005625	0.0006138		0.729	0.82	-0.00749	0.02218	0.7353175
rs10269689	7	78453356	A	C	0.265	1.00	-0.152	0.044	0.0005723	0.0006214		0.271	0.82	0.007523	0.02218	0.7342079
rs12537793	7	78454397	A	G	0.648	1.00	0.135	0.041	0.0009277	0.0008839		0.643	0.83	0.011473	0.02093	0.5833594
rs7809089	7	78457083	C	T	0.735	1.00	0.152	0.044	0.0005758	0.0006259		0.729	0.82	-0.00755	0.02214	0.733055
rs6466468	7	78471245	A	T	0.733	1.00	0.145	0.044	0.0009804	0.0014131		0.734	0.96	-0.00757	0.02065	0.7137135
rs1476106	7	78482013	C	T	0.268	1.00	-0.145	0.044	0.0009652	0.0014215		0.276	1.00	0.007968	0.01996	0.6894384
rs10224972	7	78497383	A	G	0.751	1.00	0.159	0.045	0.0003993	0.001499		0.728	0.96	-0.01085	0.02047	0.5958223
rs13438061	7	78499486	C	G	0.249	1.00	-0.159	0.045	0.0004242	0.0016117		0.272	0.96	0.011075	0.02046	0.5880741
rs13229394	7	78506344	G	T	0.770	0.93	0.186	0.048	0.0001006	0.0004242		0.764	0.81	-0.01279	0.02337	0.5838022
rs10231279	7	78514550	A	T	0.665	0.99	0.15	0.042	0.0003227	0.0015619		0.641	0.95	0.003985	0.01934	0.8366073
rs4437570	7	78516290	C	T	0.608	1.00	0.131	0.04	0.0009826	0.0047489		0.576	0.99	0.00529	0.01842	0.7737842
rs6466941	7	79547988	C	G	0.654	0.93	0.145	0.043	0.0006557	0.0269904		0.656	0.91	-0.00214	0.0202	0.9155427
rs6960044	7	79553200	A	G	0.653	0.94	0.143	0.042	0.0007224	0.0255177		0.655	0.92	-0.00201	0.02011	0.9204591
rs4731198	7	79556553	A	T	0.348	0.95	-0.14	0.042	0.0008368	0.0248788		0.345	0.92	0.001987	0.0201	0.9211883
rs11772324	7	82067470	C	G	0.930	0.99	0.214	0.076	0.004874	0.0009375		0.937	0.62	0.002022	0.04911	0.9671277
rs10252388	7	82074230	A	G	0.072	0.99	-0.212	0.075	0.004861	0.0009442		0.072	0.96	0.001464	0.03731	0.9686809
rs10260359	7	82088579	C	T	0.928	0.99	0.212	0.075	0.004876	0.0009485		0.928	0.98	-0.00269	0.03687	0.9418747
rs7784368	7	82551589	A	C	0.498	0.96	0.127	0.04	0.001354	0.0009537		0.512	0.97	0.013351	0.01847	0.4692952
rs4732512	7	82941991	A	G	0.126	0.99	0.175	0.058	0.002663	0.0003898		0.101	1.00	-0.00448	0.03073	0.884087
rs2255614	7	82954322	C	T	0.878	1.00	-0.182	0.059	0.001989	0.0004021		0.895	0.97	0.004436	0.03057	0.8845367
rs3801511	7	82957840	C	T	0.123	1.00	0.177	0.059	0.002558	0.0005615		0.107	0.97	-0.00678	0.0302	0.8221419
rs2535379	7	82962076	C	G	0.123	1.00	0.177	0.059	0.002564	0.0005629		0.107	0.97	-0.00685	0.0302	0.8204075
rs2535375	7	82967319	C	T	0.877	1.00	-0.177	0.059	0.00258	0.0005662		0.893	0.97	0.007419	0.03023	0.8059894
rs7791887	7	82968551	A	T	0.197	1.00	0.184	0.049	0.0001625	0.0000424		0.207	0.95	-0.00216	0.02334	0.9263139
rs2535374	7	82980627	C	G	0.137	0.96	0.178	0.057	0.001887	0.0003692		0.117	0.94	-0.00071	0.02954	0.9807939
rs2041426	7	82984970	C	G	0.140	1.00	0.188	0.056	0.0007806	0.0001141		0.128	0.99	0.004074	0.02787	0.8836937
rs2535370	7	82985641	A	G	0.204	1.00	0.169	0.048	0.000395	0.000082		0.213	0.99	0.026443	0.02249	0.239387
rs1860610	7	82987500	G	T	0.140	1.00	0.188	0.056	0.0007665	0.0001124		0.126	1.00	0.002003	0.02798	0.9428917
rs2535369	7	82988944	A	G	0.860	1.00	-0.188	0.056	0.0007668	0.0001125		0.874	1.00	-0.002	0.02799	0.9431249
rs2109543	7	82989958	A	G	0.860	1.00	-0.188	0.056	0.0007666	0.0001124		0.874	0.99	-0.002	0.028	0.9429457
rs2535364	7	82997426	C	T	0.860	0.99	-0.188	0.056	0.0007802	0.0001183		0.873	0.98	-0.00217	0.0282	0.938481
rs2713140	7	83000507	C	T	0.140	0.98	0.189	0.056	0.0008005	0.0001271		0.127	0.96	0.002493	0.02846	0.9301302
rs978582	7	83003451	A	C	0.860	0.96	-0.19	0.057	0.0008463	0.0001485		0.872	0.91	-0.00349	0.02921	0.9049258
rs1533030	7	85194990	A	T	0.200	0.74	-0.147	0.056	0.00884	0.0009722		0.212	0.70	0.002112	0.02596	0.9351215
rs1533029	7	85199723	C	T	0.791	1.00	0.129	0.048	0.006458	0.000581		0.780	1.00	-0.00294	0.02228	0.8949633
rs10240983	7	85205632	A	T	0.791	1.00	0.13	0.048	0.006333	0.000575		0.780	1.00	-0.00283	0.02229	0.8988507
rs13340496	7	85208465	C	T	0.209	1.00	-0.13	0.048	0.006301	0.0005734		0.220	1.00	0.002808	0.02229	0.8996638
rs2089596	7	85209283	G	T	0.209	1.00	-0.13	0.048	0.00629	0.0005729		0.220	1.00	0.00278	0.02229	0.9006842
rs1509079	7	85216674	C	T	0.791	1.00	0.13	0.048	0.006196	0.0005684		0.780	1.00	-0.00268	0.0223	0.9041198
rs10952857	7	85219550	A	G	0.791	1.00	0.13	0.048	0.00616	0.0005667		0.780	1.00	-0.00264	0.0223	0.905854
rs9649087	7	85220929	G	T	0.808	0.94	0.133	0.051	0.008893	0.0005527		0.795	0.94	-0.00427	0.02361	0.8563708
rs2221693	7	85225921	C	T	0.791	1.00	0.13	0.048	0.006103	0.0005598		0.780	1.00	-0.00257	0.02231	0.9081111
rs1509081	7	85226840	C	T	0.791	1.00	0.131	0.048	0.005781	0.0005219		0.780	1.00	-0.00254	0.02231	0.9091328
rs11984434	7	85238379	A	G	0.209	1.00	-0.133	0.048	0.005056	0.00044		0.220	1.00	0.002451	0.02231	0.9124678
rs1588290	7	85322791	G	T	0.716	0.93	0.125	0.044	0.004676	0.0009729		0.658	0.92	0.023363	0.02016	0.246112
rs12704197	7	85349810	C	G	0.271	0.97	-0.121	0.044	0.00581	0.0009863		0.333	0.97	-0.02032	0.01981	0.3046243
rs13244655	7	85350069	A	G	0.244	0.95	-0.124	0.046	0.006816	0.0008319		0.290	0.90	-0.01711	0.02134	0.4221893
rs13244673	7	87886980	A	G	0.791	0.99	-0.162	0.048	0.0007676	0.0037287		0.762	0.99	-0.00942	0.02145	0.6603819
rs41654	7	87892493	G	T	0.792	1.00	-0.16	0.048	0.000844	0.0039038		0.762	1.00	-0.00942	0.0214	0.6593419
rs7795474	7	87896199	C	T	0.791	0.99	-0.16	0.048	0.0008577	0.0040556		0.761	0.99	-0.00837	0.02147	0.6963328
rs7795579	7	87896221	A	T	0.791	0.99	-0.16	0.048	0.0008562	0.0040628		0.761	0.99	-0.00835	0.02147	0.697031
rs41656	7	87896467	A	T	0.213	0.97	0.162	0.048	0.0007493	0.0040046		0.243	0.98	0.008938	0.02146	0.6767231
rs13236112	7	93045809	A	G	0.079	0.99	-0.227	0.072	0.001717	0.0007173		0.049	0.99	-0.01721	0.03975	0.6648127
rs12668387	7	93046331	C	T	0.921	1.00	0.226	0.072	0.001731	0.0007032		0.951	0.99	0.01726	0.03975	0.6638446
rs17735265	7	93047142	A	C	0.079	1.00	-0.226	0.072	0.001732	0.0007022		0.049	0.99	-0.01873	0.03953	0.6353555
rs12673424	7	93072333	A	G	0.919	1.00	0.224	0.071	0.001681	0.0007697		0.949	0.99	0.028359	0.039	0.4667667
rs12669252	7	93072362	A	G	0.081	1.00	-0.224	0.071	0.001685	0.0007721		0.051	0.99	-0.02835	0.039	0.4668626
rs17736498	7	93077753	C	T	0.919	1.00	0.224	0.071	0.001709	0.0007861		0.949	0.99	0.028241	0.03897	0.4682955
rs957728	7	93090336	C	T	0.081	1.00	-0.223	0.071	0.001754	0.0008129		0.051	0.99	-0.02783	0.0389	0.4740095
rs13227315	7	93093597	C	T	0.919	1.00	0.223	0.071	0.001778	0.0008215		0.949	0.99	0.027848	0.03896	0.4744177
rs17799060	7	93094619	C	T	0.919	1.00	0.222	0.071	0.001866	0.0008558		0.949	0.98	0.027825	0.03901	0.4753216
rs12669841	7	93096373	A	G	0.919	1.00	0.222	0.071	0.001882	0.0008609		0.949	0.98	0.027819	0.03902	0.4755309
rs17737212	7	93100110	A	G	0.919	0.99	0.222	0.072	0.001946	0.000886		0.949	0.97	0.027905	0.03917	0.4758514
rs17799235	7	93100269	C	T	0.919	0.99	0.221	0.072	0.001956	0.0008892		0.949	0.97	0.027863	0.0392	0.4767985
rs17165636	7	93101449	A	C	0.919	0.99	0.221	0.072	0.001984	0.0009006		0.949	0.97	0.027871	0.03922	0.4769666
rs13245089	7	93103448	A	G	0.919	0.99	0.221	0.072	0.001992	0.0009044		0.949	0.97	0.027885	0.03923	0.4768516

					GOYA QC		GOYA Overweight/control			GOYA BMI continuous	known	IARC QC		IARC results		
MARKER	chr	position	A1	A2	FREQ1	Rsqr	Beta	SE	p	p	gene name	freq1	Rsqr	in_beta	in_SE	in_p
rs41542	7	93559972	C	T	0.917	1.00	-0.232	0.07	0.0009545	0.0008823		0.887	0.99	0.036833	0.02871	0.1991278
rs6465405	7	93590520	C	T	0.083	0.99	0.231	0.07	0.001031	0.0009042		0.112	0.95	-0.04112	0.02945	0.1623209
rs12704800	7	95011506	C	T	0.415	0.84	-0.159	0.043	0.0002264	0.0024894		0.451	0.76	0.032108	0.02153	0.1356398
rs12704801	7	95013121	C	T	0.420	0.85	-0.15	0.043	0.0004318	0.0033133		0.442	0.71	0.038952	0.02218	0.0788862
rs12111976	7	95015513	A	G	0.598	0.95	0.15	0.041	0.0002243	0.0015398		0.563	0.83	-0.03084	0.02047	0.131599
rs7801196	7	95017197	C	T	0.402	0.99	-0.145	0.04	0.0002634	0.0016593		0.442	0.84	0.030629	0.02033	0.1316669
rs6465469	7	95017529	A	G	0.402	1.00	-0.145	0.04	0.0002671	0.0016676		0.442	0.85	0.030475	0.02024	0.1319067
rs6465470	7	95017693	A	G	0.402	1.00	-0.145	0.04	0.0002551	0.0016199		0.437	0.84	0.030584	0.02035	0.132522
rs6953838	7	95017954	A	G	0.402	1.00	-0.144	0.04	0.0002776	0.0017099		0.443	0.85	0.030404	0.02021	0.1321606
rs4727315	7	95018394	A	C	0.403	0.99	-0.144	0.04	0.0002983	0.0017925		0.443	0.85	0.030343	0.02017	0.1322784
rs4727316	7	95018425	C	T	0.408	0.97	-0.147	0.04	0.0002395	0.0015074		0.441	0.83	0.029299	0.02047	0.1519824
rs4729196	7	95018782	A	G	0.409	0.99	-0.137	0.04	0.0005618	0.0027607		0.438	0.87	0.029871	0.01999	0.134839
rs4727318	7	95018824	C	T	0.591	0.99	0.136	0.04	0.0006042	0.0028966		0.557	0.89	-0.02966	0.0198	0.1337673
rs4727319	7	95019120	C	G	0.571	0.94	0.135	0.04	0.0007968	0.0030667		0.553	0.83	-0.03316	0.02042	0.1041681
rs12704802	7	95019416	C	T	0.591	0.99	0.137	0.04	0.0005729	0.0027991		0.562	0.88	-0.0296	0.01985	0.1356161
rs7785595	7	95021254	C	T	0.410	0.99	-0.136	0.04	0.0005896	0.0028544		0.438	0.89	0.029247	0.01967	0.1368461
rs7802572	7	95021392	A	G	0.590	1.00	0.135	0.04	0.0006265	0.0029779		0.562	0.90	-0.02911	0.0196	0.137212
rs2375021	7	95022599	C	G	0.411	1.00	-0.134	0.04	0.0007275	0.0033338		0.443	0.94	0.028556	0.0192	0.1365582
rs12704805	7	95023044	A	C	0.589	1.00	0.134	0.04	0.00073	0.0033441		0.557	0.95	-0.02834	0.01907	0.1370725
rs12704806	7	95023061	A	G	0.411	1.00	-0.134	0.04	0.0007328	0.0033548		0.444	0.96	0.028214	0.019	0.1372405
rs2106510	7	95024163	A	G	0.411	1.00	-0.133	0.04	0.0007407	0.0033858		0.444	0.96	0.028196	0.01899	0.1373135
rs2375006	7	95024323	A	G	0.411	1.00	-0.133	0.04	0.0007434	0.003396		0.444	0.98	0.027792	0.01877	0.1384529
rs7810538	7	95024662	G	T	0.411	1.00	-0.133	0.04	0.0007431	0.0033951		0.444	0.98	0.027749	0.01874	0.1383541
rs7778478	7	95024886	C	T	0.411	1.00	-0.133	0.04	0.0007428	0.0033942		0.444	0.99	0.027629	0.01868	0.1387813
rs10281863	7	95025514	C	G	0.589	1.00	0.133	0.04	0.0007426	0.0033934		0.556	0.99	-0.02762	0.01867	0.1386435
rs10226503	7	95025951	C	T	0.589	1.00	0.133	0.04	0.0007425	0.0033929		0.556	1.00	-0.02739	0.01854	0.1393783
rs916711	7	95028184	G	T	0.589	1.00	0.133	0.04	0.0007401	0.0033939		0.556	1.00	-0.02719	0.01854	0.1422555
rs916712	7	95028457	A	G	0.405	0.98	-0.133	0.04	0.000836	0.0036884		0.438	0.98	0.02864	0.0188	0.1274379
rs916714	7	95028595	C	T	0.590	1.00	0.134	0.04	0.0007141	0.0033324		0.557	0.99	-0.02694	0.01859	0.1469576
rs10251365	7	103207855	A	G	0.331	1.00	-0.135	0.041	0.001084	0.0009339		0.338	1.00	0.009665	0.01924	0.6150929
rs10280683	7	103207991	A	G	0.669	1.00	0.135	0.041	0.001084	0.0009369		0.663	1.00	-0.00974	0.01924	0.6122067
rs2237637	7	103211834	C	T	0.353	1.00	-0.132	0.041	0.00127	0.0009773		0.357	1.00	0.011441	0.01896	0.5459835
rs669641	7	105133474	C	T	0.683	0.99	-0.133	0.042	0.001557	0.0006377		0.739	0.85	-0.03763	0.02212	0.0886637
rs505288	7	105149071	C	T	0.086	0.88	0.234	0.073	0.00144	0.0002162		0.097	0.89	0.058248	0.03342	0.0811749
rs11536571	7	111596308	C	T	0.107	0.81	0.243	0.07	0.0005213	0.0068831		0.093	0.73	0.006062	0.03432	0.8596749
rs13244206	7	118055796	C	T	0.025	0.43	0.659	0.191	0.0005553	0.0002791		0.020	0.30	-0.0359	0.10841	0.7403526
rs13226251	7	118112850	A	T	0.806	0.83	-0.178	0.053	0.000898	0.0006909		0.828	0.76	0.016598	0.02719	0.5412061
rs10487814	7	123656803	A	G	0.954	1.00	-0.316	0.094	0.0007562	0.0014307		0.960	0.99	0.021414	0.0487	0.6599033
rs17556632	7	123662340	A	G	0.047	1.00	0.31	0.094	0.0009308	0.0016941		0.040	0.99	-0.02067	0.04867	0.670859
rs1019196	7	123674265	C	G	0.954	1.00	-0.309	0.093	0.0009551	0.0016913		0.960	0.99	0.019001	0.04854	0.6952015
rs17556666	7	123679159	C	T	0.954	1.00	-0.308	0.093	0.0009587	0.0016931		0.960	0.99	0.016752	0.04827	0.7283133
rs17556861	7	123679947	A	G	0.047	1.00	0.308	0.093	0.0009618	0.0016957		0.040	0.99	-0.01629	0.0482	0.7351381
rs4427107	7	123681074	C	T	0.954	1.00	-0.308	0.093	0.0009633	0.0016974		0.960	0.99	0.015777	0.04812	0.7428011
rs12706574	7	123682255	A	G	0.047	1.00	0.308	0.093	0.0009647	0.0016999		0.040	0.99	-0.01535	0.04805	0.7491081
rs12706575	7	123682387	A	C	0.047	1.00	0.308	0.093	0.0009478	0.0016833		0.040	0.99	-0.01521	0.04802	0.7512652
rs17637743	7	123684470	A	T	0.047	1.00	0.309	0.093	0.0009144	0.0016499		0.040	0.99	-0.01522	0.04801	0.7509745
rs12706576	7	123684924	C	T	0.047	1.00	0.309	0.093	0.0009065	0.0016421		0.040	0.99	-0.01537	0.048	0.7485494
rs13224724	7	123686252	A	T	0.047	1.00	0.31	0.093	0.0008705	0.0016079		0.040	0.99	-0.01547	0.048	0.747059
rs12706577	7	123690380	A	G	0.047	1.00	0.31	0.093	0.0008706	0.0016221		0.040	0.99	-0.01645	0.04802	0.7318069
rs12706578	7	123690412	C	T	0.047	1.00	0.31	0.093	0.0008709	0.0016229		0.040	0.99	-0.01659	0.04803	0.7296122
rs12706579	7	123690504	C	T	0.953	1.00	-0.31	0.093	0.0008715	0.0016243		0.960	0.99	0.016812	0.04804	0.7261361
rs13231290	7	123693316	G	T	0.047	1.00	0.31	0.093	0.0008714	0.0016275		0.040	0.99	-0.01744	0.04806	0.7165185
rs12706581	7	123694360	C	T	0.953	1.00	-0.31	0.093	0.0008704	0.0016265		0.960	0.99	0.017728	0.04807	0.7120156
rs13228061	7	123695811	C	G	0.047	1.00	0.31	0.093	0.0008701	0.0016261		0.040	0.99	-0.01785	0.04807	0.7100831
rs12706584	7	123700369	A	G	0.953	1.00	-0.31	0.093	0.0008685	0.0016243		0.960	0.99	0.018692	0.04808	0.6972238
rs13231197	7	123702223	A	G	0.047	1.00	0.309	0.093	0.0008976	0.0016989		0.040	0.99	-0.01913	0.04809	0.6905653
rs13234240	7	123713356	C	T	0.953	1.00	-0.309	0.093	0.0008914	0.0016638		0.960	0.99	0.023296	0.04808	0.6277224
rs6943547	7	126731496	C	T	0.130	0.90	-0.218	0.061	0.000337	0.0063454		0.138	0.88	-0.00554	0.02765	0.8411833
rs17867823	7	126739530	A	G	0.877	0.98	0.2	0.06	0.0008688	0.0127168		0.868	0.97	0.008469	0.0269	0.7526896
rs17862359	7	126775526	C	T	0.876	1.00	0.197	0.059	0.0009239	0.0125488		0.867	1.00	0.009323	0.02647	0.7244498
rs10253460	7	126789577	C	T	0.124	1.00	-0.197	0.059	0.0008864	0.0119861		0.133	1.00	-0.0093	0.02646	0.7250482
rs1565630	7	128232502	C	T	0.197	1.00	-0.167	0.049	0.0006667	0.0270615		0.240	1.00	-0.00269	0.02136	0.8997354
rs2270590	7	128243152	A	G	0.173	1.00	-0.177	0.052	0.0006208	0.0281358		0.208	0.99	-0.00478	0.02243	0.8312724
rs1994765	7	128260217	A	G	0.795	1.00	0.18	0.048	0.0001765	0.004685		0.799	0.99	0.001193	0.02252	0.9577017
rs2291567	7	128270463	C	T	0.183	1.00	-0.217	0.05	0.0000134	0.0007419		0.169	0.99	0.015194	0.0238	0.5229416
rs11765908	7	130939202	A	G	0.178	1.00	-0.185	0.051	0.0003089	0.0004287		0.204	0.50	-0.03881	0.03278	0.2360533
rs1318460	7	132119714	A	C	0.333	0.73	-0.179	0.048	0.0001833	0.0001998		0.348	0.70	0.012563	0.02281	0.5814571
rs2241439	7	132131435	G	T	0.256	1.00	-0.137	0.044	0.001762	0.0009648		0.295	0.99	0.009719	0.02002	0.6270102
rs918886	7	132133948	C	G	0.256	1.00	-0.138	0.044	0.001735	0.0009544		0.295	1.00	0.011308	0.02001	0.5716882
rs889944	7	132142244	A	G	0.253	1.00	-0.15	0.044	0.0006908	0.000443		0.294	0.99	0.0124	0.02004	0.5358275
rs10435414	7	132145839	C	T	0.253	1.00	-0.149	0.044	0.0007056	0.0004526		0.294	0.99	0.01247	0.02005	0.5336413
rs1978508	7	132163285	C	T	0.214	0.90	-0.157	0.049	0.001481	0.0005282		0.247	0.85	0.012629	0.02298	0.5822371
rs4731903	7	132172271	C	G	0.253	1.00	-0.147	0.044	0.0008329	0.0005438		0.294	0.99	0.014855	0.0201	0.4594731
rs7803568	7	134021058	A	C	0.885	0.90	0.218	0.064	0.0006733	0.0013189		0.854	0.79	-0.00137	0.02918	0.9624038
rs7803365	7	134671939	A	G	0.319	1.00	-0.144	0.041	0.0004395	0.0142311		0.337	0.98	0.008466	0.01944	0.6629691
rs11767423	7	134672438	C	T	0.537	1.00	0.156	0.039	0.0000555	0.0004479		0.507	0.92	-0.02519	0.0192	0.1892624
rs11772685	7	134674454	A	G	0.447	0.91	-0.151	0.041	0.0002164	0.0014248		0.472	0.85	0.029134	0.02001	0.1450178
rs12673359	7	134682643	C	T	0.427	0.95	0.136	0.04	0.0006344	0.0005774		0.410	0.94	-0.02387	0.01905	0.2097733
rs11765559	7	134686918	A	G	0.600	1.00	-0.149	0.039	0.0001516	0.0001476		0.621	1.00	0.02173	0.01887	0.2492077
rs889933	7	134688928	A	G	0.598	1.00	-0.156	0.039	0.0000719	0.0000855		0.616	1.00	0.020399	0.01882	0.2780983
rs10447873	7	135998896	C	T	0.836	0.98	0.169	0.053	0.001335	0.0002188		0.832	0.97	0.004335	0.02613	0.8681415
rs9691292	7	137295495	G	T	0.906	0.96	0.172	0.068	0.0111	0.0009034		0.935	0.94	0.010493	0.03641	0.7729946

					GOYA QC		GOYA Overweight/control			GOYA BMI continuous	known	IARC QC		IARC results		
MARKER	chr	position	A1	A2	FREQ1	Rsqr	Beta	SE	p	p	gene name	freq1	Rsqr	in_beta	in_SE	in_p
rs13247635	7	143358198	A	G	0.317	0.87	0.154	0.045	0.0005476	0.0005211		0.254	0.83	-0.00879	0.02246	0.6954072
rs4118165	7	146871717	C	G	0.812	1.00	0.165	0.05	0.0008946	0.0040717		0.833	1.00	0.015954	0.02457	0.5158294
rs9640345	7	153577484	C	T	0.644	1.00	0.154	0.04	0.0001336	0.0000476		0.643	0.35	0.040635	0.03211	0.2053782
rs13245274	7	153587587	A	T	0.640	0.88	0.158	0.042	0.0001845	0.000165		0.627	0.51	0.04077	0.02634	0.121402
rs12703333	7	153597525	C	T	0.440	1.00	-0.131	0.039	0.0006972	0.0005621		0.432	0.76	-0.02681	0.02096	0.200471
rs10274585	7	153598752	C	G	0.560	1.00	0.13	0.039	0.0007615	0.0006139		0.568	0.76	0.026728	0.02095	0.2017708
rs12703334	7	153600740	A	T	0.439	1.00	-0.13	0.039	0.0007787	0.0006367		0.421	0.78	-0.02658	0.02076	0.2002157
rs12538214	7	154969302	A	G	0.237	0.98	-0.155	0.046	0.0007036	0.0014677		0.242	0.60	-0.00994	0.0275	0.7175648
rs10224330	7	154970100	C	T	0.745	0.92	0.16	0.046	0.0005371	0.0011307		0.740	0.66	0.003505	0.02553	0.8907232
rs7803510	7	154970977	C	G	0.256	0.92	-0.161	0.046	0.0005182	0.0011023		0.260	0.66	-0.00346	0.02551	0.8919024
rs288746	7	155299433	A	G	0.872	0.99	0.186	0.059	0.001463	0.0003487		0.891	0.99	-0.02632	0.02951	0.3719586
rs10277539	7	156220392	C	T	0.064	0.99	-0.26	0.08	0.001193	0.0005032		0.057	0.96	0.134517	0.03894	0.0005491
rs6972360	7	156234612	C	T	0.937	1.00	0.309	0.081	0.0001447	0.0000495		0.943	0.99	-0.13231	0.03863	0.0006126
rs10488060	7	156241406	C	T	0.104	1.00	-0.181	0.064	0.004759	0.0004938		0.097	0.99	0.083444	0.03049	0.0061692
rs10253338	7	156245332	A	T	0.895	1.00	0.177	0.064	0.005626	0.0005296		0.903	1.00	-0.08451	0.03043	0.0054532
rs6957768	7	156247591	C	T	0.063	1.00	-0.302	0.081	0.0001861	0.0000565		0.057	1.00	0.131595	0.0385	0.0006273
rs17837692	7	156250504	C	T	0.063	1.00	-0.296	0.081	0.0002517	0.0000815		0.057	1.00	0.131395	0.03849	0.0006386
rs17837694	7	156265737	A	G	0.938	1.00	0.303	0.081	0.0001994	0.0000736		0.944	1.00	-0.12927	0.03858	0.0008036
rs10243302	7	156269443	G	T	0.937	1.00	0.302	0.081	0.0001864	0.0000566		0.943	1.00	-0.12972	0.03853	0.000757
rs10250838	7	156270892	A	G	0.063	1.00	-0.302	0.081	0.0001864	0.0000566		0.057	1.00	0.129665	0.03854	0.0007632
rs10235980	7	156275671	A	C	0.937	1.00	0.302	0.081	0.0001882	0.0000608		0.943	1.00	-0.12939	0.03851	0.0007751
rs10226504	7	156293175	A	G	0.937	1.00	0.295	0.081	0.0002594	0.000095		0.943	1.00	-0.12878	0.03845	0.0008059
rs10244145	7	156294224	A	G	0.064	1.00	-0.301	0.081	0.0001922	0.0000661		0.057	1.00	0.128811	0.03845	0.0008029
rs9654686	7	156299393	A	G	0.066	1.00	-0.277	0.079	0.0004919	0.0001521		0.058	0.99	0.123596	0.03822	0.0012159
rs9654687	7	156299473	C	T	0.064	1.00	-0.301	0.081	0.0001922	0.0000662		0.057	1.00	0.128621	0.03844	0.0008149
rs2286993	7	156303941	C	T	0.064	1.00	-0.301	0.081	0.0001922	0.0000662		0.057	1.00	0.128524	0.03843	0.0008207
rs10265935	7	156309713	C	G	0.063	1.00	-0.296	0.081	0.000253	0.0000903		0.057	1.00	0.12899	0.03858	0.0008235
rs10253387	7	156319165	C	T	0.937	1.00	0.297	0.081	0.0002445	0.0000841		0.943	0.99	-0.1294	0.03881	0.000853
rs6946878	7	156320243	A	G	0.063	1.00	-0.303	0.081	0.0001801	0.0000577		0.057	0.99	0.129442	0.03886	0.0008618
rs2886451	7	156320667	A	T	0.063	1.00	-0.303	0.081	0.0001796	0.0000574		0.057	0.99	0.129501	0.03889	0.0008657
rs9886041	7	156321996	A	G	0.063	1.00	-0.303	0.081	0.0001783	0.0000564		0.056	0.99	0.129619	0.03897	0.0008769
rs10479647	7	156330867	A	C	0.933	1.00	0.303	0.079	0.0001169	0.0000211		0.942	0.99	-0.12168	0.03878	0.0016957
rs10479648	7	156331078	C	T	0.067	1.00	-0.297	0.079	0.000158	0.0000306		0.058	0.99	0.12172	0.0388	0.0016963
rs10275055	7	156405364	C	T	0.949	0.99	0.314	0.09	0.0005119	0.0002079		0.961	0.99	-0.09463	0.04532	0.036672
rs7777527	7	156456608	A	G	0.961	1.00	0.373	0.101	0.0002269	0.0005324		0.958	0.98	-0.09271	0.04627	0.0449552
rs12671438	7	156474366	A	G	0.038	0.94	-0.377	0.106	0.0003709	0.001038		0.038	0.87	0.078481	0.05123	0.1252798
rs7797488	7	158577616	A	C	0.734	0.88	0.12	0.046	0.009986	0.0008623		0.680	0.88	-0.02845	0.02099	0.1750647
rs6459925	7	158606970	A	G	0.258	0.95	-0.12	0.045	0.007732	0.0005482		0.305	0.91	0.01609	0.02073	0.4372146
rs6459927	7	158610009	A	T	0.258	0.95	-0.12	0.045	0.007792	0.0005584		0.304	0.90	0.016157	0.02075	0.4357186
rs3793239	7	158610760	G	T	0.265	0.92	-0.122	0.045	0.00716	0.0004718		0.314	0.84	0.015574	0.02129	0.4641115
rs3793241	7	158612203	C	T	0.256	0.93	-0.118	0.046	0.009632	0.0008423		0.303	0.86	0.017446	0.02116	0.4093977
rs896523	8	510044	A	G	0.114	1.00	0.188	0.061	0.002149	0.0004578		0.115	0.99	-0.03704	0.03039	0.2225985
rs17065149	8	512863	A	G	0.907	0.96	-0.227	0.068	0.0008971	0.000184		0.900	0.95	0.041415	0.03346	0.2154727
rs1669648	8	518709	C	T	0.102	0.95	0.213	0.066	0.001233	0.0001486		0.108	0.95	-0.04318	0.03198	0.1765936
rs2019513	8	1477462	A	C	0.933	0.97	-0.262	0.079	0.0008341	0.0051428		0.907	0.98	-0.00492	0.03105	0.8739196
rs2956896	8	1492697	A	G	0.905	0.93	-0.241	0.068	0.0004275	0.0042865		0.884	0.91	-0.0391	0.02887	0.175209
rs2404631	8	1500528	C	T	0.932	0.95	-0.26	0.078	0.0009	0.0053974		0.906	0.92	-0.01439	0.03145	0.6468419
rs2906589	8	1505408	C	T	0.930	0.95	-0.255	0.077	0.0009765	0.0074131		0.906	0.92	-0.01461	0.03145	0.6420354
rs7834457	8	1609162	C	G	0.216	0.89	0.165	0.05	0.0009434	0.0026574		0.291	0.89	0.003044	0.0214	0.8868055
rs2957085	8	1679412	C	G	0.825	0.82	-0.185	0.056	0.0009085	0.0009092		0.841	0.82	0.04606	0.02759	0.0947418
rs7005592	8	1687805	C	T	0.825	1.00	-0.162	0.051	0.001354	0.0006908		0.838	1.00	0.038597	0.02498	0.1219599
rs6558527	8	1690318	G	T	0.822	0.98	-0.168	0.051	0.0009177	0.000677		0.836	0.99	0.038967	0.02499	0.118635
rs4595147	8	1690976	G	T	0.320	0.98	0.13	0.042	0.002058	0.0009394		0.293	0.95	-0.02897	0.02025	0.1522552
rs7842425	8	1693448	A	C	0.703	0.97	-0.139	0.043	0.001199	0.0006681		0.735	0.95	0.026075	0.02085	0.2107583
rs7846610	8	1694082	C	T	0.297	0.97	0.139	0.043	0.001183	0.0006579		0.265	0.95	-0.02626	0.02089	0.2083262
rs4639549	8	1695451	A	G	0.297	0.97	0.14	0.043	0.001167	0.0006486		0.264	0.95	-0.02632	0.0209	0.2075906
rs4370560	8	1695601	A	G	0.703	0.97	-0.14	0.043	0.00116	0.0006447		0.736	0.95	0.026358	0.02091	0.2070406
rs7460287	8	1695681	C	G	0.703	0.97	-0.14	0.043	0.001152	0.0006401		0.736	0.95	0.026385	0.02091	0.2066844
rs6558533	8	1697325	A	G	0.178	0.97	0.174	0.051	0.000636	0.0005356		0.162	0.94	-0.03879	0.0255	0.1279765
rs6558534	8	1697379	A	G	0.178	0.97	0.174	0.051	0.000632	0.000533		0.162	0.94	-0.03869	0.02556	0.1298748
rs2293977	8	1698528	C	G	0.180	0.96	0.178	0.051	0.000485	0.0003939		0.163	0.93	-0.03575	0.02551	0.1607752
rs3812477	8	1721859	C	G	0.263	0.96	0.146	0.045	0.001065	0.0008682		0.226	0.92	-0.02864	0.02239	0.2004106
rs17685088	8	2091382	C	G	0.595	0.93	-0.142	0.041	0.0005515	0.00894		0.596	0.92	-0.02422	0.01928	0.2086009
rs12547890	8	2093140	G	T	0.585	0.96	-0.134	0.041	0.0009681	0.0131391		0.579	0.94	-0.04028	0.01913	0.0350775
rs7822374	8	3124112	C	G	0.982	0.56	-0.732	0.204	0.0003394	0.0005947		0.967	0.45	-0.09814	0.08007	0.2199971
rs7837956	8	4251947	A	G	0.773	0.95	0.153	0.047	0.001072	0.0007125		0.791	0.84	0.009732	0.02459	0.69201
rs7838113	8	4252084	A	G	0.773	0.95	0.153	0.047	0.001088	0.0007254		0.791	0.85	0.009773	0.02457	0.690569
rs6559005	8	5197388	A	G	0.240	1.00	0.154	0.046	0.0007952	0.0008462		0.274	0.95	0.002189	0.02086	0.9163195
rs17352626	8	5205658	C	G	0.375	0.97	0.141	0.041	0.0005365	0.0021124		0.361	0.95	0.013345	0.01925	0.4878752
rs7817922	8	5207677	A	C	0.379	1.00	0.138	0.04	0.0005465	0.0018656		0.359	0.96	0.013376	0.01917	0.4849707
rs10089694	8	5208501	A	C	0.377	1.00	0.136	0.04	0.0007088	0.002362		0.353	1.00	0.012662	0.01892	0.5030162
rs17352703	8	5209564	A	C	0.621	1.00	-0.138	0.04	0.0005622	0.0017843		0.644	0.98	-0.01366	0.01901	0.4721533
rs11136865	8	5211643	C	T	0.621	1.00	-0.139	0.04	0.0005224	0.0015724		0.644	0.95	-0.01566	0.01932	0.4172467
rs11780353	8	5212391	A	G	0.379	1.00	0.139	0.04	0.0005153	0.0015264		0.356	0.93	0.016626	0.01951	0.3936359
rs9644388	8	5212689	C	T	0.621	1.00	-0.139	0.04	0.0005131	0.0015127		0.645	0.93	-0.0168	0.01954	0.3895168
rs1420823	8	5212889	C	T	0.326	1.00	0.139	0.041	0.000709	0.0024215		0.304	0.92	0.001421	0.02042	0.9444749
rs1420822	8	5212920	A	G	0.379	0.99	0.14	0.04	0.0005051	0.0014871		0.355	0.93	0.016932	0.01956	0.386286
rs2693808	8	5387422	A	G	0.539	0.98	-0.12	0.039	0.002269	0.0002413		0.508	0.95	0.006618	0.01851	0.7204552
rs2693809	8	5387568	C	T	0.460	0.98	0.12	0.039	0.002248	0.0002396		0.492	0.95	-0.00662	0.01851	0.7204635
rs2527753	8	5388192	A	G	0.466	1.00	0.111	0.039	0.004089	0.0006133		0.498	0.97	-0.01114	0.01832	0.5427166
rs2693810	8	5388497	G	T	0.545	1.00	-0.122	0.039	0.001659	0.0001933		0.515	0.97	0.00532	0.0184	0.7723404
rs2929462	8	9103682	A	G	0.211	1.00	0.151	0.047	0.001356	0.000114		0.268	0.93	0.030005	0.02115	0.1556969

					GOYA QC		GOYA Overweight/control			GOYA BMI continuous	known	IARC QC		IARC results		
MARKER	chr	position	A1	A2	FREQ1	Rsqr	Beta	SE	p	p	gene name	freq1	Rsqr	in_beta	in_SE	in_p
rs2929290	8	9108383	G	T	0.770	0.99	-0.151	0.046	0.001009	0.0002355		0.725	0.98	-0.03137	0.02047	0.1250967
rs1383691	8	9177646	A	T	0.126	0.77	0.224	0.067	0.0007952	0.0004886		0.146	0.65	-0.004	0.03073	0.8963837
rs747751	8	9189927	A	G	0.866	0.72	-0.218	0.067	0.001193	0.0007858		0.845	0.59	0.008213	0.03138	0.7933541
rs814417	8	9973894	C	T	0.835	0.97	0.157	0.053	0.002964	0.0008682		0.789	0.97	-0.02717	0.02245	0.2257674
rs289620	8	13339006	C	T	0.469	0.99	0.112	0.039	0.004062	0.0008225		0.483	0.99	-0.00441	0.01835	0.809848
rs11785407	8	15213521	A	T	0.487	0.98	0.138	0.039	0.0003776	0.0080769		0.463	0.94	0.016112	0.01897	0.3952329
rs13255369	8	15216627	A	T	0.501	0.98	0.131	0.039	0.0007831	0.0148972		0.482	0.92	0.01731	0.01901	0.3620722
rs11784248	8	15232333	A	C	0.490	1.00	0.132	0.039	0.0006201	0.0118551		0.475	0.97	0.022654	0.0186	0.2228163
rs4421354	8	15233301	A	C	0.489	0.99	0.134	0.039	0.0005797	0.0121061		0.472	0.96	0.027814	0.01866	0.1357619
rs283	8	19859378	C	T	0.815	0.82	-0.187	0.055	0.0006679	0.0027974		0.830	0.79	0.030941	0.02644	0.241532
rs9650602	8	20486200	C	T	0.754	0.80	-0.161	0.05	0.001234	0.0009288		0.760	0.66	-0.04992	0.02569	0.0517878
rs12676503	8	21049990	C	T	0.859	0.87	-0.197	0.06	0.0009568	0.003255		0.868	0.87	-0.00504	0.02929	0.8632836
rs13257229	8	21072324	C	T	0.701	0.51	-0.215	0.059	0.000258	0.0004077		0.720	0.39	-0.02395	0.03159	0.4480607
rs2010283	8	21515161	C	T	0.120	0.99	-0.177	0.061	0.003441	0.0007318		0.097	0.88	-0.01072	0.03176	0.7354312
rs2010282	8	21515167	C	T	0.901	0.77	0.269	0.075	0.0003269	0.0001381		0.920	0.64	0.02274	0.04029	0.5721683
rs7813762	8	21758991	A	T	0.918	0.98	0.213	0.072	0.003041	0.0004463		0.924	0.96	-0.00095	0.03537	0.9784651
rs17522554	8	28707720	A	G	0.161	0.99	-0.207	0.053	0.0000945	0.0012677		0.160	0.94	-0.03907	0.02606	0.1335523
rs17451343	8	28762194	C	T	0.844	0.99	0.208	0.054	0.0001019	0.0008877		0.847	0.96	0.038172	0.02658	0.150708
rs11786316	8	28774749	A	C	0.131	0.99	-0.231	0.058	0.0000715	0.0003214		0.148	0.98	-0.03585	0.02706	0.1848729
rs10503833	8	28775203	G	T	0.131	0.99	-0.231	0.058	0.0000697	0.0003076		0.148	0.98	-0.03576	0.02705	0.1858843
rs17523818	8	28782961	A	G	0.870	0.99	0.232	0.058	0.0000701	0.0003179		0.852	0.98	0.036019	0.02707	0.1829693
rs12547794	8	28797503	G	T	0.203	1.00	-0.165	0.048	0.0006482	0.0009614		0.234	0.99	0.008094	0.02191	0.7115567
rs1512851	8	28826391	G	T	0.840	1.00	0.188	0.053	0.0003869	0.0038465		0.842	0.99	0.034918	0.02556	0.1715152
rs17524494	8	28835122	A	C	0.840	1.00	0.188	0.053	0.0003931	0.0038935		0.842	0.99	0.034911	0.02556	0.1715885
rs11784622	8	28838438	C	T	0.840	1.00	0.188	0.053	0.0003938	0.0038918		0.842	0.99	0.034906	0.02556	0.1716596
rs17524918	8	28861827	A	G	0.160	1.00	-0.188	0.053	0.000389	0.0037916		0.158	0.99	-0.0348	0.02556	0.1730791
rs11778263	8	28881341	C	G	0.840	1.00	0.188	0.053	0.0003838	0.003707		0.842	0.99	0.034718	0.02556	0.1740488
rs17525359	8	28885336	A	C	0.160	1.00	-0.189	0.053	0.0003669	0.0035051		0.158	0.99	-0.03458	0.02556	0.1757465
rs17454230	8	28982000	G	T	0.841	0.98	0.191	0.053	0.0003532	0.0037193		0.844	0.99	0.031854	0.02565	0.2139225
rs17526904	8	29003762	C	T	0.841	1.00	0.19	0.053	0.0003307	0.0039232		0.845	1.00	0.030601	0.02557	0.2310298
rs11780819	8	29055978	A	T	0.867	0.99	0.188	0.057	0.0009452	0.0157406		0.880	0.99	0.025214	0.0279	0.3657873
rs17455458	8	29075907	G	T	0.105	0.95	-0.252	0.065	0.0001144	0.0040321		0.110	0.96	-0.03624	0.03011	0.2282986
rs17455598	8	29077926	A	G	0.145	0.99	-0.204	0.055	0.0002087	0.0089146		0.125	0.99	-0.02805	0.02723	0.3025683
rs17456888	8	29139846	A	G	0.144	0.98	-0.2	0.056	0.0003172	0.0119507		0.123	0.96	-0.03079	0.02774	0.2667565
rs11775002	8	29175034	A	T	0.880	0.93	0.226	0.061	0.0002372	0.0033075		0.918	0.86	0.02168	0.03484	0.5334613
rs2294085	8	30556321	C	T	0.520	1.00	0.13	0.039	0.0008015	0.0015726		0.549	0.99	0.022627	0.01822	0.2138306
rs10503871	8	30556394	C	T	0.520	1.00	0.13	0.039	0.0007916	0.0015603		0.549	0.99	0.022613	0.01822	0.2141139
rs10503872	8	30556573	C	G	0.480	1.00	-0.131	0.039	0.0007721	0.0015358		0.451	0.99	-0.0226	0.01822	0.2144229
rs7833529	8	30574742	C	T	0.480	1.00	-0.132	0.039	0.0006614	0.0013962		0.451	1.00	-0.02229	0.01818	0.2199649
rs13278463	8	31016511	A	T	0.093	0.86	-0.259	0.072	0.000319	0.0019332		0.106	0.87	0.037307	0.03158	0.2371694
rs11574304	8	31093353	C	T	0.920	1.00	0.267	0.072	0.0002113	0.0015928		0.904	0.81	-0.059	0.03389	0.081469
rs1443080	8	33667649	C	T	0.586	0.87	0.126	0.042	0.002941	0.0003957		0.557	0.87	-0.01314	0.01952	0.5006388
rs1443079	8	33669122	G	T	0.410	0.96	-0.118	0.04	0.003288	0.0006245		0.423	0.90	0.005163	0.01944	0.7903644
rs1121332	8	33669433	C	T	0.410	0.96	-0.118	0.04	0.003288	0.0006251		0.423	0.90	0.005148	0.01943	0.7908562
rs4733441	8	33684403	A	C	0.589	0.99	0.117	0.04	0.003384	0.0006591		0.577	0.90	-0.00499	0.01939	0.7968521
rs11785633	8	33685862	A	C	0.588	1.00	0.117	0.04	0.003191	0.0006298		0.577	0.91	-0.00468	0.01928	0.8080822
rs12056870	8	33686298	A	G	0.588	1.00	0.117	0.04	0.00315	0.0006225		0.577	0.91	-0.00454	0.01926	0.813315
rs10954928	8	33693168	A	G	0.408	1.00	-0.111	0.04	0.005241	0.000763		0.376	0.99	-0.01199	0.01885	0.524311
rs4733167	8	33695814	A	G	0.408	1.00	-0.11	0.04	0.005728	0.0008021		0.374	0.98	-0.01032	0.01893	0.5853657
rs4733168	8	33698459	C	T	0.593	1.00	0.109	0.04	0.005904	0.0008164		0.626	0.98	0.010261	0.01894	0.587579
rs7845393	8	38478625	A	C	0.161	1.00	0.203	0.052	0.0001042	0.0000792		0.178	1.00	-0.00249	0.0238	0.9165824
rs565491	8	41637017	A	G	0.270	0.86	-0.147	0.048	0.00201	0.0008172		0.269	0.79	0.006786	0.02328	0.7704921
rs7824779	8	56093419	C	G	0.885	1.00	0.142	0.061	0.02086	0.0007837		0.881	0.88	0.03683	0.02859	0.1972809
rs7842680	8	56093552	A	G	0.885	1.00	0.142	0.061	0.02079	0.0007782		0.881	0.89	0.036888	0.02848	0.1948208
rs10113399	8	69025086	C	T	0.975	0.99	0.341	0.126	0.006643	0.0009501		0.971	0.92	-0.02324	0.05634	0.6796971
rs10113688	8	69025132	C	G	0.026	0.92	-0.347	0.128	0.006884	0.0009401		0.031	0.85	0.024955	0.05768	0.6649837
rs16919108	8	69791641	C	T	0.095	1.00	-0.222	0.067	0.0009541	0.0034619		0.089	0.90	0.070656	0.03318	0.0330824
rs723290	8	70647956	C	T	0.552	1.00	0.115	0.039	0.003239	0.0009221		0.566	1.00	0.004347	0.01843	0.8133781
rs6472466	8	70669327	A	T	0.209	0.98	0.137	0.047	0.003768	0.0008015		0.200	0.95	-0.0511	0.02338	0.0287243
rs4737998	8	70679494	G	T	0.570	1.00	0.159	0.039	0.0000441	0.0001698		0.524	1.00	-0.0137	0.01853	0.4592926
rs4738000	8	70681580	A	T	0.561	0.96	0.16	0.039	0.0000479	0.0002928		0.517	0.96	-0.01356	0.01886	0.4715832
rs349364	8	73594920	C	T	0.087	0.99	0.185	0.07	0.008028	0.0009941		0.085	1.00	-0.02245	0.03211	0.4840594
rs1038676	8	73644407	C	T	0.901	0.98	-0.185	0.066	0.005214	0.0004276		0.901	0.94	0.021729	0.03127	0.486793
rs349330	8	73647779	A	G	0.119	0.97	0.197	0.062	0.001407	0.0002241		0.118	0.94	-0.01671	0.0289	0.5628859
rs6989807	8	73649668	A	G	0.087	0.89	0.273	0.074	0.0002148	0.0011738		0.084	0.83	-0.02804	0.03445	0.4152265
rs4483131	8	75624895	C	G	0.973	0.94	0.473	0.127	0.0002047	0.0020536		0.975	0.91	-0.0497	0.05921	0.4009194
rs10504581	8	75703174	G	T	0.970	0.91	0.416	0.122	0.0006936	0.0053759		0.974	0.88	-0.05689	0.0578	0.32458
rs10504582	8	75704880	C	T	0.040	0.90	-0.376	0.107	0.0004436	0.0081932		0.032	0.88	0.050664	0.05177	0.3273605
rs3793404	8	75707335	C	T	0.040	0.90	-0.376	0.107	0.0004417	0.0081615		0.032	0.89	0.050377	0.05175	0.3299638
rs4735660	8	75711446	A	G	0.972	0.99	0.413	0.121	0.0006269	0.004067		0.977	0.97	-0.03721	0.0581	0.5216038
rs10504583	8	75718343	A	G	0.964	1.00	0.387	0.106	0.0002544	0.0043614		0.972	1.00	-0.03492	0.05175	0.4994617
rs4735663	8	75721014	A	G	0.963	0.99	0.382	0.105	0.0002852	0.0049242		0.972	1.00	-0.03523	0.05177	0.495832
rs12544130	8	75724848	A	G	0.029	0.98	-0.398	0.12	0.0009288	0.0058741		0.023	0.98	0.036066	0.05798	0.5335721
rs4735664	8	75725041	C	T	0.963	0.98	0.378	0.105	0.0003331	0.0055109		0.972	0.99	-0.03563	0.05181	0.4913301
rs16938932	8	75726157	A	T	0.038	0.96	-0.371	0.106	0.0004349	0.0066844		0.028	0.99	0.035846	0.05184	0.4888806
rs10957789	8	77206190	A	C	0.416	0.99	-0.135	0.04	0.0006858	0.0003458		0.452	0.97	-0.01552	0.01845	0.3997195
rs1459174	8	77207137	A	G	0.416	0.99	-0.135	0.04	0.0006865	0.0003453		0.452	0.97	-0.01552	0.01844	0.3996913
rs7011070	8	77207830	C	T	0.584	0.99	0.135	0.04	0.0006887	0.0003458		0.548	0.97	0.015529	0.01844	0.3992859
rs7844413	8	77210473	A	G	0.417	1.00	-0.135	0.04	0.0006979	0.0003458		0.453	0.98	-0.01576	0.01832	0.3892233
rs2054262	8	77214023	A	G	0.583	1.00	0.135	0.04	0.0006939	0.0003427		0.547	0.99	0.015972	0.01821	0.3800157
rs1903129	8	77218702	A	T	0.400	1.00	-0.132	0.04	0.0009574	0.0005722		0.427	0.99	-0.01048	0.01838	0.5683917
rs10102325	8	77222036	A	C	0.583	1.00	0.135	0.04	0.0006867	0.0003406		0.547	0.99	0.01598	0.0182	0.3796244

					GOYA QC		GOYA Overweight/control			GOYA BMI continuous	known	IARC QC		IARC results		
MARKER	chr	position	A1	A2	FREQ1	Rsqr	Beta	SE	p	p	gene name	freq1	Rsqr	in_beta	in_SE	in_p
rs10102558	8	77222228	C	T	0.400	1.00	-0.132	0.04	0.0009436	0.0005649		0.427	0.99	-0.01048	0.01838	0.5682514
rs7846206	8	77224182	C	T	0.583	1.00	0.135	0.04	0.0006646	0.00033		0.547	0.99	0.01598	0.0182	0.3795342
rs6985418	8	77225242	C	T	0.583	1.00	0.136	0.04	0.0006369	0.0003163		0.547	0.99	0.015978	0.0182	0.3795222
rs1380229	8	77227650	C	T	0.583	1.00	0.136	0.04	0.0006216	0.0003087		0.547	0.99	0.015983	0.0182	0.3793365
rs9298266	8	77231048	A	G	0.601	1.00	0.133	0.04	0.0008474	0.0005082		0.573	0.99	0.010487	0.01837	0.5677439
rs1380230	8	77231944	C	T	0.601	1.00	0.133	0.04	0.0008373	0.0005023		0.573	0.99	0.010484	0.01837	0.5678288
rs10957791	8	77233573	C	T	0.601	1.00	0.133	0.04	0.0008324	0.0004994		0.573	1.00	0.010487	0.01837	0.5677249
rs6472953	8	77233846	C	T	0.399	1.00	-0.133	0.04	0.0008275	0.0004965		0.427	1.00	-0.01049	0.01837	0.5676479
rs7006542	8	77235004	C	T	0.601	1.00	0.134	0.04	0.0008179	0.0004909		0.573	1.00	0.010491	0.01837	0.5675257
rs13281526	8	77237413	G	T	0.417	1.00	-0.137	0.04	0.0005717	0.0002841		0.453	1.00	-0.01601	0.01819	0.3783903
rs1431017	8	77237476	G	T	0.399	1.00	-0.134	0.04	0.000794	0.0004768		0.427	1.00	-0.0105	0.01836	0.5672693
rs1431018	8	77240307	A	G	0.399	1.00	-0.134	0.04	0.0007751	0.0004657		0.427	1.00	-0.0105	0.01836	0.5671213
rs6472954	8	77241954	C	G	0.399	1.00	-0.134	0.04	0.0007758	0.0004661		0.427	1.00	-0.0105	0.01836	0.567174
rs6982578	8	77242687	C	T	0.584	1.00	0.137	0.04	0.0005555	0.0002762		0.547	1.00	0.016022	0.01818	0.3777186
rs1367564	8	77243477	A	G	0.417	1.00	-0.137	0.04	0.000556	0.0002764		0.453	1.00	-0.01602	0.01818	0.3776416
rs10103327	8	77243877	C	T	0.601	1.00	0.134	0.04	0.0007782	0.0004675		0.573	1.00	0.010491	0.01835	0.5672592
rs1431019	8	77248112	A	G	0.399	1.00	-0.134	0.04	0.0007789	0.0004679		0.427	1.00	-0.01049	0.01835	0.567142
rs6994771	8	77248841	A	C	0.399	1.00	-0.134	0.04	0.0007952	0.0004772		0.427	1.00	-0.01049	0.01835	0.567158
rs7463018	8	77251366	G	T	0.584	1.00	0.137	0.04	0.0005777	0.0002868		0.547	1.00	0.016057	0.01817	0.3763383
rs9643701	8	77253031	C	T	0.601	1.00	0.134	0.04	0.0008075	0.0004843		0.573	1.00	0.010502	0.01834	0.5665933
rs10091125	8	77256827	A	G	0.601	1.00	0.134	0.04	0.0008081	0.0004846		0.573	1.00	0.0105	0.01834	0.5666648
rs7013630	8	77257149	A	G	0.399	1.00	-0.134	0.04	0.0008092	0.0004852		0.427	1.00	-0.0105	0.01834	0.5667972
rs10100261	8	77260467	C	T	0.584	1.00	0.136	0.04	0.0005815	0.0002887		0.548	1.00	0.016066	0.01816	0.3759047
rs2383968	8	77267133	A	C	0.523	0.98	0.125	0.04	0.001589	0.0009715		0.497	0.99	-0.00056	0.01816	0.975428
rs6989292	8	77270618	A	G	0.399	1.00	-0.134	0.04	0.0008123	0.000487		0.427	1.00	-0.0106	0.01833	0.5626964
rs7825295	8	77271605	A	G	0.399	1.00	-0.133	0.04	0.0008171	0.0004898		0.427	1.00	-0.01058	0.01833	0.5636791
rs7835161	8	77273664	A	G	0.399	1.00	-0.133	0.04	0.000822	0.0004925		0.427	1.00	-0.01052	0.01833	0.565675
rs9298268	8	77276166	A	C	0.601	1.00	0.133	0.04	0.0008369	0.0005011		0.573	1.00	0.010469	0.01833	0.5676775
rs9298269	8	77280045	C	T	0.417	1.00	-0.136	0.04	0.0006178	0.0003064		0.453	1.00	-0.01584	0.01816	0.3826191
rs6991713	8	77283035	A	G	0.399	1.00	-0.133	0.04	0.0008502	0.0005072		0.427	1.00	-0.01019	0.01834	0.5780376
rs13282738	8	77285089	A	G	0.399	1.00	-0.133	0.04	0.0008334	0.0004985		0.429	1.00	-0.00805	0.01839	0.6613896
rs13256169	8	77291501	C	T	0.398	1.00	-0.137	0.04	0.0005825	0.0003784		0.428	1.00	-0.00796	0.01838	0.6646685
rs1431015	8	77294135	C	T	0.602	1.00	0.137	0.04	0.0005988	0.0003887		0.572	1.00	0.007971	0.01837	0.6641772
rs1431014	8	77297273	C	T	0.602	1.00	0.137	0.04	0.0006102	0.000396		0.572	1.00	0.007973	0.01837	0.6640459
rs4735717	8	77301805	A	G	0.398	1.00	-0.137	0.04	0.0006104	0.0003961		0.428	1.00	-0.00798	0.01837	0.6638654
rs9298270	8	77305548	C	T	0.398	1.00	-0.137	0.04	0.0006104	0.0003961		0.428	1.00	-0.00798	0.01837	0.6636
rs919577	8	77308136	A	G	0.602	1.00	0.137	0.04	0.0006104	0.0003961		0.572	1.00	0.007983	0.01836	0.6634865
rs7845313	8	77349565	C	T	0.533	0.82	0.151	0.043	0.0004274	0.000827		0.503	0.72	0.008727	0.02137	0.6827416
rs6997500	8	93859671	C	T	0.832	0.88	-0.166	0.055	0.002616	0.0001766		0.794	0.86	-0.02721	0.02458	0.2678782
rs12545094	8	93931740	C	T	0.193	1.00	0.148	0.049	0.002667	0.000455		0.230	0.99	0.022003	0.02168	0.3097222
rs2651460	8	97706848	G	T	0.406	1.00	0.131	0.04	0.0009922	0.0016187		0.478	0.94	0.001728	0.01865	0.9261177
rs920576	8	98216715	C	T	0.220	1.00	0.119	0.047	0.01036	0.0007768		0.175	1.00	-0.02338	0.02365	0.3224626
rs2436051	8	98220143	A	G	0.746	1.00	-0.118	0.045	0.008211	0.0004587		0.760	0.99	0.029715	0.02118	0.1602421
rs1693586	8	101738096	A	G	0.500	1.00	0.104	0.039	0.006864	0.0009898		0.474	0.99	-0.00917	0.01843	0.6185364
rs618673	8	108986046	G	T	0.927	1.00	0.259	0.075	0.000588	0.0024148		0.950	0.98	0.014618	0.04489	0.7444812
rs2453423	8	109059393	C	T	0.974	0.53	0.672	0.172	0.0000952	0.0001941		0.978	0.52	0.059785	0.09622	0.5340545
rs2514837	8	109111417	A	T	0.348	1.00	-0.105	0.041	0.009341	0.0008158		0.363	1.00	0.053211	0.01881	0.0046525
rs2440386	8	109112644	C	G	0.348	1.00	-0.105	0.041	0.009339	0.0008163		0.363	1.00	0.053221	0.01881	0.0046415
rs2443782	8	109112957	C	T	0.652	1.00	0.105	0.041	0.009336	0.0008168		0.637	1.00	-0.05323	0.01881	0.0046336
rs1452020	8	109120516	A	C	0.314	0.99	-0.111	0.042	0.007704	0.0008114		0.323	0.98	0.048154	0.01961	0.0140147
rs6990312	8	110671493	G	T	0.881	0.98	0.206	0.061	0.0007363	0.0038606		0.893	0.98	0.0033	0.02834	0.9072255
rs6989019	8	110678019	A	C	0.128	0.98	-0.22	0.059	0.0002043	0.0033613		0.122	0.91	-0.00051	0.02793	0.9853358
rs6982592	8	110682176	A	G	0.117	1.00	-0.202	0.061	0.0009567	0.003819		0.107	1.00	-0.00408	0.02825	0.8849666
rs7833753	8	110686139	A	G	0.126	1.00	-0.217	0.059	0.0002386	0.0036662		0.115	0.99	0.00063	0.02759	0.9817743
rs11995060	8	110978485	A	C	0.201	1.00	-0.16	0.048	0.0008862	0.0339582		0.228	1.00	-0.00212	0.02227	0.9240314
rs7832016	8	110983650	G	T	0.201	1.00	-0.16	0.048	0.0008791	0.0338902		0.227	0.97	-0.00319	0.02255	0.8873136
rs1017876	8	110984574	C	T	0.799	1.00	0.16	0.048	0.0008648	0.0337395		0.773	0.97	0.003237	0.02255	0.8857882
rs12675695	8	110989391	A	G	0.201	1.00	-0.16	0.048	0.000861	0.0337263		0.227	0.97	-0.00327	0.02256	0.8846592
rs1605128	8	111044375	A	G	0.201	1.00	-0.164	0.049	0.000706	0.0243196		0.262	1.00	0.005734	0.02114	0.7859982
rs12155554	8	118391632	A	G	0.960	1.00	0.425	0.101	0.0000257	0.0001035		0.957	1.00	0.032927	0.04382	0.4520746
rs12156001	8	118391743	C	T	0.960	1.00	0.424	0.101	0.0000268	0.0001052		0.957	1.00	0.032875	0.04385	0.4530251
rs10505305	8	118398192	A	C	0.960	1.00	0.418	0.101	0.0000353	0.0001192		0.957	1.00	0.032366	0.04391	0.4606903
rs2124038	8	126671312	C	G	0.217	0.99	-0.168	0.047	0.0003681	0.0003625		0.255	0.99	0.041893	0.0213	0.049034
rs10110589	8	126672572	G	T	0.222	0.99	-0.164	0.047	0.0004716	0.0002887		0.256	0.99	0.04184	0.02131	0.0493932
rs1454617	8	126680485	A	C	0.778	1.00	0.151	0.047	0.001244	0.0006596		0.737	0.99	-0.03774	0.02109	0.0732669
rs1454618	8	126680536	A	G	0.222	1.00	-0.151	0.047	0.001252	0.0006643		0.263	0.99	0.037669	0.02109	0.0739117
rs10095552	8	127432587	A	G	0.451	1.00	-0.141	0.039	0.0002704	0.0001726		0.458	1.00	0.017456	0.01832	0.3403406
rs1554962	8	127449251	C	T	0.474	0.99	-0.125	0.039	0.001276	0.0004745		0.500	0.99	0.031532	0.01835	0.0854747
rs2142213	8	127451545	A	G	0.474	1.00	-0.123	0.039	0.001496	0.0005587		0.500	0.99	0.031372	0.01832	0.0866432
rs7006327	8	127453030	C	G	0.474	1.00	-0.123	0.039	0.001587	0.0005605		0.500	0.99	0.031257	0.01836	0.0884384
rs11783521	8	127455376	A	G	0.523	1.00	0.119	0.039	0.002146	0.0006193		0.500	0.99	-0.0312	0.01837	0.0891691
rs10112693	8	127459463	A	G	0.477	1.00	-0.119	0.039	0.002136	0.0006251		0.500	0.99	0.031039	0.01837	0.0908504
rs10956307	8	127460782	A	G	0.523	1.00	0.119	0.039	0.002135	0.0006983		0.501	1.00	-0.02968	0.01821	0.102858
rs10086471	8	127461308	C	T	0.523	1.00	0.119	0.039	0.00214	0.0007049		0.501	1.00	-0.02967	0.01821	0.102935
rs10956308	8	127462634	A	G	0.523	0.99	0.119	0.039	0.002155	0.0007143		0.500	0.95	-0.03037	0.01858	0.1018976
rs12056564	8	127463296	A	T	0.477	0.97	-0.12	0.039	0.002248	0.0007701		0.500	0.93	0.030856	0.01886	0.1015175
rs7014905	8	127463801	C	T	0.523	0.97	0.12	0.039	0.002257	0.0007743		0.499	0.91	-0.03122	0.01905	0.1010838
rs11986722	8	127597922	C	G	0.902	0.94	-0.238	0.068	0.0004247	0.0063374		0.876	0.88	-0.00306	0.02966	0.9178213
rs6989132	8	127608781	C	T	0.155	0.93	0.193	0.056	0.0005116	0.0063879		0.198	0.93	-0.01465	0.02391	0.5397179
rs11990954	8	127612296	A	C	0.146	1.00	0.187	0.055	0.0006739	0.0077699		0.189	0.99	-0.01467	0.02365	0.5346563
rs16901493	8	127614227	G	T	0.146	1.00	0.187	0.055	0.0006716	0.0077536		0.189	0.99	-0.01478	0.02366	0.5319626
rs7818201	8	127629535	A	G	0.074	0.88	0.292	0.079	0.0002375	0.0047789		0.101	0.76	-0.05287	0.03485	0.1290114

					GOYA QC		GOYA Overweight/control			GOYA BMI continuous	known	IARC QC		IARC results		
MARKER	chr	position	A1	A2	FREQ1	Rsqr	Beta	SE	p	p	gene name	freq1	Rsqr	in_beta	in_SE	in_p
rs6998716	8	127652749	C	T	0.935	0.92	-0.284	0.082	0.0005	0.0073762		0.912	0.80	0.065093	0.03631	0.072849
rs10090632	8	127654508	C	G	0.935	0.92	-0.284	0.082	0.0005242	0.0077444		0.912	0.79	0.064954	0.03635	0.0737532
rs16904363	8	131881504	C	T	0.170	0.93	-0.158	0.054	0.003193	0.0006921		0.211	0.90	0.023971	0.02321	0.3012099
rs7461567	8	132341363	A	C	0.653	1.00	-0.142	0.041	0.00047	0.0048497		0.635	1.00	-0.04383	0.01898	0.0208698
rs7837225	8	132353928	C	T	0.653	1.00	-0.141	0.041	0.0005296	0.005145		0.635	1.00	-0.04351	0.01899	0.0218639
rs4736771	8	132354250	A	G	0.347	1.00	0.141	0.041	0.0005367	0.0051796		0.365	1.00	0.043485	0.01899	0.0219403
rs4513159	8	132361772	C	T	0.347	1.00	0.138	0.041	0.0006672	0.0057893		0.365	1.00	0.042744	0.01898	0.0242647
rs4527077	8	132361899	A	C	0.347	1.00	0.138	0.041	0.000665	0.0057969		0.365	1.00	0.042726	0.01899	0.0243307
rs10102742	8	132373690	C	T	0.594	1.00	-0.134	0.039	0.0006965	0.0024067		0.574	1.00	-0.05659	0.01843	0.0021245
rs13254146	8	134294046	A	C	0.057	0.63	-0.302	0.106	0.004514	0.000972		0.043	0.50	-0.03674	0.06132	0.5487112
rs13265653	8	134867876	A	G	0.018	0.81	-0.479	0.167	0.004056	0.0003475		0.009	0.63	0.089303	0.09944	0.3687502
rs6471216	8	135408565	C	T	0.154	1.00	-0.191	0.054	0.000438	0.0012224		0.183	1.00	-0.00509	0.02372	0.8299388
rs4412399	8	135412601	C	T	0.179	0.99	-0.171	0.051	0.0008127	0.0022762		0.195	0.99	-0.01027	0.02323	0.6580312
rs4272415	8	135450865	C	T	0.832	1.00	0.174	0.052	0.0008537	0.0009696		0.830	1.00	0.004024	0.02393	0.8663293
rs6989835	8	135454682	C	T	0.832	1.00	0.174	0.052	0.00086	0.0009739		0.830	1.00	0.003297	0.02391	0.8902315
rs4529495	8	135455269	A	G	0.169	1.00	-0.174	0.052	0.0008609	0.0009746		0.170	1.00	-0.00322	0.02391	0.892669
rs7011517	8	135458552	G	T	0.831	1.00	0.173	0.052	0.0009379	0.0010619		0.826	0.97	0.000559	0.02407	0.9814619
rs10813812	9	324016	G	T	0.879	1.00	-0.203	0.059	0.0006126	0.0007426		0.914	0.78	0.091502	0.03747	0.0145554
rs17722518	9	325387	C	T	0.912	1.00	-0.223	0.068	0.001136	0.0002993		0.946	0.67	0.08475	0.04792	0.0767817
rs4237127	9	1119674	A	G	0.743	1.00	-0.152	0.044	0.0006428	0.0041554		0.743	1.00	0.000791	0.02092	0.9698169
rs4741117	9	1120580	A	G	0.744	1.00	-0.153	0.044	0.0005578	0.003446		0.743	1.00	-0.00173	0.02094	0.9341476
rs7871304	9	1144454	A	G	0.266	0.99	0.147	0.044	0.0007544	0.0030462		0.248	0.93	-0.00146	0.02201	0.9471902
rs2290465	9	2635201	C	G	0.778	0.66	0.195	0.058	0.000715	0.0121856		0.785	0.47	-0.03857	0.03176	0.2242492
rs6476553	9	3633012	A	G	0.942	0.92	0.272	0.088	0.001961	0.0001519		0.927	0.94	-0.01101	0.03768	0.7698822
rs6476554	9	3637685	A	G	0.944	1.00	0.271	0.086	0.001602	0.0001912		0.929	1.00	-0.006	0.03683	0.870432
rs1537523	9	3639097	A	C	0.056	1.00	-0.27	0.086	0.001664	0.0002032		0.071	1.00	0.006069	0.03682	0.8689805
rs1537524	9	3639190	C	T	0.944	1.00	0.269	0.086	0.001751	0.0002205		0.929	1.00	-0.0061	0.03682	0.8683336
rs1342854	9	5781151	A	G	0.317	1.00	0.147	0.042	0.0004899	0.0041809		0.331	0.99	-0.02496	0.01947	0.1993432
rs7859471	9	6013626	C	T	0.054	0.99	-0.297	0.088	0.0007484	0.0184417		0.065	0.98	0.022818	0.03748	0.5422851
rs1578325	9	6907652	C	G	0.666	0.97	0.138	0.042	0.0008841	0.0003609		0.639	0.91	0.044646	0.01982	0.0241716
rs12380111	9	6914118	C	T	0.671	0.98	0.143	0.041	0.0005628	0.0001989		0.652	0.95	0.044335	0.0196	0.0236383
rs2185463	9	6914416	A	G	0.329	0.98	-0.143	0.041	0.0005647	0.0001992		0.348	0.95	-0.04434	0.0196	0.0236275
rs2185464	9	6914843	C	T	0.326	1.00	-0.139	0.041	0.0007683	0.0002403		0.342	0.97	-0.04638	0.01953	0.0174546
rs7043262	9	6916738	A	G	0.675	1.00	0.139	0.041	0.0007863	0.0002442		0.660	0.98	0.046139	0.01945	0.0176419
rs7861518	9	6926862	C	T	0.325	1.00	-0.14	0.041	0.0007298	0.000223		0.340	0.98	-0.04599	0.01945	0.0179803
rs10975928	9	6940134	A	C	0.682	0.99	0.151	0.042	0.0002851	0.0001079		0.667	0.96	0.044768	0.01979	0.0236236
rs7019108	9	6943554	C	T	0.318	1.00	-0.154	0.042	0.0002035	0.0000836		0.345	0.94	-0.03838	0.01987	0.0531868
rs10758809	9	6948299	A	C	0.683	0.99	0.156	0.042	0.0001816	0.0000662		0.669	0.97	0.043413	0.01979	0.0281251
rs7042372	9	6949840	A	G	0.656	0.99	0.137	0.041	0.0007898	0.0003936		0.645	0.99	0.041768	0.01923	0.0297843
rs9657633	9	6951871	C	T	0.304	0.95	-0.175	0.043	0.0000459	0.0000183		0.329	0.98	-0.04138	0.01975	0.0360667
rs10815490	9	6952574	C	T	0.302	0.95	-0.175	0.043	0.0000439	0.0000184		0.327	0.96	-0.04184	0.01995	0.0358432
rs10815491	9	6952695	G	T	0.302	0.95	-0.175	0.043	0.0000439	0.0000185		0.326	0.96	-0.04186	0.01996	0.0358052
rs1890918	9	6955451	C	G	0.730	0.98	0.177	0.044	0.0000562	0.0000251		0.694	0.89	0.040034	0.02119	0.0586494
rs10815495	9	6956001	A	G	0.730	0.98	0.177	0.044	0.0000567	0.0000254		0.694	0.89	0.039954	0.0212	0.0593277
rs10815496	9	6956486	A	T	0.255	0.93	-0.174	0.046	0.0001493	0.0000812		0.278	0.83	-0.0476	0.02264	0.0353612
rs7865491	9	6957846	C	T	0.731	0.98	0.177	0.044	0.0000572	0.0000258		0.695	0.89	0.039923	0.02121	0.0595954
rs10758814	9	6961932	A	G	0.732	0.98	0.175	0.044	0.0000644	0.0000299		0.698	0.89	0.038547	0.02132	0.0703695
rs10758815	9	6962193	A	G	0.736	1.00	0.165	0.044	0.0001534	0.0000779		0.699	0.88	0.036816	0.0214	0.0851539
rs10815498	9	6962479	A	G	0.264	1.00	-0.166	0.044	0.0001529	0.0000778		0.301	0.88	-0.03663	0.02141	0.0868485
rs872389	9	7216117	A	G	0.751	1.00	0.132	0.045	0.003105	0.0007279		0.731	0.99	-0.0023	0.02037	0.9101046
rs872388	9	7216167	A	G	0.249	1.00	-0.133	0.045	0.002945	0.0006928		0.269	0.99	0.002332	0.02036	0.908714
rs10815558	9	7217918	C	T	0.753	1.00	0.132	0.045	0.003223	0.0008228		0.730	0.98	-0.0024	0.02049	0.9065946
rs7862604	9	7218110	C	T	0.247	1.00	-0.132	0.045	0.003225	0.0008279		0.270	0.98	0.002407	0.02049	0.9063936
rs16925615	9	7218282	A	G	0.248	1.00	-0.132	0.045	0.003267	0.0009023		0.270	0.98	0.002409	0.02049	0.906337
rs10120747	9	7218727	A	T	0.248	1.00	-0.132	0.045	0.003289	0.0009276		0.270	0.98	0.002418	0.02049	0.9059824
rs10117675	9	7219427	C	G	0.751	0.99	0.132	0.045	0.003248	0.0008232		0.730	0.97	-0.00238	0.02053	0.9075184
rs7022149	9	7221343	A	G	0.750	0.99	0.132	0.045	0.003275	0.0007025		0.730	0.93	-0.00183	0.02101	0.9307293
rs10976135	9	7221559	C	T	0.750	0.99	0.132	0.045	0.003327	0.0006639		0.729	0.92	-0.00161	0.02118	0.9393965
rs7026290	9	7221991	C	T	0.250	0.94	-0.133	0.046	0.003698	0.0007187		0.271	0.90	0.00129	0.02145	0.952011
rs7026398	9	7222230	A	G	0.751	0.90	0.135	0.047	0.004116	0.0007806		0.729	0.88	-0.00097	0.02168	0.9643116
rs12341185	9	8467980	A	G	0.664	1.00	-0.143	0.041	0.0004714	0.0124363		0.690	0.99	-0.02545	0.01981	0.1986068
rs2381804	9	8468068	C	T	0.664	1.00	-0.143	0.041	0.0004579	0.0122929		0.690	0.99	-0.02555	0.01981	0.196736
rs10977149	9	8468115	C	T	0.333	0.99	0.144	0.041	0.0004862	0.0121481		0.310	0.99	0.025574	0.01981	0.1962934
rs10758976	9	8488248	A	G	0.326	0.99	0.141	0.041	0.0006127	0.0140332		0.307	0.99	0.022048	0.0199	0.2674732
rs7035558	9	8490472	C	T	0.672	0.99	-0.148	0.041	0.0003341	0.0097287		0.693	0.98	-0.02216	0.01999	0.2672029
rs10758977	9	8494101	G	T	0.673	0.99	-0.148	0.041	0.0003203	0.0096354		0.693	0.98	-0.02225	0.02002	0.266137
rs1998516	9	8498148	A	G	0.279	1.00	0.146	0.043	0.0006403	0.0202142		0.287	0.99	0.007317	0.02041	0.719789
rs10977361	9	8912089	A	G	0.088	1.00	-0.196	0.068	0.004047	0.0009688		0.123	0.60	0.015079	0.03611	0.676025
rs10756542	9	14227649	A	C	0.468	0.95	-0.133	0.04	0.0007835	0.0038609		0.442	0.94	-0.00342	0.0188	0.8555914
rs10733289	9	14765859	A	G	0.627	1.00	-0.13	0.04	0.001248	0.0005819		0.621	0.99	-0.04048	0.01866	0.029931
rs10962186	9	15859511	G	T	0.891	0.84	0.175	0.067	0.009111	0.0008606		0.925	0.57	0.107257	0.04848	0.0268492
rs10810478	9	15911354	A	C	0.072	0.90	-0.217	0.079	0.00586	0.0009885		0.048	0.47	-0.13775	0.06745	0.040997
rs10962240	9	15951950	A	G	0.020	0.62	-0.473	0.179	0.008295	0.0005738		0.014	0.41	-0.20453	0.13599	0.1322716
rs10810515	9	16006603	A	G	0.036	0.99	-0.28	0.106	0.008216	0.0005467		0.033	0.91	-0.06997	0.05725	0.2212894
rs10810516	9	16010997	C	T	0.036	1.00	-0.279	0.106	0.008436	0.0005609		0.031	0.99	-0.06018	0.05654	0.286792
rs7040587	9	16246964	A	G	0.276	1.00	0.144	0.044	0.0009626	0.0042255		0.297	1.00	0.00459	0.02017	0.8198621
rs7043514	9	16247016	C	T	0.724	1.00	-0.144	0.044	0.0009731	0.004242		0.703	1.00	-0.00463	0.02018	0.8184227
rs12002727	9	21586183	C	G	0.090	0.58	0.332	0.09	0.0002342	0.0010127		0.106	0.51	0.018956	0.04036	0.6383383
rs10965079	9	21594969	A	G	0.644	0.60	0.187	0.052	0.0003299	0.0008856		0.642	0.49	0.016751	0.02689	0.5330099
rs7036435	9	25608261	C	T	0.682	0.99	-0.167	0.042	0.0000712	0.0016907		0.650	0.99	0.003648	0.01929	0.8498486
rs1336466	9	26323942	C	T	0.180	0.99	-0.173	0.051	0.0006282	0.0004522		0.215	0.94	0.010509	0.02273	0.6435859
rs1855980	9	26404780	C	T	0.176	0.78	-0.248	0.057	0.0000155	0.0000224		0.212	0.75	0.006765	0.02531	0.7890745

					GOYA QC		GOYA Overweight/control			GOYA BMI continuous	known	IARC QC		IARC results		
MARKER	chr	position	A1	A2	FREQ1	Rsqr	Beta	SE	p	p	gene name	freq1	Rsqr	in_beta	in_SE	in_p
rs1328425	9	26447170	C	T	0.367	0.97	-0.105	0.04	0.007956	0.0005907		0.367	0.95	-0.00157	0.01909	0.9343097
rs7867403	9	26447404	A	G	0.805	0.99	0.155	0.049	0.001588	0.000171		0.781	0.99	-0.01613	0.02199	0.463006
rs1028893	9	26450924	A	G	0.821	0.94	0.154	0.052	0.003032	0.000827		0.801	0.93	-0.01557	0.02335	0.5044687
rs868639	9	26453105	C	G	0.869	0.93	0.194	0.06	0.001213	0.0004891		0.832	0.92	-0.0004	0.02501	0.9870708
rs4576502	9	26453378	A	G	0.294	0.98	-0.118	0.043	0.005626	0.0005973		0.308	0.98	-0.00893	0.01982	0.6521758
rs3930533	9	26456575	A	C	0.317	1.00	-0.116	0.042	0.005456	0.0008094		0.331	1.00	-0.01569	0.01939	0.4180559
rs1407300	9	27220196	C	T	0.036	1.00	0.26	0.104	0.01212	0.0004592		0.053	1.00	-0.05921	0.04083	0.1466946
rs1590254	9	27224089	A	T	0.966	0.96	-0.266	0.109	0.0146	0.0004963		0.949	0.98	0.066962	0.04213	0.1116846
rs17756997	9	27229130	C	T	0.036	1.00	0.258	0.104	0.01286	0.0004899		0.053	1.00	-0.05958	0.04084	0.1442882
rs12554250	9	27239044	A	G	0.037	1.00	0.254	0.104	0.01418	0.0005411		0.053	0.99	-0.06013	0.04088	0.1410029
rs10511801	9	27239599	C	G	0.037	1.00	0.255	0.104	0.01365	0.0005203		0.053	0.99	-0.06014	0.04088	0.1409002
rs12555318	9	27247873	A	G	0.037	0.99	0.258	0.104	0.0127	0.0004743		0.053	0.99	-0.06024	0.04088	0.1402914
rs12555565	9	27248084	A	G	0.037	0.99	0.259	0.104	0.01248	0.0004633		0.053	0.99	-0.0603	0.04088	0.1399444
rs17695332	9	27249071	A	G	0.963	0.99	-0.26	0.104	0.01222	0.0004177		0.947	0.99	0.060022	0.04091	0.1420417
rs10120133	9	27740034	A	T	0.011	0.99	-0.672	0.198	0.0006892	0.0005881		0.012	0.59	0.128896	0.11928	0.2794732
rs10968093	9	27743227	A	G	0.011	0.99	-0.667	0.196	0.0006818	0.0005957		0.023	0.35	0.095331	0.09951	0.3376462
rs7035239	9	27743785	A	C	0.011	0.99	-0.678	0.198	0.0006116	0.0005369		0.012	0.57	0.121121	0.11901	0.3083896
rs10968269	9	27994402	C	T	0.012	0.97	-0.822	0.201	0.0000425	0.0000377		0.034	0.87	0.008292	0.0562	0.8826129
rs10968271	9	27995432	C	T	0.011	0.98	-0.822	0.2	0.0000411	0.0000363		0.035	0.91	0.015377	0.05457	0.7779323
rs12236967	9	28002746	A	C	0.011	1.00	-0.822	0.199	0.000037	0.0000305		0.035	0.92	0.017278	0.05387	0.7482021
rs12235674	9	28017694	A	G	0.014	0.97	-0.652	0.174	0.0001795	0.0002428		0.036	0.99	0.011287	0.05107	0.8249489
rs10968291	9	28021741	A	G	0.014	0.97	-0.649	0.174	0.0001895	0.0002599		0.036	0.99	0.010633	0.05116	0.8352314
rs10968296	9	28026229	C	T	0.986	0.98	0.61	0.172	0.0003807	0.0005899		0.965	1.00	-0.00379	0.05146	0.9412458
rs16912333	9	28027486	C	T	0.014	0.97	-0.612	0.172	0.000374	0.0005811		0.035	1.00	0.003446	0.05149	0.9465885
rs1409924	9	28768877	A	G	0.748	0.94	-0.154	0.046	0.0008318	0.0008699		0.726	0.94	0.00222	0.02175	0.9186183
rs1409925	9	28768923	A	T	0.265	0.97	0.149	0.045	0.0008132	0.0011013		0.282	0.93	-0.00191	0.02164	0.9297043
rs10968770	9	28770795	C	T	0.714	0.94	-0.148	0.044	0.0008156	0.0004416		0.698	0.94	0.000713	0.02099	0.9728672
rs10812875	9	28771231	C	T	0.744	1.00	-0.151	0.045	0.0007269	0.00086		0.722	1.00	0.003012	0.021	0.8858269
rs7024949	9	29188980	A	G	0.267	0.88	0.162	0.047	0.0005136	0.0005187		0.288	0.85	0.018922	0.02215	0.3926441
rs760402	9	35676376	A	G	0.701	0.98	-0.139	0.042	0.0009334	0.0027488		0.775	0.96	-0.00605	0.02168	0.7799783
rs3793537	9	35677556	C	G	0.284	0.99	0.142	0.043	0.0008113	0.0023848		0.211	0.96	0.010439	0.02206	0.635825
rs2295795	9	35702003	A	G	0.284	1.00	0.144	0.042	0.0007011	0.0020433		0.211	1.00	0.012472	0.0216	0.5634002
rs2295797	9	35712282	C	T	0.287	1.00	0.142	0.042	0.0007972	0.0015762		0.213	1.00	0.012287	0.02153	0.567897
rs1534847	9	35713712	C	T	0.286	0.99	0.144	0.042	0.0007044	0.0013749		0.213	1.00	0.012241	0.02155	0.5696384
rs4142495	9	35718494	A	G	0.304	0.98	0.144	0.042	0.0006285	0.001518		0.225	0.99	0.007618	0.02129	0.7201898
rs1936637	9	36891161	C	T	0.345	0.99	-0.131	0.041	0.001276	0.0001725		0.353	0.98	-0.02632	0.01928	0.1717536
rs7031673	9	36894769	A	G	0.642	1.00	0.131	0.04	0.001108	0.000208		0.635	0.97	0.022387	0.01921	0.2435288
rs10814736	9	38668071	C	G	0.149	0.62	0.238	0.07	0.0006467	0.0021546		0.170	0.49	-0.10294	0.0366	0.0048906
rs4382540	9	70373001	C	T	0.092	1.00	0.207	0.067	0.002081	0.0007648		0.090	1.00	0.070442	0.03189	0.0270987
rs9644996	9	70375735	A	T	0.092	1.00	0.206	0.067	0.002171	0.0008294		0.090	1.00	0.070351	0.0319	0.0273163
rs9644997	9	70375812	A	G	0.908	1.00	-0.206	0.067	0.002188	0.0008423		0.910	1.00	-0.0703	0.0319	0.0274389
rs11143012	9	73626635	C	G	0.931	0.53	-0.351	0.105	0.000875	0.0018086		0.931	0.37	-0.08366	0.05683	0.1407271
rs7862749	9	74833655	C	T	0.687	0.95	-0.11	0.043	0.01046	0.0008291		0.669	0.97	-0.01687	0.0196	0.3889334
rs7849838	9	74866275	G	T	0.693	1.00	-0.123	0.042	0.00321	0.0002458		0.671	1.00	-0.01744	0.01947	0.3699945
rs10869216	9	74869563	A	C	0.694	1.00	-0.123	0.042	0.003272	0.0002514		0.671	1.00	-0.01745	0.01946	0.3696211
rs1961831	9	74872369	C	T	0.694	1.00	-0.122	0.042	0.003456	0.0002658		0.671	1.00	-0.01746	0.01946	0.369261
rs2068212	9	74879002	C	T	0.307	1.00	0.12	0.042	0.004076	0.0003018		0.329	1.00	0.017317	0.01947	0.3733447
rs1342024	9	74885480	C	G	0.307	1.00	0.119	0.042	0.004236	0.0003098		0.330	1.00	0.017814	0.01946	0.3595361
rs7865396	9	74891695	C	T	0.308	1.00	0.121	0.042	0.003735	0.0002603		0.330	1.00	0.01808	0.01945	0.3521051
rs10781111	9	74893887	C	T	0.692	1.00	-0.122	0.042	0.003575	0.0002461		0.670	1.00	-0.01813	0.01944	0.3507569
rs1342022	9	74895327	A	G	0.692	1.00	-0.122	0.042	0.003514	0.0002407		0.670	1.00	-0.01816	0.01944	0.349858
rs1342023	9	74895540	A	G	0.308	1.00	0.122	0.042	0.003498	0.0002414		0.330	1.00	0.01814	0.01945	0.3505459
rs7048360	9	74895694	A	G	0.308	1.00	0.122	0.042	0.003487	0.0002418		0.330	1.00	0.018104	0.01945	0.35167
rs2153769	9	74895926	C	T	0.692	1.00	-0.122	0.042	0.003482	0.000242		0.670	1.00	-0.01808	0.01946	0.3522631
rs2310333	9	74897200	A	G	0.308	1.00	0.122	0.042	0.003401	0.0002475		0.330	1.00	0.018005	0.01946	0.3545375
rs12335799	9	82863942	C	G	0.386	1.00	-0.135	0.04	0.0007663	0.0111259		0.373	1.00	0.004288	0.0191	0.822172
rs7875211	9	86272271	A	G	0.514	1.00	-0.109	0.039	0.00488	0.0008406		0.519	1.00	-0.00828	0.0181	0.6470497
rs10868166	9	86280229	A	T	0.484	1.00	0.109	0.039	0.004838	0.0007771		0.469	0.98	0.005881	0.01832	0.7479992
rs17804667	9	88314703	A	G	0.952	0.98	0.311	0.092	0.0007402	0.0056955		0.906	0.92	-0.04544	0.03248	0.1615326
rs17052330	9	88742375	C	T	0.987	0.70	-0.782	0.212	0.0002281	0.0003885		0.991	0.54	-0.10568	0.123	0.3898644
rs11142070	9	89857247	C	T	0.939	1.00	-0.251	0.081	0.001997	0.0004868		0.944	0.74	-0.01376	0.04729	0.7708731
rs10760017	9	99727744	C	G	0.358	0.90	0.081	0.043	0.05633	0.0007405		0.340	0.86	0.027916	0.02058	0.1745881
rs7869762	9	99854394	A	C	0.195	0.94	-0.148	0.05	0.003028	0.0008649		0.170	0.96	0.019632	0.02467	0.4258601
rs6478539	9	99858541	C	T	0.806	0.95	0.148	0.05	0.003109	0.000882		0.830	0.96	-0.01941	0.02465	0.4306429
rs16917111	9	100360884	C	T	0.042	0.52	-0.429	0.135	0.001511	0.0000834		0.043	0.44	-0.03309	0.06284	0.5981514
rs10988705	9	100908392	A	G	0.036	0.95	-0.387	0.109	0.0004058	0.0001551		0.068	0.18	0.126026	0.07857	0.1084599
rs12342980	9	100979787	A	G	0.965	0.38	-0.497	0.174	0.004245	0.0005427		0.971	0.30	-0.02445	0.09833	0.8034405
rs12378614	9	105372252	A	T	0.043	0.61	0.387	0.124	0.001841	0.0004019		0.040	0.55	-0.16037	0.06592	0.014923
rs13302605	9	106869975	C	T	0.854	0.85	0.207	0.06	0.0005374	0.0022021		0.891	0.51	0.001924	0.03883	0.9604611
rs2138620	9	107880920	A	G	0.105	0.90	0.224	0.066	0.000727	0.0002694		0.093	0.90	0.014468	0.03319	0.6626304
rs2138630	9	107904397	A	C	0.106	0.93	0.221	0.065	0.0006581	0.0001724		0.092	0.92	0.016328	0.03282	0.6185569
rs7020549	9	107907253	A	G	0.895	0.93	-0.22	0.065	0.0006875	0.0001749		0.908	0.92	-0.01661	0.03275	0.6116833
rs7023595	9	107907424	C	T	0.105	0.93	0.22	0.065	0.0006872	0.0001748		0.092	0.92	0.016646	0.03275	0.6109527
rs6477492	9	107907541	A	G	0.895	0.93	-0.22	0.065	0.0006872	0.000175		0.908	0.92	-0.01668	0.03274	0.6101009
rs10759171	9	107909927	C	T	0.105	0.93	0.22	0.065	0.0006892	0.0001756		0.092	0.92	0.016738	0.03273	0.6087652
rs1516892	9	107913411	C	T	0.891	0.91	-0.217	0.065	0.0008159	0.0001739		0.907	0.92	-0.01687	0.0326	0.6045088
rs1878368	9	107913818	C	T	0.109	0.91	0.216	0.065	0.0008258	0.0001746		0.093	0.92	0.016851	0.0326	0.6048923
rs7018790	9	107916612	A	G	0.870	0.99	-0.163	0.058	0.005185	0.0005886		0.904	0.95	-0.01616	0.03147	0.6072969
rs1516893	9	107917466	A	C	0.132	1.00	0.16	0.058	0.005734	0.0006453		0.098	1.00	0.015236	0.03009	0.6123542
rs4742986	9	107918881	A	G	0.132	0.99	0.158	0.058	0.006435	0.0007403		0.100	0.99	0.014264	0.03009	0.6352248
rs10978426	9	107924741	A	G	0.042	0.74	0.371	0.115	0.001244	0.0009888		0.031	0.67	-0.01367	0.06546	0.83448
rs10816771	9	110917784	C	T	0.768	0.99	-0.156	0.046	0.0006284	0.0013514		0.763	0.98	-0.02211	0.02181	0.3102456

					GOYA QC		GOYA Overweight/control			GOYA BMI continuous	known	IARC QC		IARC results		
MARKER	chr	position	A1	A2	FREQ1	Rsqr	Beta	SE	p	p	gene name	freq1	Rsqr	in_beta	in_SE	in_p
rs2805888	9	110920557	C	T	0.757	1.00	-0.165	0.045	0.0002298	0.0004849		0.747	1.00	-0.02381	0.02125	0.262049
rs1533641	9	110951596	A	G	0.759	0.96	-0.159	0.046	0.0005102	0.0009521		0.747	0.99	-0.0219	0.02143	0.3064533
rs10759330	9	110953858	C	T	0.750	0.93	-0.159	0.046	0.0005165	0.0007525		0.735	0.94	-0.01782	0.02154	0.4077396
rs6477693	9	110958186	A	C	0.709	0.97	-0.164	0.043	0.0001446	0.0004062		0.692	0.99	-0.04112	0.02004	0.0400506
rs4978774	9	110965136	A	G	0.263	1.00	0.168	0.044	0.0001178	0.0005007		0.262	1.00	0.022911	0.02094	0.2735116
rs6477694	9	110972163	C	T	0.392	1.00	0.131	0.039	0.0008655	0.0061097		0.408	1.00	0.035902	0.01849	0.0519891
rs2798311	9	114439415	C	T	0.952	0.64	0.535	0.116	0.00000384	0.0000177		0.949	0.55	-0.06148	0.05327	0.2481055
rs786965	9	114488016	A	C	0.908	0.99	0.286	0.068	0.0000241	0.0000628		0.914	0.96	0.005928	0.03298	0.8572012
rs786966	9	114488162	G	T	0.092	0.99	-0.287	0.068	0.0000238	0.000063		0.086	0.95	-0.00573	0.03297	0.8618693
rs786969	9	114489377	C	G	0.923	0.85	0.292	0.08	0.0002462	0.0004274		0.933	0.76	0.032586	0.04184	0.4357303
rs1711746	9	114492477	A	G	0.905	1.00	0.284	0.067	0.0000207	0.0001016		0.914	0.95	0.005696	0.03297	0.8627139
rs1711745	9	114492730	A	C	0.095	1.00	-0.284	0.067	0.0000209	0.0001037		0.086	0.95	-0.00569	0.03297	0.862938
rs786977	9	114499529	A	G	0.905	1.00	0.283	0.067	0.0000211	0.0001072		0.914	0.95	0.005428	0.03295	0.8690273
rs786978	9	114500060	A	G	0.095	1.00	-0.283	0.067	0.0000211	0.0001078		0.086	0.95	-0.00542	0.03294	0.8692217
rs2798316	9	114502373	C	T	0.098	1.00	-0.223	0.065	0.0006164	0.0018023		0.094	0.93	-0.01184	0.03208	0.7117785
rs2185769	9	114502415	A	G	0.907	1.00	0.265	0.067	0.0000738	0.0003672		0.917	0.99	-0.00074	0.03282	0.9821134
rs2185768	9	114502466	C	T	0.902	1.00	0.223	0.065	0.0006172	0.0018042		0.906	0.94	0.011744	0.03207	0.7139577
rs2153837	9	114502822	A	T	0.094	1.00	-0.265	0.067	0.0000741	0.0003683		0.083	0.99	0.000811	0.03279	0.9802443
rs1711739	9	114504295	A	G	0.094	1.00	-0.265	0.067	0.0000745	0.0003699		0.084	1.00	0.00095	0.03275	0.9768438
rs1418410	9	114505103	A	G	0.907	1.00	0.265	0.067	0.0000749	0.0003716		0.917	1.00	-0.00096	0.03274	0.9764679
rs786961	9	114511167	C	T	0.094	1.00	-0.265	0.067	0.0000751	0.0003723		0.084	1.00	0.000971	0.03273	0.9763175
rs787278	9	114513352	A	C	0.094	1.00	-0.265	0.067	0.0000762	0.0003796		0.085	0.98	0.001705	0.03286	0.9585803
rs2645994	9	114523143	A	G	0.093	1.00	-0.265	0.067	0.0000755	0.0003794		0.086	0.97	0.002664	0.0327	0.9350224
rs9409123	9	114546986	A	G	0.095	0.99	-0.247	0.067	0.0002135	0.0009765		0.090	1.00	0.003534	0.0312	0.9097406
rs2796032	9	114559725	C	T	0.688	1.00	0.154	0.042	0.000227	0.0025348		0.685	1.00	0.012275	0.01978	0.5346309
rs1891403	9	114567004	C	T	0.095	1.00	-0.245	0.066	0.0002248	0.0010596		0.091	1.00	0.003613	0.03113	0.9075406
rs2796028	9	114568576	C	T	0.905	1.00	0.245	0.066	0.0002248	0.0010595		0.910	1.00	-0.0036	0.03113	0.9078615
rs813101	9	114572728	C	G	0.095	1.00	-0.245	0.066	0.0002247	0.0010611		0.091	1.00	0.00355	0.03113	0.9091176
rs787287	9	114574066	C	T	0.905	1.00	0.245	0.066	0.0002246	0.0010634		0.910	1.00	-0.00355	0.03113	0.9090357
rs1361720	9	114575400	A	G	0.905	1.00	0.245	0.066	0.0002246	0.0010645		0.910	1.00	-0.00355	0.03113	0.9090835
rs2645993	9	114576088	A	T	0.905	1.00	0.245	0.066	0.0002247	0.0010657		0.910	1.00	-0.00355	0.03113	0.9090645
rs787284	9	114577861	A	G	0.095	1.00	-0.245	0.066	0.0002247	0.0010668		0.091	1.00	0.003554	0.03113	0.9090267
rs787280	9	114578906	A	G	0.905	1.00	0.245	0.066	0.0002248	0.0010687		0.910	1.00	-0.00355	0.03113	0.9090083
rs787294	9	114580245	C	T	0.905	1.00	0.245	0.066	0.0002248	0.0010695		0.910	1.00	-0.00355	0.03113	0.909017
rs787295	9	114581427	C	G	0.095	1.00	-0.245	0.066	0.0002249	0.0010708		0.091	1.00	0.003552	0.03113	0.9090693
rs787297	9	114581954	A	C	0.905	1.00	0.245	0.066	0.0002251	0.0010723		0.910	1.00	-0.00355	0.03113	0.90912
rs787298	9	114582027	C	T	0.095	1.00	-0.245	0.066	0.0002251	0.001073		0.091	1.00	0.003551	0.03113	0.9091033
rs787299	9	114582190	C	T	0.905	1.00	0.245	0.066	0.0002252	0.0010746		0.910	1.00	-0.00355	0.03113	0.9090332
rs787301	9	114582558	A	C	0.095	1.00	-0.245	0.066	0.0002255	0.0010771		0.091	1.00	0.003552	0.03113	0.9090662
rs787302	9	114583660	A	G	0.905	1.00	0.245	0.066	0.0002257	0.0010788		0.910	1.00	-0.00355	0.03113	0.9090717
rs787271	9	114589284	C	T	0.095	1.00	-0.245	0.066	0.0002263	0.0010845		0.091	1.00	0.00355	0.03113	0.9091277
rs787272	9	114589368	C	T	0.918	1.00	0.255	0.071	0.0003225	0.0019368		0.927	0.86	-0.00247	0.03768	0.9476336
rs787274	9	114590184	A	C	0.082	1.00	-0.255	0.071	0.0003223	0.0019357		0.073	0.86	0.002486	0.03768	0.9473484
rs787275	9	114590344	C	G	0.082	1.00	-0.255	0.071	0.0003224	0.001936		0.073	0.86	0.002489	0.03768	0.9472871
rs700122	9	114591668	A	G	0.095	1.00	-0.245	0.066	0.0002264	0.0010846		0.091	1.00	0.003555	0.03113	0.908993
rs787276	9	114591712	C	T	0.905	1.00	0.245	0.066	0.0002264	0.0010846		0.910	1.00	-0.00355	0.03113	0.909018
rs10733594	9	114592451	G	T	0.905	1.00	0.245	0.066	0.0002264	0.0010847		0.910	1.00	-0.00356	0.03113	0.9089143
rs6477959	9	114594117	A	G	0.905	1.00	0.245	0.066	0.0002264	0.0010847		0.910	1.00	-0.00356	0.03113	0.9089296
rs4979153	9	114594805	A	G	0.905	1.00	0.245	0.066	0.0002264	0.0010847		0.910	1.00	-0.00355	0.03113	0.9090648
rs4979154	9	114594852	A	G	0.095	1.00	-0.245	0.066	0.0002265	0.0010849		0.091	1.00	0.003554	0.03113	0.9090273
rs10739370	9	114596762	A	G	0.095	1.00	-0.245	0.066	0.0002264	0.0010848		0.091	1.00	0.003553	0.03113	0.9090506
rs11794493	9	114597435	A	G	0.030	0.82	0.425	0.128	0.0008847	0.0005943		0.024	0.70	-0.02559	0.07475	0.7318552
rs7030795	9	114598783	A	G	0.905	1.00	0.245	0.066	0.0002283	0.0010903		0.910	1.00	-0.00372	0.03113	0.9049195
rs10124260	9	114599162	C	T	0.905	1.00	0.244	0.066	0.0002319	0.0011015		0.910	1.00	-0.00405	0.03113	0.8964846
rs1891402	9	114607005	C	T	0.905	1.00	0.243	0.066	0.0002479	0.0011494		0.909	1.00	-0.00437	0.03113	0.8882243
rs4979157	9	114611612	C	T	0.905	1.00	0.243	0.066	0.0002544	0.0011686		0.909	1.00	-0.0047	0.03112	0.8799399
rs1418409	9	114613528	C	T	0.905	1.00	0.242	0.066	0.0002588	0.0011814		0.910	1.00	-0.00492	0.03121	0.8746749
rs7868830	9	114616946	A	G	0.905	1.00	0.242	0.066	0.0002586	0.001181		0.910	1.00	-0.00486	0.03126	0.8763019
rs1539338	9	114626008	A	G	0.095	1.00	-0.243	0.066	0.0002583	0.0011797		0.089	0.99	0.004279	0.03167	0.8924369
rs7859175	9	114626981	A	G	0.905	1.00	0.243	0.066	0.0002583	0.0011797		0.913	0.98	-0.00291	0.03222	0.9279419
rs4246892	9	114628470	A	G	0.905	1.00	0.243	0.066	0.0002583	0.0011797		0.913	0.98	-0.0029	0.03222	0.9282902
rs10739373	9	114633060	A	G	0.905	1.00	0.243	0.066	0.0002556	0.0011717		0.913	0.98	-0.00289	0.03223	0.928474
rs885387	9	114636211	A	G	0.905	1.00	0.244	0.066	0.0002375	0.0011149		0.913	0.98	-0.00288	0.03224	0.9286717
rs4978507	9	114649197	A	G	0.906	0.99	0.246	0.067	0.0002446	0.0011067		0.913	0.97	-0.00292	0.03247	0.9283655
rs4979165	9	114649360	C	T	0.906	0.99	0.246	0.067	0.000245	0.0011075		0.913	0.97	-0.00293	0.0325	0.9281241
rs10759590	9	114650388	C	G	0.911	0.97	0.229	0.069	0.0009521	0.0019839		0.918	0.96	-0.00824	0.03352	0.8057024
rs6415814	9	114663600	A	T	0.724	0.99	0.16	0.044	0.0002455	0.0004825		0.719	0.98	0.017555	0.02039	0.3889628
rs2182046	9	114665520	C	T	0.279	0.99	-0.163	0.044	0.000187	0.0003571		0.286	0.99	-0.01833	0.02021	0.3640857
rs2025544	9	114672790	A	G	0.088	0.99	-0.23	0.069	0.0008399	0.0015182		0.080	1.00	0.015273	0.03302	0.6434023
rs10733599	9	114674055	A	T	0.088	0.99	-0.231	0.069	0.0008259	0.0014838		0.080	1.00	0.016193	0.03303	0.6236721
rs1324931	9	114674103	C	T	0.086	1.00	-0.231	0.069	0.0008249	0.0013945		0.080	1.00	0.017118	0.03303	0.6039771
rs7873289	9	114678207	A	G	0.914	1.00	0.231	0.069	0.0008248	0.0013944		0.921	1.00	-0.01727	0.03307	0.6012718
rs10759603	9	114679402	C	G	0.086	1.00	-0.231	0.069	0.0008248	0.0013944		0.079	1.00	0.01731	0.03308	0.6004531
rs10739378	9	114679452	C	T	0.086	1.00	-0.231	0.069	0.0008248	0.0013944		0.079	1.00	0.017488	0.03312	0.5972188
rs10759604	9	114679569	A	G	0.914	1.00	0.231	0.069	0.0008248	0.0013944		0.921	1.00	-0.01755	0.03314	0.5961289
rs4979176	9	114681002	A	G	0.914	1.00	0.231	0.069	0.0008248	0.0013944		0.921	0.99	-0.01809	0.03323	0.5857655
rs4978510	9	114683616	C	G	0.915	1.00	0.239	0.07	0.0006275	0.0011313		0.921	0.99	-0.01816	0.03324	0.5844585
rs4979178	9	114684156	C	G	0.911	1.00	0.227	0.069	0.0009403	0.001596		0.914	0.99	-0.02547	0.03245	0.4321408
rs10124939	9	114694504	C	T	0.929	1.00	0.252	0.076	0.0008995	0.0029183		0.940	1.00	-0.01665	0.03727	0.6548597
rs10124979	9	114694664	G	T	0.929	1.00	0.251	0.076	0.0009432	0.0030204		0.940	1.00	-0.01662	0.03727	0.6552638
rs10115711	9	114695084	A	G	0.071	1.00	-0.25	0.076	0.0009742	0.0030928		0.060	1.00	0.016519	0.03727	0.6573342
rs11793088	9	114700897	C	T	0.956	1.00	-0.309	0.094	0.001063	0.0004452		0.955	0.89	-0.02399	0.04674	0.6074255

					GOYA QC		GOYA Overweight/control			GOYA BMI continuous	known	IARC QC		IARC results		
MARKER	chr	position	A1	A2	FREQ1	Rsqr	Beta	SE	p	p	gene name	freq1	Rsqr	in_beta	in_SE	in_p
rs11788257	9	114702338	C	T	0.044	1.00	0.309	0.094	0.001064	0.0004437		0.045	0.89	0.023991	0.0467	0.6071044
rs11794662	9	114709279	G	T	0.956	0.99	-0.315	0.095	0.0008678	0.0003794		0.955	0.87	-0.0243	0.04738	0.6076808
rs10981543	9	114710809	C	T	0.041	0.97	0.35	0.1	0.0004727	0.0003688		0.035	0.77	0.046052	0.05953	0.4387769
rs11789893	9	114711130	C	T	0.278	0.91	0.157	0.045	0.0005503	0.0016182		0.243	0.75	0.013293	0.02459	0.5885
rs7854949	9	114713951	A	G	0.950	0.90	-0.315	0.094	0.0008236	0.0011598		0.954	0.59	-0.01526	0.05834	0.7935003
rs1952004	9	114714102	A	G	0.050	0.90	0.315	0.094	0.0008196	0.0011504		0.046	0.59	0.015088	0.05841	0.795984
rs1952005	9	114714134	C	G	0.950	0.90	-0.315	0.094	0.0008202	0.0011516		0.954	0.58	-0.01511	0.05874	0.7967697
rs7855402	9	114714247	C	G	0.950	0.91	-0.314	0.094	0.0008034	0.0011029		0.954	0.57	-0.01494	0.05913	0.8003666
rs11789944	9	114714413	A	G	0.039	0.99	0.363	0.102	0.0003536	0.0003115		0.031	0.65	0.035107	0.06812	0.6059722
rs10981545	9	114714907	A	G	0.039	0.99	0.362	0.102	0.000356	0.000314		0.031	0.63	0.034917	0.06935	0.6143248
rs1249730	9	116096672	A	G	0.653	1.00	-0.15	0.041	0.0002148	0.000262		0.697	0.99	-0.00276	0.01984	0.8893998
rs1249729	9	116098075	A	C	0.570	1.00	-0.121	0.039	0.001884	0.0005971		0.600	0.89	-0.00715	0.01971	0.7167284
rs1249728	9	116098435	A	T	0.567	0.96	-0.124	0.04	0.001717	0.0005214		0.604	0.90	-0.00818	0.01955	0.6754159
rs10982136	9	116098544	A	G	0.885	0.72	-0.254	0.071	0.000371	0.0003369		0.921	0.66	0.03429	0.04095	0.4020099
rs7031311	9	116101072	A	G	0.639	0.96	-0.142	0.041	0.0004889	0.0005477		0.672	0.92	-0.00072	0.02013	0.9713854
rs12349585	9	116853185	A	G	0.110	1.00	0.208	0.062	0.000835	0.0027479		0.101	1.00	0.025225	0.0298	0.3969362
rs7859478	9	116857226	G	T	0.110	1.00	0.208	0.062	0.0008362	0.0027504		0.101	1.00	0.025743	0.02979	0.3870842
rs1934486	9	119175134	C	T	0.972	0.99	-0.405	0.119	0.0006354	0.0022468		0.979	0.95	0.025785	0.05749	0.6535112
rs7875709	9	119185166	C	T	0.028	1.00	0.414	0.119	0.0005024	0.0016284		0.022	0.99	-0.03108	0.05695	0.5849054
rs10984339	9	120758935	C	T	0.077	0.95	0.262	0.075	0.000471	0.0037017		0.098	0.96	-0.05752	0.0337	0.087598
rs10984340	9	120759449	A	G	0.090	1.00	0.249	0.068	0.0002675	0.0008347		0.109	1.00	-0.03935	0.03081	0.20117
rs10121662	9	121642899	A	G	0.211	0.99	0.133	0.047	0.00462	0.0009832		0.182	0.99	-0.00668	0.02335	0.7744642
rs17482229	9	121648411	A	C	0.026	0.91	0.321	0.127	0.01163	0.0007984		0.011	0.27	-0.20584	0.17051	0.2269651
rs16909165	9	121653418	C	G	0.026	0.88	0.327	0.13	0.01173	0.0008028		0.011	0.24	-0.22256	0.1797	0.2151649
rs7849492	9	121658852	C	T	0.026	0.81	0.346	0.137	0.01141	0.0007651		0.011	0.22	-0.23464	0.18722	0.2097429
rs10760193	9	123645717	C	T	0.401	1.00	0.138	0.04	0.000476	0.0005974		0.379	0.96	-0.00539	0.01904	0.776945
rs10818604	9	123648430	A	T	0.599	1.00	-0.138	0.04	0.0004798	0.0006208		0.627	0.94	0.007137	0.01932	0.7116222
rs10760197	9	123650312	A	G	0.401	0.99	0.138	0.04	0.0004898	0.000648		0.373	0.94	-0.00724	0.01934	0.7078807
rs10760198	9	123650357	A	G	0.599	0.99	-0.138	0.04	0.0004946	0.0006608		0.627	0.94	0.0073	0.01935	0.7057502
rs2808411	9	125186018	C	G	0.088	0.76	-0.191	0.078	0.01474	0.0009852		0.081	0.81	-0.01939	0.03645	0.5943792
rs7048446	9	125372676	A	G	0.898	1.00	-0.193	0.064	0.002679	0.000696		0.888	1.00	-0.01423	0.02934	0.627262
rs7048972	9	125372925	C	T	0.898	1.00	-0.194	0.064	0.002507	0.0006452		0.889	1.00	-0.01412	0.02933	0.6299136
rs17287018	9	125406488	C	T	0.103	1.00	0.191	0.064	0.002874	0.0007764		0.110	0.99	0.009716	0.02953	0.7419341
rs7044210	9	125577489	A	C	0.835	0.92	-0.184	0.055	0.0007654	0.0017554		0.764	0.91	-0.00334	0.02292	0.8838705
rs1752164	9	125598358	C	T	0.153	1.00	0.187	0.055	0.000614	0.0015874		0.214	1.00	0.011215	0.02237	0.6157663
rs1752165	9	125598631	C	T	0.188	1.00	0.175	0.05	0.0004961	0.0006975		0.256	1.00	0.009961	0.02077	0.631314
rs1752166	9	125598664	G	T	0.188	1.00	0.175	0.05	0.0004967	0.0006982		0.256	1.00	0.009971	0.02077	0.6309076
rs10819188	9	128410776	A	G	0.288	0.99	-0.151	0.043	0.0004391	0.0129979		0.268	0.95	0.017521	0.02091	0.4017038
rs10819189	9	128419339	A	G	0.287	0.99	-0.15	0.043	0.0004533	0.0131932		0.268	0.95	0.017801	0.02087	0.3933757
rs12377679	9	128437576	A	G	0.289	1.00	-0.147	0.043	0.0005836	0.0148988		0.272	0.96	0.019046	0.02068	0.3567156
rs7859156	9	128439849	C	T	0.707	0.95	0.15	0.044	0.0006005	0.0180552		0.728	0.94	-0.01858	0.02085	0.3726442
rs11793373	9	128447364	A	G	0.283	0.93	-0.17	0.045	0.000131	0.0092722		0.262	0.91	0.008226	0.02154	0.7023313
rs10760446	9	128448266	C	G	0.719	0.92	0.169	0.045	0.0001793	0.0140512		0.741	0.89	-0.00643	0.02177	0.7675832
rs4322101	9	128468498	A	G	0.618	1.00	-0.149	0.04	0.0001697	0.0031675		0.581	1.00	-0.01814	0.01853	0.3271269
rs10121527	9	128471034	C	T	0.619	0.99	-0.151	0.04	0.0001589	0.0029865		0.584	0.98	-0.01835	0.01875	0.327443
rs4339739	9	128487952	G	T	0.392	0.96	0.151	0.04	0.0001791	0.0034673		0.422	0.96	0.020528	0.01887	0.2761844
rs10987411	9	128490260	C	T	0.397	0.95	0.15	0.041	0.0002182	0.0039864		0.428	0.93	0.022608	0.01909	0.2359466
rs2277158	9	128493050	A	G	0.607	0.96	-0.151	0.04	0.000189	0.0033806		0.579	0.95	-0.02379	0.01892	0.2082242
rs13295990	9	128495408	C	G	0.399	0.98	0.16	0.04	0.0000664	0.0009616		0.426	0.96	0.026816	0.01874	0.1520783
rs10987413	9	128499259	A	G	0.288	1.00	0.16	0.043	0.0001803	0.0067853		0.325	0.99	0.01457	0.0194	0.4521718
rs10987414	9	128499299	C	T	0.712	1.00	-0.16	0.043	0.0001818	0.0068169		0.675	0.99	-0.01471	0.01938	0.4473765
rs10760450	9	128499449	C	T	0.771	1.00	0.179	0.047	0.0001292	0.0027036		0.842	0.50	0.018258	0.03442	0.5954997
rs10733682	9	128500735	A	G	0.465	0.98	0.178	0.039	0.00000626	0.0001583		0.502	0.98	0.038294	0.01825	0.0357955
rs3861878	9	128501733	A	G	0.743	1.00	-0.148	0.044	0.0007348	0.0023584		0.645	0.87	-0.0106	0.02052	0.6052155
rs10987417	9	128502322	G	T	0.614	0.92	-0.188	0.041	0.00000514	0.0000507		0.533	0.83	-0.01637	0.01995	0.4115686
rs3906146	9	128503434	C	T	0.547	1.00	-0.156	0.039	0.0000589	0.0003609		0.478	1.00	-0.02502	0.01814	0.1675286
rs4358894	9	128504623	C	G	0.547	1.00	-0.157	0.039	0.0000541	0.0003274		0.478	1.00	-0.02494	0.01814	0.1689022
rs867560	9	128505054	C	G	0.547	1.00	-0.157	0.039	0.0000533	0.0003215		0.478	1.00	-0.02482	0.01815	0.1711616
rs13287548	9	128506277	C	T	0.575	0.95	-0.162	0.04	0.0000456	0.0001896		0.497	0.95	-0.02528	0.01861	0.1740313
rs13292976	9	128507161	C	T	0.548	1.00	-0.159	0.039	0.0000428	0.0002114		0.478	0.99	-0.02491	0.01818	0.1703785
rs12684797	9	128509360	C	G	0.370	0.95	0.17	0.041	0.0000322	0.0002809		0.440	0.93	0.003421	0.01911	0.8578237
rs7870976	9	128511718	A	C	0.370	0.95	0.17	0.041	0.0000326	0.0002808		0.440	0.93	0.00353	0.0191	0.8532431
rs2417033	9	128513397	A	G	0.319	1.00	0.148	0.041	0.0003243	0.0017024		0.402	0.99	0.005429	0.0188	0.7725856
rs10760452	9	128518729	C	T	0.307	0.74	0.173	0.049	0.0003807	0.0011548		0.356	0.74	-0.01415	0.02248	0.5288587
rs10987447	9	128548433	A	G	0.812	0.97	-0.165	0.05	0.000989	0.011642		0.779	0.92	-0.01104	0.02299	0.6308802
rs10987448	9	128548480	G	T	0.812	0.98	-0.166	0.05	0.0008739	0.0106591		0.780	0.98	-0.00933	0.02233	0.6760119
rs10819218	9	128550644	C	T	0.193	1.00	0.164	0.049	0.0007882	0.0118462		0.229	1.00	0.011571	0.02174	0.5942343
rs10819220	9	128551263	C	T	0.807	1.00	-0.165	0.049	0.0007713	0.0116143		0.771	1.00	-0.01159	0.02174	0.5935159
rs10114029	9	128552072	A	G	0.193	1.00	0.165	0.049	0.0007522	0.0113524		0.229	1.00	0.011618	0.02175	0.5929091
rs10819224	9	128553007	A	G	0.188	0.98	0.172	0.05	0.0005586	0.0111954		0.230	0.97	0.011254	0.02203	0.6091148
rs1005919	9	128553484	C	G	0.812	0.97	-0.173	0.05	0.0005491	0.0110427		0.770	0.95	-0.01073	0.02235	0.6309069
rs10124697	9	134579510	A	T	0.813	0.74	-0.21	0.058	0.0002823	0.0003081		0.827	0.60	0.038069	0.03073	0.2150634
rs2772000	9	134580819	A	C	0.813	0.72	-0.214	0.059	0.00025	0.000266		0.827	0.60	0.03839	0.03089	0.2135848
rs457298	9	134582856	C	G	0.813	0.70	-0.219	0.059	0.0002186	0.0002254		0.827	0.58	0.039414	0.03133	0.2080649
rs12550888	9	134595260	C	T	0.772	0.67	-0.184	0.056	0.0009912	0.0010869		0.784	0.57	0.032399	0.02855	0.2561198
rs2502740	9	135551188	A	G	0.393	0.83	-0.16	0.044	0.0002608	0.0002375		0.432	0.61	-0.0366	0.02332	0.116145
rs694821	9	135560561	C	T	0.458	0.99	0.165	0.039	0.000027	0.0003556		0.435	0.68	0.034184	0.02189	0.1180826
rs7040372	9	136920045	C	T	0.779	1.00	0.144	0.047	0.002035	0.0004409		0.720	0.77	0.031869	0.02304	0.1662087
rs7046516	9	136920096	A	G	0.779	1.00	0.144	0.047	0.00205	0.0004375		0.720	0.77	0.031864	0.02304	0.166337
rs6537958	9	136920644	C	T	0.779	1.00	0.144	0.047	0.002058	0.0004366		0.720	0.77	0.031903	0.02306	0.1661115
rs6537959	9	136920713	A	T	0.218	0.98	-0.149	0.047	0.00155	0.0003651		0.273	0.76	-0.02929	0.02332	0.2087434
rs6537962	9	136920756	C	T	0.221	1.00	-0.144	0.047	0.002048	0.000436		0.280	0.77	-0.03209	0.02312	0.1648427

					GOYA QC		GOYA Overweight/control			GOYA BMI continuous	known	IARC QC		IARC results		
MARKER	chr	position	A1	A2	FREQ1	Rsqr	Beta	SE	p	p	gene name	freq1	Rsqr	in_beta	in_SE	in_p
rs7863721	9	136921556	C	G	0.214	0.98	-0.149	0.048	0.001872	0.0002864		0.272	0.75	-0.03855	0.02372	0.1038886
rs472048	9	137111739	C	T	0.882	0.96	0.217	0.062	0.000419	0.0127521		0.858	0.96	-0.02241	0.02767	0.4175566
rs665748	9	137113249	A	C	0.118	0.96	-0.217	0.061	0.0004182	0.012725		0.142	0.96	0.022448	0.02766	0.4166496
rs500461	9	137121948	C	T	0.881	0.99	0.253	0.06	0.0000269	0.0014581		0.855	0.98	-0.02461	0.02717	0.3646249
rs542172	9	137127054	A	G	0.119	1.00	-0.254	0.06	0.0000252	0.0013845		0.144	1.00	0.02478	0.02697	0.3577486
rs12555792	9	137205913	A	G	0.271	1.00	0.15	0.044	0.0006215	0.0023427		0.284	0.74	0.003499	0.02346	0.8813153
rs10858362	9	137210299	A	C	0.391	0.89	0.131	0.042	0.001827	0.0008841		0.381	0.88	0.037405	0.02001	0.0614244
rs12253295	10	1662126	C	G	0.680	0.91	0.15	0.043	0.0005637	0.0007455		0.688	0.79	0.042258	0.02198	0.0543841
rs7078498	10	1662408	A	G	0.322	0.98	-0.143	0.042	0.0006024	0.0007426		0.294	0.89	-0.03435	0.02081	0.0986484
rs7070200	10	3320587	C	T	0.815	0.70	-0.172	0.059	0.003786	0.0000672		0.854	0.58	-0.02566	0.03382	0.4475957
rs2031574	10	3975194	A	G	0.223	0.82	0.171	0.052	0.0008962	0.0009948		0.178	0.78	0.032272	0.02678	0.2278089
rs11252655	10	4710626	A	G	0.390	0.99	-0.113	0.04	0.004623	0.0007431		0.372	0.72	0.010959	0.0219	0.6164126
rs7082806	10	5091653	A	C	0.210	0.90	-0.174	0.05	0.0005572	0.0033719		0.195	0.70	0.000805	0.02788	0.9769578
rs7099555	10	5091963	C	G	0.229	0.97	-0.159	0.047	0.0006579	0.0025276		0.210	0.74	0.000856	0.02628	0.9739901
rs608694	10	6608621	C	G	0.026	0.95	-0.339	0.126	0.006985	0.0007517		0.020	0.70	-0.08853	0.07334	0.2270384
rs1983910	10	6616499	A	G	0.026	0.95	-0.338	0.125	0.007057	0.0007631		0.020	0.71	-0.08799	0.07235	0.2235556
rs1887325	10	6624457	C	T	0.027	0.96	-0.346	0.125	0.005465	0.0006766		0.020	0.71	-0.08778	0.07234	0.2246339
rs7916556	10	6625420	A	G	0.974	0.96	0.346	0.125	0.005451	0.0006761		0.980	0.71	0.087724	0.07234	0.224884
rs11259486	10	6627678	C	T	0.027	0.96	-0.346	0.125	0.005426	0.0006751		0.020	0.72	-0.08707	0.07228	0.2279592
rs10752351	10	6628023	A	G	0.027	0.96	-0.346	0.125	0.0054	0.0006743		0.020	0.72	-0.08707	0.07227	0.2279288
rs943446	10	6636544	C	G	0.974	0.96	0.346	0.124	0.005389	0.0006756		0.980	0.72	0.087087	0.07227	0.2278201
rs2011261	10	6637374	C	T	0.027	0.96	-0.344	0.124	0.005531	0.000715		0.020	0.72	-0.08716	0.07227	0.2274352
rs1327245	10	6638564	A	G	0.973	0.97	0.341	0.123	0.005672	0.0007565		0.980	0.72	0.08724	0.07226	0.2269619
rs7916931	10	6640274	C	T	0.027	0.97	-0.341	0.123	0.005741	0.0007742		0.020	0.72	-0.08751	0.07225	0.2254846
rs10752356	10	6640839	C	T	0.027	0.99	-0.332	0.121	0.006272	0.0009399		0.020	0.72	-0.08759	0.07225	0.2250383
rs1998939	10	6641859	C	T	0.027	0.99	-0.332	0.121	0.006264	0.0009398		0.020	0.72	-0.08838	0.07221	0.2206283
rs1998940	10	6641954	G	T	0.973	0.99	0.332	0.122	0.006247	0.0009432		0.980	0.72	0.089622	0.07214	0.2137447
rs1327246	10	6642223	A	G	0.027	0.98	-0.337	0.123	0.006015	0.0009923		0.020	0.72	-0.0896	0.07214	0.2138736
rs1327247	10	6642273	C	G	0.973	0.98	0.337	0.123	0.006012	0.0009975		0.980	0.72	0.089634	0.07214	0.2136603
rs17389505	10	8603840	A	G	0.826	1.00	0.148	0.051	0.004153	0.0009932		0.849	1.00	-0.04987	0.02475	0.043748
rs2646437	10	8606317	A	G	0.443	1.00	0.117	0.039	0.002655	0.0005125		0.488	0.88	-0.00326	0.01942	0.8663681
rs1359567	10	8606807	C	T	0.652	0.99	-0.119	0.041	0.003418	0.0006414		0.615	0.87	0.012299	0.02007	0.5396563
rs7093770	10	8607135	C	T	0.348	0.99	0.119	0.041	0.003417	0.0006407		0.385	0.87	-0.01228	0.02007	0.5403178
rs12416202	10	8607895	C	T	0.348	0.99	0.119	0.041	0.003417	0.0006389		0.385	0.87	-0.01221	0.02007	0.5427667
rs7894791	10	8631375	A	C	0.409	1.00	0.134	0.039	0.0007049	0.0000676		0.399	0.90	0.003717	0.01965	0.8498923
rs11256627	10	10575960	A	G	0.718	0.91	0.157	0.045	0.0005169	0.0026076		0.668	0.83	0.01945	0.0216	0.3674258
rs4747860	10	10580170	C	T	0.329	1.00	-0.138	0.041	0.0008759	0.0037904		0.378	0.98	-0.03009	0.01921	0.1169492
rs2224693	10	10963857	A	T	0.513	0.99	-0.132	0.04	0.0008694	0.0010735		0.505	0.98	0.003673	0.01829	0.8407213
rs7070614	10	10965180	A	T	0.468	0.99	-0.134	0.04	0.0007004	0.0005903		0.455	0.98	-0.00446	0.01842	0.8085387
rs7087274	10	10966217	A	G	0.467	1.00	-0.133	0.039	0.0007577	0.0007093		0.455	0.98	-0.00436	0.01842	0.8128799
rs1018573	10	10968274	G	T	0.516	0.99	-0.132	0.039	0.0008557	0.0013312		0.516	0.95	0.006607	0.01859	0.7219752
rs7895452	10	10970862	A	T	0.467	1.00	-0.131	0.039	0.0008907	0.0008266		0.452	0.97	-0.00293	0.01844	0.8734765
rs1018574	10	10973579	C	T	0.788	0.82	-0.159	0.052	0.002411	0.0009042		0.768	0.74	0.004464	0.02507	0.8585327
rs7085558	10	10974763	A	G	0.469	1.00	-0.132	0.039	0.0008077	0.0006183		0.448	0.99	-9.2E-05	0.01828	0.99598
rs985640	10	10980362	A	G	0.467	1.00	-0.128	0.039	0.001148	0.0009486		0.445	1.00	0.00149	0.01825	0.9348783
rs7099743	10	13487201	A	G	0.028	0.97	0.43	0.12	0.0003531	0.0050038		0.020	0.92	0.094268	0.05925	0.1113285
rs17154771	10	14254916	C	G	0.081	0.99	0.238	0.072	0.0009109	0.0016791		0.081	0.90	0.040728	0.0348	0.2415097
rs11593981	10	16270378	G	T	0.695	0.97	-0.145	0.042	0.0006267	0.0002729		0.726	0.91	0.007182	0.02128	0.7354736
rs17318083	10	16436878	A	G	0.498	0.75	0.13	0.045	0.003626	0.0005739		0.502	0.73	-0.02363	0.02128	0.2664678
rs12357285	10	17108134	C	G	0.952	0.48	0.454	0.133	0.0006423	0.0011334		0.963	0.32	0.113097	0.08441	0.1799763
rs10795449	10	17203087	G	T	0.333	0.98	-0.144	0.042	0.0005877	0.0053898		0.400	0.89	-0.00981	0.02005	0.6241916
rs12781325	10	18341387	C	T	0.022	0.73	-0.488	0.16	0.002365	0.000991		0.021	0.69	-0.05174	0.07581	0.4945278
rs2497802	10	18392620	C	G	0.339	0.95	0.138	0.042	0.0009854	0.0002168		0.375	0.91	0.035785	0.0197	0.0690946
rs2185765	10	18395652	A	G	0.348	0.95	0.141	0.042	0.0007315	0.0002125		0.384	0.89	0.036665	0.01985	0.0645673
rs2497806	10	18398265	C	T	0.627	0.98	-0.147	0.041	0.0002744	0.0000906		0.592	0.97	-0.02753	0.01862	0.1389905
rs2488154	10	18400312	A	C	0.652	0.99	-0.14	0.041	0.0006131	0.0001723		0.620	0.99	-0.03536	0.01877	0.0594268
rs2488155	10	18401335	G	T	0.653	1.00	-0.137	0.041	0.0008144	0.0002224		0.620	1.00	-0.03521	0.01873	0.0599969
rs2250678	10	18408136	A	G	0.323	0.98	0.114	0.042	0.006382	0.0006304		0.364	0.96	0.036679	0.0193	0.0572208
rs2497820	10	18408855	A	G	0.667	0.98	-0.116	0.041	0.004974	0.0006529		0.626	0.93	-0.03801	0.01954	0.0516293
rs2497821	10	18409965	A	T	0.339	0.96	0.112	0.042	0.00731	0.0007339		0.385	0.95	0.034909	0.01927	0.0698569
rs550117	10	21346328	C	G	0.557	0.71	0.136	0.046	0.003291	0.0009099		0.565	0.42	0.020401	0.029	0.4813775
rs16924404	10	24560654	A	G	0.233	1.00	-0.138	0.046	0.002572	0.000645		0.227	0.93	0.014202	0.02297	0.5360127
rs6482376	10	24574045	A	G	0.231	1.00	-0.148	0.046	0.001366	0.0002253		0.224	0.95	0.018697	0.02281	0.4119958
rs7923307	10	24575448	A	G	0.764	1.00	0.144	0.046	0.00159	0.000234		0.767	0.90	-0.01791	0.02318	0.4393712
rs9971228	10	25233324	A	T	0.578	0.93	0.117	0.041	0.004096	0.0007426		0.603	0.82	0.013713	0.02023	0.4976025
rs11014291	10	25238693	C	T	0.346	0.99	-0.122	0.041	0.002562	0.000396		0.311	0.98	-0.01721	0.01969	0.3814614
rs10828722	10	25239957	A	G	0.655	0.99	0.122	0.041	0.002547	0.0003955		0.690	0.99	0.017052	0.01963	0.3847332
rs11014297	10	25242050	A	G	0.421	0.99	0.112	0.039	0.004312	0.0004809		0.453	0.99	-0.00098	0.01835	0.9574278
rs10828723	10	25245064	C	T	0.344	1.00	-0.122	0.04	0.002549	0.0004036		0.309	1.00	-0.01687	0.01957	0.3881904
rs7896608	10	25246418	G	T	0.344	1.00	-0.122	0.04	0.002553	0.0004061		0.309	1.00	-0.0168	0.01955	0.389764
rs7908585	10	25246560	C	T	0.656	1.00	0.122	0.04	0.002551	0.0004064		0.692	1.00	0.016791	0.01955	0.3899622
rs10828724	10	25247409	A	C	0.656	1.00	0.122	0.04	0.002553	0.0004066		0.692	1.00	0.016775	0.01955	0.3904288
rs12266014	10	25251297	C	T	0.635	0.96	0.118	0.04	0.003601	0.0006386		0.605	0.84	0.015768	0.02008	0.4319976
rs10828725	10	25258249	G	T	0.655	1.00	0.119	0.04	0.00325	0.0004465		0.691	1.00	0.016672	0.01955	0.3933802
rs1409307	10	25259844	A	G	0.580	1.00	-0.107	0.039	0.006265	0.0007342		0.548	1.00	0.000486	0.01831	0.9788077
rs11818463	10	25263373	C	G	0.345	1.00	-0.119	0.04	0.003246	0.0004521		0.309	0.99	-0.01671	0.01958	0.3929412
rs7086456	10	33642418	C	T	0.851	0.98	0.197	0.055	0.0003291	0.0014766		0.844	0.97	0.020994	0.0264	0.4261489
rs11009338	10	33646312	A	T	0.643	0.85	0.147	0.044	0.0008914	0.0026601		0.620	0.67	0.009326	0.02303	0.6852616
rs17366560	10	43176618	A	T	0.947	0.57	-0.39	0.115	0.0007326	0.0001713		0.952	0.48	0.031027	0.06198	0.6163423
rs17155988	10	44112060	A	G	0.018	0.89	-0.398	0.159	0.01231	0.000791		0.026	0.68	0.011309	0.06806	0.8679215
rs17176921	10	50250881	A	G	0.098	1.00	0.166	0.065	0.01079	0.0009162		0.075	1.00	0.031684	0.03573	0.3748335
rs7088781	10	50253121	C	T	0.902	1.00	-0.166	0.065	0.01074	0.0009063		0.926	1.00	-0.03107	0.03566	0.3831765

					GOYA QC		GOYA Overweight/control			GOYA BMI continuous	known	IARC QC		IARC results		
MARKER	chr	position	A1	A2	FREQ1	Rsqr	Beta	SE	p	p	gene name	freq1	Rsqr	in_beta	in_SE	in_p
rs17783607	10	50265347	A	G	0.902	0.95	-0.174	0.067	0.008817	0.0006166		0.928	0.96	-0.02873	0.03687	0.435478
rs6479821	10	63922889	A	G	0.050	0.87	-0.316	0.096	0.0009439	0.004111		0.060	0.84	0.005142	0.04236	0.9033049
rs16917302	10	63931204	A	C	0.935	0.83	0.306	0.087	0.0004533	0.0031481		0.918	0.74	-0.00072	0.03905	0.985292
rs11819137	10	63931662	C	T	0.946	0.89	0.308	0.092	0.0007801	0.0046414		0.933	0.81	0.001041	0.04122	0.9798404
rs10995995	10	66153590	G	T	0.217	0.82	-0.161	0.052	0.001968	0.0007488		0.219	0.76	0.026236	0.02558	0.3047575
rs12359386	10	66154996	A	G	0.197	1.00	-0.155	0.049	0.001566	0.0005004		0.188	1.00	0.02596	0.02363	0.2716572
rs10995999	10	66161236	G	T	0.800	1.00	0.159	0.049	0.001158	0.0002537		0.808	0.99	-0.02558	0.02343	0.2745523
rs12412844	10	66163781	C	T	0.201	1.00	-0.158	0.049	0.001157	0.0002395		0.192	0.99	0.025614	0.02342	0.2737338
rs12414101	10	66163789	A	G	0.799	1.00	0.158	0.049	0.001156	0.0002395		0.808	0.99	-0.02562	0.02342	0.2737118
rs10996007	10	66169041	A	G	0.200	1.00	-0.159	0.049	0.00116	0.0002441		0.192	0.99	0.025644	0.02347	0.2741808
rs10996013	10	66172578	G	T	0.200	0.99	-0.159	0.049	0.001164	0.000248		0.192	0.97	0.025722	0.02366	0.2765493
rs12184383	10	66179054	C	T	0.196	0.83	-0.173	0.054	0.001329	0.0003578		0.186	0.77	0.026537	0.02713	0.3275522
rs1879603	10	66179901	C	T	0.673	0.47	-0.214	0.06	0.0003651	0.0001261		0.657	0.45	-0.02413	0.0292	0.4081655
rs612371	10	84280542	C	T	0.256	0.95	-0.159	0.046	0.0004669	0.0007333		0.216	0.94	-0.00659	0.02289	0.7731912
rs1481126	10	84290423	C	T	0.263	0.99	-0.148	0.044	0.0008631	0.0012008		0.221	0.98	-0.00408	0.02222	0.8541183
rs12412061	10	84698419	A	T	0.540	0.96	-0.122	0.04	0.002135	0.0009759		0.538	0.95	0.012552	0.01874	0.5026423
rs10886391	10	85204704	A	G	0.287	0.93	-0.114	0.045	0.01119	0.0007149		0.291	0.92	0.014823	0.02127	0.4855201
rs12770083	10	87919777	C	T	0.950	1.00	-0.282	0.089	0.001604	0.0003556		0.939	0.92	0.02318	0.0404	0.5658275
rs10881613	10	91379205	G	T	0.261	0.97	0.151	0.044	0.0006824	0.0059597		0.292	0.98	0.016003	0.02053	0.4352826
rs927907	10	91379718	A	G	0.261	0.97	0.15	0.044	0.0006879	0.0060011		0.292	0.98	0.015864	0.02048	0.4382223
rs1359624	10	91385408	C	T	0.259	1.00	0.148	0.044	0.000751	0.0063938		0.292	0.99	0.015601	0.02037	0.4434744
rs3827866	10	98157414	C	T	0.320	1.00	-0.111	0.042	0.007454	0.0005131		0.344	0.96	0.01174	0.01976	0.552043
rs7896883	10	98777344	C	T	0.194	1.00	-0.167	0.049	0.0006439	0.0016022		0.220	0.98	-0.04945	0.02214	0.0254101
rs1105999	10	99350523	G	T	0.341	0.99	-0.14	0.041	0.0006019	0.0027197		0.368	0.72	0.011406	0.02228	0.6083736
rs12765071	10	102825908	A	G	0.853	1.00	-0.174	0.055	0.001488	0.0008871		0.847	0.98	-0.04446	0.0252	0.0774517
rs10786621	10	102870783	A	G	0.563	1.00	-0.085	0.039	0.02994	0.0007259		0.602	1.00	-0.02698	0.0185	0.1444219
rs12765602	10	103197170	A	T	0.277	0.88	-0.16	0.046	0.0005489	0.001929		0.293	0.85	-0.03504	0.02183	0.1082723
rs12769144	10	103359544	A	C	0.900	0.89	0.213	0.069	0.001916	0.0002245		0.891	0.87	0.051231	0.03316	0.1221199
rs11191732	10	105321751	A	G	0.218	1.00	0.158	0.047	0.0008064	0.0019782		0.169	0.94	-0.01778	0.02484	0.473703
rs11191749	10	105368830	A	G	0.737	0.99	-0.147	0.044	0.0008182	0.001151		0.796	0.96	-0.0002	0.02265	0.9927912
rs12359556	10	105380667	C	T	0.730	0.98	-0.145	0.044	0.0009413	0.0012078		0.776	0.94	0.005907	0.02222	0.7901686
rs17127166	10	111979315	A	G	0.072	0.99	-0.236	0.076	0.001987	0.0006982		0.107	0.90	0.017161	0.03132	0.5834064
rs17127170	10	111982212	C	T	0.063	0.84	-0.276	0.089	0.001849	0.0006306		0.089	0.68	0.018028	0.0394	0.6470277
rs12412423	10	111992160	C	G	0.929	1.00	0.236	0.076	0.002052	0.0009452		0.893	0.90	-0.01627	0.03129	0.6027488
rs716595	10	111996476	A	G	0.072	1.00	-0.236	0.076	0.00205	0.0009447		0.107	0.90	0.016289	0.0313	0.6025059
rs3793889	10	112017646	A	G	0.928	0.99	0.238	0.077	0.001867	0.000778		0.893	0.90	-0.01631	0.03134	0.6025052
rs4342958	10	113184663	G	T	0.978	0.97	0.525	0.139	0.0001546	0.0025474		0.971	0.91	-0.03879	0.05441	0.4756016
rs7069675	10	116016596	G	T	0.080	0.99	-0.242	0.072	0.0007953	0.0013203		0.070	0.96	0.021634	0.03523	0.5387943
rs10886654	10	122036498	A	C	0.220	0.99	0.17	0.047	0.0002844	0.0002026		0.194	0.92	0.005503	0.02444	0.821667
rs7917723	10	122036818	C	G	0.782	0.99	-0.172	0.047	0.0002538	0.00019		0.807	0.92	-0.00146	0.0246	0.9524956
rs10749367	10	122037345	C	T	0.190	0.98	0.19	0.049	0.0001091	0.0000748		0.175	0.98	0.005392	0.0248	0.8277006
rs10510067	10	122037465	C	T	0.188	0.98	0.181	0.049	0.0002366	0.0001419		0.171	0.99	0.007446	0.02495	0.7651846
rs10430700	10	122039915	A	G	0.190	0.98	0.189	0.049	0.0001211	0.0000855		0.174	0.99	0.005554	0.02473	0.8221287
rs2420694	10	122040129	C	T	0.190	0.98	0.189	0.049	0.0001237	0.0000878		0.174	0.99	0.005561	0.02471	0.8218222
rs11199264	10	122047147	A	G	0.812	1.00	-0.172	0.049	0.0004502	0.0003503		0.831	1.00	-0.00346	0.02502	0.8899447
rs11199266	10	122051602	A	G	0.629	1.00	-0.146	0.039	0.0002084	0.0001471		0.615	0.99	-0.01468	0.01903	0.440032
rs2901224	10	122052861	A	G	0.189	1.00	0.167	0.049	0.0006294	0.0004374		0.170	1.00	0.003435	0.02499	0.8905691
rs10886660	10	122053346	A	G	0.811	1.00	-0.167	0.049	0.0006358	0.0004413		0.831	1.00	-0.00343	0.02499	0.8907545
rs2420686	10	122054570	C	T	0.811	1.00	-0.167	0.049	0.0006392	0.0004435		0.831	1.00	-0.00342	0.02499	0.8912113
rs2103332	10	122055022	A	G	0.189	1.00	0.167	0.049	0.0006555	0.0004538		0.169	1.00	0.003405	0.025	0.8915597
rs10749368	10	122056471	A	G	0.586	1.00	-0.14	0.039	0.0002977	0.0004886		0.588	0.95	-0.00796	0.01904	0.675553
rs11199268	10	122056539	A	G	0.187	0.99	0.162	0.049	0.0009687	0.0005371		0.164	0.99	-0.00193	0.02566	0.9400114
rs2010946	10	122057169	A	T	0.586	1.00	-0.14	0.039	0.0003028	0.0004919		0.588	0.95	-0.00794	0.01904	0.6765144
rs11199269	10	122057667	C	T	0.812	1.00	-0.161	0.049	0.001043	0.0007426		0.828	0.99	-0.00067	0.02495	0.9786418
rs10886662	10	122059154	A	G	0.189	1.00	0.162	0.049	0.0009654	0.000672		0.172	0.99	0.00065	0.02495	0.9791999
rs3889706	10	126751524	C	T	0.901	0.55	-0.299	0.088	0.0006421	0.001111		0.895	0.37	0.004257	0.04908	0.9308255
rs580556	10	130946061	C	T	0.507	0.95	-0.102	0.04	0.01035	0.0009171		0.530	0.94	-0.01137	0.01898	0.5487246
rs1421041	10	130964856	A	G	0.513	1.00	-0.109	0.039	0.005045	0.000438		0.534	1.00	-0.01314	0.01854	0.4782015
rs2803452	10	130965508	C	T	0.513	0.97	-0.11	0.039	0.005223	0.0004636		0.534	0.99	-0.01318	0.01863	0.4788628
rs888373	10	130965570	A	G	0.477	0.91	0.116	0.041	0.004556	0.0004112		0.456	0.93	0.013981	0.01922	0.4666556
rs11146273	10	133923383	A	G	0.589	1.00	-0.13	0.039	0.0009261	0.0021355		0.636	0.92	0.020673	0.01967	0.2927699
rs11146313	10	133949994	C	T	0.605	0.98	-0.137	0.04	0.0005902	0.000449		0.657	0.99	0.014411	0.01917	0.4518192
rs7897895	10	133951664	A	G	0.559	0.89	-0.152	0.041	0.0002373	0.0011573		0.622	0.90	0.006976	0.01973	0.7233781
rs7911172	10	133952045	G	T	0.608	1.00	-0.138	0.04	0.0004843	0.0003513		0.657	0.99	0.014383	0.01916	0.4524804
rs10781566	10	133954851	A	G	0.608	1.00	-0.138	0.04	0.0004832	0.0003503		0.657	0.99	0.014358	0.01915	0.4530813
rs10870287	10	133955034	C	T	0.608	1.00	-0.138	0.04	0.0004913	0.0003513		0.658	0.99	0.01434	0.01917	0.454054
rs11818502	10	133957017	C	T	0.608	0.99	-0.138	0.04	0.000528	0.0003658		0.659	0.99	0.014198	0.01926	0.4607245
rs7074272	10	133958682	C	T	0.608	0.99	-0.138	0.04	0.0005403	0.0003735		0.659	0.97	0.014147	0.01939	0.465337
rs7944761	11	1361414	C	T	0.461	1.00	0.099	0.039	0.01139	0.0007392		0.431	0.99	0.021167	0.01871	0.2576686
rs10741669	11	2600056	A	G	0.901	0.61	0.295	0.084	0.0004604	0.000937		0.899	0.47	0.064862	0.04649	0.1626106
rs7118939	11	2603196	A	G	0.085	0.82	-0.259	0.078	0.0009174	0.002056		0.087	0.57	-0.05238	0.0452	0.246177
rs10832405	11	2605095	A	C	0.084	0.88	-0.255	0.076	0.0007619	0.0018335		0.084	0.62	-0.05651	0.0442	0.2006413
rs4271371	11	2607986	C	T	0.916	0.91	0.25	0.074	0.0007837	0.0019144		0.915	0.64	0.055425	0.04364	0.203733
rs231347	11	2631012	A	G	0.093	0.92	-0.239	0.071	0.0008083	0.001846		0.097	0.70	-0.04246	0.03919	0.2782515
rs231343	11	2635229	A	C	0.915	1.00	0.239	0.071	0.0007451	0.0017842		0.913	0.79	0.046264	0.03892	0.2342331
rs151216	11	2637391	C	T	0.916	1.00	0.247	0.071	0.0005477	0.0017124		0.895	0.67	0.044686	0.03866	0.2473499
rs12808061	11	5646551	A	C	0.104	0.99	0.223	0.064	0.000526	0.000153		0.089	0.67	0.006277	0.03853	0.8704796
rs1498553	11	5665604	C	T	0.467	0.99	0.128	0.039	0.001207	0.0009728		0.474	0.97	-0.00217	0.01849	0.906679
rs7928052	11	5715409	C	T	0.363	1.00	-0.142	0.041	0.0004848	0.0025572		0.380	1.00	-0.01709	0.01886	0.3643287
rs2133266	11	5726964	G	T	0.359	0.63	-0.175	0.051	0.0006138	0.0024851		0.399	0.65	-0.03054	0.02328	0.1892175
rs6486307	11	9362599	C	T	0.648	0.99	0.152	0.041	0.0001932	0.0002098		0.577	0.99	0.019521	0.01865	0.294767
rs11042329	11	9372834	C	T	0.193	0.99	-0.177	0.049	0.00031	0.0009137		0.273	0.99	-0.02149	0.02082	0.3017736

					GOYA QC		GOYA Overweight/control			GOYA BMI continuous	known	IARC QC		IARC results		
MARKER	chr	position	A1	A2	FREQ1	Rsqr	Beta	SE	p	p	gene name	freq1	Rsqr	in_beta	in_SE	in_p
rs12795344	11	9380117	C	T	0.361	1.00	-0.146	0.04	0.0002947	0.0003707		0.432	1.00	-0.02068	0.0185	0.2632032
rs7115477	11	9386195	C	T	0.361	1.00	-0.147	0.04	0.000278	0.0003469		0.432	1.00	-0.02052	0.0185	0.2669854
rs7480643	11	9388462	A	G	0.648	1.00	0.153	0.041	0.0001669	0.0001774		0.576	1.00	0.019073	0.01862	0.3051849
rs2290423	11	9406953	G	T	0.517	0.99	0.14	0.039	0.0002982	0.0003003		0.456	0.99	0.035073	0.01822	0.0540132
rs7122252	11	9408376	A	G	0.518	0.99	0.14	0.039	0.0002988	0.000293		0.456	0.99	0.034963	0.01821	0.0546976
rs8785	11	9425030	A	G	0.521	1.00	0.139	0.039	0.000337	0.0001631		0.458	1.00	0.034945	0.0182	0.0547275
rs1020621	11	9426439	G	T	0.478	0.99	-0.135	0.039	0.0005296	0.000246		0.542	0.99	-0.03523	0.01821	0.0528676
rs5010	11	10285820	C	T	0.030	0.61	0.488	0.147	0.0008921	0.0004208		0.040	0.59	-0.01337	0.06173	0.8283427
rs11021824	11	11352559	G	T	0.329	0.88	-0.154	0.044	0.0004891	0.0000783		0.286	0.82	-0.03486	0.02229	0.117462
rs7118622	11	11355982	C	T	0.261	1.00	0.142	0.044	0.001395	0.0008071		0.348	0.72	0.053769	0.02267	0.0176093
rs4910328	11	11357094	C	T	0.351	1.00	-0.147	0.041	0.0003126	0.0001277		0.306	0.99	-0.03308	0.01994	0.0968605
rs4453231	11	11359653	C	G	0.651	1.00	0.143	0.041	0.000475	0.000151		0.693	0.93	0.033335	0.02049	0.1034744
rs10765844	11	11360293	A	G	0.449	1.00	0.144	0.039	0.0002349	0.0000453		0.499	0.74	0.028692	0.02131	0.1778535
rs7104941	11	11977192	C	T	0.842	1.00	0.178	0.053	0.0007322	0.0023685		0.826	1.00	-0.02032	0.02406	0.3980151
rs903014	11	11977300	C	T	0.158	1.00	-0.178	0.053	0.0007793	0.0025686		0.173	1.00	0.020233	0.02411	0.4008717
rs2896594	11	11978236	A	T	0.842	1.00	0.177	0.053	0.0008259	0.0027622		0.827	0.99	-0.02021	0.02411	0.4016918
rs11023518	11	15286691	A	G	0.926	1.00	-0.267	0.075	0.0003581	0.0008362		0.925	1.00	0.02479	0.03367	0.4612325
rs9645645	11	15298200	C	G	0.917	0.92	-0.276	0.074	0.0001743	0.0004024		0.914	0.90	0.021888	0.03335	0.5112724
rs11023543	11	15332445	A	T	0.073	1.00	0.267	0.075	0.0003545	0.0008905		0.075	1.00	-0.01842	0.03372	0.5845622
rs12577240	11	15334185	C	G	0.073	1.00	0.267	0.075	0.0003552	0.0008935		0.075	1.00	-0.01862	0.03373	0.580668
rs11023568	11	15395857	C	T	0.074	0.99	0.265	0.075	0.0003846	0.0010573		0.075	0.98	-0.02073	0.03407	0.5424635
rs4581442	11	22173971	C	T	0.684	1.00	-0.144	0.042	0.0005109	0.0003264		0.735	1.00	0.034746	0.02054	0.0905133
rs10741929	11	22178360	A	C	0.317	1.00	0.144	0.042	0.000546	0.0003388		0.265	1.00	-0.03477	0.02056	0.0906811
rs1915070	11	24048326	C	G	0.442	0.72	0.126	0.046	0.006541	0.0006537		0.457	0.56	0.016558	0.02422	0.4937841
rs7101766	11	24055176	A	T	0.422	0.67	0.146	0.048	0.002254	0.0003082		0.435	0.56	0.015479	0.02434	0.5244313
rs1915063	11	24056349	A	G	0.394	0.72	0.156	0.047	0.0008884	0.0001302		0.413	0.59	0.011417	0.02387	0.6321459
rs4344486	11	24123708	A	G	0.152	0.99	0.146	0.054	0.006993	0.0009266		0.155	0.99	0.016111	0.02477	0.5150653
rs12418503	11	24125609	G	T	0.848	0.99	-0.146	0.054	0.00691	0.0009202		0.845	0.99	-0.01613	0.02475	0.5143537
rs12418800	11	24125671	G	T	0.848	0.99	-0.146	0.054	0.006879	0.0009179		0.845	0.99	-0.01613	0.02475	0.5141811
rs12418826	11	24125905	A	G	0.152	0.99	0.146	0.054	0.006753	0.0009087		0.155	0.99	0.01614	0.02472	0.5134888
rs12418831	11	24126100	A	C	0.152	0.99	0.146	0.054	0.006722	0.0009063		0.155	0.99	0.016143	0.02472	0.5133266
rs2957771	11	24126781	C	T	0.848	0.99	-0.146	0.054	0.006691	0.0009039		0.845	0.99	-0.01615	0.02471	0.5131867
rs12419276	11	24127266	C	T	0.848	0.99	-0.147	0.054	0.006648	0.0009012		0.845	0.99	-0.01615	0.02471	0.5130995
rs12420740	11	24127403	C	T	0.152	0.99	0.147	0.054	0.006587	0.0008967		0.155	0.99	0.016145	0.02471	0.5130827
rs2947750	11	24128116	C	T	0.153	1.00	0.148	0.054	0.005846	0.0008188		0.155	0.99	0.016156	0.0247	0.5126722
rs2947749	11	24128287	C	T	0.153	1.00	0.148	0.054	0.005822	0.0008169		0.155	0.99	0.016145	0.02469	0.5128971
rs2947745	11	24128608	C	T	0.154	1.00	0.148	0.054	0.0058	0.0008155		0.155	0.99	0.016152	0.02469	0.5126265
rs2957776	11	24131622	C	T	0.847	1.00	-0.148	0.054	0.005813	0.0008174		0.845	0.99	-0.01609	0.02473	0.5149369
rs2947760	11	24131757	C	G	0.153	1.00	0.148	0.054	0.005817	0.0008182		0.155	0.99	0.016086	0.02474	0.5151318
rs12418372	11	24131911	A	C	0.847	1.00	-0.148	0.054	0.005825	0.0008193		0.845	0.99	-0.01608	0.02474	0.5152171
rs12421188	11	24131999	C	T	0.847	1.00	-0.148	0.054	0.005835	0.000821		0.845	0.99	-0.01608	0.02475	0.5154697
rs11027985	11	24513273	A	G	0.175	0.97	0.149	0.052	0.004244	0.0009633		0.165	0.94	-0.01215	0.02508	0.6277049
rs7892	11	26974183	C	G	0.955	0.93	-0.3	0.098	0.002176	0.0005342	BDNF					
rs11029769	11	26975202	A	G	0.045	0.96	0.3	0.096	0.001807	0.0005905	BDNF					
rs7928331	11	26975700	A	T	0.045	0.97	0.299	0.096	0.001784	0.0006063	BDNF					
rs7928580	11	26975907	C	T	0.045	0.98	0.298	0.095	0.001772	0.0006382	BDNF					
rs7127273	11	27496660	C	T	0.947	0.99	-0.277	0.087	0.001509	0.0001558	BDNF					
rs7106909	11	29311927	C	T	0.816	1.00	0.175	0.051	0.000549	0.0024419		0.816	0.99	0.011256	0.02425	0.6423044
rs11822904	11	32615327	A	G	0.033	0.85	0.401	0.119	0.0007428	0.0019058		0.051	0.77	0.042815	0.04649	0.3566955
rs11034781	11	38467029	G	T	0.556	0.98	-0.135	0.039	0.0006513	0.0006714		0.558	0.91	0.003509	0.01924	0.8551726
rs12361508	11	38484099	C	T	0.399	0.95	0.135	0.041	0.0009601	0.0016701		0.383	0.83	0.004649	0.02043	0.8198071
rs3847638	11	38484748	A	G	0.399	0.95	0.135	0.041	0.0009448	0.0016573		0.384	0.83	0.004579	0.02042	0.8224805
rs3898491	11	38485204	C	G	0.601	0.95	-0.135	0.041	0.0009391	0.0016519		0.616	0.83	-0.00455	0.02042	0.8234783
rs7936967	11	38500945	C	T	0.596	0.98	-0.139	0.04	0.0005083	0.001257		0.613	0.83	-0.00333	0.02032	0.8697153
rs7115870	11	38504753	C	T	0.404	0.98	0.139	0.04	0.0005035	0.0012523		0.388	0.84	0.003095	0.02029	0.8786284
rs10837001	11	38516943	C	T	0.574	0.94	-0.156	0.041	0.000128	0.0004433		0.604	0.86	-0.00191	0.01992	0.9233983
rs1011138	11	38518525	G	T	0.440	1.00	0.153	0.039	0.0001	0.000376		0.401	0.88	0.000653	0.01958	0.9733775
rs10837002	11	38522303	C	G	0.611	1.00	-0.132	0.04	0.0008952	0.003275		0.636	0.91	-0.00071	0.01991	0.9716589
rs11606965	11	38529970	C	T	0.562	1.00	-0.147	0.039	0.0001685	0.0006882		0.600	0.96	0.001027	0.0188	0.9563968
rs11034857	11	38545887	C	T	0.438	0.99	0.147	0.039	0.0001753	0.0006914		0.400	0.96	-0.00057	0.01881	0.9758419
rs11034860	11	38550270	C	T	0.562	0.99	-0.147	0.039	0.000177	0.0006926		0.600	0.96	0.000622	0.01883	0.973614
rs17623660	11	38551921	G	T	0.563	0.99	-0.147	0.039	0.00018	0.0006953		0.600	0.96	0.000646	0.01884	0.9726085
rs2054580	11	38552500	C	T	0.438	0.99	0.147	0.039	0.0001804	0.0006964		0.400	0.95	-0.00066	0.01885	0.9719533
rs7117710	11	38553189	A	G	0.438	0.99	0.147	0.039	0.0001809	0.0006974		0.400	0.95	-0.00067	0.01885	0.9717093
rs17623761	11	38557800	A	G	0.438	0.99	0.147	0.039	0.0001815	0.0006981		0.400	0.95	-0.00066	0.01885	0.9720917
rs7127335	11	38559782	A	G	0.438	0.99	0.147	0.039	0.0001815	0.0006979		0.400	0.95	-0.00065	0.01886	0.9723393
rs1481294	11	38560651	A	G	0.437	0.98	0.147	0.04	0.0001901	0.0007076		0.400	0.95	-0.00066	0.01886	0.9722296
rs1481295	11	38560734	A	G	0.563	0.98	-0.148	0.04	0.0001902	0.0007075		0.600	0.95	0.000669	0.01886	0.9716631
rs11034867	11	38560955	A	T	0.437	0.98	0.147	0.04	0.000191	0.0007085		0.400	0.95	-0.00067	0.01886	0.9714925
rs10837017	11	38561056	C	T	0.437	0.98	0.147	0.04	0.0001915	0.0007092		0.400	0.95	-0.00068	0.01887	0.9710694
rs997192	11	38563376	A	G	0.437	0.98	0.147	0.04	0.0001922	0.0007095		0.400	0.95	-0.00071	0.01888	0.9698814
rs11034869	11	38564804	A	G	0.563	0.98	-0.147	0.04	0.0001927	0.0007108		0.600	0.95	0.000722	0.01888	0.9694927
rs12807104	11	38620013	C	G	0.436	0.98	-0.144	0.039	0.0002408	0.0005156		0.484	0.94	-0.00198	0.0187	0.9154149
rs10837029	11	38635789	C	T	0.565	0.99	0.149	0.039	0.0001391	0.0004208		0.511	0.92	0.005345	0.01893	0.7774635
rs3817089	11	38664229	A	G	0.549	1.00	0.152	0.039	0.0000891	0.0001461		0.509	0.92	0.006136	0.01891	0.7453628
rs12805836	11	38671243	C	T	0.565	0.99	0.157	0.039	0.0000618	0.000254		0.512	0.92	0.00454	0.01898	0.8107743
rs11034944	11	38720784	A	T	0.437	0.99	-0.161	0.039	0.0000391	0.0001976		0.487	0.92	-0.00444	0.01898	0.8148178
rs1867398	11	38742828	G	T	0.596	0.93	0.165	0.041	0.0000535	0.0004922		0.532	0.88	0.006843	0.01948	0.7250952
rs11034952	11	38745191	C	T	0.573	1.00	0.178	0.039	0.0000056	0.0000305		0.563	1.00	-0.00791	0.01837	0.6662651
rs11600794	11	38753799	C	T	0.405	1.00	-0.164	0.039	0.0000338	0.0000831		0.421	1.00	0.013954	0.01859	0.4524248
rs2655389	11	38760790	A	C	0.407	0.99	-0.163	0.04	0.0000397	0.0001082		0.426	0.99	0.00814	0.01867	0.6626504
rs1996032	11	38761839	A	G	0.598	1.00	0.165	0.04	0.0000288	0.0000569		0.580	1.00	-0.01047	0.01858	0.5726854

					GOYA QC		GOYA Overweight/control			GOYA BMI continuous	known	IARC QC		IARC results		
MARKER	chr	position	A1	A2	FREQ1	Rsqr	Beta	SE	p	p	gene name	freq1	Rsqr	in_beta	in_SE	in_p
rs10837042	11	38762222	A	G	0.598	1.00	0.165	0.04	0.0000288	0.0000572		0.580	1.00	-0.01049	0.01858	0.5719023
rs10837044	11	38764625	C	T	0.598	1.00	0.165	0.04	0.0000288	0.0000578		0.580	1.00	-0.01051	0.01858	0.5711699
rs10837045	11	38764650	A	C	0.402	1.00	-0.165	0.04	0.0000288	0.0000585		0.420	1.00	0.010608	0.01858	0.5676401
rs7949686	11	38766398	C	T	0.402	1.00	-0.165	0.04	0.0000288	0.0000591		0.420	1.00	0.010711	0.01857	0.5637212
rs7924767	11	38766974	A	G	0.402	1.00	-0.165	0.04	0.0000299	0.0000611		0.420	1.00	0.010719	0.01857	0.563561
rs7925174	11	38767305	A	G	0.402	1.00	-0.165	0.04	0.0000302	0.0000617		0.420	1.00	0.010739	0.01858	0.5630119
rs2901919	11	38767500	A	C	0.402	1.00	-0.165	0.04	0.0000314	0.0000637		0.420	1.00	0.010738	0.01858	0.5630651
rs7114799	11	38770039	A	T	0.598	1.00	0.164	0.04	0.0000322	0.0000652		0.580	1.00	-0.01075	0.01859	0.5629227
rs12360728	11	38773918	C	T	0.396	1.00	-0.166	0.04	0.0000291	0.0000637		0.412	0.94	0.011664	0.01918	0.5428038
rs10768414	11	38774275	C	T	0.396	1.00	-0.166	0.04	0.0000294	0.0000644		0.412	0.94	0.011658	0.01918	0.5430397
rs7951041	11	38778523	A	G	0.396	1.00	-0.165	0.04	0.0000311	0.0000677		0.412	0.94	0.011666	0.01919	0.542843
rs7938113	11	38788014	A	C	0.605	1.00	0.164	0.04	0.0000333	0.0000719		0.588	0.94	-0.0117	0.0192	0.5419764
rs2068461	11	38788518	C	T	0.605	1.00	0.164	0.04	0.0000343	0.0000737		0.588	0.94	-0.01171	0.0192	0.5418563
rs10768418	11	38789198	C	T	0.395	1.00	-0.164	0.04	0.0000353	0.0000756		0.412	0.94	0.011723	0.01921	0.5413405
rs1913056	11	38793624	C	T	0.395	1.00	-0.163	0.04	0.0000368	0.0000784		0.411	0.93	0.011773	0.01923	0.5400026
rs11034968	11	38796075	A	G	0.605	1.00	0.163	0.04	0.0000378	0.0000804		0.589	0.93	-0.01179	0.01923	0.5394657
rs7928323	11	38797635	A	C	0.394	1.00	-0.16	0.04	0.0000556	0.0001203		0.411	0.93	0.011797	0.01922	0.5389953
rs10837053	11	38798069	A	T	0.603	1.00	0.163	0.04	0.0000381	0.0000962		0.589	0.93	-0.01179	0.01922	0.5391244
rs10837055	11	38801750	A	C	0.603	1.00	0.163	0.04	0.0000368	0.0000946		0.589	0.93	-0.01179	0.01922	0.5392385
rs10837056	11	38803091	A	C	0.603	1.00	0.163	0.04	0.0000363	0.0000935		0.589	0.93	-0.01179	0.01921	0.5391002
rs10837057	11	38803115	A	G	0.603	1.00	0.164	0.04	0.0000359	0.0000926		0.589	0.93	-0.01179	0.01921	0.5389857
rs2861328	11	38803333	A	G	0.603	1.00	0.164	0.04	0.0000357	0.0000921		0.589	0.93	-0.01179	0.01921	0.5391195
rs4612778	11	38809762	C	T	0.397	1.00	-0.164	0.04	0.000035	0.0000902		0.411	0.93	0.011797	0.01921	0.5387975
rs2137359	11	38811328	C	T	0.603	1.00	0.164	0.04	0.0000346	0.0000893		0.589	0.93	-0.0118	0.01921	0.5387228
rs7930395	11	38816008	A	G	0.603	1.00	0.164	0.04	0.0000344	0.0000889		0.589	0.93	-0.01179	0.0192	0.538969
rs7930559	11	38816292	C	T	0.397	1.00	-0.164	0.04	0.0000339	0.0000876		0.411	0.93	0.011778	0.0192	0.5391879
rs11034972	11	38816598	C	T	0.397	1.00	-0.164	0.04	0.0000337	0.0000871		0.411	0.93	0.011777	0.0192	0.5392153
rs2861329	11	38821406	G	T	0.603	1.00	0.164	0.04	0.0000334	0.0000863		0.589	0.93	-0.01178	0.0192	0.5389141
rs2861331	11	38825943	G	T	0.397	1.00	-0.164	0.04	0.0000334	0.0000862		0.411	0.94	0.011787	0.01919	0.5387776
rs1913033	11	38826444	A	T	0.394	0.92	-0.166	0.041	0.0000555	0.0001046		0.406	0.92	0.011191	0.01936	0.5628253
rs10742471	11	39239502	C	T	0.346	0.99	-0.131	0.041	0.001387	0.0008952		0.385	0.97	0.005953	0.01915	0.7556615
rs4755505	11	39240453	A	C	0.654	0.99	0.131	0.041	0.001388	0.0008956		0.615	0.97	-0.00595	0.01914	0.7558348
rs12146430	11	59823115	G	T	0.045	0.98	-0.336	0.096	0.0004535	0.0015652		0.025	0.74	-0.02093	0.06396	0.7433327
rs4633490	11	59989342	C	T	0.560	1.00	-0.094	0.039	0.01588	0.0008857		0.548	0.99	-0.0373	0.01836	0.0420356
rs4471445	11	59989414	A	G	0.440	1.00	0.094	0.039	0.01586	0.0008843		0.452	0.99	0.037313	0.01836	0.0419455
rs1051756	11	59992848	A	G	0.434	0.99	0.095	0.039	0.01481	0.0009056		0.441	0.98	0.033819	0.01856	0.0681747
rs1941032	11	60004501	A	G	0.555	1.00	-0.098	0.039	0.01226	0.0006892		0.545	1.00	-0.037	0.01831	0.0431954
rs4939369	11	60013911	A	T	0.446	1.00	0.097	0.039	0.01301	0.0007911		0.455	1.00	0.037053	0.01833	0.0430474
rs8181503	11	60020879	C	T	0.434	0.99	0.095	0.039	0.01541	0.0007612		0.437	0.98	0.036162	0.01854	0.0509837
rs2298615	11	65108638	C	T	0.767	0.92	-0.152	0.048	0.001522	0.0009725		0.810	0.94	-0.00091	0.02353	0.9691086
rs17137594	11	78400902	A	G	0.947	0.98	0.301	0.089	0.0007412	0.0044627		0.940	0.83	-0.00648	0.04178	0.8765866
rs682366	11	85379389	C	T	0.798	0.99	0.158	0.048	0.0009942	0.0379117		0.810	0.99	-0.01296	0.0232	0.5759982
rs7951988	11	85391936	C	T	0.799	0.98	0.165	0.048	0.0006352	0.0120007		0.804	0.96	-0.00683	0.0234	0.7702404
rs2509608	11	85456945	G	T	0.804	0.98	0.161	0.049	0.0009515	0.0340395		0.815	0.98	-0.01755	0.0237	0.4585241
rs7934904	11	85580128	A	C	0.977	0.75	-0.473	0.152	0.001858	0.0006429		0.968	0.55	0.078144	0.06807	0.2505759
rs12364596	11	88068136	C	T	0.945	0.88	-0.316	0.091	0.0004842	0.0001507		0.970	0.62	-0.00677	0.06567	0.9178683
rs10830277	11	88848502	C	T	0.109	0.66	0.275	0.077	0.0003463	0.0005811		0.127	0.57	0.034761	0.03869	0.3685608
rs10830402	11	89482799	A	C	0.698	0.97	-0.143	0.043	0.0009398	0.0014943		0.658	0.88	0.012527	0.02062	0.5430866
rs10734125	11	89538489	G	T	0.690	1.00	-0.147	0.042	0.0004809	0.0008488		0.649	0.97	0.014515	0.01945	0.4550692
rs3758755	11	89543053	A	G	0.310	0.99	0.148	0.042	0.0004382	0.0007026		0.349	0.97	-0.01391	0.01955	0.4766305
rs10765270	11	89556910	A	G	0.691	1.00	-0.152	0.042	0.0003172	0.0004993		0.655	0.94	0.01155	0.01986	0.5604764
rs2155056	11	89602382	A	G	0.276	1.00	-0.134	0.043	0.001903	0.0002402		0.312	0.94	0.008921	0.02008	0.6565114
rs7941284	11	89605757	C	T	0.834	1.00	-0.214	0.053	0.0000469	0.0000337		0.831	0.91	0.004608	0.02616	0.8600587
rs7101892	11	89626315	A	G	0.708	0.97	0.134	0.043	0.00201	0.0002758		0.672	0.95	-0.0101	0.0197	0.6079425
rs1370018	11	90655656	C	T	0.917	1.00	0.234	0.071	0.0009628	0.0031512		0.918	1.00	0.030219	0.03391	0.3724509
rs3740862	11	94501936	A	G	0.069	1.00	0.273	0.077	0.00042	0.0035799		0.081	1.00	-0.05896	0.03369	0.0799199
rs10501905	11	98689812	C	T	0.868	0.98	-0.193	0.058	0.0008551	0.0282709		0.851	0.94	0.015644	0.02712	0.563644
rs1025888	11	99252539	C	T	0.506	1.00	0.126	0.039	0.001121	0.0003698		0.465	0.99	-0.00795	0.0183	0.6637653
rs11226159	11	103483750	G	T	0.273	1.00	0.139	0.044	0.001377	0.0002762		0.294	0.90	0.001346	0.02085	0.9484758
rs11226165	11	103485825	C	T	0.744	1.00	-0.154	0.045	0.0005221	0.0002198		0.736	0.90	-0.00393	0.02158	0.8553343
rs673545	11	107579542	A	G	0.012	0.40	0.737	0.291	0.01127	0.000299		0.013	0.35	-0.05645	0.1131	0.6174168
rs2640769	11	107899229	A	T	0.989	0.37	-0.876	0.313	0.005109	0.0001593		0.989	0.33	0.006163	0.13509	0.9635836
rs17112579	11	110683355	A	G	0.041	0.85	0.334	0.106	0.001658	0.0007162		0.051	0.87	0.038815	0.04667	0.4051584
rs7109448	11	110706518	A	C	0.951	1.00	-0.303	0.091	0.0008094	0.0005402		0.938	0.98	-0.04037	0.04037	0.3168657
rs1893989	11	110707688	A	G	0.949	0.98	-0.301	0.089	0.0007659	0.0007561		0.935	0.96	-0.0329	0.04013	0.4119159
rs10502141	11	110726764	A	C	0.049	1.00	0.306	0.091	0.0007443	0.0005082		0.062	0.99	0.040854	0.04033	0.3106924
rs10502142	11	110727016	C	T	0.049	1.00	0.306	0.091	0.0007398	0.0005055		0.062	0.99	0.040855	0.04033	0.3106689
rs7950136	11	110751864	G	T	0.952	1.00	-0.295	0.091	0.001254	0.0006072		0.940	0.99	-0.03029	0.04081	0.4575884
rs11213864	11	110756040	C	T	0.048	1.00	0.292	0.092	0.001443	0.000697		0.059	0.99	0.029172	0.04093	0.4756067
rs11213867	11	110756850	A	G	0.953	0.98	-0.298	0.093	0.001336	0.0005968		0.944	0.92	-0.01666	0.04399	0.7047163
rs2187472	11	110767823	A	C	0.960	0.94	-0.308	0.102	0.002571	0.0007218		0.946	0.95	-0.00919	0.04402	0.8344157
rs17112736	11	110768955	A	T	0.960	0.96	-0.3	0.102	0.003235	0.0009142		0.946	0.96	-0.00751	0.04389	0.8640262
rs10502155	11	111710681	A	G	0.074	0.92	0.282	0.078	0.000292	0.0046024		0.105	0.87	0.010462	0.03157	0.7401282
rs7120198	11	111718340	C	G	0.927	0.93	-0.284	0.078	0.0002479	0.0040157		0.895	0.87	-0.00874	0.03145	0.7808264
rs12801998	11	111721858	C	G	0.076	0.90	0.259	0.078	0.000838	0.0138294		0.110	0.85	0.003286	0.03124	0.9161555
rs4936321	11	114546799	A	C	0.549	0.99	0.108	0.039	0.005997	0.0002034		0.570	0.99	-0.02548	0.01839	0.1655472
rs1048932	11	114550060	A	C	0.417	0.99	-0.111	0.04	0.005139	0.0003552		0.415	0.98	0.029036	0.01857	0.1176312
rs10128746	11	114580646	C	T	0.048	1.00	0.314	0.091	0.0005891	0.0011136		0.047	0.84	-0.09017	0.04736	0.0567356
rs11215417	11	114582591	A	C	0.048	0.99	0.318	0.092	0.0005584	0.0010216		0.046	0.82	-0.09086	0.04796	0.0579854
rs3802858	11	114583702	C	T	0.454	0.94	-0.121	0.04	0.002527	0.0002101		0.430	0.93	0.028641	0.01886	0.1284787
rs11215418	11	114585104	A	T	0.952	0.98	-0.321	0.093	0.000536	0.0009545		0.954	0.81	0.09117	0.04831	0.0589827
rs10458966	11	114607968	C	T	0.530	0.97	0.112	0.04	0.004833	0.0008402		0.533	0.93	-0.01637	0.0189	0.3860684

					GOYA QC		GOYA Overweight/control			GOYA BMI continuous	known	IARC QC		IARC results		
MARKER	chr	position	A1	A2	FREQ1	Rsqr	Beta	SE	p	p	gene name	freq1	Rsqr	in_beta	in_SE	in_p
rs500713	11	116826186	C	T	0.081	0.84	0.258	0.077	0.0008563	0.0012412		0.086	0.69	0.039886	0.03912	0.3075263
rs2511847	11	118708569	A	G	0.137	0.99	-0.198	0.057	0.0004538	0.0001566		0.105	0.95	-0.02116	0.0295	0.4727225
rs2248863	11	118712551	A	G	0.137	1.00	-0.2	0.056	0.0004106	0.000137		0.104	0.98	-0.01976	0.02922	0.4985559
rs2248853	11	118712656	C	T	0.861	0.99	0.198	0.055	0.0003391	0.0001374		0.894	0.99	0.021961	0.02856	0.4415878
rs2511840	11	118713771	C	G	0.375	0.98	-0.126	0.04	0.001767	0.0005648		0.379	0.82	0.00934	0.02048	0.6481661
rs2509656	11	118713786	A	G	0.137	0.98	-0.202	0.057	0.0003899	0.0001301		0.109	0.80	-0.0197	0.03159	0.5325602
rs9640	11	118715189	A	T	0.137	0.98	-0.202	0.057	0.0003889	0.0001298		0.110	0.78	-0.01971	0.03181	0.5352557
rs7950427	11	125166832	C	T	0.881	0.87	0.219	0.065	0.0006983	0.0026225		0.876	0.82	0.018611	0.03147	0.5539337
rs12577641	11	125828547	C	G	0.811	0.99	0.168	0.05	0.0007589	0.0231601		0.793	0.93	-0.01852	0.02319	0.4240927
rs11220503	11	125838088	C	T	0.811	0.98	0.176	0.05	0.0004571	0.0154809		0.798	0.93	-0.01884	0.02332	0.4187876
rs11220504	11	125839955	C	T	0.186	0.96	-0.184	0.051	0.0003103	0.0109639		0.201	0.92	0.018815	0.02364	0.425801
rs11220506	11	125840539	A	G	0.183	0.95	-0.183	0.052	0.0004066	0.0125231		0.199	0.91	0.018807	0.0239	0.4309496
rs588957	11	128206751	C	T	0.707	0.99	-0.118	0.043	0.006032	0.0008668		0.721	1.00	-0.01755	0.02028	0.3864199
rs588472	11	128206861	A	G	0.325	0.97	0.132	0.042	0.001678	0.0003456		0.308	0.96	0.009997	0.02001	0.6169702
rs651925	11	128209926	C	T	0.320	0.97	0.13	0.042	0.002162	0.0002828		0.305	0.94	0.010664	0.0202	0.597276
rs4366492	11	130222466	C	T	0.570	0.98	0.119	0.04	0.002547	0.0007428		0.557	0.89	-0.01611	0.01966	0.4119611
rs10791097	11	130223840	G	T	0.564	1.00	0.117	0.039	0.002876	0.0009673		0.549	0.90	-0.01738	0.01955	0.3737348
rs10791098	11	130223921	G	T	0.564	1.00	0.117	0.039	0.00287	0.0009616		0.549	0.90	-0.01737	0.01955	0.3738768
rs10791099	11	130224023	C	G	0.564	1.00	0.117	0.039	0.002732	0.0009141		0.549	0.90	-0.01735	0.01955	0.3744856
rs10750450	11	130224271	G	T	0.436	1.00	-0.119	0.039	0.002473	0.0008259		0.451	0.90	0.017232	0.01956	0.3779596
rs4601795	11	130234640	C	T	0.556	0.99	0.124	0.039	0.001605	0.0005519		0.534	0.86	-0.01659	0.01994	0.4050879
rs10791100	11	130244156	A	T	0.477	1.00	0.134	0.039	0.0005485	0.0011429		0.497	1.00	-0.01023	0.0185	0.5799658
rs2236711	11	130244494	A	G	0.477	1.00	0.134	0.039	0.0005337	0.0011069		0.497	1.00	-0.01025	0.01849	0.5790432
rs4936123	11	130246177	A	G	0.477	1.00	0.135	0.039	0.0005172	0.0010932		0.497	1.00	-0.01064	0.01849	0.5648042
rs10894273	11	130247365	G	T	0.523	1.00	-0.135	0.039	0.0005017	0.0010805		0.503	1.00	0.011019	0.01848	0.5507821
rs1054869	11	130247840	A	G	0.477	1.00	0.135	0.039	0.0004958	0.0010756		0.498	1.00	-0.01113	0.01848	0.5468062
rs10160281	11	130247998	C	T	0.477	1.00	0.135	0.039	0.0005102	0.0011046		0.498	1.00	-0.01112	0.01849	0.5470288
rs12363140	11	130249478	A	C	0.523	1.00	-0.135	0.039	0.0005252	0.0011347		0.502	1.00	0.011124	0.01849	0.5470824
rs11222369	11	130249880	A	G	0.522	1.00	-0.133	0.039	0.0005901	0.0012644		0.502	1.00	0.01112	0.01849	0.5472272
rs1050071	11	130252441	C	G	0.535	0.97	-0.134	0.039	0.0006437	0.0015068		0.580	0.84	-0.00185	0.02039	0.927502
rs6590520	11	130252596	C	G	0.522	1.00	-0.133	0.039	0.0006328	0.0013487		0.502	1.00	0.011107	0.0185	0.5478866
rs3190331	11	130253341	A	G	0.522	1.00	-0.133	0.039	0.000621	0.0013253		0.502	1.00	0.011095	0.01851	0.5485318
rs7942621	11	130256518	C	G	0.522	1.00	-0.133	0.039	0.0006044	0.0012946		0.501	0.99	0.01106	0.01855	0.5506231
rs876641	11	130257250	A	C	0.522	1.00	-0.133	0.039	0.0006043	0.0012945		0.501	0.99	0.011056	0.01856	0.5510212
rs2155751	11	130260450	A	G	0.478	0.99	0.133	0.039	0.0006253	0.001302		0.500	0.99	-0.01104	0.01858	0.5520924
rs4457753	11	130260785	A	G	0.522	0.99	-0.133	0.039	0.0006267	0.0013026		0.500	0.99	0.011032	0.01858	0.5523933
rs4436551	11	130260949	A	G	0.479	0.99	0.133	0.039	0.0006289	0.0013035		0.500	0.99	-0.01104	0.01858	0.5521964
rs4264159	11	130260994	G	T	0.521	0.99	-0.133	0.039	0.0006383	0.0013106		0.500	0.99	0.011033	0.01859	0.552462
rs4456262	11	130261023	C	T	0.479	0.99	0.133	0.039	0.0006393	0.0013102		0.500	0.99	-0.01098	0.01863	0.5552361
rs2155752	11	130261208	C	T	0.521	0.99	-0.133	0.039	0.0006416	0.0013123		0.500	0.99	0.010982	0.01863	0.5552975
rs4459316	11	130261386	C	T	0.479	0.99	0.133	0.039	0.000642	0.0013123		0.500	0.99	-0.01098	0.01864	0.5553526
rs1893017	11	130261463	C	T	0.551	0.97	-0.133	0.039	0.0007807	0.0011028		0.519	0.95	0.012957	0.0189	0.4927421
rs1893018	11	130261842	C	T	0.479	0.99	0.133	0.039	0.0006459	0.0013145		0.500	0.98	-0.01098	0.01864	0.5554789
rs1893019	11	130261874	C	G	0.522	0.99	-0.133	0.039	0.0006522	0.0013179		0.512	0.94	0.011105	0.0191	0.5607015
rs10791103	11	130262371	A	G	0.479	0.99	0.133	0.039	0.0006537	0.0013206		0.501	0.98	-0.01089	0.01869	0.5597092
rs7106973	11	130263323	A	G	0.463	0.95	0.136	0.04	0.0006362	0.0009536		0.490	0.95	-0.0131	0.01891	0.4879656
rs7107595	11	130263766	A	G	0.480	0.98	0.132	0.039	0.000708	0.001362		0.501	0.98	-0.0109	0.0187	0.5596048
rs11222391	11	130297258	A	G	0.382	0.93	0.14	0.041	0.00068	0.0002358		0.377	0.96	0.009588	0.01925	0.6181651
rs7925214	11	130299463	C	T	0.475	0.97	-0.126	0.039	0.001325	0.0005094		0.494	0.93	0.000143	0.01917	0.9940423
rs11222395	11	130299565	A	C	0.504	0.99	-0.121	0.039	0.001894	0.0007709		0.524	0.91	0.001157	0.01925	0.9520453
rs7944782	11	130300908	G	T	0.526	0.98	0.125	0.039	0.001437	0.0005585		0.506	0.93	-0.00043	0.01913	0.9819864
rs10750455	11	130305540	C	T	0.499	0.94	-0.126	0.04	0.001575	0.0006686		0.526	0.90	0.004629	0.01947	0.8118798
rs10736592	11	130305782	C	T	0.499	0.94	-0.126	0.04	0.00156	0.0006621		0.526	0.90	0.004679	0.01945	0.8097614
rs1991899	11	130306859	A	G	0.459	1.00	-0.127	0.039	0.001064	0.0004665		0.483	1.00	0.002175	0.01853	0.9065065
rs1991898	11	130306996	C	G	0.491	0.97	-0.126	0.039	0.001388	0.000627		0.515	0.94	0.003829	0.019	0.8401718
rs12790299	11	130308844	A	G	0.491	0.97	-0.126	0.039	0.001394	0.0006313		0.515	0.94	0.003842	0.019	0.8396741
rs6590540	11	130310654	C	T	0.506	0.96	0.127	0.039	0.001326	0.0008775		0.483	0.92	-0.00358	0.01926	0.8524914
rs10894287	11	130313693	C	T	0.494	0.96	-0.127	0.039	0.001329	0.0008813		0.517	0.92	0.003585	0.01926	0.8522128
rs7106928	11	130314469	G	T	0.417	0.85	0.145	0.042	0.0006636	0.000337		0.407	0.83	-0.00393	0.02056	0.8483087
rs3910267	11	130315492	C	T	0.494	0.96	-0.127	0.039	0.001335	0.0008879		0.517	0.92	0.003603	0.01927	0.8515248
rs11222406	11	130319303	A	G	0.463	0.99	-0.127	0.039	0.001048	0.0006768		0.484	0.98	0.001516	0.01879	0.935648
rs3910266	11	130319482	A	C	0.494	0.96	-0.127	0.039	0.001355	0.0009092		0.516	0.92	0.003701	0.01929	0.8477163
rs10791104	11	130320103	A	T	0.494	0.96	-0.126	0.039	0.001399	0.0009571		0.516	0.92	0.003723	0.01929	0.8468286
rs7111478	11	130324838	A	G	0.461	1.00	-0.125	0.039	0.001311	0.0009992		0.480	0.98	0.001696	0.01875	0.927862
rs7942900	11	130344454	A	G	0.534	0.99	0.127	0.039	0.001059	0.0007453		0.516	0.99	-0.00154	0.01867	0.9340232
rs10750457	11	130355487	C	T	0.534	0.99	0.127	0.039	0.00111	0.0007844		0.516	0.99	-0.00154	0.01866	0.9340276
rs10791109	11	130355587	G	T	0.534	0.99	0.127	0.039	0.001133	0.0008024		0.516	0.99	-0.00154	0.01865	0.9340551
rs2535392	12	581907	C	T	0.612	1.00	-0.12	0.04	0.002642	0.0008623		0.557	0.97	0.018512	0.01879	0.3241331
rs2535393	12	581923	C	T	0.611	1.00	-0.119	0.04	0.002774	0.0009212		0.557	0.97	0.018546	0.01878	0.3228997
rs17800834	12	852255	C	G	0.135	0.99	-0.189	0.057	0.0009275	0.0043896		0.132	0.99	0.021992	0.02666	0.409005
rs11571475	12	892613	A	G	0.862	1.00	0.188	0.056	0.0008521	0.0045058		0.876	0.99	-0.02329	0.02693	0.3867651
rs4766076	12	3200273	A	C	0.254	0.88	0.142	0.047	0.002563	0.0002717		0.270	0.87	0.048786	0.02256	0.0304818
rs11062805	12	3742620	C	T	0.630	0.96	0.137	0.041	0.0007351	0.0016074		0.662	0.94	0.01112	0.01961	0.5703932
rs1800378	12	6042463	C	T	0.656	0.85	-0.146	0.044	0.0009366	0.001623		0.653	0.82	0.033795	0.02123	0.1111249
rs3181295	12	6214435	A	G	0.414	0.97	0.135	0.04	0.0007934	0.0008577		0.434	0.94	0.00744	0.01881	0.6922215
rs4371001	12	8414279	A	G	0.580	0.95	0.119	0.039	0.002575	0.0009658		0.517	0.16	-0.03483	0.04482	0.4366763
rs9668333	12	8420146	A	G	0.431	0.98	-0.122	0.039	0.001738	0.0005074		0.504	0.17	0.034351	0.04332	0.4274514
rs11055289	12	13267163	C	G	0.779	1.00	-0.158	0.046	0.0006272	0.0032961		0.804	1.00	0.040733	0.02266	0.0719976
rs17395410	12	13268231	A	G	0.221	1.00	0.158	0.046	0.0006284	0.0033598		0.196	1.00	-0.04045	0.02263	0.0736842
rs10845718	12	13270242	A	G	0.779	1.00	-0.158	0.046	0.0006331	0.0034636		0.804	1.00	0.040236	0.02262	0.0750553
rs11614016	12	13270907	A	T	0.229	0.94	0.157	0.047	0.0008685	0.0030973		0.207	0.92	-0.04821	0.02336	0.0389355
rs10772635	12	13271753	A	C	0.222	1.00	0.157	0.046	0.0006597	0.0037294		0.197	1.00	-0.03994	0.02259	0.0769114

					GOYA QC		GOYA Overweight/control			GOYA BMI continuous	known	IARC QC		IARC results		
MARKER	chr	position	A1	A2	FREQ1	Rsqr	Beta	SE	p	p	gene name	freq1	Rsqr	in_beta	in_SE	in_p
rs12825370	12	14551075	C	G	0.022	0.74	0.496	0.155	0.001421	0.000535		0.017	0.48	-0.09432	0.09915	0.3410829
rs11057069	12	16819777	G	T	0.038	0.45	0.525	0.153	0.0005869	0.0015379		0.039	0.33	-0.0583	0.07645	0.445302
rs3112079	12	17128382	G	T	0.309	0.99	0.133	0.042	0.001651	0.0008228		0.316	0.98	-0.03819	0.01995	0.055366
rs994882	12	17134429	C	T	0.692	0.99	-0.137	0.042	0.001182	0.0005751		0.687	0.96	0.037978	0.02013	0.0590901
rs994881	12	17134532	A	G	0.692	0.99	-0.137	0.042	0.001181	0.000575		0.687	0.96	0.038088	0.02017	0.0588454
rs12817084	12	17654581	C	T	0.105	0.89	-0.223	0.068	0.001002	0.0005204		0.108	0.82	-0.01723	0.03142	0.5832149
rs10505808	12	17679831	A	G	0.101	1.00	-0.238	0.066	0.0002841	0.0000656		0.089	0.98	-0.01371	0.03133	0.6612548
rs10840916	12	17958713	C	T	0.250	0.93	-0.151	0.047	0.001335	0.0008548		0.257	0.91	1.41E-05	0.02108	0.9994673
rs11043845	12	18132519	G	T	0.336	0.96	0.137	0.041	0.0009071	0.0034339		0.304	0.96	-0.00765	0.02044	0.7081329
rs11043876	12	18168644	A	T	0.185	1.00	0.164	0.05	0.0009294	0.0074695		0.147	0.99	-0.00178	0.02671	0.9466785
rs16913855	12	18178883	A	C	0.815	1.00	-0.165	0.05	0.0009138	0.0074524		0.853	1.00	0.002705	0.02658	0.9188793
rs12579910	12	18181910	C	T	0.185	1.00	0.165	0.05	0.0009047	0.0076667		0.148	1.00	-0.00272	0.02657	0.9182965
rs4309203	12	18190052	A	C	0.184	1.00	0.165	0.05	0.0009198	0.0080441		0.147	1.00	-0.00282	0.02655	0.9154328
rs16913870	12	18190358	C	G	0.816	0.91	-0.174	0.052	0.0008412	0.006841		0.851	0.92	0.003697	0.02759	0.8933229
rs17471532	12	18191771	A	G	0.107	0.78	0.232	0.071	0.0009912	0.0123626		0.078	0.69	0.022266	0.04116	0.5882217
rs11046043	12	21479435	C	T	0.079	1.00	0.27	0.072	0.00019	0.0002744		0.078	0.86	0.043791	0.03507	0.2114589
rs11046048	12	21483467	A	C	0.921	1.00	-0.269	0.072	0.0001898	0.0002759		0.924	1.00	-0.04367	0.03262	0.1803206
rs7309377	12	21484160	A	G	0.921	1.00	-0.27	0.072	0.0001887	0.0002755		0.924	1.00	-0.04366	0.03263	0.1805258
rs10841813	12	21489133	A	G	0.078	1.00	0.271	0.072	0.0001804	0.0002731		0.077	1.00	0.04344	0.03264	0.1829532
rs11046061	12	21492219	C	T	0.922	0.99	-0.273	0.073	0.0001756	0.0002721		0.923	1.00	-0.04332	0.03265	0.1842769
rs10841814	12	21494696	A	C	0.078	0.99	0.273	0.073	0.0001733	0.0002719		0.077	1.00	0.043214	0.03266	0.1854825
rs11046075	12	21512686	A	C	0.075	0.98	0.274	0.074	0.0002333	0.0004515		0.076	0.98	0.039823	0.03295	0.2264931
rs2192170	12	21524303	C	T	0.917	1.00	-0.242	0.071	0.0006828	0.002556		0.905	0.96	-0.03291	0.03223	0.3067749
rs11046081	12	21532444	A	T	0.085	0.99	0.245	0.071	0.0005466	0.0010872		0.096	0.96	0.029028	0.03212	0.36575
rs4762703	12	21536089	A	G	0.085	0.98	0.245	0.071	0.0005552	0.0010947		0.096	0.96	0.028836	0.03214	0.3692648
rs4480631	12	24017042	C	T	0.377	0.96	0.138	0.041	0.0006802	0.0033671		0.427	0.93	-0.04045	0.01908	0.0338711
rs11047188	12	24025393	C	T	0.410	0.97	0.132	0.04	0.000859	0.0051145		0.456	0.97	-0.03743	0.01853	0.0431731
rs11047189	12	24030031	G	T	0.637	1.00	-0.138	0.04	0.0005875	0.0035043		0.573	0.98	0.040379	0.01862	0.0299718
rs10505921	12	24031096	A	G	0.637	1.00	-0.138	0.04	0.0005718	0.0034238		0.572	0.98	0.040187	0.01861	0.0307104
rs1391781	12	24031367	C	T	0.630	1.00	-0.133	0.04	0.0008961	0.0046071		0.566	0.99	0.036593	0.01848	0.0475257
rs7975877	12	30959464	G	T	0.964	0.39	-0.602	0.169	0.000363	0.0069181		0.977	0.15	-0.06191	0.14064	0.6595054
rs7961486	12	32526504	A	G	0.048	0.63	0.367	0.115	0.001341	0.0003765		0.058	0.71	-0.05679	0.04723	0.2287656
rs7305985	12	32639399	A	T	0.980	0.86	-0.455	0.152	0.002694	0.0008582		0.982	0.81	-0.00631	0.06821	0.926181
rs17483228	12	37856840	C	T	0.041	0.86	0.384	0.107	0.0003382	0.0000197		0.042	0.89	0.050725	0.04863	0.2964889
rs17559744	12	38588128	C	T	0.063	0.89	0.265	0.085	0.001926	0.000435		0.054	0.80	0.020257	0.04434	0.6475311
rs28370608	12	38638136	A	G	0.051	0.79	0.332	0.099	0.0007834	0.0001819		0.035	0.67	0.020603	0.0588	0.7258332
rs17458459	12	38778704	A	G	0.035	0.79	0.403	0.119	0.0006742	0.000146		0.028	0.65	0.067382	0.06659	0.3112109
rs2698791	12	46097226	C	G	0.725	1.00	-0.147	0.043	0.0006973	0.0020138		0.737	1.00	-0.0242	0.02085	0.2452903
rs2720295	12	48457981	A	C	0.613	0.84	-0.159	0.043	0.0002222	0.0010626	FAIM2					
rs2720293	12	48461647	A	G	0.687	0.89	-0.19	0.044	0.0000165	0.0001293	FAIM2					
rs11836282	12	48463591	A	T	0.679	0.92	-0.188	0.043	0.0000122	0.0000884	FAIM2					
rs2603107	12	48470141	G	T	0.411	0.98	0.131	0.039	0.0009168	0.0045789	FAIM2					
rs1470909	12	48470233	C	T	0.410	0.98	0.13	0.039	0.0009409	0.004671	FAIM2					
rs2603105	12	48474701	A	T	0.340	0.96	0.166	0.041	0.0000597	0.0002183	FAIM2					
rs1075366	12	48478276	C	T	0.410	0.99	0.129	0.039	0.0009675	0.0047567	FAIM2					
rs2603112	12	48486216	A	G	0.628	1.00	-0.144	0.04	0.0003021	0.0013052	FAIM2					
rs7313563	12	48490604	A	C	0.372	1.00	0.144	0.04	0.0003022	0.0013295	FAIM2					
rs11169162	12	48493393	C	T	0.373	0.99	0.144	0.04	0.0002917	0.001303	FAIM2					
rs11169163	12	48494610	A	G	0.639	0.96	-0.153	0.041	0.0001944	0.0008138	FAIM2					
rs4075681	12	48500904	C	T	0.654	0.94	-0.17	0.042	0.0000426	0.0001447	FAIM2					
rs11169167	12	48503068	C	T	0.607	1.00	0.148	0.039	0.0001672	0.002277	FAIM2					
rs1031477	12	48504911	C	T	0.431	1.00	-0.161	0.039	0.0000361	0.0004947	FAIM2					
rs11169170	12	48509280	C	T	0.465	0.97	-0.17	0.039	0.0000118	0.0001266	FAIM2					
rs4391887	12	48511517	A	G	0.367	0.96	-0.155	0.041	0.0001519	0.0019316	FAIM2					
rs10875976	12	48512734	A	G	0.534	0.96	0.173	0.039	0.0000108	0.0001307	FAIM2					
rs4898534	12	48517292	A	G	0.466	0.96	-0.173	0.039	0.0000108	0.0001309	FAIM2					
rs7973894	12	48519523	A	G	0.535	0.95	0.173	0.039	0.0000109	0.0001345	FAIM2					
rs4898536	12	48526417	C	T	0.312	1.00	-0.146	0.042	0.0004666	0.0020903	FAIM2					
rs11169176	12	48527280	A	G	0.486	0.96	0.186	0.04	0.0000031	0.0000177	FAIM2					
rs4898537	12	48529609	C	G	0.313	0.99	-0.146	0.042	0.0004752	0.0020745	FAIM2					
rs10875980	12	48531034	A	T	0.528	1.00	-0.184	0.039	0.00000274	0.0000103	FAIM2					
rs10875982	12	48531973	A	G	0.528	1.00	-0.183	0.039	0.00000281	0.0000102	FAIM2					
rs7138803	12	48533735	A	G	0.415	1.00	0.215	0.04	6.36E-08	0.000000462	FAIM2					
rs10783321	12	48535870	C	T	0.315	0.99	-0.143	0.042	0.000604	0.0022732	FAIM2					
rs4898539	12	48538177	G	T	0.315	0.99	-0.143	0.042	0.0006024	0.0022733	FAIM2					
rs1975732	12	48544213	C	T	0.315	0.99	-0.143	0.042	0.0005969	0.0022749	FAIM2					
rs3741557	12	48544592	G	T	0.683	0.96	0.149	0.042	0.0004179	0.0015347	FAIM2					
rs9805137	12	48545329	A	T	0.657	0.89	0.151	0.043	0.000487	0.0021742	FAIM2					
rs2271688	12	48545796	A	C	0.321	0.95	-0.155	0.043	0.0002619	0.0008678	FAIM2					
rs7132908	12	48549415	A	G	0.440	0.98	0.233	0.04	6.07E-09	1.83E-08	FAIM2					
rs706798	12	48552431	A	G	0.705	1.00	0.162	0.042	0.0001282	0.0004002	FAIM2					
rs897057	12	48552546	C	T	0.705	1.00	0.162	0.042	0.000129	0.0004012	FAIM2					
rs706797	12	48553125	C	G	0.323	0.95	-0.146	0.042	0.0005766	0.0009446	FAIM2					
rs1248263	12	48557639	G	T	0.280	1.00	-0.161	0.043	0.0001863	0.0001151	FAIM2					
rs836959	12	48561404	A	C	0.280	1.00	-0.161	0.043	0.0001845	0.0001121	FAIM2					
rs383307	12	48563396	G	T	0.729	0.96	0.163	0.044	0.0002372	0.0001436	FAIM2					
rs423934	12	48563679	C	T	0.720	1.00	0.162	0.043	0.0001707	0.0001068	FAIM2					
rs297924	12	48571328	C	T	0.413	1.00	0.176	0.04	0.00000797	0.00000383	FAIM2					
rs17201502	12	48571829	C	T	0.617	1.00	-0.176	0.04	0.0000111	0.00000266	FAIM2					
rs1465058	12	51901260	A	G	0.897	0.97	-0.205	0.066	0.001784	0.0005377		0.883	0.99	0.001615	0.0289	0.955391
rs4759282	12	52358702	A	C	0.309	0.97	0.101	0.042	0.0169	0.0007551		0.291	0.95	-0.02383	0.02023	0.2384659
rs7963016	12	52359086	A	T	0.311	0.99	0.103	0.042	0.01374	0.0006654		0.292	0.98	-0.02416	0.01992	0.2247723

					GOYA QC		GOYA Overweight/control			GOYA BMI continuous	known	IARC QC		IARC results		
MARKER	chr	position	A1	A2	FREQ1	Rsqr	Beta	SE	p	p	gene name	freq1	Rsqr	in_beta	in_SE	in_p
rs7976793	12	52359336	G	T	0.688	0.99	-0.103	0.042	0.01362	0.0006672		0.708	0.98	0.024159	0.01991	0.2245805
rs7312853	12	52359864	C	G	0.688	0.99	-0.103	0.042	0.0135	0.0006699		0.708	0.98	0.024155	0.0199	0.2245412
rs2355001	12	57149128	A	G	0.459	1.00	0.113	0.039	0.003849	0.0009542		0.424	1.00	0.013905	0.01839	0.4491749
rs2354999	12	57149218	C	G	0.459	1.00	0.113	0.039	0.003836	0.0009495		0.424	1.00	0.013918	0.01839	0.4487528
rs2883575	12	57149374	A	G	0.459	1.00	0.113	0.039	0.003823	0.0009451		0.424	1.00	0.013968	0.01839	0.4470938
rs7311412	12	57150423	C	G	0.562	0.96	-0.121	0.04	0.002369	0.0007153		0.590	0.97	-0.01315	0.01862	0.4795871
rs10783895	12	57150938	C	T	0.562	0.96	-0.121	0.04	0.002359	0.0007123		0.589	0.97	-0.01323	0.01863	0.4773175
rs7969824	12	57157880	A	T	0.566	0.96	-0.126	0.04	0.001624	0.0005137		0.596	0.97	-0.0138	0.01867	0.4592147
rs3847723	12	57158228	C	G	0.539	1.00	-0.116	0.039	0.003104	0.0007933		0.575	1.00	-0.0142	0.01838	0.4394922
rs12312133	12	57158555	C	T	0.441	0.96	0.124	0.04	0.001902	0.0005891		0.412	0.97	0.013299	0.0186	0.4741363
rs11172627	12	57158938	A	G	0.538	0.92	-0.134	0.041	0.0009941	0.0004073		0.572	0.93	-0.01481	0.01897	0.4346113
rs10783899	12	57159166	C	T	0.538	1.00	-0.116	0.039	0.003059	0.0007851		0.575	1.00	-0.01422	0.01836	0.4380916
rs3913091	12	57159719	G	T	0.442	0.96	0.124	0.04	0.001874	0.0005822		0.412	0.97	0.013308	0.01857	0.4733358
rs3913092	12	57159862	A	G	0.442	0.96	0.124	0.04	0.001862	0.0005797		0.412	0.97	0.01332	0.01857	0.4727843
rs11172630	12	57161148	C	T	0.463	1.00	0.116	0.039	0.002928	0.0007771		0.426	1.00	0.014237	0.01834	0.4371239
rs12317552	12	61326877	C	T	0.938	0.98	0.265	0.082	0.001218	0.0003172		0.966	0.96	0.020411	0.0468	0.6624717
rs17732506	12	61327574	G	T	0.062	0.99	-0.265	0.082	0.001207	0.0003156		0.034	0.97	-0.02117	0.04677	0.6505384
rs10506445	12	61331522	A	G	0.937	1.00	0.265	0.081	0.001031	0.0002811		0.960	1.00	0.035226	0.04358	0.4185525
rs17676109	12	61336518	G	T	0.064	0.99	-0.261	0.081	0.001251	0.0003653		0.041	0.98	-0.03557	0.04366	0.4147975
rs12297077	12	66161447	A	C	0.043	0.94	0.284	0.1	0.004539	0.0006515		0.025	0.55	0.002386	0.06973	0.9726782
rs12319640	12	66164606	C	T	0.955	1.00	-0.286	0.095	0.002561	0.0003663		0.974	0.53	-0.00447	0.06964	0.9488194
rs12299984	12	66164667	G	T	0.041	0.81	0.355	0.111	0.001322	0.0000808		0.031	0.44	-0.04697	0.07485	0.5299502
rs12309810	12	66166309	A	G	0.955	1.00	-0.287	0.095	0.002476	0.0003562		0.974	0.53	-0.00488	0.06961	0.9440177
rs12305709	12	66171054	C	T	0.954	0.92	-0.3	0.098	0.002153	0.0003533		0.972	0.51	-0.00843	0.06864	0.9022195
rs1578876	12	66171198	A	G	0.046	0.91	0.302	0.098	0.002135	0.0003578		0.029	0.51	0.008974	0.06826	0.895318
rs12306340	12	66171879	A	G	0.046	0.90	0.303	0.099	0.002117	0.0003612		0.029	0.51	0.009353	0.06788	0.8903206
rs12830473	12	67785127	A	T	0.749	0.77	0.179	0.051	0.0004658	0.0014577		0.763	0.65	0.004513	0.0262	0.8631542
rs1879914	12	70772216	A	G	0.192	1.00	0.162	0.049	0.0009578	0.0026027		0.191	1.00	-0.00093	0.02326	0.9679911
rs10784950	12	70776561	A	G	0.192	1.00	0.162	0.049	0.0009562	0.0026425		0.191	1.00	-0.00098	0.02326	0.9664575
rs2367897	12	70778371	A	G	0.809	1.00	-0.162	0.049	0.0009554	0.0026629		0.809	1.00	0.001299	0.02326	0.9554133
rs7133520	12	70780674	A	G	0.192	1.00	0.162	0.049	0.000961	0.0026719		0.191	1.00	-0.00128	0.02326	0.9561212
rs7977245	12	70784135	A	G	0.809	1.00	-0.162	0.049	0.0009642	0.0026764		0.809	1.00	0.001265	0.02327	0.9566089
rs6582086	12	70784458	A	G	0.192	1.00	0.162	0.049	0.0009662	0.0026795		0.191	0.96	-0.00052	0.02379	0.9825898
rs1484834	12	70787031	A	G	0.195	0.98	0.162	0.049	0.0009962	0.0027587		0.193	0.95	-0.00115	0.02377	0.9612353
rs985207	12	70787623	A	G	0.192	1.00	0.162	0.049	0.0009738	0.0026928		0.190	0.96	-0.00043	0.02383	0.9855763
rs1484833	12	70788253	A	T	0.809	1.00	-0.162	0.049	0.0009848	0.0027125		0.810	0.96	0.000379	0.02386	0.9873297
rs1508271	12	73580811	C	T	0.987	0.48	0.624	0.25	0.01246	0.0006492		0.983	0.35	-0.04816	0.1039	0.6426902
rs10879997	12	74559773	A	C	0.067	1.00	-0.27	0.077	0.0004549	0.0006214		0.071	1.00	0.02008	0.03595	0.5761056
rs1795669	12	74559959	A	G	0.067	1.00	-0.27	0.077	0.0004549	0.000621		0.072	1.00	0.019716	0.03594	0.582978
rs11180815	12	74729174	C	T	0.199	0.50	0.196	0.069	0.004452	0.000965		0.230	0.50	0.009042	0.03099	0.7702698
rs12831663	12	74730398	A	G	0.801	0.50	-0.196	0.069	0.004478	0.0009605		0.770	0.50	-0.00907	0.03099	0.7695713
rs1433363	12	74750874	C	T	0.201	0.39	0.276	0.077	0.000346	0.0000302		0.226	0.38	-0.00192	0.03681	0.9584544
rs10778828	12	80641347	A	C	0.236	0.98	-0.164	0.046	0.0003832	0.0004111		0.227	0.94	-0.04043	0.02226	0.069148
rs1343933	12	80647432	A	C	0.764	0.99	0.162	0.046	0.0004118	0.0004455		0.773	0.95	0.04078	0.02223	0.0663873
rs11615729	12	80652449	A	T	0.234	0.98	-0.163	0.046	0.0004265	0.0003475		0.223	0.93	-0.04503	0.02259	0.0460859
rs2244802	12	80677880	C	G	0.313	1.00	-0.142	0.042	0.0007061	0.0012581		0.292	0.99	-0.03994	0.02018	0.0476627
rs10862355	12	80686829	C	T	0.756	1.00	0.155	0.045	0.0005968	0.0007193		0.767	1.00	0.039128	0.02152	0.0688922
rs12579530	12	80688061	A	C	0.763	1.00	0.159	0.046	0.0005043	0.0006872		0.778	1.00	0.043231	0.0218	0.0472361
rs12579544	12	80688174	A	C	0.763	1.00	0.159	0.046	0.0005041	0.0006871		0.778	1.00	0.043198	0.0218	0.0474052
rs11115007	12	80688804	A	T	0.237	1.00	-0.159	0.046	0.0005041	0.000687		0.222	1.00	-0.04313	0.0218	0.0477455
rs11115008	12	80688990	A	G	0.237	1.00	-0.159	0.046	0.000504	0.000687		0.222	1.00	-0.043	0.0218	0.048443
rs11115009	12	80689540	A	C	0.763	1.00	0.159	0.046	0.0005039	0.0006869		0.778	1.00	0.042964	0.0218	0.0486199
rs11115010	12	80690026	G	T	0.763	1.00	0.159	0.046	0.0005037	0.0006868		0.779	1.00	0.042858	0.0218	0.0491624
rs7300900	12	80690952	G	T	0.783	0.98	0.161	0.047	0.0007013	0.001101		0.796	0.98	0.039724	0.0225	0.0771993
rs17748942	12	80694609	A	G	0.219	1.00	-0.156	0.047	0.0008729	0.0011474		0.207	1.00	-0.03832	0.02224	0.0846768
rs17692407	12	80695500	C	G	0.219	1.00	-0.156	0.047	0.0008808	0.0011502		0.207	1.00	-0.03792	0.02224	0.0879424
rs17692461	12	80695886	C	G	0.781	1.00	0.156	0.047	0.0008849	0.0011518		0.793	1.00	0.037714	0.02224	0.089627
rs11613196	12	80697382	A	T	0.219	1.00	-0.156	0.047	0.0008869	0.0011512		0.207	1.00	-0.0376	0.02223	0.0905871
rs7956826	12	80697496	A	G	0.781	1.00	0.156	0.047	0.0008865	0.001148		0.794	1.00	0.037547	0.02223	0.0910353
rs11115015	12	80698674	C	T	0.219	1.00	-0.156	0.047	0.0008862	0.0011463		0.207	1.00	-0.0375	0.02223	0.0914479
rs7306395	12	80699998	C	T	0.219	1.00	-0.156	0.047	0.0008852	0.0011388		0.206	1.00	-0.03729	0.02223	0.093216
rs11115017	12	80701045	A	C	0.219	1.00	-0.156	0.047	0.0008837	0.0011361		0.206	1.00	-0.03724	0.02223	0.0936883
rs17749243	12	80702210	A	C	0.781	1.00	0.156	0.047	0.0008837	0.001136		0.794	1.00	0.037237	0.02223	0.0937035
rs17749267	12	80702439	A	G	0.219	1.00	-0.156	0.047	0.0008842	0.0011365		0.206	1.00	-0.03723	0.02223	0.0937526
rs7965168	12	80708598	A	C	0.219	1.00	-0.155	0.047	0.0009636	0.0012157		0.207	1.00	-0.03755	0.02226	0.0913745
rs11610365	12	80742239	C	T	0.786	0.93	0.163	0.049	0.0008121	0.0014666		0.788	0.93	0.036117	0.02304	0.116726
rs12829448	12	92990845	C	T	0.226	0.86	-0.125	0.049	0.01165	0.0009338		0.195	0.80	-0.02885	0.02473	0.2430061
rs17022644	12	93468035	A	G	0.067	0.95	0.265	0.08	0.0008724	0.0033776		0.067	0.91	0.016644	0.038	0.6611009
rs12814712	12	93884840	A	G	0.103	0.97	-0.222	0.065	0.0006723	0.0212957		0.095	0.92	0.00189	0.032	0.9528498
rs11108830	12	96048333	G	T	0.076	0.94	0.218	0.076	0.003952	0.0007993		0.059	0.96	-0.04606	0.04134	0.2648571
rs11609353	12	96184422	C	T	0.955	0.34	0.591	0.165	0.0003291	0.0000482		0.939	0.38	-0.03822	0.06163	0.5347745
rs7962117	12	96443183	A	G	0.824	0.93	-0.152	0.053	0.004106	0.0008044		0.846	0.92	0.033737	0.02624	0.1981822
rs1349414	12	96463114	A	C	0.812	0.97	-0.161	0.051	0.001464	0.000243		0.838	0.95	0.024164	0.02518	0.3368894
rs12370302	12	96463920	C	T	0.134	0.88	0.197	0.061	0.001239	0.0000594		0.126	0.87	-0.03442	0.02934	0.240367
rs1450989	12	96466976	C	G	0.188	0.97	0.161	0.051	0.001455	0.0002412		0.163	0.95	-0.02242	0.02516	0.3726319
rs10507090	12	96469343	G	T	0.866	0.88	-0.197	0.061	0.001226	0.0000586		0.874	0.87	0.03207	0.02934	0.2740604
rs11109106	12	96476013	G	T	0.807	0.94	-0.159	0.051	0.001667	0.0002629		0.830	0.91	0.022309	0.02529	0.3772996
rs10860219	12	96483923	A	G	0.814	0.96	-0.166	0.051	0.0011	0.0001848		0.838	0.94	0.020684	0.02523	0.4118628
rs11109114	12	96494269	A	G	0.825	0.98	-0.161	0.052	0.001874	0.0003169		0.843	0.96	0.022848	0.02537	0.3674326
rs7960848	12	97057608	A	G	0.449	1.00	0.135	0.039	0.0005904	0.0142855		0.425	0.92	0.007186	0.01913	0.7070326
rs7960881	12	97057789	C	T	0.551	1.00	-0.135	0.039	0.0005928	0.0142927		0.575	0.92	-0.00716	0.01913	0.7080346
rs12315137	12	97058620	A	G	0.449	1.00	0.134	0.039	0.0005951	0.0142945		0.426	0.93	0.006292	0.01906	0.7411105

					GOYA QC		GOYA Overweight/control			GOYA BMI continuous	known	IARC QC		IARC results		
MARKER	chr	position	A1	A2	FREQ1	Rsqr	Beta	SE	p	p	gene name	freq1	Rsqr	in_beta	in_SE	in_p
rs11109356	12	97059649	C	T	0.551	1.00	-0.134	0.039	0.0006014	0.0142981		0.574	0.93	-0.00625	0.01906	0.7426617
rs10778007	12	98617214	A	G	0.843	1.00	0.177	0.053	0.000903	0.0028154		0.868	0.99	0.039729	0.0266	0.1349411
rs10860494	12	98617554	A	C	0.157	1.00	-0.177	0.053	0.0008936	0.0027818		0.133	1.00	-0.0397	0.02659	0.1350957
rs1500660	12	98618910	G	T	0.157	1.00	-0.177	0.053	0.000886	0.0027545		0.133	1.00	-0.03948	0.0265	0.136069
rs7955548	12	98620971	C	T	0.833	1.00	0.171	0.052	0.0009989	0.0024078		0.855	1.00	0.026687	0.02558	0.296356
rs4986685	12	98621431	A	G	0.157	1.00	-0.177	0.053	0.0008917	0.0027848		0.133	1.00	-0.03948	0.02651	0.1361122
rs4986687	12	98622879	C	T	0.843	1.00	0.177	0.053	0.0008947	0.0028001		0.868	1.00	0.03948	0.02651	0.1361272
rs6538945	12	98676267	C	T	0.833	1.00	0.172	0.052	0.0009067	0.0025226		0.855	0.98	0.027265	0.02581	0.2903689
rs11110031	12	98688498	A	T	0.835	0.99	0.181	0.052	0.0005573	0.0017253		0.854	0.95	0.028012	0.02615	0.2836622
rs7979049	12	98690370	A	G	0.830	0.97	0.175	0.052	0.0008411	0.0021576		0.847	0.91	0.030971	0.02618	0.2365227
rs10860519	12	98693967	C	T	0.836	0.99	0.185	0.053	0.0004255	0.0014022		0.853	0.94	0.028218	0.02622	0.2814614
rs7309802	12	106564812	G	T	0.675	1.00	0.142	0.042	0.0007048	0.014896		0.670	0.97	-0.02918	0.01934	0.1310831
rs2241187	12	106569269	G	T	0.224	1.00	-0.191	0.047	0.0000545	0.0007772		0.218	1.00	0.015567	0.02166	0.472009
rs4964670	12	107218887	A	G	0.245	1.00	-0.127	0.045	0.004901	0.0009332		0.240	0.99	-0.02318	0.0215	0.2805094
rs12311469	12	107375209	G	T	0.919	1.00	0.237	0.071	0.0008504	0.00229		0.931	0.97	0.024012	0.03546	0.4978998
rs10861927	12	107375691	A	G	0.081	1.00	-0.237	0.071	0.0008533	0.0022867		0.068	0.97	-0.02403	0.03551	0.4982233
rs2029852	12	115659649	C	G	0.580	0.77	-0.151	0.045	0.0007152	0.003792		0.564	0.77	0.01469	0.02109	0.4857368
rs637368	12	116624195	C	T	0.675	1.00	-0.147	0.041	0.0003648	0.001605		0.680	0.46	0.000667	0.02885	0.9815264
rs16939761	12	117029587	A	G	0.066	0.91	-0.227	0.082	0.005692	0.0008861		0.098	0.90	0.053568	0.03293	0.1035024
rs2293444	12	117058565	A	C	0.923	0.92	0.196	0.076	0.009597	0.0007438		0.896	0.97	-0.04812	0.03087	0.1188167
rs7977921	12	117060993	C	T	0.079	0.96	-0.198	0.074	0.007442	0.000565		0.103	0.97	0.044971	0.03096	0.146089
rs11068953	12	117373942	A	G	0.675	0.52	0.231	0.057	0.0000566	0.0000284		0.673	0.19	-0.02009	0.04342	0.6433257
rs1167725	12	119452942	A	T	0.200	0.82	0.134	0.053	0.01122	0.0009906		0.161	0.75	0.006087	0.0274	0.8240469
rs4766974	12	119553498	A	G	0.288	1.00	0.162	0.042	0.0001409	0.0016451		0.312	0.96	-0.0106	0.02025	0.600327
rs12370780	12	119555356	C	T	0.712	1.00	-0.161	0.042	0.0001447	0.0016811		0.688	0.96	0.010615	0.02025	0.5998342
rs668622	12	119682682	A	G	0.535	1.00	0.113	0.039	0.00409	0.0007908		0.585	1.00	-0.00019	0.01862	0.991652
rs1678960	12	120749018	C	T	0.179	0.89	0.189	0.054	0.000467	0.0039252		0.201	0.87	-0.01169	0.02432	0.6304685
rs1449562	12	120772268	A	G	0.464	1.00	-0.122	0.039	0.001687	0.0009037		0.486	1.00	-0.00274	0.01822	0.8804269
rs2707070	12	120774822	A	G	0.464	1.00	-0.122	0.039	0.001673	0.0008974		0.486	1.00	-0.00274	0.01822	0.8804712
rs2596140	12	120777345	G	T	0.464	1.00	-0.123	0.039	0.001561	0.0008492		0.486	1.00	-0.00274	0.01822	0.8805646
rs2928283	12	120788138	C	T	0.464	1.00	-0.125	0.039	0.001225	0.0007019		0.486	1.00	-0.00274	0.01822	0.8805594
rs7309105	12	120806082	A	G	0.717	1.00	0.154	0.043	0.0003159	0.000967		0.675	1.00	0.01799	0.01944	0.3542662
rs483416	12	120822496	A	G	0.283	1.00	-0.153	0.043	0.0003336	0.0010126		0.325	1.00	-0.01828	0.01946	0.3470583
rs1168658	12	120822949	C	T	0.717	1.00	0.153	0.043	0.0003354	0.001017		0.675	1.00	0.018293	0.01946	0.346734
rs3825172	12	120824858	C	T	0.707	0.97	0.162	0.043	0.0001636	0.0006448		0.670	0.98	0.016599	0.01956	0.3957876
rs1043307	12	120838179	A	G	0.730	0.94	0.155	0.045	0.0005481	0.0014357		0.687	0.94	0.017906	0.0202	0.3749029
rs2272135	12	120840540	C	T	0.718	0.99	0.151	0.043	0.0004508	0.0013721		0.675	0.99	0.018998	0.0195	0.3294532
rs1720068	12	120848078	C	T	0.694	0.92	0.156	0.044	0.0003599	0.0013428		0.661	0.94	0.019702	0.01984	0.3203791
rs12578258	12	120853536	A	G	0.719	0.99	0.146	0.043	0.0007151	0.0021462		0.674	0.98	0.021043	0.01957	0.2817935
rs7313748	12	120854145	C	T	0.719	0.99	0.146	0.043	0.0007232	0.0021624		0.674	0.98	0.021053	0.01957	0.2815693
rs2309275	12	120885196	C	T	0.720	1.00	0.142	0.043	0.0009261	0.0019601		0.674	1.00	0.025754	0.01944	0.1849979
rs1169081	12	120890295	G	T	0.720	1.00	0.144	0.043	0.0008199	0.0017108		0.674	1.00	0.025758	0.01944	0.1848892
rs830121	12	120911544	A	C	0.279	0.99	-0.147	0.043	0.0006771	0.0012876		0.330	0.99	-0.02329	0.01951	0.2321483
rs11057513	12	123254454	C	G	0.095	0.79	0.257	0.075	0.0006076	0.0022364		0.070	0.77	0.025641	0.0388	0.5083326
rs1268892	12	123411366	G	T	0.051	0.80	-0.334	0.1	0.0008808	0.0303073		0.058	0.69	0.132032	0.04936	0.0074384
rs1244068	12	123411866	A	G	0.051	0.84	-0.328	0.098	0.0008437	0.0306567		0.058	0.70	0.131035	0.049	0.0074556
rs906302	12	123416410	C	G	0.949	0.88	0.325	0.096	0.0007039	0.0290638		0.942	0.71	-0.12979	0.04849	0.0074102
rs1244050	12	123428650	A	G	0.051	0.89	-0.329	0.096	0.0005649	0.0257678		0.057	0.71	0.126779	0.04876	0.009274
rs4765158	12	123658249	A	C	0.602	0.99	0.134	0.04	0.000763	0.0009616		0.596	0.47	0.075364	0.02704	0.005294
rs1726353	12	123793273	A	G	0.408	1.00	-0.129	0.039	0.0009253	0.0024172		0.422	1.00	-0.0011	0.01836	0.9522786
rs10847146	12	125454321	A	G	0.750	0.96	-0.128	0.046	0.005419	0.0003517		0.800	0.94	-0.00433	0.02313	0.8514237
rs12368748	12	125454941	C	G	0.758	1.00	-0.129	0.046	0.004592	0.0002508		0.809	1.00	-0.00048	0.02288	0.9831639
rs1000000	12	125456933	A	G	0.242	1.00	0.129	0.046	0.004596	0.0002508		0.192	1.00	0.000469	0.02287	0.9836424
rs7957025	12	125457919	A	G	0.242	1.00	0.129	0.046	0.004596	0.0002508		0.192	1.00	0.000459	0.02287	0.9839681
rs7315833	12	125459435	C	T	0.242	1.00	0.129	0.046	0.004596	0.0002508		0.192	1.00	0.000298	0.02283	0.9895744
rs11058635	12	125460777	C	T	0.242	1.00	0.129	0.046	0.004618	0.0002529		0.192	1.00	0.000398	0.02284	0.9860858
rs7972985	12	125461949	G	T	0.749	0.96	-0.131	0.046	0.004367	0.0002799		0.798	0.94	-0.00105	0.02308	0.9638379
rs7132131	12	125464326	A	G	0.242	1.00	0.127	0.046	0.005098	0.000318		0.193	0.99	0.000696	0.02285	0.975698
rs2347016	12	125469984	A	T	0.758	1.00	-0.127	0.046	0.005279	0.0003435		0.807	0.99	-0.00061	0.02287	0.9787223
rs6489076	12	125474620	A	C	0.254	1.00	0.124	0.045	0.005787	0.0002015		0.200	0.98	-0.00212	0.02267	0.9253652
rs6489077	12	125474637	C	T	0.253	1.00	0.124	0.045	0.005853	0.0002075		0.200	0.98	-0.00212	0.02267	0.9253724
rs6489079	12	125474791	A	G	0.253	1.00	0.124	0.045	0.005886	0.0002107		0.200	0.98	-0.00216	0.02268	0.9240777
rs6489080	12	125474816	C	G	0.236	0.97	0.109	0.047	0.0186	0.0006751		0.184	0.95	-0.00544	0.02396	0.820264
rs6489082	12	125475065	A	G	0.747	1.00	-0.124	0.045	0.005905	0.0002127		0.800	0.98	0.00218	0.02268	0.9233414
rs10773271	12	125475286	G	T	0.253	0.99	0.123	0.045	0.006016	0.0002237		0.200	0.98	-0.00219	0.02268	0.923041
rs10744255	12	125475468	C	G	0.748	0.99	-0.122	0.045	0.006749	0.0003007		0.800	0.98	0.002687	0.02269	0.9056346
rs10773272	12	125475975	C	G	0.757	0.99	-0.116	0.046	0.011	0.0007808		0.807	0.98	0.002272	0.02297	0.9211538
rs2286627	12	125476392	C	T	0.757	1.00	-0.116	0.046	0.01107	0.0007918		0.807	0.98	0.002309	0.02295	0.9198118
rs887335	12	125481356	A	C	0.766	1.00	-0.134	0.046	0.003446	0.0004418		0.809	1.00	0.004913	0.02294	0.8302314
rs2302271	12	125484541	C	G	0.234	1.00	0.134	0.046	0.003438	0.0004409		0.191	1.00	-0.00484	0.02295	0.8328442
rs10492047	12	125501283	A	C	0.937	1.00	-0.255	0.081	0.001594	0.0008761		0.950	1.00	-0.01101	0.03959	0.78082
rs4760085	12	129215496	C	G	0.049	0.88	-0.301	0.097	0.001939	0.0007765		0.057	0.82	-0.02779	0.04416	0.5288582
rs1566672	12	129220149	A	G	0.063	0.99	-0.263	0.081	0.001183	0.0002362		0.074	0.99	-0.00094	0.03551	0.9787921
rs1532724	12	129220735	A	G	0.937	0.99	0.263	0.081	0.001182	0.0002351		0.926	0.98	0.000935	0.03555	0.9790084
rs4759624	12	129224078	C	T	0.063	0.97	-0.269	0.082	0.001028	0.0001881		0.074	0.91	-0.00021	0.03703	0.995416
rs6598178	12	130600181	A	G	0.415	0.94	0.125	0.041	0.002058	0.0003326		0.358	0.93	-0.03933	0.01939	0.0423623
rs6598218	12	130605166	C	T	0.512	0.93	0.111	0.04	0.00519	0.0005466		0.444	0.91	-0.02254	0.01906	0.2365244
rs7398018	12	130605295	C	T	0.492	0.97	-0.111	0.039	0.004258	0.0007071		0.573	0.98	0.025734	0.01849	0.163586
rs4133254	12	130608911	A	G	0.591	0.98	-0.132	0.04	0.0009633	0.0001671		0.648	0.96	0.036992	0.01918	0.053559
rs7398258	12	130611635	C	T	0.312	0.76	0.18	0.048	0.0001877	0.0000113		0.270	0.70	-0.04969	0.02414	0.0394166
rs7488571	12	130611957	C	T	0.591	0.98	-0.132	0.04	0.0009317	0.0001627		0.648	0.96	0.036742	0.01917	0.0551508
rs11246799	12	130617425	A	G	0.548	0.97	0.127	0.039	0.00124	0.0001408		0.461	0.96	-0.01311	0.01856	0.4796298

					GOYA QC		GOYA Overweight/control			GOYA BMI continuous	known	IARC QC		IARC results		
MARKER	chr	position	A1	A2	FREQ1	Rsqr	Beta	SE	p	p	gene name	freq1	Rsqr	in_beta	in_SE	in_p
rs11614599	12	130620734	A	C	0.795	0.85	-0.172	0.052	0.0008684	0.0004339		0.824	0.77	-0.01156	0.02728	0.6715589
rs11246872	12	130634043	A	G	0.390	1.00	0.122	0.04	0.002044	0.0002591		0.348	1.00	-0.0247	0.0188	0.1884249
rs6421998	12	130638019	A	G	0.390	1.00	0.123	0.04	0.002033	0.0002566		0.348	1.00	-0.02474	0.01881	0.1880511
rs7398669	12	130640809	C	T	0.610	0.99	-0.125	0.04	0.001828	0.0002256		0.652	0.99	0.024895	0.01886	0.1865545
rs7302004	12	130641523	C	G	0.390	0.98	0.125	0.04	0.001777	0.0002186		0.348	0.98	-0.02518	0.01895	0.1837352
rs7294672	12	130667902	C	T	0.841	0.94	0.195	0.054	0.000291	0.0035081		0.825	0.94	0.015481	0.02527	0.5398176
rs7134297	12	130750267	C	T	0.982	0.86	-0.62	0.164	0.0001597	0.0006722		0.983	0.59	-0.00411	0.09254	0.9645761
rs4964984	12	130756911	G	T	0.888	0.96	-0.225	0.063	0.0003424	0.0014012		0.874	0.91	-0.03337	0.02917	0.2521576
rs4964985	12	130768615	A	G	0.889	1.00	-0.224	0.062	0.0002977	0.0011864		0.877	0.99	-0.03142	0.02833	0.2669486
rs4964986	12	130773122	A	C	0.111	1.00	0.224	0.062	0.0002933	0.0011757		0.123	0.99	0.031597	0.02832	0.264153
rs10902438	12	130779526	C	T	0.889	1.00	-0.225	0.062	0.0002774	0.0011547		0.875	0.99	-0.03207	0.02813	0.254016
rs12370005	12	130783940	A	G	0.111	1.00	0.223	0.062	0.0003223	0.0012503		0.124	0.98	0.031193	0.0283	0.2700309
rs7980865	12	130785380	A	G	0.941	0.99	-0.345	0.083	0.0000327	0.0000541		0.927	0.97	-0.01657	0.03514	0.6368625
rs10902439	12	130785760	G	T	0.111	1.00	0.223	0.062	0.0003263	0.0012605		0.124	0.98	0.031117	0.0283	0.2711183
rs10902440	12	130786300	C	T	0.111	1.00	0.222	0.062	0.0003284	0.0012653		0.124	0.98	0.031083	0.0283	0.2716115
rs737434	12	130787012	C	T	0.890	1.00	-0.222	0.062	0.0003305	0.0012709		0.876	0.98	-0.03324	0.02825	0.2389361
rs12312632	12	130789833	C	G	0.111	1.00	0.223	0.062	0.0003267	0.0012597		0.124	0.98	0.03099	0.02829	0.2729343
rs12314207	12	130790233	C	G	0.107	0.98	0.228	0.064	0.0003475	0.0009473		0.119	0.96	0.03206	0.02923	0.2723008
rs11246796	12	130799560	A	C	0.889	0.99	-0.225	0.062	0.0002844	0.0011367		0.875	0.98	-0.03067	0.02826	0.2774069
rs4964926	12	130882177	C	G	0.915	0.85	-0.311	0.076	0.0000397	0.00000663		0.901	0.76	-0.00611	0.03419	0.8580656
rs7134349	12	130883782	C	G	0.086	0.85	0.311	0.076	0.0000398	0.00000663		0.099	0.76	0.006159	0.03419	0.8569153
rs11246838	12	130883944	A	G	0.914	0.84	-0.311	0.076	0.0000402	0.00000668		0.901	0.76	-0.00616	0.03419	0.8567849
rs11613757	12	130898190	C	T	0.925	0.71	-0.326	0.088	0.0001965	0.0000415		0.935	0.61	-0.00286	0.04421	0.9484052
rs9509962	13	21467496	A	G	0.025	0.84	-0.406	0.138	0.003188	0.0007729		0.027	0.26	-0.06618	0.10608	0.5323641
rs9509964	13	21479435	C	T	0.025	0.83	-0.416	0.14	0.003048	0.0007165		0.025	0.26	-0.03914	0.10977	0.7211862
rs9506854	13	21925350	C	T	0.700	0.37	-0.188	0.07	0.007288	0.0002812		0.715	0.33	-0.04004	0.03535	0.2569363
rs9581389	13	25023731	C	T	0.616	0.89	-0.145	0.042	0.0005803	0.0046041		0.663	0.87	-0.01337	0.02056	0.5152761
rs7320319	13	33052360	A	G	0.049	0.99	-0.265	0.091	0.00368	0.0006256		0.044	0.98	0.004015	0.04276	0.9251357
rs9597686	13	33054521	C	T	0.049	0.99	-0.266	0.091	0.003522	0.0005907		0.044	0.98	0.003736	0.04263	0.930096
rs9569841	13	33063186	C	T	0.951	0.99	0.267	0.091	0.003267	0.0005289		0.956	0.99	-0.00354	0.04249	0.9334695
rs9591868	13	33065004	C	G	0.951	0.99	0.267	0.091	0.003311	0.00054		0.956	0.99	-0.00343	0.0425	0.935595
rs9597714	13	33073761	C	T	0.050	0.97	-0.264	0.091	0.003524	0.000623		0.045	0.86	0.009449	0.04518	0.8341883
rs9572720	13	34371956	A	G	0.036	0.99	0.354	0.105	0.0007443	0.0009967		0.035	0.98	-0.11298	0.05062	0.0255098
rs7332133	13	34471743	A	T	0.933	0.47	0.384	0.114	0.0007729	0.000714		0.937	0.33	-0.00339	0.0657	0.9587567
rs9544180	13	34851882	A	G	0.956	0.99	0.319	0.097	0.0009748	0.0026474		0.960	0.98	-0.0267	0.04621	0.5630032
rs1461968	13	34868655	C	T	0.956	0.99	0.319	0.097	0.0009812	0.0027393		0.960	0.98	-0.02624	0.04622	0.5698587
rs7399673	13	36199640	C	T	0.211	1.00	0.16	0.048	0.0008093	0.0017852		0.270	0.74	-0.0113	0.02376	0.6339741
rs12865228	13	38238418	G	T	0.155	1.00	0.16	0.054	0.002905	0.00054		0.195	0.69	-0.0286	0.02816	0.3093568
rs12584459	13	38247467	G	T	0.163	0.62	0.194	0.066	0.003547	0.0005269		0.200	0.51	-0.04512	0.03272	0.1675643
rs9566497	13	39671110	C	T	0.110	1.00	-0.22	0.062	0.0003831	0.0000446		0.101	0.98	-0.07475	0.03011	0.0129974
rs9566498	13	39671652	C	T	0.110	1.00	-0.22	0.062	0.0003828	0.0000445		0.101	0.98	-0.07473	0.03012	0.0130455
rs12161861	13	39692113	C	T	0.107	0.97	-0.218	0.064	0.0006079	0.0001358		0.098	0.96	-0.05929	0.03066	0.0529832
rs9549137	13	39721228	A	G	0.879	0.90	0.183	0.062	0.003268	0.0006785		0.885	0.88	0.045182	0.02968	0.1276921
rs4943747	13	39722245	C	T	0.121	0.90	-0.183	0.062	0.003248	0.0006683		0.115	0.88	-0.04247	0.02969	0.1523693
rs9576966	13	39726990	C	T	0.885	0.95	0.168	0.062	0.00678	0.0009238		0.891	0.92	0.032018	0.02977	0.2817187
rs9525397	13	40323711	C	G	0.697	1.00	-0.15	0.042	0.000402	0.0011464		0.662	0.99	-0.04193	0.01963	0.0325506
rs7333274	13	40334667	C	T	0.682	0.98	-0.139	0.042	0.0009236	0.0026653		0.642	0.98	-0.0515	0.0195	0.0082412
rs4254182	13	40351256	C	T	0.687	0.96	-0.15	0.043	0.000479	0.0007382		0.644	0.93	-0.04443	0.0201	0.0269444
rs7320344	13	40357638	A	G	0.303	1.00	0.15	0.042	0.0004053	0.0010848		0.339	0.98	0.041931	0.01968	0.0330131
rs1989252	13	40383668	C	T	0.306	1.00	0.152	0.042	0.0003082	0.001084		0.339	0.98	0.04187	0.0197	0.0334341
rs7333447	13	40389722	A	T	0.306	1.00	0.152	0.042	0.0003125	0.0010924		0.339	0.98	0.041842	0.01971	0.033614
rs9532670	13	40399827	C	T	0.692	0.99	-0.139	0.042	0.0009916	0.0025042		0.646	0.98	-0.05112	0.01962	0.0091281
rs3736953	13	40423586	A	G	0.303	0.98	0.159	0.043	0.0001939	0.000857		0.356	0.97	0.050671	0.01974	0.0102279
rs9532689	13	40430126	C	T	0.697	1.00	-0.161	0.042	0.0001472	0.0008304		0.641	1.00	-0.04771	0.01949	0.0142884
rs7799	13	40431052	C	T	0.697	1.00	-0.161	0.042	0.0001456	0.0008351		0.641	1.00	-0.04755	0.01946	0.0145074
rs10467369	13	40433038	A	G	0.303	1.00	0.16	0.042	0.0001514	0.0008517		0.359	1.00	0.047517	0.01947	0.0145902
rs7329302	13	40434532	A	G	0.697	1.00	-0.16	0.042	0.000152	0.0008536		0.641	1.00	-0.0475	0.01947	0.0146315
rs9525438	13	40436259	A	G	0.303	1.00	0.16	0.042	0.0001526	0.0008552		0.359	1.00	0.047479	0.01947	0.0146976
rs4942014	13	40438502	A	T	0.303	1.00	0.16	0.042	0.0001532	0.000857		0.359	1.00	0.047464	0.01947	0.0147388
rs9532690	13	40440790	C	T	0.697	1.00	-0.16	0.042	0.0001545	0.0008609		0.641	1.00	-0.04746	0.01948	0.0147595
rs9532691	13	40441494	C	T	0.697	1.00	-0.16	0.042	0.0001552	0.0008628		0.641	1.00	-0.04745	0.01948	0.0147841
rs2324748	13	40461588	C	T	0.697	1.00	-0.159	0.042	0.0001729	0.0009165		0.642	1.00	-0.04659	0.0195	0.0168367
rs9525439	13	40464781	A	G	0.697	1.00	-0.159	0.042	0.0001751	0.000923		0.642	1.00	-0.04644	0.0195	0.0171666
rs1543589	13	40471200	C	T	0.307	0.95	0.162	0.043	0.0001757	0.0009384		0.346	0.96	0.044114	0.02012	0.0282606
rs7489810	13	40885767	A	G	0.171	1.00	-0.173	0.051	0.0007445	0.007627		0.136	0.99	-0.00903	0.02616	0.7298679
rs4427698	13	40886053	A	C	0.171	1.00	-0.173	0.051	0.0007505	0.0077425		0.136	0.99	-0.00958	0.02611	0.7134524
rs17069282	13	46472727	C	T	0.183	0.99	-0.168	0.051	0.0009409	0.0059067		0.184	0.98	-0.00624	0.024	0.7947577
rs17310690	13	46475522	C	T	0.828	1.00	0.188	0.052	0.0002712	0.0022094		0.829	0.98	-0.00206	0.02482	0.933648
rs11619704	13	46496307	C	T	0.790	0.99	0.156	0.047	0.0008667	0.0025098		0.781	0.99	0.021353	0.02252	0.3427337
rs2082292	13	46498908	C	G	0.211	0.99	-0.157	0.047	0.0008577	0.0024809		0.219	0.98	-0.02193	0.02261	0.3315962
rs9569733	13	57214613	C	T	0.168	0.99	-0.185	0.052	0.0003617	0.0006449		0.170	0.98	-0.0174	0.02444	0.4761757
rs9569738	13	57228644	A	G	0.211	1.00	-0.156	0.047	0.0009905	0.0019983		0.219	1.00	-0.02035	0.02227	0.3603624
rs1537445	13	58289913	A	T	0.078	1.00	0.222	0.072	0.002109	0.0004266		0.079	0.99	0.059028	0.03259	0.069922
rs722122	13	58297035	A	C	0.784	1.00	-0.149	0.048	0.001797	0.0006605		0.795	1.00	-0.04485	0.02229	0.0440652
rs9569993	13	58311680	A	G	0.086	1.00	0.235	0.069	0.0006896	0.0001702		0.090	1.00	0.04399	0.03118	0.1579963
rs9538228	13	58314120	A	G	0.086	1.00	0.234	0.069	0.0007162	0.0001733		0.090	1.00	0.043668	0.03118	0.1610979
rs7326173	13	58314409	A	G	0.212	1.00	0.151	0.048	0.001684	0.0005113		0.204	1.00	0.046414	0.02235	0.0377227
rs7983390	13	58319712	A	T	0.789	1.00	-0.151	0.048	0.001615	0.0004904		0.797	1.00	-0.04644	0.02237	0.0377287
rs6561989	13	58322537	A	C	0.789	1.00	-0.152	0.048	0.001575	0.0004782		0.797	1.00	-0.04646	0.02238	0.0377556
rs1333423	13	58323112	A	T	0.211	1.00	0.152	0.048	0.00157	0.0004768		0.203	1.00	0.046464	0.02238	0.0377396
rs7328849	13	58324900	A	G	0.793	0.97	-0.159	0.049	0.001218	0.0003595		0.800	0.98	-0.04733	0.02273	0.0371692
rs1537449	13	58327474	A	C	0.789	1.00	-0.152	0.048	0.001499	0.0004555		0.797	1.00	-0.04648	0.0224	0.0378149

					GOYA QC		GOYA Overweight/control			GOYA BMI continuous	known	IARC QC		IARC results		
MARKER	chr	position	A1	A2	FREQ1	Rsqr	Beta	SE	p	p	gene name	freq1	Rsqr	in_beta	in_SE	in_p
rs2321886	13	58349584	A	G	0.202	0.95	0.151	0.05	0.002363	0.0009309		0.195	0.89	0.046166	0.02402	0.054436
rs2321884	13	58349733	C	T	0.806	0.99	-0.163	0.05	0.001008	0.0002178		0.819	0.99	-0.05279	0.02348	0.0244823
rs2321882	13	58349990	C	G	0.194	0.99	0.163	0.05	0.001008	0.0002176		0.181	0.99	0.052785	0.02348	0.0244929
rs17608913	13	58351600	C	T	0.806	0.97	-0.165	0.05	0.0009277	0.0003357		0.811	0.94	-0.04443	0.02368	0.0604276
rs9652215	13	58775042	A	G	0.931	0.71	0.319	0.091	0.0004353	0.0009477		0.924	0.62	-0.02187	0.0452	0.6282803
rs9598123	13	59825099	C	T	0.819	0.88	-0.187	0.054	0.0005117	0.0003258		0.832	0.88	0.006884	0.02552	0.7871875
rs9317115	13	59825897	A	G	0.181	0.88	0.187	0.054	0.0005074	0.0003203		0.168	0.88	-0.00687	0.02551	0.7875473
rs9598426	13	61638248	A	G	0.045	0.51	0.446	0.131	0.0006693	0.0040422		0.035	0.39	0.027317	0.07332	0.7092163
rs868349	13	70016927	A	C	0.972	0.73	0.502	0.141	0.0003712	0.0015922		0.981	0.61	0.014934	0.09485	0.8747777
rs9542937	13	71876870	C	T	0.599	0.69	0.156	0.047	0.0008857	0.0014514		0.618	0.65	-0.01942	0.02319	0.4018347
rs12431220	13	75365526	A	G	0.172	0.98	0.171	0.052	0.0009223	0.0040871		0.151	0.99	0.0178	0.02511	0.4779951
rs2760105	13	79007868	A	G	0.600	0.96	0.145	0.04	0.0003066	0.0005879		0.588	0.96	-0.00064	0.01904	0.97316
rs9515626	13	89230620	A	G	0.464	0.97	-0.132	0.04	0.0008515	0.0016356		0.437	0.88	-0.03013	0.01962	0.1244678
rs17550263	13	89231055	C	T	0.546	0.97	0.133	0.04	0.000818	0.0027243		0.571	0.91	0.027783	0.01938	0.1512856
rs4773517	13	89231603	A	T	0.546	0.97	0.133	0.04	0.00082	0.0027352		0.571	0.91	0.027774	0.01936	0.1510614
rs4773520	13	89231710	A	G	0.454	0.97	-0.133	0.04	0.0008252	0.0027615		0.429	0.94	-0.02781	0.01912	0.1455718
rs12585404	13	93110396	A	C	0.021	0.99	0.519	0.141	0.0002384	0.0001627		0.040	0.97	0.00035	0.04771	0.9941404
rs9561428	13	93139097	A	G	0.021	0.99	0.53	0.141	0.0001676	0.0001158		0.040	0.97	0.001219	0.04764	0.9795644
rs9561432	13	93174799	G	T	0.980	0.99	-0.539	0.142	0.0001424	0.0000969		0.960	0.97	-0.00392	0.04784	0.9346868
rs9301901	13	93180551	A	G	0.980	0.99	-0.54	0.142	0.0001409	0.000096		0.960	0.97	-0.0043	0.04787	0.9284267
rs12584151	13	93189989	A	C	0.022	1.00	0.441	0.135	0.001076	0.000322		0.041	0.93	0.01558	0.04753	0.7428368
rs9561435	13	93194515	C	G	0.980	1.00	-0.539	0.142	0.0001387	0.0000949		0.960	0.96	-0.00558	0.04798	0.9073374
rs9561436	13	93199085	C	T	0.978	1.00	-0.441	0.135	0.001095	0.0003405		0.959	0.93	-0.01597	0.04753	0.7367228
rs4612931	13	93222197	G	T	0.027	1.00	0.407	0.121	0.0007851	0.0033282		0.041	0.96	0.022199	0.04615	0.6301872
rs9561441	13	93224307	A	G	0.027	1.00	0.408	0.121	0.000788	0.0033491		0.041	0.96	0.02227	0.04629	0.6301709
rs9556323	13	93224463	A	G	0.027	1.00	0.408	0.122	0.0007926	0.0033774		0.041	0.96	0.022248	0.04631	0.6306495
rs9561465	13	93356113	G	T	0.028	0.90	0.424	0.127	0.000831	0.0044802		0.035	0.81	0.022298	0.05288	0.673025
rs17189376	13	94622104	C	T	0.246	1.00	-0.165	0.045	0.0002543	0.0026429		0.233	0.70	-0.02783	0.02553	0.2753094
rs7319175	13	94819184	A	C	0.013	0.72	-0.646	0.212	0.002347	0.0007903		0.013	0.71	0.023054	0.1053	0.8265528
rs6491307	13	96252811	A	G	0.201	1.00	-0.16	0.048	0.000957	0.0120953		0.164	1.00	0.003459	0.02439	0.8871418
rs7326447	13	98206541	C	T	0.971	0.83	0.436	0.13	0.0008094	0.0033184		0.972	0.58	0.022111	0.07202	0.7586479
rs7324019	13	104888861	A	C	0.904	0.97	-0.209	0.067	0.001919	0.0007721		0.918	0.51	-0.02199	0.04621	0.6339293
rs16972216	13	107732621	A	G	0.129	0.58	-0.253	0.077	0.001005	0.000862		0.147	0.51	-0.00627	0.03663	0.8640558
rs9559537	13	108736856	A	C	0.095	0.98	-0.223	0.068	0.00096	0.0014879		0.077	0.89	0.096535	0.03466	0.0053274
rs2149202	13	108830666	A	G	0.040	0.99	0.33	0.1	0.0009501	0.0161052		0.033	0.37	0.134429	0.0801	0.0930523
rs9559982	13	110548443	A	T	0.796	0.88	0.168	0.052	0.001318	0.0000945		0.838	0.85	0.039444	0.02547	0.1212066
rs831159	13	110580878	A	T	0.702	0.86	0.156	0.045	0.0005689	0.0002143		0.746	0.82	0.011248	0.02225	0.6128594
rs9577345	13	112059457	A	T	0.896	0.86	0.248	0.069	0.0003518	0.006714		0.911	0.69	-0.08382	0.03766	0.0259471
rs9603986	13	112604358	C	T	0.988	0.34	-1.267	0.334	0.0001491	0.0008057		0.974	0.38	-0.0663	0.10271	0.5182337
rs9603799	13	112604585	A	G	0.012	0.34	1.268	0.334	0.0001488	0.000801		0.026	0.38	0.066443	0.10301	0.5185617
rs10047878	14	21857530	A	T	0.539	0.99	-0.186	0.039	0.00000256	0.0000475		0.533	0.95	-0.01102	0.01856	0.5524215
rs8007205	14	21859312	A	G	0.458	0.96	-0.165	0.04	0.0000381	0.0013075		0.460	0.89	-0.01489	0.01933	0.4407773
rs916049	14	21860911	C	G	0.531	0.98	-0.175	0.04	0.00000964	0.0001016		0.533	0.92	-0.00457	0.01874	0.8071851
rs2204990	14	21861403	A	G	0.542	1.00	-0.182	0.039	0.00000353	0.0000587		0.561	1.00	-0.00806	0.01823	0.6580279
rs11847479	14	21862055	C	T	0.478	0.98	-0.162	0.039	0.000038	0.000938		0.498	0.88	-0.01261	0.01935	0.5142157
rs17116418	14	21862480	C	T	0.596	0.95	0.151	0.041	0.0002402	0.0001607		0.633	0.93	-0.00913	0.01993	0.6466937
rs10139769	14	21863305	A	G	0.293	0.93	0.179	0.044	0.0000463	0.0002273		0.285	0.93	-0.00607	0.02081	0.7702317
rs1107390	14	21865110	C	T	0.107	0.99	-0.226	0.064	0.0003897	0.0000618		0.077	0.97	-0.02518	0.03563	0.4793754
rs12587680	14	21869348	A	G	0.893	1.00	0.227	0.064	0.0003638	0.0000583		0.924	0.99	0.023815	0.03541	0.5008698
rs2213246	14	21873203	C	T	0.713	1.00	-0.163	0.043	0.0001298	0.0002255		0.719	1.00	0.006117	0.02025	0.7623873
rs2213247	14	21873224	A	G	0.287	1.00	0.163	0.043	0.0001301	0.0002257		0.281	1.00	-0.00614	0.02025	0.761676
rs17183505	14	21876908	C	G	0.107	1.00	-0.228	0.064	0.0003291	0.0000551		0.076	0.99	-0.02315	0.03543	0.5131965
rs4981423	14	21878594	A	G	0.287	1.00	0.163	0.043	0.0001323	0.0002274		0.282	1.00	-0.00622	0.02023	0.7582524
rs8014927	14	21908470	A	G	0.714	1.00	-0.165	0.043	0.0001162	0.0002212		0.718	1.00	0.007671	0.02025	0.7046115
rs2051536	14	21911637	C	T	0.714	1.00	-0.164	0.043	0.0001185	0.0002273		0.718	1.00	0.007577	0.02027	0.7082571
rs2877454	14	21919601	A	C	0.287	0.99	0.163	0.043	0.0001513	0.0002857		0.279	0.98	-0.00593	0.02046	0.7719368
rs17183519	14	21921082	A	C	0.106	0.99	-0.223	0.064	0.0005058	0.0000955		0.075	0.99	-0.02604	0.03567	0.4650563
rs741711	14	21923525	C	T	0.896	1.00	0.215	0.064	0.0008079	0.0001629		0.927	1.00	0.028135	0.03568	0.4299816
rs1076548	14	21924406	C	T	0.861	0.84	0.242	0.062	0.0000921	0.0000215		0.902	0.77	0.029107	0.03558	0.4129627
rs3811250	14	21925810	A	G	0.404	1.00	-0.145	0.04	0.0002991	0.0001217		0.362	1.00	0.007285	0.01932	0.7059194
rs12434373	14	21926789	C	T	0.363	0.99	0.148	0.041	0.0002784	0.0021916		0.355	1.00	-0.01426	0.0192	0.4571103
rs4982624	14	21927364	C	T	0.715	0.99	-0.157	0.043	0.0002607	0.0006916		0.721	0.99	0.004802	0.02042	0.8139424
rs3811244	14	21929322	G	T	0.363	1.00	0.145	0.041	0.0003574	0.0028679		0.355	1.00	-0.01441	0.01917	0.4519131
rs4982626	14	21929899	A	T	0.637	1.00	-0.145	0.041	0.0003525	0.0028454		0.645	1.00	0.014549	0.01917	0.4475902
rs12896137	14	21931452	C	T	0.637	1.00	-0.146	0.041	0.0003323	0.0027546		0.645	1.00	0.014864	0.01918	0.4380981
rs4981424	14	21931569	C	T	0.637	1.00	-0.146	0.041	0.0003282	0.002737		0.645	1.00	0.014966	0.01919	0.4350488
rs4982628	14	21931730	C	T	0.363	1.00	0.146	0.041	0.0003284	0.0027407		0.355	1.00	-0.015	0.01919	0.4340729
rs6572425	14	21932036	G	T	0.595	1.00	0.144	0.04	0.0003401	0.000117		0.638	1.00	-0.01119	0.01937	0.5632378
rs9919897	14	21932716	C	T	0.716	0.99	-0.157	0.043	0.0002632	0.0007607		0.721	0.99	0.005202	0.02042	0.7988081
rs9919922	14	21932964	A	G	0.637	1.00	-0.146	0.041	0.0003292	0.0027559		0.645	1.00	0.015343	0.0192	0.4238223
rs8011618	14	21934153	A	G	0.637	1.00	-0.146	0.041	0.0003294	0.0027593		0.646	0.99	0.01587	0.01921	0.408392
rs1040303	14	21941618	A	G	0.109	1.00	-0.204	0.063	0.001147	0.0003789		0.078	1.00	-0.02219	0.03555	0.5320619
rs10149541	14	21943149	C	T	0.718	0.98	-0.155	0.043	0.0003275	0.0009801		0.722	0.98	0.006721	0.02051	0.7429716
rs714795	14	21946458	C	T	0.891	1.00	0.206	0.063	0.00103	0.0003319		0.923	1.00	0.022204	0.03556	0.5319847
rs2014778	14	21946656	G	T	0.282	0.98	0.155	0.043	0.0003526	0.0010573		0.278	0.98	-0.00683	0.02052	0.7391751
rs760019	14	21953018	A	G	0.492	1.00	-0.127	0.039	0.001075	0.0001433		0.449	1.00	0.003472	0.01848	0.8508554
rs11846244	14	21965715	A	G	0.109	0.99	-0.234	0.063	0.0002257	0.0000508		0.076	0.96	-0.02493	0.03657	0.4949568
rs17183603	14	21969987	C	G	0.109	0.99	-0.234	0.063	0.0002249	0.0000507		0.076	0.96	-0.02486	0.03656	0.4961592
rs11625456	14	21993981	A	C	0.946	0.99	0.256	0.087	0.003383	0.0002054		0.955	0.98	0.023859	0.04639	0.6067404
rs7157021	14	22668792	A	G	0.457	1.00	0.127	0.039	0.001021	0.0009428		0.453	1.00	-0.00701	0.01836	0.7023158
rs1958365	14	31740992	A	G	0.625	0.98	-0.153	0.04	0.0001438	0.0025182		0.635	0.94	0.000231	0.0195	0.9905301
rs1958364	14	31741921	C	T	0.372	0.98	0.155	0.04	0.0001217	0.0019848		0.355	0.95	0.00398	0.01961	0.8390388

					GOYA QC		GOYA Overweight/control			GOYA BMI continuous	known	IARC QC		IARC results		
MARKER	chr	position	A1	A2	FREQ1	Rsqr	Beta	SE	p	p	gene name	freq1	Rsqr	in_beta	in_SE	in_p
rs17388327	14	31745754	A	C	0.646	1.00	-0.156	0.04	0.0001132	0.0021572		0.667	0.92	0.000796	0.02024	0.9686112
rs7155347	14	31767309	C	T	0.358	1.00	0.16	0.04	0.0000702	0.0018696		0.347	0.97	0.012365	0.01938	0.5231403
rs1951680	14	31769148	C	T	0.382	1.00	0.146	0.04	0.0002401	0.0041089		0.375	0.96	0.016025	0.01911	0.401359
rs10145223	14	31771218	C	T	0.619	1.00	-0.146	0.04	0.0002334	0.0040357		0.626	0.96	-0.0158	0.01913	0.4084729
rs8011923	14	31771615	C	T	0.619	1.00	-0.146	0.04	0.0002367	0.0040858		0.626	0.96	-0.0158	0.01913	0.4085004
rs7144586	14	31783043	A	G	0.349	1.00	0.166	0.04	0.0000411	0.0008503		0.340	1.00	0.011706	0.01929	0.5435373
rs1007279	14	31784577	A	T	0.350	1.00	0.166	0.04	0.0000401	0.0008461		0.340	0.99	0.011566	0.01931	0.5487946
rs7161546	14	31789254	C	T	0.858	0.62	-0.25	0.07	0.0003317	0.002557		0.905	0.37	-0.03275	0.0505	0.5162682
rs7160483	14	31789427	A	C	0.351	0.99	0.167	0.04	0.0000368	0.0008359		0.341	0.99	0.011517	0.01931	0.550612
rs7161530	14	31791118	C	T	0.375	0.99	0.153	0.04	0.0001303	0.0019999		0.369	0.97	0.014866	0.01911	0.4361843
rs4981953	14	31791941	C	G	0.647	0.96	-0.175	0.041	0.0000191	0.0004434		0.659	0.99	-0.01176	0.01936	0.5430479
rs6571507	14	31842078	A	G	0.373	0.99	0.179	0.04	0.00000767	0.0000771		0.363	0.98	0.013115	0.01912	0.4924618
rs10872860	14	31842726	C	T	0.373	0.99	0.179	0.04	0.00000784	0.0000795		0.363	0.98	0.013119	0.01912	0.492347
rs2208869	14	31845602	C	T	0.374	1.00	0.177	0.04	0.0000101	0.0001159		0.361	0.98	0.01331	0.01913	0.4861977
rs2383310	14	31845887	C	T	0.626	1.00	-0.176	0.04	0.0000102	0.0001179		0.639	0.98	-0.0133	0.01913	0.4865269
rs8005191	14	31846053	G	T	0.637	0.96	-0.183	0.041	0.00000795	0.0001483		0.657	0.92	-0.01542	0.01995	0.4390939
rs9672094	14	31849098	A	G	0.496	0.89	-0.135	0.041	0.0009504	0.0029312		0.516	0.83	-0.01351	0.01995	0.4978358
rs12884331	14	34908427	C	T	0.965	0.96	0.386	0.109	0.0004154	0.0007473		0.962	0.68	0.005404	0.05433	0.9206941
rs8007950	14	34913536	A	T	0.965	0.96	0.385	0.109	0.0004362	0.0007899		0.962	0.68	0.005098	0.05431	0.9251499
rs8008408	14	34913567	A	G	0.131	0.96	-0.245	0.059	0.0000366	0.0005564		0.121	0.77	0.016038	0.03149	0.6102384
rs12892002	14	34919935	A	G	0.035	0.94	-0.381	0.11	0.0005308	0.0009898		0.038	0.68	-0.00493	0.0543	0.9275963
rs10140059	14	39049504	A	G	0.334	0.99	0.151	0.041	0.0002363	0.0097569		0.357	1.00	-0.01638	0.01894	0.3867962
rs2038279	14	39052178	A	T	0.262	0.99	0.148	0.044	0.0008615	0.0403492		0.280	1.00	-0.00078	0.02013	0.9690897
rs1950339	14	39053580	G	T	0.258	1.00	0.148	0.045	0.0008809	0.0571641		0.280	1.00	-0.0006	0.02011	0.9760534
rs11624961	14	39068209	C	T	0.257	1.00	0.148	0.044	0.0008559	0.0562387		0.280	1.00	-0.00061	0.0201	0.9756876
rs11623451	14	39075725	C	T	0.743	1.00	-0.148	0.045	0.0008688	0.0565923		0.721	0.99	-0.00134	0.02021	0.9470156
rs8017099	14	39076410	C	T	0.683	1.00	-0.144	0.042	0.0005778	0.034854		0.653	0.99	0.016752	0.0191	0.3801605
rs1955705	14	39082203	C	T	0.241	0.99	0.155	0.046	0.0006567	0.0327612		0.265	0.99	0.004161	0.02047	0.8387436
rs4902768	14	39086443	C	T	0.586	1.00	-0.155	0.039	0.0000863	0.006289		0.577	1.00	0.01624	0.01827	0.3737125
rs10134241	14	39092714	A	G	0.473	1.00	0.145	0.039	0.0001964	0.0053878		0.481	1.00	-0.00799	0.01813	0.6592574
rs10483536	14	39096649	A	G	0.473	1.00	0.147	0.039	0.0001685	0.0049228		0.480	1.00	-0.00764	0.01813	0.6731271
rs4902773	14	39101739	A	G	0.527	1.00	-0.146	0.039	0.0001796	0.0052152		0.520	1.00	0.007564	0.01813	0.6762429
rs1955711	14	39102940	C	T	0.473	1.00	0.145	0.039	0.0001904	0.0054928		0.481	1.00	-0.00748	0.01813	0.6795632
rs1014896	14	39106984	C	T	0.586	1.00	-0.157	0.039	0.0000658	0.0054855		0.575	1.00	0.015266	0.01827	0.4030624
rs12881979	14	39107789	C	T	0.473	1.00	0.146	0.039	0.0001767	0.0050488		0.481	1.00	-0.00754	0.01813	0.6771551
rs175677	14	39119446	A	G	0.527	1.00	-0.146	0.039	0.000174	0.0049403		0.519	1.00	0.00757	0.01815	0.6763865
rs175678	14	39122270	A	G	0.414	1.00	0.158	0.039	0.0000608	0.0050275		0.424	0.98	-0.01595	0.0184	0.385707
rs175686	14	39130439	C	G	0.423	0.97	0.156	0.04	0.0000845	0.0063569		0.433	0.96	-0.0133	0.01861	0.4744401
rs6571945	14	39136999	A	G	0.586	1.00	-0.158	0.039	0.0000598	0.0049145		0.576	0.98	0.016108	0.01842	0.3815783
rs11626685	14	39321630	C	G	0.942	0.63	-0.368	0.105	0.0004478	0.021058		0.946	0.50	0.052909	0.05816	0.3625908
rs1393835	14	41787701	A	G	0.884	0.93	0.226	0.063	0.0003508	0.0011145		0.852	0.90	-0.00288	0.02721	0.915661
rs1504610	14	56544718	C	T	0.734	1.00	0.151	0.044	0.0005678	0.0012435		0.749	1.00	-0.00507	0.02149	0.8132797
rs8008798	14	61017755	C	T	0.164	0.99	-0.175	0.052	0.0008327	0.0022344		0.159	0.94	0.03561	0.02563	0.1644546
rs4902067	14	61026711	C	G	0.168	1.00	-0.177	0.051	0.0005937	0.0027765		0.159	0.93	0.037665	0.0257	0.1424325
rs10136208	14	61027797	C	T	0.162	0.95	-0.179	0.053	0.00079	0.0050726		0.145	0.89	0.031915	0.02706	0.237825
rs17751556	14	63953859	C	T	0.088	0.75	0.282	0.078	0.0002984	0.0002428		0.077	0.63	0.007095	0.04165	0.8646251
rs9671808	14	64111236	A	G	0.077	0.93	0.268	0.075	0.0003686	0.0005134		0.062	0.89	7.95E-05	0.03867	0.9983575
rs10151495	14	64213981	A	C	0.927	0.95	-0.242	0.076	0.001551	0.000795		0.935	0.93	-0.01203	0.03771	0.7495507
rs4902306	14	64218952	A	G	0.079	1.00	0.244	0.072	0.0007079	0.0005889		0.064	0.87	0.022128	0.03852	0.5653772
rs11158557	14	64222939	A	G	0.919	1.00	-0.238	0.071	0.0008426	0.0008394		0.933	0.90	-0.0287	0.03724	0.4405125
rs229676	14	64225023	C	G	0.657	1.00	0.113	0.041	0.005775	0.000659		0.598	0.99	-0.02328	0.01854	0.2090894
rs229605	14	64229799	C	T	0.343	1.00	-0.113	0.041	0.005678	0.0006567		0.403	0.99	0.023723	0.01851	0.1996176
rs179336	14	64230337	C	T	0.741	0.99	0.148	0.044	0.0008404	0.0004049		0.724	0.97	-0.01981	0.02058	0.3353493
rs170679	14	64299117	A	G	0.075	0.88	0.261	0.079	0.0009138	0.0010509		0.060	0.37	0.067776	0.06222	0.2756121
rs10132579	14	68044030	A	G	0.855	0.91	-0.166	0.058	0.004031	0.0005222		0.837	0.90	-0.01325	0.02575	0.6066216
rs8015246	14	68045144	C	T	0.823	0.98	-0.151	0.051	0.003301	0.0002444		0.802	0.96	-0.00424	0.0234	0.8559968
rs10131789	14	68047253	C	T	0.180	0.99	0.151	0.051	0.002934	0.0002259		0.201	0.99	0.005273	0.02294	0.8180348
rs1541390	14	68049682	A	G	0.820	1.00	-0.151	0.051	0.002921	0.0002267		0.799	0.99	-0.00526	0.02291	0.8182114
rs10135398	14	68055359	A	G	0.820	0.99	-0.152	0.051	0.002858	0.0002204		0.799	0.99	-0.00526	0.02295	0.8185554
rs1023530	14	68059761	A	G	0.805	0.97	-0.13	0.05	0.008606	0.0007227		0.767	0.96	-0.00288	0.02224	0.897011
rs12882030	14	68075036	A	G	0.204	1.00	0.132	0.048	0.005791	0.0009105		0.237	1.00	0.005228	0.02158	0.8084438
rs4902841	14	70249464	A	G	0.587	0.92	-0.164	0.041	0.0000693	0.0005095		0.601	0.94	-0.00273	0.01911	0.8862916
rs8021668	14	70258350	C	T	0.577	1.00	-0.154	0.039	0.0000947	0.0005294		0.597	0.99	0.002498	0.01855	0.8927713
rs3742848	14	70261176	C	T	0.423	1.00	0.153	0.039	0.0000966	0.0005472		0.403	0.99	-0.00255	0.01854	0.8904287
rs7156271	14	70263056	A	G	0.413	0.99	0.157	0.04	0.0000707	0.0004018		0.402	0.99	-0.003	0.01855	0.8712726
rs11625206	14	70263906	C	T	0.673	1.00	0.13	0.041	0.001452	0.0006605		0.662	0.85	-0.0155	0.02098	0.45967
rs11844774	14	70268304	C	T	0.423	1.00	0.152	0.039	0.0001115	0.000627		0.403	0.99	-0.00314	0.01849	0.8651323
rs699363	14	71762246	A	G	0.965	0.70	-0.373	0.126	0.003147	0.0007359		0.957	0.60	-0.20357	0.06085	0.0008179
rs12435198	14	73771322	C	T	0.813	1.00	-0.166	0.05	0.0009429	0.0010914		0.835	0.99	0.011122	0.02557	0.6632789
rs12435201	14	73771360	G	T	0.813	1.00	-0.167	0.05	0.0008855	0.0010489		0.835	0.98	0.010693	0.02567	0.6767161
rs10130040	14	73783435	A	C	0.186	1.00	0.166	0.05	0.0009739	0.0013218		0.165	0.97	-0.01005	0.02581	0.6966901
rs10142706	14	73787057	A	G	0.186	0.99	0.168	0.05	0.0008931	0.0012618		0.165	0.96	-0.00964	0.02589	0.7092822
rs11627178	14	73788033	C	G	0.814	0.99	-0.168	0.051	0.0008827	0.0012558		0.835	0.96	0.009423	0.02593	0.7160407
rs2021012	14	76683024	A	T	0.647	0.95	0.114	0.042	0.006	0.0009251		0.659	0.90	0.043716	0.02015	0.0299049
rs748686	14	76683611	C	G	0.647	0.95	0.114	0.042	0.005967	0.0009222		0.659	0.90	0.043876	0.02017	0.0294639
rs11159268	14	77043934	A	C	0.682	0.99	0.127	0.041	0.002089	0.0007604		0.644	0.95	0.024651	0.01967	0.2096908
rs10140593	14	79661751	A	G	0.620	1.00	-0.151	0.04	0.000155	0.0001407		0.610	0.99	0.030972	0.01877	0.0986694
rs10148083	14	80083468	C	T	0.843	1.00	0.153	0.053	0.003765	0.0004449		0.842	1.00	-0.01183	0.02521	0.6386607
rs7160604	14	80084658	C	T	0.843	1.00	0.153	0.053	0.003774	0.0004457		0.842	1.00	-0.01181	0.0252	0.6391276
rs7152418	14	87003159	A	C	0.016	0.99	0.525	0.16	0.001075	0.0008685		0.031	0.94	-0.08746	0.05234	0.094445
rs6420918	14	87003596	A	C	0.984	0.98	-0.531	0.161	0.001012	0.0008082		0.969	0.94	0.087817	0.0525	0.0941174
rs8006733	14	87005636	C	T	0.984	0.97	-0.539	0.163	0.0009653	0.0007466		0.970	0.94	0.089586	0.05291	0.0901843

					GOYA QC		GOYA Overweight/control			GOYA BMI continuous	known	IARC QC		IARC results		
MARKER	chr	position	A1	A2	FREQ1	Rsqr	Beta	SE	p	p	gene name	freq1	Rsqr	in_beta	in_SE	in_p
rs11850024	14	87015128	A	C	0.016	0.96	0.548	0.166	0.0009287	0.0006778		0.029	0.95	-0.09257	0.05355	0.083622
rs10483999	14	87018626	C	T	0.015	0.96	0.554	0.167	0.0008812	0.0006342		0.029	0.95	-0.09265	0.05362	0.0838133
rs2401694	14	87018915	G	T	0.985	0.96	-0.556	0.167	0.0008622	0.0006181		0.971	0.95	0.092696	0.05368	0.0839744
rs11852146	14	87022499	C	T	0.985	0.95	-0.57	0.169	0.0007393	0.0005171		0.972	0.94	0.093151	0.05452	0.0872918
rs11845141	14	87051144	A	G	0.014	0.99	0.621	0.175	0.000397	0.0002342		0.025	0.98	-0.08502	0.05673	0.1336602
rs11848625	14	87054344	A	C	0.014	0.99	0.619	0.175	0.0004147	0.0002383		0.025	0.96	-0.08563	0.05692	0.1321734
rs9919909	14	89473177	C	T	0.177	1.00	0.169	0.051	0.0008459	0.0000992		0.202	0.80	-0.03759	0.02571	0.1434645
rs10148012	14	89476406	C	T	0.177	1.00	0.169	0.051	0.0008397	0.0000979		0.202	0.80	-0.0378	0.02564	0.1400181
rs1286290	14	90383565	C	G	0.637	0.93	0.144	0.041	0.0004732	0.0005209		0.601	0.87	-0.00866	0.01989	0.6628891
rs1152431	14	90443043	A	G	0.516	1.00	0.135	0.039	0.0004975	0.0007033		0.540	1.00	0.017116	0.01844	0.3528551
rs1286135	14	90445534	A	C	0.730	1.00	0.162	0.043	0.0001838	0.0006896		0.730	0.99	-0.00957	0.0208	0.6450757
rs1286133	14	90448573	C	T	0.730	1.00	0.162	0.043	0.0001824	0.0006903		0.730	0.99	-0.00958	0.0208	0.6447822
rs1286124	14	90464884	G	T	0.270	1.00	-0.162	0.043	0.000181	0.0006844		0.270	0.99	0.009601	0.02079	0.6439595
rs1286118	14	90477981	A	T	0.738	1.00	0.166	0.044	0.0001429	0.0004023		0.737	1.00	-0.01043	0.02085	0.6165454
rs1286112	14	90482514	C	G	0.262	1.00	-0.166	0.044	0.000143	0.0004027		0.263	1.00	0.010431	0.02084	0.6164765
rs1286110	14	90485396	A	T	0.738	1.00	0.166	0.044	0.0001432	0.000403		0.737	1.00	-0.01043	0.02084	0.6163753
rs1286109	14	90486305	A	G	0.260	0.96	-0.161	0.045	0.0003094	0.0009965		0.262	0.97	0.010562	0.0213	0.6197494
rs1286106	14	90488187	C	G	0.270	1.00	-0.164	0.043	0.0001509	0.0005749		0.270	1.00	0.009854	0.02072	0.6341425
rs1296092	14	90494774	C	T	0.262	1.00	-0.166	0.044	0.0001438	0.0004045		0.263	1.00	0.010436	0.02081	0.6158175
rs1286097	14	90497010	C	T	0.738	1.00	0.166	0.044	0.0001453	0.0004085		0.737	1.00	-0.01003	0.02082	0.6296247
rs1297776	14	90497348	A	G	0.270	1.00	-0.164	0.043	0.000154	0.000585		0.270	1.00	0.009509	0.02073	0.6462115
rs1286096	14	90497415	C	T	0.262	1.00	-0.166	0.044	0.000151	0.0004239		0.263	1.00	0.010107	0.02084	0.6273497
rs3783832	14	90505458	C	T	0.285	1.00	-0.146	0.043	0.0006428	0.0039198		0.278	1.00	0.006391	0.02046	0.7545641
rs2401952	14	90507186	A	G	0.512	0.95	0.133	0.04	0.0008342	0.0024535		0.535	0.95	0.022876	0.01879	0.2230733
rs1286065	14	90521536	C	T	0.291	1.00	-0.162	0.042	0.0001295	0.0005331		0.266	0.99	0.001549	0.02075	0.9404461
rs1286146	14	90544201	A	G	0.718	1.00	0.163	0.043	0.0001385	0.0004448		0.742	0.99	-0.00136	0.02094	0.9481258
rs1286144	14	90546939	C	G	0.291	1.00	-0.162	0.042	0.0001245	0.0005131		0.265	0.99	0.000813	0.0208	0.9687824
rs1296093	14	90551958	A	G	0.291	1.00	-0.163	0.042	0.000124	0.0005108		0.265	0.99	0.000597	0.02082	0.9771011
rs1286138	14	90555198	G	T	0.672	1.00	0.18	0.041	0.0000126	0.0000493		0.699	0.99	-0.0031	0.0201	0.8773274
rs7150723	14	90559782	A	C	0.291	0.99	-0.163	0.042	0.0001198	0.000493		0.264	0.99	-9.2E-06	0.02086	0.9996482
rs7151354	14	90559858	A	G	0.717	0.98	0.162	0.043	0.0001735	0.0006319		0.744	0.96	0.005401	0.02146	0.8011454
rs8003112	14	90565126	C	T	0.710	0.99	0.164	0.042	0.000114	0.0004676		0.736	0.99	0.000196	0.0209	0.9925233
rs6575169	14	90567682	C	T	0.673	0.99	0.182	0.041	0.0000112	0.0000442		0.701	0.98	-0.00257	0.0202	0.8986104
rs2012280	14	90570825	G	T	0.301	1.00	-0.155	0.042	0.0001975	0.0011033		0.272	0.99	-0.01442	0.02058	0.4830588
rs7156252	14	90572871	C	T	0.700	1.00	0.155	0.042	0.0001987	0.0011058		0.728	0.99	0.014441	0.02057	0.4823866
rs1951454	14	90575121	A	G	0.284	0.99	-0.157	0.043	0.0002351	0.0009951		0.254	0.98	-0.00544	0.02117	0.7970264
rs10150964	14	90577033	C	T	0.700	1.00	0.156	0.042	0.000189	0.0010283		0.729	0.98	0.014487	0.02074	0.4843795
rs1951455	14	90582092	C	T	0.710	0.98	0.159	0.043	0.0001765	0.0009002		0.743	0.94	0.00567	0.02153	0.7921187
rs1951456	14	90582093	C	T	0.711	0.98	0.16	0.043	0.0001719	0.0008701		0.743	0.94	0.005678	0.02155	0.7919515
rs7156757	14	90582352	A	G	0.294	1.00	-0.16	0.042	0.0001388	0.0006885		0.265	0.93	-0.01461	0.02143	0.4952649
rs1285998	14	90582704	C	G	0.706	1.00	0.16	0.042	0.0001395	0.0006902		0.735	0.93	0.014608	0.02144	0.4953544
rs1285997	14	90582782	C	G	0.294	1.00	-0.16	0.042	0.0001412	0.0006947		0.265	0.93	-0.01461	0.02145	0.4954281
rs1075014	14	90600783	A	G	0.706	0.99	0.159	0.042	0.0001532	0.0007282		0.736	0.92	0.014619	0.02155	0.4972219
rs11159988	14	90600927	C	T	0.294	0.99	-0.16	0.042	0.0001526	0.0007263		0.264	0.92	-0.01461	0.02156	0.4976473
rs11624274	14	90606987	A	G	0.706	0.99	0.16	0.042	0.0001523	0.0007256		0.736	0.92	0.014599	0.02157	0.4981502
rs7492628	14	90616889	C	G	0.694	0.93	0.172	0.043	0.0000726	0.000485		0.720	0.90	0.010432	0.02163	0.6293707
rs11627189	14	90660709	A	G	0.961	0.98	0.336	0.102	0.00099	0.0033584		0.974	0.45	-0.19657	0.0884	0.0260656
rs11628275	14	90691201	A	G	0.039	0.82	-0.381	0.11	0.0005525	0.0006681		0.044	0.68	0.119436	0.05456	0.0284948
rs2145143	14	90698302	C	G	0.954	0.81	0.392	0.103	0.000147	0.0002127		0.953	0.66	-0.12337	0.05357	0.0212068
rs17127484	14	91172512	A	G	0.786	1.00	-0.178	0.047	0.0001755	0.000331		0.756	0.65	-0.0462	0.02623	0.0779794
rs1285655	14	91210181	A	G	0.261	1.00	0.152	0.044	0.0005591	0.0007176		0.324	0.73	0.042514	0.02309	0.0654126
rs11622488	14	94605375	A	G	0.966	0.75	0.436	0.126	0.000508	0.0018226		0.983	0.47	0.100172	0.08983	0.2643888
rs1187652	14	94624958	C	T	0.041	0.84	-0.429	0.109	0.0000823	0.0004955		0.025	0.64	-0.07248	0.06898	0.2929968
rs17095577	14	97098181	A	G	0.969	0.87	0.404	0.122	0.0009334	0.0007428		0.935	0.70	0.033475	0.04467	0.453221
rs4905992	14	100210843	C	T	0.025	0.83	-0.496	0.141	0.0004401	0.0016815		0.037	0.80	0.03356	0.0514	0.5134144
rs4572321	14	100875245	A	C	0.276	0.98	0.152	0.043	0.0004614	0.0008667		0.242	0.97	0.04574	0.02122	0.0309818
rs11626278	14	100885110	A	C	0.724	1.00	-0.151	0.043	0.0004516	0.0008775		0.760	0.99	-0.04544	0.02098	0.0301991
rs1959021	14	100886448	A	G	0.276	1.00	0.151	0.043	0.0004512	0.0008765		0.241	0.99	0.045455	0.02098	0.0301205
rs11629011	14	100887557	A	G	0.721	0.98	-0.154	0.043	0.0003818	0.0008232		0.755	0.97	-0.04574	0.02111	0.0301174
rs11621338	14	100888333	A	G	0.724	1.00	-0.151	0.043	0.0004495	0.0008724		0.760	1.00	-0.04548	0.02097	0.0300117
rs4900501	14	100890106	C	T	0.725	1.00	-0.151	0.043	0.000479	0.0009433		0.760	1.00	-0.04543	0.02098	0.0302141
rs12879397	14	100893266	A	G	0.725	1.00	-0.149	0.043	0.0005316	0.0010616		0.760	0.99	-0.04539	0.02099	0.0304661
rs4900502	14	100897818	C	G	0.275	1.00	0.151	0.043	0.0004827	0.0009843		0.241	0.99	0.045501	0.021	0.0301812
rs7149987	14	100898119	C	T	0.275	1.00	0.151	0.043	0.0004777	0.0009763		0.241	0.99	0.045501	0.02101	0.0301979
rs10138500	14	100899261	C	T	0.755	1.00	-0.153	0.045	0.0007046	0.0013281		0.784	0.97	-0.04707	0.02212	0.0332131
rs8011165	14	102688507	A	C	0.649	0.74	0.161	0.047	0.0006203	0.0004847		0.694	0.70	-0.01658	0.02378	0.4854232
rs11634027	15	24188508	A	G	0.020	0.59	-0.508	0.182	0.00536	0.000794		0.017	0.43	-0.02001	0.09473	0.8326061
rs8040523	15	28085050	A	G	0.040	0.96	0.333	0.1	0.0008988	0.0033034		0.060	0.90	-0.0357	0.04123	0.3861898
rs11636121	15	29126443	A	C	0.409	0.96	0.14	0.041	0.0005375	0.0004636		0.389	0.97	0.002263	0.01898	0.9050529
rs2879436	15	31887114	A	G	0.256	0.99	-0.153	0.044	0.0005646	0.0011086		0.270	0.70	0.00528	0.02541	0.8352838
rs4780184	15	31920167	A	G	0.327	0.93	-0.141	0.043	0.0009405	0.0000947		0.320	0.66	0.01101	0.02383	0.6438269
rs12441112	15	31921018	A	G	0.767	0.99	0.148	0.046	0.001239	0.0004874		0.761	0.63	-0.00204	0.02688	0.9393025
rs3889750	15	31978684	A	G	0.300	1.00	-0.115	0.042	0.005972	0.0009755		0.295	0.98	0.008373	0.02017	0.6778757
rs17696730	15	34215141	A	T	0.907	0.76	-0.262	0.077	0.0006278	0.0017498		0.906	0.68	-0.00675	0.03557	0.84927
rs11629908	15	34264165	C	T	0.933	0.99	-0.263	0.078	0.0007548	0.0016562		0.934	0.97	-0.00787	0.03449	0.8193756
rs17531839	15	35324939	C	T	0.452	0.99	0.129	0.039	0.0009812	0.0096362		0.424	0.98	-0.0325	0.01854	0.0793449
rs1869901	15	38382919	A	G	0.612	1.00	0.131	0.039	0.0008888	0.0019234		0.584	1.00	0.029275	0.01869	0.117055
rs3784397	15	38384136	C	T	0.377	0.99	-0.132	0.04	0.0009384	0.0025411		0.412	0.99	-0.02534	0.01877	0.1765877
rs2017012	15	45236677	A	G	0.543	1.00	0.087	0.039	0.02415	0.0003618		0.452	0.96	0.013143	0.01876	0.4832732
rs16961946	15	47041185	A	G	0.187	1.00	0.168	0.05	0.0008392	0.0000853		0.230	0.65	-0.05947	0.02766	0.0314278
rs1899731	15	50953478	C	T	0.330	1.00	0.108	0.041	0.008551	0.0006279		0.292	0.99	-0.01488	0.0196	0.4472168
rs335497	15	51170393	G	T	0.924	0.93	0.192	0.076	0.01163	0.0002214		0.922	0.84	-0.0038	0.03763	0.9194601

					GOYA QC		GOYA Overweight/control			GOYA BMI continuous	known	IARC QC		IARC results		
MARKER	chr	position	A1	A2	FREQ1	Rsqr	Beta	SE	p	p	gene name	freq1	Rsqr	in_beta	in_SE	in_p
rs335512	15	51181390	A	C	0.924	1.00	0.191	0.073	0.009047	0.0001639		0.922	0.99	-0.00223	0.03482	0.9488807
rs12148408	15	51193176	G	T	0.923	1.00	0.188	0.073	0.01019	0.0001909		0.922	0.99	-0.00256	0.03474	0.9411187
rs16965523	15	51198133	A	G	0.077	1.00	-0.189	0.073	0.009847	0.0001928		0.076	0.98	0.005996	0.03497	0.8637613
rs3848136	15	51212732	A	G	0.077	0.99	-0.198	0.073	0.006759	0.0001327		0.075	0.99	0.003401	0.03507	0.9226785
rs11070955	15	51228508	A	G	0.924	0.99	0.208	0.074	0.004763	0.0000821		0.925	0.99	-0.00522	0.03526	0.8821235
rs11070956	15	51230222	A	G	0.076	0.99	-0.208	0.074	0.004663	0.00008		0.075	0.99	0.005268	0.03527	0.8811545
rs16965603	15	51242302	C	T	0.926	0.98	0.199	0.075	0.007923	0.0001263		0.926	0.97	-0.00558	0.03578	0.8759717
rs8024806	15	51261282	C	T	0.075	0.91	-0.173	0.078	0.02609	0.0009389		0.076	0.80	0.007415	0.0398	0.8520879
rs3816815	15	57254281	C	G	0.139	0.97	-0.19	0.057	0.0008041	0.0074287		0.156	0.96	-0.00682	0.02495	0.7842978
rs7162383	15	61453473	A	C	0.961	0.76	-0.408	0.116	0.0004293	0.0028751		0.979	0.63	0.063802	0.07462	0.3921248
rs16950615	15	65203806	C	G	0.955	0.97	0.321	0.095	0.0007265	0.0048134		0.937	0.77	0.054838	0.04165	0.1875969
rs16950635	15	65205259	A	G	0.045	0.98	-0.318	0.095	0.0007683	0.0051167		0.064	0.78	-0.05465	0.04154	0.1879263
rs1529768	15	68369395	C	G	0.769	0.99	-0.108	0.045	0.01757	0.000686		0.781	0.98	-0.06737	0.0219	0.0020852
rs1912967	15	68370390	A	T	0.769	0.99	-0.108	0.045	0.01751	0.0006816		0.781	0.98	-0.06735	0.0219	0.0020922
rs292853	15	68385219	G	T	0.228	1.00	0.109	0.046	0.01665	0.0008914		0.221	1.00	0.064532	0.02174	0.0029748
rs2554818	15	68385295	A	G	0.228	1.00	0.109	0.046	0.01666	0.000898		0.221	1.00	0.064474	0.02174	0.0030015
rs11631406	15	77232844	C	T	0.432	1.00	-0.12	0.039	0.002111	0.0005574		0.431	0.98	-0.0014	0.01859	0.9397579
rs922878	15	79652902	C	T	0.160	0.99	0.164	0.053	0.001831	0.000932		0.156	0.99	-0.00424	0.02499	0.8650957
rs1482934	15	79653713	C	T	0.160	0.99	0.166	0.053	0.001622	0.0008436		0.156	0.99	-0.00427	0.02497	0.8642712
rs2672093	15	79660292	A	G	0.159	0.99	0.173	0.053	0.001061	0.0005988		0.156	1.00	-0.00436	0.02489	0.8606806
rs2624996	15	79668790	A	G	0.843	1.00	-0.177	0.053	0.0008126	0.0004676		0.845	1.00	0.002166	0.025	0.9308799
rs1482943	15	79675391	A	G	0.844	1.00	-0.166	0.053	0.001735	0.0008123		0.844	1.00	0.006296	0.02488	0.800073
rs9783695	15	79676298	G	T	0.156	1.00	0.166	0.053	0.001763	0.0008341		0.156	1.00	-0.00641	0.0249	0.7968235
rs6495599	15	79677666	A	G	0.150	0.96	0.171	0.055	0.001896	0.0009227		0.146	0.94	-0.00717	0.02616	0.7838815
rs11854289	15	80345857	C	T	0.435	1.00	-0.141	0.039	0.0003499	0.002501		0.393	1.00	0.012439	0.01872	0.5061067
rs11854483	15	80346613	C	T	0.435	1.00	-0.141	0.039	0.0003499	0.0025012		0.393	1.00	0.012405	0.01872	0.5072455
rs13313527	15	86171266	A	G	0.363	1.00	0.12	0.041	0.003317	0.0008195		0.412	0.99	-0.00671	0.01853	0.7171941
rs7161806	15	86253599	A	G	0.437	0.77	-0.113	0.044	0.01105	0.000736		0.399	0.72	0.022469	0.02191	0.3048263
rs11073754	15	86311173	A	G	0.215	1.00	0.154	0.047	0.001022	0.000883		0.276	0.75	0.041899	0.02379	0.0780388
rs12592042	15	86734218	A	C	0.010	0.73	-0.693	0.235	0.003176	0.0005873		0.010	0.62	0.09891	0.10532	0.3472539
rs16941785	15	86735203	A	G	0.990	0.73	0.689	0.234	0.003263	0.0006196		0.990	0.64	-0.09302	0.10311	0.3666082
rs16941788	15	86735686	C	G	0.990	0.73	0.688	0.234	0.0033	0.0006327		0.990	0.64	-0.09145	0.10253	0.3720464
rs16941790	15	86736310	A	G	0.990	0.73	0.686	0.234	0.003338	0.0006476		0.989	0.65	-0.09049	0.10214	0.3752626
rs8028203	15	86736615	A	G	0.011	0.74	-0.664	0.23	0.003956	0.0008723		0.011	0.66	0.087351	0.10092	0.3863322
rs8029945	15	86736908	C	G	0.990	0.74	0.663	0.23	0.003969	0.0008778		0.989	0.66	-0.08569	0.10018	0.3919412
rs7174952	15	86738320	A	G	0.011	0.75	-0.658	0.229	0.004064	0.0009356		0.011	0.68	0.079407	0.09776	0.4162385
rs7180499	15	86738967	G	T	0.989	0.75	0.674	0.223	0.002546	0.0005659		0.989	0.69	-0.07829	0.09749	0.4215658
rs7165137	15	86739040	C	T	0.011	0.75	-0.676	0.222	0.002379	0.0005264		0.012	0.70	0.073218	0.09601	0.4452997
rs12594217	15	86766143	C	T	0.019	0.99	-0.449	0.145	0.002015	0.0007607		0.018	0.80	0.042884	0.07494	0.5668563
rs10220837	15	86775881	C	T	0.019	0.98	-0.456	0.147	0.001877	0.0006847		0.018	0.80	0.042991	0.07519	0.5671751
rs7496743	15	86784141	G	T	0.981	0.98	0.456	0.147	0.001871	0.0006807		0.982	0.78	-0.04268	0.0764	0.5761074
rs7166118	15	86786514	C	T	0.030	0.72	-0.485	0.138	0.0004555	0.0005279		0.030	0.56	-0.02806	0.07093	0.6921442
rs6496484	15	86786678	A	C	0.019	0.99	-0.455	0.146	0.001896	0.0006862		0.018	0.77	0.042469	0.07671	0.5795313
rs11854995	15	86789213	G	T	0.019	0.99	-0.456	0.147	0.001893	0.0006841		0.018	0.77	0.04251	0.07673	0.5792529
rs7182360	15	86792891	C	T	0.021	0.59	-0.631	0.184	0.0006273	0.0002078		0.020	0.60	0.04199	0.08303	0.6127623
rs11855748	15	86795694	A	G	0.988	0.76	0.719	0.216	0.0008798	0.0002051		0.986	0.74	-0.04194	0.09139	0.6459691
rs11858838	15	86795717	A	G	0.012	0.75	-0.726	0.218	0.0008731	0.0002017		0.014	0.74	0.04179	0.0914	0.6472507
rs7167341	15	86796854	A	G	0.021	0.46	-0.693	0.21	0.0009921	0.0002788		0.022	0.49	0.026177	0.09079	0.7729014
rs16941873	15	86804563	A	T	0.989	0.71	0.761	0.228	0.0008418	0.0001898		0.986	0.73	-0.0417	0.09151	0.648379
rs6496486	15	86806342	G	T	0.989	0.71	0.762	0.228	0.000842	0.0001898		0.986	0.73	-0.04155	0.09158	0.6497504
rs3809584	15	86810107	A	G	0.989	0.71	0.763	0.228	0.0008447	0.0001901		0.986	0.73	-0.04153	0.0916	0.6499588
rs16941911	15	86812636	A	G	0.011	0.71	-0.768	0.23	0.0008489	0.0001904		0.014	0.73	0.041357	0.09171	0.6517332
rs7176734	15	86816290	G	T	0.989	0.71	0.769	0.23	0.0008485	0.0001899		0.986	0.73	-0.04129	0.09176	0.6524886
rs2289416	15	86816754	A	C	0.989	0.70	0.774	0.232	0.0008439	0.0001886		0.986	0.73	-0.0411	0.09191	0.654474
rs16941960	15	86823753	C	T	0.989	0.70	0.774	0.232	0.0008442	0.0001884		0.986	0.73	-0.04103	0.09195	0.6551651
rs16941972	15	86825924	G	T	0.989	0.69	0.789	0.236	0.0008408	0.0001863		0.986	0.73	-0.04093	0.09201	0.6561818
rs11858314	15	86827572	C	T	0.011	0.68	-0.792	0.237	0.0008406	0.0001854		0.014	0.72	0.038427	0.09309	0.6795046
rs16941976	15	86829106	C	T	0.011	0.68	-0.793	0.237	0.0008399	0.0001851		0.014	0.72	0.038327	0.0931	0.6803279
rs16941984	15	86829941	C	T	0.011	0.68	-0.797	0.239	0.0008393	0.0001841		0.014	0.71	0.037955	0.09327	0.6837987
rs1000301	15	86830288	A	G	0.011	0.68	-0.798	0.239	0.0008392	0.0001842		0.014	0.71	0.037894	0.09332	0.6844414
rs12101433	15	86833976	A	C	0.011	0.68	-0.798	0.239	0.000837	0.0001837		0.014	0.71	0.037839	0.09333	0.6849236
rs12101408	15	86834123	A	G	0.011	0.68	-0.799	0.239	0.0008382	0.0001834		0.014	0.71	0.036625	0.09368	0.6955913
rs16941985	15	86834672	C	T	0.010	0.64	-0.843	0.253	0.000883	0.0001873		0.014	0.71	0.036435	0.09372	0.6971973
rs744886	15	86834723	C	T	0.990	0.64	0.844	0.254	0.0008792	0.0001865		0.986	0.71	-0.03645	0.09373	0.6970946
rs2169934	15	93042147	C	T	0.414	0.96	0.134	0.041	0.0009365	0.0065858		0.477	0.47	-0.0484	0.02625	0.0650464
rs1026750	15	93043126	G	T	0.410	0.99	0.133	0.04	0.0008507	0.0068389		0.474	0.47	-0.04883	0.02618	0.0619741
rs6496036	15	93050906	A	C	0.580	0.89	0.158	0.042	0.0001821	0.0004102		0.601	0.76	-0.05178	0.02125	0.0147532
rs899463	15	93053087	C	T	0.651	0.98	-0.139	0.041	0.0007666	0.0024436		0.666	0.95	0.045116	0.01978	0.0224726
rs899464	15	93053533	C	T	0.642	0.96	-0.14	0.042	0.000753	0.0027654		0.658	0.93	0.045465	0.01988	0.022139
rs2046003	15	93053671	A	T	0.651	0.98	-0.139	0.041	0.0007767	0.0024558		0.666	0.95	0.045113	0.01977	0.0223904
rs2388778	15	93054581	A	G	0.349	0.98	0.139	0.041	0.0007829	0.0024633		0.334	0.95	-0.04509	0.01975	0.0223405
rs736664	15	93055139	G	T	0.349	0.98	0.138	0.041	0.0007989	0.0024784		0.334	0.96	-0.04509	0.01974	0.0222632
rs2388779	15	93058282	C	T	0.670	0.99	-0.142	0.041	0.0006402	0.0026004		0.694	0.96	0.034694	0.02005	0.0833972
rs11637906	15	93059245	C	T	0.669	1.00	-0.137	0.041	0.000877	0.0028189		0.693	0.97	0.03484	0.01993	0.0802797
rs12901421	15	93060797	A	G	0.481	1.00	-0.144	0.039	0.0002089	0.0003297		0.449	0.98	0.042632	0.01855	0.0214808
rs2199722	15	93060885	A	C	0.674	0.97	-0.144	0.042	0.000596	0.0025487		0.700	0.95	0.036222	0.0203	0.0741335
rs7168523	15	93064915	C	T	0.373	1.00	0.137	0.04	0.0005909	0.0065826		0.406	1.00	-0.02483	0.01864	0.1824455
rs11073381	15	93066016	G	T	0.495	1.00	-0.151	0.039	0.0001009	0.0002413		0.453	1.00	0.043388	0.0184	0.0183109
rs7170128	15	93066856	A	G	0.496	1.00	-0.151	0.039	0.0001008	0.0002411		0.453	1.00	0.043419	0.0184	0.0182045
rs7170018	15	93066997	A	T	0.373	1.00	0.137	0.04	0.0005807	0.0064716		0.406	1.00	-0.0251	0.01863	0.177536
rs8036171	15	93067814	A	C	0.372	1.00	0.137	0.04	0.000573	0.0063874		0.406	1.00	-0.02527	0.01862	0.174484
rs8037823	15	93067984	C	T	0.628	1.00	-0.138	0.04	0.0005362	0.0061514		0.594	1.00	0.025024	0.01863	0.1788541

					GOYA QC		GOYA Overweight/control			GOYA BMI continuous	known	IARC QC		IARC results		
MARKER	chr	position	A1	A2	FREQ1	Rsqr	Beta	SE	p	p	gene name	freq1	Rsqr	in_beta	in_SE	in_p
rs7181659	15	93068487	A	G	0.504	1.00	0.152	0.039	0.0000917	0.0002269		0.547	1.00	-0.0434	0.01839	0.0182218
rs11634297	15	93068568	A	T	0.496	1.00	-0.152	0.039	0.0000889	0.0002225		0.453	1.00	0.043364	0.01839	0.0183181
rs9302331	15	93069024	C	T	0.496	1.00	-0.153	0.039	0.0000787	0.0002062		0.453	1.00	0.043293	0.01839	0.0185111
rs4984406	15	93069498	C	T	0.496	1.00	-0.154	0.039	0.000072	0.0001952		0.453	1.00	0.043179	0.01839	0.0188143
rs7173947	15	93071471	C	T	0.372	1.00	0.143	0.04	0.0003232	0.0044164		0.409	1.00	-0.02216	0.01861	0.2332027
rs7178731	15	93071736	A	G	0.372	1.00	0.143	0.04	0.00032	0.0043838		0.409	1.00	-0.02219	0.01861	0.2326215
rs11633473	15	93071909	A	G	0.628	1.00	-0.143	0.04	0.0003208	0.0043922		0.591	1.00	0.022245	0.01861	0.2315899
rs11638765	15	93072168	A	T	0.628	1.00	-0.143	0.04	0.0003218	0.0044026		0.591	1.00	0.022284	0.01861	0.2308689
rs7179870	15	93072268	C	G	0.372	1.00	0.143	0.04	0.0003223	0.0044093		0.409	1.00	-0.0223	0.01862	0.2305881
rs11633659	15	93072364	C	G	0.628	1.00	-0.143	0.04	0.0003236	0.004422		0.591	1.00	0.022318	0.01862	0.2302527
rs11633626	15	93072382	A	C	0.628	1.00	-0.143	0.04	0.0003284	0.0044714		0.591	1.00	0.022326	0.01862	0.2301151
rs7181498	15	93072408	C	T	0.628	1.00	-0.143	0.04	0.0003308	0.0044964		0.591	1.00	0.022399	0.01863	0.2287559
rs8025516	15	93072876	G	T	0.628	1.00	-0.143	0.04	0.0003328	0.0045152		0.591	1.00	0.02242	0.01863	0.228374
rs2046002	15	93073239	C	T	0.622	1.00	-0.134	0.04	0.000714	0.0082563		0.586	0.98	0.023202	0.01861	0.2120478
rs7169847	15	93073924	G	T	0.383	1.00	0.137	0.04	0.0005438	0.0072756		0.415	0.98	-0.02534	0.01863	0.1733177
rs11073382	15	93075281	A	G	0.479	1.00	-0.144	0.039	0.000202	0.0003242		0.445	0.98	0.03863	0.01848	0.0364106
rs11073383	15	93075353	A	G	0.479	1.00	-0.144	0.039	0.0002029	0.0003254		0.445	0.98	0.038613	0.01847	0.0364882
rs7183500	15	93901927	A	G	0.686	1.00	-0.141	0.042	0.0006726	0.0080569		0.655	0.68	-0.01644	0.02385	0.4903419
rs7183672	15	93902022	A	G	0.697	0.96	-0.154	0.043	0.0002968	0.002339		0.679	0.76	-0.01962	0.02299	0.393069
rs6496121	15	93902501	C	G	0.697	0.96	-0.154	0.043	0.000301	0.0023232		0.679	0.76	-0.01965	0.02295	0.3915829
rs10468114	15	93919961	A	G	0.358	0.95	0.136	0.041	0.000901	0.0025952		0.396	0.90	0.006822	0.02006	0.7335787
rs8028726	15	93932964	G	T	0.629	0.95	-0.134	0.041	0.0009591	0.0031484		0.600	0.73	-0.01405	0.02227	0.527765
rs4581667	15	93938912	C	T	0.340	0.67	0.193	0.049	0.0000985	0.0017114		0.356	0.56	0.018662	0.02595	0.4716156
rs2397806	15	93940144	C	G	0.341	0.66	0.194	0.05	0.0001017	0.0017874		0.357	0.55	0.018499	0.02627	0.480959
rs12593006	15	95047389	A	G	0.163	0.33	0.342	0.091	0.000166	0.0022656		0.169	0.32	0.02065	0.04378	0.6368504
rs2654528	15	95080636	A	G	0.855	0.96	-0.17	0.056	0.002495	0.0001004		0.821	0.88	-0.01015	0.02512	0.6859098
rs970843	15	96693552	C	G	0.170	0.93	-0.158	0.053	0.003085	0.0009069		0.146	0.88	-0.07127	0.02736	0.0091403
rs8042984	15	96714385	C	T	0.812	1.00	0.154	0.05	0.001838	0.0009577		0.825	0.99	0.050127	0.02429	0.0388853
rs2898887	15	99881699	A	G	0.431	0.92	0.141	0.041	0.0005009	0.0082158		0.444	0.61	0.019094	0.0229	0.4040703
rs2100045	15	99882157	A	G	0.410	0.96	0.146	0.04	0.0002595	0.0048085		0.414	0.66	0.019054	0.02222	0.3906921
rs7359236	15	99882858	G	T	0.590	0.96	-0.146	0.04	0.0002603	0.0048243		0.586	0.66	-0.01899	0.0222	0.3918085
rs7359250	15	99883121	C	T	0.571	0.99	-0.14	0.039	0.0003579	0.0049248		0.555	0.72	-0.01591	0.02127	0.4540617
rs2084148	15	99883984	C	T	0.571	0.99	-0.14	0.039	0.0003597	0.0049589		0.555	0.73	-0.01567	0.02118	0.4589452
rs2235487	16	1670312	A	G	0.760	1.00	-0.149	0.045	0.0009824	0.0066696		0.739	1.00	-0.02056	0.02046	0.314673
rs8045169	16	1812460	C	T	0.970	0.43	0.708	0.179	0.0000777	0.0013596		0.987	0.38	0.024603	0.12038	0.8379178
rs960274	16	3016664	C	T	0.985	0.52	0.594	0.227	0.008731	0.0008621		0.980	0.46	0.109086	0.10411	0.2943605
rs4786427	16	3658445	A	G	0.614	0.99	-0.125	0.04	0.001649	0.00037		0.620	0.98	-0.00379	0.01903	0.8418162
rs12444804	16	3663249	A	C	0.400	0.95	0.132	0.04	0.001084	0.0002629		0.379	0.94	-0.00349	0.01936	0.8566067
rs2074805	16	3664858	A	T	0.611	0.97	-0.138	0.04	0.0005772	0.0001559		0.632	0.97	0.002332	0.01923	0.9033907
rs2238436	16	3965386	G	T	0.758	0.99	0.176	0.045	0.0001039	0.0002699		0.699	0.99	0.025632	0.02053	0.2115876
rs2072346	16	3967424	C	T	0.178	0.99	-0.176	0.051	0.0005224	0.0009546		0.217	0.99	-0.02355	0.02289	0.3032654
rs2240735	16	3967606	C	T	0.241	0.99	-0.177	0.045	0.0001026	0.0002675		0.302	0.99	-0.02538	0.02049	0.2150679
rs2238439	16	3971349	A	G	0.219	1.00	-0.164	0.047	0.000453	0.0007869		0.269	1.00	-0.03115	0.02134	0.1441424
rs2531982	16	3973098	A	G	0.749	0.99	-0.169	0.045	0.0001636	0.0001027		0.775	0.81	-0.04307	0.0236	0.067799
rs9928772	16	4289111	A	C	0.019	0.33	0.96	0.261	0.0002323	0.0041931		0.023	0.22	0.09347	0.12689	0.4609656
rs17139483	16	6153750	C	T	0.845	1.00	0.171	0.054	0.001449	0.0008153		0.831	0.79	0.024923	0.02701	0.3558353
rs1478702	16	7086478	A	G	0.336	0.97	-0.14	0.042	0.0007469	0.0005097		0.334	0.75	0.01285	0.02204	0.5595235
rs694773	16	9419761	C	T	0.134	0.99	-0.213	0.058	0.0002208	0.0004892		0.139	0.99	-0.01877	0.02662	0.4803538
rs249279	16	9423179	C	T	0.279	1.00	-0.175	0.043	0.0000504	0.0005698		0.287	0.99	-0.00426	0.02034	0.8338508
rs249278	16	9423371	A	T	0.279	1.00	-0.175	0.043	0.0000511	0.0005767		0.287	1.00	-0.00429	0.02033	0.8328007
rs183657	16	9423591	C	T	0.135	1.00	-0.213	0.057	0.0002097	0.0004472		0.140	0.99	-0.01836	0.0265	0.4881932
rs8059436	16	9423816	C	T	0.280	1.00	-0.174	0.043	0.0000549	0.0006129		0.287	1.00	-0.00444	0.0203	0.8265845
rs249277	16	9424141	C	T	0.720	1.00	0.174	0.043	0.0000558	0.0006215		0.713	1.00	0.004453	0.0203	0.8262266
rs190345	16	9424148	C	T	0.865	1.00	0.213	0.057	0.0002065	0.0004338		0.860	1.00	0.018114	0.02643	0.4927356
rs249276	16	9424185	G	T	0.720	1.00	0.173	0.043	0.0000575	0.0006374		0.713	1.00	0.004503	0.02029	0.8241842
rs837351	16	9424493	A	G	0.865	1.00	0.213	0.057	0.0001997	0.0004187		0.860	1.00	0.018122	0.02641	0.4922438
rs837353	16	9425994	C	T	0.135	1.00	-0.214	0.057	0.0001834	0.0003857		0.140	1.00	-0.01843	0.02641	0.4849372
rs17681985	16	9426726	A	G	0.865	1.00	0.216	0.057	0.0001642	0.0003468		0.860	1.00	0.01904	0.02639	0.4701859
rs837354	16	9427428	A	T	0.864	1.00	0.218	0.057	0.0001373	0.0002673		0.859	1.00	0.020121	0.02641	0.4458044
rs930096	16	9429971	A	G	0.888	0.99	0.217	0.062	0.0004317	0.0009057		0.882	0.99	0.009644	0.02889	0.7382914
rs16965263	16	9439808	A	G	0.276	0.99	-0.184	0.044	0.0000223	0.0002674		0.282	0.97	-0.00357	0.02065	0.8626766
rs16965266	16	9440479	C	T	0.724	0.99	0.185	0.044	0.0000219	0.0002639		0.718	0.97	0.003526	0.02068	0.8644979
rs11075335	16	9441595	A	T	0.456	0.96	0.137	0.04	0.0006032	0.0034115		0.388	0.96	0.018969	0.01897	0.3170155
rs1019798	16	9441700	A	G	0.544	0.96	-0.137	0.04	0.0005985	0.0033907		0.612	0.96	-0.01888	0.01897	0.3192131
rs2304474	16	9443019	G	T	0.884	1.00	0.235	0.061	0.0001148	0.0002564		0.881	1.00	0.00844	0.02845	0.7665087
rs1014568	16	9443216	C	T	0.457	0.96	0.137	0.04	0.0005904	0.0033508		0.388	0.96	0.018888	0.01897	0.3190358
rs9931240	16	9443972	A	G	0.531	0.93	-0.142	0.04	0.0004522	0.0034301		0.598	0.89	-0.0173	0.01947	0.3738224
rs11640563	16	9444132	A	G	0.471	0.93	0.158	0.04	0.0000938	0.0009199		0.398	0.93	0.019248	0.01918	0.3151977
rs1428795	16	9444348	C	G	0.283	0.97	-0.188	0.043	0.0000144	0.0001944		0.282	0.94	-0.00672	0.02091	0.747906
rs9933849	16	9445381	G	T	0.540	1.00	-0.135	0.039	0.0005331	0.0025002		0.618	1.00	-0.0201	0.01859	0.2790502
rs2075152	16	9445562	C	T	0.539	1.00	-0.135	0.039	0.0005328	0.0024695		0.618	1.00	-0.02011	0.01858	0.2788062
rs2075153	16	9445760	C	T	0.540	0.99	-0.137	0.039	0.0004395	0.0021568		0.618	1.00	-0.02012	0.01859	0.278544
rs1898230	16	9446186	G	T	0.460	0.99	0.137	0.039	0.0004365	0.002146		0.382	1.00	0.020137	0.01859	0.2783579
rs759318	16	9447087	A	G	0.532	0.99	-0.132	0.039	0.0007322	0.0029298		0.600	0.98	-0.01519	0.01856	0.4129892
rs16965285	16	9451009	A	T	0.827	0.96	0.221	0.052	0.0000256	0.0000328		0.845	0.91	0.010422	0.02634	0.6920827
rs765081	16	9456164	A	C	0.420	0.96	0.149	0.04	0.0002196	0.0009608		0.349	0.96	0.013798	0.01933	0.4749731
rs1428801	16	9456460	C	T	0.561	1.00	-0.144	0.039	0.0002274	0.0011548		0.634	1.00	-0.01652	0.01875	0.3780746
rs1428803	16	9461328	A	G	0.410	0.97	-0.133	0.04	0.000882	0.0007719		0.485	0.92	-0.01523	0.01876	0.4163461
rs1024784	16	9461846	A	C	0.437	0.94	0.167	0.041	0.0000387	0.0002412		0.359	0.88	0.018118	0.02008	0.3665285
rs7202570	16	9462870	A	T	0.175	0.96	-0.218	0.052	0.0000323	0.0000408		0.160	0.88	-0.0058	0.0264	0.8260585
rs7188066	16	9462965	C	T	0.826	0.96	0.217	0.052	0.0000323	0.0000408		0.840	0.88	0.005788	0.0264	0.8262873
rs1428804	16	9462990	C	T	0.826	0.95	0.217	0.052	0.0000324	0.000041		0.840	0.88	0.005779	0.0264	0.8265666

					GOYA QC		GOYA Overweight/control			GOYA BMI continuous	known	IARC QC		IARC results		
MARKER	chr	position	A1	A2	FREQ1	Rsqr	Beta	SE	p	p	gene name	freq1	Rsqr	in_beta	in_SE	in_p
rs9930055	16	9464414	A	C	0.454	0.97	0.162	0.04	0.0000434	0.0003088		0.369	0.89	0.02214	0.01976	0.2621211
rs4780421	16	9469426	C	T	0.355	0.88	-0.182	0.043	0.0000242	0.0000738		0.372	0.83	-0.00891	0.02046	0.6631064
rs887948	16	9469869	A	G	0.355	0.86	-0.187	0.044	0.0000177	0.0000666		0.365	0.81	-0.0076	0.02086	0.7154511
rs759321	16	9470246	A	T	0.432	0.77	-0.148	0.044	0.0008521	0.0015693		0.431	0.67	-0.00345	0.02231	0.8768852
rs11074725	16	10311911	C	T	0.560	0.96	-0.117	0.04	0.003019	0.0004798		0.558	0.94	0.016606	0.01899	0.3814005
rs12446552	16	11627567	C	T	0.290	0.95	-0.15	0.044	0.0006789	0.0003941		0.257	0.92	-0.03323	0.02148	0.1214602
rs7204584	16	13227230	A	G	0.204	1.00	-0.161	0.049	0.0009853	0.0039098		0.160	0.37	0.004328	0.04002	0.9138097
rs2100401	16	16867138	G	T	0.559	0.56	-0.174	0.052	0.0008216	0.0007403		0.573	0.50	-0.03184	0.02586	0.2178648
rs4344725	16	16872606	A	G	0.601	1.00	-0.13	0.039	0.0009474	0.0050672		0.589	0.82	0.005994	0.02081	0.7730923
rs7206357	16	16876075	C	T	0.614	1.00	0.136	0.04	0.0007142	0.0030183		0.609	1.00	-0.00241	0.01863	0.8969488
rs4238659	16	16882437	C	T	0.388	1.00	-0.137	0.04	0.0006543	0.0036084		0.398	0.98	0.003144	0.01873	0.8665966
rs17755585	16	23937673	C	T	0.854	1.00	-0.161	0.055	0.003553	0.0008688		0.845	0.89	-0.00801	0.02665	0.7635344
rs7199551	16	23941146	G	T	0.491	0.96	-0.134	0.039	0.0006702	0.0006001		0.468	0.93	-0.02286	0.01895	0.2274288
rs1014632	16	24007245	A	T	0.549	0.92	-0.137	0.041	0.0008434	0.0011478		0.473	0.92	-0.00427	0.01897	0.8216748
rs2636957	16	24009089	A	G	0.562	1.00	-0.13	0.039	0.0009862	0.0015602		0.492	1.00	0.000557	0.01821	0.9755647
rs2560406	16	24010063	G	T	0.547	0.97	-0.134	0.04	0.0007879	0.0011349		0.471	0.94	-0.00381	0.01875	0.8387684
rs880824	16	24011829	A	G	0.377	1.00	0.135	0.04	0.0008165	0.0014261		0.422	1.00	-0.00656	0.01837	0.7206979
rs174828	16	24035866	A	C	0.477	0.82	0.161	0.043	0.0001828	0.0003851		0.480	0.47	-0.02784	0.02697	0.3015176
rs169142	16	24039431	A	G	0.545	0.97	-0.14	0.04	0.0004254	0.0007969		0.538	0.54	0.024688	0.02501	0.3232706
rs182068	16	24042184	C	G	0.537	0.98	-0.142	0.039	0.000303	0.000525		0.531	0.56	0.021485	0.02469	0.3837962
rs151181	16	28398018	C	T	0.407	0.95	0.125	0.04	0.001924	0.0006907	SH2B1					
rs180743	16	28415145	C	G	0.565	0.97	-0.117	0.04	0.003328	0.000457	SH2B1					
rs4788084	16	28447349	C	T	0.560	1.00	-0.116	0.039	0.00306	0.0004648	SH2B1					
rs12446550	16	28450882	A	G	0.440	1.00	0.116	0.039	0.003053	0.000464	SH2B1					
rs2008514	16	28733106	A	G	0.416	1.00	0.13	0.039	0.0009659	0.0004227	SH2B1					
rs8049439	16	28745016	C	T	0.419	1.00	0.124	0.039	0.001506	0.0005916	SH2B1					
rs8062405	16	28745407	A	G	0.584	1.00	-0.13	0.039	0.0009664	0.000424	SH2B1					
rs12443881	16	28749278	C	T	0.584	1.00	-0.13	0.039	0.0009674	0.0004242	SH2B1					
rs12928404	16	28754747	C	T	0.424	1.00	0.125	0.039	0.001387	0.0004805	SH2B1					
rs12325113	16	28756169	C	T	0.416	1.00	0.13	0.039	0.0009694	0.0004245	SH2B1					
rs12325278	16	28756319	A	G	0.584	1.00	-0.13	0.039	0.0009677	0.0004267	SH2B1					
rs4788099	16	28763228	A	G	0.584	1.00	-0.13	0.039	0.0009679	0.0004277	SH2B1					
rs7187776	16	28765146	A	G	0.581	1.00	-0.124	0.039	0.001512	0.000599	SH2B1					
rs4788100	16	28772174	C	T	0.416	1.00	0.13	0.039	0.0009682	0.0004288	SH2B1					
rs7205323	16	28773393	C	T	0.584	1.00	-0.13	0.039	0.0009681	0.0004288	SH2B1					
rs7187333	16	28773417	A	G	0.416	1.00	0.13	0.039	0.0009679	0.0004287	SH2B1					
rs4788101	16	28775305	C	T	0.578	0.98	-0.126	0.04	0.001469	0.000775	SH2B1					
rs4788102	16	28780899	A	G	0.416	1.00	0.13	0.039	0.0009676	0.0004286	SH2B1					
rs8055982	16	28788703	A	C	0.584	1.00	-0.129	0.039	0.0009764	0.0004311	SH2B1					
rs7498665	16	28790742	A	G	0.584	1.00	-0.129	0.039	0.001002	0.0004385	SH2B1					
rs7359397	16	28793160	C	T	0.584	1.00	-0.129	0.039	0.001049	0.0004521	SH2B1					
rs3888190	16	28796987	A	C	0.416	1.00	0.129	0.039	0.001069	0.0004577	SH2B1					
rs8055138	16	28798966	C	T	0.584	1.00	-0.128	0.039	0.001089	0.0004635	SH2B1					
rs8061590	16	28802631	A	G	0.584	1.00	-0.128	0.039	0.001111	0.0004702	SH2B1					
rs1364182	16	29563594	A	C	0.069	1.00	-0.272	0.078	0.0004577	0.0004518	SH2B1					
rs1364184	16	29563711	A	G	0.931	1.00	0.273	0.078	0.0004452	0.0004424	SH2B1					
rs4788172	16	29575754	A	G	0.063	1.00	-0.271	0.081	0.0008558	0.0007518	SH2B1					
rs3764275	16	29580483	A	C	0.937	1.00	0.274	0.082	0.0008056	0.0006568	SH2B1					
rs3764276	16	29580704	C	T	0.072	0.99	-0.264	0.076	0.0005314	0.0006032	SH2B1					
rs3759987	16	29581404	G	T	0.937	1.00	0.274	0.082	0.0008034	0.0006469	SH2B1					
rs11574550	16	29581471	C	G	0.937	0.99	0.274	0.082	0.0008028	0.0006461	SH2B1					
rs11574552	16	29581534	C	T	0.072	0.99	-0.265	0.076	0.0005228	0.0005918	SH2B1					
rs2071420	16	29582324	A	G	0.928	0.99	0.266	0.076	0.0005185	0.0005848	SH2B1					
rs1050881	16	29583429	C	T	0.937	0.95	0.28	0.083	0.0007432	0.0005643	SH2B1					
rs41212	16	47881321	A	G	0.215	1.00	0.146	0.047	0.001939	0.0004165		0.229	0.98	-0.01301	0.02181	0.5504548
rs7195955	16	47905250	C	T	0.558	0.97	-0.161	0.039	0.0000399	0.000013		0.544	0.96	0.018704	0.01854	0.312659
rs2883894	16	47908190	A	T	0.548	0.99	-0.155	0.039	0.0000604	0.0000225		0.532	0.99	0.017639	0.01822	0.3326946
rs1894975	16	47908246	G	T	0.452	0.99	0.155	0.039	0.0000604	0.0000225		0.468	0.99	-0.01762	0.01822	0.3331881
rs12923050	16	47908842	C	T	0.452	0.99	0.155	0.039	0.0000607	0.0000227		0.468	0.99	-0.01763	0.01822	0.3329288
rs2216263	16	47911005	A	T	0.452	0.99	0.155	0.039	0.0000608	0.0000228		0.468	0.99	-0.01761	0.01822	0.3333528
rs152710	16	47912295	C	T	0.452	0.99	0.155	0.039	0.0000609	0.0000229		0.468	0.99	-0.0176	0.01821	0.333482
rs152709	16	47916202	C	T	0.406	1.00	-0.129	0.039	0.0009089	0.0007795		0.418	1.00	-0.00689	0.01844	0.7086262
rs152708	16	47916445	A	G	0.463	1.00	0.139	0.038	0.000303	0.0001191		0.469	1.00	-0.0172	0.01813	0.3422741
rs152707	16	47916622	A	G	0.450	1.00	0.157	0.039	0.0000462	0.0000168		0.466	1.00	-0.01687	0.01813	0.3517544
rs152712	16	47917859	C	T	0.450	1.00	0.156	0.038	0.0000493	0.0000179		0.466	1.00	-0.0168	0.01813	0.3536741
rs152713	16	47918682	C	T	0.450	1.00	0.156	0.038	0.0000494	0.000018		0.466	1.00	-0.01671	0.01812	0.3562309
rs27788	16	47921013	C	T	0.487	1.00	-0.128	0.038	0.0008717	0.0003164		0.480	1.00	0.004577	0.01814	0.8006052
rs27789	16	47921338	C	T	0.594	1.00	0.13	0.039	0.0008322	0.0007136		0.581	1.00	0.00722	0.01847	0.6956064
rs27790	16	47921645	C	T	0.594	1.00	0.13	0.039	0.0008364	0.0007158		0.581	1.00	0.007187	0.01847	0.6969303
rs27791	16	47922017	A	G	0.450	1.00	0.157	0.039	0.0000476	0.0000173		0.466	1.00	-0.01705	0.01813	0.3467177
rs27920	16	47922622	A	T	0.548	0.99	-0.154	0.039	0.0000677	0.0000272		0.533	0.99	0.017842	0.01816	0.3254771
rs27793	16	47923162	A	G	0.450	1.00	0.156	0.039	0.0000523	0.0000186		0.466	1.00	-0.01759	0.01813	0.3314806
rs36507	16	47925689	A	G	0.452	0.99	0.153	0.039	0.0000741	0.0000291		0.467	1.00	-0.01809	0.01816	0.3187518
rs36508	16	47926142	C	T	0.487	1.00	-0.125	0.038	0.0011	0.0003787		0.481	1.00	0.005595	0.01813	0.757412
rs28134	16	47927155	G	T	0.444	0.99	0.118	0.039	0.002318	0.0006705		0.460	0.98	-0.01195	0.01834	0.5143611
rs12595945	16	50291509	C	T	0.186	1.00	-0.164	0.05	0.0009891	0.019263		0.176	1.00	-0.04395	0.02414	0.0684435
rs12598982	16	51094386	C	T	0.559	0.98	-0.103	0.039	0.008879	0.0004053		0.506	0.92	-0.04819	0.01879	0.0103042
rs4784221	16	51095027	A	G	0.809	0.96	-0.186	0.05	0.0002194	0.000014		0.785	0.91	-0.03959	0.02372	0.0949004
rs4784222	16	51095087	C	G	0.441	0.99	0.103	0.039	0.008719	0.0003896		0.494	0.93	0.048265	0.01872	0.0099048
rs9933638	16	51097098	A	G	0.559	1.00	-0.102	0.039	0.008631	0.0003798		0.506	0.93	-0.0483	0.01869	0.0097059
rs9302556	16	51101146	A	C	0.441	1.00	0.102	0.039	0.008911	0.0003927		0.494	0.94	0.048325	0.01866	0.009568
rs7190749	16	51101579	A	G	0.559	1.00	-0.101	0.039	0.009354	0.0004132		0.506	0.94	-0.04836	0.01864	0.009442

					GOYA QC		GOYA Overweight/control			GOYA BMI continuous	known	IARC QC		IARC results		
MARKER	chr	position	A1	A2	FREQ1	Rsqr	Beta	SE	p	p	gene name	freq1	Rsqr	in_beta	in_SE	in_p
rs12443621	16	51105538	A	G	0.560	1.00	-0.101	0.039	0.009664	0.0004279		0.506	0.94	-0.04837	0.01862	0.0093509
rs9933556	16	51105803	C	T	0.559	1.00	-0.101	0.039	0.009593	0.0004227		0.506	0.94	-0.04837	0.0186	0.0092599
rs1362546	16	51107436	C	T	0.559	1.00	-0.101	0.039	0.009521	0.0004176		0.506	0.94	-0.04839	0.01858	0.0091847
rs1075367	16	51112503	A	G	0.441	1.00	0.101	0.039	0.009451	0.0004126		0.494	0.95	0.048426	0.01856	0.0090395
rs8046979	16	51113243	A	G	0.566	0.98	-0.097	0.039	0.0138	0.0005686		0.514	0.93	-0.05028	0.01876	0.0073277
rs1420529	16	51116174	G	T	0.441	1.00	0.101	0.039	0.009348	0.0004052		0.494	0.95	0.04846	0.01851	0.0087936
rs1420531	16	51116335	A	G	0.191	0.98	0.183	0.05	0.0002529	0.0000157		0.215	0.93	0.039886	0.02339	0.0878585
rs11642645	16	51116408	A	C	0.441	1.00	0.102	0.039	0.00921	0.0003956		0.494	0.95	0.048483	0.01849	0.0087087
rs1420533	16	51121127	A	G	0.442	1.00	0.103	0.039	0.008015	0.0003398		0.497	1.00	0.048768	0.01803	0.0068177
rs2193094	16	51123955	G	T	0.442	1.00	0.103	0.039	0.008233	0.0003517		0.496	1.00	0.048733	0.01803	0.0068585
rs11648477	16	51128470	C	T	0.191	0.98	0.183	0.05	0.000254	0.0000147		0.214	0.98	0.040063	0.02287	0.0796122
rs4783780	16	51128937	A	C	0.558	1.00	-0.102	0.039	0.008533	0.0003689		0.504	1.00	-0.04869	0.01803	0.0069124
rs3112581	16	51129034	A	G	0.558	1.00	-0.102	0.039	0.008609	0.000377		0.504	1.00	-0.04867	0.01804	0.0069449
rs3112580	16	51129106	C	T	0.443	1.00	0.102	0.039	0.008646	0.0003812		0.496	1.00	0.048649	0.01804	0.0069695
rs9931232	16	51130333	A	G	0.443	1.00	0.102	0.039	0.0087	0.000387		0.496	1.00	0.048642	0.01804	0.0069798
rs1123428	16	51135336	A	T	0.443	1.00	0.102	0.039	0.008725	0.0003898		0.496	1.00	0.048632	0.01804	0.0069956
rs4594251	16	51155345	C	T	0.807	1.00	-0.178	0.049	0.0003212	0.0000187		0.784	1.00	-0.03964	0.02257	0.078862
rs4784228	16	51158119	A	G	0.807	1.00	-0.177	0.049	0.0003307	0.0000202		0.784	1.00	-0.03946	0.02256	0.0801139
rs3104751	16	51160529	C	T	0.789	1.00	-0.17	0.048	0.0003586	0.0000254		0.762	0.99	-0.04152	0.02179	0.0565338
rs3104753	16	51161412	C	T	0.789	1.00	-0.169	0.048	0.0003716	0.0000272		0.762	0.99	-0.0413	0.02177	0.0577125
rs3481	16	51162597	A	G	0.789	1.00	-0.169	0.048	0.0003748	0.0000276		0.762	0.99	-0.04126	0.02177	0.0578745
rs3104755	16	51163738	A	G	0.789	1.00	-0.169	0.048	0.0003748	0.0000276		0.762	0.99	-0.04113	0.02176	0.0586067
rs3112562	16	51165764	C	G	0.789	1.00	-0.17	0.048	0.0003677	0.0000268		0.762	0.99	-0.04108	0.02178	0.0590378
rs1109951	16	51169545	C	T	0.810	1.00	-0.18	0.05	0.000282	0.0000153		0.786	0.99	-0.03643	0.0227	0.1083003
rs3112638	16	51170120	C	T	0.810	1.00	-0.18	0.05	0.0002815	0.0000152		0.786	0.99	-0.03634	0.0227	0.1091497
rs12935019	16	51170538	A	G	0.190	1.00	0.18	0.05	0.0002812	0.0000152		0.214	1.00	0.03597	0.02269	0.112709
rs3104763	16	51171082	A	G	0.810	1.00	-0.18	0.05	0.000281	0.0000152		0.787	1.00	-0.03584	0.02269	0.1139172
rs4784230	16	51175614	C	T	0.190	1.00	0.18	0.05	0.0002807	0.0000151		0.213	1.00	0.035641	0.02268	0.1158734
rs11645620	16	51176454	C	T	0.809	1.00	-0.183	0.05	0.0002249	0.0000125		0.787	1.00	-0.03564	0.02269	0.1159457
rs3112633	16	51178078	C	T	0.808	1.00	-0.186	0.049	0.0001683	0.00001		0.787	0.99	-0.03561	0.02272	0.1166918
rs11640537	16	51179613	A	G	0.192	1.00	0.186	0.049	0.0001667	0.0000099		0.214	0.99	0.035611	0.02274	0.1170185
rs6498960	16	51181138	C	T	0.838	0.99	-0.192	0.053	0.0002909	0.000062		0.800	0.96	-0.02897	0.02388	0.2246716
rs4262942	16	51181337	G	T	0.162	0.98	0.192	0.053	0.0002893	0.0000614		0.200	0.96	0.028912	0.02391	0.2261156
rs2335	16	51181947	G	T	0.162	0.98	0.192	0.053	0.0002881	0.000061		0.200	0.96	0.028872	0.02392	0.2270177
rs7194368	16	51260247	C	T	0.969	0.99	-0.302	0.112	0.007197	0.00066		0.953	0.99	0.030068	0.04114	0.4645278
rs11859514	16	51285074	C	G	0.968	0.99	-0.306	0.112	0.006184	0.0005333		0.952	0.99	0.029426	0.04094	0.4719302
rs1111474	16	51353441	C	T	0.930	1.00	-0.235	0.076	0.001919	0.0004072		0.924	0.83	0.031781	0.03713	0.3915923
rs12597881	16	52153867	A	G	0.055	0.99	-0.286	0.086	0.0008128	0.001632	FTO					
rs12928335	16	52172247	C	T	0.919	1.00	0.256	0.071	0.0003178	0.0017853	FTO					
rs12444460	16	52194555	C	T	0.945	1.00	0.282	0.086	0.0009618	0.0022178	FTO					
rs3213758	16	52196939	C	T	0.945	1.00	0.282	0.086	0.0009661	0.0022361	FTO					
rs2111119	16	52229255	C	T	0.925	0.99	0.247	0.074	0.0008567	0.0011554	FTO					
rs12445162	16	52307638	A	G	0.067	0.87	-0.283	0.084	0.000717	0.0035545	FTO					
rs7206010	16	52312678	A	G	0.372	0.99	-0.138	0.04	0.0005538	0.0016027	FTO					
rs4386132	16	52316624	C	T	0.944	0.99	0.301	0.086	0.000432	0.0026637	FTO					
rs4280233	16	52322350	G	T	0.944	0.99	0.303	0.086	0.0004139	0.002552	FTO					
rs7203521	16	52326794	A	G	0.628	1.00	0.138	0.04	0.0005335	0.0014473	FTO					
rs6499640	16	52327178	A	G	0.628	1.00	0.138	0.04	0.0005235	0.001416	FTO					
rs8059991	16	52329847	A	G	0.477	0.93	-0.136	0.04	0.0007079	0.0039884	FTO					
rs6499641	16	52330127	A	T	0.523	0.92	0.137	0.04	0.0006746	0.0038246	FTO					
rs4396532	16	52330548	A	G	0.057	0.91	-0.321	0.089	0.0002992	0.0018733	FTO					
rs4784323	16	52355066	A	G	0.333	0.70	-0.242	0.049	8.09E-07	0.00000132	FTO					
rs7206790	16	52355409	C	G	0.521	0.78	-0.268	0.044	8.92E-10	1.44E-09	FTO					
rs8047395	16	52356024	A	G	0.526	0.93	0.25	0.04	5.06E-10	5.26E-10	FTO					
rs9937053	16	52357008	A	G	0.469	0.99	0.29	0.039	1.05E-13	9.95E-15	FTO					
rs9928094	16	52357406	A	G	0.531	1.00	-0.289	0.039	1.06E-13	9.95E-15	FTO					
rs9930333	16	52357478	G	T	0.469	1.00	0.289	0.039	1.06E-13	1E-14	FTO					
rs12446228	16	52357888	A	G	0.364	0.99	-0.217	0.04	7.87E-08	8.05E-08	FTO					
rs9939973	16	52358069	A	G	0.469	1.00	0.289	0.039	1.05E-13	9.87E-15	FTO					
rs9940646	16	52358130	C	G	0.522	0.97	-0.295	0.039	7.51E-14	7.98E-15	FTO					
rs9940128	16	52358255	A	G	0.469	1.00	0.289	0.039	1.04E-13	9.8E-15	FTO					
rs1421085	16	52358455	C	T	0.449	1.00	0.294	0.039	5E-14	4E-15	FTO					
rs9923147	16	52359050	C	T	0.531	1.00	-0.289	0.039	1.03E-13	9.73E-15	FTO					
rs9923544	16	52359486	C	T	0.531	1.00	-0.289	0.039	1.02E-13	9.7E-15	FTO					
rs8055197	16	52360657	A	G	0.536	1.00	0.241	0.039	5.53E-10	7.8E-10	FTO					
rs1558902	16	52361075	A	T	0.449	1.00	0.294	0.039	4.86E-14	3.94E-15	FTO					
rs1861866	16	52361841	C	T	0.464	1.00	-0.241	0.039	5.51E-10	7.8E-10	FTO					
rs10852521	16	52362466	C	T	0.537	1.00	0.241	0.039	5.41E-10	7.79E-10	FTO					
rs11075985	16	52362708	A	C	0.469	1.00	0.289	0.039	1E-13	9.66E-15	FTO					
rs2058908	16	52363646	C	T	0.746	0.74	0.26	0.052	4.46E-07	0.00000133	FTO					
rs9922047	16	52363781	C	G	0.464	1.00	-0.241	0.039	5.77E-10	8.25E-10	FTO					
rs17817288	16	52365265	A	G	0.484	1.00	-0.244	0.039	3.76E-10	4.93E-10	FTO					
rs1477196	16	52365759	A	G	0.364	0.99	-0.216	0.04	8.32E-08	8.85E-08	FTO					
rs1121980	16	52366748	A	G	0.468	1.00	0.289	0.039	1.11E-13	1.03E-14	FTO					
rs7193144	16	52368187	C	T	0.439	1.00	0.286	0.039	2.35E-13	7.98E-15	FTO					
rs8057044	16	52370115	A	G	0.511	1.00	0.234	0.039	1.68E-09	4.2E-10	FTO					
rs17817449	16	52370868	G	T	0.439	1.00	0.284	0.039	3.08E-13	1.16E-14	FTO					
rs8043757	16	52370951	A	T	0.561	1.00	-0.284	0.039	3.16E-13	1.2E-14	FTO					
rs11075987	16	52372662	G	T	0.526	1.00	0.229	0.039	3.4E-09	2.34E-09	FTO					
rs8050136	16	52373776	A	C	0.439	1.00	0.284	0.039	3.49E-13	1.38E-14	FTO					
rs4783819	16	52374148	C	G	0.635	0.99	0.213	0.04	1.21E-07	0.000000133	FTO					
rs8051591	16	52374253	A	G	0.561	1.00	-0.284	0.039	3.57E-13	1.38E-14	FTO					

					GOYA QC		GOYA Overweight/control			GOYA BMI continuous	known	IARC QC		IARC results		
MARKER	chr	position	A1	A2	FREQ1	Rsqr	Beta	SE	p	p	gene name	freq1	Rsqr	in_beta	in_SE	in_p
rs9935401	16	52374339	A	G	0.439	1.00	0.283	0.039	3.71E-13	1.42E-14	FTO					
rs3751812	16	52375961	G	T	0.562	1.00	-0.283	0.039	3.79E-13	1.44E-14	FTO					
rs3751813	16	52376209	G	T	0.433	1.00	-0.251	0.039	1.45E-10	1.31E-11	FTO					
rs9936385	16	52376670	C	T	0.420	0.94	0.299	0.04	1.42E-13	2.79E-15	FTO					
rs9923233	16	52376699	C	G	0.445	0.99	0.284	0.039	4.15E-13	1.32E-14	FTO					
rs11075989	16	52377378	C	T	0.562	1.00	-0.283	0.039	3.79E-13	1.44E-14	FTO					
rs11075990	16	52377394	A	G	0.562	1.00	-0.283	0.039	3.79E-13	1.44E-14	FTO					
rs9939609	16	52378028	A	T	0.438	1.00	0.283	0.039	3.79E-13	1.44E-14	FTO					
rs7202116	16	52379116	A	G	0.562	1.00	-0.283	0.039	3.76E-13	1.43E-14	FTO					
rs7201850	16	52379363	C	T	0.542	1.00	-0.278	0.039	7.39E-13	2.76E-14	FTO					
rs7185735	16	52380152	A	G	0.562	1.00	-0.283	0.039	3.77E-13	1.43E-14	FTO					
rs9941349	16	52382989	C	T	0.542	1.00	-0.278	0.039	7.42E-13	2.77E-14	FTO					
rs9931494	16	52384680	C	G	0.542	1.00	-0.278	0.039	7.8E-13	3.1E-14	FTO					
rs17817964	16	52385567	C	T	0.562	1.00	-0.282	0.039	4.32E-13	1.91E-14	FTO					
rs7190492	16	52386253	A	G	0.367	1.00	-0.212	0.04	1.37E-07	0.000000166	FTO					
rs9930501	16	52387953	A	G	0.529	0.99	-0.269	0.039	5.44E-12	7.25E-13	FTO					
rs9930506	16	52387966	A	G	0.528	0.99	-0.269	0.039	5.98E-12	8.34E-13	FTO					
rs9932754	16	52387992	C	T	0.472	0.99	0.269	0.039	6.02E-12	8.43E-13	FTO					
rs9922708	16	52388647	C	T	0.528	0.99	-0.269	0.039	6.11E-12	8.6E-13	FTO					
rs9922619	16	52389272	G	T	0.528	0.99	-0.268	0.039	6.28E-12	8.93E-13	FTO					
rs8044769	16	52396636	C	T	0.540	1.00	0.223	0.039	1.14E-08	0.000000025	FTO					
rs12149832	16	52400409	A	G	0.453	0.99	0.272	0.039	3.76E-12	6.03E-13	FTO					
rs11642841	16	52402988	A	C	0.447	0.87	0.287	0.042	6.66E-12	1.2E-12	FTO					
rs9935403	16	52404427	A	G	0.023	0.54	-0.505	0.181	0.005423	0.0006983	FTO					
rs1861867	16	52406062	A	G	0.332	0.72	-0.282	0.049	6.84E-09	1.94E-08	FTO					
rs31042	16	53887102	A	G	0.499	1.00	-0.127	0.039	0.0009731	0.002281		0.467	0.99	0.024446	0.01803	0.1749193
rs258586	16	53947024	C	T	0.724	1.00	-0.184	0.044	0.0000277	0.000765		0.691	0.99	-0.0045	0.01993	0.8214148
rs365760	16	53949133	A	T	0.725	0.99	-0.183	0.044	0.0000307	0.0007388		0.698	0.96	-0.00478	0.02032	0.8137494
rs17369468	16	55585266	A	T	0.698	0.80	-0.158	0.048	0.0009516	0.0002863		0.678	0.72	-0.0181	0.0227	0.424809
rs11859012	16	55634021	A	G	0.987	0.67	0.696	0.221	0.001641	0.000207		0.991	0.47	0.045771	0.14349	0.7495281
rs17378109	16	56221459	A	G	0.909	0.98	-0.199	0.068	0.003235	0.0005422		0.905	0.33	-0.04353	0.05652	0.4408288
rs9302698	16	56924898	G	T	0.188	0.76	0.181	0.057	0.001421	0.000388		0.197	0.63	-0.00257	0.02858	0.9282212
rs17241126	16	56971665	C	T	0.936	0.66	-0.263	0.097	0.00671	0.0005153		0.938	0.47	0.081175	0.05275	0.1235722
rs7197453	16	70636628	C	G	0.368	1.00	0.134	0.041	0.0009862	0.0126754		0.346	1.00	0.011651	0.01935	0.546661
rs7197869	16	70636853	C	G	0.368	1.00	0.134	0.041	0.0009752	0.0126217		0.346	1.00	0.011728	0.01935	0.5440467
rs8062041	16	70646465	C	T	0.632	1.00	-0.135	0.041	0.0009216	0.0123973		0.654	1.00	-0.01212	0.01936	0.5309547
rs2550038	16	70669514	C	T	0.410	1.00	0.133	0.04	0.0008434	0.018492		0.419	1.00	0.005297	0.01844	0.7737368
rs2550035	16	70670917	C	G	0.590	1.00	-0.133	0.04	0.0008361	0.0183774		0.581	1.00	-0.00533	0.01844	0.7723244
rs10492813	16	70679964	C	T	0.590	1.00	-0.133	0.04	0.0008029	0.0178666		0.581	1.00	-0.00553	0.01844	0.7642379
rs3852781	16	70681057	A	T	0.410	1.00	0.134	0.04	0.0007969	0.0177813		0.419	1.00	0.005681	0.01844	0.7577938
rs7191127	16	70681493	G	T	0.612	1.00	-0.137	0.04	0.0006531	0.0096134		0.630	1.00	-0.00579	0.01902	0.7607628
rs7190995	16	70681658	A	G	0.612	1.00	-0.137	0.04	0.0006507	0.0095919		0.630	1.00	-0.00584	0.01902	0.7587451
rs2303285	16	70681996	A	C	0.591	1.00	-0.134	0.04	0.0007762	0.0174793		0.581	1.00	-0.00591	0.01843	0.7484265
rs3764311	16	70683118	C	G	0.591	1.00	-0.134	0.04	0.0007704	0.0173945		0.581	1.00	-0.00596	0.01843	0.7462257
rs3764312	16	70683234	A	C	0.612	1.00	-0.138	0.04	0.0006327	0.009444		0.630	1.00	-0.00595	0.01901	0.7539849
rs10492814	16	70684015	A	G	0.409	1.00	0.135	0.04	0.0007224	0.0162113		0.419	1.00	0.00659	0.01843	0.7204144
rs12924413	16	70687246	C	T	0.400	0.99	0.136	0.04	0.0006988	0.0134171		0.415	0.99	0.008409	0.01861	0.6510937
rs1050361	16	70687704	C	G	0.591	1.00	-0.134	0.04	0.0007489	0.0166881		0.581	1.00	-0.00646	0.01844	0.7256129
rs1050362	16	70688316	A	C	0.396	0.95	0.143	0.041	0.0004615	0.0063849		0.386	0.95	0.014555	0.01931	0.4507334
rs2072142	16	70690214	C	T	0.633	1.00	-0.139	0.041	0.0006214	0.0096696		0.653	1.00	-0.01493	0.01938	0.4404944
rs2072141	16	70690712	A	G	0.400	0.98	0.135	0.04	0.0007887	0.0147939		0.415	0.98	0.008187	0.01862	0.6599783
rs12708928	16	70694728	A	C	0.358	0.98	0.14	0.041	0.0006438	0.0082001		0.343	0.98	0.017186	0.01965	0.3813268
rs2240243	16	70695062	A	G	0.358	0.98	0.14	0.041	0.0006509	0.0082594		0.343	0.98	0.017167	0.01965	0.381927
rs12325142	16	70695613	G	T	0.633	1.00	-0.138	0.041	0.0007331	0.0107908		0.653	0.99	-0.01454	0.01943	0.4537816
rs2074626	16	70696685	A	C	0.367	1.00	0.138	0.041	0.0007366	0.0108245		0.347	0.99	0.014536	0.01943	0.4540319
rs8051882	16	70699573	C	T	0.622	0.98	-0.138	0.041	0.0007078	0.0085373		0.634	0.98	-0.00762	0.0193	0.6927971
rs9926156	16	70700396	C	G	0.388	1.00	0.136	0.04	0.0007788	0.01097		0.371	0.99	0.005536	0.01907	0.7714257
rs2241412	16	70701785	A	G	0.612	0.99	-0.135	0.04	0.0008256	0.0113279		0.629	0.99	-0.00549	0.01908	0.7733902
rs6680	16	70704071	C	T	0.388	0.99	0.134	0.04	0.0008661	0.0116304		0.371	0.99	0.005391	0.01908	0.7773551
rs6499560	16	70705167	A	C	0.633	0.99	-0.135	0.041	0.0009029	0.0124107		0.652	0.99	-0.01415	0.01945	0.4664387
rs8047377	16	70705840	C	T	0.388	0.99	0.134	0.04	0.0009192	0.0120194		0.371	0.99	0.005248	0.01909	0.7831797
rs6499561	16	70705905	A	G	0.602	0.97	-0.137	0.041	0.0007884	0.0094652		0.619	0.96	-0.00555	0.01928	0.7734822
rs17605951	16	70855143	A	C	0.823	0.98	-0.174	0.051	0.000703	0.003075		0.848	0.87	-0.02946	0.02702	0.2752811
rs4130513	16	77016251	A	G	0.062	0.83	0.294	0.089	0.0009299	0.0068384		0.067	0.74	0.046465	0.04394	0.2899206
rs2978632	16	77117381	C	T	0.678	0.98	-0.167	0.042	0.0000725	0.0000565		0.670	0.96	-0.00742	0.01968	0.7057984
rs11150084	16	77119321	C	G	0.333	0.98	0.153	0.042	0.0002368	0.0003706		0.366	0.92	-0.00102	0.01983	0.9589114
rs2738499	16	77125917	A	G	0.195	1.00	0.175	0.049	0.0003298	0.002211		0.221	0.96	0.015498	0.02249	0.4903656
rs2667627	16	77125931	C	T	0.187	1.00	0.179	0.05	0.0002984	0.0016706		0.207	0.97	0.015174	0.02291	0.5073417
rs2738502	16	77128042	C	G	0.665	1.00	-0.155	0.041	0.0001855	0.0001627		0.653	0.99	-0.00568	0.01936	0.7689657
rs1106217	16	77129743	C	T	0.338	1.00	0.146	0.041	0.0003959	0.0003002		0.350	0.99	0.003566	0.01933	0.8535034
rs1317575	16	77129756	C	G	0.680	1.00	-0.152	0.042	0.0002867	0.0001109		0.680	0.99	-0.00462	0.01952	0.8127553
rs1105314	16	77130043	A	G	0.320	1.00	0.152	0.042	0.0003021	0.0001255		0.320	0.99	0.004557	0.01951	0.815191
rs2667634	16	77131129	C	G	0.332	0.99	0.153	0.042	0.0002352	0.0001517		0.345	0.98	0.006429	0.01946	0.7408717
rs804889	16	79654040	G	T	0.763	0.98	-0.154	0.046	0.0007321	0.0012331		0.789	0.86	-0.01416	0.02424	0.5587898
rs12929474	16	80174828	A	C	0.063	0.34	0.476	0.138	0.0005458	0.0018593		0.067	0.29	-0.02226	0.06997	0.7501596
rs10514529	16	80701188	A	G	0.981	0.90	0.513	0.154	0.0008533	0.0080682		0.976	0.54	0.073495	0.08172	0.3680517
rs4082806	16	82723121	C	T	0.602	0.97	0.127	0.04	0.001496	0.0006993		0.530	0.96	0.035006	0.01848	0.0580007
rs417190	16	83126602	C	T	0.227	1.00	0.153	0.046	0.0009497	0.0041681		0.198	0.94	-0.00545	0.02333	0.8151523
rs1808568	16	83195530	G	T	0.224	0.59	0.203	0.061	0.0007833	0.0050151		0.202	0.54	-0.00312	0.03022	0.9178144
rs7202346	16	84662164	C	T	0.424	0.98	0.1	0.039	0.01106	0.0003012		0.363	0.49	0.008227	0.02711	0.7613508
rs2241084	16	88602913	C	T	0.856	0.97	0.192	0.056	0.0006554	0.0000758		0.864	0.97	-0.00492	0.02729	0.8568078
rs8059973	16	88607035	A	G	0.150	0.99	-0.166	0.055	0.002481	0.0003044		0.151	0.97	0.004013	0.02623	0.8783194

					GOYA QC		GOYA Overweight/control			GOYA BMI continuous	known	IARC QC		IARC results		
MARKER	chr	position	A1	A2	FREQ1	Rsqr	Beta	SE	p	p	gene name	freq1	Rsqr	in_beta	in_SE	in_p
rs899446	17	4443411	A	G	0.266	1.00	-0.164	0.044	0.0001923	0.0037093		0.258	0.97	0.007354	0.0212	0.7284971
rs899445	17	4443496	G	T	0.232	0.90	-0.173	0.048	0.0003587	0.0054291		0.228	0.80	0.018593	0.02452	0.4479657
rs9908079	17	4443833	G	T	0.718	1.00	0.147	0.043	0.0006655	0.0093567		0.732	0.95	-0.00962	0.02116	0.6488872
rs4790207	17	4449059	G	T	0.348	1.00	-0.143	0.041	0.0004616	0.0021746		0.356	0.86	-0.00477	0.02038	0.8148348
rs2439938	17	5082524	A	G	0.200	1.00	-0.125	0.049	0.01034	0.0008736		0.191	1.00	-0.03428	0.02308	0.137156
rs7213706	17	5992630	C	T	0.935	0.98	0.264	0.08	0.0009539	0.0024757		0.923	0.97	-0.00096	0.03386	0.9774013
rs1860084	17	13950591	A	G	0.836	0.81	-0.18	0.058	0.001788	0.0004631		0.834	0.76	0.027033	0.02979	0.36382
rs12150136	17	14058288	A	G	0.865	0.94	-0.174	0.058	0.002804	0.0006336		0.855	0.92	-0.00511	0.02759	0.852896
rs1984634	17	28991573	C	T	0.861	1.00	0.224	0.056	0.0000573	0.0001333		0.858	0.99	0.031105	0.02608	0.2326428
rs2106883	17	31498590	A	G	0.383	0.43	0.143	0.061	0.01797	0.0008185		0.387	0.32	0.044095	0.03275	0.1777782
rs16966168	17	36134745	A	G	0.129	0.99	0.218	0.058	0.0001767	0.0005144		0.135	0.98	0.02067	0.02758	0.4532619
rs1073839	17	36144260	G	T	0.130	1.00	0.212	0.058	0.000239	0.0006583		0.137	1.00	0.022671	0.02721	0.4042938
rs9303295	17	36144689	A	G	0.870	1.00	-0.211	0.058	0.0002479	0.0006786		0.863	1.00	-0.02344	0.02718	0.3881266
rs1724411	17	41025714	C	T	0.197	0.97	0.16	0.05	0.001379	0.0009877		0.192	0.97	0.052117	0.0246	0.0339784
rs440778	17	41042203	A	G	0.826	0.82	-0.189	0.057	0.0008415	0.0007286		0.834	0.73	-0.05603	0.02963	0.0584295
rs413844	17	41085167	A	G	0.201	0.98	0.162	0.049	0.0009954	0.0009027		0.200	0.97	0.044062	0.02413	0.0676574
rs241030	17	41090286	A	G	0.799	0.98	-0.162	0.049	0.0009833	0.0008899		0.800	0.97	-0.04395	0.02413	0.0683524
rs17688916	17	41134463	A	T	0.179	0.94	0.172	0.053	0.001047	0.0006186		0.172	0.86	0.049373	0.02722	0.0695004
rs1526128	17	41135407	C	T	0.809	0.99	-0.158	0.05	0.001573	0.0009395		0.809	0.95	-0.04682	0.02469	0.0577007
rs1105569	17	41149171	C	T	0.785	0.80	-0.197	0.053	0.0002289	0.0000716		0.778	0.76	-0.05572	0.02596	0.0317434
rs7218457	17	41158289	A	G	0.680	1.00	0.122	0.041	0.00308	0.0006071		0.644	0.91	0.007523	0.02029	0.7105091
rs1358071	17	41158972	A	C	0.758	1.00	-0.15	0.046	0.0009861	0.0019728		0.762	0.95	-0.0324	0.02243	0.1481497
rs1880753	17	41167031	A	G	0.555	1.00	-0.167	0.039	0.000017	0.000057		0.557	1.00	-0.02245	0.0184	0.2220319
rs17563718	17	41167444	C	T	0.806	1.00	-0.158	0.05	0.001441	0.000997		0.808	0.95	-0.05068	0.02472	0.0401854
rs1526125	17	41167948	C	T	0.806	1.00	-0.158	0.05	0.001436	0.0009927		0.808	0.95	-0.05068	0.02472	0.0402155
rs1526126	17	41167989	C	T	0.806	1.00	-0.158	0.05	0.001434	0.0009908		0.808	0.95	-0.05068	0.02472	0.0401927
rs12938476	17	41168668	C	T	0.396	1.00	0.162	0.04	0.0000427	0.0000431		0.397	0.99	0.028935	0.01869	0.1213508
rs12944235	17	41168992	A	G	0.555	1.00	-0.168	0.039	0.0000166	0.0000556		0.557	1.00	-0.02239	0.01841	0.2235556
rs17563787	17	41169023	C	G	0.806	1.00	-0.158	0.05	0.001435	0.0009901		0.808	0.95	-0.05068	0.02472	0.0402366
rs17563800	17	41173230	C	T	0.806	1.00	-0.158	0.05	0.001439	0.000993		0.808	0.95	-0.05068	0.02472	0.0402404
rs17563827	17	41173993	A	C	0.194	1.00	0.158	0.05	0.001441	0.0009945		0.192	0.95	0.050659	0.02472	0.0403169
rs17563861	17	41174677	A	G	0.806	1.00	-0.158	0.05	0.001445	0.000997		0.808	0.95	-0.05066	0.02472	0.0403055
rs17563889	17	41174717	A	T	0.806	1.00	-0.158	0.05	0.001446	0.0009977		0.808	0.95	-0.05067	0.02472	0.0402931
rs4074462	17	41210994	G	T	0.807	0.99	-0.158	0.05	0.001486	0.0009797		0.809	0.96	-0.05023	0.02467	0.0415991
rs4277389	17	41251434	A	G	0.806	1.00	-0.16	0.05	0.001278	0.0009147		0.811	0.99	-0.04728	0.02445	0.053018
rs4566211	17	41251477	A	G	0.194	1.00	0.16	0.05	0.00128	0.0009181		0.189	0.99	0.047245	0.02445	0.0531661
rs17689653	17	41254744	A	T	0.806	1.00	-0.16	0.05	0.001279	0.0009182		0.812	0.99	-0.04724	0.02445	0.0531925
rs1912151	17	41258725	C	T	0.806	1.00	-0.16	0.05	0.00128	0.0009196		0.812	0.99	-0.04718	0.02445	0.0534325
rs1396862	17	41258778	A	G	0.194	1.00	0.16	0.05	0.00128	0.0009196		0.189	0.99	0.047177	0.02445	0.0534655
rs17689824	17	41260178	C	T	0.806	1.00	-0.16	0.05	0.001281	0.0009196		0.812	0.99	-0.04718	0.02445	0.0534437
rs17763086	17	41261262	G	T	0.194	1.00	0.16	0.05	0.001285	0.0009201		0.189	0.99	0.04717	0.02445	0.0535161
rs17689882	17	41262609	A	G	0.194	1.00	0.16	0.05	0.001291	0.0009208		0.189	0.99	0.047172	0.02445	0.0535097
rs1876831	17	41263526	C	T	0.806	1.00	-0.16	0.05	0.001292	0.0009213		0.812	0.99	-0.04717	0.02445	0.0535138
rs16940665	17	41263677	C	T	0.194	1.00	0.16	0.05	0.001292	0.0009214		0.189	0.99	0.047168	0.02445	0.0535331
rs17689918	17	41265869	A	G	0.194	1.00	0.16	0.05	0.001292	0.0009212		0.188	0.99	0.047152	0.02445	0.053629
rs16940674	17	41266288	C	T	0.806	1.00	-0.16	0.05	0.001294	0.000922		0.812	0.99	-0.04715	0.02445	0.0536685
rs1876829	17	41267224	C	T	0.194	1.00	0.16	0.05	0.001296	0.0009222		0.188	0.99	0.047138	0.02445	0.053719
rs878886	17	41268271	C	G	0.806	1.00	-0.16	0.05	0.001297	0.0009228		0.812	0.99	-0.04714	0.02445	0.0537068
rs4640231	17	41268567	C	G	0.194	1.00	0.16	0.05	0.001298	0.0009231		0.188	0.99	0.047139	0.02445	0.0537161
rs10445364	17	41272136	A	G	0.194	1.00	0.16	0.05	0.001313	0.0009251		0.188	0.99	0.04714	0.02445	0.0537155
rs17763533	17	41273970	C	T	0.194	1.00	0.159	0.05	0.001315	0.0009259		0.188	0.99	0.047106	0.02446	0.0539137
rs17690314	17	41275664	G	T	0.194	1.00	0.159	0.05	0.001315	0.0009257		0.188	0.99	0.047107	0.02446	0.0539101
rs17690326	17	41276754	C	T	0.194	1.00	0.159	0.05	0.001315	0.0009241		0.188	0.99	0.047113	0.02446	0.0538859
rs17690703	17	41281077	C	T	0.764	1.00	-0.141	0.046	0.002087	0.0008015		0.776	1.00	-0.02703	0.02261	0.2314025
rs2055794	17	41307507	A	G	0.179	0.89	0.174	0.054	0.001291	0.0006332		0.167	0.86	0.05014	0.02774	0.0704373
rs1864325	17	41333623	C	T	0.808	0.97	-0.163	0.05	0.001232	0.0008329		0.810	0.95	-0.0505	0.02499	0.0431476
rs17563986	17	41347100	A	G	0.807	1.00	-0.159	0.05	0.001377	0.0008702		0.823	0.91	-0.05082	0.02609	0.051305
rs17564153	17	41350926	A	G	0.194	1.00	0.157	0.05	0.001505	0.0009976		0.194	0.96	0.04845	0.02455	0.0482841
rs17649641	17	41353200	C	T	0.195	1.00	0.157	0.05	0.001513	0.0009982		0.194	0.96	0.048466	0.02455	0.0482183
rs17564223	17	41353348	C	T	0.806	1.00	-0.157	0.05	0.001514	0.0009987		0.806	0.96	-0.04847	0.02455	0.0481922
rs17649700	17	41353729	C	G	0.195	1.00	0.157	0.05	0.001515	0.0009991		0.194	0.96	0.048498	0.02455	0.0480799
rs1467969	17	41354156	C	T	0.806	1.00	-0.157	0.05	0.001515	0.0009993		0.806	0.96	-0.04854	0.02455	0.0479131
rs1467970	17	41354402	G	T	0.195	1.00	0.157	0.05	0.001515	0.0009992		0.194	0.96	0.048548	0.02455	0.0478721
rs767058	17	41354595	C	T	0.195	1.00	0.157	0.05	0.001516	0.0009997		0.194	0.96	0.048546	0.02456	0.0478849
rs17650842	17	41393329	A	G	0.804	0.97	-0.158	0.05	0.001527	0.0009767		0.810	0.97	-0.05087	0.02456	0.0381988
rs17650860	17	41394844	A	G	0.196	0.97	0.158	0.05	0.001525	0.0009737		0.190	0.97	0.050876	0.02456	0.0381599
rs17650872	17	41395352	G	T	0.804	0.97	-0.158	0.05	0.00151	0.0009632		0.810	0.97	-0.05089	0.02455	0.0380756
rs17650901	17	41395527	A	G	0.805	0.98	-0.158	0.05	0.001466	0.0009271		0.810	0.97	-0.05092	0.02454	0.0378471
rs17650973	17	41400056	A	T	0.806	0.99	-0.158	0.05	0.00146	0.0009234		0.810	0.97	-0.05092	0.02453	0.037812
rs17650991	17	41400344	A	C	0.780	0.92	-0.2	0.049	0.0000476	0.000029		0.794	0.91	-0.0524	0.02457	0.0328617
rs17572147	17	41400821	A	G	0.806	0.99	-0.158	0.05	0.001446	0.0009123		0.810	0.97	-0.05093	0.02453	0.0377188
rs17572169	17	41401810	C	T	0.806	0.99	-0.158	0.05	0.001445	0.000912		0.810	0.98	-0.05093	0.02452	0.0376922
rs17651093	17	41405386	A	G	0.194	0.99	0.158	0.05	0.001446	0.0009121		0.190	0.98	0.050941	0.02452	0.0375973
rs17572248	17	41405703	A	G	0.806	0.99	-0.158	0.05	0.001445	0.0009112		0.811	0.98	-0.05095	0.02451	0.0375522
rs17651134	17	41406176	A	G	0.194	0.99	0.158	0.05	0.001443	0.0009077		0.189	0.98	0.050943	0.0245	0.0374896
rs1800547	17	41407682	A	G	0.806	0.99	-0.158	0.05	0.001442	0.0009088		0.811	0.98	-0.05095	0.0245	0.0374221
rs17651213	17	41407760	A	G	0.194	1.00	0.158	0.05	0.001437	0.0009042		0.189	0.98	0.050948	0.0245	0.0374137
rs17572361	17	41407845	C	T	0.194	1.00	0.158	0.05	0.001435	0.0009029		0.189	0.98	0.050952	0.0245	0.0373922
rs17651243	17	41408120	A	G	0.194	1.00	0.158	0.05	0.00143	0.0008983		0.189	0.98	0.050961	0.02449	0.0372928
rs2217394	17	41409284	A	G	0.806	1.00	-0.158	0.05	0.00143	0.000899		0.811	0.98	-0.05095	0.02448	0.0372858
rs17651285	17	41410073	A	G	0.194	1.00	0.158	0.05	0.001428	0.0008974		0.189	0.98	0.050968	0.02448	0.037202
rs17572467	17	41410224	C	T	0.194	1.00	0.158	0.05	0.001433	0.0009004		0.189	0.98	0.050974	0.02448	0.0371708

					GOYA QC		GOYA Overweight/control			GOYA BMI continuous	known	IARC QC		IARC results		
MARKER	chr	position	A1	A2	FREQ1	Rsqr	Beta	SE	p	p	gene name	freq1	Rsqr	in_beta	in_SE	in_p
rs17572495	17	41410432	G	T	0.194	1.00	0.158	0.05	0.001449	0.0009087		0.189	0.99	0.051118	0.02447	0.0365961
rs754512	17	41411483	A	T	0.807	1.00	-0.158	0.05	0.001455	0.0009123		0.813	1.00	-0.0517	0.02445	0.0343397
rs1981997	17	41412603	A	G	0.193	1.00	0.158	0.05	0.001458	0.0009144		0.187	1.00	0.051727	0.02445	0.034258
rs1981998	17	41412669	A	G	0.194	1.00	0.157	0.05	0.001529	0.0009836		0.187	1.00	0.051703	0.02445	0.0343403
rs17573607	17	41438918	A	G	0.157	0.82	0.194	0.059	0.001132	0.0008151		0.156	0.82	0.065373	0.0287	0.0226394
rs1991556	17	41439239	A	G	0.203	0.96	0.163	0.05	0.001047	0.0007031		0.198	0.94	0.048711	0.02464	0.0479302
rs8067056	17	41439785	C	T	0.362	0.95	0.172	0.042	0.0000433	0.00000831		0.357	0.93	0.013507	0.02001	0.4993559
rs17652449	17	41444774	C	G	0.194	1.00	0.158	0.05	0.00143	0.0009585		0.189	0.99	0.051097	0.0245	0.0368567
rs733966	17	41445400	C	T	0.806	1.00	-0.158	0.05	0.001418	0.0009503		0.811	0.99	-0.0511	0.0245	0.0368537
rs12150506	17	41446373	A	G	0.194	1.00	0.158	0.05	0.001417	0.0009493		0.189	0.99	0.051113	0.0245	0.0368141
rs12150170	17	41446483	A	G	0.806	1.00	-0.158	0.05	0.001394	0.0009335		0.811	0.99	-0.05111	0.0245	0.0368113
rs17573858	17	41446633	C	T	0.194	1.00	0.159	0.05	0.001348	0.0009033		0.189	0.99	0.051117	0.0245	0.0368027
rs17652502	17	41450308	A	G	0.194	1.00	0.159	0.05	0.001347	0.0009023		0.189	0.99	0.051118	0.0245	0.0367994
rs9468	17	41457408	C	T	0.194	1.00	0.159	0.05	0.001317	0.0008815		0.191	0.98	0.050998	0.02446	0.0369794
rs1052587	17	41458449	C	T	0.194	1.00	0.159	0.05	0.001317	0.000881		0.191	0.98	0.050985	0.02446	0.0370076
rs1052594	17	41458535	C	G	0.194	1.00	0.159	0.05	0.001315	0.0008802		0.191	0.98	0.050984	0.02446	0.0370072
rs17574040	17	41458711	A	C	0.806	1.00	-0.159	0.05	0.001313	0.0008785		0.809	0.98	-0.05098	0.02446	0.0370166
rs16940799	17	41458779	C	T	0.194	1.00	0.159	0.05	0.001312	0.0008781		0.191	0.98	0.050971	0.02446	0.0370422
rs7687	17	41459142	C	T	0.194	1.00	0.159	0.05	0.00131	0.0008765		0.191	0.98	0.050968	0.02446	0.0370483
rs17652748	17	41459462	C	T	0.806	1.00	-0.159	0.05	0.001308	0.0008756		0.809	0.98	-0.05098	0.02446	0.0370135
rs2158257	17	41460189	A	C	0.816	0.94	-0.17	0.052	0.001119	0.0006515		0.814	0.96	-0.05238	0.02506	0.0364962
rs17574228	17	41460355	C	T	0.194	1.00	0.159	0.05	0.001305	0.0008731		0.191	0.98	0.050938	0.02445	0.0371101
rs7350928	17	41463947	C	T	0.806	1.00	-0.159	0.05	0.001298	0.0008685		0.809	0.98	-0.05094	0.02445	0.0370986
rs17574361	17	41464049	A	G	0.806	1.00	-0.159	0.05	0.001297	0.0008677		0.809	0.98	-0.05094	0.02445	0.0370881
rs17652961	17	41464202	A	G	0.194	1.00	0.159	0.05	0.001296	0.0008664		0.191	0.98	0.050937	0.02445	0.0370853
rs17574425	17	41465035	C	G	0.806	1.00	-0.16	0.05	0.001294	0.0008654		0.809	0.98	-0.05093	0.02445	0.0370905
rs1076222	17	41465616	C	G	0.806	1.00	-0.16	0.05	0.001292	0.0008642		0.809	0.98	-0.05094	0.02445	0.0370718
rs7350980	17	41466118	A	G	0.199	1.00	0.158	0.049	0.001289	0.0009474		0.194	0.98	0.053122	0.02437	0.0291965
rs4597358	17	41466517	C	G	0.801	1.00	-0.158	0.049	0.001288	0.0009466		0.806	0.98	-0.05314	0.02437	0.0291477
rs17574604	17	41467460	A	G	0.806	1.00	-0.16	0.05	0.00129	0.0008628		0.809	0.98	-0.05097	0.02445	0.0369618
rs17653162	17	41467674	A	C	0.194	1.00	0.16	0.05	0.00129	0.0008623		0.191	0.98	0.050983	0.02445	0.0369154
rs17653193	17	41468288	A	G	0.184	0.93	0.168	0.052	0.001228	0.0005864		0.181	0.90	0.056189	0.02608	0.031101
rs17653211	17	41468485	C	T	0.194	1.00	0.16	0.05	0.001289	0.0008616		0.191	0.98	0.051	0.02445	0.0368567
rs17653255	17	41468590	A	C	0.187	0.98	0.161	0.05	0.001427	0.000719		0.182	0.95	0.052796	0.0254	0.0375044
rs17574796	17	41470921	C	T	0.194	1.00	0.16	0.05	0.001285	0.0008588		0.191	0.98	0.05102	0.02445	0.0367981
rs17574824	17	41470954	C	T	0.806	1.00	-0.16	0.05	0.001284	0.000858		0.809	0.98	-0.05102	0.02445	0.0367988
rs12185243	17	41471198	C	T	0.191	0.95	0.167	0.051	0.001107	0.000712		0.190	0.93	0.054203	0.02509	0.0306373
rs8077487	17	41471287	C	T	0.199	1.00	0.158	0.049	0.001283	0.0009423		0.194	0.98	0.053253	0.02438	0.0288263
rs11079729	17	41471416	A	C	0.194	1.00	0.16	0.05	0.001283	0.0008567		0.191	0.98	0.051032	0.02445	0.0367666
rs12150542	17	41471577	A	G	0.194	1.00	0.16	0.05	0.001282	0.000856		0.191	0.98	0.051043	0.02445	0.0367271
rs12150090	17	41471733	C	T	0.806	1.00	-0.16	0.05	0.001281	0.0008553		0.809	0.98	-0.05106	0.02445	0.0366785
rs7221390	17	41472797	G	T	0.199	1.00	0.158	0.049	0.001282	0.0009409		0.194	0.98	0.053309	0.02438	0.0286693
rs10514897	17	41473244	A	G	0.806	1.00	-0.16	0.05	0.00128	0.0008547		0.809	0.98	-0.05107	0.02445	0.0366485
rs10514898	17	41475485	A	C	0.806	1.00	-0.16	0.05	0.00128	0.000854		0.809	0.98	-0.05111	0.02446	0.0365163
rs7218319	17	41482217	C	T	0.801	1.00	-0.158	0.049	0.001282	0.0009398		0.806	0.98	-0.05344	0.02438	0.0283141
rs12150447	17	41483977	A	C	0.806	1.00	-0.16	0.05	0.00128	0.0008533		0.809	0.98	-0.05116	0.02446	0.0363579
rs12150064	17	41484259	A	C	0.194	1.00	0.16	0.05	0.001279	0.0008526		0.191	0.98	0.051164	0.02446	0.0363344
rs17653836	17	41484728	A	T	0.194	1.00	0.16	0.05	0.001279	0.0008517		0.191	0.98	0.051179	0.02446	0.0362833
rs17575423	17	41485105	C	T	0.194	1.00	0.16	0.05	0.001278	0.0008512		0.191	0.98	0.051181	0.02446	0.0362782
rs17575437	17	41488867	A	T	0.194	1.00	0.16	0.05	0.001278	0.0008499		0.191	0.98	0.051214	0.02446	0.0361709
rs17653889	17	41488906	A	T	0.806	1.00	-0.16	0.05	0.001277	0.0008497		0.809	0.98	-0.05127	0.02447	0.0359925
rs17653906	17	41488978	A	G	0.194	1.00	0.16	0.05	0.001277	0.0008498		0.191	0.98	0.051285	0.02447	0.0359471
rs17575507	17	41489931	A	G	0.806	1.00	-0.16	0.05	0.001277	0.0008496		0.809	0.98	-0.05133	0.02447	0.0357886
rs876944	17	41490227	G	T	0.806	1.00	-0.16	0.05	0.001277	0.0008492		0.809	0.98	-0.05134	0.02447	0.035772
rs1107820	17	41491195	C	T	0.194	1.00	0.16	0.05	0.001277	0.0008488		0.191	0.98	0.051373	0.02447	0.0356759
rs17575556	17	41491663	A	G	0.194	1.00	0.16	0.05	0.001277	0.0008493		0.191	0.98	0.051394	0.02447	0.0356153
rs17653998	17	41491821	A	G	0.806	1.00	-0.16	0.05	0.001277	0.0008488		0.809	0.98	-0.05142	0.02448	0.0355267
rs17654016	17	41493743	C	T	0.198	0.97	0.159	0.05	0.001419	0.0008883		0.204	0.91	0.05298	0.02472	0.0319804
rs2316951	17	41494901	C	G	0.194	1.00	0.16	0.05	0.001277	0.0008482		0.191	0.98	0.051518	0.02448	0.0352309
rs17575683	17	41495480	C	T	0.806	1.00	-0.16	0.05	0.001278	0.0008484		0.809	0.98	-0.05155	0.02448	0.0351371
rs2838	17	41497167	A	G	0.806	1.00	-0.16	0.05	0.001276	0.0008474		0.809	0.98	-0.05157	0.02449	0.0350651
rs17575850	17	41500209	A	C	0.194	1.00	0.16	0.05	0.001274	0.0008467		0.191	0.98	0.05157	0.0245	0.035182
rs17659731	17	41500456	A	G	0.194	1.00	0.16	0.05	0.001273	0.0008459		0.191	0.98	0.051588	0.02451	0.0351573
rs1117253	17	41505119	A	C	0.806	1.00	-0.16	0.05	0.00127	0.0008445		0.809	0.98	-0.05158	0.02451	0.0351921
rs974293	17	41506055	A	G	0.194	1.00	0.16	0.05	0.00127	0.0008441		0.191	0.98	0.051598	0.02451	0.0351589
rs2066899	17	41511550	C	T	0.802	1.00	-0.158	0.049	0.001291	0.0009381		0.806	0.98	-0.05392	0.02445	0.0273411
rs2316954	17	41511986	A	G	0.194	1.00	0.16	0.05	0.001265	0.0008419		0.191	0.98	0.051639	0.02453	0.0351579
rs17659881	17	41513416	A	G	0.806	1.00	-0.16	0.05	0.001265	0.000842		0.809	0.98	-0.05163	0.02453	0.035195
rs1476554	17	41514921	C	T	0.806	1.00	-0.16	0.05	0.001264	0.0008411		0.809	0.98	-0.05164	0.02454	0.0351948
rs17659953	17	41515544	C	T	0.806	1.00	-0.16	0.05	0.00126	0.0008393		0.809	0.98	-0.05162	0.02454	0.035291
rs17660017	17	41516493	C	G	0.193	1.00	0.16	0.05	0.00129	0.0008517		0.188	0.96	0.050924	0.02495	0.0410943
rs17660065	17	41518102	C	T	0.194	1.00	0.16	0.05	0.001243	0.0008312		0.191	0.97	0.051754	0.02461	0.0353284
rs8080583	17	41518415	A	C	0.198	1.00	0.158	0.049	0.001306	0.0009397		0.194	0.97	0.054089	0.02454	0.027399
rs3912061	17	41520916	C	T	0.806	1.00	-0.16	0.05	0.00124	0.0008296		0.809	0.97	-0.05177	0.02462	0.0353411
rs17660132	17	41521621	C	T	0.194	1.00	0.16	0.05	0.001239	0.000829		0.191	0.97	0.051779	0.02462	0.035362
rs17660167	17	41522129	C	T	0.806	1.00	-0.16	0.05	0.001238	0.0008286		0.809	0.97	-0.05177	0.02463	0.0354016
rs17660228	17	41522318	A	G	0.194	1.00	0.16	0.05	0.001237	0.0008282		0.191	0.97	0.051783	0.02463	0.0353891
rs17660251	17	41522464	C	T	0.806	1.00	-0.16	0.05	0.001236	0.0008273		0.809	0.97	-0.05178	0.02463	0.0354161
rs17660294	17	41522919	C	T	0.194	1.00	0.16	0.05	0.001235	0.0008267		0.191	0.97	0.051792	0.02464	0.0354132
rs17660337	17	41525423	C	T	0.194	1.00	0.16	0.05	0.001235	0.0008268		0.191	0.97	0.051794	0.02464	0.0354151
rs17576631	17	41525836	C	G	0.194	1.00	0.16	0.05	0.001233	0.0008259		0.191	0.97	0.0518	0.02464	0.0354215
rs17660398	17	41526429	C	T	0.807	1.00	-0.16	0.05	0.001232	0.0008256		0.809	0.97	-0.0518	0.02464	0.0354298

					GOYA QC		GOYA Overweight/control			GOYA BMI continuous	known	IARC QC		IARC results		
MARKER	chr	position	A1	A2	FREQ1	Rsqr	Beta	SE	p	p	gene name	freq1	Rsqr	in_beta	in_SE	in_p
rs17576695	17	41528144	A	T	0.194	1.00	0.16	0.05	0.001231	0.0008252		0.191	0.97	0.051804	0.02464	0.0354259
rs17576709	17	41529173	C	G	0.807	1.00	-0.16	0.05	0.00123	0.0008245		0.809	0.97	-0.0518	0.02465	0.0354606
rs17660464	17	41533806	A	C	0.194	1.00	0.16	0.05	0.001229	0.0008243		0.191	0.97	0.051809	0.02465	0.035477
rs17576779	17	41534085	C	T	0.807	1.00	-0.16	0.05	0.001228	0.0008233		0.809	0.97	-0.05182	0.02466	0.0354437
rs17660488	17	41534150	C	T	0.194	1.00	0.16	0.05	0.001226	0.0008222		0.191	0.97	0.051831	0.02466	0.0354232
rs17576842	17	41535804	A	G	0.807	1.00	-0.16	0.05	0.001222	0.0008204		0.809	0.97	-0.05183	0.02466	0.0354616
rs17576870	17	41536254	A	G	0.807	1.00	-0.16	0.05	0.001221	0.0008198		0.809	0.97	-0.05182	0.02466	0.0354899
rs17660595	17	41536766	A	G	0.807	1.00	-0.16	0.05	0.00122	0.000819		0.809	0.97	-0.05183	0.02466	0.0354971
rs17576954	17	41537745	C	T	0.194	1.00	0.16	0.05	0.001217	0.0008175		0.191	0.97	0.051834	0.02467	0.0354805
rs17576989	17	41539399	A	C	0.194	1.00	0.16	0.05	0.001214	0.000816		0.191	0.96	0.051856	0.02467	0.0354385
rs17577024	17	41542035	A	G	0.194	1.00	0.161	0.05	0.00121	0.0008136		0.191	0.96	0.051868	0.02468	0.0354865
rs17577052	17	41542084	C	T	0.194	1.00	0.161	0.05	0.001206	0.0008116		0.191	0.96	0.051882	0.02469	0.0354653
rs9303525	17	41543040	A	G	0.802	1.00	-0.158	0.049	0.001306	0.0009312		0.806	0.96	-0.05419	0.02462	0.0276354
rs17577094	17	41543275	A	G	0.807	1.00	-0.161	0.05	0.0012	0.0008082		0.809	0.96	-0.05187	0.02469	0.0355475
rs17577159	17	41544260	G	T	0.194	1.00	0.161	0.05	0.0012	0.0008079		0.191	0.96	0.051894	0.02469	0.0354834
rs17660847	17	41545156	C	T	0.807	1.00	-0.161	0.05	0.001228	0.0008202		0.819	0.91	-0.0518	0.02602	0.0463662
rs17660865	17	41545191	C	T	0.194	1.00	0.161	0.05	0.001198	0.000808		0.191	0.96	0.0519	0.0247	0.0354967
rs17660907	17	41546868	A	G	0.194	1.00	0.161	0.05	0.001199	0.0008083		0.191	0.96	0.051908	0.0247	0.035478
rs17660936	17	41546942	C	T	0.807	1.00	-0.161	0.05	0.001198	0.0008086		0.809	0.96	-0.05191	0.0247	0.0354999
rs17577313	17	41547612	A	G	0.807	1.00	-0.161	0.05	0.001199	0.0008093		0.809	0.96	-0.0519	0.0247	0.0355352
rs4548919	17	41548172	G	T	0.807	1.00	-0.161	0.05	0.001199	0.0008099		0.809	0.96	-0.0519	0.02471	0.0355386
rs4630591	17	41548345	C	T	0.807	1.00	-0.161	0.05	0.001201	0.0008106		0.816	0.92	-0.05273	0.02554	0.0387864
rs17577369	17	41548702	A	G	0.807	1.00	-0.161	0.05	0.001199	0.0008109		0.809	0.96	-0.0519	0.02471	0.0355763
rs17661015	17	41548736	C	T	0.194	1.00	0.161	0.05	0.0012	0.0008114		0.191	0.96	0.051897	0.02471	0.0355921
rs17661027	17	41548876	A	C	0.194	1.00	0.161	0.05	0.0012	0.0008117		0.191	0.96	0.051898	0.02471	0.035599
rs17661045	17	41549931	A	T	0.194	1.00	0.161	0.05	0.0012	0.0008121		0.191	0.96	0.051885	0.02471	0.035651
rs17577447	17	41550160	A	T	0.194	1.00	0.161	0.05	0.0012	0.0008129		0.191	0.96	0.0519	0.02471	0.0356072
rs10514901	17	41550514	G	T	0.807	1.00	-0.161	0.05	0.001201	0.000814		0.809	0.96	-0.05189	0.02472	0.0356464
rs17577496	17	41550614	C	T	0.194	1.00	0.161	0.05	0.001201	0.0008142		0.191	0.96	0.051896	0.02472	0.0356533
rs17661141	17	41551794	C	G	0.806	1.00	-0.161	0.05	0.001202	0.0008149		0.809	0.96	-0.0519	0.02472	0.0356686
rs1468241	17	41551932	A	G	0.806	1.00	-0.161	0.05	0.001202	0.0008155		0.809	0.96	-0.05189	0.02472	0.0357249
rs4383188	17	41553381	C	T	0.194	1.00	0.161	0.05	0.001202	0.000816		0.191	0.96	0.051885	0.02473	0.035746
rs17577650	17	41561278	A	G	0.806	1.00	-0.161	0.05	0.001203	0.0008172		0.809	0.96	-0.0519	0.02473	0.0357429
rs4471723	17	41561468	C	T	0.806	1.00	-0.161	0.05	0.001203	0.0008181		0.809	0.96	-0.05191	0.02474	0.0357272
rs1122381	17	41561617	A	G	0.806	1.00	-0.161	0.05	0.001204	0.0008187		0.809	0.96	-0.05192	0.02474	0.0357221
rs1122380	17	41561857	C	T	0.806	1.00	-0.161	0.05	0.001204	0.0008196		0.809	0.96	-0.05194	0.02474	0.0356778
rs17661348	17	41562844	A	G	0.806	1.00	-0.161	0.05	0.001206	0.0008221		0.809	0.96	-0.05195	0.02475	0.0357073
rs17661385	17	41563555	A	G	0.806	1.00	-0.161	0.05	0.001206	0.0008223		0.809	0.96	-0.05194	0.02476	0.0357754
rs10514904	17	41563664	C	T	0.806	1.00	-0.161	0.05	0.001206	0.0008228		0.809	0.96	-0.05193	0.02476	0.0358299
rs17661428	17	41563921	C	G	0.806	1.00	-0.161	0.05	0.001207	0.0008232		0.809	0.96	-0.05193	0.02476	0.0358802
rs17577877	17	41563995	A	G	0.806	1.00	-0.161	0.05	0.001207	0.0008239		0.809	0.96	-0.05193	0.02477	0.0359042
rs6503457	17	41564089	G	T	0.802	1.00	-0.158	0.049	0.00133	0.0009614		0.806	0.95	-0.05431	0.0247	0.0278175
rs8070942	17	41564451	G	T	0.802	1.00	-0.158	0.049	0.001331	0.0009622		0.806	0.95	-0.05433	0.02471	0.0278048
rs17577954	17	41565405	C	T	0.194	1.00	0.161	0.05	0.001208	0.0008256		0.191	0.95	0.051955	0.02482	0.0361734
rs17577975	17	41566219	C	T	0.806	1.00	-0.161	0.05	0.001208	0.0008258		0.809	0.95	-0.05196	0.02482	0.036193
rs7207582	17	41566710	A	G	0.802	1.00	-0.158	0.049	0.001332	0.0009662		0.806	0.95	-0.05437	0.02476	0.0280134
rs12150087	17	41566765	C	G	0.806	1.00	-0.161	0.05	0.00121	0.0008278		0.809	0.95	-0.05197	0.02483	0.0362233
rs10221243	17	41568087	A	G	0.198	1.00	0.158	0.049	0.001333	0.000968		0.194	0.95	0.054369	0.02478	0.0280973
rs9915547	17	41568559	C	T	0.198	1.00	0.158	0.049	0.001334	0.0009691		0.194	0.95	0.054373	0.02478	0.0281051
rs12150320	17	41568981	C	T	0.806	1.00	-0.161	0.05	0.001211	0.0008296		0.809	0.95	-0.05198	0.02485	0.0363138
rs2532316	17	41569489	A	G	0.194	1.00	0.161	0.05	0.001212	0.0008308		0.191	0.95	0.051979	0.02485	0.0363577
rs2696605	17	41569698	A	C	0.806	1.00	-0.161	0.05	0.001212	0.0008313		0.809	0.95	-0.05197	0.02486	0.036411
rs2532315	17	41569711	A	G	0.194	1.00	0.161	0.05	0.001213	0.0008317		0.191	0.95	0.051982	0.02486	0.0364367
rs2696604	17	41569871	A	G	0.806	1.00	-0.161	0.05	0.001213	0.0008324		0.809	0.95	-0.05199	0.02487	0.0364476
rs2077551	17	41570665	C	T	0.194	1.00	0.16	0.05	0.001215	0.0008335		0.178	0.88	0.056908	0.0265	0.0316746
rs2696602	17	41571673	C	T	0.806	1.00	-0.161	0.05	0.001214	0.0008334		0.809	0.95	-0.05198	0.02488	0.0365731
rs2696601	17	41571935	C	T	0.194	1.00	0.161	0.05	0.001214	0.0008337		0.191	0.95	0.051979	0.02488	0.0365862
rs2696600	17	41572003	A	G	0.806	1.00	-0.161	0.05	0.001214	0.0008344		0.809	0.95	-0.05196	0.02489	0.0367013
rs2532314	17	41572889	G	T	0.806	1.00	-0.16	0.05	0.001215	0.0008354		0.809	0.95	-0.05198	0.0249	0.0366683
rs2532313	17	41573070	A	G	0.194	1.00	0.16	0.05	0.001216	0.0008363		0.191	0.95	0.051996	0.0249	0.0366382
rs1918801	17	41573821	A	T	0.806	1.00	-0.16	0.05	0.001217	0.0008375		0.809	0.94	-0.052	0.0249	0.0366864
rs1918800	17	41573915	C	T	0.806	1.00	-0.16	0.05	0.001217	0.0008379		0.809	0.94	-0.05199	0.02491	0.0367394
rs1918799	17	41574019	C	T	0.806	1.00	-0.16	0.05	0.001218	0.000839		0.809	0.94	-0.052	0.02491	0.0367237
rs1528075	17	41576231	G	T	0.194	1.00	0.16	0.05	0.001219	0.0008401		0.191	0.94	0.051987	0.02495	0.0370683
rs2532307	17	41577127	A	G	0.194	1.00	0.16	0.05	0.00122	0.0008417		0.191	0.94	0.051994	0.02496	0.0371064
rs2696592	17	41577253	A	T	0.806	1.00	-0.16	0.05	0.00122	0.0008422		0.810	0.94	-0.05199	0.02496	0.0371548
rs2696590	17	41577379	C	G	0.193	0.99	0.161	0.05	0.00125	0.0008557		0.181	0.89	0.051822	0.02631	0.0487553
rs2222746	17	41577796	G	T	0.806	1.00	-0.16	0.05	0.001222	0.0008443		0.810	0.94	-0.05199	0.02498	0.0372435
rs1406074	17	41577844	G	T	0.194	1.00	0.16	0.05	0.001223	0.0008448		0.191	0.94	0.052012	0.02498	0.0371985
rs1918798	17	41578112	A	G	0.194	1.00	0.16	0.05	0.001224	0.000847		0.190	0.94	0.051995	0.02502	0.0375348
rs2696587	17	41578237	G	T	0.194	1.00	0.16	0.05	0.001225	0.000848		0.190	0.94	0.052002	0.02502	0.0375507
rs2532305	17	41578837	A	G	0.194	1.00	0.16	0.05	0.001226	0.0008488		0.190	0.94	0.052	0.02502	0.0375763
rs1918797	17	41579020	A	G	0.194	1.00	0.16	0.05	0.001226	0.0008497		0.190	0.93	0.052	0.02503	0.0376015
rs1406069	17	41581663	A	G	0.194	1.00	0.16	0.05	0.001228	0.0008511		0.178	0.87	0.056899	0.02668	0.0328168
rs2696581	17	41582963	A	G	0.194	1.00	0.16	0.05	0.001228	0.0008515		0.190	0.93	0.051966	0.02505	0.0378667
rs17585012	17	41583287	A	T	0.806	1.00	-0.16	0.05	0.001228	0.000852		0.810	0.93	-0.05197	0.02505	0.0378509
rs17662115	17	41583308	C	T	0.194	1.00	0.16	0.05	0.00123	0.0008546		0.190	0.93	0.05196	0.02505	0.0379186
rs2696577	17	41583659	C	G	0.806	1.00	-0.16	0.05	0.00123	0.0008547		0.822	0.87	-0.05691	0.02669	0.0328749
rs1918792	17	41584306	A	G	0.194	1.00	0.16	0.05	0.001255	0.0008658		0.190	0.86	0.052249	0.02607	0.0449347
rs1918791	17	41584386	C	G	0.806	1.00	-0.16	0.05	0.001255	0.0008663		0.810	0.86	-0.05224	0.02608	0.0449813
rs1918790	17	41584396	C	T	0.806	1.00	-0.16	0.05	0.001256	0.0008675		0.810	0.86	-0.05226	0.02608	0.0449714
rs2696576	17	41584547	A	G	0.806	1.00	-0.16	0.05	0.001256	0.0008678		0.810	0.86	-0.05226	0.02609	0.0450096

					GOYA QC		GOYA Overweight/control			GOYA BMI continuous	known	IARC QC		IARC results		
MARKER	chr	position	A1	A2	FREQ1	Rsqr	Beta	SE	p	p	gene name	freq1	Rsqr	in_beta	in_SE	in_p
rs2696575	17	41584601	A	G	0.806	1.00	-0.16	0.05	0.001256	0.0008682		0.810	0.86	-0.05226	0.02609	0.0450069
rs2532303	17	41585141	C	T	0.194	1.00	0.16	0.05	0.001257	0.000869		0.190	0.86	0.052263	0.02609	0.0450248
rs2532302	17	41585192	C	T	0.194	1.00	0.16	0.05	0.001258	0.0008704		0.190	0.86	0.052261	0.0261	0.0450611
rs2696574	17	41585874	C	T	0.806	1.00	-0.16	0.05	0.001258	0.0008707		0.822	0.80	-0.05732	0.02788	0.0396715
rs2696573	17	41586424	C	T	0.806	1.00	-0.16	0.05	0.001258	0.0008718		0.810	0.86	-0.05226	0.02611	0.045207
rs2532298	17	41587072	A	G	0.194	1.00	0.16	0.05	0.001258	0.0008721		0.190	0.86	0.052279	0.02612	0.0451799
rs2532297	17	41587103	A	G	0.194	1.00	0.16	0.05	0.00126	0.000874		0.190	0.86	0.052273	0.02613	0.0452642
rs2696572	17	41587394	A	T	0.806	1.00	-0.16	0.05	0.001261	0.0008746		0.810	0.86	-0.05228	0.02613	0.045276
rs2532296	17	41587604	C	T	0.194	1.00	0.16	0.05	0.001261	0.000875		0.190	0.86	0.052285	0.02614	0.0453128
rs2109092	17	41588736	A	G	0.194	1.00	0.16	0.05	0.001263	0.0008767		0.190	0.86	0.052285	0.02614	0.0453445
rs1534456	17	41588767	C	T	0.187	0.99	0.161	0.05	0.001402	0.0007307		0.181	0.84	0.053488	0.02712	0.04839
rs17662235	17	41589553	C	T	0.194	1.00	0.16	0.05	0.001264	0.0008776		0.190	0.85	0.052272	0.02626	0.0464202
rs17585214	17	41589588	C	T	0.806	1.00	-0.16	0.05	0.001264	0.0008781		0.810	0.85	-0.05226	0.02627	0.046498
rs1528074	17	41589837	A	G	0.813	0.99	-0.161	0.05	0.001403	0.000732		0.819	0.83	-0.05347	0.02713	0.0486074
rs1406068	17	41590303	C	T	0.806	1.00	-0.16	0.05	0.001265	0.0008805		0.810	0.85	-0.05223	0.02629	0.0468185
rs1528072	17	41592502	A	C	0.194	1.00	0.16	0.05	0.001267	0.0008822		0.190	0.85	0.052202	0.0263	0.0469731
rs2532292	17	41592845	A	T	0.806	1.00	-0.16	0.05	0.001268	0.0008831		0.810	0.85	-0.05221	0.02631	0.0470106
rs2696571	17	41593149	C	G	0.194	1.00	0.16	0.05	0.001268	0.0008841		0.190	0.85	0.052227	0.02631	0.0469818
rs2532291	17	41594201	A	G	0.194	1.00	0.16	0.05	0.00127	0.0008864		0.190	0.85	0.052235	0.02632	0.0470321
rs2532290	17	41594267	A	G	0.194	1.00	0.16	0.05	0.00127	0.0008867		0.190	0.85	0.052256	0.02632	0.0469726
rs17662403	17	41594715	C	T	0.194	1.00	0.16	0.05	0.001271	0.0008879		0.190	0.85	0.052249	0.02632	0.047017
rs17585426	17	41594743	C	T	0.194	1.00	0.16	0.05	0.001272	0.0008894		0.190	0.85	0.052247	0.02633	0.0470368
rs2532288	17	41595735	C	T	0.806	1.00	-0.16	0.05	0.001273	0.0008902		0.810	0.85	-0.05226	0.02633	0.0470111
rs1918789	17	41595884	C	T	0.194	1.00	0.16	0.05	0.001273	0.0008907		0.190	0.85	0.052254	0.02633	0.0470338
rs2141299	17	41596763	A	C	0.194	1.00	0.16	0.05	0.001274	0.000892		0.190	0.85	0.052247	0.02633	0.0471065
rs2696567	17	41597081	C	G	0.806	1.00	-0.16	0.05	0.001275	0.0008926		0.810	0.85	-0.05223	0.02634	0.0472248
rs2532286	17	41597441	C	T	0.194	1.00	0.16	0.05	0.001278	0.0008967		0.190	0.84	0.052212	0.02635	0.0473499
rs4792843	17	41598956	A	G	0.806	1.00	-0.16	0.05	0.001278	0.0008972		0.810	0.84	-0.0522	0.02635	0.04747
rs17585608	17	41599756	C	T	0.806	1.00	-0.16	0.05	0.001278	0.0008975		0.810	0.84	-0.05221	0.02636	0.047487
rs2696684	17	41600174	A	G	0.194	1.00	0.16	0.05	0.001279	0.0008983		0.190	0.84	0.052226	0.02636	0.0474475
rs17585644	17	41600358	C	T	0.194	1.00	0.16	0.05	0.001279	0.0008988		0.190	0.84	0.052194	0.02637	0.0476219
rs2532282	17	41600673	C	G	0.806	1.00	-0.16	0.05	0.00128	0.0008995		0.823	0.78	-0.05724	0.02815	0.0418958
rs2696657	17	41600703	G	T	0.194	1.00	0.16	0.05	0.00128	0.0009002		0.190	0.84	0.05219	0.02637	0.0476811
rs2532281	17	41600948	G	T	0.806	1.00	-0.16	0.05	0.001281	0.0009006		0.823	0.78	-0.05727	0.02816	0.0418532
rs2532280	17	41601136	A	G	0.194	1.00	0.16	0.05	0.001281	0.000901		0.177	0.78	0.05727	0.02816	0.0418723
rs2696660	17	41601988	A	G	0.806	1.00	-0.16	0.05	0.001281	0.0009016		0.810	0.84	-0.05219	0.02639	0.0477725
rs2532278	17	41602182	A	C	0.194	1.00	0.16	0.05	0.001282	0.0009021		0.190	0.84	0.052187	0.02639	0.0478436
rs2532277	17	41602304	C	T	0.194	1.00	0.16	0.05	0.001283	0.0009033		0.177	0.78	0.057235	0.02818	0.0420861
rs2532276	17	41602401	A	C	0.194	1.00	0.16	0.05	0.001283	0.0009041		0.190	0.84	0.05219	0.0264	0.0478963
rs2532275	17	41602774	A	G	0.194	1.00	0.16	0.05	0.001284	0.0009049		0.190	0.84	0.052186	0.0264	0.0479548
rs2532273	17	41603091	C	T	0.806	1.00	-0.16	0.05	0.001261	0.0008909		0.810	0.84	-0.05218	0.02642	0.0480588
rs2532271	17	41603819	A	G	0.194	1.00	0.16	0.05	0.001258	0.0008889		0.190	0.84	0.052181	0.02642	0.0480879
rs1881193	17	41604546	C	T	0.194	1.00	0.16	0.05	0.001249	0.0008832		0.190	0.84	0.052139	0.02643	0.0483459
rs1881194	17	41604591	A	G	0.194	1.00	0.16	0.05	0.001246	0.0008809		0.190	0.84	0.052135	0.02643	0.0483805
rs17662889	17	41604873	A	C	0.812	0.99	-0.161	0.05	0.001368	0.000726		0.820	0.83	-0.05321	0.02725	0.0507117
rs17585974	17	41604976	G	T	0.193	0.95	0.162	0.051	0.001389	0.0007222		0.176	0.73	0.059787	0.02937	0.0416791
rs2696662	17	41605398	A	G	0.806	1.00	-0.16	0.05	0.00121	0.0008581		0.811	0.84	-0.05212	0.02645	0.0486448
rs2532270	17	41605577	A	G	0.194	1.00	0.161	0.05	0.001203	0.000854		0.190	0.84	0.052137	0.02646	0.0486068
rs2532269	17	41605885	C	T	0.194	1.00	0.161	0.05	0.001194	0.0008478		0.177	0.78	0.057132	0.02825	0.0430283
rs2532268	17	41606250	C	G	0.194	1.00	0.161	0.05	0.001197	0.0008491		0.177	0.77	0.057125	0.02826	0.0430848
rs2532267	17	41606393	A	G	0.194	1.00	0.161	0.05	0.001198	0.0008497		0.189	0.84	0.052093	0.02648	0.0490404
rs956329	17	41607007	C	T	0.194	1.00	0.161	0.05	0.001198	0.00085		0.189	0.83	0.052045	0.02652	0.0495424
rs2732643	17	41607684	C	T	0.806	1.00	-0.161	0.05	0.001199	0.0008503		0.811	0.83	-0.05205	0.02652	0.0495495
rs2696666	17	41607749	A	G	0.194	1.00	0.161	0.05	0.0012	0.0008506		0.189	0.83	0.052043	0.02652	0.0495867
rs2532264	17	41608193	C	G	0.194	1.00	0.161	0.05	0.0012	0.0008508		0.189	0.83	0.052037	0.02653	0.0496438
rs2532259	17	41609141	A	G	0.194	1.00	0.161	0.05	0.001205	0.0008529		0.189	0.83	0.052027	0.02653	0.0497267
rs2732645	17	41610068	C	T	0.806	1.00	-0.16	0.05	0.001205	0.0008532		0.811	0.83	-0.05201	0.02654	0.0498404
rs2732646	17	41610156	C	T	0.806	1.00	-0.16	0.05	0.001206	0.0008535		0.811	0.83	-0.05202	0.02654	0.0498547
rs2732647	17	41610190	A	G	0.806	1.00	-0.16	0.05	0.001206	0.0008537		0.811	0.83	-0.05202	0.02655	0.0498877
rs2532257	17	41610271	C	T	0.194	1.00	0.16	0.05	0.001207	0.0008539		0.189	0.83	0.052019	0.02655	0.0499091
rs2532256	17	41610794	C	T	0.194	1.00	0.16	0.05	0.001207	0.000854		0.189	0.83	0.052017	0.02655	0.0499526
rs2532255	17	41611309	C	T	0.194	1.00	0.16	0.05	0.001208	0.0008544		0.189	0.83	0.052014	0.02656	0.0499946
rs2696696	17	41611554	A	G	0.194	1.00	0.16	0.05	0.001209	0.0008546		0.189	0.83	0.051983	0.02656	0.0501684
rs2532253	17	41612073	A	G	0.194	1.00	0.16	0.05	0.001211	0.0008556		0.177	0.77	0.056984	0.02835	0.0442879
rs740708	17	41612432	A	G	0.194	1.00	0.16	0.05	0.001212	0.000856		0.189	0.83	0.051958	0.02657	0.0503504
rs1881195	17	41613250	C	T	0.194	1.00	0.16	0.05	0.001212	0.0008563		0.189	0.83	0.051963	0.02657	0.0503429
rs740706	17	41614131	C	G	0.194	1.00	0.16	0.05	0.001213	0.0008564		0.189	0.83	0.05196	0.02657	0.0503721
rs758523	17	41614199	G	T	0.806	1.00	-0.16	0.05	0.001213	0.0008568		0.811	0.83	-0.05197	0.02657	0.0503628
rs740705	17	41614223	A	G	0.812	0.99	-0.162	0.05	0.001347	0.000712		0.820	0.82	-0.05287	0.02735	0.0530263
rs12150012	17	41615296	A	G	0.806	1.00	-0.16	0.05	0.001214	0.0008575		0.811	0.83	-0.05194	0.02659	0.0505778
rs2732585	17	41615316	A	C	0.806	1.00	-0.16	0.05	0.001217	0.0008584		0.823	0.77	-0.05696	0.02838	0.0445691
rs2696701	17	41615696	A	G	0.806	1.00	-0.16	0.05	0.001217	0.0008584		0.811	0.83	-0.05191	0.0266	0.0508454
rs2696702	17	41616193	C	T	0.194	1.00	0.16	0.05	0.001218	0.0008588		0.189	0.83	0.051897	0.02661	0.0509757
rs2696704	17	41617530	C	T	0.194	1.00	0.16	0.05	0.001219	0.0008592		0.189	0.83	0.051893	0.02661	0.0510071
rs2532246	17	41618195	A	G	0.194	1.00	0.16	0.05	0.001219	0.0008596		0.189	0.83	0.051893	0.02661	0.0510321
rs17663351	17	41619700	C	T	0.194	1.00	0.16	0.05	0.001221	0.0008606		0.189	0.83	0.05187	0.02663	0.0512612
rs2696706	17	41619822	C	T	0.194	1.00	0.16	0.05	0.001222	0.0008609		0.189	0.83	0.051849	0.02663	0.0513641
rs2696707	17	41620494	A	C	0.806	1.00	-0.16	0.05	0.00124	0.0008693		0.811	0.83	-0.05184	0.02663	0.0514516
rs2696708	17	41620720	C	T	0.194	1.00	0.16	0.05	0.001242	0.0008702		0.189	0.82	0.051687	0.02669	0.0526403
rs2696709	17	41621254	C	G	0.194	1.00	0.16	0.05	0.001245	0.0008709		0.177	0.76	0.056695	0.02848	0.04635
rs2732589	17	41621799	A	G	0.806	1.00	-0.16	0.05	0.001243	0.0008708		0.811	0.82	-0.05169	0.02669	0.0526403
rs1918788	17	41623394	A	G	0.813	0.99	-0.161	0.05	0.001388	0.0007258		0.821	0.82	-0.05246	0.02741	0.055466

					GOYA QC		GOYA Overweight/control			GOYA BMI continuous	known	IARC QC		IARC results		
MARKER	chr	position	A1	A2	FREQ1	Rsqr	Beta	SE	p	p	gene name	freq1	Rsqr	in_beta	in_SE	in_p
rs2696526	17	41623640	A	G	0.194	1.00	0.16	0.05	0.001248	0.000873		0.189	0.82	0.051674	0.0267	0.0527469
rs2732596	17	41625323	C	T	0.806	1.00	-0.16	0.05	0.00125	0.000874		0.811	0.82	-0.05167	0.0267	0.052792
rs2532235	17	41627777	A	G	0.188	0.99	0.161	0.05	0.001391	0.0007268		0.179	0.82	0.052455	0.02741	0.0555123
rs2532234	17	41628043	A	G	0.806	1.00	-0.16	0.05	0.001251	0.0008743		0.811	0.82	-0.05167	0.0267	0.0528404
rs17663792	17	41628329	C	T	0.806	1.00	-0.16	0.05	0.001252	0.0008748		0.811	0.82	-0.05166	0.02671	0.0528752
rs2732660	17	41628456	C	G	0.194	1.00	0.16	0.05	0.001253	0.0008752		0.189	0.82	0.051649	0.02671	0.052967
rs2696438	17	41628705	A	C	0.194	1.00	0.16	0.05	0.001253	0.0008754		0.189	0.82	0.051649	0.02671	0.0529775
rs1048343	17	41629666	C	T	0.813	0.99	-0.161	0.05	0.001394	0.0007275		0.821	0.82	-0.05207	0.02745	0.0576435
rs1048333	17	41629696	A	G	0.813	0.99	-0.161	0.05	0.001394	0.0007274		0.821	0.82	-0.05207	0.02745	0.0576369
rs2696440	17	41630337	C	T	0.194	1.00	0.16	0.05	0.001258	0.0008777		0.188	0.81	0.050832	0.02687	0.0583013
rs1815	17	41632355	C	T	0.194	1.00	0.16	0.05	0.00126	0.0008787		0.188	0.81	0.050833	0.02687	0.0583013
rs1816	17	41632395	A	G	0.806	1.00	-0.16	0.05	0.001261	0.0008791		0.812	0.81	-0.05082	0.02687	0.0583652
rs2696443	17	41632598	A	G	0.194	1.00	0.16	0.05	0.001261	0.000879		0.188	0.81	0.050828	0.02687	0.0583543
rs2532229	17	41633042	A	C	0.806	1.00	-0.16	0.05	0.001261	0.0008794		0.812	0.81	-0.0508	0.02687	0.0585306
rs2732665	17	41633468	C	T	0.194	1.00	0.16	0.05	0.001262	0.0008798		0.188	0.81	0.050792	0.02687	0.0585552
rs2532228	17	41633595	A	G	0.813	0.99	-0.161	0.05	0.001403	0.00073		0.822	0.82	-0.05128	0.02748	0.0618268
rs2696446	17	41634438	A	G	0.194	1.00	0.16	0.05	0.001268	0.0008828		0.176	0.76	0.055545	0.02866	0.0524101
rs17664048	17	41634519	A	G	0.806	1.00	-0.16	0.05	0.001265	0.0008809		0.812	0.81	-0.05075	0.02688	0.0588057
rs1358438	17	41635537	A	G	0.194	1.00	0.16	0.05	0.001266	0.0008816		0.188	0.81	0.050744	0.02688	0.0588461
rs1358437	17	41635580	G	T	0.194	1.00	0.16	0.05	0.001267	0.0008821		0.188	0.81	0.050718	0.02688	0.0589845
rs2732675	17	41635965	A	T	0.194	1.00	0.16	0.05	0.00127	0.0008835		0.188	0.81	0.050685	0.02688	0.0591753
rs2732674	17	41636474	C	T	0.194	1.00	0.16	0.05	0.001274	0.0008855		0.188	0.81	0.05068	0.02688	0.059207
rs2668659	17	41637229	A	G	0.806	1.00	-0.16	0.05	0.001275	0.0008857		0.813	0.81	-0.05068	0.02688	0.0592191
rs2696455	17	41639348	C	T	0.806	1.00	-0.16	0.05	0.001276	0.0008862		0.813	0.81	-0.05066	0.02688	0.0592995
rs2668653	17	41643933	C	T	0.194	1.00	0.16	0.05	0.001281	0.0008884		0.188	0.81	0.05065	0.02688	0.0593878
rs2532419	17	41644058	C	T	0.813	0.99	-0.161	0.05	0.001424	0.0007375		0.822	0.82	-0.05112	0.02748	0.0626502
rs2732628	17	41644061	A	G	0.194	1.00	0.16	0.05	0.001283	0.0008894		0.187	0.81	0.050634	0.02689	0.0594856
rs2532418	17	41644356	C	T	0.194	1.00	0.16	0.05	0.001285	0.00089		0.187	0.81	0.050636	0.02689	0.0594856
rs2668645	17	41644417	A	G	0.806	1.00	-0.16	0.05	0.001285	0.0008903		0.813	0.81	-0.05058	0.02689	0.0597745
rs2732629	17	41644878	A	G	0.806	1.00	-0.16	0.05	0.001285	0.0008905		0.813	0.81	-0.05056	0.02689	0.0598823
rs2732630	17	41644927	A	C	0.806	1.00	-0.16	0.05	0.001286	0.0008908		0.813	0.81	-0.05057	0.02689	0.0598315
rs2532417	17	41644997	C	T	0.806	1.00	-0.16	0.05	0.001287	0.0008914		0.813	0.81	-0.05055	0.02689	0.059981
rs2732631	17	41645009	G	T	0.806	1.00	-0.16	0.05	0.001288	0.0008918		0.813	0.81	-0.05054	0.02689	0.0600378
rs2532416	17	41645068	A	C	0.806	1.00	-0.16	0.05	0.001289	0.000892		0.813	0.81	-0.05053	0.0269	0.0601139
rs2668665	17	41645824	A	G	0.194	1.00	0.16	0.05	0.00129	0.0008926		0.187	0.81	0.050515	0.0269	0.0601803
rs2732606	17	41647142	C	T	0.806	1.00	-0.16	0.05	0.00129	0.0008928		0.813	0.81	-0.0505	0.0269	0.0602551
rs2732605	17	41647158	A	G	0.806	1.00	-0.16	0.05	0.001291	0.0008932		0.813	0.81	-0.05051	0.0269	0.060222
rs2668695	17	41647903	C	T	0.194	1.00	0.16	0.05	0.001292	0.0008934		0.187	0.81	0.050484	0.0269	0.0603544
rs2668692	17	41648797	A	G	0.194	1.00	0.16	0.05	0.001293	0.0008939		0.187	0.81	0.050484	0.0269	0.0603551
rs2668691	17	41649323	C	T	0.813	0.99	-0.161	0.05	0.001442	0.0007441		0.822	0.82	-0.05092	0.02748	0.0636804
rs2696689	17	41652925	A	G	0.194	0.99	0.159	0.05	0.001366	0.0009347		0.186	0.89	0.049528	0.0257	0.0537505
rs2668711	17	41697404	G	T	0.891	0.65	-0.253	0.077	0.001112	0.0003004		0.935	0.39	-0.11999	0.06326	0.057691
rs2732651	17	41700840	C	T	0.812	0.98	-0.157	0.051	0.001867	0.0008889		0.835	0.85	-0.05417	0.02788	0.0518801
rs2668719	17	41700867	A	C	0.188	0.98	0.157	0.051	0.001873	0.0008905		0.165	0.85	0.054129	0.02788	0.0520329
rs2696558	17	41702728	A	T	0.313	0.86	0.184	0.046	0.0000569	0.000017		0.286	0.70	0.026729	0.0245	0.2749466
rs2458216	17	41702739	C	T	0.813	0.98	-0.155	0.051	0.002154	0.0009741		0.836	0.85	-0.05376	0.02785	0.0534312
rs2532335	17	41702874	A	G	0.813	0.98	-0.155	0.051	0.002158	0.0009753		0.836	0.85	-0.05366	0.02784	0.0537695
rs2532332	17	41703504	A	T	0.813	0.98	-0.155	0.051	0.002225	0.0009944		0.836	0.85	-0.05357	0.02784	0.05416
rs2950694	17	41704075	A	G	0.813	0.98	-0.155	0.051	0.002227	0.0009952		0.836	0.85	-0.0535	0.02783	0.0544076
rs2668624	17	41708649	A	G	0.812	0.98	-0.151	0.051	0.002731	0.0009768		0.827	0.94	-0.04663	0.02593	0.0719049
rs2668625	17	41708747	A	C	0.188	0.98	0.151	0.051	0.002736	0.0009749		0.173	0.94	0.046605	0.02593	0.0720442
rs2668626	17	41708952	C	T	0.188	0.98	0.152	0.051	0.002737	0.0009714		0.162	0.88	0.050935	0.02757	0.064468
rs2668627	17	41709470	C	T	0.188	0.98	0.151	0.051	0.00274	0.0009701		0.173	0.94	0.04635	0.02589	0.0732333
rs2732702	17	41709505	G	T	0.188	0.98	0.151	0.051	0.002741	0.000968		0.173	0.94	0.046327	0.02589	0.0733476
rs2668628	17	41709662	A	G	0.188	0.98	0.151	0.051	0.00275	0.0009614		0.173	0.94	0.046313	0.02589	0.0734184
rs2732701	17	41709880	A	G	0.812	0.98	-0.151	0.051	0.002753	0.0009594		0.827	0.94	-0.04631	0.02589	0.0734499
rs2261201	17	41710326	C	G	0.812	0.98	-0.151	0.051	0.002755	0.0009587		0.827	0.94	-0.04629	0.02589	0.0735015
rs2696531	17	41711411	C	G	0.804	0.98	-0.151	0.05	0.002536	0.0009776		0.818	0.92	-0.04529	0.02546	0.0751403
rs2696530	17	41711491	A	G	0.804	0.98	-0.151	0.05	0.002542	0.0009748		0.818	0.92	-0.04525	0.02546	0.0753251
rs2696525	17	41712414	C	T	0.804	0.98	-0.151	0.05	0.002543	0.0009727		0.818	0.92	-0.04523	0.02546	0.0754678
rs17588637	17	41713074	G	T	0.804	0.98	-0.151	0.05	0.002545	0.0009728		0.818	0.92	-0.04522	0.02546	0.0755076
rs17665188	17	41713128	C	T	0.196	0.98	0.151	0.05	0.002551	0.000972		0.182	0.93	0.045194	0.02546	0.075653
rs2532324	17	41713538	A	C	0.194	0.97	0.153	0.05	0.002319	0.0009294		0.169	0.85	0.047612	0.02729	0.0808302
rs2696501	17	41719662	G	T	0.811	0.99	-0.148	0.05	0.003112	0.0008173		0.830	0.98	-0.04398	0.02557	0.085185
rs2732615	17	41719833	C	T	0.189	0.99	0.147	0.05	0.00324	0.0007964		0.171	0.98	0.043983	0.02557	0.0851323
rs2732614	17	41719986	C	T	0.806	0.96	-0.149	0.05	0.003191	0.0008116		0.833	0.87	-0.04947	0.02734	0.0702033
rs2732613	17	41720606	A	C	0.811	1.00	-0.147	0.05	0.00332	0.0007876		0.830	0.98	-0.0439	0.02555	0.0855864
rs2532426	17	41724181	C	T	0.811	1.00	-0.147	0.05	0.003342	0.0007857		0.830	0.98	-0.0438	0.02554	0.0861181
rs538628	17	42142496	C	G	0.190	1.00	0.147	0.05	0.003349	0.0007855		0.170	0.98	0.043778	0.02554	0.0862696
rs183211	17	42143493	A	G	0.217	1.00	0.137	0.047	0.003566	0.0006529		0.187	0.95	0.035251	0.02499	0.1580369
rs199436	17	42144468	A	G	0.783	1.00	-0.137	0.047	0.003556	0.0006504		0.814	0.95	-0.03526	0.02499	0.1579431
rs199438	17	42146826	A	G	0.217	1.00	0.138	0.047	0.003547	0.0006483		0.186	0.95	0.03527	0.025	0.1578993
rs199439	17	42148686	A	G	0.811	1.00	-0.147	0.05	0.003239	0.0007532		0.830	0.98	-0.04373	0.02554	0.0866341
rs142167	17	42150418	A	G	0.783	1.00	-0.138	0.047	0.003521	0.0006421		0.814	0.95	-0.03529	0.02501	0.157893
rs199456	17	42153103	C	T	0.811	1.00	-0.147	0.05	0.003194	0.0007402		0.830	0.98	-0.04367	0.02554	0.0870795
rs199455	17	42154400	C	T	0.217	1.00	0.138	0.047	0.003527	0.0006432		0.186	0.95	0.03534	0.02503	0.1577285
rs199454	17	42155294	A	G	0.783	1.00	-0.138	0.047	0.003558	0.0006462		0.814	0.95	-0.03532	0.02504	0.1579879
rs199452	17	42156524	C	T	0.783	1.00	-0.137	0.047	0.003578	0.0006472		0.814	0.95	-0.03533	0.02504	0.1578878
rs199449	17	42164086	A	G	0.217	1.00	0.137	0.047	0.003602	0.0006487		0.186	0.95	0.03532	0.02504	0.1580589
rs199448	17	42164185	A	G	0.811	1.00	-0.147	0.05	0.003209	0.0007247		0.831	0.99	-0.04347	0.02553	0.0883162
rs199445	17	42172573	C	T	0.811	1.00	-0.147	0.05	0.003321	0.0007247		0.831	0.99	-0.04336	0.02552	0.0890397
rs199443	17	42174733	C	T	0.811	1.00	-0.146	0.05	0.003433	0.0007282		0.831	0.99	-0.04328	0.02551	0.0894763

					GOYA QC		GOYA Overweight/control			GOYA BMI continuous	known	IARC QC		IARC results		
MARKER	chr	position	A1	A2	FREQ1	Rsqr	Beta	SE	p	p	gene name	freq1	Rsqr	in_beta	in_SE	in_p
rs199442	17	42175290	A	G	0.217	1.00	0.136	0.047	0.003918	0.0006585		0.185	0.95	0.035292	0.02505	0.1585837
rs199536	17	42175593	C	T	0.783	1.00	-0.136	0.047	0.004031	0.0006613		0.815	0.95	-0.03528	0.02505	0.158701
rs199535	17	42177829	A	G	0.811	1.00	-0.145	0.05	0.003617	0.0007382		0.831	0.99	-0.04318	0.02549	0.0900835
rs199534	17	42179380	G	T	0.189	1.00	0.145	0.05	0.003652	0.0007403		0.169	0.99	0.043167	0.02549	0.0901418
rs199533	17	42184098	A	G	0.189	1.00	0.144	0.05	0.003804	0.0007519		0.168	0.99	0.043097	0.02548	0.0905342
rs199530	17	42191820	A	G	0.783	1.00	-0.135	0.047	0.004278	0.0006794		0.813	0.93	-0.03428	0.02524	0.1740977
rs199529	17	42192384	A	C	0.783	1.00	-0.135	0.047	0.004286	0.0006816		0.813	0.93	-0.03424	0.02525	0.1746898
rs199528	17	42198305	C	T	0.813	0.98	-0.143	0.05	0.004535	0.0009079		0.830	0.91	-0.03911	0.02647	0.1393366
rs199524	17	42203606	G	T	0.214	0.98	0.135	0.048	0.004893	0.0007769		0.187	0.88	0.031849	0.026	0.2202334
rs199520	17	42209035	A	G	0.786	0.98	-0.135	0.048	0.004943	0.0007878		0.813	0.88	-0.03182	0.026	0.2207129
rs199516	17	42211648	C	T	0.187	0.99	0.143	0.05	0.0047	0.0009513		0.171	0.91	0.03885	0.02649	0.1421453
rs7208024	17	44123853	C	G	0.137	0.99	-0.189	0.057	0.0009127	0.012236		0.113	0.98	0.011374	0.03061	0.7099409
rs7213972	17	44123898	G	T	0.137	0.99	-0.189	0.057	0.0009275	0.0123477		0.113	0.98	0.011409	0.0306	0.7090178
rs8081391	17	44127199	A	G	0.137	0.99	-0.188	0.057	0.000956	0.0125623		0.113	0.99	0.011924	0.03045	0.6951336
rs7222822	17	44129871	C	T	0.863	0.98	0.188	0.057	0.0009805	0.0127334		0.887	0.97	-0.01189	0.03071	0.6985325
rs7214800	17	44241613	C	G	0.847	0.99	-0.181	0.054	0.0007612	0.0159043		0.885	1.00	0.011512	0.02871	0.6881944
rs2671641	17	44952726	C	G	0.144	0.81	0.214	0.062	0.0005868	0.0013923		0.139	0.74	-0.02087	0.03215	0.5158771
rs169217	17	45368187	A	T	0.699	0.51	-0.2	0.059	0.0006727	0.0038195		0.701	0.31	-0.06201	0.0357	0.0821791
rs11871528	17	47370786	G	T	0.975	0.99	-0.423	0.128	0.0009324	0.0010267		0.973	0.96	-0.05933	0.05325	0.2648014
rs10515134	17	52218447	A	G	0.073	1.00	-0.265	0.076	0.0004623	0.0013349		0.086	1.00	-0.01323	0.03016	0.660607
rs6503772	17	52271688	A	C	0.571	1.00	-0.137	0.039	0.0004346	0.0019164		0.575	0.99	0.018865	0.01852	0.3079098
rs11651692	17	52293014	A	G	0.289	0.95	-0.172	0.044	0.0000821	0.0002969		0.283	0.93	0.007944	0.02074	0.7014102
rs11651323	17	52302968	C	G	0.705	0.92	0.169	0.044	0.0001287	0.0004163		0.709	0.90	-0.00905	0.02102	0.6666557
rs9895389	17	52697517	C	T	0.863	1.00	-0.19	0.056	0.0007558	0.0003999		0.875	0.54	0.001716	0.03771	0.9636665
rs2465454	17	58113684	A	C	0.014	0.93	0.478	0.177	0.006846	0.0009839		0.018	0.87	-0.11792	0.08055	0.1429437
rs2429388	17	58117763	A	G	0.014	0.94	0.479	0.177	0.006665	0.0009592		0.017	0.97	-0.10736	0.07859	0.1715622
rs2465429	17	58120215	A	G	0.013	0.96	0.483	0.176	0.00618	0.0008956		0.017	0.97	-0.10698	0.07851	0.1726733
rs2460289	17	58120465	A	G	0.987	0.97	-0.484	0.176	0.006018	0.000873		0.984	0.97	0.106938	0.07849	0.1727229
rs2465428	17	58120518	G	T	0.013	0.98	0.486	0.176	0.005834	0.0008498		0.017	0.97	-0.10682	0.07848	0.1731219
rs2465427	17	58120536	C	T	0.013	0.99	0.486	0.176	0.005805	0.0008468		0.017	0.97	-0.10681	0.07846	0.1730953
rs2429387	17	58120747	A	G	0.987	0.99	-0.486	0.176	0.005791	0.0008462		0.984	0.97	0.106676	0.07844	0.1734859
rs2465426	17	58121193	C	G	0.013	0.99	0.489	0.177	0.005639	0.0008226		0.017	0.96	-0.10763	0.079	0.1726964
rs11868614	17	58262928	A	G	0.384	0.84	-0.151	0.044	0.0005444	0.0202156		0.377	0.78	-0.02437	0.02101	0.2457367
rs11871421	17	58267175	C	T	0.616	0.85	0.15	0.043	0.0005424	0.0202294		0.623	0.81	0.024306	0.0207	0.2400041
rs12051594	17	58281335	A	G	0.402	0.97	-0.135	0.04	0.0007715	0.0208951		0.411	0.95	-0.03194	0.0189	0.0908205
rs7216013	17	58346265	A	C	0.096	1.00	0.24	0.066	0.0002668	0.0011793		0.134	0.60	0.064268	0.03404	0.0588238
rs9898645	17	62826620	G	T	0.922	0.75	0.233	0.084	0.005313	0.0002967		0.909	0.54	0.027493	0.04292	0.5214193
rs7223685	17	66500336	A	T	0.252	0.99	0.13	0.045	0.003606	0.0001884		0.199	0.94	-0.016	0.02303	0.4868854
rs1005316	17	66501964	A	G	0.238	0.99	0.135	0.045	0.002992	0.0001225		0.181	1.00	-0.019	0.02302	0.4087852
rs8080412	17	66508046	A	C	0.761	1.00	-0.134	0.045	0.003191	0.000118		0.817	0.99	0.017479	0.02299	0.4466445
rs758022	17	66518601	C	T	0.238	0.92	0.128	0.047	0.006663	0.0002465		0.179	0.86	-0.01733	0.02491	0.4863789
rs12939126	17	68172022	C	T	0.984	0.63	0.767	0.206	0.000194	0.0009896		0.989	0.27	0.033861	0.18912	0.8577797
rs7222924	17	71055772	G	T	0.471	0.93	0.122	0.04	0.002523	0.0007962		0.490	0.92	0.025703	0.01925	0.181462
rs11652039	17	72622221	A	G	0.162	0.96	0.185	0.053	0.0005153	0.0007627		0.160	0.90	-0.04346	0.0265	0.1007614
rs16969682	17	72638607	A	C	0.840	1.00	-0.191	0.053	0.0002926	0.0005746		0.851	0.98	0.04062	0.02619	0.1206016
rs16967342	17	72653238	C	T	0.058	1.00	-0.297	0.084	0.000382	0.0007899		0.063	1.00	-0.02246	0.03629	0.5356073
rs2176757	17	72657861	C	T	0.059	1.00	-0.297	0.083	0.0003648	0.0007903		0.064	1.00	-0.02168	0.03612	0.5479531
rs2411070	17	72660408	A	G	0.941	1.00	0.297	0.083	0.0003699	0.0007996		0.937	1.00	0.021864	0.03612	0.5446918
rs2008417	17	72667371	C	T	0.073	1.00	-0.33	0.076	0.0000129	0.0000613		0.082	0.94	0.002377	0.03348	0.9433463
rs17612259	17	72669461	A	T	0.073	1.00	-0.33	0.076	0.0000128	0.0000611		0.082	0.94	0.002381	0.03349	0.9432573
rs17612441	17	72694406	C	T	0.058	1.00	-0.299	0.084	0.0003526	0.0008458		0.064	0.99	-0.02295	0.03625	0.5263465
rs6501941	17	72695976	C	T	0.058	0.99	-0.301	0.084	0.0003368	0.0008437		0.064	0.99	-0.02298	0.03625	0.5258987
rs7222475	17	72696238	A	G	0.058	0.99	-0.302	0.084	0.0003253	0.0008437		0.064	0.99	-0.02298	0.03626	0.5259187
rs17540424	17	72701707	C	T	0.942	0.99	0.305	0.084	0.0002981	0.0008506		0.936	0.99	0.022963	0.0363	0.5266635
rs2271792	17	72713124	C	G	0.058	0.99	-0.306	0.084	0.0002959	0.0008467		0.064	0.95	-0.02314	0.03684	0.529588
rs17540750	17	72716740	A	G	0.942	0.98	0.306	0.085	0.0002965	0.0008616		0.935	0.86	0.02393	0.03851	0.5339852
rs2280270	17	72717157	C	T	0.939	0.93	0.305	0.084	0.0003033	0.000859		0.930	0.77	0.027415	0.03939	0.486106
rs7215278	17	72726698	G	T	0.917	0.73	0.346	0.083	0.0000297	0.0004348		0.905	0.49	-0.05376	0.04316	0.2126342
rs16969774	17	72730163	G	T	0.110	0.99	-0.276	0.063	0.0000114	0.0009561		0.134	0.27	0.039547	0.04856	0.4150751
rs7501958	17	73272598	C	T	0.487	0.99	-0.151	0.039	0.0001008	0.0000745		0.471	0.74	0.016166	0.02105	0.4420424
rs7209723	17	76212934	C	G	0.226	0.98	0.133	0.046	0.004164	0.0007879		0.237	0.97	0.009458	0.02168	0.6623504
rs6565472	17	76257215	C	T	0.790	1.00	-0.147	0.047	0.001842	0.0003351		0.783	1.00	-0.00515	0.02172	0.8123575
rs9807041	17	76262465	A	G	0.790	1.00	-0.147	0.047	0.001804	0.0003323		0.783	1.00	-0.00515	0.0217	0.812364
rs11869453	17	76283025	A	G	0.219	0.95	0.148	0.047	0.001827	0.0005512		0.221	0.94	0.006186	0.02237	0.7819725
rs11655435	17	76284038	C	G	0.219	1.00	0.15	0.046	0.001199	0.0004183		0.222	1.00	0.005856	0.02157	0.7858945
rs9911171	17	76285498	C	T	0.784	0.99	-0.148	0.047	0.001656	0.0004646		0.783	0.98	-0.00321	0.0219	0.883501
rs8065598	17	76292838	A	G	0.219	1.00	0.15	0.046	0.001217	0.000424		0.222	1.00	0.005803	0.02157	0.7877587
rs9898952	17	76292994	A	G	0.763	0.94	-0.14	0.047	0.002628	0.0009631		0.759	0.93	-0.00742	0.0218	0.7334073
rs11150739	17	76293574	A	G	0.796	0.95	-0.156	0.049	0.001438	0.000656		0.793	0.94	0.000103	0.02303	0.9964145
rs8081444	17	76297164	A	G	0.204	0.95	0.156	0.049	0.001464	0.0006671		0.208	0.94	-0.00011	0.02303	0.9962003
rs7211818	17	76303498	A	G	0.781	1.00	-0.149	0.046	0.001293	0.0004498		0.778	1.00	-0.00578	0.02157	0.7886376
rs9896820	17	76308129	C	T	0.781	1.00	-0.149	0.046	0.00128	0.000448		0.778	1.00	-0.00577	0.02158	0.7891285
rs9899843	17	76315428	C	T	0.781	1.00	-0.15	0.046	0.001271	0.0004528		0.779	0.99	-0.00553	0.02177	0.7994401
rs9674559	17	76330203	A	G	0.780	1.00	-0.148	0.046	0.001402	0.0005326		0.780	0.99	-0.00536	0.02186	0.8061683
rs9894401	17	76357121	A	G	0.259	1.00	0.137	0.044	0.001757	0.0009734		0.268	0.99	0.014637	0.02055	0.4759842
rs8077901	17	76363397	G	T	0.742	0.99	-0.138	0.044	0.00174	0.0007471		0.743	0.99	-0.01624	0.0207	0.4324965
rs1383227	18	435039	C	T	0.196	1.00	0.173	0.048	0.0003461	0.0022801		0.187	0.99	-0.01134	0.02337	0.6273482
rs1893134	18	440102	A	G	0.196	1.00	0.174	0.048	0.0003009	0.0017715		0.188	1.00	-0.01142	0.02328	0.6233163
rs9967577	18	506584	A	G	0.883	0.64	-0.215	0.075	0.00409	0.000121		0.881	0.67	0.013129	0.0323	0.684166
rs3897601	18	757210	A	G	0.467	1.00	0.11	0.039	0.004634	0.0009339		0.487	1.00	-0.02629	0.01846	0.1539581
rs491222	18	2079892	A	G	0.436	0.76	0.139	0.044	0.001748	0.0005333		0.424	0.70	0.027681	0.02216	0.2112455
rs12967693	18	2095249	A	G	0.737	0.88	0.157	0.047	0.0008659	0.0017678		0.778	0.83	0.049086	0.02487	0.0482542

					GOYA QC		GOYA Overweight/control			GOYA BMI continuous	known	IARC QC		IARC results		
MARKER	chr	position	A1	A2	FREQ1	Rsqr	Beta	SE	p	p	gene name	freq1	Rsqr	in_beta	in_SE	in_p
rs16945616	18	3891635	C	G	0.024	0.83	-0.397	0.142	0.005097	0.0005652		0.021	0.75	-0.10599	0.08045	0.187315
rs16945619	18	3898444	C	T	0.024	0.86	-0.386	0.138	0.005154	0.000605		0.021	0.81	-0.09534	0.07719	0.2164204
rs16945636	18	3907600	C	T	0.024	0.87	-0.381	0.137	0.005477	0.0006545		0.021	0.82	-0.09304	0.07688	0.2258212
rs16945669	18	3921898	C	G	0.026	0.95	-0.384	0.128	0.002721	0.0003561		0.021	0.91	-0.06672	0.07225	0.3553976
rs16945698	18	3932065	C	G	0.026	0.93	-0.393	0.129	0.002403	0.0003087		0.021	0.89	-0.0655	0.07357	0.3729159
rs2174539	18	5212012	C	G	0.985	0.47	-0.665	0.232	0.004164	0.0007166		0.987	0.42	-0.18419	0.11465	0.107877
rs11663723	18	6160017	A	G	0.045	1.00	-0.298	0.095	0.001827	0.0002841		0.043	0.61	-0.03937	0.05783	0.4957103
rs9966928	18	6533564	A	G	0.418	0.81	-0.147	0.043	0.0006931	0.0027792		0.416	0.72	-0.00291	0.02172	0.8932434
rs1941494	18	6539764	A	G	0.402	1.00	-0.132	0.039	0.0007932	0.0012309		0.413	1.00	-0.00451	0.01863	0.8084441
rs367057	18	7164228	A	G	0.712	0.91	-0.159	0.045	0.0003594	0.0012351		0.712	0.92	0.016468	0.02058	0.4231331
rs454719	18	7164604	G	T	0.712	0.92	-0.159	0.044	0.0003611	0.0012426		0.712	0.93	0.016459	0.02056	0.4230261
rs333100	18	7166547	C	G	0.705	0.99	-0.143	0.043	0.000792	0.0024896		0.705	0.98	0.015135	0.01986	0.4455662
rs333099	18	7166761	G	T	0.295	1.00	0.142	0.042	0.0007968	0.0025057		0.295	0.99	-0.01504	0.01974	0.4457187
rs333097	18	7167383	G	T	0.705	0.99	-0.143	0.042	0.0007908	0.0024786		0.705	0.98	0.014943	0.01983	0.4508094
rs12607083	18	7434318	C	T	0.842	0.51	-0.245	0.074	0.0008627	0.0009303		0.838	0.53	0.039477	0.03288	0.2295302
rs582683	18	7707565	A	T	0.369	0.87	0.161	0.043	0.0001572	0.0000444		0.393	0.80	0.015596	0.02078	0.4525497
rs594771	18	7753363	A	G	0.450	0.96	-0.131	0.04	0.0009865	0.000245		0.426	0.95	-0.00929	0.01906	0.6256551
rs4798593	18	7761132	C	T	0.523	1.00	-0.12	0.039	0.001834	0.0008556		0.489	1.00	-0.01169	0.0186	0.529347
rs875530	18	7764104	C	T	0.523	1.00	-0.12	0.039	0.001834	0.0008562		0.489	0.99	-0.01164	0.01862	0.5315582
rs2155524	18	7773076	A	T	0.455	0.99	0.121	0.039	0.001975	0.0005371		0.498	0.96	0.019289	0.01883	0.3051849
rs655078	18	7777550	C	T	0.545	0.99	-0.121	0.039	0.001979	0.0005385		0.502	0.96	-0.01927	0.01883	0.3058574
rs4614805	18	7778329	C	T	0.811	1.00	0.135	0.05	0.006433	0.0007084		0.829	0.99	0.030835	0.02453	0.2083176
rs422656	18	7783429	A	C	0.631	1.00	-0.138	0.04	0.0006233	0.000303		0.600	0.83	-0.02079	0.0203	0.3052493
rs746128	18	7784366	C	T	0.480	0.98	0.121	0.039	0.001844	0.000792		0.510	0.96	0.011019	0.01889	0.5593338
rs624519	18	7785543	A	T	0.629	0.98	-0.14	0.041	0.0005696	0.0002384		0.600	0.83	-0.02076	0.02036	0.3074055
rs789451	18	7785723	A	G	0.475	0.99	-0.129	0.039	0.0009212	0.0000574		0.468	0.79	-0.02288	0.0207	0.2686318
rs638251	18	7786393	C	T	0.451	0.85	0.145	0.042	0.0005957	0.0000662		0.468	0.83	-0.00207	0.02009	0.9180123
rs653973	18	7787536	G	T	0.471	0.87	0.134	0.041	0.001248	0.0001975		0.486	0.85	-0.00882	0.0199	0.6573896
rs331426	18	7799127	A	G	0.560	0.99	-0.109	0.039	0.005428	0.0008218		0.546	0.99	0.000321	0.01856	0.9862006
rs331425	18	7799253	A	G	0.560	0.99	-0.109	0.039	0.005445	0.0008233		0.546	0.99	0.000306	0.01856	0.9868255
rs11876907	18	8609336	A	G	0.062	0.98	-0.231	0.082	0.005055	0.0007964		0.051	0.79	-0.02861	0.04934	0.5616159
rs497084	18	8760517	C	T	0.763	0.87	0.175	0.049	0.000369	0.0005382		0.745	0.82	-0.01593	0.02368	0.5007474
rs9956741	18	8921724	G	T	0.522	0.90	-0.117	0.04	0.003709	0.0006914		0.501	0.85	0.03091	0.01968	0.1160896
rs17435624	18	10040992	A	G	0.812	0.48	-0.166	0.071	0.01977	0.0008952		0.834	0.41	-0.00618	0.03725	0.868207
rs7228576	18	10398492	C	T	0.867	1.00	0.193	0.057	0.000758	0.000087		0.864	0.48	-0.02384	0.03933	0.5440615
rs11664249	18	10407003	C	T	0.859	0.96	0.179	0.057	0.001699	0.0003142		0.866	0.59	-0.01118	0.036	0.7559025
rs17542665	18	10407527	A	T	0.853	0.92	0.175	0.057	0.002218	0.0004486		0.862	0.57	-0.01335	0.03599	0.7105121
rs10468666	18	10419422	C	T	0.603	0.80	-0.143	0.045	0.001321	0.00049		0.573	0.76	0.021305	0.02081	0.3054864
rs11663409	18	10420126	C	T	0.572	0.88	-0.144	0.042	0.0006589	0.0001195		0.547	0.83	0.018088	0.01995	0.3641489
rs11660127	18	10420734	C	T	0.427	0.89	0.143	0.042	0.000652	0.0001174		0.453	0.84	-0.01794	0.0198	0.364296
rs11080418	18	10421749	C	T	0.427	0.90	0.143	0.042	0.0006395	0.0001141		0.452	0.86	-0.01777	0.01965	0.3655195
rs12955584	18	10423382	A	G	0.534	1.00	-0.13	0.039	0.0009641	0.0003385		0.506	0.97	0.022656	0.01845	0.2190903
rs12966945	18	10425574	C	T	0.535	1.00	-0.13	0.039	0.0009115	0.0003126		0.506	0.98	0.022402	0.01828	0.2201137
rs10468615	18	10425868	A	T	0.466	1.00	0.131	0.039	0.0008854	0.000304		0.494	0.98	-0.02241	0.0183	0.2203547
rs2115706	18	10426288	A	G	0.632	0.81	0.18	0.045	0.0000548	0.000006		0.650	0.64	-0.0253	0.0236	0.2833546
rs206439	18	10434655	C	T	0.468	0.87	-0.17	0.042	0.0000468	0.0000105		0.447	0.72	0.018284	0.02143	0.3932627
rs564991	18	10455054	A	C	0.619	0.90	0.14	0.042	0.0008569	0.0007535		0.651	0.91	0.004612	0.02023	0.8195317
rs17470203	18	24110579	A	C	0.058	0.52	0.397	0.116	0.0005914	0.0003201		0.072	0.53	-0.02417	0.04834	0.6167584
rs17448275	18	24165856	G	T	0.028	0.40	0.627	0.188	0.0008322	0.0001445		0.035	0.43	-0.02741	0.07012	0.6956621
rs12607637	18	25757491	C	T	0.226	0.72	-0.189	0.054	0.0004671	0.0005236		0.206	0.66	-0.03051	0.02824	0.2796356
rs2015085	18	25760060	A	C	0.842	1.00	0.192	0.053	0.0002908	0.0003905		0.854	1.00	0.032328	0.0266	0.2237902
rs12965207	18	25786830	C	T	0.839	1.00	0.193	0.053	0.0002388	0.0004021		0.852	1.00	0.033353	0.02624	0.2033597
rs9966721	18	25796043	C	T	0.839	1.00	0.193	0.053	0.0002328	0.0003888		0.852	1.00	0.033377	0.02625	0.2031155
rs7243787	18	25826032	C	T	0.834	1.00	0.204	0.052	0.0000835	0.0001139		0.847	0.99	0.034955	0.02561	0.1719075
rs12967036	18	25829752	A	G	0.166	1.00	-0.205	0.052	0.0000789	0.0001087		0.153	0.99	-0.03495	0.02561	0.1721155
rs7244533	18	25834901	C	T	0.739	1.00	0.15	0.044	0.0006305	0.0011295		0.744	1.00	0.040853	0.02059	0.0471191
rs7234178	18	25883206	A	T	0.177	0.98	-0.18	0.051	0.0004159	0.0009967		0.157	0.86	-0.01763	0.02732	0.5184363
rs12604702	18	25885783	A	C	0.165	1.00	-0.194	0.052	0.0001759	0.0002244		0.139	0.89	-0.02734	0.02831	0.3336994
rs12970785	18	25905770	A	C	0.799	0.91	0.188	0.05	0.0001747	0.0002699		0.810	0.86	0.012065	0.02544	0.6350407
rs1534063	18	25926647	A	G	0.165	1.00	-0.18	0.052	0.000482	0.0007444		0.150	1.00	-0.02214	0.02594	0.392985
rs1534064	18	25928058	A	G	0.165	1.00	-0.18	0.052	0.0004807	0.0007432		0.150	1.00	-0.02207	0.02594	0.3944898
rs9959395	18	25931667	C	T	0.835	1.00	0.181	0.052	0.0004699	0.0007345		0.850	1.00	0.021705	0.02594	0.402307
rs12968899	18	25934978	C	T	0.835	1.00	0.181	0.052	0.0004627	0.0007287		0.850	1.00	0.021682	0.02594	0.4027908
rs9961904	18	25945743	C	T	0.165	1.00	-0.181	0.052	0.0004601	0.0007259		0.150	1.00	-0.02177	0.02594	0.4008006
rs9960213	18	25968229	A	C	0.835	1.00	0.182	0.052	0.000426	0.0006831		0.851	1.00	0.022044	0.02593	0.394867
rs12969614	18	25969767	G	T	0.165	1.00	-0.182	0.052	0.0004208	0.0006767		0.150	1.00	-0.02207	0.02593	0.3944034
rs9959805	18	25977309	A	G	0.165	1.00	-0.182	0.052	0.0004167	0.0006694		0.150	1.00	-0.02232	0.02593	0.3890277
rs9956642	18	25983669	C	G	0.165	1.00	-0.183	0.052	0.0003949	0.0006401		0.150	1.00	-0.02343	0.02594	0.3660769
rs1554649	18	25988903	C	T	0.165	1.00	-0.183	0.052	0.0003932	0.0006367		0.150	1.00	-0.02352	0.02594	0.3640357
rs9959731	18	25989775	A	C	0.165	1.00	-0.183	0.052	0.0003909	0.000632		0.150	1.00	-0.02358	0.02594	0.3628283
rs4799794	18	25991647	A	G	0.165	1.00	-0.183	0.052	0.0003897	0.0006297		0.150	1.00	-0.02362	0.02594	0.3620589
rs12605594	18	25994245	C	T	0.835	1.00	0.184	0.052	0.0003839	0.000618		0.850	1.00	0.023721	0.02593	0.3599542
rs1547567	18	25998422	A	G	0.165	1.00	-0.184	0.052	0.0003825	0.0006154		0.150	1.00	-0.02373	0.02593	0.3597588
rs1547568	18	26000537	C	T	0.165	1.00	-0.184	0.052	0.0003766	0.0006075		0.150	1.00	-0.02375	0.02593	0.3593819
rs9962557	18	26011575	A	G	0.836	1.00	0.188	0.052	0.0003083	0.0005284		0.851	1.00	0.024023	0.02597	0.3546579
rs9304552	18	26027903	A	G	0.163	1.00	-0.191	0.052	0.0002549	0.0004775		0.149	1.00	-0.02407	0.02598	0.3538467
rs7232093	18	26044491	G	T	0.806	1.00	0.162	0.049	0.0009304	0.0016744		0.825	0.99	0.02254	0.02429	0.3529948
rs4393646	18	26044930	G	T	0.194	1.00	-0.162	0.049	0.0009354	0.0016856		0.175	0.99	-0.02252	0.0243	0.3536797
rs4527093	18	26045083	C	T	0.193	1.00	-0.162	0.049	0.000945	0.0017071		0.175	0.99	-0.02249	0.0243	0.3544544
rs16960876	18	26047775	C	T	0.166	0.99	-0.18	0.052	0.0005551	0.000855		0.154	0.96	-0.01978	0.0262	0.4499543
rs11664615	18	26169218	G	T	0.383	0.99	-0.136	0.04	0.0006208	0.0016792		0.394	0.99	0.005696	0.01878	0.761436
rs2419037	18	26180575	C	T	0.380	1.00	-0.139	0.04	0.0004675	0.0014271		0.393	0.98	0.006314	0.01882	0.7370516

					GOYA QC		GOYA Overweight/control			GOYA BMI continuous	known	IARC QC		IARC results		
MARKER	chr	position	A1	A2	FREQ1	Rsqr	Beta	SE	p	p	gene name	freq1	Rsqr	in_beta	in_SE	in_p
rs3107987	18	26189929	C	T	0.379	1.00	-0.141	0.04	0.000397	0.001144		0.396	0.94	0.007365	0.0192	0.7010537
rs12373195	18	27983601	G	T	0.639	0.91	-0.155	0.043	0.0002735	0.0007763		0.636	0.83	0.014298	0.02093	0.494176
rs4799915	18	33182637	C	T	0.325	0.99	-0.142	0.042	0.0006856	0.0028137		0.356	0.99	0.002628	0.01935	0.8918719
rs3865392	18	33183462	A	C	0.677	0.98	0.14	0.042	0.0008894	0.0022736		0.647	0.97	-0.00431	0.01957	0.8254007
rs3895875	18	33190880	C	T	0.827	1.00	0.172	0.052	0.0009627	0.0020124		0.818	1.00	-0.00096	0.02457	0.9689419
rs3865393	18	33191107	A	C	0.173	1.00	-0.172	0.052	0.000978	0.0020792		0.182	1.00	0.000902	0.02458	0.9706879
rs12605960	18	33288500	C	T	0.080	0.81	-0.276	0.08	0.0005722	0.000323		0.087	0.83	-0.0424	0.03655	0.2456141
rs9675529	18	33290211	C	T	0.928	0.80	0.296	0.085	0.0004768	0.0006603		0.918	0.82	0.050401	0.03755	0.1791791
rs948535	18	33291001	G	T	0.930	0.98	0.261	0.077	0.0007168	0.0008341		0.917	0.97	0.041144	0.03442	0.2315459
rs17750143	18	33292481	C	T	0.070	0.99	-0.26	0.077	0.0007265	0.0008505		0.083	0.97	-0.04113	0.03437	0.2310379
rs17682727	18	33303454	A	G	0.070	1.00	-0.262	0.077	0.0006389	0.00072		0.083	0.98	-0.03937	0.03413	0.2483751
rs7228860	18	33306970	A	C	0.274	1.00	-0.165	0.044	0.0001479	0.00011		0.295	0.99	0.011585	0.02071	0.5756329
rs11874426	18	33309754	C	T	0.726	1.00	0.167	0.043	0.0001222	0.0000808		0.705	0.99	-0.01187	0.02071	0.566206
rs9304173	18	33315195	A	G	0.929	1.00	0.261	0.076	0.0006184	0.0006024		0.916	1.00	0.035276	0.03383	0.2967106
rs1196943	18	34202297	C	G	0.393	0.91	-0.147	0.041	0.0003995	0.0063882		0.391	0.93	0.004872	0.01947	0.8022127
rs1196945	18	34202378	A	G	0.393	0.92	-0.146	0.041	0.0004016	0.0065287		0.391	0.93	0.004907	0.01946	0.8007539
rs1145555	18	34202653	C	T	0.385	0.98	-0.144	0.04	0.0003549	0.0062793		0.380	0.98	0.003695	0.01907	0.846249
rs1789783	18	34203455	C	T	0.616	0.99	0.142	0.04	0.00037	0.0066473		0.620	0.98	-0.00372	0.01906	0.8450919
rs977767	18	34204721	C	T	0.616	1.00	0.142	0.04	0.0003731	0.0067055		0.621	1.00	-0.00416	0.0189	0.8258789
rs1212408	18	34205790	G	T	0.287	1.00	-0.152	0.043	0.0004242	0.003844		0.304	0.94	0.00399	0.02048	0.8453783
rs1145557	18	34207568	A	C	0.616	1.00	0.142	0.04	0.0003742	0.0067373		0.621	1.00	-0.00417	0.01889	0.8250565
rs1145559	18	34208026	G	T	0.287	1.00	-0.151	0.043	0.0004263	0.0038776		0.304	0.94	0.004051	0.02046	0.8429139
rs1145560	18	34208173	C	T	0.616	1.00	0.142	0.04	0.0003686	0.0066896		0.621	1.00	-0.00421	0.01888	0.823256
rs1511944	18	34209724	C	T	0.616	1.00	0.142	0.04	0.0003689	0.0066948		0.621	1.00	-0.00421	0.01888	0.8234711
rs1511942	18	34210007	A	G	0.384	1.00	-0.142	0.04	0.0003692	0.0067001		0.379	1.00	0.004194	0.01888	0.8240653
rs1511941	18	34210044	A	G	0.616	1.00	0.142	0.04	0.0003695	0.0067047		0.621	1.00	-0.00418	0.01888	0.8247291
rs1789611	18	34211825	C	T	0.616	1.00	0.142	0.04	0.0003696	0.0067075		0.621	1.00	-0.00417	0.01888	0.8250334
rs1606249	18	34213526	C	T	0.384	1.00	-0.142	0.04	0.0003697	0.0067098		0.379	1.00	0.004165	0.01888	0.8252755
rs1196942	18	34213824	A	C	0.616	1.00	0.142	0.04	0.0003724	0.0068497		0.621	1.00	-0.00388	0.01889	0.8373282
rs1196941	18	34213837	A	C	0.615	1.00	0.142	0.04	0.0003781	0.0068602		0.621	1.00	-0.00413	0.01888	0.8267223
rs1145562	18	34214364	A	T	0.385	1.00	-0.141	0.04	0.000384	0.0069628		0.379	1.00	0.004107	0.01889	0.8277223
rs958801	18	34214924	C	T	0.615	1.00	0.141	0.04	0.0003871	0.0070151		0.621	1.00	-0.0041	0.01889	0.8281134
rs1145567	18	34218817	G	T	0.615	1.00	0.141	0.04	0.000387	0.007031		0.621	1.00	-0.00404	0.0189	0.830487
rs1511938	18	34221918	A	G	0.513	0.97	-0.167	0.039	0.0000204	0.0007049		0.507	0.99	0.001033	0.01827	0.9548991
rs1145569	18	34221998	C	T	0.384	0.97	-0.138	0.04	0.0006064	0.0107541		0.379	0.99	0.003967	0.01895	0.8340836
rs1196937	18	34223242	C	T	0.616	0.97	0.138	0.04	0.0006428	0.011314		0.621	0.99	-0.00389	0.01899	0.837391
rs1196598	18	34226467	C	T	0.616	0.97	0.137	0.04	0.0006647	0.0116412		0.621	0.97	-0.00355	0.0192	0.8531495
rs1209307	18	34229452	C	T	0.687	0.99	0.142	0.042	0.0007507	0.0175069		0.670	0.80	0.013939	0.02199	0.5258431
rs1196604	18	34230558	C	T	0.313	0.99	-0.142	0.042	0.0007648	0.0177937		0.330	0.80	-0.01394	0.022	0.5259738
rs1196606	18	34231066	G	T	0.687	0.99	0.142	0.042	0.0007804	0.0181274		0.671	0.80	0.013939	0.02207	0.5274013
rs1196607	18	34231098	C	T	0.313	0.99	-0.142	0.042	0.0007835	0.018176		0.330	0.80	-0.01393	0.02208	0.5276858
rs1196608	18	34231137	A	C	0.687	0.99	0.141	0.042	0.0007844	0.0181953		0.671	0.80	0.013937	0.02208	0.5276393
rs1789782	18	34231566	A	G	0.313	0.99	-0.141	0.042	0.0007852	0.0182119		0.329	0.79	-0.01392	0.02209	0.5280716
rs981531	18	34232514	C	T	0.684	0.99	0.149	0.042	0.0003949	0.012499		0.667	0.78	0.010949	0.02224	0.6222563
rs1621192	18	34233295	A	T	0.313	1.00	-0.141	0.042	0.0007942	0.0185685		0.328	0.79	-0.014	0.02223	0.5285445
rs2861744	18	34234162	A	G	0.313	1.00	-0.141	0.042	0.0007934	0.0185939		0.328	0.79	-0.01403	0.02226	0.5282224
rs1706317	18	34234526	C	T	0.687	1.00	0.141	0.042	0.0007949	0.01868		0.672	0.79	0.014035	0.02227	0.5281748
rs1546523	18	34234806	A	G	0.485	1.00	-0.162	0.039	0.0000256	0.0021268		0.474	0.82	-0.00057	0.0201	0.9772834
rs1196577	18	34236626	A	G	0.312	1.00	-0.141	0.042	0.0007847	0.0186467		0.327	0.78	-0.01417	0.02235	0.5256422
rs977153	18	34236916	C	T	0.688	1.00	0.141	0.042	0.0007793	0.0186279		0.673	0.78	0.014197	0.02236	0.525043
rs1196579	18	34238127	A	G	0.688	1.00	0.141	0.042	0.0007864	0.0187142		0.673	0.78	0.014249	0.0224	0.5243842
rs1196582	18	34239757	A	G	0.312	0.99	-0.142	0.042	0.000779	0.0184706		0.326	0.78	-0.01439	0.02248	0.5216692
rs1196583	18	34240310	A	G	0.312	0.99	-0.142	0.042	0.0007758	0.0183758		0.325	0.77	-0.01456	0.02256	0.5183039
rs1196584	18	34240557	C	T	0.688	0.98	0.143	0.042	0.0007642	0.0179946		0.676	0.77	0.01461	0.02258	0.5172426
rs11659348	18	34245474	A	G	0.411	0.98	0.148	0.04	0.0002265	0.0066741		0.412	0.99	0.00359	0.01878	0.8482643
rs11661910	18	34248029	A	T	0.591	0.99	-0.146	0.04	0.0002401	0.0075591		0.588	0.99	-0.00407	0.01874	0.8278078
rs4476251	18	34249171	C	T	0.591	0.99	-0.147	0.04	0.0002308	0.0074364		0.588	0.99	-0.00421	0.01873	0.8221649
rs1944800	18	34261411	A	G	0.397	1.00	-0.138	0.04	0.0004872	0.0039457		0.389	1.00	0.001091	0.01892	0.9540029
rs11082143	18	35431732	A	C	0.952	0.99	-0.305	0.092	0.0009847	0.0034301		0.946	0.97	0.006396	0.03745	0.8642626
rs1377341	18	36883715	A	G	0.363	0.99	-0.103	0.04	0.01066	0.0002915		0.349	0.98	-0.0288	0.01935	0.1363938
rs9947540	18	36883986	G	T	0.363	0.99	-0.103	0.04	0.01064	0.0002912		0.349	0.98	-0.02877	0.01935	0.1367814
rs11664452	18	36887072	A	G	0.291	0.99	-0.099	0.043	0.01997	0.0008881		0.264	1.00	-0.04736	0.02094	0.0236251
rs1453036	18	36890479	C	T	0.290	1.00	-0.099	0.043	0.01964	0.0009276		0.264	1.00	-0.04753	0.02094	0.0231162
rs2060913	18	36894276	C	T	0.290	1.00	-0.1	0.043	0.01883	0.0008614		0.264	1.00	-0.04774	0.02094	0.0225272
rs12373433	18	36895741	C	T	0.290	1.00	-0.1	0.043	0.01848	0.0008346		0.264	1.00	-0.04783	0.02094	0.0222661
rs4243274	18	36899016	A	G	0.705	1.00	0.103	0.042	0.01502	0.0009717		0.729	1.00	0.041168	0.02076	0.0472352
rs10853477	18	36905434	C	T	0.710	1.00	0.099	0.043	0.01967	0.0008948		0.736	1.00	0.048338	0.02093	0.0208179
rs12959140	18	36907805	A	G	0.710	0.99	0.1	0.043	0.01974	0.0009089		0.736	0.99	0.048607	0.021	0.0205614
rs12959352	18	38479277	A	G	0.872	0.99	0.231	0.058	0.0000709	0.0008635		0.864	0.61	0.016266	0.03459	0.6378546
rs617460	18	38771445	C	T	0.476	0.86	-0.122	0.042	0.00368	0.0007298		0.480	0.79	0.003637	0.02048	0.8589131
rs10468972	18	40689971	A	C	0.854	1.00	0.168	0.055	0.002274	0.0006724		0.844	0.98	-0.00461	0.02515	0.8543857
rs328168	18	42411418	G	T	0.866	0.91	0.211	0.06	0.0004558	0.0033987		0.878	0.90	-0.01562	0.02992	0.6011772
rs328166	18	42411504	G	T	0.533	0.95	-0.147	0.04	0.0002392	0.0004187		0.516	0.89	-0.00775	0.01946	0.6903127
rs328165	18	42412629	C	G	0.133	0.95	-0.209	0.059	0.0003967	0.0032749		0.122	0.90	0.015612	0.02985	0.6007236
rs167872	18	42414476	A	G	0.867	0.96	0.209	0.059	0.0003904	0.0032622		0.878	0.95	-0.01513	0.02906	0.602173
rs1818567	18	42415674	A	G	0.873	0.96	0.202	0.06	0.0007133	0.0058576		0.886	0.94	-0.00566	0.02999	0.850189
rs328161	18	42416848	A	T	0.132	0.97	-0.208	0.059	0.000379	0.0032502		0.122	0.96	0.015006	0.0289	0.6033486
rs655140	18	42418428	A	T	0.873	0.96	0.202	0.06	0.0007062	0.0058714		0.886	0.95	-0.00558	0.02982	0.8513355
rs328159	18	42418695	C	T	0.132	0.97	-0.208	0.059	0.000379	0.0032604		0.122	0.97	0.014966	0.02882	0.6032748
rs328158	18	42421693	A	C	0.868	0.99	0.207	0.058	0.0003685	0.0032437		0.878	0.98	-0.01488	0.02868	0.6036016
rs328157	18	42421840	C	T	0.868	0.99	0.207	0.058	0.0003692	0.0032501		0.878	0.99	-0.01475	0.02844	0.6037627
rs101941	18	42422441	A	G	0.207	1.00	-0.157	0.048	0.001095	0.0009764		0.212	1.00	0.003066	0.02221	0.8901159

					GOYA QC		GOYA Overweight/control			GOYA BMI continuous	known	IARC QC		IARC results		
MARKER	chr	position	A1	A2	FREQ1	Rsqr	Beta	SE	p	p	gene name	freq1	Rsqr	in_beta	in_SE	in_p
rs328155	18	42422942	C	T	0.132	0.99	-0.207	0.058	0.0003671	0.0032944		0.122	1.00	0.016111	0.02839	0.5700816
rs328154	18	42423142	A	G	0.868	0.99	0.207	0.058	0.0003667	0.0032989		0.878	1.00	-0.01619	0.02839	0.5680881
rs328153	18	42424206	C	T	0.131	1.00	-0.207	0.058	0.0003574	0.0035202		0.122	1.00	0.016354	0.02839	0.5643014
rs328152	18	42424283	C	G	0.131	1.00	-0.207	0.058	0.0003572	0.0035274		0.122	1.00	0.016393	0.02839	0.5633704
rs328151	18	42424344	A	G	0.869	1.00	0.207	0.058	0.0003565	0.0035527		0.878	1.00	-0.01643	0.02839	0.5624127
rs328150	18	42424383	C	T	0.131	1.00	-0.207	0.058	0.0003561	0.0035815		0.122	1.00	0.016916	0.02839	0.5509478
rs328149	18	42424469	A	G	0.869	1.00	0.208	0.058	0.0003346	0.0034501		0.878	1.00	-0.01735	0.02839	0.5407714
rs328147	18	42425462	A	G	0.869	1.00	0.208	0.058	0.00033	0.0034222		0.878	1.00	-0.01792	0.02839	0.5274256
rs328145	18	42427447	A	C	0.131	1.00	-0.209	0.058	0.0003168	0.0033399		0.122	1.00	0.018918	0.02838	0.5046505
rs328144	18	42428551	C	T	0.535	1.00	-0.143	0.039	0.000248	0.0003829		0.516	1.00	-0.00503	0.01843	0.7849776
rs328143	18	42428922	C	T	0.870	1.00	0.214	0.058	0.0002289	0.0027409		0.878	1.00	-0.01966	0.02837	0.4878639
rs328142	18	42430301	A	G	0.131	1.00	-0.214	0.058	0.0002234	0.002697		0.122	1.00	0.019753	0.02837	0.485955
rs328141	18	42430835	C	T	0.869	0.96	0.215	0.059	0.00027	0.0028734		0.878	0.99	-0.01982	0.02849	0.4863901
rs328140	18	42431139	C	T	0.131	0.96	-0.215	0.059	0.000278	0.002904		0.122	0.99	0.019823	0.02852	0.486625
rs328138	18	42432167	G	T	0.869	0.96	0.215	0.059	0.0002811	0.002918		0.878	0.99	-0.01982	0.02852	0.4868493
rs328137	18	42432594	A	G	0.869	0.95	0.215	0.059	0.0002847	0.0029382		0.878	0.98	-0.01987	0.02868	0.4880244
rs328136	18	42432624	A	T	0.869	0.95	0.215	0.059	0.0002896	0.0029627		0.878	0.98	-0.01988	0.0287	0.4881262
rs328135	18	42433706	A	C	0.868	0.94	0.214	0.059	0.0003082	0.0030377		0.878	0.98	-0.0199	0.02871	0.4879625
rs660618	18	42434693	C	T	0.873	0.90	0.203	0.061	0.0009442	0.0069074		0.886	0.94	-0.01098	0.02999	0.7141399
rs2044350	18	47974021	C	G	0.211	0.97	-0.164	0.048	0.0006732	0.0138974		0.194	0.97	0.001902	0.02413	0.9371379
rs4939693	18	47974848	C	T	0.789	0.97	0.163	0.048	0.000693	0.0141546		0.806	0.97	-0.00192	0.02413	0.9365199
rs10502980	18	49321617	A	C	0.192	0.82	0.106	0.053	0.04645	0.0008965		0.160	0.42	0.009032	0.0386	0.8148576
rs7242745	18	55783281	A	G	0.598	1.00	0.133	0.04	0.0007621	0.0099207	MC4R					
rs7229781	18	55784634	A	G	0.592	0.93	0.138	0.041	0.000729	0.0098324	MC4R					
rs3897645	18	55879927	C	T	0.550	0.94	-0.121	0.04	0.002428	0.0008454	MC4R					
rs4940927	18	55883669	A	G	0.291	1.00	0.175	0.042	0.0000359	0.00000447	MC4R					
rs7234864	18	55885837	C	T	0.720	0.99	-0.179	0.043	0.0000335	0.00000473	MC4R					
rs7231154	18	55889077	A	G	0.358	1.00	0.127	0.04	0.001515	0.0002019	MC4R					
rs7241535	18	55889582	G	T	0.279	0.99	0.179	0.043	0.0000335	0.00000482	MC4R					
rs11664369	18	55890052	C	T	0.721	0.99	-0.179	0.043	0.0000335	0.00000487	MC4R					
rs11664212	18	55890159	G	T	0.358	1.00	0.127	0.04	0.001511	0.0002048	MC4R					
rs11664327	18	55890603	C	T	0.366	0.98	0.129	0.04	0.001365	0.0002056	MC4R					
rs1942866	18	55892763	C	G	0.605	1.00	-0.122	0.039	0.002072	0.0007511	MC4R					
rs8086627	18	55894076	A	C	0.395	1.00	0.122	0.039	0.002079	0.0007573	MC4R					
rs7240566	18	55895169	A	G	0.722	1.00	-0.178	0.043	0.0000361	0.00000625	MC4R					
rs11520442	18	55895556	C	T	0.278	1.00	0.178	0.043	0.0000365	0.00000641	MC4R					
rs1942863	18	55896724	C	T	0.395	1.00	0.121	0.039	0.002096	0.0007789	MC4R					
rs12957347	18	55901994	C	T	0.278	1.00	0.178	0.043	0.0000369	0.00000659	MC4R					
rs4299252	18	55902165	A	T	0.722	1.00	-0.178	0.043	0.0000369	0.00000659	MC4R					
rs8091524	18	55902940	C	T	0.278	1.00	0.178	0.043	0.0000369	0.00000659	MC4R					
rs8084085	18	55904998	C	T	0.269	0.96	0.182	0.044	0.0000407	0.00000671	MC4R					
rs6567155	18	55906097	C	T	0.722	1.00	-0.178	0.043	0.000037	0.00000661	MC4R					
rs1942860	18	55907035	C	T	0.722	1.00	-0.178	0.043	0.0000358	0.00000646	MC4R					
rs8094501	18	55907713	A	G	0.641	1.00	-0.127	0.04	0.001494	0.0002313	MC4R					
rs11665227	18	55908608	A	G	0.359	1.00	0.127	0.04	0.001462	0.0002274	MC4R					
rs11662368	18	55908958	A	G	0.722	1.00	-0.179	0.043	0.0000332	0.00000616	MC4R					
rs8086895	18	55909615	A	G	0.648	0.98	-0.135	0.041	0.0008868	0.0001156	MC4R					
rs12956755	18	55910039	G	T	0.648	0.98	-0.135	0.041	0.0008817	0.0001151	MC4R					
rs1539952	18	55917492	A	G	0.721	0.99	-0.181	0.043	0.0000275	0.00000522	MC4R					
rs11660783	18	55918615	C	T	0.209	1.00	0.179	0.048	0.0001796	0.0001384	MC4R					
rs9966951	18	55926275	A	G	0.354	1.00	0.142	0.04	0.0003908	0.0000697	MC4R					
rs9951795	18	55928971	C	G	0.646	1.00	-0.142	0.04	0.0003918	0.0000699	MC4R					
rs948760	18	55932168	A	G	0.646	1.00	-0.142	0.04	0.0003922	0.00007	MC4R					
rs1893512	18	55938539	C	T	0.354	1.00	0.142	0.04	0.0003935	0.0000703	MC4R					
rs756190	18	55941032	A	T	0.646	1.00	-0.143	0.04	0.0003885	0.000069	MC4R					
rs6567157	18	55941205	G	T	0.354	1.00	0.143	0.04	0.000379	0.0000664	MC4R					
rs8087080	18	55942061	G	T	0.646	1.00	-0.143	0.04	0.0003745	0.0000652	MC4R					
rs1942880	18	55944189	C	T	0.646	1.00	-0.143	0.04	0.0003638	0.0000623	MC4R					
rs8084834	18	55944569	A	C	0.354	1.00	0.143	0.04	0.0003634	0.0000622	MC4R					
rs953442	18	55948465	C	T	0.278	0.99	0.189	0.043	0.0000118	0.00000242	MC4R					
rs952044	18	55949090	C	T	0.647	0.99	-0.144	0.04	0.0003551	0.0000597	MC4R					
rs1942859	18	55950429	A	T	0.354	0.99	0.144	0.04	0.0003542	0.0000595	MC4R					
rs17700144	18	55962962	A	G	0.246	0.96	0.19	0.046	0.0000342	0.0000171	MC4R					
rs6567160	18	55980115	C	T	0.270	1.00	0.202	0.044	0.00000369	0.000000437	MC4R					
rs663129	18	55989381	A	G	0.270	1.00	0.2	0.044	0.00000461	0.000000487	MC4R					
rs571312	18	55990749	A	C	0.270	1.00	0.199	0.044	0.00000462	0.000000488	MC4R					
rs523288	18	55999349	A	T	0.728	0.99	-0.198	0.044	0.00000579	0.000000612	MC4R					
rs2168711	18	55999511	C	T	0.272	0.99	0.198	0.044	0.00000582	0.000000616	MC4R					
rs12967135	18	56000003	A	G	0.272	0.99	0.198	0.044	0.00000584	0.000000618	MC4R					
rs538656	18	56001402	G	T	0.727	1.00	-0.194	0.043	0.00000779	0.000000848	MC4R					
rs17782313	18	56002077	C	T	0.273	1.00	0.194	0.043	0.00000787	0.000000858	MC4R					
rs10871777	18	56002743	A	G	0.727	1.00	-0.194	0.043	0.00000789	0.00000086	MC4R					
rs476828	18	56003567	C	T	0.274	1.00	0.194	0.043	0.00000791	0.000000862	MC4R					
rs11152213	18	56003928	A	C	0.727	1.00	-0.194	0.043	0.00000779	0.000000843	MC4R					
rs1942873	18	56006866	C	G	0.824	0.76	0.234	0.059	0.0000664	0.0000736	MC4R					
rs492443	18	56009782	A	G	0.696	0.99	-0.208	0.042	8.87E-07	0.000000051	MC4R					
rs8089364	18	56009809	C	T	0.302	0.99	0.209	0.042	7.17E-07	3.21E-08	MC4R					
rs12969709	18	56010543	A	C	0.303	1.00	0.208	0.042	7.49E-07	3.26E-08	MC4R					
rs921971	18	56012643	C	T	0.303	1.00	0.207	0.042	0.00000075	3.26E-08	MC4R					
rs1457489	18	56012941	A	G	0.303	1.00	0.207	0.042	7.63E-07	3.34E-08	MC4R					
rs12954782	18	56015072	C	G	0.697	1.00	-0.207	0.042	7.65E-07	3.35E-08	MC4R					
rs9947403	18	56020730	C	T	0.631	1.00	-0.144	0.04	0.0002802	0.0000222	MC4R					

					GOYA QC		GOYA Overweight/control			GOYA BMI continuous	known	IARC QC		IARC results		
MARKER	chr	position	A1	A2	FREQ1	Rsqr	Beta	SE	p	p	gene name	freq1	Rsqr	in_beta	in_SE	in_p
rs639407	18	56021159	A	G	0.629	1.00	-0.143	0.04	0.0003301	0.0000314	MC4R					
rs1942876	18	56022246	A	G	0.411	1.00	0.105	0.039	0.006722	0.0009856	MC4R					
rs996022	18	56023341	G	T	0.411	1.00	0.105	0.039	0.006702	0.0009872	MC4R					
rs8084515	18	56023429	A	T	0.631	1.00	-0.144	0.04	0.0002855	0.0000231	MC4R					
rs12955983	18	56023969	A	G	0.683	0.96	-0.207	0.042	0.00000087	0.000000056	MC4R					
rs11665563	18	56027014	C	T	0.697	1.00	-0.206	0.042	8.92E-07	4.39E-08	MC4R					
rs11663816	18	56027207	C	T	0.311	0.99	0.195	0.042	0.00000313	0.000000151	MC4R					
rs11664883	18	56028780	A	T	0.303	1.00	0.206	0.042	9.01E-07	4.47E-08	MC4R					
rs489693	18	56033767	A	C	0.348	1.00	0.155	0.04	0.0001154	0.0000145	MC4R					
rs12970134	18	56035730	A	G	0.303	1.00	0.205	0.042	0.00000103	5.35E-08	MC4R					
rs9958386	18	56041805	C	T	0.632	1.00	-0.146	0.04	0.0002619	0.0000236	MC4R					
rs8083289	18	56044598	C	G	0.698	1.00	-0.208	0.042	7.86E-07	4.02E-08	MC4R					
rs528074	18	56045103	G	T	0.370	1.00	0.144	0.04	0.000315	0.0000334	MC4R					
rs557416	18	56046039	A	G	0.631	1.00	-0.143	0.04	0.0003242	0.0000357	MC4R					
rs477181	18	56047018	G	T	0.631	1.00	-0.143	0.04	0.0003234	0.0000357	MC4R					
rs502933	18	56047454	A	C	0.369	1.00	0.143	0.04	0.0003227	0.0000356	MC4R					
rs17175643	18	56047722	C	T	0.699	1.00	-0.208	0.042	7.13E-07	3.65E-08	MC4R					
rs12960928	18	56048783	C	T	0.302	1.00	0.208	0.042	7.99E-07	4.39E-08	MC4R					
rs2219375	18	56049529	A	G	0.367	1.00	0.146	0.04	0.0002609	0.0000249	MC4R					
rs8089366	18	56049580	G	T	0.633	1.00	-0.146	0.04	0.0002602	0.000025	MC4R					
rs8091286	18	56052117	A	G	0.367	1.00	0.145	0.04	0.000275	0.0000263	MC4R					
rs2083	18	56052762	C	T	0.633	1.00	-0.145	0.04	0.0002748	0.0000263	MC4R					
rs7227739	18	56052930	G	T	0.367	1.00	0.145	0.04	0.0002748	0.0000263	MC4R					
rs12456624	18	56054066	G	T	0.633	1.00	-0.146	0.04	0.0002523	0.0000238	MC4R					
rs12964203	18	56054584	C	T	0.301	1.00	0.209	0.042	6.41E-07	3.32E-08	MC4R					
rs590654	18	56054991	A	G	0.369	1.00	0.144	0.04	0.0002933	0.0000328	MC4R					
rs590215	18	56055068	C	T	0.697	1.00	-0.208	0.042	7.93E-07	5.14E-08	MC4R					
rs534234	18	56055187	A	G	0.369	1.00	0.144	0.04	0.0002974	0.0000333	MC4R					
rs2168708	18	56058291	G	T	0.699	1.00	-0.209	0.042	6.99E-07	0.000000036	MC4R					
rs12966550	18	56062310	A	G	0.699	1.00	-0.208	0.042	7.43E-07	3.81E-08	MC4R					
rs9955666	18	56063765	A	G	0.364	0.99	0.148	0.04	0.0002353	0.0000247	MC4R					
rs4450508	18	56064414	A	G	0.387	0.99	0.131	0.04	0.0009503	0.0000987	MC4R					
rs12960049	18	57811663	A	G	0.051	0.96	0.275	0.089	0.002085	0.000592		0.059	0.86	-0.04003	0.04113	0.3300441
rs17677569	18	58949590	C	G	0.064	0.97	-0.242	0.08	0.002692	0.0002265		0.077	0.88	-0.00391	0.03909	0.9202868
rs4987825	18	58963493	G	T	0.938	1.00	0.232	0.08	0.003949	0.000311		0.932	1.00	-0.0041	0.03935	0.9169404
rs4987821	18	58964195	C	T	0.938	1.00	0.231	0.08	0.00405	0.0003145		0.932	1.00	-0.00386	0.03934	0.9217681
rs17678177	18	58968859	C	G	0.064	0.97	-0.229	0.081	0.004448	0.0003096		0.073	0.91	0.000507	0.03975	0.989826
rs17756365	18	58969792	C	T	0.027	0.57	-0.451	0.16	0.004722	0.0007672		0.028	0.42	-0.04251	0.0945	0.6525326
rs1944268	18	59798459	A	T	0.269	0.97	-0.148	0.045	0.0009248	0.0040433		0.333	0.86	0.022387	0.02081	0.2815693
rs638006	18	63658055	A	G	0.637	1.00	0.131	0.04	0.0009606	0.0072036		0.651	0.87	0.013872	0.02039	0.4959626
rs636662	18	63658346	A	T	0.363	1.00	-0.131	0.04	0.0009583	0.007194		0.348	0.88	-0.01433	0.02027	0.4792388
rs504131	18	63659309	C	T	0.363	1.00	-0.132	0.04	0.0009461	0.0071398		0.345	0.91	-0.01512	0.02002	0.4497606
rs569775	18	63663388	A	G	0.638	0.99	0.133	0.04	0.0008543	0.006734		0.658	0.94	0.01618	0.0197	0.4111614
rs565364	18	63663799	G	T	0.362	0.99	-0.134	0.04	0.0008518	0.006723		0.342	0.94	-0.01622	0.0197	0.4099765
rs2613164	18	63666062	C	T	0.361	0.99	-0.136	0.04	0.0006741	0.0056915		0.342	0.95	-0.01726	0.01959	0.3779946
rs580923	18	63687106	A	G	0.349	1.00	-0.138	0.04	0.0005968	0.0081		0.332	0.99	-0.0171	0.0193	0.3750228
rs12969376	18	63687277	C	T	0.610	0.97	0.132	0.04	0.0009595	0.0052229		0.640	0.97	0.021582	0.01906	0.2571844
rs8084950	18	63688332	G	T	0.402	1.00	-0.13	0.039	0.0009686	0.0049753		0.371	1.00	-0.02118	0.01863	0.2551836
rs11151548	18	65773325	G	T	0.886	0.70	0.202	0.073	0.005923	0.0008046		0.864	0.61	-0.08672	0.03534	0.0140677
rs17230566	18	65818011	C	T	0.807	0.94	0.141	0.051	0.005403	0.0004478		0.779	0.89	-0.05419	0.02331	0.0200401
rs4525583	18	69216811	A	T	0.179	0.81	0.206	0.056	0.0002359	0.000175						
rs1943807	18	69218106	C	T	0.215	0.96	0.159	0.048	0.0009438	0.0014199						
rs744142	18	71004315	C	T	0.104	0.98	-0.273	0.064	0.000021	0.0013914						
rs596996	18	71004971	C	T	0.104	0.99	-0.27	0.064	0.000022	0.0014288						
rs17056473	18	71005940	A	G	0.961	0.51	0.578	0.143	0.0000499	0.0007642						
rs12604612	18	72999465	C	T	0.646	0.67	0.165	0.049	0.0008369	0.0101072						
rs618409	19	315695	G	T	0.604	0.99	-0.083	0.04	0.0375	0.0007961		0.560	0.80	-0.01466	0.0203	0.4699175
rs689448	19	317451	A	C	0.382	1.00	0.098	0.04	0.01355	0.0001609		0.401	0.99	0.015082	0.01848	0.4139207
rs585487	19	318313	A	G	0.570	1.00	0.096	0.039	0.01489	0.0007988		0.589	0.99	0.005836	0.01835	0.7503252
rs13004	19	5866594	A	G	0.200	0.87	0.165	0.052	0.001538	0.0007786		0.161	0.76	-0.04029	0.02828	0.1539155
rs11672480	19	6149291	C	T	0.040	0.62	-0.39	0.128	0.002321	0.0009514		0.059	0.63	-0.06181	0.05111	0.2261489
rs11666589	19	6545602	C	T	0.137	1.00	0.195	0.056	0.0004836	0.001649		0.106	1.00	0.020219	0.02908	0.4864612
rs168259	19	6546779	A	G	0.137	1.00	0.195	0.056	0.0004742	0.0016071		0.106	1.00	0.020316	0.02906	0.484058
rs7254060	19	7234414	A	G	0.059	1.00	0.268	0.083	0.001167	0.0003844		0.072	0.99	-0.08658	0.03747	0.0207601
rs11673642	19	7235597	A	T	0.031	0.76	0.442	0.13	0.0006561	0.0005793		0.034	0.69	-0.14321	0.06672	0.0317303
rs265539	19	17784160	C	G	0.770	0.84	0.156	0.05	0.001901	0.0009286		0.762	0.25	0.041094	0.04389	0.3487026
rs273266	19	18144501	C	T	0.223	1.00	0.122	0.046	0.008734	0.0001204		0.245	1.00	-0.00815	0.02078	0.6945112
rs2241090	19	18146751	C	G	0.223	1.00	0.121	0.046	0.009086	0.0001345		0.245	1.00	-0.00821	0.02079	0.6926328
rs7125	19	18149069	A	G	0.517	0.99	-0.097	0.039	0.01259	0.0006344		0.453	0.99	0.018571	0.01803	0.3024887
rs1045747	19	18149898	C	T	0.223	1.00	0.121	0.046	0.009157	0.0001384		0.245	1.00	-0.00823	0.0208	0.691956
rs12461975	19	21653948	A	C	0.053	0.87	-0.337	0.094	0.0003215	0.0003166		0.037	0.66	-0.05444	0.06434	0.3971314
rs12463030	19	21739447	C	T	0.047	0.99	-0.356	0.094	0.000146	0.0001197		0.026	0.71	-0.03906	0.07623	0.6080557
rs17701305	19	21739860	A	G	0.954	0.99	0.356	0.094	0.000146	0.00012		0.974	0.71	0.039291	0.07633	0.606437
rs11085493	19	21740588	C	T	0.943	0.98	0.286	0.084	0.0007076	0.0003107		0.954	0.75	0.039188	0.05563	0.4807838
rs2915922	19	22505936	C	G	0.576	0.97	0.139	0.04	0.0004715	0.0011536		0.549	0.77	0.005594	0.02067	0.7865196
rs11879691	19	34082694	C	T	0.362	0.97	-0.139	0.041	0.0006965	0.0106621		0.340	0.90	0.017045	0.02043	0.4037329
rs8108438	19	34082713	C	T	0.493	0.98	-0.158	0.039	0.0000552	0.0017012		0.487	0.94	0.014245	0.01917	0.4570306
rs8108541	19	34082756	C	T	0.495	1.00	-0.149	0.039	0.0001312	0.0026557		0.494	0.92	0.014589	0.01929	0.4491393
rs8109135	19	34082927	A	G	0.493	1.00	-0.157	0.039	0.0000531	0.0016199		0.486	0.96	0.014113	0.01898	0.4567484
rs734145	19	34084032	C	T	0.638	1.00	0.137	0.041	0.0006981	0.0103457		0.661	0.98	-0.01641	0.01963	0.4027514
rs734146	19	34084045	A	T	0.493	1.00	-0.158	0.039	0.0000487	0.001553		0.486	0.98	0.013931	0.01876	0.4573623
rs7252491	19	34084586	A	C	0.507	1.00	0.158	0.039	0.0000481	0.0015439		0.514	1.00	-0.0138	0.01856	0.4568675

					GOYA QC		GOYA Overweight/control			GOYA BMI continuous	known	IARC QC		IARC results		
MARKER	chr	position	A1	A2	FREQ1	Rsqr	Beta	SE	p	p	gene name	freq1	Rsqr	in_beta	in_SE	in_p
rs4805297	19	34086204	A	G	0.505	1.00	0.15	0.039	0.000115	0.0024747		0.506	0.97	-0.01409	0.01881	0.453597
rs17772698	19	34087398	A	G	0.493	1.00	-0.158	0.039	0.0000471	0.0015187		0.486	1.00	0.013725	0.01857	0.4593644
rs8107677	19	34087454	C	T	0.507	1.00	0.158	0.039	0.0000467	0.0015107		0.514	1.00	-0.01367	0.01857	0.4612387
rs8103259	19	34087463	A	G	0.507	1.00	0.158	0.039	0.0000464	0.0015027		0.514	1.00	-0.01363	0.01857	0.4624717
rs8106631	19	34087888	A	G	0.638	1.00	0.138	0.041	0.0006834	0.0101076		0.661	1.00	-0.0163	0.0194	0.4002487
rs4805298	19	34088184	C	T	0.493	1.00	-0.158	0.039	0.000046	0.0014911		0.487	1.00	0.01349	0.01856	0.4670027
rs4805299	19	34088669	G	T	0.638	1.00	0.138	0.041	0.0006816	0.0100691		0.661	1.00	-0.01634	0.01939	0.3990378
rs889349	19	34089098	A	G	0.638	1.00	0.138	0.041	0.0006819	0.0100819		0.661	1.00	-0.01634	0.01939	0.39919
rs889350	19	34089180	A	G	0.362	1.00	-0.138	0.041	0.0006821	0.0100939		0.339	1.00	0.01633	0.01939	0.3994003
rs1423703	19	34090466	G	T	0.335	0.91	-0.152	0.043	0.0003987	0.0090909		0.309	0.88	0.021213	0.02101	0.3123744
rs1423702	19	34090471	G	T	0.362	1.00	-0.138	0.041	0.0006827	0.0101247		0.339	1.00	0.016297	0.01939	0.4003336
rs1423701	19	34090561	C	T	0.362	1.00	-0.138	0.041	0.0006832	0.0101389		0.339	1.00	0.016282	0.01939	0.4007579
rs7507702	19	34090695	C	T	0.493	1.00	-0.158	0.039	0.0000486	0.0015026		0.487	1.00	0.013553	0.01856	0.4649701
rs6509122	19	34091492	C	T	0.362	1.00	-0.138	0.041	0.0006846	0.0101842		0.339	1.00	0.016243	0.01939	0.4018649
rs2113129	19	34091532	C	T	0.362	1.00	-0.138	0.041	0.000685	0.0101923		0.339	1.00	0.016231	0.01939	0.4022171
rs10417077	19	34091857	G	T	0.362	1.00	-0.138	0.041	0.0006855	0.0102072		0.339	1.00	0.016208	0.01939	0.402862
rs10417120	19	34091909	A	G	0.638	1.00	0.138	0.041	0.0006861	0.0102233		0.661	1.00	-0.0162	0.01939	0.4032101
rs10419624	19	34091986	A	G	0.362	1.00	-0.138	0.041	0.0006864	0.0102313		0.339	1.00	0.016179	0.01939	0.4036597
rs8112381	19	34092886	A	G	0.641	0.99	0.137	0.041	0.0008252	0.0123138		0.664	0.99	-0.01746	0.01954	0.3711846
rs979628	19	34093360	C	T	0.638	1.00	0.138	0.041	0.0006847	0.0102068		0.661	1.00	-0.01614	0.01939	0.404796
rs979629	19	34093535	C	G	0.638	1.00	0.138	0.041	0.0006828	0.0101739		0.661	1.00	-0.01612	0.01939	0.4052052
rs2902965	19	34093608	C	T	0.638	1.00	0.138	0.041	0.0006809	0.0101401		0.661	1.00	-0.01611	0.01939	0.4056329
rs1423699	19	34093875	A	G	0.362	1.00	-0.138	0.041	0.0006865	0.0101872		0.339	1.00	0.016101	0.01939	0.405856
rs1345832	19	34095410	A	C	0.638	1.00	0.138	0.041	0.0006893	0.0102121		0.661	1.00	-0.01608	0.01939	0.4065912
rs1345831	19	34095540	G	T	0.638	1.00	0.138	0.041	0.0006949	0.0102609		0.661	1.00	-0.01603	0.01941	0.4085474
rs12974259	19	34096487	A	T	0.638	1.00	0.137	0.041	0.0007005	0.0103082		0.661	1.00	-0.01602	0.01941	0.4087976
rs889356	19	34096819	C	T	0.638	1.00	0.137	0.041	0.0007089	0.0103799		0.661	1.00	-0.01602	0.01941	0.4088913
rs889357	19	34096891	C	T	0.362	1.00	-0.137	0.041	0.000715	0.010433		0.339	1.00	0.015998	0.01941	0.4094948
rs1991016	19	34099083	C	T	0.638	1.00	0.137	0.041	0.0007238	0.0105074		0.661	1.00	-0.016	0.01942	0.4095158
rs2161623	19	34099407	C	T	0.362	1.00	-0.137	0.041	0.0007326	0.0105796		0.339	1.00	0.015987	0.01942	0.409942
rs1345833	19	34099579	A	G	0.362	1.00	-0.137	0.041	0.0007415	0.0106544		0.339	0.99	0.015944	0.01943	0.4114469
rs889359	19	34100237	C	T	0.638	1.00	0.137	0.041	0.0007597	0.0108044		0.661	0.99	-0.01593	0.01943	0.4120615
rs12709873	19	34100402	C	T	0.362	1.00	-0.136	0.041	0.0007956	0.0111022		0.339	0.99	0.015922	0.01943	0.4122224
rs7359994	19	34100698	C	T	0.638	1.00	0.136	0.041	0.0008228	0.0113219		0.661	0.99	-0.01584	0.01945	0.4149961
rs889344	19	34101019	C	G	0.638	1.00	0.136	0.041	0.0008261	0.0113474		0.661	0.99	-0.01583	0.01946	0.4155605
rs4804859	19	34104893	C	T	0.530	0.99	0.135	0.039	0.0005515	0.0176533		0.521	0.98	-0.00511	0.0185	0.7820557
rs17690917	19	34128496	C	T	0.735	0.95	0.16	0.045	0.0004057	0.0089003		0.768	0.93	-0.03687	0.02157	0.0872134
rs1368481	19	34140864	C	T	0.709	0.97	0.144	0.043	0.0009662	0.0219452		0.749	1.00	-0.03324	0.02042	0.1033488
rs4513216	19	34147430	A	G	0.292	0.97	-0.144	0.043	0.0009401	0.0221068		0.254	0.98	0.032765	0.02054	0.1104913
rs12460607	19	34149925	A	C	0.708	0.97	0.144	0.044	0.0009374	0.0221591		0.746	0.98	-0.03267	0.02056	0.1119071
rs10425144	19	34164657	C	T	0.505	0.94	-0.133	0.04	0.0009334	0.002203		0.470	0.91	0.029108	0.01911	0.1273724
rs8102754	19	34180882	A	T	0.577	0.93	0.17	0.041	0.000031	0.0014136		0.566	0.84	-0.02868	0.0201	0.1533104
rs17691054	19	34182459	C	T	0.591	0.99	-0.141	0.04	0.0004167	0.0081541		0.610	0.84	0.035141	0.0208	0.0909606
rs892176	19	34188203	A	G	0.591	0.99	-0.141	0.04	0.0004154	0.0081416		0.610	0.83	0.035347	0.02088	0.0902376
rs16964099	19	35517408	C	T	0.767	0.98	0.17	0.047	0.0002631	0.0046031		0.747	0.97	-0.01802	0.02116	0.3939227
rs12977050	19	35520535	A	G	0.221	0.98	-0.178	0.047	0.0001561	0.0021503		0.237	0.99	0.014314	0.0214	0.5031681
rs10406668	19	35539979	C	T	0.847	0.97	-0.207	0.055	0.0001551	0.0008143		0.822	0.80	0.011553	0.02627	0.6598746
rs4805554	19	35540980	C	T	0.163	1.00	0.192	0.053	0.0002627	0.0019935		0.183	0.77	-0.01581	0.02612	0.5447006
rs33430	19	35631283	A	G	0.360	1.00	-0.14	0.04	0.0005178	0.0232746		0.356	1.00	0.008724	0.01884	0.6430843
rs33431	19	35631829	C	T	0.369	1.00	-0.133	0.04	0.0009306	0.0356393		0.360	1.00	0.008633	0.01876	0.6451124
rs33432	19	35632270	C	T	0.640	1.00	0.141	0.04	0.0004744	0.0218063		0.644	0.99	-0.0087	0.01888	0.6446259
rs33435	19	35635295	A	G	0.623	1.00	0.163	0.04	0.0000445	0.0021744		0.572	1.00	0.005928	0.01837	0.7467398
rs33436	19	35635517	A	G	0.364	0.99	-0.164	0.04	0.0000533	0.004755		0.416	0.97	7.58E-06	0.01877	0.9996773
rs33439	19	35637011	C	T	0.375	0.97	-0.164	0.041	0.0000552	0.0028829		0.428	0.97	-0.00565	0.01858	0.7609464
rs33442	19	35640082	C	T	0.706	0.88	0.151	0.045	0.0007568	0.0274003		0.657	0.83	-0.00553	0.02134	0.7953492
rs12459372	19	35640797	A	G	0.755	1.00	0.195	0.045	0.0000157	0.0005372		0.806	0.81	-0.04661	0.02529	0.0651889
rs759952	19	35641183	A	G	0.761	0.97	0.201	0.046	0.0000139	0.0002133		0.795	0.87	-0.04611	0.0243	0.0575597
rs3786814	19	35643118	A	G	0.171	1.00	-0.182	0.051	0.0003784	0.0034753		0.123	1.00	0.071602	0.02809	0.0107538
rs3786813	19	35643218	A	G	0.772	0.99	0.196	0.046	0.0000259	0.0004389		0.805	0.93	-0.05477	0.02406	0.0227156
rs3786812	19	35643240	C	T	0.264	1.00	-0.179	0.044	0.0000498	0.0012201		0.269	0.98	0.034552	0.02094	0.0986187
rs9304822	19	35643934	A	T	0.228	0.99	-0.196	0.047	0.0000257	0.0004343		0.194	0.93	0.054706	0.02413	0.0232883
rs12985250	19	35644025	A	G	0.171	1.00	-0.183	0.051	0.0003672	0.0033213		0.121	0.97	0.070911	0.02863	0.0131868
rs12985528	19	35644200	A	G	0.171	0.99	-0.184	0.051	0.0003453	0.0030532		0.121	0.96	0.070796	0.02866	0.013455
rs10460186	19	35644397	A	C	0.224	0.96	-0.202	0.047	0.0000211	0.0002662		0.187	0.93	0.051771	0.02458	0.03503
rs3786811	19	35644646	C	T	0.225	1.00	-0.188	0.046	0.0000538	0.0004835		0.193	0.94	0.043054	0.02404	0.0731086
rs14810	19	38996743	C	G	0.303	0.92	-0.174	0.044	0.0000762	0.000388	KCTD15					
rs185350	19	38998656	C	T	0.484	1.00	-0.134	0.038	0.0004707	0.0041755	KCTD15					
rs29944	19	38998738	A	G	0.319	1.00	-0.164	0.042	0.0000802	0.0003585	KCTD15					
rs29943	19	39000050	C	T	0.681	1.00	0.166	0.042	0.0000705	0.0003297	KCTD15					
rs29942	19	39001117	A	G	0.319	1.00	-0.167	0.042	0.0000634	0.0003048	KCTD15					
rs29941	19	39001372	A	G	0.319	1.00	-0.167	0.042	0.0000634	0.0003049	KCTD15					
rs29939	19	39002640	A	G	0.319	1.00	-0.167	0.042	0.000063	0.0003026	KCTD15					
rs29938	19	39003321	C	T	0.670	0.99	0.156	0.041	0.0001564	0.0010003	KCTD15					
rs29937	19	39003788	G	T	0.346	0.95	-0.169	0.042	0.0000508	0.0004171	KCTD15					
rs256335	19	39007736	C	T	0.517	1.00	-0.143	0.038	0.0002032	0.000891	KCTD15					
rs368794	19	39012292	A	T	0.661	0.95	0.16	0.042	0.0001408	0.0003121	KCTD15					
rs2546057	19	39012694	A	C	0.519	1.00	-0.143	0.038	0.0002053	0.0008723	KCTD15					
rs11084753	19	39013977	A	G	0.339	0.95	-0.16	0.042	0.0001368	0.000307	KCTD15					
rs415237	19	39015054	C	T	0.630	0.93	0.173	0.042	0.0000326	0.0002197	KCTD15					
rs17326595	19	39025686	C	T	0.340	0.91	-0.141	0.043	0.0009556	0.0082397	KCTD15					
rs2377288	19	39039582	A	G	0.213	0.49	-0.273	0.068	0.0000667	0.0006492	KCTD15					
rs17326700	19	39044611	A	G	0.149	0.47	-0.265	0.08	0.0008979	0.0108128	KCTD15					

					GOYA QC		GOYA Overweight/control			GOYA BMI continuous	known	IARC QC		IARC results		
MARKER	chr	position	A1	A2	FREQ1	Rsqr	Beta	SE	p	p	gene name	freq1	Rsqr	in_beta	in_SE	in_p
rs10406069	19	40528370	A	G	0.214	0.99	0.167	0.048	0.0005014	0.0004831		0.177	0.99	-0.00583	0.0238	0.8064574
rs2013666	19	51478966	A	G	0.931	0.98	-0.265	0.077	0.0006055	0.0000789		0.907	0.97	-0.01244	0.03196	0.6968923
rs4803920	19	51486612	C	G	0.069	0.90	0.276	0.081	0.0006157	0.0000804		0.090	0.93	0.018499	0.03308	0.5756847
rs11083841	19	51811873	A	G	0.831	1.00	0.139	0.052	0.007597	0.0005499		0.808	1.00	0.015804	0.02384	0.5069926
rs8105279	19	56706239	C	T	0.869	0.98	0.203	0.059	0.0005342	0.0007146		0.913	0.98	-0.00861	0.03123	0.782576
rs16982764	19	56709299	C	T	0.128	1.00	-0.204	0.059	0.000525	0.0007689		0.087	1.00	0.006422	0.03102	0.8358711
rs7247380	19	56712588	A	G	0.121	1.00	-0.216	0.06	0.0003458	0.0003397		0.086	1.00	0.00352	0.03119	0.9100741
rs11084422	19	61240204	C	T	0.735	1.00	-0.146	0.044	0.0008457	0.0041063		0.790	0.92	0.012451	0.0226	0.5813738
rs10415951	19	63111686	C	T	0.395	0.97	-0.149	0.04	0.0001695	0.0039168		0.386	0.99	0.023152	0.0189	0.2201996
rs8108626	19	63124308	C	T	0.393	1.00	-0.145	0.039	0.0002256	0.0048699		0.386	1.00	0.023392	0.01886	0.214527
rs1544930	19	63127832	C	T	0.683	0.95	0.146	0.042	0.0005777	0.0022819		0.685	0.97	-0.02581	0.02028	0.2027895
rs3760622	19	63139716	C	G	0.607	1.00	0.145	0.039	0.0002239	0.004685		0.615	1.00	-0.02316	0.01889	0.2198592
rs257656	19	63152020	A	T	0.671	1.00	0.14	0.041	0.0007113	0.0027103		0.676	1.00	-0.02487	0.0197	0.2063445
rs257662	19	63156441	C	T	0.329	1.00	-0.14	0.041	0.0006973	0.0026158		0.323	0.99	0.024126	0.0198	0.2227136
rs257664	19	63157276	C	T	0.672	1.00	0.14	0.041	0.0006817	0.0025088		0.677	0.99	-0.02397	0.01982	0.2261109
rs8105738	19	63187472	C	G	0.677	1.00	0.137	0.041	0.0009737	0.0052695		0.682	0.88	-0.02333	0.02106	0.2674712
rs11880378	19	63192083	G	T	0.677	1.00	0.137	0.041	0.0009584	0.0052154		0.681	0.87	-0.02359	0.02116	0.2644386
rs11668979	19	63193763	C	G	0.323	1.00	-0.137	0.041	0.0009501	0.0051862		0.319	0.87	0.023662	0.02118	0.2636423
rs4801538	19	63198049	C	T	0.323	1.00	-0.137	0.041	0.0009377	0.0051427		0.328	0.77	0.026847	0.02236	0.2296012
rs7245862	19	63198596	C	T	0.323	1.00	-0.137	0.041	0.000928	0.0051094		0.328	0.76	0.027009	0.02242	0.2280616
rs3088169	19	63201168	C	G	0.677	1.00	0.137	0.041	0.0009242	0.0050961		0.672	0.76	-0.02703	0.02243	0.2278248
rs7248365	19	63202315	C	T	0.677	1.00	0.137	0.041	0.0009101	0.0050474		0.667	0.71	-0.029	0.0231	0.208886
rs1035522	19	63202744	C	T	0.677	1.00	0.137	0.041	0.0009032	0.0050237		0.666	0.70	-0.02942	0.02323	0.2051241
rs10411019	19	63208042	C	T	0.672	0.99	0.139	0.041	0.0008359	0.0046868		0.644	0.62	-0.03446	0.02443	0.1580683
rs2076651	20	2421885	A	G	0.018	0.46	0.577	0.22	0.008771	0.0009124		0.016	0.22	0.078337	0.14111	0.5784704
rs297733	20	4357955	G	T	0.805	0.94	0.175	0.051	0.0006205	0.0003616		0.762	0.90	-0.0186	0.02254	0.4088989
rs172471	20	4358190	A	G	0.799	0.94	0.166	0.051	0.001077	0.0007197		0.751	0.90	-0.02217	0.02223	0.3182888
rs16989646	20	4367244	C	T	0.838	0.96	0.176	0.054	0.001222	0.0006711		0.798	0.94	-0.02382	0.02319	0.3039164
rs6052568	20	4372494	A	G	0.203	0.94	-0.174	0.05	0.000491	0.0002074		0.241	0.93	0.029074	0.02175	0.1810212
rs2014101	20	7518459	A	G	0.129	1.00	0.195	0.058	0.0007773	0.0107112		0.149	1.00	-0.03994	0.02539	0.1154394
rs2207696	20	7521011	A	G	0.129	1.00	0.194	0.058	0.0008293	0.011109		0.149	1.00	-0.03943	0.02539	0.1201649
rs6055170	20	7536116	C	T	0.130	0.99	0.196	0.058	0.0007363	0.0110374		0.152	0.98	-0.03801	0.0254	0.1342611
rs971060	20	7536599	A	T	0.871	0.99	-0.192	0.058	0.0009008	0.0115795		0.851	1.00	0.038558	0.02538	0.128359
rs2223532	20	7539479	C	T	0.129	0.99	0.193	0.058	0.0008747	0.0115245		0.150	0.99	-0.03779	0.02534	0.1356828
rs2206397	20	7539596	C	T	0.871	0.99	-0.193	0.058	0.0008746	0.0115285		0.850	0.99	0.037744	0.02534	0.1360726
rs6055180	20	7542928	A	G	0.871	1.00	-0.193	0.058	0.0008725	0.0116215		0.850	0.99	0.037414	0.02533	0.1392814
rs6055182	20	7544750	A	T	0.871	0.99	-0.193	0.058	0.0008851	0.0117918		0.850	0.99	0.037355	0.02534	0.1400673
rs6086122	20	7551590	A	G	0.133	1.00	0.196	0.058	0.0006381	0.0204451		0.148	0.95	-0.01362	0.02592	0.5990045
rs6117887	20	7553773	C	G	0.133	1.00	0.196	0.058	0.0006677	0.0212125		0.148	0.95	-0.01302	0.02581	0.6135964
rs2223531	20	7562537	A	G	0.132	1.00	0.192	0.057	0.0008144	0.0251874		0.151	0.99	-0.01054	0.02517	0.6750392
rs1040479	20	7565221	A	G	0.132	1.00	0.192	0.057	0.0008226	0.0263639		0.152	0.99	-0.01016	0.02504	0.6847752
rs4813836	20	7568823	C	T	0.868	1.00	-0.192	0.057	0.000815	0.0265091		0.848	1.00	0.010199	0.02503	0.6833538
rs6086136	20	7569465	C	T	0.132	1.00	0.193	0.057	0.000809	0.0261585		0.152	1.00	-0.01022	0.02502	0.682721
rs6077236	20	7573986	C	T	0.132	1.00	0.193	0.057	0.0007814	0.0243751		0.152	1.00	-0.01144	0.02498	0.6466812
rs6086144	20	7574342	C	T	0.868	1.00	-0.193	0.057	0.0007815	0.0243779		0.848	1.00	0.011681	0.02497	0.6396952
rs2050081	20	7575224	A	G	0.132	1.00	0.193	0.057	0.0007814	0.024376		0.153	1.00	-0.0118	0.02497	0.6362237
rs2223535	20	7579706	A	G	0.132	1.00	0.193	0.057	0.0007776	0.0244733		0.153	1.00	-0.01154	0.02498	0.6437222
rs2206406	20	7580355	A	G	0.132	1.00	0.193	0.057	0.0007769	0.0244946		0.153	1.00	-0.01152	0.02498	0.6444588
rs6055231	20	7581349	C	T	0.132	1.00	0.193	0.057	0.0007764	0.0245191		0.153	1.00	-0.01143	0.02498	0.6470772
rs6086146	20	7581484	A	C	0.132	1.00	0.193	0.057	0.000776	0.0245426		0.153	1.00	-0.01138	0.02498	0.6483553
rs4278998	20	7582063	A	T	0.132	1.00	0.193	0.057	0.0007755	0.0245663		0.153	1.00	-0.01133	0.02498	0.6498479
rs6038913	20	7583654	A	G	0.868	1.00	-0.193	0.057	0.0007745	0.0246291		0.847	1.00	0.011216	0.02499	0.6532349
rs6055235	20	7584126	G	T	0.868	1.00	-0.193	0.057	0.000774	0.0246694		0.847	1.00	0.011122	0.02499	0.6559919
rs2223534	20	7587093	G	T	0.132	1.00	0.193	0.057	0.0007735	0.0248042		0.154	0.99	-0.00939	0.02496	0.7063689
rs6055240	20	7592199	G	T	0.868	1.00	-0.193	0.057	0.0007665	0.0248176		0.845	0.99	0.009074	0.02495	0.7158416
rs6055241	20	7592651	C	T	0.868	1.00	-0.193	0.057	0.0007648	0.0248107		0.845	0.99	0.009053	0.02495	0.7164887
rs1474673	20	7600458	A	T	0.867	1.00	-0.196	0.057	0.0006585	0.0305878		0.856	0.93	0.005364	0.02632	0.8383738
rs4813838	20	7601342	C	T	0.133	1.00	0.196	0.057	0.0006595	0.0306518		0.144	0.93	-0.00528	0.02632	0.840819
rs2064267	20	7602978	C	T	0.866	1.00	-0.198	0.057	0.0005037	0.0257354		0.855	0.93	0.001969	0.02612	0.9398532
rs2143197	20	7604210	C	G	0.867	1.00	-0.196	0.057	0.0006533	0.0306244		0.856	0.93	0.004835	0.02632	0.8541542
rs2143196	20	7604312	A	G	0.867	1.00	-0.196	0.057	0.0006529	0.0306186		0.856	0.93	0.004802	0.02632	0.855132
rs6038918	20	7605356	A	C	0.867	1.00	-0.196	0.057	0.0006486	0.0305021		0.856	0.93	0.004731	0.02633	0.8572603
rs4813944	20	10717877	C	T	0.442	1.00	0.111	0.039	0.003948	0.0009386		0.424	1.00	0.002469	0.01814	0.8916755
rs1321255	20	13087821	G	T	0.051	0.98	0.292	0.089	0.0009689	0.0045622		0.042	0.96	0.025287	0.04784	0.5968146
rs6033633	20	13089809	C	T	0.051	0.98	0.292	0.089	0.0009654	0.0045494		0.042	0.96	0.025301	0.04784	0.5966069
rs6041927	20	13090371	A	G	0.949	0.97	-0.3	0.089	0.0007126	0.0035696		0.958	0.95	-0.02544	0.04783	0.5945392
rs6041963	20	13152935	G	T	0.491	0.41	0.232	0.06	0.0001086	0.0008674		0.514	0.30	0.000295	0.03335	0.9929363
rs6109800	20	13217848	C	G	0.304	0.99	0.139	0.042	0.001015	0.0007461		0.311	0.97	0.015189	0.01993	0.4455791
rs6078976	20	13218765	C	T	0.850	1.00	-0.183	0.053	0.0005979	0.0093513		0.840	0.99	-0.0203	0.02469	0.410574
rs6074585	20	13223202	C	G	0.099	0.80	0.278	0.072	0.00011	0.0021906		0.097	0.68	0.027567	0.03667	0.4518268
rs1998313	20	13224909	C	T	0.305	0.96	0.136	0.043	0.001467	0.0008724		0.312	0.91	0.018898	0.02058	0.3580893
rs6078979	20	13227124	A	T	0.099	0.79	0.278	0.072	0.0001226	0.0023591		0.095	0.64	0.027502	0.03853	0.4749437
rs2618627	20	17728208	C	T	0.645	1.00	0.146	0.041	0.000312	0.0001026		0.668	0.99	-0.03905	0.01975	0.0478525
rs2745820	20	17728641	C	T	0.363	0.96	-0.15	0.041	0.000258	0.000089		0.343	0.95	0.040315	0.01999	0.0435393
rs2745821	20	17728921	G	T	0.355	1.00	-0.146	0.041	0.0003247	0.0001057		0.332	1.00	0.038673	0.01974	0.0498951
rs6080964	20	18011552	A	G	0.225	1.00	-0.156	0.046	0.000751	0.0029337		0.188	0.38	0.005629	0.03724	0.8797641
rs11299	20	18071022	C	T	0.086	0.93	0.287	0.072	0.0000738	0.0047512		0.069	0.99	0.045721	0.03437	0.1830681
rs6132035	20	18086397	A	G	0.914	0.92	-0.286	0.072	0.0000789	0.0049261		0.930	0.97	-0.04524	0.03451	0.1894808
rs12626093	20	18087042	A	C	0.914	0.92	-0.286	0.072	0.0000791	0.0049326		0.929	0.94	-0.04185	0.03499	0.2313096
rs13045938	20	18087552	A	G	0.086	0.92	0.284	0.073	0.00009	0.0052872		0.071	0.94	0.041529	0.03498	0.2347727
rs6045264	20	18153644	G	T	0.911	0.94	-0.252	0.071	0.0003589	0.0061593		0.928	0.80	-0.04163	0.03791	0.2717206
rs17807467	20	18436837	C	G	0.035	0.57	-0.412	0.142	0.003595	0.000484		0.028	0.39	-0.06129	0.08071	0.4472137

					GOYA QC		GOYA Overweight/control			GOYA BMI continuous	known	IARC QC		IARC results		
MARKER	chr	position	A1	A2	FREQ1	Rsqr	Beta	SE	p	p	gene name	freq1	Rsqr	in_beta	in_SE	in_p
rs6046614	20	20019634	C	T	0.728	0.93	-0.154	0.045	0.0006598	0.0000266		0.718	0.91	-0.03906	0.02128	0.0662159
rs6081871	20	20020205	A	G	0.278	0.94	0.149	0.044	0.0008211	0.0000311		0.298	0.90	0.043124	0.02111	0.0408941
rs2179683	20	20020939	A	C	0.270	0.99	0.136	0.044	0.001897	0.0000894		0.286	0.95	0.042694	0.02085	0.0404512
rs6081873	20	20023273	A	G	0.268	1.00	0.137	0.044	0.001713	0.0000696		0.273	0.97	0.038139	0.02092	0.0680894
rs6075613	20	20024863	C	T	0.732	1.00	-0.137	0.044	0.00171	0.0000689		0.727	0.98	-0.03806	0.02088	0.0681888
rs1474754	20	20025178	A	G	0.270	1.00	0.135	0.044	0.002036	0.0000948		0.286	0.97	0.042384	0.02071	0.0405984
rs1155188	20	20025897	A	G	0.274	0.99	0.138	0.043	0.001486	0.0000598		0.290	0.96	0.041953	0.02069	0.042492
rs4814937	20	20026767	A	C	0.712	0.97	-0.134	0.043	0.001986	0.0001643		0.709	0.95	-0.04229	0.02075	0.0413823
rs6046620	20	20028132	A	G	0.268	1.00	0.137	0.044	0.001736	0.0000673		0.272	1.00	0.037692	0.02072	0.0687615
rs2328491	20	20028492	C	T	0.732	1.00	-0.137	0.044	0.001747	0.0000686		0.728	1.00	-0.03775	0.02073	0.0684447
rs1040564	20	20030067	C	T	0.737	0.98	-0.141	0.044	0.001485	0.0000648		0.726	0.96	-0.03927	0.02101	0.0614326
rs1883798	20	20032496	A	G	0.268	1.00	0.137	0.044	0.001734	0.0000659		0.278	0.98	0.038718	0.02078	0.062197
rs17736770	20	21200862	A	G	0.972	0.57	-0.545	0.156	0.0004968	0.0018772		0.983	0.34	0.167135	0.10905	0.1250662
rs6076126	20	23700553	C	T	0.768	0.86	-0.144	0.049	0.003114	0.0002408		0.770	0.78	-0.01929	0.02424	0.4258663
rs6049096	20	23701523	A	T	0.301	0.96	0.13	0.043	0.002192	0.0008212		0.302	0.91	0.017246	0.02055	0.4009498
rs6132660	20	23711450	A	G	0.280	1.00	0.131	0.043	0.002078	0.0005464		0.287	0.99	0.015294	0.02012	0.4467269
rs6076129	20	23715335	C	G	0.280	1.00	0.131	0.043	0.002127	0.0005764		0.287	1.00	0.015226	0.0201	0.4484842
rs6036534	20	23722560	A	G	0.280	1.00	0.131	0.043	0.002041	0.0005437		0.287	1.00	0.015315	0.0201	0.4457574
rs6049130	20	23724643	C	T	0.249	0.91	0.135	0.046	0.003505	0.0004569		0.253	0.86	0.018052	0.02267	0.4254999
rs6083265	20	23724813	C	G	0.732	0.99	-0.126	0.043	0.003601	0.0005484		0.726	0.94	-0.01576	0.02095	0.4514747
rs6076130	20	23725114	C	T	0.280	1.00	0.132	0.043	0.001932	0.0005001		0.287	1.00	0.015603	0.0201	0.4372294
rs6049134	20	23727557	A	G	0.721	1.00	-0.132	0.043	0.001882	0.00048		0.713	1.00	-0.01584	0.0201	0.4301868
rs2071445	20	23805982	A	G	0.669	1.00	0.139	0.041	0.0007225	0.0023983		0.645	1.00	-0.0103	0.01925	0.5922927
rs2071443	20	23807753	C	T	0.669	1.00	0.139	0.041	0.0007263	0.0024161		0.644	0.99	-0.01037	0.01931	0.5910445
rs6037194	20	25620011	A	G	0.015	0.51	-0.721	0.237	0.002382	0.0006265		0.018	0.61	-0.0652	0.08092	0.4200269
rs17341431	20	29969782	A	G	0.892	0.98	0.209	0.063	0.000866	0.001003		0.896	0.92	0.029709	0.03127	0.3417309
rs4324393	20	32000494	A	G	0.070	0.99	-0.25	0.077	0.001191	0.0009592		0.085	0.98	0.005056	0.03293	0.8778927
rs976728	20	32001013	C	T	0.943	0.98	0.298	0.085	0.0004558	0.0010314		0.932	0.95	-0.00011	0.03733	0.9976622
rs6120450	20	32002073	C	T	0.930	0.99	0.249	0.077	0.0012	0.000968		0.915	0.98	-0.00518	0.03294	0.8750522
rs6088352	20	32004259	A	G	0.070	0.99	-0.249	0.077	0.001212	0.0009826		0.085	0.98	0.005329	0.03295	0.8714157
rs6059698	20	32221658	C	T	0.856	0.98	0.19	0.056	0.0007079	0.0016174		0.838	0.99	-0.00732	0.0245	0.7649231
rs17332951	20	32227844	C	T	0.056	1.00	-0.281	0.085	0.0009385	0.0018227		0.063	0.99	0.017253	0.0378	0.6477851
rs6059710	20	32244311	C	T	0.866	0.99	0.198	0.057	0.0005798	0.0012532		0.849	0.97	-0.01189	0.02527	0.6378053
rs6059711	20	32244483	C	T	0.869	0.99	0.194	0.058	0.000829	0.0021451		0.855	0.96	-0.00333	0.02595	0.8979325
rs2065912	20	32247148	G	T	0.892	0.99	0.226	0.064	0.0003745	0.0002905		0.875	0.98	-0.00922	0.02729	0.7351214
rs6087561	20	32247488	C	T	0.098	1.00	-0.264	0.066	0.0000658	0.0000739		0.115	0.99	0.006603	0.02813	0.8142386
rs12480555	20	32248155	A	G	0.902	1.00	0.271	0.066	0.0000414	0.0000492		0.885	0.99	-0.00655	0.02812	0.8157578
rs6088423	20	32251802	G	T	0.099	1.00	-0.272	0.066	0.0000368	0.0000446		0.115	0.99	0.006525	0.02812	0.8163255
rs6088425	20	32255181	A	T	0.099	1.00	-0.273	0.066	0.0000362	0.0000436		0.116	0.99	0.006405	0.02813	0.8197375
rs6120568	20	32257161	C	T	0.099	1.00	-0.273	0.066	0.0000355	0.0000427		0.116	0.99	0.006339	0.02814	0.8216332
rs12625272	20	32257822	C	G	0.901	1.00	0.273	0.066	0.0000346	0.0000414		0.884	0.99	-0.00629	0.02815	0.8230977
rs6088429	20	32262391	C	T	0.901	1.00	0.273	0.066	0.0000343	0.000041		0.884	0.98	-0.00623	0.02815	0.8248395
rs6059729	20	32270552	A	G	0.886	1.00	0.26	0.062	0.0000275	0.0001385		0.873	0.91	-0.00293	0.02828	0.91735
rs6119473	20	32272910	C	T	0.114	1.00	-0.26	0.062	0.0000273	0.0001373		0.127	0.91	0.002889	0.02827	0.918543
rs6087565	20	32285120	A	G	0.901	1.00	0.279	0.066	0.0000226	0.0000257		0.875	0.98	-0.00337	0.02763	0.9028426
rs819178	20	32289453	A	G	0.901	1.00	0.28	0.066	0.0000218	0.0000238		0.874	0.99	-0.00356	0.02746	0.8967266
rs6088443	20	32295792	C	T	0.886	1.00	0.265	0.062	0.0000197	0.000088		0.866	0.95	-0.00242	0.02743	0.9296712
rs819164	20	32303115	A	G	0.901	1.00	0.28	0.066	0.000021	0.0000203		0.873	1.00	-0.00373	0.02731	0.8911867
rs819163	20	32306018	G	T	0.114	1.00	-0.265	0.062	0.0000196	0.0000864		0.134	0.95	0.002324	0.02743	0.9324241
rs6120580	20	32308191	C	T	0.887	1.00	0.267	0.062	0.0000178	0.000078		0.866	0.95	-0.00228	0.02743	0.9337801
rs2378132	20	32309697	A	G	0.887	1.00	0.267	0.062	0.0000178	0.000078		0.866	0.95	-0.00205	0.02745	0.9404137
rs819136	20	32312380	A	G	0.098	1.00	-0.283	0.066	0.000019	0.0000179		0.127	1.00	0.002986	0.02735	0.9129826
rs819162	20	32315943	A	T	0.887	1.00	0.267	0.062	0.0000177	0.000078		0.866	0.95	-0.0016	0.02748	0.9535647
rs6088454	20	32318238	A	T	0.887	1.00	0.267	0.062	0.0000177	0.0000783		0.866	0.95	-0.0016	0.02748	0.9534887
rs819142	20	32324668	A	G	0.886	1.00	0.265	0.062	0.0000203	0.0001028		0.866	0.94	-0.00154	0.02752	0.955336
rs819144	20	32325217	G	T	0.886	1.00	0.265	0.062	0.0000203	0.0001032		0.866	0.94	-0.00142	0.02754	0.9587228
rs819145	20	32325488	G	T	0.886	1.00	0.264	0.062	0.0000205	0.0001045		0.866	0.94	-0.00138	0.02757	0.9599259
rs819177	20	32327964	C	G	0.114	1.00	-0.264	0.062	0.0000207	0.0001059		0.134	0.94	0.001296	0.02761	0.9625348
rs819176	20	32329505	G	T	0.886	1.00	0.264	0.062	0.0000208	0.0001067		0.866	0.94	-0.00127	0.02762	0.9632157
rs819175	20	32329842	C	G	0.886	1.00	0.264	0.062	0.0000209	0.0001081		0.866	0.93	-0.00122	0.02764	0.9646819
rs819173	20	32330223	C	T	0.114	1.00	-0.262	0.062	0.0000229	0.0001307		0.143	0.88	0.00041	0.02766	0.9881645
rs819172	20	32331167	C	T	0.892	0.99	0.265	0.064	0.0000319	0.0003019		0.862	0.85	0.006403	0.02841	0.8215435
rs819133	20	32333975	G	T	0.886	1.00	0.264	0.062	0.0000211	0.0001093		0.866	0.93	-0.0012	0.02765	0.965239
rs864702	20	32335211	A	G	0.114	1.00	-0.264	0.062	0.0000211	0.0001095		0.134	0.93	0.001094	0.0277	0.9684617
rs866027	20	32337971	A	G	0.886	1.00	0.264	0.062	0.0000212	0.0001099		0.866	0.93	-0.00109	0.0277	0.9685279
rs1205357	20	32341427	C	T	0.111	0.98	-0.253	0.063	0.000065	0.0004042		0.165	0.73	0.007793	0.0285	0.7843594
rs819159	20	32343381	A	T	0.114	0.99	-0.264	0.062	0.000021	0.0001084		0.134	0.93	0.001047	0.02771	0.9698223
rs819158	20	32344399	A	G	0.886	0.99	0.264	0.062	0.000021	0.000108		0.866	0.93	-0.00105	0.02771	0.969758
rs819156	20	32344684	A	C	0.114	0.99	-0.265	0.062	0.0000209	0.0001067		0.134	0.93	0.001027	0.02772	0.9704326
rs819148	20	32352198	A	G	0.886	0.99	0.266	0.062	0.0000202	0.0001013		0.866	0.92	-0.00096	0.02774	0.9724877
rs819147	20	32353365	C	T	0.114	0.98	-0.266	0.062	0.0000202	0.0001003		0.134	0.92	0.000951	0.02775	0.9726298
rs1205366	20	32353998	C	T	0.886	0.98	0.266	0.062	0.0000199	0.0000986		0.866	0.92	-0.00092	0.02776	0.9734065
rs819146	20	32354861	G	T	0.114	0.98	-0.267	0.063	0.0000196	0.0000953		0.135	0.91	0.000596	0.02788	0.982941
rs1205349	20	32363335	C	G	0.918	0.83	0.273	0.079	0.0005619	0.0004465		0.897	0.87	0.007526	0.0323	0.8156186
rs819167	20	32367348	A	G	0.066	0.86	-0.319	0.085	0.0001876	0.0001409		0.074	0.86	0.03087	0.03754	0.410442
rs819168	20	32367506	G	T	0.934	0.86	0.319	0.085	0.0001875	0.0001419		0.924	0.85	-0.02882	0.03749	0.4417725
rs843901	20	32368297	G	T	0.934	0.86	0.318	0.085	0.0001861	0.0001441		0.924	0.85	-0.02877	0.03749	0.4424207
rs819131	20	32371510	A	G	0.930	0.89	0.274	0.081	0.0007501	0.0011269		0.915	0.88	-0.01118	0.03527	0.7509853
rs17302686	20	32373966	A	G	0.069	0.97	-0.258	0.078	0.0009401	0.0032785		0.070	0.90	0.009354	0.03814	0.8060786
rs2275274	20	33320958	C	T	0.891	1.00	0.235	0.063	0.0001953	0.0047589		0.896	1.00	-0.02803	0.03017	0.3525226
rs6129410	20	34414796	A	G	0.018	0.61	-0.721	0.193	0.0001957	0.0102604		0.013	0.44	-0.04736	0.1084	0.6619338
rs6129926	20	34434692	C	T	0.018	0.62	-0.718	0.193	0.0001938	0.0108744		0.012	0.52	-0.05761	0.10527	0.5838943

					GOYA QC		GOYA Overweight/control			GOYA BMI continuous	known	IARC QC		IARC results		
MARKER	chr	position	A1	A2	FREQ1	Rsqr	Beta	SE	p	p	gene name	freq1	Rsqr	in_beta	in_SE	in_p
rs6124782	20	44389056	C	T	0.933	0.39	-0.478	0.125	0.0001359	0.000174		0.933	0.24	-0.02304	0.07168	0.7476834
rs4810497	20	44392172	C	G	0.933	0.39	-0.482	0.126	0.0001327	0.000161		0.934	0.23	-0.01249	0.07379	0.8654643
rs6124784	20	44394404	C	T	0.934	0.37	-0.498	0.129	0.0001079	0.0001259		0.934	0.21	-0.01078	0.07674	0.8882353
rs6067085	20	47458509	A	G	0.548	1.00	-0.113	0.039	0.003771	0.0002578		0.572	0.99	-0.01287	0.01853	0.4869962
rs6021473	20	49879228	C	T	0.971	1.00	0.392	0.119	0.0009566	0.0000968		0.974	0.96	-0.06399	0.05498	0.2440841
rs1996017	20	51356045	C	T	0.612	1.00	0.093	0.04	0.02124	0.0006247		0.652	1.00	-0.01889	0.01898	0.3192562
rs6070703	20	57055252	C	T	0.883	1.00	0.215	0.06	0.0003562	0.0030094		0.895	0.99	-0.0068	0.02881	0.8133312
rs6070710	20	57075577	G	T	0.075	0.71	-0.343	0.089	0.0001093	0.000541		0.083	0.70	0.026747	0.03929	0.4956217
rs11697681	20	57080816	C	G	0.075	0.71	-0.343	0.089	0.00011	0.0005434		0.083	0.70	0.026524	0.03931	0.4994467
rs6064744	20	57105922	C	T	0.910	0.70	0.284	0.081	0.0004641	0.0018338		0.910	0.69	0.003364	0.0377	0.9288299
rs7267048	20	57567610	A	G	0.400	0.85	0.15	0.043	0.0004269	0.0023494		0.435	0.78	-0.00163	0.02068	0.9372398
rs6064811	20	57572412	A	G	0.037	0.92	0.432	0.109	0.0000719	0.0028506		0.036	0.84	0.009726	0.0494	0.8437793
rs3848704	20	57574691	C	T	0.726	0.99	-0.164	0.044	0.000171	0.0068645		0.706	0.84	0.018438	0.02167	0.3943936
rs6128626	20	57575107	A	G	0.957	1.00	-0.334	0.096	0.0005261	0.0103766		0.956	0.98	-0.00745	0.04115	0.8563136
rs3848705	20	57575472	A	G	0.726	1.00	-0.163	0.043	0.0001743	0.0069383		0.708	0.87	0.018911	0.02135	0.3753494
rs3848706	20	57577582	A	C	0.274	1.00	0.163	0.043	0.0001781	0.0070203		0.292	0.87	-0.01892	0.02132	0.3743775
rs6015529	20	57578156	A	G	0.626	1.00	-0.159	0.04	0.0000658	0.0004446		0.591	0.89	0.019186	0.01959	0.3270083
rs4812097	20	57578349	C	T	0.726	1.00	-0.163	0.043	0.0001838	0.0071798		0.708	0.87	0.018995	0.0213	0.3720801
rs4812098	20	57578385	A	G	0.626	1.00	-0.158	0.04	0.0000682	0.0004582		0.591	0.89	0.019263	0.01959	0.3249741
rs4812104	20	57579362	A	C	0.275	0.99	0.162	0.043	0.0001899	0.0073397		0.292	0.87	-0.01916	0.02128	0.3676296
rs4812108	20	57579738	A	G	0.374	1.00	0.158	0.04	0.0000727	0.0004846		0.409	0.89	-0.01956	0.01957	0.3170082
rs6026972	20	57580906	G	T	0.359	1.00	0.134	0.04	0.0008693	0.0024071		0.409	0.97	-0.01265	0.0188	0.5008197
rs1105262	20	57582094	G	T	0.359	0.99	0.135	0.04	0.0008548	0.0023517		0.410	0.96	-0.01272	0.01882	0.498958
rs2426791	20	57595448	C	G	0.330	0.96	-0.105	0.042	0.01236	0.0002902		0.257	0.83	-0.03073	0.02287	0.1787315
rs6070854	20	57597045	A	G	0.331	0.97	-0.105	0.042	0.01201	0.0002734		0.257	0.83	-0.03081	0.02285	0.17718
rs6100518	20	57604101	C	G	0.335	0.98	-0.105	0.042	0.01143	0.0002001		0.264	0.86	-0.02634	0.02231	0.2374079
rs6026990	20	57611010	A	T	0.055	0.96	0.261	0.086	0.002493	0.0002833		0.075	0.91	-0.01359	0.03769	0.7181926
rs6026992	20	57611622	C	G	0.945	0.96	-0.263	0.086	0.00223	0.0002541		0.925	0.91	0.012578	0.03775	0.7387504
rs6026995	20	57615094	C	T	0.982	0.95	-0.509	0.152	0.0007995	0.0026787		0.989	0.82	0.129565	0.0891	0.1455946
rs6123918	20	57617254	G	T	0.945	0.96	-0.264	0.086	0.002173	0.000248		0.925	0.91	0.012456	0.03775	0.7411917
rs2145263	20	57620770	A	C	0.057	1.00	0.27	0.083	0.001177	0.0001556		0.083	0.87	-0.01705	0.03635	0.6387604
rs6128644	20	57621749	C	T	0.982	0.98	-0.5	0.149	0.0007925	0.0021302		0.989	0.82	0.128799	0.08915	0.1482263
rs6026998	20	57623030	A	G	0.057	1.00	0.274	0.083	0.001011	0.0001269		0.075	0.91	-0.01201	0.03775	0.7502096
rs6123921	20	57623552	A	G	0.943	1.00	-0.275	0.083	0.000983	0.000123		0.925	0.91	0.011982	0.03775	0.7507093
rs6026999	20	57624496	A	G	0.057	1.00	0.275	0.083	0.0009685	0.000121		0.075	0.91	-0.01196	0.03775	0.7512388
rs1555365	20	57626469	A	G	0.059	0.99	0.291	0.082	0.0003908	0.000062		0.083	0.92	0.003773	0.03612	0.9167337
rs6015534	20	57629819	A	G	0.356	1.00	0.118	0.04	0.003336	0.0007467		0.380	1.00	0.009213	0.01909	0.6291443
rs6064818	20	57636409	A	C	0.387	1.00	0.107	0.04	0.007022	0.0008179		0.401	1.00	-0.00885	0.01869	0.6357038
rs1736135	21	15727091	C	T	0.427	0.97	0.107	0.04	0.00692	0.0007222		0.396	0.96	0.003521	0.0188	0.8513227
rs1297258	21	15728580	C	T	0.573	0.97	-0.107	0.039	0.006866	0.0007094		0.604	0.96	-0.00354	0.01879	0.8504688
rs972010	21	15728900	C	T	0.428	0.98	0.106	0.039	0.006846	0.000703		0.396	0.97	0.003713	0.0187	0.8424716
rs1736018	21	15730091	A	G	0.572	0.98	-0.106	0.039	0.006841	0.0007021		0.604	0.98	-0.00377	0.01866	0.8399386
rs1736140	21	15731782	A	T	0.428	0.98	0.106	0.039	0.006843	0.0007009		0.396	0.98	0.003783	0.01866	0.8391745
rs991774	21	15732981	A	G	0.572	0.98	-0.106	0.039	0.006831	0.0006994		0.604	0.98	-0.00387	0.01861	0.8350521
rs1736144	21	15733353	C	G	0.428	0.98	0.106	0.039	0.00683	0.0006988		0.396	0.98	0.003883	0.0186	0.834511
rs1736145	21	15733867	C	T	0.428	0.99	0.106	0.039	0.006734	0.0006751		0.396	0.99	0.003977	0.01854	0.829991
rs1736020	21	15734423	A	C	0.428	0.99	0.106	0.039	0.006733	0.0006758		0.397	0.99	0.004006	0.01853	0.8286849
rs1297260	21	15734470	C	T	0.572	0.99	-0.106	0.039	0.006708	0.0006699		0.604	0.99	-0.00402	0.01852	0.828131
rs1736023	21	15734753	C	T	0.572	0.99	-0.106	0.039	0.006711	0.0006697		0.604	0.99	-0.00403	0.01851	0.8276652
rs1736147	21	15734924	A	G	0.428	0.99	0.106	0.039	0.006708	0.0006684		0.397	0.99	0.004028	0.01851	0.827549
rs1736148	21	15735083	C	T	0.428	0.99	0.106	0.039	0.006706	0.000668		0.397	1.00	0.004077	0.01848	0.8252503
rs7281316	21	19329198	A	C	0.235	0.65	-0.188	0.057	0.0009444	0.0213026		0.186	0.56	-0.04223	0.03034	0.1635242
rs952125	21	20020072	A	C	0.835	1.00	-0.18	0.052	0.0004765	0.0118265		0.899	1.00	0.046003	0.02899	0.1122276
rs8132987	21	23860188	A	G	0.352	0.96	-0.105	0.042	0.01201	0.0008703		0.363	0.95	0.002204	0.01989	0.911691
rs2828264	21	23864281	A	G	0.453	1.00	-0.103	0.039	0.008839	0.0004605		0.454	0.93	0.000532	0.01925	0.9779392
rs2828266	21	23865484	C	T	0.318	0.99	-0.114	0.042	0.006575	0.0003033		0.317	0.97	0.025859	0.02038	0.2041912
rs2828268	21	23866073	C	T	0.658	1.00	0.107	0.041	0.009155	0.0007768		0.645	0.99	-0.01384	0.01954	0.4784427
rs2828279	21	23871851	C	T	0.546	0.98	0.097	0.04	0.01399	0.0009363		0.529	0.89	-0.00433	0.01957	0.8247386
rs16999927	21	24761194	A	G	0.053	1.00	0.289	0.087	0.0009249	0.0014203		0.060	0.89	0.016658	0.0439	0.7041037
rs12483406	21	24765552	A	T	0.052	0.98	0.295	0.089	0.0008844	0.0014547		0.059	0.90	0.01415	0.04386	0.7467679
rs10482950	21	24768196	A	G	0.949	0.98	-0.297	0.089	0.0008781	0.0015028		0.941	0.90	-0.01379	0.04386	0.7530299
rs2830169	21	26613035	A	G	0.415	1.00	0.128	0.04	0.001198	0.0008144		0.391	1.00	0.030177	0.01839	0.100613
rs1888428	21	26615418	C	T	0.414	1.00	0.126	0.04	0.001449	0.000982		0.390	1.00	0.030806	0.0184	0.0938029
rs2212897	21	26626179	A	G	0.416	1.00	0.131	0.04	0.0009326	0.0006607		0.396	1.00	0.025573	0.01838	0.1637674
rs976105	21	26631318	C	G	0.772	0.99	-0.164	0.047	0.0004424	0.0003944		0.795	0.97	-0.00717	0.02266	0.7515945
rs2212898	21	26633077	A	T	0.769	1.00	-0.163	0.046	0.0004273	0.0004585		0.787	0.99	-0.01289	0.02214	0.5599124
rs7277841	21	26634048	A	G	0.231	1.00	0.163	0.046	0.0004269	0.0004581		0.213	0.99	0.012887	0.02214	0.5600948
rs10775636	21	26647771	C	T	0.865	1.00	-0.203	0.057	0.0003637	0.0024729		0.866	0.99	-0.0072	0.02644	0.7852001
rs2830406	21	26991578	A	G	0.981	0.33	0.901	0.256	0.0004308	0.0017489		0.975	0.35	-0.1056	0.09591	0.2704644
rs13052228	21	36133960	A	G	0.017	0.92	-0.553	0.165	0.0008197	0.0054636		0.019	0.92	0.075426	0.07207	0.2949056
rs9606527	22	15767645	A	C	0.670	0.72	0.166	0.049	0.0006755	0.0010613		0.666	0.62	0.018209	0.02441	0.4553882
rs174294	22	16374585	C	T	0.253	1.00	0.146	0.044	0.000979	0.0057154		0.245	0.99	0.018873	0.02102	0.3688233
rs174302	22	16377619	G	T	0.747	1.00	-0.149	0.044	0.0007875	0.0048816		0.755	0.99	-0.01883	0.02102	0.3699076
rs2330334	22	21693882	A	G	0.303	0.99	-0.11	0.042	0.009061	0.0003948		0.292	0.99	0.020717	0.02061	0.3144075
rs876101	22	21694370	A	G	0.300	1.00	-0.109	0.042	0.009506	0.0003947		0.290	1.00	0.021314	0.02058	0.3000054
rs5996449	22	21695301	A	G	0.697	1.00	0.11	0.042	0.008727	0.0003795		0.708	0.99	-0.02062	0.02059	0.3161157
rs6003472	22	21699826	C	T	0.700	1.00	0.11	0.042	0.008772	0.0003665		0.711	1.00	-0.02131	0.02058	0.2999897
rs8139695	22	21703902	A	T	0.718	0.98	0.104	0.043	0.01681	0.0005166		0.719	0.98	-0.01574	0.02104	0.4540178
rs5996454	22	21709206	A	G	0.716	0.99	0.11	0.043	0.01052	0.0002354		0.714	0.99	-0.00967	0.02088	0.6430016
rs5996455	22	21709255	A	G	0.282	0.99	-0.104	0.043	0.01662	0.0004466		0.281	0.99	0.014047	0.02097	0.5026818
rs6003482	22	21709362	A	G	0.716	0.99	0.11	0.043	0.01052	0.000234		0.714	0.99	-0.00963	0.02087	0.644145
rs6003483	22	21709625	C	T	0.282	0.99	-0.104	0.043	0.01662	0.0004432		0.281	0.99	0.013996	0.02097	0.5040476

					GOYA QC		GOYA Overweight/control			GOYA BMI continuous	known	IARC QC		IARC results		
MARKER	chr	position	A1	A2	FREQ1	Rsqr	Beta	SE	p	p	gene name	freq1	Rsqr	in_beta	in_SE	in_p
rs6003484	22	21709985	A	G	0.284	1.00	-0.11	0.043	0.01052	0.0002325		0.286	0.99	0.009605	0.02086	0.6449769
rs2078726	22	21712483	C	T	0.284	1.00	-0.11	0.043	0.01053	0.0002315		0.286	1.00	0.009556	0.02085	0.6465049
rs2078727	22	21715024	A	T	0.716	1.00	0.11	0.043	0.01054	0.0002303		0.714	1.00	-0.00955	0.02085	0.6468325
rs2078728	22	21715124	C	T	0.716	1.00	0.11	0.043	0.01055	0.0002299		0.714	1.00	-0.00952	0.02085	0.6477725
rs875602	22	21725684	C	T	0.716	1.00	0.11	0.043	0.01057	0.0002261		0.714	1.00	-0.0094	0.02083	0.6514994
rs6003495	22	21729743	A	G	0.716	1.00	0.11	0.043	0.01053	0.000224		0.714	1.00	-0.00958	0.02083	0.6452028
rs17443175	22	27818644	C	T	0.973	0.88	-0.372	0.128	0.003762	0.0006291		0.978	0.83	-0.08518	0.06873	0.2148725
rs5997591	22	28934667	A	C	0.979	0.72	0.58	0.164	0.0003997	0.0020154		0.979	0.48	-0.04509	0.09336	0.6288364
rs5753638	22	30199892	C	T	0.057	0.95	0.285	0.086	0.000932	0.0007286		0.064	0.95	-0.02533	0.03503	0.4693088
rs8136019	22	38401491	C	T	0.784	1.00	-0.121	0.047	0.01032	0.0008406		0.734	0.99	-0.01052	0.02085	0.6135157
rs3747179	22	38413676	G	T	0.215	1.00	0.127	0.047	0.007147	0.0007275		0.264	1.00	0.014552	0.02088	0.4855398
rs7289512	22	41126858	A	G	0.474	0.95	-0.131	0.04	0.0009995	0.0011302		0.480	0.90	0.021587	0.01921	0.2607709
rs13055479	22	45383771	A	G	0.046	0.81	0.342	0.104	0.001007	0.0007832		0.057	0.80	-0.03557	0.03825	0.3521043
rs735252	22	45899473	G	T	0.392	1.00	0.127	0.04	0.001513	0.0005111		0.377	1.00	0.019881	0.01862	0.2852618
rs5766697	22	45919956	A	G	0.592	0.97	-0.151	0.04	0.000185	0.0000282		0.624	0.93	-0.00639	0.01924	0.7394745
rs13057083	22	45922455	A	G	0.686	1.00	-0.165	0.042	0.0000879	0.000092		0.723	0.76	-0.02277	0.02272	0.3159761
rs2295249	22	45922607	C	T	0.408	0.97	0.15	0.04	0.0001928	0.0000294		0.376	0.93	0.006219	0.01923	0.746187
rs5766699	22	45923643	A	G	0.408	0.97	0.15	0.04	0.0001954	0.0000298		0.376	0.93	0.006121	0.01922	0.7499406
rs7292231	22	45924935	C	T	0.588	0.96	-0.153	0.04	0.000145	0.0000545		0.617	0.96	-0.00809	0.01896	0.6691815
rs5767536	22	45925806	A	G	0.401	0.99	0.146	0.04	0.0002299	0.0000676		0.376	0.98	0.005195	0.0187	0.7809426
rs738667	22	45926776	A	C	0.601	1.00	-0.145	0.04	0.0002556	0.0000779		0.627	1.00	-0.00485	0.01863	0.7943177
rs738669	22	45926985	C	T	0.399	1.00	0.145	0.04	0.0002524	0.0000766		0.373	1.00	0.004995	0.01864	0.7885633
rs5767541	22	45927202	A	G	0.596	0.97	-0.14	0.04	0.0005021	0.0000702		0.623	0.97	-0.00265	0.01885	0.8882041
rs738670	22	45927541	A	G	0.398	0.97	0.148	0.04	0.0002342	0.0000677		0.375	0.97	0.004774	0.01887	0.8000585
rs738671	22	45927613	A	G	0.596	0.96	-0.14	0.04	0.0005024	0.0000689		0.623	0.97	-0.0026	0.0189	0.8906205
rs4823601	22	45928487	A	G	0.422	0.94	0.152	0.041	0.0001774	0.0000788		0.391	0.94	0.008116	0.01905	0.6698309
rs6008046	22	45930191	C	T	0.763	0.81	-0.151	0.05	0.002659	0.0002544		0.772	0.82	0.004036	0.0247	0.8700736
rs5767545	22	45931285	A	G	0.482	0.99	-0.14	0.039	0.0003288	0.000315		0.494	0.98	0.003984	0.01829	0.8274425
rs5767547	22	45931701	C	G	0.393	1.00	0.142	0.04	0.0003563	0.0000616		0.372	0.99	0.008981	0.01879	0.6323786
rs6007611	22	45931914	C	T	0.622	0.97	-0.136	0.041	0.0008663	0.0002399		0.635	0.97	-0.01308	0.01902	0.4914098
rs910542	22	45935007	C	T	0.393	1.00	0.142	0.04	0.0003693	0.0000656		0.372	1.00	0.008957	0.01875	0.6326115
rs754892	22	45935170	A	C	0.483	1.00	-0.138	0.039	0.0003743	0.0003656		0.495	1.00	0.004225	0.01816	0.8158913
rs761739	22	45935887	C	G	0.607	1.00	-0.142	0.04	0.0003698	0.000066		0.628	1.00	-0.00892	0.01874	0.6337685
rs4823471	22	45936073	C	T	0.393	1.00	0.142	0.04	0.0003691	0.000066		0.372	1.00	0.008904	0.01874	0.6343409
rs4823472	22	45936141	C	G	0.640	0.92	-0.156	0.042	0.0002025	0.0000475		0.656	0.92	-0.01331	0.01988	0.5029392
rs4823603	22	45936302	A	G	0.607	1.00	-0.142	0.04	0.0003693	0.0000663		0.628	1.00	-0.00888	0.01873	0.6349746
rs4823473	22	45936542	A	G	0.517	1.00	0.138	0.039	0.0003774	0.0003689		0.505	1.00	-0.00427	0.01814	0.8138946
rs5005651	22	45936699	A	G	0.483	1.00	-0.138	0.039	0.0003864	0.0003782		0.495	1.00	0.004263	0.01814	0.8140816
rs17763276	22	45936770	C	T	0.518	1.00	0.138	0.039	0.0003901	0.000382		0.505	1.00	-0.00426	0.01815	0.8144348
rs4823605	22	45937348	C	T	0.519	1.00	0.134	0.039	0.0005499	0.0005565		0.507	1.00	-0.0042	0.0182	0.8172158
rs5767553	22	45938113	G	T	0.519	1.00	0.134	0.039	0.0005585	0.0005655		0.507	1.00	-0.00419	0.0182	0.8175724
rs5766701	22	45938461	A	G	0.481	1.00	-0.134	0.039	0.0005747	0.0005787		0.493	0.99	0.004247	0.0182	0.8153804
rs5766705	22	45940011	C	G	0.618	1.00	-0.137	0.04	0.0006376	0.0001765		0.632	0.98	-0.01058	0.01894	0.576202
rs5766706	22	45940131	A	G	0.438	0.98	0.113	0.039	0.004119	0.000946		0.412	0.83	0.015908	0.02015	0.4294283
rs4823608	22	45941661	C	T	0.615	0.99	-0.142	0.04	0.0004043	0.000091		0.628	1.00	-0.00766	0.01877	0.68295
rs738672	22	45944546	C	G	0.397	0.91	0.133	0.042	0.001378	0.0002584		0.388	0.84	0.007219	0.0203	0.7219173
rs2017931	22	45944915	C	T	0.603	0.91	-0.133	0.042	0.001392	0.0002608		0.612	0.83	-0.00733	0.02044	0.7198338
rs5767849	22	46400684	A	C	0.584	0.94	0.147	0.041	0.0003159	0.0152499		0.581	0.94	-0.00406	0.019	0.8306579
rs11705127	22	46402482	C	T	0.420	0.94	-0.149	0.041	0.0002554	0.0113774		0.423	0.93	0.003515	0.01905	0.8534431
rs6008438	22	46658741	A	G	0.472	0.98	0.12	0.039	0.002302	0.0005404		0.427	0.97	0.002898	0.01878	0.877219
rs2338258	22	46662689	C	T	0.288	1.00	-0.151	0.043	0.0004742	0.0007554		0.294	1.00	0.010418	0.01998	0.6018351
rs1884517	22	46704229	A	G	0.297	0.99	-0.132	0.042	0.001838	0.0001564		0.311	0.98	-0.02646	0.01981	0.1812311
rs5768182	22	46704575	A	G	0.703	0.99	0.132	0.042	0.001825	0.0001565		0.689	0.98	0.026525	0.0198	0.1800393
rs715609	22	46705477	C	T	0.719	1.00	0.123	0.043	0.004244	0.0004442		0.706	1.00	0.021611	0.02001	0.2798578
rs10433324	22	46714536	C	T	0.282	0.97	-0.125	0.043	0.004195	0.00043		0.297	0.92	-0.02397	0.0207	0.2465724
rs6007848	22	47194416	A	C	0.557	0.97	-0.106	0.04	0.007287	0.0008284		0.523	0.72	0.001491	0.02133	0.9442522
rs11090839	22	47194900	G	T	0.434	0.99	0.105	0.039	0.007477	0.0009975		0.463	0.74	-0.00161	0.02114	0.9391102
rs11705307	22	47917704	A	G	0.850	0.99	-0.146	0.054	0.006945	0.0007638		0.849	0.97	0.006318	0.02665	0.8124214
rs743782	22	48164218	C	T	0.944	0.62	0.24	0.108	0.02672	0.0008893		0.939	0.62	-0.03813	0.05018	0.4469818

A1 denotes the effect allele, A2 denotes the other allele. Freq denotes the allele frequency of the effect allele. Rsqr denotes the imputation quality score
