# Supplementary material for: Genome-Wide Population-Based Association Study of Extremely Overweight Young Adults – The GOYA Study
Source: PLoS One. 2011 Sep 15;6(9):e24303. doi: 10.1371/journal.pone.0024303 (PMC3174168; doi:10.1371/journal.pone.0024303)
Supplement: Table S3 — Reasons for including each SNP in the stage 2 replication. (PDF) [file pone.0024303.s006.pdf]

SNP name	Gene	GOYA beta	GOYA CACO p	GOYA LinReg p	Brennan beta	Brennan p	Genesniffer	Evidence
rs699363	RGS6	-	0.003	0.0007	-0.204	0.0008		
rs1912967	TLE3	-	0.018	0.0007	-0.067	0.0021		
rs10102742	ADCY8	-	0.0007	0.0024	-0.057	0.0021		
rs12130212	CAMK1G	+	0.003	0.0005	0.064	0.0046		
rs4765158	NCOR2	+	0.0008	0.001	0.075	0.0053		
rs7542125	KLH12	+	0.0009	0.0003	0.059	0.0065		
rs1420533	TNCR9	+	0.008	0.0003	0.049	0.0068		
rs9532670	ELF1	-	0.001	0.0025	-0.051	0.0091		
rs970843	FLJ39743	-	0.003	0.0009	-0.071	0.0091		
rs32930	CENTD3	-	0.00004	0.0002	-0.052	0.0095		
rs734597	TFAP2B	-	0.00002	0.000007	-0.037	0.0918	✓	adiposity
rs876338	CXCR4	+	0.0009	0.00002	0.02	0.2898	✓	t2 diabetes
rs12938476	CRHR1	-	0.00004	0.00004	-0.029	0.1214	✓	satiety/obesity
rs582683	PTPRM	-	0.0002	0.00004	-0.016	0.4525		bmi in t2 diabetes patients - unpublished*
rs9566498	FOX01A	+	0.0004	0.00004	0.075	0.013	✓	insulin sensitivity
rs10987417	LMX1B	-	0.000005	0.00005	-0.016	0.4116		bmi - supp data Thorleifsson**
rs12155554	SLC30A8	+	0.00003	0.0001	0.033	0.4521	✓	t2 diabetes
rs365760	MMP2	-	0.00003	0.0007	-0.005	0.8137	✓	obesity
rs17241549	SIM1	+	0.00004	0.0001	0.001	0.9894	✓	obesity
rs17504169	CLDN16	-	0.0001	0.000003	-0.025	0.3838		
rs791903	IHPK3	+	0.0005	0.000005	0.022	0.2395		
rs7720663	???	-	0.0002	0.000006	-0.028	0.1378		
rs4964926	MMP17	-	0.00004	0.000007	-0.006	0.8581		
rs8067056	MAPT	-	0.00004	0.000008	-0.014	0.4994		
rs10047878	DAD1	-	0.000003	0.00005	-0.011	0.5524		
rs17104665	TCERG1	+	0.000005	0.00001	0.014	0.7086		
rs6571507	???	-	0.000008	0.00008	-0.013	0.4925		
rs6758546	AKAP6	+	0.000009	0.00009	0.017	0.5818		

* The online thesis of Market Sjogren (http://lup.lub.lu.se/luur/download?func=downloadFile&recordOId=1267326&fileOId=1267491) refers to unpublished DGI data where PTPRM SNPs are associated with BMI in T2D patients (rs644399 p=2x10-5, rs4121619 p=0.00028)

**An nearby LMX1B SNP (rs867559) appears in the supplementary table of Thorleifsson et al 2009 (Nature Genetics 41:18-24) showing preliminary association with BMI, $p=7x10^{-6}$

shaded cells indicate the evidence that led to the inclusion of each SNP in the replication set.

For GOYA 'beta' denotes the direction of effect (sign of the beta coefficient), which is the same for both the overweight/control (CACO) and the continuous BMI z-score linear regression (LinReg) analyses
