# Supplementary material for: Genome-Wide Population-Based Association Study of Extremely Overweight Young Adults – The GOYA Study
Source: PLoS One. 2011 Sep 15;6(9):e24303. doi: 10.1371/journal.pone.0024303 (PMC3174168; doi:10.1371/journal.pone.0024303)
Supplement: Text S1 — Stage 1 Replication—IARC participants, genotyping and analysis; GeneSniffer methods; Stage 2 Replication—Danish Cohorts participants, genotyping and analysis; and Stage 2 Replication—ALSPAC participants, genotyping and analysis. (DOC) [file pone.0024303.s007.doc]

**Supplementary Material**

**Stage 1 Replication - IARC Participants, Genotyping and Analysis**

The International Agency for Research on Cancer (IARC) participants were recruited between 1999 and 2002 in 15 centres across 6 countries including the Czech Republic (Prague, Olomouc, Brno), Hungary (Borsod, Heves, Szabolcs, Szolnok, Budapest), Poland (Warsaw, Lodz), Romania (Bucharest), Russia (Moscow) and Slovakia (Banska Bystrica, Bratislava, Nitra). Each centre followed identical protocols and questionnaires and recruited all newly diagnosed lung, head and neck, and kidney cancer cases. Controls (except in Warsaw) were selected among subjects admitted to the same hospital as the cases, and had conditions unrelated to tobacco consumption. In Warsaw, the controls were selected randomly using the Polish Electronic List of Residents. All subjects were interviewed by trained personnel and were questioned on demographic information (including age, sex, marital status, education, height, weight 2 years prior to interview, weight at age 20), detailed smoking history and diet information. Blood samples were taken at the time of interview and DNA extracted at IARC. Written consent was obtained for each participant, and ethical approval was obtained at each study centre as well as at the IARC [1].

Genome-wide genotyping on the Illumina 317k chip was carried out at the Centre National de Génotypage (CNG), Evry, France. SNPs which were selected for in silico replication and not present on the 317k chip were imputed with MACH software using CEU individuals from HAPMAP2 as the reference set. Individuals showing <70% European ancestry were excluded.

Using ProbAbel software from the ABEL set of programs (<http://mga.bionet.nsc.ru/yurii/ABEL>) [2], we ran a linear regression of the inverse rank normal BMI, adjusted for age sex and country.

(N.B - Country was evaluated to be the best way to deal with stratification in the data. No eigenvectors were significant in the null model of inverse rank normal BMI ~ country + age + sex. Lambda for the genome-wide analysis was 1.02 indicating little population stratification).

**GeneSniffer Methods**

For each gene, GeneSniffer downloaded appropriate webpages from the NCBI’s Gene, OMIM and PubMed databases and interrogated the text using a list of obesity/BMI specific words (assigned a score between 1 and 10 depending on significance). Homologues of each gene were identified by BLAST, interactants identified from the BIND and HPRD databases, the mouse orthologues were identified from Homologene and pages downloaded from Jackson's MGI database and these pages were also scored. A cumulative hitscore was calculated from all of the collected information and candidacy evidence was determined as a hitscore of >1000 for a gene within 0.1cM of the SNP.

**Stage 2 Replication - Danish Cohorts Participants, Genotyping and Analysis**

Subjects were included from six studies. 1) Inter99: includes 6,360 individuals from a population-based randomized non-pharmacological intervention study for prevention of cardiovascular disease conducted at the Research Centre for Prevention and Health in Copenhagen [3]. 2) Health06: includes 3,229 individuals from a population-based study of the prevalence and causes of cardiovascular disease, diabetes, allergy and asthma [4]. 3) SDC1: includes 681 unrelated non-diabetic middle-aged individuals from a population-based sample recruited at Steno Diabetes Center. 4) SDC2: includes 2,038 patients with type 2 diabetes recruited at the out-patient clinic at Steno Diabetes Center. 5) ADDITION Denmark screening sample: includes 8,602 individuals from a high-risk screening, and intervention study for type 2 diabetes recruited from Department of General Practice at the University of Aarhus [5]. 6) 377 young healthy individuals recruited from the Research Centre for Prevention and Health [6]. All individuals were recruited from the Copenhagen area of Denmark and all self-reported Danish nationality. Informed written consent was obtained from all subjects before participation. The study was approved by the Ethical Committee of Copenhagen County and was in accordance with the principles of the Helsinki Declaration.

Height and weight were measured in light indoor clothes and without shoes in all studies. Characteristics of the subjects in each of the six studies are shown in **Table S1**.

The variants were genotyped using allele-specific PCR (KASPar) (KBioscience, Hoddesdon, U.K.) with genotype success rates above 96.7% and error rates below 0.6 % in 1,369 duplicate samples. All variants were in Hardy-Weinberg equilibrium except rs4964926 (p = 0.006).

The data was analysed using linear regression of the sex-specific z-scores (adjusting for age) using RGui version 2.7.0 and reporting only the direction of effects of the biased beta coefficients.

**Stage 2 Replication - ALSPAC Participants, Genotyping and Analysis**

The Avon Longitudinal Study of Parents and their Children (ALSPAC) is a longitudinal population-based birth cohort that recruited pregnant women residing in Avon, UK, with an expected delivery data between 1^st^ April 1991 and 31^st^ December 1992. 14,541 pregnant women were initially enrolled with 14,062 children born (see [7] and website http://www.alspac.bris.ac.uk). Biological samples including DNA have been collected for 10,121 of the children from this cohort. Ethical approval was obtained from the ALSPAC Law and Ethics committee and relevant local ethics committees, and written informed consent provided by all parents.

Pertinent to the current study, the children were invited to a clinic when they were 15 years old, where height and weight measures were used to calculate BMI. Genotype data for the relevant SNPs were extracted from genome-wide data (imputed to HapMap release 22) that were available for 3,233 of the ALSPAC participants. Participants were genotyped using either Illumina 317K or 610K genome-wide SNP genotyping platforms by the Wellcome Trust Sanger Institute, Cambridge, UK and the Centre National de Génotypage, Evry, France. A common set of SNPs (present in both genotyping platforms) were extracted and the resulting raw genome-wide data was subjected to standard quality control methods. Individuals were excluded on the basis of having incorrect gender assignments; minimal (0.34) or excessive (0.36) heterozygosity; disproportionate levels of individual missingness (>3%) and evidence of cryptic relatedness (PI HAT > 0.11). The remaining individuals were assessed for evidence of population stratification by multidimensional scaling analysis, using CEU, Yoruba, Japanese and Chinese individuals as reference ethnic groups. The underlying population stratification was thereafter controlled for by using EIGENSTRAT derived ancestry informative covariates. SNPs with a minor allele frequency of < 0.5% and call rate of < 97% were removed. Furthermore, only SNPs which passed an exact test of Hardy-Weinberg equilibrium (P > 5E-7) were considered for analysis. The resulting dataset consisted of 3233 individuals and 285,531 SNPs. Missing genotypes were subsequently imputed with MACH 1.0 Markov Chain Haplotyping software, using CEPH individuals from phase two of the HapMap project as a reference set (release 22). The final imputed dataset consisted of 3233 subjects, each with 2,483,534 imputed markers. Only imputed genotypes with minor allele frequencies >1% and R-sqr >0.3 were considered for replication analysis.

2,418 adolescents with both BMI and genome-wide SNP data were available. We calculated sex specific BMI z-scores and carried out linear regression of z-score BMI on expected SNP dosage in MACH2QTL, adjusting for the appropriate EIGENSTRAT values.

**Supplementary References**

1. Scélo G, Constantinescu V, Csiki I, Zaridze D, Szeszenia-Dabrowska N, et al. (2004) Occupational exposure to vinyl chloride, acrylonitrile and styrene and lung cancer risk (europe). Cancer causes & control : CCC 15: 445-52. doi:10.1023/B:CACO.0000036444.11655.be

2. Aulchenko YS, Ripke S, Isaacs A, Duijn CM van (2007) GenABEL: an R library for genome-wide association analysis. Bioinformatics (Oxford, England) 23: 1294-6. doi:10.1093/bioinformatics/btm108

3. Jørgensen T, Borch-Johnsen K, Thomsen TF, Ibsen H, Glümer C, et al. (2003) A randomized non-pharmacological intervention study for prevention of ischaemic heart disease: baseline results Inter99. European journal of cardiovascular prevention and rehabilitation : official journal of the European Society of Cardiology, Working Groups on Epidemiology & Prevention and Cardiac Rehabilitation and Exercise Physiology 10: 377-86. doi:10.1097/01.hjr.0000096541.30533.82

4. Hersoug L-G, Husemoen LLN, Sigsgaard T, Madsen F, Linneberg A (2010) Indoor exposure to environmental cigarette smoke, but not other inhaled particulates associates with respiratory symptoms and diminished lung function in adults. Respirology (Carlton, Vic.) 15: 993-1000. doi:10.1111/j.1440-1843.2010.01758.x

5. Lauritzen T, Griffin S, Borch-Johnsen K, Wareham NJ, Wolffenbuttel BH, et al. (2000) The ADDITION study: proposed trial of the cost-effectiveness of an intensive multifactorial intervention on morbidity and mortality among people with Type 2 diabetes detected by screening. International journal of obesity and related metabolic disorders : journal of the International Association for the Study of Obesity 24 Suppl 3: S6-11.

6. Clausen JO, Borch-Johnsen K, Ibsen H, Bergman RN, Hougaard P, et al. (1996) Insulin sensitivity index, acute insulin response, and glucose effectiveness in a population-based sample of 380 young healthy Caucasians. Analysis of the impact of gender, body fat, physical fitness, and life-style factors. The Journal of clinical investigation 98: 1195-209. doi:10.1172/JCI118903

7. Golding J, Pembrey M, Jones R (2001) ALSPAC-the avon longitudinal study of parents and children. I. study methodology. Paediatric and Perinatal Epidemiology 15: 74–87. doi:10.1046/j.1365-3016.2001.00325.x
